# Bacterial Infection Induces Ultrastructural and Transcriptional Changes in the King Oyster Mushroom (*Pleurotus eryngii*)

**DOI:** 10.1128/spectrum.01445-22

**Published:** 2022-05-26

**Authors:** Qi Gao, Yu Liu, Jianbo Xie, Shuang Zhao, Wentao Qin, Qinggang Song, Shouxian Wang, Chengbo Rong

**Affiliations:** a Institute of Plant Protection, Beijing Academy of Agriculture and Forestry Sciences, Beijing Engineering Research Center for Edible Mushroom, Beijing, China; b National Engineering Laboratory for Tree Breeding, College of Biological Sciences and Technology, Beijing Forestry University, Beijing, China; Yeungnam University

**Keywords:** *Pleurotus eryngii*, soft rot disease, transcriptome, pathogen, reactive oxygen species, cell wall, *Erwinia beijingensis*

## Abstract

*Pleurotus eryngii* (king oyster mushroom) is a commercially important mushroom with high nutritional and economic value. However, soft rot disease, caused by the pathogenic bacterium *Erwinia beijingensis,* poses a threat to its quality and production. Morphological and ultrastructural observations of *P. eryngii* were conducted at early, middle, and late stages of infection. At 2 days postinoculation (dpi), small yellow spots on the fruiting body were observed. The infected tissue displayed hyphal deformations and breaks at 5 dpi. At 9 dpi, damage to cell wall integrity and absence of intact cellular organelles were observed and the diseased fruiting bodies were unable to grow normally. Transcriptome analysis identified 4,296 differentially expressed genes in the fruiting body following infection. In fact, broad transcriptional reprogramming was observed in infected fruiting bodies compared to controls. The affected pathways included antioxidant systems, peroxisome biogenesis, autophagy, and oxidation-reduction. More specifically, *pex* genes were downregulated during infection, indicating impaired peroxisome homeostasis and redox balance. Additionally, genes encoding chitinase, β-1,3-glucanase, and proteases associated with cell wall degradation were upregulated in infected *P. eryngii*. This study provides insights into the responses of *P*. *eryngii* during soft rot disease and facilitates the understanding of the pathogenic process of bacteriosis in mushrooms.

**IMPORTANCE** *Pleurotus eryngii* (king oyster mushroom) is a popular and economically valuable edible mushroom; however, it suffers from various bacterial diseases, including soft rot disease caused by the bacterium *Erwinia beijingensis*. Here, we examined bacterial infection of the mushroom through morphological and ultrastructural observations as well as transcriptome analysis. Pathogen attack damaged the cell structure of *P. eryngii*, including the cell wall, and also induced high levels of reactive oxygen species. These results were reflected in differential gene expression in *P. eryngii* as a response to the pathogenic bacteria, including genes involved in antioxidant systems, peroxisome biogenesis, autophagy, oxidation-reduction, ribosome biogenesis, and cell-wall degradation, among others. This study provides insights into the structural and molecular responses of *P. eryngii* during soft rot disease, improving our understanding and the potential control of the pathogenic process of bacteriosis in mushrooms.

## INTRODUCTION

*Pleurotus eryngii* (king oyster mushroom) is one of the most popular edible mushrooms owing to its delicious taste, excellent texture, and high nutrient value (1, 2). In East Asia, *P. eryngii* is widely and industrially cultivated on a large scale, with annual production exceeding 1 million tons in China. However, diseases of *P. eryngii* limit commercial cultivation, resulting in significant economic loss. Most notably, the yeast species *Sporobolomyces symmetricus* induces red spot disease (3), the bacterium *Pantoea pleuroti* causes blight disease (4), the fungus *Cladobotryum mycophilum* causes cobweb disease (5), and the Gram-negative pathogenic bacterium *Pantoea beijingensis* (recently renamed *Erwinia beijingensis*) (6) causes soft rot disease in *P. eryngii* (7).

Among the diseases affecting *P. eryngii*, soft rot disease is responsible for the most significant economic losses in China. The symptoms of this disease include yellow spots and water-soaked lesions, which can extend to the stipe and pileus; thereafter, soft rot reaches the fruiting body, effectively inhibiting the normal growth of the mushroom (7, 8). Prevention and control of soft rot are generally achieved via strict hygiene practices within production areas. However, even with these measures in place, *E. beijingensis* continues to cause soft rot outbreaks in *P. eryngii* crops throughout China. Thus, investigations aimed at advancing our current understanding of how *P. eryngii* responds to *E. beijingensis* infection may facilitate the development of strategies to control rot disease outbreaks.

Although research examining the response of mushroom fruiting bodies to pathogen infections is limited, select studies have reported important information in this regard. For instance, one study identified differentially expressed genes (DEGs) in the fruiting bodies of *Flammulina velutipes* experiencing blight disease, following infection with Arthrobacter arilaitensis and Pseudomonas yamanorum, which were significantly enriched in xenobiotic metabolism via cytochrome P450 and tyrosine metabolism (9). Additionally, genes associated with the chitin deacetylase pathway were upregulated by nearly 1,000-fold in *Agaricus bisporus* during infection with *Lecanicillium fungicola* (10). Meanwhile, in *A. bisporus* exhibiting symptoms of brown pileus following infection with mushroom virus X, an increase in the abundance of serine proteases associated with melanin biosynthesis and brown tissue formation, as well as increased expression of a ribosomal protein gene associated with a delay in primordium formation, were reported (11). Alternatively, the metabolic functions determined to be most highly associated with the downregulated genes in *A. bisporus* included protein synthesis/processing and turnover, membrane function and architecture, and nucleic acid binding (11). Bacterial blotch is one of the most economically important diseases in the cultivation of *A. bisporus*, and the activation of tyrosinase is a specific response toward the pathogen Pseudomonas tolaasii (12, 13).

In contrast to the minimal information available regarding the response of mushroom species to pathogens, plants have a defined complex network that becomes activated following infections. This system includes innate immunity, multiple signaling pathways, secondary metabolite products, and cellular cross talk (14). Specifically, plants respond to pathogenic infections by increasing the production of reactive oxygen species (ROS), which serve as the first line of defense and function as secondary messengers to signal subsequent defense reactions (15). In the infection of strawberries by Botrytis cinerea, for example, transcriptional changes resulted in broad transcriptional reprogramming in both unripe and ripe fruits involving receptors and signaling, secondary metabolites, and defense response pathways (16). However, these processes, specifically in the context of *P. eryngii* infected with soft rot disease pathogens, are poorly characterized.

Hence, in the current study, we investigated the response of *P. eryngii* throughout the course of soft rot disease caused by infection with *E. beijingensis*. We hypothesized that *P. eryngii* fruiting bodies accumulate ROS as the first line of defense following infection with *E. beijingensis*, similar to that observed in plants, and that transcriptional changes occur during this process, leading to the characteristic morphological and ultrastructural changes associated with soft rot. To this end, we analyzed the morphology and ultrastructure of *P. eryngii*, as well as ROS levels following infection with *E. beijingensis*. Transcriptome sequencing and analysis were also employed to identify and characterize changes in gene expression associated with the responses of *P. eryngii* during the early stages of *E. beijingensis* infection through to late stages. The findings of this study have the potential to facilitate the understanding of the pathogenic process of bacteriosis in mushrooms and can be exploited to help control soft rot disease via genetic intervention and/or development of resistance markers to assist *P. eryngii* breeding programs.

## RESULTS

### Morphological and ultrastructural changes occur in *P. eryngii* postinoculation.

**FIG 1:**
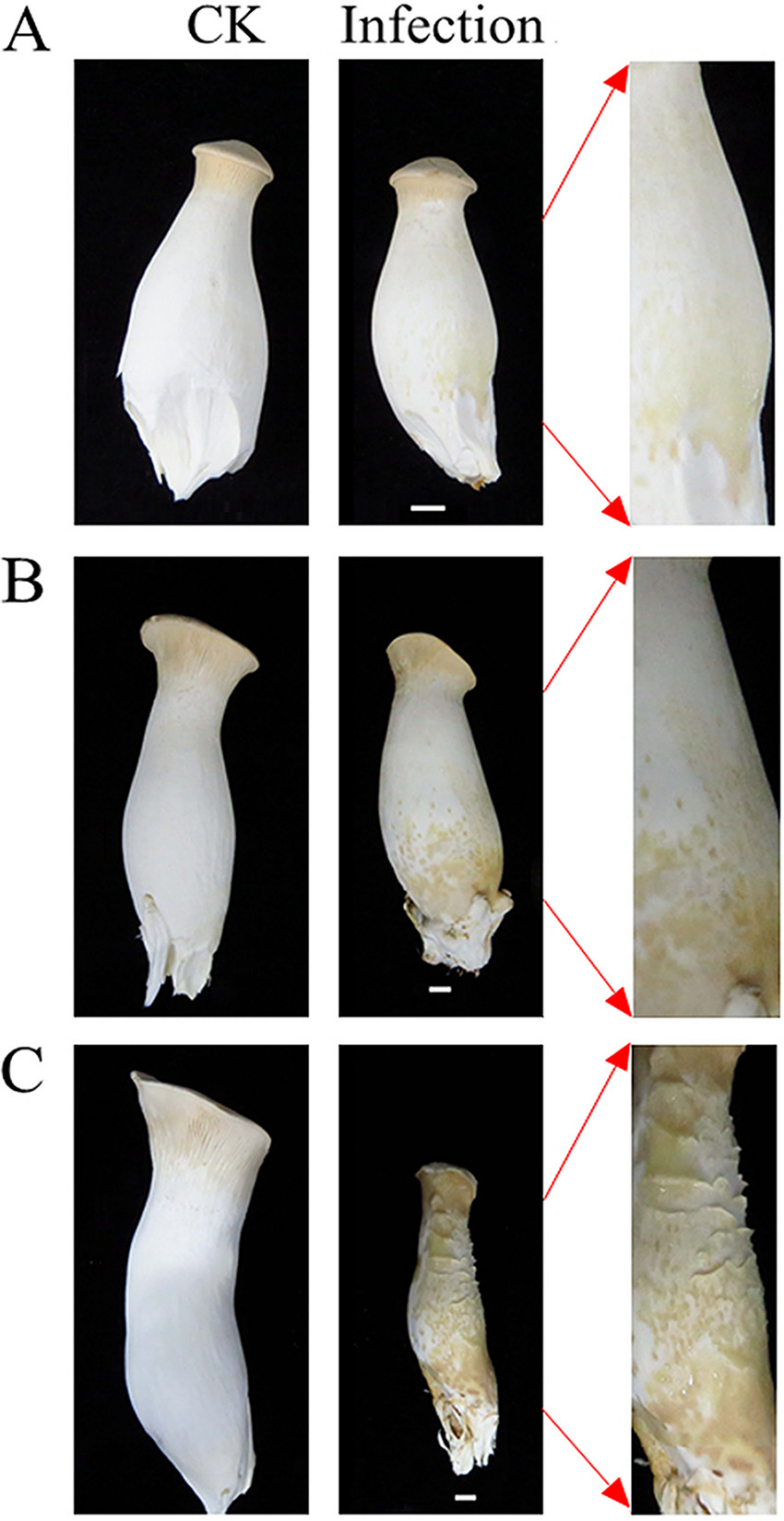
*Pleurotus eryngii* mushrooms infected with *Erwinia beijingensis*. Left, control group; right, infected group. (A) Control group (CK) and infected fruiting bodies at 2 dpi. (B) CK and infected fruiting bodies at 5 dpi. (C) CK and infected fruiting bodies at 9 dpi. Scale bar = 1 cm. Arrows indicate enlarged views of the infected fruiting bodies.

The fruiting bodies of the control group remained healthy and white throughout the experimental period. Two days after inoculating the young fruiting bodies of *P. eryngii* with *E. beijingensis*, symptoms of infection were visible as small yellow spots and light yellow water-soaked lesions on the fruiting bodies. Only a few of the total fruiting bodies were infected at the early stage (2 dpi). Meanwhile, at 5 dpi (middle-stage), infection symptoms became more severe; yellow water-soaked lesions and soft rot were observed in the stipes, and half of the fruiting bodies displayed lesions. The symptoms became most severe at 9 dpi (late-stage) with the entire fruiting body completely rotten and filled with yellow mucus; these diseased fruiting bodies were unable to grow normally (Fig. 1).

**FIG 2:**
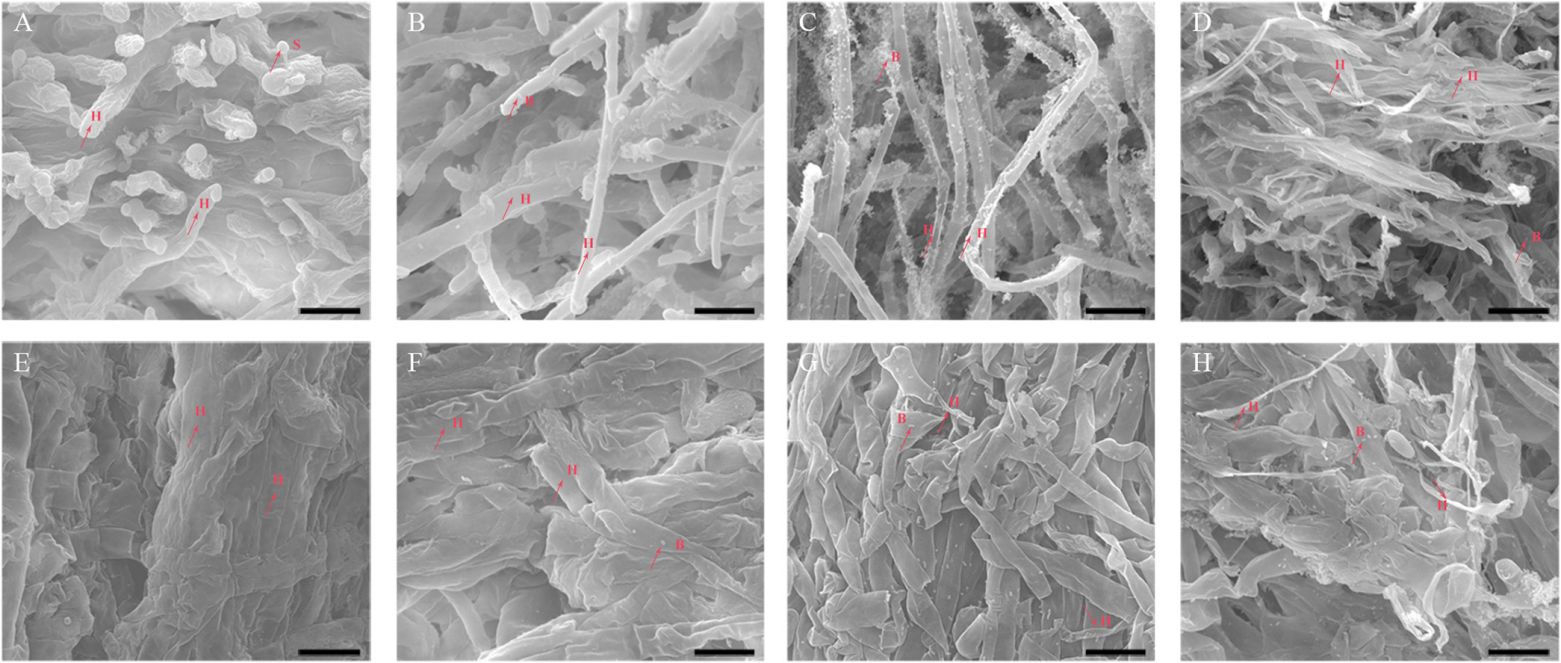
Scanning electron microscopy images of *Pleurotus eryngii* control and *Erwinia beijingensis*-infected fruiting bodies. (A) and (E) Surface and inner layer of fruiting bodies in the control group. (B) and (F) Surface and inner layer of infected fruiting bodies 2 dpi. (C) and (G) Surface and inner layer of infected fruiting bodies 5 dpi. (D) and (H) Surface and inner layer of infected fruiting bodies 9 dpi. Scale bar = 10 μm. H: hyphae; S: spore; B: bacteria.

**FIG 3:**
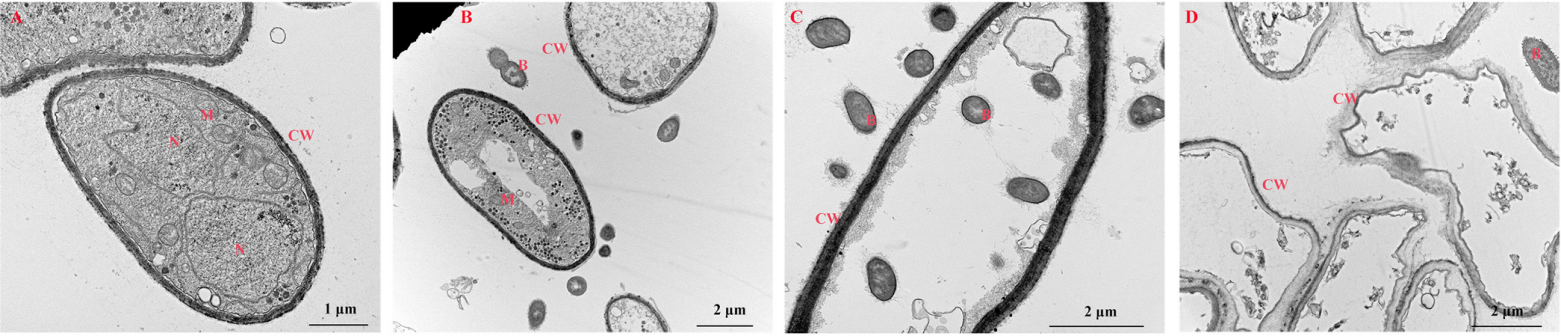
Transmission electron microscopy ultrastructural images of *Pleurotus eryngii* control and *Erwinia beijingensis*-infected fruiting bodies. (A) Control (no infection), scale bar = 1 μm; (B) 2 dpi, scale bar = 2 μm; (C) 5 dpi, scale bar = 2 μm; and (D) 9 dpi, scale bar = 2 μm. CW: cell wall; N: nucleus; M: mitochondrion; B: bacteria.

The morphology of the infected king oyster mushrooms was analyzed by SEM (Fig. 2). Normal hyphae and spores were observed in the control tissue (Fig. 2A and E). At 2 dpi, the bacteria adhered to hyphae, and the hyphal morphology were still normal (Fig. 2B and F). At the midstage of infection (5 dpi), the infected tissue displayed hyphal deformations and breakages (Fig. 2C and G). At 9 dpi, large areas of hyphal damage were observed (Fig. 2D and H). TEM images further revealed that control, uninfected fruiting bodies contained intact cells with typical organelles and well-integrated cell walls (Fig. 3A). At 2 dpi, the cells continued to exhibit well-integrated cell walls while some of the mitochondria remained intact; however, some organelles appeared damaged (Fig. 3B). At 5 dpi, the cell walls remained well-integrated; however, more organelles appeared damaged, and some bacteria had invaded the cell (Fig. 3C). At 9 dpi, the cell wall structure appeared deformed, and the border of the cell wall became blurred and irregular, suggesting loss of cell wall integrity. No intact organelles were observed in these cells (Fig. 3D).

### Analysis of RNA-seq data reveals DEGs.

RNA-seq was performed on RNA extracted from fruiting bodies collected at 2, 5, and 9 dpi (labeled R1, R2, and R3, respectively) and three uninfected control groups collected at the same time points (labeled Z1, Z2, and Z3, respectively), to compare changes in DEGs over time during infection with *E. beijingensis*. The raw data are deposited in the National Genomics Data Center, China National Center for Bioinformatics, under accession number CRA003723. Q20 values of the sequencing data ranged from 97.73% to 99.08%, Q30 values ranged from 93.02% to 97.08%, and the mapping rates were 82.60% to 89.50% (Table S2).

The q value and log2FC were used to screen for DEGs using a q value < 0.05 and |log2FC|≥1 as cutoff values. Compared with the control group, a total of 4, 296 genes were identified as DEGs. More specifically, when comparing R1 versus Z1, 2039 DEGs were identified (1561 downregulated and 478 upregulated); for R2 versus Z2, 1737 DEGs were identified (1169 downregulated and 568 upregulated); and for R3 versus Z3, 3167 DEGs were identified (1828 downregulated and 1339 upregulated) (Tables S3-S5). In general, a higher number of downregulated *versus* upregulated genes was detected in the infected fruiting bodies.

**FIG 4:**
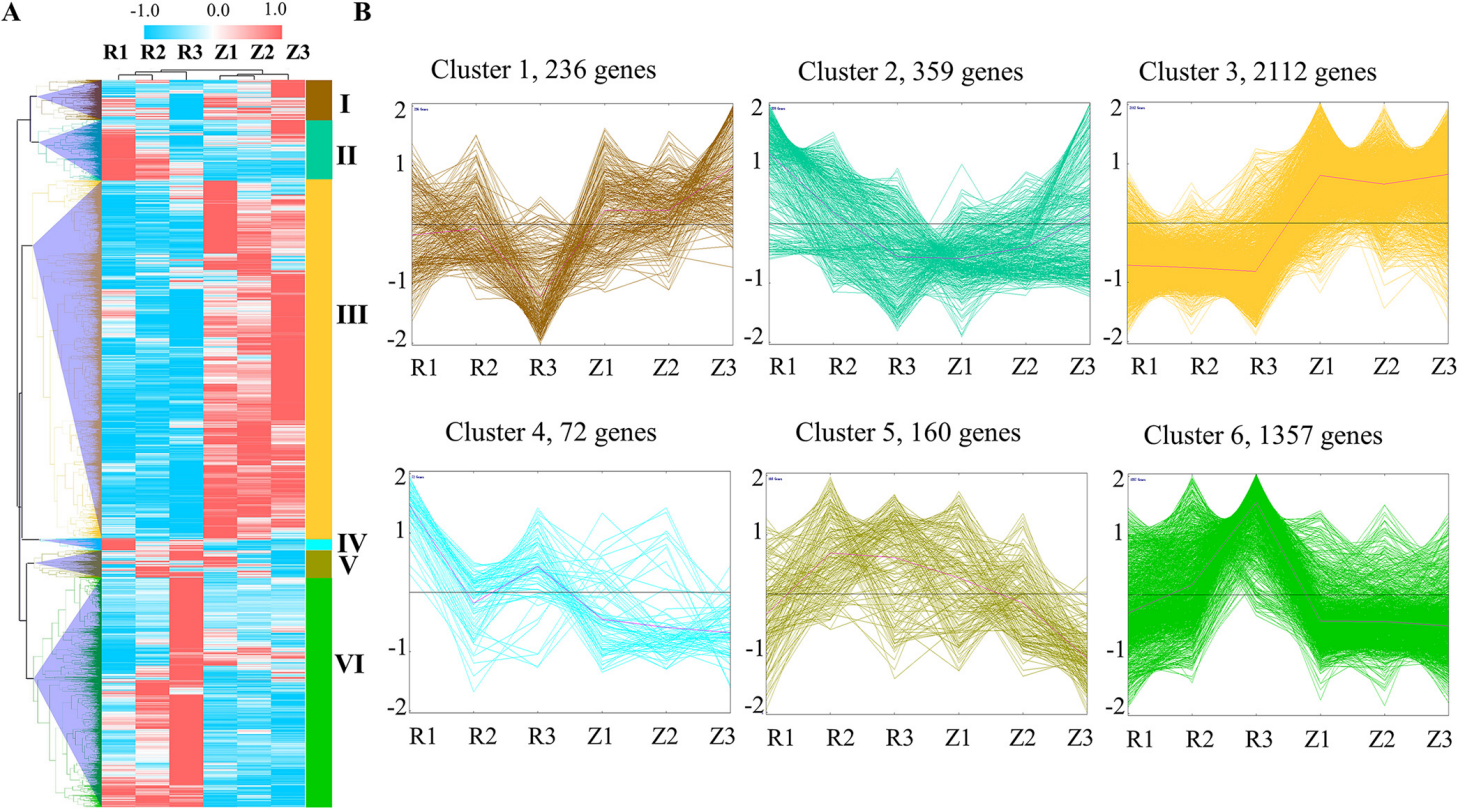
Expression patterns of all differentially expressed genes (DEGs) of *Pleurotus eryngii* over time of infection. (A) Heat map of all DEGs at 2, 5, and 9 dpi; (R1, R2, and R3, respectively) compared with their respective uninfected control (Z1, Z2, and Z3). (B) Expression trends of genes in six clusters according to the infection time course. The brown, green, blue, and yellow lines in each subgraph indicate the relative expression of DEGs in a cluster in the different treatment groups, and the pink lines indicate the average relative expression of all genes in a cluster in the different treatment groups. The *x* axis indicates the samples, and the *y* axis shows the relative gene expression.

To better understand the transcriptome changes in *P. eryngii* in response to *E. beijingensis* infection, the expression patterns of all DEGs at the three stages of infection were analyzed and found to cluster (Fig. 4A). Almost half (49.16%) of the DEGs belonged to cluster 3, genes that were downregulated after infection (Fig. 4B).

### Enriched GO terms differ among the three infection stages.

**FIG 5:**
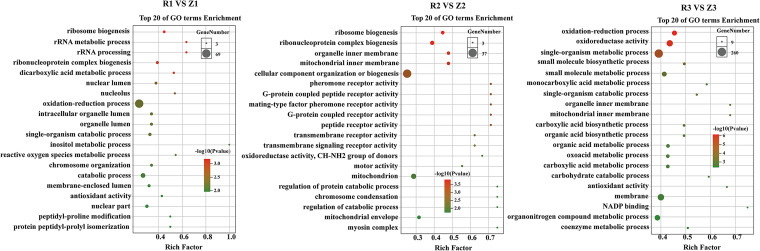
Gene Ontology (GO) enrichment analyses of differentially expressed genes (DEGs) between different treatment group comparisons. R1, R2, and R3: fruiting bodies collected at 2, 5, and 9 dpi, respectively; Z1, Z2, and Z3: three uninfected control groups collected at the same time points.

GO enrichment analyses were performed for all significant DEGs between treatment and control groups. Different group comparisons showed similar distribution patterns with regard to the number and type of enriched pathways, which can be divided into three main functional groups, including 16 biological processes (BP), 12 molecular functions (MF), and 10 cellular components (CC; Fig. S1). DEGs were enriched in basic metabolism processes, including ribosome biogenesis, rRNA metabolic process, and rRNA processing at 2 dpi (R1 versus Z1). Meanwhile, genes involved in ribosome biogenesis, ribonucleoprotein complex biogenesis, and organelle inner membrane were enriched at 5 dpi (R2 versus Z2), and oxidation-reduction processes, oxidoreductase activity, and single-organism metabolic process were enriched at 9 dpi (R3 versus Z3; Fig. 5).

**FIG 6:**
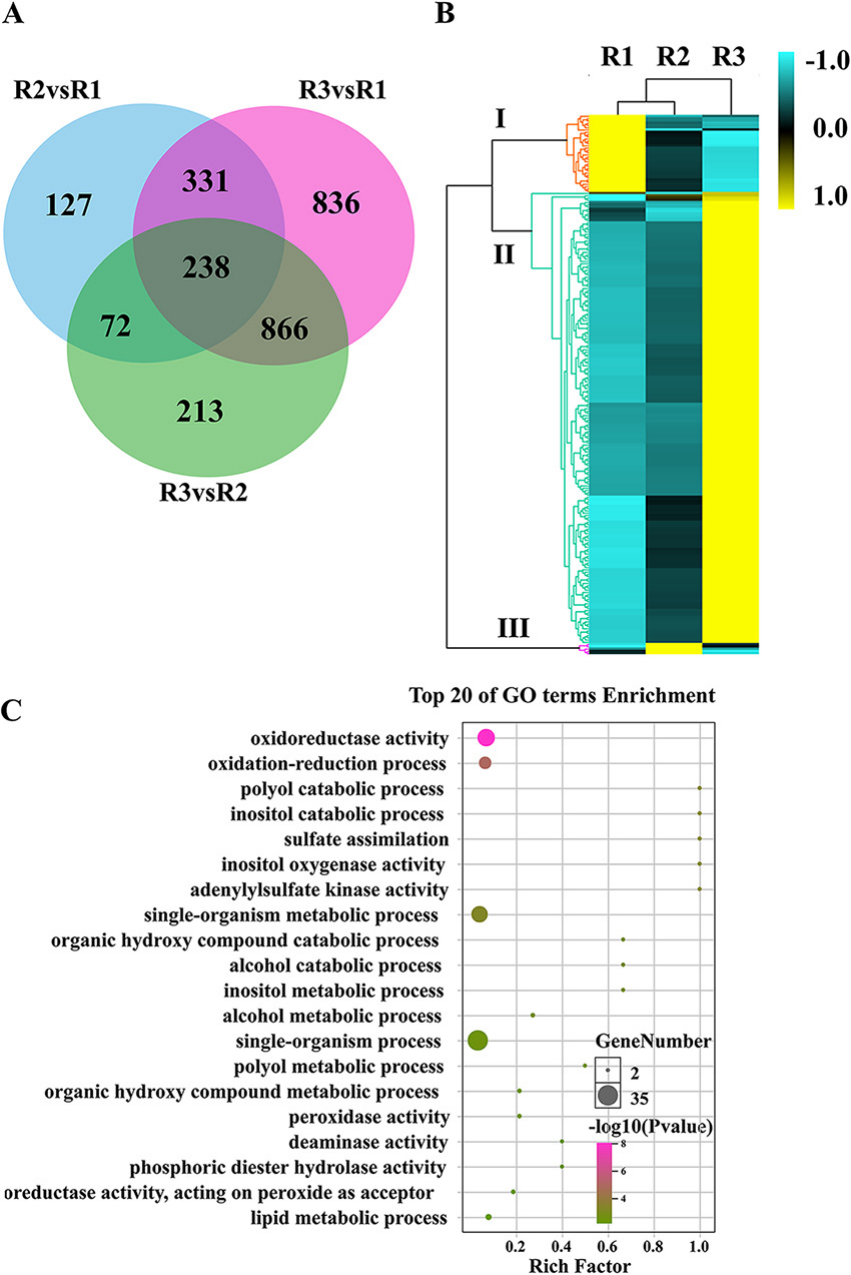
Overlapping differentially expressed genes (DEGs) at different infection time points (R2 versus R1, R3 versus R1, and R3 versus R2). (A) Venn diagram displaying the distribution and overlap of DEGs at different infection time points. (B) Heat map showing changes in expression among the common DEGs. (C) Scatterplots of GO enrichment analysis. R1, R2, and R3: fruiting bodies collected at 2, 5, and 9 dpi, respectively.

When comparing treatment groups R2 versus R1, 768 DEGs were identified; for R3 versus R1, 2271 DEGs; and for R3 versus R2, 1389 DEGs. A total of 238 DEGs overlapped among the three treatment groups (R2 versus R1, R3 versus R1, and R3 versus R2) (Fig. 6A). The 238 DEGs could be divided into three clusters based on expression patterns. In clusters I and III, gene expression was upregulated at 2 dpi and 5 dpi, respectively; 83.61% of common genes belonged to cluster II, in which genes were upregulated at 9 dpi (Fig. 6B). In addition, oxidoreductase activity and oxidation-reduction processes were enriched among the 238 common genes (Fig. 6C).

### The peroxisome pathway is commonly enriched among the three infection stages.

**FIG 7:**
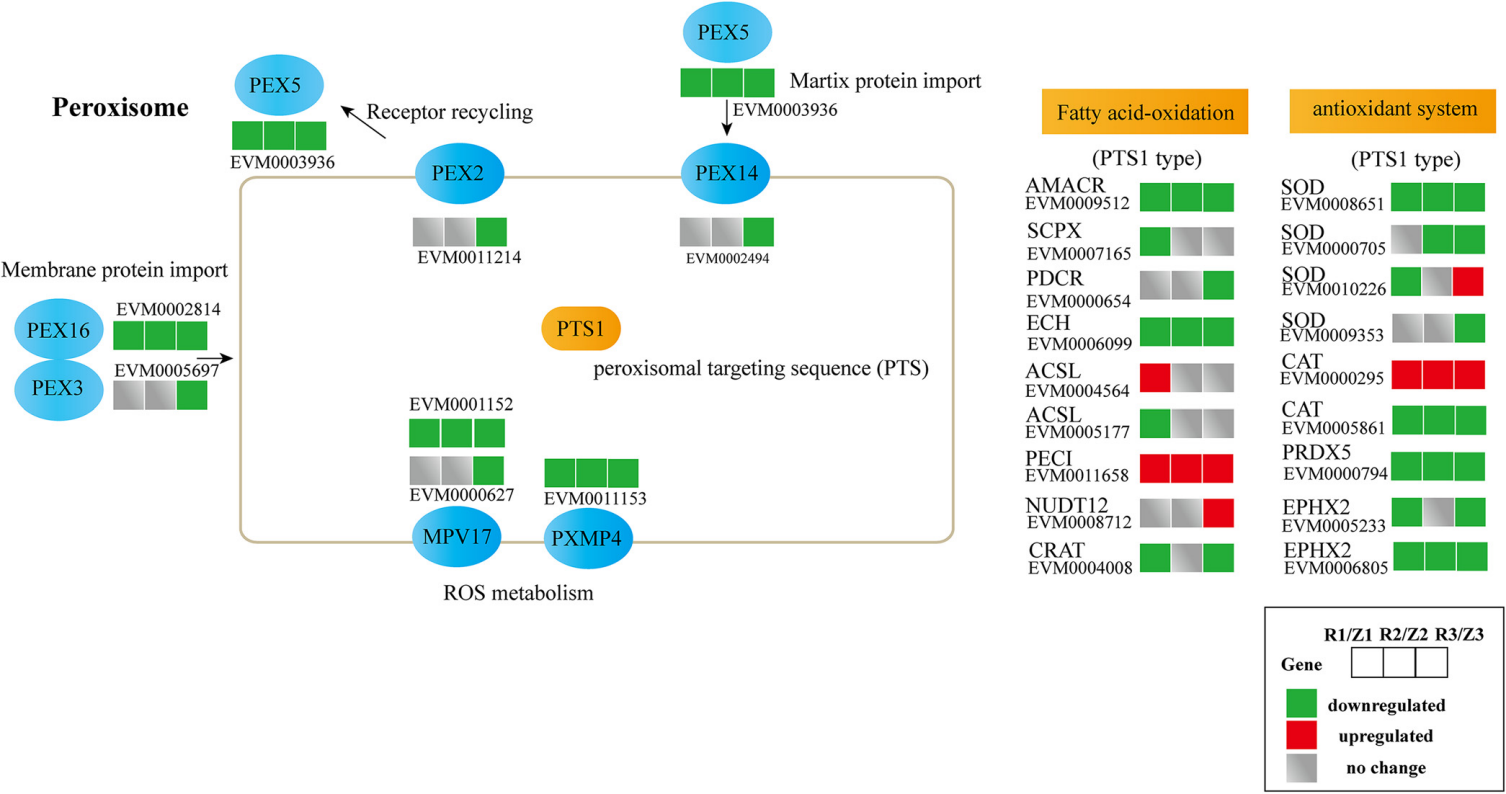
DEGs involved in the peroxisome pathway in *Pleurotus eryngii* infected with *Erwinia beijingensis*. R1, R2, and R3: fruiting bodies collected at 2, 5, and 9 dpi, respectively; Z1, Z2, and Z3: three uninfected control groups collected at the same time points.

To further identify the biological pathways associated with the DEGs, KEGG enrichment was performed (Fig. 7 and Fig. S2). In the R1 versus Z1 comparison, the DEGs were enriched in peroxisome, fatty acid biosynthesis, purine metabolism, and longevity regulating pathways. In the R2 versus Z2 analysis, the DEGs were related to selenocompound metabolism, glutathione metabolism, nitrogen metabolism protein export, taurine, and hypotaurine metabolism, as well as methane metabolism. Moreover, the top 20 enriched pathways included the peroxisome pathway in R2 versus Z2. Nineteen pathways were significantly enriched in the R3 versus Z3 analysis, including biosynthesis of amino acids, biosynthesis of antibiotics, and peroxisome (Fig. S2). Meanwhile, the peroxisome pathway was the common enriched pathway among the three time points. Peroxisomes play important roles in cell metabolism and possess intricate protective mechanisms to counteract oxidative stress and maintain redox balance (17, 18). Additionally, four superoxide dismutase (SOD) and two catalase (CAT) genes, which are related to ROS scavenging, were found in the peroxisome pathway, and three SOD genes and one catalase gene were downregulated in infected tissues (Fig. 7).

### Most DEGs common across the three infection stages are downregulated after infection.

**FIG 8:**
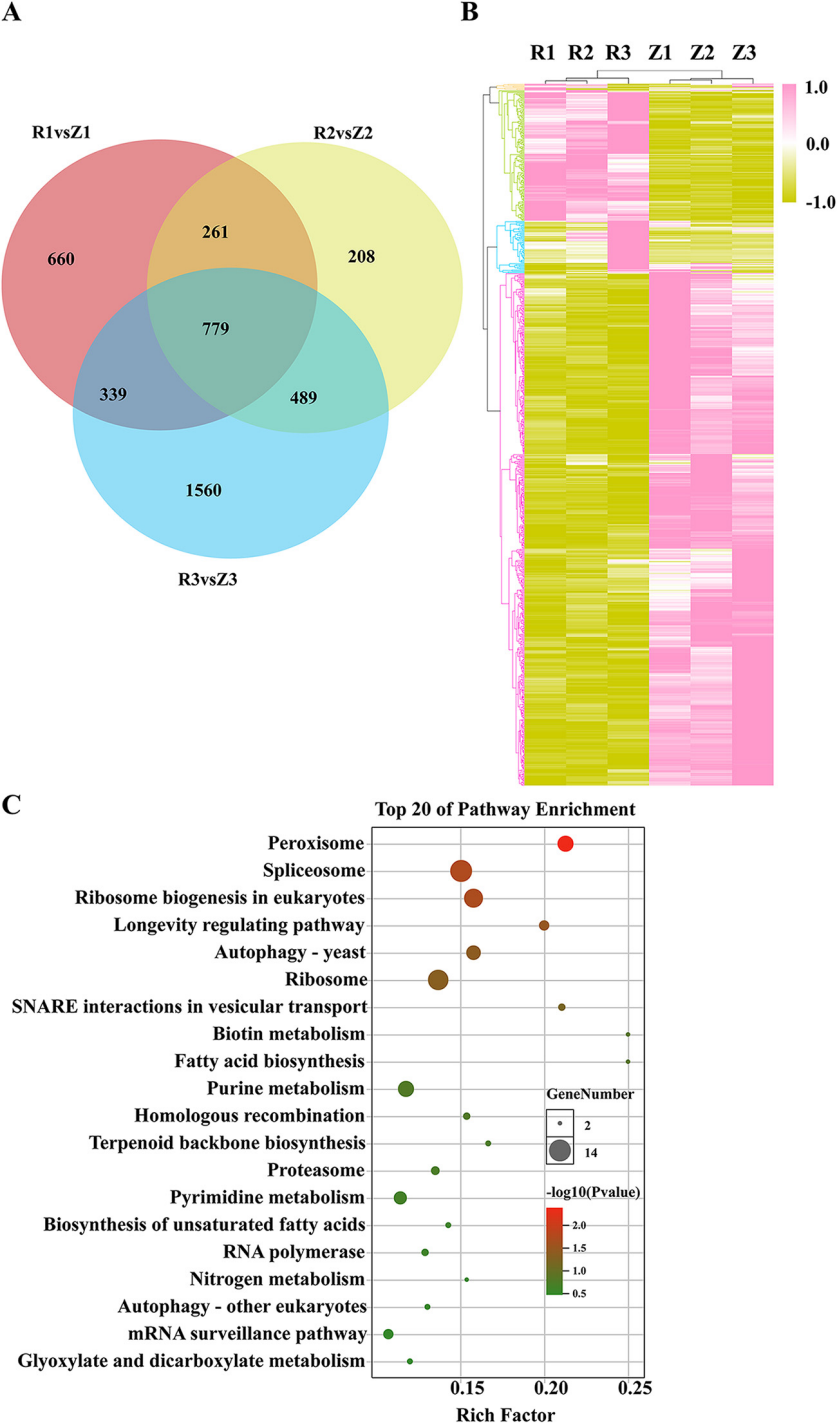
Common differentially expressed genes (DEGs) among the different time treatment groups. (A) Venn diagram displaying the distribution and overlap of DEGs at the three different infection time points. (B) Heat map showing the expression changes in common DEGs. (C) Scatterplot of KEGG enrichment analysis of common DEGs. R1, R2, and R3: fruiting bodies collected at 2, 5, and 9 dpi, respectively; Z1, Z2, and Z3: three uninfected control groups collected at the same time points.

A total of 779 DEGs overlapped among the three treatment groups (Fig. 8A, Table S6). The expression patterns of these common DEGs included two main clusters, which could be divided into four subclusters; 73.04% belonged to subcluster 4, genes were downregulated after infection (Fig. 8B). KEGG pathway annotation further showed that these genes are involved in different cellular and metabolic processes, including transport and catabolism, cell growth and death, as well as lipid and amino acid metabolism. The six most enriched pathways were peroxisome, ribosome biogenesis, spliceosome, longevity regulating pathway, ribosome, and autophagy (Fig. 8C).

### Cell wall-degradation genes are differentially expressed in the infection groups.

The fungal cell wall is a dynamic structure, composed typically of chitin, β-glucan, and proteins (19), that protects the cell from changes in response to environmental stresses (20). At 9 dpi, the structure of the *P. eryngii* cell wall exhibited damage and loss of integrity. Genes related to fungal cell wall degradation were differentially expressed in the infection groups (Table 1), including two encoding cellulase (EVM0004568 and EVM0008131) that were upregulated following *E. beijingensis* infection at all three time points. Three additional cellulase genes (EVM0002286, EVM0004678, and EVM0010790) were upregulated at 9 dpi. Moreover, three chitinase encoding genes (EVM0011078, EVM0006112, and EVM0006155) were significantly upregulated after *E. beijingensis* infection at 9 dpi. Similarly, the EVM0001416 gene that belongs to the thaumatin family was upregulated after *E. beijingensis* infection at 9 dpi; members of this family possess endo-β-1,3-glucanase activity and can degrade the cell wall components of shiitake mushrooms (Lentinula edodes) (21). Increased expression (up to 6.9-fold) of five genes (EVM0002115, EVM0008710, EVM0010621, EVM0006789, and EVM0007642) encoding proteases were found at all three time points; three of these genes are metalloproteases, one is a serine proteinase, and one is an aspartic protease. Eight genes encoding proteases were significantly upregulated during pathogen attack at 9 dpi. The aspartic protease gene EVM0007544 was upregulated during infection at 5 and 9 dpi.

**TABLE 1:** Genes involved in cell wall degradation

Gene ID	Annotation	log2 fold change (R1Z1)	q value	log2 fold change (R2Z2)	q value	log2 fold change (R3Z3)	q value
EVM0011078	Chitinase	0.47	4.61E-01	0.80	1.38E-01	1.78^*a*^	4.28E-04
EVM0006112	Chitinase	−0.47	3.18E-01	−0.63	1.48E-01	1.58^*a*^	4.28E-04
EVM0006155	Chitinase	−0.51	2.21E-01	0.07	9.03E-01	1.04^*a*^	1.45E-02
EVM0002286	Cellulase	0.70	2.98E-01	0.77	1.65E-01	1.33^*a*^	3.26E-02
EVM0010790	Cellulase	0.53	1.60E-01	0.79	2.86E-02	2.40^*a*^	4.28E-04
EVM0004568	Cellulase	1.06^*a*^	4.73E-03	0.96	1.06E-02	1.45^*a*^	4.28E-04
EVM0008131	Cellulase	1.17^*a*^	4.28E-04	0.70	4.42E-02	2.01^*a*^	4.28E-04
EVM0004678	Cellulase	−0.55	2.29E-01	0.21	6.88E-01	1.43^*a*^	4.28E-04
EVM0001416	Thaumatin family	0.13	8.62E-01	0.37	5.71E-01	1.23^*a*^	2.42E-02
EVM0000675	Aspartyl protease	−1.37^*a*^	4.28E-04	−0.30	5.17E-01	1.81^*a*^	4.28E-04
EVM0001273	Aspartyl protease	−0.48	2.53E-01	0.77	2.96E-02	1.71^*a*^	4.28E-04
EVM0002115	Metalloprotease	3.19^*a*^	4.28E-04	1.66^*a*^	4.28E-04	1.97^*a*^	4.28E-04
EVM0008710	Serine protease	4.95^*a*^	4.28E-04	5.11^*a*^	4.28E-04	3.35^*a*^	4.28E-04
EVM0006129	Cuticle-degrading protease	0.97	6.85E-01	2.27^*a*^	2.88E-01	6.38^*a*^	4.49E-02
EVM0010175	Aspartyl protease	0.80	1.70E-02	0.50	1.71E-01	1.46^*a*^	4.28E-04
EVM0010621	Metalloprotease	4.74^*a*^	4.28E-04	3.99^*a*^	4.28E-04	6.92^*a*^	4.28E-04
EVM0009659	Ubiquitin-specific protease	0.64	9.35E-02	0.73	4.40E-02	1.44^*a*^	4.28E-04
EVM0001510	Aspartyl protease	0.32	4.53E-01	−1.62^*a*^	4.28E-04	3.14^*a*^	4.28E-04
EVM0006789	Aspartyl protease	1.28^*a*^	4.28E-04	1.26^*a*^	4.28E-04	1.45^*a*^	4.28E-04
EVM0007642	Metalloprotease	4.50^*a*^	4.28E-04	3.94^*a*^	4.28E-04	2.68^*a*^	4.28E-04
EVM0005816	Aspartyl protease	0.66	8.96E-02	0.29	5.28E-01	1.68^*a*^	4.28E-04
EVM0007544	Aspartyl protease	0.83	1.53E-01	1.82^*a*^	4.28E-04	4.68^*a*^	4.28E-04
EVM0011893	Subtilisin-like protease	0.57	2.93E-01	0.74	1.40E-−01	1.40^*a*^	2.70E-02

a Significant differentially expressed genes, |log2FC| ≥1, q value < 0.05.

### ROS and antioxidant-related genes are modulated after infection.

**FIG 9:**
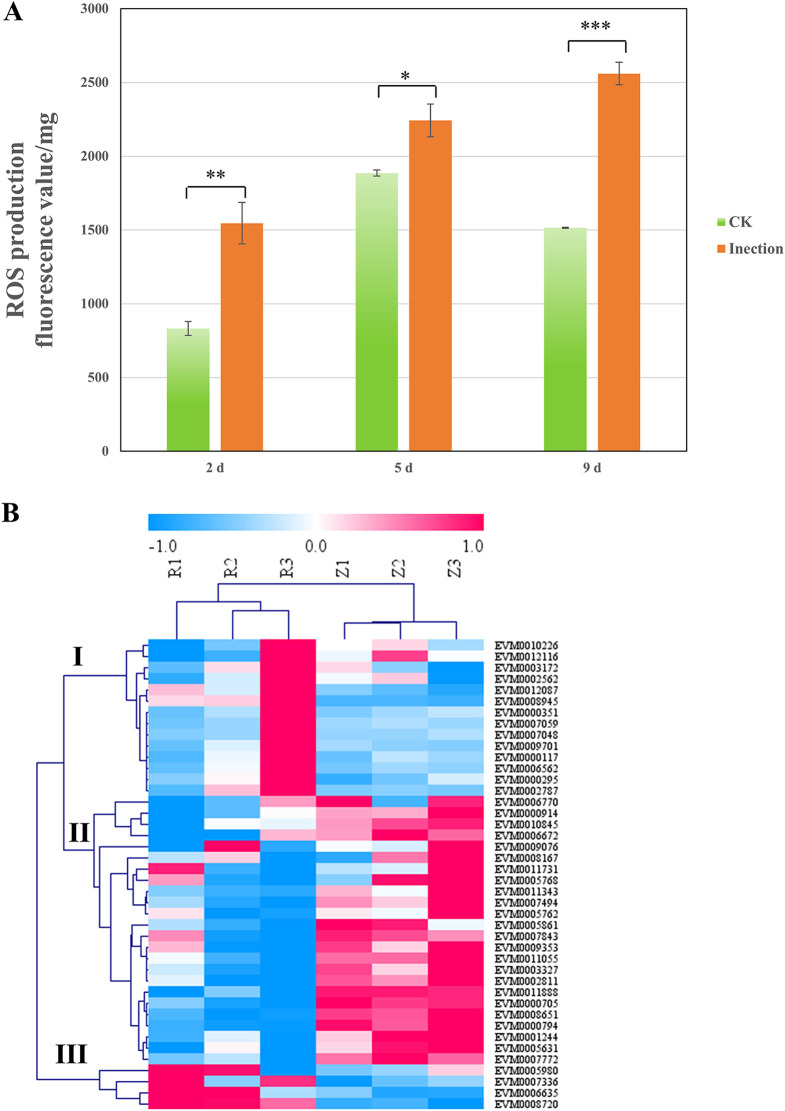
Reactive oxygen species (ROS) production and antioxidant-related genes analysis. (A) ROS levels of different treatment groups (infected) compared to the uninfected control (*n* = 3; *, *P < *0.05; **, *P < *0.01; ***, *P < *0.001). (B) Heat map showing changes in expression of antioxidant-related genes. R1, R2, and R3: fruiting bodies collected at 2, 5, and 9 dpi, respectively; Z1, Z2, and Z3: three uninfected control groups collected at the same time points.

GO and KEGG analyses revealed that certain redox-related terms were enriched among the treatments. Oxidative stress is a process that relies on an imbalance between the production and degradation of ROS (22). A significant increase in ROS levels was observed in the fruiting bodies after infection by *E. beijingensis* at each of the time points compared to that of the corresponding control (Fig. 9A). The ROS concentration also increased in a time-dependent manner within the infected fruiting bodies.

Moreover, SOD, CAT, the glutathione system, thioredoxin system, and peroxidase system represent the prominent enzymatic antioxidants used to scavenge excess internal and external ROS (22). Fifty-six genes related to antioxidant activity were identified in *P. eryngii*, including 4 SOD, 23 peroxidase, 2 CAT, 1 glutathione peroxidase, 1 glutathione reductase, 5 glutaredoxins, 4 peroxiredoxin, 15 thioredoxin, and 1 thioredoxin reductase. Among these genes, 42 were DEGs in at least one time point (Fig. 9B). When the expression pattern of the 42 DEGs was examined across all three infection time points, three main clusters emerged (Fig. 9B). Cluster II, including genes downregulated after infection, dominated (57.14%); among this cluster are genes encoding 3 SOD, 1 CAT, 12 peroxidase, 2 glutaredoxin, 1 peroxiredoxin, and 5 thioredoxin.

### Expression of *atg* genes are induced in soft rot tissue.

TEM observation showed that as the infection time progressed, organelles gradually degraded in the cells of the fruiting bodies. Autophagy is a ubiquitous, nonselective mechanism of continuous degradation of proteins and organelles (23), and protein kinase Tor1 is a signaling protein required for autophagy induction (18). The gene EVM0010923, encoding Tor1, was upregulated in the fruiting bodies at 5 and 9 dpi (Table 2). Additionally, the autophagy-related gene *atg8* EVM0008410 was upregulated at 9 dpi, whereas *atg2* was upregulated at all three time points and *atg7* was upregulated at 5 and 9 dpi. Phosphatidylinositol 3-kinase Vps34 complexes regulate intracellular membrane trafficking in endocytic sorting, cytokinesis, and autophagy (24). EVM0002585 *vps34* was upregulated at all three infection time points. EVM0008710 encoding PRB1, a proteinase that can degrade the inner vesicle, was also upregulated at all three time points. These events are critically important for the completion of autophagy in autophagic processes.

**TABLE 2:** Genes involved in autophagy

Gene ID	Annotation	log2 fold change (R1Z1)	q value	log2 fold change (R2Z2)	q value	log2 fold change (R3Z3)	q value
EVM0010923	*tor1*	0.66	6.51E−02	0.84	1.53E−02	1.31^*a*^	4.28E−04
EVM0008410	*atg8*	0.36	3.91E−01	0.41	3.15E−01	2.13^*a*^	4.28E−04
EVM0008733	*atg7*	0.33	4.10E−01	0.74	2.65E−02	1.91^*a*^	4.28E−04
EVM0002999	*atg2*	1.13^*a*^	7.96E−04	1.17^*a*^	7.96E−04	1.57^*a*^	4.28E−04
EVM0002585	*vps34*	0.74	3.15E−02	1.07^*a*^	1.47E−03	1.29^*a*^	4.28E−04
EVM0008710	*PRB1*	4.95^*a*^	4.28E−04	5.11^*a*^	4.28E−04	3.35^*a*^	4.28E−04

a Significant differentially expressed genes, |log2FC| ≥1, q value <0.05.

### RNA-seq data are validated by RT-qPCR.

Five candidate DEGs were selected for validation experiments by RT-qPCR, including EVM0006723, EVM0004026, EVM0001604, EVM0002562, and EVM0004221. The RT-qPCR results were in accordance with the transcriptomic data, supporting the accuracy of the results (Fig. S3).

## DISCUSSION

To date, the response of *P. eryngii* to *E*. *beijingensis* infection remains poorly understood; hence, this study aimed to characterize the ultrastructural changes, as well as specific DEGs associated with early, middle, and late stages of *E*. *beijingensis* infection. As such, this is the first report to comprehensively describe the response of *P*. *eryngii* to pathogenic infection by *E. beijingensis*.

Similar to what has been described for plant species facing various biotic or abiotic stresses, including pathogen attack (25, 26), we observed enhanced synthesis of ROS in the fruiting bodies of *P. eryngii* following *E. beijingensis* infection. Accordingly, we postulate that induction of a ROS burst represents the initial defense strategy of *P. eryngii* when facing pathogenic attack. Under normal conditions, ROS are counterbalanced by antioxidant systems to maintain the integrity of the organism (22). Specifically, antioxidative enzymes and or metabolites, such as SODs, CATs, glutathione peroxidases, glutathione reductases, thiol peroxidases, thioredoxins, glutaredoxins, and peroxiredoxins, participate in ROS detoxification (22). Meanwhile, following infection of *P. eryngii*, 57.14% of the genes encoding antioxidative enzymes were downregulated, thereby inhibiting the ROS scavenging capacity of cells and consequently increasing the ROS levels in infected fruiting body tissue. ROS then react readily with lipids, DNA, proteins, and other macromolecules; however, excess ROS can lead to cell injury, and eventually, cell death or strain degeneration (25, 27, 28). Specifically, ROS mediate programmed cell death pathways, including autophagy-related programmed cell death (29). Within the current study, we observed upregulation of certain essential autophagy-related (*atg*) genes during *P*. *eryngii* infection, including *atg8* and *atg2*, which encode ubiquitin-like and lipid transfer protein, respectively (30, 31). These results agree with those of a previous study, which reported upregulation of *atg* genes in soft rot tobacco plants (32). Additionally, phosphatidylinositol 3-kinase (Vps34), which is present in all eukaryotes, plays an important role in all vesicle sorting pathways to lysosomes, including phagocytosis and autophagy (24). In this study, EVM0002585, encoding Vps34, was significantly upregulated during infection, as was the gene encoding Tor1. Thus, we postulate that the expression of Tor1, a signaling protein required for autophagy induction (18), was mediated by the cellular response of *P*. *eryngii* to the ROS burst, and subsequently functioned to initiate autophagy.

Additionally, KEGG analysis revealed that genes related to peroxisomal pathways were enriched in the comparison of infected with control fruiting bodies. Peroxisomes are conserved organelles of eukaryotic cells that play an important role in cellular metabolism, redox homeostasis, and intracellular metabolite transfer and signaling (33). Perhaps most importantly, peroxisomes possess intricate protective mechanisms to counteract oxidative stress, including the generation of reactive oxygen and nitrogen species in the organelle itself to maintain redox balance (17). Meanwhile, we observed downregulation of seven genes involved in peroxisome biogenesis (PEX genes) (18) during *E. beijingensis* infection (Fig. 7). Moreover, 67.7% of the genes involved in fatty acid oxidation of peroxisomes and 77.8% of the genes involved in the antioxidant system were downregulated during infection. These results clearly suggest that peroxisome homeostasis, which is achieved by the regulated interplay between peroxisome biogenesis and degradation via selective autophagy (29), was disrupted in infected *P*. *eryngii*.

Genes associated with oxidoreductase activity and oxidation-reduction processes (Table S7), including those encoding cytochrome P450, CATs, SODs, dehydrogenases, oxidases, and reductases, were enhanced in *P*. *eryngii* fruiting bodies after infection. Among these genes, that encoding cytochrome P450 was the most predominantly induced after *E*. *beijingensis* infection (19.11%). Cytochrome P450s, which are heme-containing proteins, represent one of the largest protein families and are involved in the biosynthesis of structural components, signaling networks, secondary metabolism, and xenobiotic/drug detoxification (34). As such, genes encoding cytochrome P450 have been reported to function in the defense response of *F. velutipes* to pathogenic bacteria (9). In line with these results, we observed upregulation of 24 cytochrome P450 genes at 9 dpi, highlighting the importance of P450 in the defense response of *P*. *eryngii* to soft rot disease pathogens. At different infection time points (R2 versus R1, R3 versus R1, and R3 versus R2), 238 common DEGs were observed. The two most enriched GO terms were oxidoreductase activity and oxidation-reduction processes, including nine cytochrome P450 genes, which were upregulated at the third infection stage. Furthermore, considering that enriched oxidation-reduction processes have also been reported in blight fruiting bodies of winter mushrooms (9), redox homeostasis is clearly an integral component of the defense mechanism employed by various mushroom species to attack invading pathogens.

The fungal cell wall is a dynamic structure composed of glycoproteins and polysaccharides, primarily β-glucan and chitin, that protects the cell from environmental stress and provides cellular integrity (20, 35). Disruption of the cell wall structure has a profound effect on the growth and morphology of fungal cells, often rendering them susceptible to lysis and death (20). When *P. ostreatus* was subjected to postharvest stress, expressed sequence tags (EST) for carbohydrate active enzymes (CAZy) were predominant, including chitin metabolism related to cell wall structure (36). When *P. ostreatus* attacked the plant cell, it secreted a variety of glycoside hydrolases, redox enzymes, carbohydrate esterases, polysaccharide lyases, and proteases (37, 38). In the current study, the cell wall of infected cells was deformed with diminished integrity during late-stage infection. Analysis of the genes related to cell wall degradation in infected *P*. *eryngii* at 9 dpi revealed 23 differentially upregulated genes, including 3 chitinase genes, 1 β-1,3-glucanase gene, 5 cellulase genes (glycoside hydrolase family 5), and 14 protease genes. Similarly, three genes encoding β-1,3-glucanase were previously reported to be upregulated in bacteria-infected *F. velutipes* (9). Chitinases, β-1,3-glucanase, and proteases represent the main cell wall degrading enzymes in fungi and thus, cell wall hydrolysis catalyzed by these enzymes plays a major role in cell wall softening in fungi (20, 39, 40).

Taken together, these results indicate that the transcriptional changes in genes associated with cell wall degradation likely caused the collapse of the *P*. *eryngii* cell wall, effectively destroying the first line of defense against *E*. *beijingensis* infection.

Based on the cumulative results of this study, we propose a potential disease mechanism model for *P. eryngii* infected by soft rot bacteria such as *E. beijingensis.* First, ROS are rapidly released following the downregulation of antioxidative genes. Second, high levels of ROS induce autophagy-related gene expression changes leading to damage of the cellular organelles, including peroxisomes. Consequently, redox homeostasis is disrupted, and an oxidative burst accompanies cell death and cell wall damage in infected king oyster mushrooms.

## MATERIALS AND METHODS

### Pathogen infection manipulation.

*Erwinia beijingensis* LMG 27579^T^ was cultured in Trypticase soy broth (TSB; Oxoid company, Wade Road, Basingstoke, UK) and shaken at 28°C until logarithmic phase, followed by dilution of the bacterial suspension to 10^8^ CFU (CFU)/mL. *Pleurotus eryngii* strain JZB2107045 was cultured at 15°C in 90% relative humidity and grown on raw substrates, including 37% cottonseed hull, 30% corncob, 10% sawdust, 18% wheat bran, 3% corn flour, and 2% lime. Young fruiting bodies with a 2 cm stipe were inoculated in bottles by spraying their surface with 1 mL of the *E. beijingensis* bacterial suspension (10^8^ CFU/mL). Infected fruiting bodies were collected at 2, 5, and 9 days postinoculation (dpi; *n* = 3 bags containing 3 to 6 mushrooms per bag per time point). According to the disease process, 2, 5, and 9 dpi correspond with early, middle, and late stages of infection, respectively. Meanwhile, a control group was similarly treated with 1 mL of sterile water, and samples were collected at the same time points as for the infected samples. The surface of the infected or control stipe was cut from each fruiting body, and samples were immediately frozen in liquid nitrogen and stored at −80°C until RNA was extracted. Samples from each of the three different bags were defined as three biological replicates for each group, which were collected at each time point.

### Cell ultrastructure observation.

For scanning electron microscopy (SEM), tissue fragments were cut from fruiting body and fixed in 2% glutaraldehyde in phosphate buffer (pH 7.2) for 2 h at 4°C. After being rinsed with the buffer, tissues were postfixed overnight in 2% osmium tetroxide in the buffer at 4°C. The fixed material was dehydrated in an ethanol series, transferred to isoamyl acetate and critical-point dried in a dryer using carbon dioxide. Samples were observed using a transmission electron microscope (JEOL JEM-1OOCX IL; JEOL, Tokyo, Japan) at 80 kV. The morphological features of the samples were observed with scanning electron microscopy (SEM; S-4800, Hitachi, Tokyo, Japan) at an accelerating voltage of 20 kV, in secondary electron imaging mode. Magnification 5000×, and the effective working distance was 12.3 mm. The ultrastructural characteristics of fruiting bodies were observed using transmission electron microscopy (TEM). The control and infected samples were fixed with 2.5% glutaraldehyde in 0.1 M phosphate buffer (pH 7.2) for 24 h at 4°C. The fixed samples were dehydrated using an ethanol gradient and embedded in Epon 812 resin (Electron Microscopy Sciences, Hatfield, PA, USA). Thin sections (70 nm) were cut with a diamond knife (EM UC7; Leica, Wetzlar, Germany) and stained with 2% uranyl acetate for 10 min, followed by 3% lead citrate for 3 min. The samples were observed using a transmission electron microscope (JEOL JEM-1OOCX IL; JEOL, Tokyo, Japan) at 80 kV.

### RNA sequencing and data processing.

Total RNA was extracted using TRIzol according to the manufacturer’s instructions (Invitrogen, Carlsbad CA, USA). mRNA purification, cDNA library construction, and adapter ligation were performed following the methodologies for Illumina RNA-seq libraries by Shanghai Majorbio Bio-Pharm Technology (Shanghai, China). Sequencing was performed on an Illumina HiSeq 4000. The raw paired-end reads were trimmed and subjected to quality control using SeqPrep (https://github.com/jstjohn/SeqPrep) and sickle (https://github.com/najoshi/sickle) with default parameters. Clean reads were then separately aligned to the reference genome with orientation mode using TopHat v2.0.0 software (http://tophat.cbcb.umd.edu/) (41). The data sets generated in this study are deposited with the National Genomics Data Center, China National Center for Bioinformatics, under accession number CRA003723 (https://bigd.big.ac.cn/?_blank). The accession number of the reference genome is GWHBGBW00000000.

### DEG analysis.

To identify DEGs between two different samples, the expression level of each transcript was calculated according to the fragments per kilobase of exon per million mapped reads (FPKM) method. RSEM (http://deweylab.biostat.wisc.edu/rsem/) (42) was used to quantify gene abundance and Cufflinks software was used for differential expression analysis (43). Genes with a threshold fold change ≥ 1 and a q value < 0.05 were considered significant DEGs, which were then subjected to enrichment analysis using gene ontology (GO) (44, 45) functions and Kyoto Encyclopedia of Genes and Genomes (KEGG) pathways (46, 47). GO enrichment analysis provides all GO terms significantly enriched in DEGs compared to the whole transcriptome background. GO functional enrichment was carried out using Goatools (https://github.com/tanghaibao/Goatools) (48), and pathway enrichment analysis was performed using the KEGG database (49).

### ROS level detection.

ROS levels were measured as previously described, with minor modifications (50). The fruiting bodies of mushrooms were cut into small pieces comprising five gradient-weighed pieces per tissue. These tissues were then washed with phosphate-buffered saline (PBS), and suspended in 10 μM 2,7′-dichlorodihydrofluorescein diacetate (DCFH-DA) fluorescent dye for 30 min at room temperature for ROS detection. The fruiting bodies were washed three times with PBS (10 mM, pH 7.5) and fluorescence was measured using a microplate reader (infinite m200PRO; TECAN, Männedorf, Switzerland) at an excitation wavelength of 488 nm. The slope of the line after plotting fluorescence *versus* weight (mg) was used to calculate the ROS level/mg of fruiting body tissue.

### Real-time qPCR analysis.

To validate the transcriptome sequencing results, reverse transcriptase quantitative PCR (RT-qPCR) was performed. RNA (1 μg) was treated with DNase I (TaKaRa Bio Inc. Kusatsu, Shiga, Japan) to remove DNA before using the RevertAid First Strand cDNA Synthesis kit (Thermo Fisher Scientific, Waltham, MA, USA), according to the manufacturer’s instructions. Five genes were randomly selected for RT-qPCR analysis with glyceraldehyde-3-phosphate dehydrogenase (GAPDH) as the endogenous control to normalize the gene expression data. All primers are listed in Table S1. RT-qPCR was performed on an ABI 7500 real-time PCR system (Applied Biosystems, Carlsbad, CA, USA) using SYBR Premix Ex Taq II (TaKaRa Bio Inc.), according to the manufacturer’s instructions. Each RT-qPCR experiment was conducted in triplicate. Relative gene expression was determined using the 2^-△△CT^ method (51).
