# Supplementary material for: Bacterial Infection Induces Ultrastructural and Transcriptional Changes in the King Oyster Mushroom (Pleurotus eryngii)
Source: Microbiol Spectr. 2022 May 26;10(3):e01445-22. doi: 10.1128/spectrum.01445-22 (PMC9241817; doi:10.1128/spectrum.01445-22)
Supplement: SUPPLEMENTAL FILE 1 — Supplemental material. Download spectrum.01445-22-s001.pdf, PDF file, 4.9 MB [file spectrum.01445-22-s001.pdf]

Supplementary Material: Supplemental material is available online.

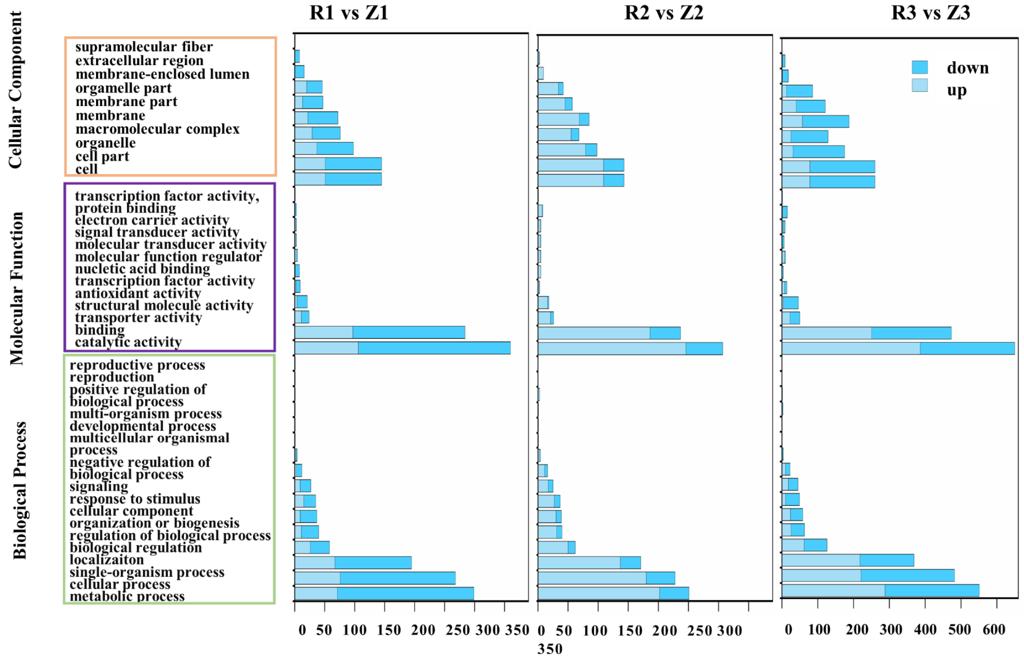

Supplementary Figure S1. Summary of the distribution and number of DEGs in three GO classes, including molecular function, cellular component, and biological process. R1, R2, and R3: fruiting bodies collected at 2, 5, and 9 dpi, respectively; Z1, Z2, and Z3: three uninfected control groups collected at the same time points.

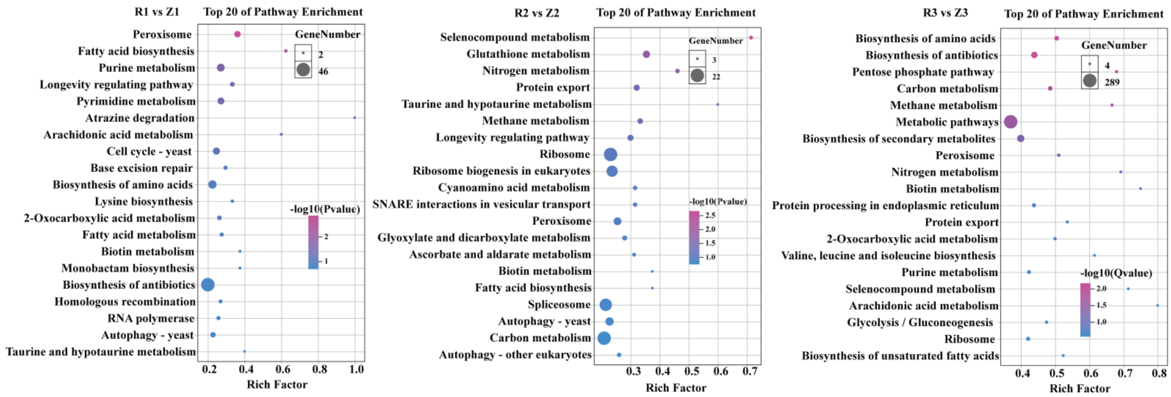

Supplementary Figure S2. Comparisons of KEGG enrichment analyses of differentially expressed genes (DEGs) between different treatment and control groups. R1, R2, and R3: fruiting bodies collected at 2, 5, and 9 dpi, respectively; Z1, Z2, and Z3: three uninfected control groups collected at the same time points.

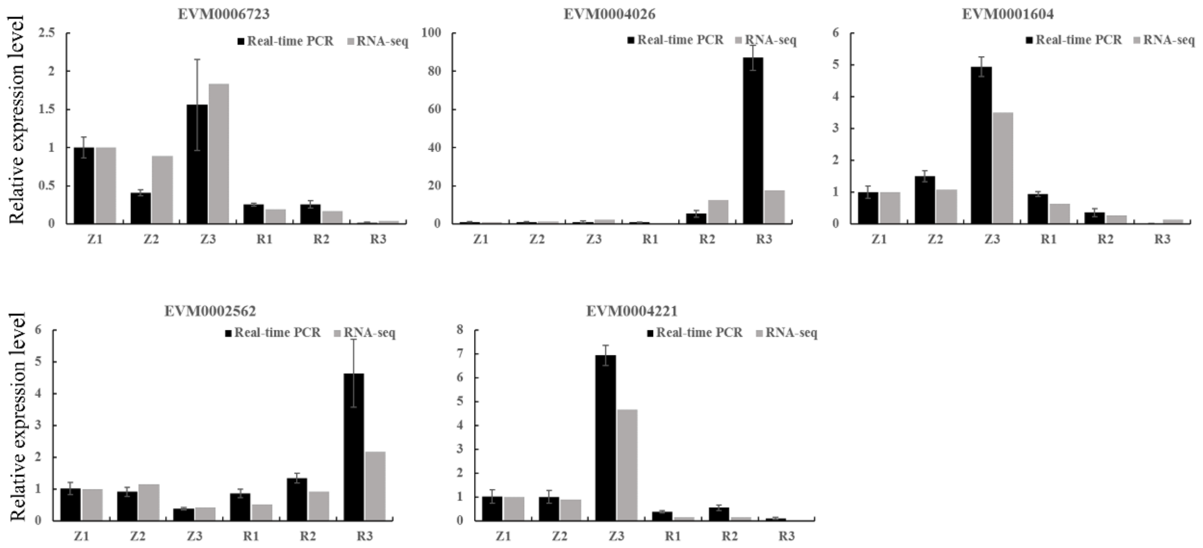

Supplementary Figure S3. RT-qPCR analysis of the five selected DEGs. The horizontal axis represents different groups, including R1, R2, R3, Z1, Z2, and Z3. The vertical axis represents relative expression levels.

Data Set S1. Information and primers used for RT-qPCR of selected differentially expression genes.

Data Set S2. Information of RNA-seq data for the 18 samples.

Data Set S3. Information of DEGs in R1 vs Z1.

Data Set S4. Information of DEGs in R2 vs Z2.

Data Set S5. Information of DEGs in R3 vs Z3.

Data Set S6. FPKM values for common DEGs of mushroom *P. eryngii* after *E. beijingensis* infection.

Data Set S7. Genes invovled in oxidoreductase activity and oxidation-reduction process.

Data Set S1 Information and primers used for RT-qPCR of selected differentially expression genes

Gene ID	Gene annotation	Forward primer(5’-3’)	Reverse primer(5’-3’)	Efficiency of PCR amplification
EVM0006723	Conidiation protein 6	CTCGTGATTTGCTCTACG	GTGTTCTTTGGTCCATCTTC	92.05%
EVM0004026	Secondary metabolites biosynthesis	ACCCTGCTTGGAACATCA	TATTGGCGACCGAACTCT	92.59%
EVM0001604	Sorbose reductase	TTCCAGGGAGCATCATTC	CAGTTCGCAAGCCATAGA	91.43%
EVM0002562	Mitochondrial peroxiredoxin PRX1	TCGTGGGCAGTCTTGTTCT	ATTCGTGGTGTTCCTGGAG	92.59%
EVM0004221	hypothetical protein	ACAGTGGTTCCAAGCCGT	CCTCGTCAAACAGTCCCA	101.74%
GAPDH*	Glyceraldehyde phosphatedehydrogenase	GTGATCTCAAATGCGTCATG	TGGGTAGCAGTGGTAGCG	93.46%

* represents the internal control gene

Data Set S2 Information of RNA-seq data for the 18 samples

Sample ID	Total_Reads	base pairs	Q20%	Q30%	GC%	mapping rate
Z1-A	50974830	7531536295	97.73	93.18	53.39	0.875
Z1-B	47929898	7082205205	97.79	93.32	53.08	0.894
Z1-C	48054594	7096997784	97.76	93.24	53.23	0.863
Z2-A	54860886	8119892714	97.88	93.52	53.16	0.858
Z2-B	52059346	7688398377	97.74	93.2	53.26	0.843
Z2-C	43531194	6429230721	97.66	93.02	53.27	0.826
Z3-A	59448596	8796840763	97.85	93.47	53.26	0.864
Z3-B	50798230	7516311797	97.84	93.44	53.2	0.881
Z3-C	54640940	8071514102	97.76	93.24	53.3	0.832
R1_A	98061730	14369696114	98.52	95.29	51.11	0.881
R1-B	75582742	10944449989	98.92	96.64	51.09	0.892
R1-C	79313902	11501811236	99.02	96.91	50.92	0.853
R2_A	109929638	16171054375	98.59	95.5	51.31	0.872
R2-B	80414886	11683166147	99.04	96.96	50.41	0.895

R2-C	94590562	13767515694	99.05	96.97	50.62	0.874
R3_A	95802450	14053987420	98.59	95.49	50.85	0.831
R3-B	87337482	12754774534	99.08	97.08	50.29	0.848
R3-C	85559530	12457528696	99.01	96.89	51.24	0.847

Data Set S3 Information of DEGs in R1 vs Z1

gene ID	R1 value	Z1 value	log2(fold change)	p value	q value	KOG class	Pfam annotation
EVM0009623	7.20	310.96	-5.43	5.00E-05	4.28E-04	--	--
EVM0001104	4.48	139.31	-4.96	5.00E-05	4.28E-04	--	--
EVM0007042	96.16	2483.20	-4.69	5.00E-05	4.28E-04	--	Hemerythrin HHE cation binding domain
EVM0010521	22.11	566.26	-4.68	5.00E-05	4.28E-04	--	--
EVM0008830	36.80	795.37	-4.43	5.00E-05	4.28E-04	Defense mechanisms	Dual specificity phosphatase, catalytic domain
EVM0000901	0.16	3.07	-4.26	1.10E-03	6.17E-03	Carbohydrate transport and metabolism	Starch binding domain
EVM0011483	43.53	629.06	-3.85	5.00E-05	4.28E-04	--	--
EVM0006076	21.85	315.32	-3.85	5.00E-05	4.28E-04	--	--
EVM0004031	11.09	158.24	-3.84	5.00E-05	4.28E-04	RNA processing and modification	Eukaryotic rRNA processing protein EBP2
EVM0004233	26.26	356.42	-3.76	5.00E-05	4.28E-04	--	--
EVM0006187	0.74	9.76	-3.71	5.00E-05	4.28E-04	Transcription	HMG (high mobility group) box
EVM0006622	4.48	57.86	-3.69	5.00E-05	4.28E-04	--	--
EVM0001788	26.14	336.53	-3.69	5.00E-05	4.28E-04	--	--

EVM0012049	3.07	39.20	-3.67	5.00E-05	4.28E-04	--	--
EVM0003167	14.40	181.82	-3.66	5.00E-05	4.28E-04	--	--
EVM0002148	67.67	851.70	-3.65	5.00E-05	4.28E-04	--	Uncharacterised protein family (UPF0197)
EVM0007827	19.19	241.11	-3.65	5.00E-05	4.28E-04	--	Lipase (class 3)
EVM0000032	1.27	15.56	-3.61	5.00E-05	4.28E-04	--	--
EVM0009709	4.74	56.94	-3.59	5.00E-05	4.28E-04	--	--
EVM0001299	5.89	70.13	-3.57	5.00E-05	4.28E-04	Posttranslational modification, protein turnover, chaperones	Isoprenylcysteine carboxyl methyltransferase (ICMT) family
EVM0007867	6.07	71.97	-3.57	5.00E-05	4.28E-04	--	--
EVM0002760	6.22	73.45	-3.56	5.00E-05	4.28E-04	--	--
EVM0009999	3.31	38.62	-3.54	5.00E-05	4.28E-04	--	Rare lipoprotein A (RlpA)-like double-psi beta-barrel
EVM0010589	69.26	799.29	-3.53	5.00E-05	4.28E-04	--	bZIP transcription factor
EVM0004729	34.15	382.70	-3.49	5.00E-05	4.28E-04	--	Helix-turn-helix
EVM0002270	2.33	26.09	-3.48	8.00E-04	4.73E-03	--	--
EVM0001661	5.76	64.32	-3.48	5.00E-05	4.28E-04	General function prediction only	Kinase phosphorylation protein
EVM0008465	29.96	327.87	-3.45	5.00E-05	4.28E-04	RNA processing and modification	DEAD/DEAH box helicase
EVM0006922	10.21	104.81	-3.36	5.00E-05	4.28E-04	--	--
EVM0010939	111.27	1110.35	-3.32	1.15E-03	6.41E-03	--	--
EVM0008002	56.99	561.02	-3.30	5.00E-05	4.28E-04	General function prediction only	Flavodoxin
EVM0001240	14.04	136.58	-3.28	5.00E-05	4.28E-04	--	--

EVM0005700	24.94	242.19	-3.28	5.00E-05	4.28E-04	--	Conidiation protein 6
EVM0005127	77.37	748.41	-3.27	5.00E-05	4.28E-04	--	Gamma interferon inducible lysosomal thiol reductase (GILT)
EVM0002682	38.96	373.60	-3.26	5.00E-05	4.28E-04	Transcription	RNA polymerase Rpb6
EVM0005937	28.61	274.34	-3.26	5.00E-05	4.28E-04	--	--
EVM0004346	263.09	2506.01	-3.25	5.00E-05	4.28E-04	--	--
EVM0000370	71.38	677.74	-3.25	5.00E-05	4.28E-04	--	Fungal fruit body lectin
EVM0002438	5.86	53.98	-3.20	5.00E-05	4.28E-04	Secondary metabolites biosynthesis, transport and catabolism	short chain dehydrogenase
EVM0011230	8.09	74.23	-3.20	5.00E-05	4.28E-04	--	SNARE associated Golgi protein
EVM0006251	4.83	43.80	-3.18	5.00E-05	4.28E-04	Function unknown	Surfeit locus protein 6
EVM0003241	18.07	162.41	-3.17	5.00E-05	4.28E-04	--	Helix-loop-helix DNA-binding domain
EVM0005353	1.95	17.43	-3.16	5.00E-05	4.28E-04	Posttranslational modification, protein turnover, chaperones	Peptidase family M50
EVM0000142	2.60	22.34	-3.10	5.00E-05	4.28E-04	--	Putative phosphatase regulatory subunit
EVM0010264	22.62	191.62	-3.08	5.00E-05	4.28E-04	Function unknown	Thioesterase superfamily
EVM0004635	65.35	550.54	-3.07	5.00E-05	4.28E-04	--	--
EVM0003543	68.41	570.23	-3.06	5.00E-05	4.28E-04	--	--
EVM0002451	2.49	20.67	-3.05	5.00E-05	4.28E-04	--	--
EVM0006917	1.96	16.28	-3.05	5.00E-05	4.28E-04	--	--
EVM0003732	21.94	51.82	-1.24	5.00E-05	4.28E-04	--	--

EVM0002754	7.81	64.06	-3.04	5.00E-05	4.28E-04	General function prediction only	Ankyrin repeats (3 copies)
EVM0001814	5017.20	40681.30	-3.02	5.00E-05	4.28E-04	--	Ricin-type beta-trefoil lectin domain-like
EVM0008891	8.58	69.58	-3.02	5.00E-05	4.28E-04	Amino acid transport and metabolism	Histidine biosynthesis protein
EVM0000282	155.66	1258.86	-3.02	5.00E-05	4.28E-04	--	--
EVM0009146	0.39	3.17	-3.01	1.09E-02	4.01E-02	--	Glycosyl hydrolase family 61
EVM0001153	0.36	2.93	-3.01	1.00E-03	5.70E-03	--	--
EVM0007670	30.91	247.19	-3.00	5.00E-05	4.28E-04	--	Domain of unknown function (DUF4112)
EVM0001996	21.59	171.38	-2.99	5.00E-05	4.28E-04	Translation, ribosomal structure and biogenesis	SBDS protein C-terminal domain
EVM0005265	32.85	260.64	-2.99	5.00E-05	4.28E-04	--	--
EVM0010786	16.62	131.82	-2.99	5.00E-05	4.28E-04	--	Zinc finger, C3HC4 type (RING finger)
EVM0012244	0.58	4.57	-2.99	2.50E-04	1.77E-03	--	--
EVM0009491	5.39	41.64	-2.95	5.00E-05	4.28E-04	Secondary metabolites biosynthesis, transport and catabolism	Alcohol dehydrogenase GroES-like domain
EVM0009924	0.75	5.75	-2.93	9.50E-04	5.46E-03	--	--
EVM0004071	18.19	138.73	-2.93	5.00E-05	4.28E-04	Transcription	GATA zinc finger
EVM0010256	0.46	3.53	-2.93	5.00E-05	4.28E-04	General function prediction only	GMC oxidoreductase

EVM0010422	5.58	42.54	-2.93	5.00E-05	4.28E-04	Posttranslational modification, protein turnover, chaperones	DnaJ domain
EVM0010125	8.62	64.59	-2.91	5.00E-05	4.28E-04	--	--
EVM0008632	3.35	24.92	-2.90	5.00E-05	4.28E-04	Posttranslational modification, protein turnover, chaperones	Isoprenylcysteine carboxyl methyltransferase (ICMT) family
EVM0002910	10.36	76.95	-2.89	5.00E-05	4.28E-04	--	--
EVM0005367	1.19	8.82	-2.89	5.00E-05	4.28E-04	--	--
EVM0008355	1.35	9.95	-2.89	5.00E-05	4.28E-04	--	--
EVM0010446	15.39	113.29	-2.88	5.00E-05	4.28E-04	Transcription	Homeobox KN domain
EVM0005014	1.97	14.41	-2.87	5.00E-05	4.28E-04	--	--
EVM0001622	46.31	334.99	-2.85	5.00E-05	4.28E-04	--	Ricin-type beta-trefoil lectin domain-like
EVM0003029	23.91	172.29	-2.85	5.00E-05	4.28E-04	Signal transduction mechanisms	Protein-tyrosine phosphatase
EVM0000847	2.12	15.22	-2.85	5.00E-05	4.28E-04	--	--
EVM0006132	24.32	174.52	-2.84	5.00E-05	4.28E-04	Intracellular trafficking, secretion, and vesicular transport	Peroxisomal biogenesis factor 11 (PEX11)
EVM0006981	20.16	144.03	-2.84	5.00E-05	4.28E-04	--	Rrp15p
EVM0000114	5.33	38.01	-2.84	5.00E-05	4.28E-04	Posttranslational modification, protein turnover, chaperones	ATPase family associated with various cellular activities (AAA)
EVM0003584	11.73	83.50	-2.83	5.00E-05	4.28E-04	Replication, recombination and repair	TatD related DNase

EVM0000144	6.29	44.38	-2.82	5.00E-05	4.28E-04	General function prediction only	Phenazine biosynthesis-like protein
EVM0000467	2.76	19.06	-2.79	5.00E-05	4.28E-04	--	--
EVM0002447	127.77	883.19	-2.79	5.00E-05	4.28E-04	--	--
EVM0002088	4.36	29.88	-2.78	1.29E-02	4.59E-02	--	--
EVM0002647	16.68	113.48	-2.77	5.00E-05	4.28E-04	--	Fungal Zn(2)-Cys(6) binuclear cluster domain
EVM0010870	17.58	119.36	-2.76	5.00E-05	4.28E-04	--	Conidiation protein 6
EVM0008337	15.72	106.59	-2.76	5.00E-05	4.28E-04	General function prediction only	--
EVM0005972	523.41	3525.14	-2.75	5.00E-05	4.28E-04	--	--
EVM0011432	7.75	52.00	-2.75	5.00E-05	4.28E-04	Lipid transport and metabolism	SacI homology domain
EVM0009852	9.77	65.38	-2.74	5.00E-05	4.28E-04	Transcription	Homeobox domain
EVM0007202	1.57	10.48	-2.74	5.00E-05	4.28E-04	--	non-haem dioxygenase in morphine synthesis N-terminal
EVM0008304	33.66	224.54	-2.74	5.00E-05	4.28E-04	Defense mechanisms	Dual specificity phosphatase, catalytic domain
EVM0009705	37.86	249.89	-2.72	5.00E-05	4.28E-04	--	--
EVM0009499	0.88	5.79	-2.72	8.20E-03	3.20E-02	General function prediction only	Phosphatidylethanolamine-binding protein
EVM0000914	22.79	150.35	-2.72	5.00E-05	4.28E-04	--	Peroxidase, family 2
EVM0012106	15.14	99.22	-2.71	5.00E-05	4.28E-04	Coenzyme transport and metabolism	Ketopantoate hydroxymethyltransferase
EVM0007187	2.41	15.76	-2.71	5.00E-05	4.28E-04	--	Pregnancy-associated plasma protein-A

EVM0009205	0.77	5.03	-2.71	6.15E-03	2.54E-02	--	--
EVM0006446	51.34	334.69	-2.70	5.00E-05	4.28E-04	--	FR47-like protein
EVM0004929	14.94	97.05	-2.70	5.00E-05	4.28E-04	General function prediction only	Ras family
EVM0008406	13.77	88.54	-2.69	5.00E-05	4.28E-04	Lipid transport and metabolism	AMP-binding enzyme
EVM0007650	57.53	369.31	-2.68	5.00E-05	4.28E-04	--	--
EVM0006820	17.51	112.24	-2.68	5.00E-05	4.28E-04	--	--
EVM0003361	10.01	64.08	-2.68	5.00E-05	4.28E-04	--	Lipoxygenase
EVM0007146	878.23	5607.20	-2.67	5.00E-05	4.28E-04	--	--
EVM0011786	13.44	85.69	-2.67	5.00E-05	4.28E-04	--	--
EVM0005012	2.15	13.66	-2.67	5.00E-05	4.28E-04	General function prediction only	Calcineurin-like phosphoesterase
EVM0008721	1.57	9.93	-2.66	5.00E-05	4.28E-04	Lipid transport and metabolism	Hydroxymethylglutaryl-coenzyme A synthase N terminal
EVM0008242	1.04	6.50	-2.65	5.00E-05	4.28E-04	--	Glycosyl hydrolase family 12
EVM0000445	21.36	133.41	-2.64	5.00E-05	4.28E-04	--	--
EVM0007507	17.68	110.38	-2.64	5.00E-05	4.28E-04	--	--
EVM0006282	1.37	8.53	-2.64	5.00E-05	4.28E-04	--	--
EVM0009090	19.21	118.55	-2.63	5.00E-05	4.28E-04	--	--
EVM0005323	3.08	18.96	-2.62	5.00E-05	4.28E-04	--	--
EVM0004221	7.22	44.37	-2.62	5.00E-05	4.28E-04	--	--
EVM0004778	31.63	194.28	-2.62	5.00E-05	4.28E-04	General function prediction only	Thioesterase superfamily
EVM0003455	4.82	29.51	-2.62	5.00E-05	4.28E-04	--	--
EVM0004341	81.75	497.62	-2.61	5.00E-05	4.28E-04	Function unknown	CSL zinc finger

EVM0011852	32.55	197.48	-2.60	5.00E-05	4.28E-04	--	--
EVM0002297	215.72	1306.69	-2.60	5.00E-05	4.28E-04	--	--
EVM0003306	30.60	185.23	-2.60	5.00E-05	4.28E-04	Intracellular trafficking, secretion, and vesicular transport	Snf7
EVM0002614	10.09	61.03	-2.60	5.00E-05	4.28E-04	Lipid transport and metabolism	Phospholipase/Carboxylesterase
EVM0010217	41.60	251.32	-2.59	5.00E-05	4.28E-04	--	Domain of unknown function (DUF4139)
EVM0010131	57.44	346.41	-2.59	5.00E-05	4.28E-04	--	--
EVM0008150	5.20	31.31	-2.59	5.00E-05	4.28E-04	Energy production and conversion	Aldo/keto reductase family
EVM0008586	12.89	77.50	-2.59	5.00E-05	4.28E-04	--	Chromatin remodelling complex Rsc7/Swp82 subunit
EVM0006542	48.12	288.67	-2.58	5.00E-05	4.28E-04	--	Ricin-type beta-trefoil lectin domain
EVM0009699	51.54	308.49	-2.58	5.00E-05	4.28E-04	Posttranslational modification, protein turnover, chaperones	Proteasome subunit
EVM0000332	39.29	233.59	-2.57	5.00E-05	4.28E-04	Lipid transport and metabolism	Thiolase, N-terminal domain
EVM0004176	228.07	1351.39	-2.57	5.00E-05	4.28E-04	--	--
EVM0008894	19.08	113.02	-2.57	5.00E-05	4.28E-04	--	Pterin 4 alpha carbinolamine dehydratase
EVM0009527	4.57	26.90	-2.56	5.00E-05	4.28E-04	Replication, recombination and repair	Cid1 family poly A polymerase

EVM0006770	2.41	14.19	-2.56	5.00E-05	4.28E-04	--	Domain of unknown function (DUF3415)
EVM0003318	29.54	173.34	-2.55	5.00E-05	4.28E-04	--	Autophagy protein 16 (ATG16)
EVM0003828	8.48	49.52	-2.55	5.00E-05	4.28E-04	--	Membrane bound O-acyl transferase family
EVM0002081	2.60	15.03	-2.53	5.00E-05	4.28E-04	Signal transduction mechanisms	FHA domain
EVM0005185	75.62	436.79	-2.53	5.00E-05	4.28E-04	Amino acid transport and metabolism	Aminotransferase class-V
EVM0006915	19.97	115.05	-2.53	5.00E-05	4.28E-04	--	Velvet factor
EVM0003772	3.78	21.75	-2.53	5.00E-05	4.28E-04	--	--
EVM0011121	3.69	21.23	-2.52	5.00E-05	4.28E-04	--	--
EVM0010530	40.07	230.12	-2.52	5.00E-05	4.28E-04	--	--
EVM0007419	16.83	96.38	-2.52	5.00E-05	4.28E-04	--	--
EVM0007852	779.47	4462.47	-2.52	5.00E-05	4.28E-04	Posttranslational modification, protein turnover, chaperones	DnaJ C terminal domain
EVM0008058	6.15	35.18	-2.52	5.00E-05	4.28E-04	--	--
EVM0009876	9.36	53.35	-2.51	5.00E-05	4.28E-04	Translation, ribosomal structure and biogenesis	eRF1 domain 1
EVM0002150	131.46	745.19	-2.50	5.00E-05	4.28E-04	General function prediction only	RNA recognition motif. (a.k.a. RRM, RBD, or RNP domain)
EVM0006838	2.02	11.40	-2.50	5.00E-05	4.28E-04	--	--
EVM0004717	5.74	32.28	-2.49	5.00E-05	4.28E-04	--	Protein tyrosine kinase
EVM0000819	3.52	19.76	-2.49	5.00E-05	4.28E-04	Transcription	SNF2 family N-terminal domain

EVM0008863	37.02	207.28	-2.49	5.00E-05	4.28E-04	--	Protein of unknown function (DUF3759)
EVM0007977	161.62	901.87	-2.48	5.00E-05	4.28E-04	--	--
EVM0006195	17.40	97.00	-2.48	5.00E-05	4.28E-04	--	pre-mRNA splicing factor component
EVM0004965	3.40	18.85	-2.47	5.00E-05	4.28E-04	Function unknown	Tho complex subunit 7
EVM0009031	4.48	24.64	-2.46	5.00E-05	4.28E-04	--	Glycosyl hydrolases family 43
EVM0009471	15.74	86.16	-2.45	5.00E-05	4.28E-04	--	--
EVM0011795	45.31	246.67	-2.44	5.00E-05	4.28E-04	--	--
EVM0004745	6.97	37.81	-2.44	5.00E-05	4.28E-04	--	--
EVM0005254	38.61	209.01	-2.44	5.00E-05	4.28E-04	--	--
EVM0009138	2.81	15.19	-2.44	5.00E-05	4.28E-04	--	--
EVM0006949	41.28	222.96	-2.43	5.00E-05	4.28E-04	--	N-terminal domain of ribose phosphate pyrophosphokinase
EVM0010152	4.67	25.08	-2.43	5.00E-05	4.28E-04	--	--
EVM0004565	1.88	10.12	-2.42	5.00E-05	4.28E-04	--	Domain of unknown function (DUF4139)
EVM0002391	399.28	2143.23	-2.42	5.00E-05	4.28E-04	--	--
EVM0011516	8.26	44.14	-2.42	5.00E-05	4.28E-04	--	C2H2-type zinc finger
EVM0003567	0.54	2.88	-2.42	5.00E-05	4.28E-04	--	Tryptophan halogenase
EVM0010548	17.96	95.33	-2.41	5.00E-05	4.28E-04	--	--
EVM0010001	6.31	33.16	-2.39	5.00E-05	4.28E-04	Translation, ribosomal structure and biogenesis	Endoribonuclease L-PSP
EVM0001342	29.29	153.60	-2.39	5.00E-05	4.28E-04	Defense mechanisms	TB2/DP1, HVA22 family
EVM0007661	8.33	43.70	-2.39	5.00E-05	4.28E-04	--	C2 domain
EVM0008650	53.76	281.20	-2.39	5.00E-05	4.28E-04	--	--

EVM0002822	9.96	52.08	-2.39	5.00E-05	4.28E-04	--	Caspase domain
EVM0005881	3.51	18.31	-2.38	5.00E-05	4.28E-04	--	Small nuclear RNA activating complex (SNAPc), subunit SNAP43
EVM0007292	2.97	15.51	-2.38	5.00E-05	4.28E-04	General function prediction only	Domain of unknown function (DUF4110)
EVM0007572	2.86	14.88	-2.38	5.00E-05	4.28E-04	--	--
EVM0004357	134.54	700.77	-2.38	5.00E-05	4.28E-04	--	--
EVM0006881	12.47	64.73	-2.38	5.00E-05	4.28E-04	--	NUDIX domain
EVM0003461	19.88	103.12	-2.38	5.00E-05	4.28E-04	Intracellular trafficking, secretion, and vesicular transport	Annexin
EVM0011417	13.68	70.83	-2.37	5.00E-05	4.28E-04	Energy production and conversion	Aldo/keto reductase family
EVM0011506	3.88	20.07	-2.37	5.00E-05	4.28E-04	--	BTB/POZ domain
EVM0010360	2.34	12.07	-2.37	1.00E-03	5.70E-03	--	CFEM domain
EVM0000685	3.99	20.58	-2.37	5.00E-05	4.28E-04	Carbohydrate transport and metabolism	Transaldolase
EVM0010440	49.84	256.28	-2.36	5.00E-05	4.28E-04	--	Cytochrome c/c1 heme lyase
EVM0002660	9.50	48.78	-2.36	5.00E-05	4.28E-04	Chromatin structure and dynamics	MOZ/SAS family
EVM0006723	155.42	794.72	-2.35	5.00E-05	4.28E-04	--	Conidiation protein 6
EVM0010229	34.86	178.00	-2.35	5.00E-05	4.28E-04	General function prediction only	Zinc-binding dehydrogenase
EVM0008938	1.20	6.13	-2.35	3.75E-03	1.70E-02	--	--
EVM0010957	21.02	107.26	-2.35	5.00E-05	4.28E-04	Transcription	Myb-like DNA-binding domain

EVM0001277	4.40	22.39	-2.35	5.00E-05	4.28E-04	--	--
EVM0003758	2.88	14.66	-2.35	5.00E-05	4.28E-04	--	--
EVM0009537	2.64	13.45	-2.35	7.30E-03	2.91E-02	--	--
EVM0008877	12.60	64.05	-2.35	5.00E-05	4.28E-04	--	--
EVM0004716	7.94	40.31	-2.34	5.00E-05	4.28E-04	Replication, recombination and repair	Exonuclease
EVM0000132	50.41	253.39	-2.33	5.00E-05	4.28E-04	--	--
EVM0003103	59.11	297.01	-2.33	5.00E-05	4.28E-04	--	--
EVM0009546	11.02	54.96	-2.32	5.00E-05	4.28E-04	--	Fungal specific transcription factor domain
EVM0007669	0.31	1.56	-2.31	4.40E-03	1.94E-02	General function prediction only	Zinc-finger double domain
EVM0000116	3.34	16.56	-2.31	1.00E-04	7.96E-04	--	--
EVM0006626	51.89	257.00	-2.31	5.00E-05	4.28E-04	--	--
EVM0007461	4.12	20.39	-2.31	5.00E-05	4.28E-04	General function prediction only	Aldo/keto reductase family
EVM0005865	439.25	2171.68	-2.31	4.50E-04	2.92E-03	General function prediction only	Proteolipid membrane potential modulator
EVM0010107	92.48	452.80	-2.29	5.00E-05	4.28E-04	Signal transduction mechanisms	EF hand
EVM0005796	1156.13	5651.83	-2.29	5.00E-05	4.28E-04	Translation, ribosomal structure and biogenesis	60s Acidic ribosomal protein
EVM0011171	1.31	6.42	-2.29	5.00E-05	4.28E-04	--	--
EVM0011115	2.56	12.52	-2.29	5.00E-05	4.28E-04	--	--
EVM0002995	8.80	42.90	-2.28	5.00E-05	4.28E-04	RNA processing and modification	FF domain

EVM0000062	20.49	99.66	-2.28	5.00E-05	4.28E-04	--	--
EVM0000351	10.15	49.30	-2.28	5.00E-05	4.28E-04	--	Dyp-type peroxidase family
EVM0005078	1.53	7.42	-2.28	1.00E-04	7.96E-04	Cell cycle control, cell division, chromosome partitioning	HORMA domain
EVM0011013	14.04	67.94	-2.28	2.25E-03	1.12E-02	Function unknown	COQ9
EVM0003267	9620.57	46550.10	-2.27	5.00E-05	4.28E-04	--	Conidiation protein 6
EVM0004775	20.46	98.91	-2.27	5.00E-05	4.28E-04	--	Conidiation protein 6
EVM0002596	0.45	2.18	-2.27	9.50E-04	5.46E-03	--	Phosphotransferase enzyme family
EVM0009788	218.27	1054.21	-2.27	5.00E-05	4.28E-04	--	--
EVM0002460	86.36	416.72	-2.27	1.00E-04	7.96E-04	General function prediction only	Proteolipid membrane potential modulator
EVM0004549	312.60	1507.81	-2.27	5.00E-05	4.28E-04	Lipid transport and metabolism	HIUase/Transthyretin family
EVM0010543	1.31	6.31	-2.27	5.00E-05	4.28E-04	--	--
EVM0008461	9.67	46.45	-2.26	5.00E-05	4.28E-04	--	Putative snoRNA binding domain
EVM0002926	233.05	1117.65	-2.26	5.00E-05	4.28E-04	General function prediction only	Enoyl-(Acyl carrier protein) reductase
EVM0000147	1.41	6.75	-2.26	5.00E-05	4.28E-04	--	--
EVM0002338	10.46	49.96	-2.26	5.00E-05	4.28E-04	Lipid transport and metabolism	PAP2 superfamily
EVM0006839	2.40	11.45	-2.26	5.00E-05	4.28E-04	--	--
EVM0009347	15.04	71.76	-2.25	5.00E-05	4.28E-04	--	WH2 motif
EVM0001964	8.12	38.57	-2.25	5.00E-05	4.28E-04	--	--

EVM0007348	59.12	280.71	-2.25	5.00E-05	4.28E-04	General function prediction only	Dienelactone hydrolase family
EVM0004228	10.15	48.12	-2.25	5.00E-05	4.28E-04	Translation, ribosomal structure and biogenesis	Brix domain
EVM0006689	17.21	81.39	-2.24	5.00E-05	4.28E-04	General function prediction only	--
EVM0001350	357.09	1686.44	-2.24	5.00E-05	4.28E-04	--	CsbD-like
EVM0005968	6.06	28.61	-2.24	5.00E-05	4.28E-04	--	--
EVM0008982	1.33	6.25	-2.23	5.00E-05	4.28E-04	--	--
EVM0003699	4.59	21.56	-2.23	5.00E-05	4.28E-04	RNA processing and modification	Adenosine-deaminase (editase) domain
EVM0006292	2.18	10.24	-2.23	5.00E-05	4.28E-04	--	Fungal Zn(2)-Cys(6) binuclear cluster domain
EVM0009995	5.73	26.87	-2.23	5.00E-05	4.28E-04	RNA processing and modification	WD domain, G-beta repeat
EVM0006174	3.97	18.64	-2.23	5.00E-05	4.28E-04	--	--
EVM0009798	42.06	197.31	-2.23	5.00E-05	4.28E-04	RNA processing and modification	RNA recognition motif. (a.k.a. RRM, RBD, or RNP domain)
EVM0007949	12.09	56.70	-2.23	5.00E-05	4.28E-04	RNA processing and modification	WD domain, G-beta repeat
EVM0010770	48.97	229.46	-2.23	5.00E-05	4.28E-04	--	--
EVM0009589	32.18	150.77	-2.23	5.00E-05	4.28E-04	--	--
EVM0000266	17.18	80.40	-2.23	5.00E-05	4.28E-04	--	--
EVM0003550	5.11	23.90	-2.23	5.00E-05	4.28E-04	--	--
EVM0009311	67.02	313.03	-2.22	5.00E-05	4.28E-04	--	Etoposide-induced protein 2.4 (EI24)

EVM0006056	0.88	4.12	-2.22	7.50E-04	4.49E-03	--	--
EVM0007798	2.43	11.32	-2.22	5.00E-05	4.28E-04	Transcription	TEA/ATTS domain family
EVM0004688	26.10	121.36	-2.22	5.00E-05	4.28E-04	--	--
EVM0003326	29.04	134.59	-2.21	5.00E-05	4.28E-04	General function prediction only	R3H domain
EVM0001192	69.88	323.20	-2.21	5.00E-05	4.28E-04	Transcription	RNA polymerase Rpb3/Rpb11 dimerisation domain
EVM0005036	19.90	91.96	-2.21	5.00E-05	4.28E-04	--	--
EVM0005583	13.13	60.65	-2.21	5.00E-05	4.28E-04	--	--
EVM0006543	1.87	8.60	-2.20	5.00E-05	4.28E-04	--	--
EVM0001632	27.70	127.49	-2.20	5.00E-05	4.28E-04	--	--
EVM0011248	21.44	98.36	-2.20	8.65E-03	3.34E-02	--	--
EVM0011482	17.82	81.69	-2.20	5.00E-05	4.28E-04	Function unknown	WD domain, G-beta repeat
EVM0004258	26.00	119.09	-2.20	5.00E-05	4.28E-04	--	--
EVM0001382	6.72	30.76	-2.19	5.00E-05	4.28E-04	--	--
EVM0006992	9.57	43.56	-2.19	5.00E-05	4.28E-04	--	Cytochrome P450
EVM0000329	4.50	20.38	-2.18	5.00E-05	4.28E-04	--	--
EVM0011804	4.66	21.10	-2.18	5.00E-05	4.28E-04	--	--
EVM0008113	5.78	26.14	-2.18	5.00E-05	4.28E-04	--	FAD binding domain
EVM0010481	4.81	21.77	-2.18	5.00E-05	4.28E-04	--	F-box-like
EVM0008272	0.48	2.19	-2.18	3.00E-04	2.07E-03	--	--
EVM0008558	2.24	10.12	-2.18	5.00E-05	4.28E-04	--	Glyoxal oxidase N-terminus
EVM0007254	8026.47	36270.50	-2.18	5.00E-05	4.28E-04	--	Ribosomal protein S30
EVM0005285	1421.95	6412.12	-2.17	5.00E-05	4.28E-04	--	--
EVM0009404	14.69	66.19	-2.17	5.00E-05	4.28E-04	General function prediction only	Dip2/Utp12 Family

EVM0001259	8.67	39.05	-2.17	5.00E-05	4.28E-04	General function prediction only	WD domain, G-beta repeat
EVM0006570	792.75	3557.49	-2.17	5.00E-05	4.28E-04	--	Pyridoxamine 5'-phosphate oxidase
EVM0002266	221.11	990.92	-2.16	5.00E-05	4.28E-04	--	Protein of unknown function (DUF3429)
EVM0009720	9.13	40.88	-2.16	5.00E-05	4.28E-04	Cell cycle control, cell division, chromosome partitioning	LYAR-type C2HC zinc finger
EVM0005110	30.58	136.78	-2.16	5.00E-05	4.28E-04	Transcription	--
EVM0004224	1.51	6.74	-2.16	5.00E-05	4.28E-04	--	--
EVM0001827	2.49	11.15	-2.16	5.00E-05	4.28E-04	Replication, recombination and repair	Helicase conserved C-terminal domain
EVM0000219	52.84	235.65	-2.16	5.00E-05	4.28E-04	General function prediction only	Cyclin
EVM0001614	2.25	10.01	-2.16	5.00E-05	4.28E-04	--	--
EVM0001981	5.86	26.02	-2.15	5.00E-05	4.28E-04	--	HD domain
EVM0004173	11.83	52.31	-2.15	5.00E-05	4.28E-04	--	--
EVM0008334	87.93	387.81	-2.14	5.00E-05	4.28E-04	--	--
EVM0004672	31.96	140.79	-2.14	5.00E-05	4.28E-04	--	--
EVM0002798	5.25	23.12	-2.14	5.00E-05	4.28E-04	--	F-box-like
EVM0005429	9.52	41.78	-2.13	5.00E-05	4.28E-04	Transcription	CCAAT-binding transcription factor (CBF-B/NF-YA) subunit B
EVM0009550	12.43	24.89	-1.00	4.00E-04	2.65E-03	--	--
EVM0005497	51.81	226.99	-2.13	5.00E-05	4.28E-04	--	--

EVM0005377	4.34	18.99	-2.13	5.00E-05	4.28E-04	--	Cellulase (glycosyl hydrolase family 5)
EVM0009845	12.24	53.49	-2.13	5.00E-05	4.28E-04	--	--
EVM0000913	21.01	91.71	-2.13	5.00E-05	4.28E-04	--	--
EVM0008230	15.30	66.75	-2.13	5.00E-05	4.28E-04	Transcription	Maf1 regulator
EVM0005976	75.67	328.96	-2.12	5.00E-05	4.28E-04	General function prediction only	Sugar (and other) transporter
EVM0007199	136.75	592.09	-2.11	5.00E-05	4.28E-04	General function prediction only	Yippee zinc-binding/DNA-binding /Mis18, centromere assembly
EVM0010015	0.34	1.46	-2.11	1.21E-02	4.36E-02	--	--
EVM0007358	9.21	39.84	-2.11	5.00E-05	4.28E-04	--	--
EVM0005054	24.15	104.28	-2.11	5.00E-05	4.28E-04	General function prediction only	C2 domain
EVM0003528	111.38	480.04	-2.11	5.00E-05	4.28E-04	--	--
EVM0000907	1.40	6.04	-2.11	5.00E-05	4.28E-04	--	--
EVM0005402	1.59	6.87	-2.11	5.00E-05	4.28E-04	--	F-box domain
EVM0000131	2.44	10.51	-2.11	3.50E-04	2.36E-03	--	--
EVM0000628	22.06	94.65	-2.10	5.00E-05	4.28E-04	--	--
EVM0010403	6.85	29.37	-2.10	5.00E-05	4.28E-04	--	--
EVM0003631	7.32	28.73	-1.97	2.50E-04	1.77E-03	--	--
EVM0007878	13.71	58.62	-2.10	5.00E-05	4.28E-04	Posttranslational modification, protein turnover, chaperones	BCS1 N terminal

EVM0003792	63.49	271.44	-2.10	5.00E-05	4.28E-04	Posttranslational modification, protein turnover, chaperones	ATPase family associated with various cellular activities (AAA)
EVM0008730	8.47	36.22	-2.10	5.00E-05	4.28E-04	--	--
EVM0007261	283.14	1209.34	-2.09	5.00E-05	4.28E-04	--	--
EVM0001122	37.62	160.49	-2.09	5.00E-05	4.28E-04	RNA processing and modification	Fibrillarin
EVM0012205	85.65	365.17	-2.09	5.00E-05	4.28E-04	Function unknown	RHS Repeat
EVM0007696	7.81	33.26	-2.09	5.00E-05	4.28E-04	--	Domain of unknown function (DUF4139)
EVM0007162	15.05	64.07	-2.09	5.00E-05	4.28E-04	RNA processing and modification	Helicase associated domain (HA2)
EVM0004297	32.24	137.10	-2.09	5.00E-05	4.28E-04	--	BTG family
EVM0010534	43.81	185.63	-2.08	5.00E-05	4.28E-04	--	--
EVM0008237	17.56	74.37	-2.08	5.00E-05	4.28E-04	--	RNA ligase
EVM0006494	13.62	57.63	-2.08	5.00E-05	4.28E-04	Chromatin structure and dynamics	Sas10 C-terminal domain
EVM0005139	323.22	1364.38	-2.08	5.00E-05	4.28E-04	--	--
EVM0005280	0.62	2.59	-2.07	1.35E-03	7.33E-03	--	Phosphotransferase enzyme family
EVM0009462	8.04	33.76	-2.07	5.00E-05	4.28E-04	--	--
EVM0001526	3.41	14.26	-2.06	5.00E-05	4.28E-04	--	--
EVM0008356	13.38	55.97	-2.06	5.00E-05	4.28E-04	Function unknown	Utp11 protein
EVM0001591	1.97	8.24	-2.06	5.00E-05	4.28E-04	--	--
EVM0008966	24.44	102.00	-2.06	5.00E-05	4.28E-04	--	--

EVM0002711	20.51	85.56	-2.06	5.00E-05	4.28E-04	General function prediction only	La domain
EVM0003560	18.66	77.76	-2.06	2.35E-03	1.16E-02	--	G-patch domain
EVM0009085	245.65	1022.86	-2.06	5.00E-05	4.28E-04	--	--
EVM0002160	5.93	24.59	-2.05	5.00E-05	4.28E-04	--	--
EVM0004958	8.98	37.18	-2.05	5.00E-05	4.28E-04	--	Regulator of G protein signaling domain
EVM0008871	70.49	291.35	-2.05	5.00E-05	4.28E-04	General function prediction only	Ras family
EVM0002141	2.78	11.49	-2.05	1.00E-04	7.96E-04	--	--
EVM0003995	15.66	64.64	-2.05	5.00E-05	4.28E-04	--	--
EVM0002673	29.18	120.27	-2.04	5.00E-05	4.28E-04	--	--
EVM0010419	3.65	15.01	-2.04	5.00E-05	4.28E-04	--	Mis12-Mtw1 protein family
EVM0009591	10.09	41.55	-2.04	5.00E-05	4.28E-04	Amino acid transport and metabolism	Homocysteine S-methyltransferase
EVM0006805	100.27	412.25	-2.04	5.00E-05	4.28E-04	Lipid transport and metabolism	Alpha/beta hydrolase family
EVM0001025	14.50	59.58	-2.04	5.00E-05	4.28E-04	--	Zinc-binding dehydrogenase
EVM0008442	16.24	66.68	-2.04	5.00E-05	4.28E-04	--	--
EVM0001552	13.45	55.19	-2.04	5.00E-05	4.28E-04	#N/A	#N/A
EVM0003805	1.74	7.13	-2.04	5.00E-05	4.28E-04	Transcription	HMG (high mobility group) box
EVM0006700	186.14	763.41	-2.04	5.00E-05	4.28E-04	--	Aconitase family (aconitate hydratase)
EVM0005625	1.91	7.82	-2.03	5.00E-05	4.28E-04	--	--
EVM0008511	31.79	129.79	-2.03	5.00E-05	4.28E-04	RNA processing and modification	Ribosomal RNA-processing protein 7 (RRP7)

EVM0007186	6.99	28.49	-2.03	5.00E-05	4.28E-04	--	--
EVM0003226	25.18	102.62	-2.03	5.00E-05	4.28E-04	--	Protein of unknown function (DUF3602)
EVM0009334	7.80	31.79	-2.03	5.00E-05	4.28E-04	--	--
EVM0010883	215.53	877.66	-2.03	5.00E-05	4.28E-04	Cell wall/membrane/envelope biogenesis	Acetyltransferase (GNAT) family
EVM0002867	30.59	124.43	-2.02	5.00E-05	4.28E-04	Lipid transport and metabolism	MBOAT, membrane-bound O-acyltransferase family
EVM0010074	5.91	23.97	-2.02	5.00E-05	4.28E-04	--	--
EVM0002372	6.14	24.88	-2.02	5.00E-05	4.28E-04	--	Cytochrome P450
EVM0004156	0.66	2.67	-2.02	5.00E-05	4.28E-04	--	--
EVM0009322	6.81	27.51	-2.02	5.00E-05	4.28E-04	--	--
EVM0008748	5.95	24.02	-2.01	5.00E-05	4.28E-04	--	--
EVM0003817	5.13	20.70	-2.01	5.00E-05	4.28E-04	--	--
EVM0009820	110.63	446.14	-2.01	5.00E-05	4.28E-04	--	Acetyltransferase (GNAT) domain
EVM0006719	1.65	6.64	-2.01	1.35E-02	4.76E-02	--	Fungal hydrophobin
EVM0011088	21.26	85.72	-2.01	5.00E-05	4.28E-04	Translation, ribosomal structure and biogenesis	Gar1/Naf1 RNA binding region
EVM0000363	12.35	49.78	-2.01	5.00E-05	4.28E-04	Posttranslational modification, protein turnover, chaperones	Zinc knuckle
EVM0007331	2.74	11.03	-2.01	5.00E-05	4.28E-04	--	--
EVM0003705	22.24	89.60	-2.01	5.00E-05	4.28E-04	--	alpha/beta hydrolase fold
EVM0001452	5.34	21.38	-2.00	5.00E-05	4.28E-04	--	--

EVM0002890	0.57	2.26	-2.00	1.25E-03	6.87E-03	Transcription	HMG (high mobility group) box
EVM0001316	3.10	12.36	-2.00	5.00E-05	4.28E-04	--	--
EVM0010252	34.88	139.03	-2.00	5.00E-05	4.28E-04	--	PX domain
EVM0007517	98.50	392.66	-2.00	5.00E-05	4.28E-04	--	--
EVM0005547	163.72	652.27	-1.99	6.00E-04	3.72E-03	Secondary metabolites biosynthesis, transport and catabolism	Aromatic amino acid lyase
EVM0005351	3.01	11.96	-1.99	5.00E-05	4.28E-04	--	--
EVM0003323	9.46	37.53	-1.99	5.00E-05	4.28E-04	--	--
EVM0000366	1.37	5.43	-1.99	2.00E-04	1.46E-03	--	--
EVM0010012	6.44	25.48	-1.99	5.00E-05	4.28E-04	--	Formin Homology Region 1
EVM0000406	3.96	15.66	-1.98	5.00E-05	4.28E-04	--	Protein kinase domain
EVM0001016	3.40	13.39	-1.98	5.00E-05	4.28E-04	--	--
EVM0008088	4.16	16.37	-1.98	5.00E-05	4.28E-04	--	--
EVM0007834	30.88	121.22	-1.97	5.00E-05	4.28E-04	--	ADP-ribosylglycohydrolase
EVM0011475	5.23	20.53	-1.97	5.00E-05	4.28E-04	--	--
EVM0006590	2.78	10.89	-1.97	2.50E-04	1.77E-03	--	--
EVM0004906	111.77	436.41	-1.97	5.00E-05	4.28E-04	--	Fungal specific transcription factor domain
EVM0004482	1.61	6.26	-1.96	2.70E-03	1.30E-02	--	--
EVM0005279	429.38	1672.61	-1.96	5.00E-05	4.28E-04	--	Stress responsive A/B Barrel Domain
EVM0009109	42.66	165.97	-1.96	5.00E-05	4.28E-04	--	Glycosyl hydrolases family 43
EVM0010765	105.84	411.51	-1.96	5.00E-05	4.28E-04	Posttranslational modification, protein turnover, chaperones	Prefoldin subunit

EVM0007388	15.18	58.82	-1.95	5.00E-05	4.28E-04	--	--
EVM0012116	43.91	170.07	-1.95	5.00E-05	4.28E-04	Posttranslational modification, protein turnover, chaperones	Redoxin
EVM0002792	3.70	14.31	-1.95	5.00E-05	4.28E-04	--	--
EVM0001520	2.87	11.11	-1.95	5.00E-05	4.28E-04	--	--
EVM0008447	47.15	182.04	-1.95	5.00E-05	4.28E-04	--	--
EVM0005454	49.40	190.24	-1.95	5.00E-05	4.28E-04	General function prediction only	Zinc finger, C2H2 type
EVM0006561	6.30	24.23	-1.94	5.00E-05	4.28E-04	--	--
EVM0001127	34.01	130.81	-1.94	5.00E-05	4.28E-04	General function prediction only	Ras family
EVM0002912	170.90	655.95	-1.94	5.00E-05	4.28E-04	Function unknown	Telomere stability and silencing
EVM0008190	18.34	70.35	-1.94	5.00E-05	4.28E-04	--	Uncharacterized conserved protein (DUF2156)
EVM0001740	29.14	111.64	-1.94	5.00E-05	4.28E-04	--	2OG-Fe(II) oxygenase superfamily
EVM0004489	27.83	106.41	-1.93	5.00E-05	4.28E-04	--	F-box-like
EVM0006968	5.84	22.33	-1.93	1.00E-04	7.96E-04	--	--
EVM0000880	16.93	64.72	-1.93	5.00E-05	4.28E-04	--	Glycosyl hydrolases family 15
EVM0001519	7.75	29.64	-1.93	5.00E-05	4.28E-04	--	--
EVM0000981	5.09	19.46	-1.93	2.50E-04	1.77E-03	--	--
EVM0002561	5.01	19.15	-1.93	5.00E-05	4.28E-04	--	--
EVM0000042	4.36	16.63	-1.93	2.50E-04	1.77E-03	--	--
EVM0002820	7.00	26.68	-1.93	5.00E-05	4.28E-04	--	--

EVM0007165	126.94	484.00	-1.93	5.00E-05	4.28E-04	Lipid transport and metabolism	Thiolase, C-terminal domain
EVM0010439	7.29	27.77	-1.93	5.00E-05	4.28E-04	--	--
EVM0001318	2840.53	10825.20	-1.93	3.50E-04	2.36E-03	Posttranslational modification, protein turnover, chaperones	Hsp70 protein
EVM0010645	9.49	36.14	-1.93	5.00E-05	4.28E-04	Cytoskeleton	Splicing factor, Prp19-binding domain
EVM0010702	11.31	43.06	-1.93	5.00E-05	4.28E-04	--	--
EVM0001379	2.59	9.82	-1.92	5.00E-05	4.28E-04	--	FHA domain
EVM0009023	11.18	42.43	-1.92	5.00E-05	4.28E-04	RNA processing and modification	Brix domain
EVM0011134	16.01	60.55	-1.92	5.00E-05	4.28E-04	Function unknown	NRDE-2, necessary for RNA interference
EVM0004486	31.62	119.41	-1.92	5.00E-05	4.28E-04	--	--
EVM0011517	17.34	65.39	-1.92	5.00E-05	4.28E-04	--	RNA12 protein
EVM0010513	6.04	22.76	-1.91	5.00E-05	4.28E-04	General function prediction only	Fumarylacetoacetate (FAA) hydrolase family
EVM0000393	940.82	3545.94	-1.91	5.00E-05	4.28E-04	--	--
EVM0002774	8.05	30.14	-1.91	5.00E-05	4.28E-04	--	--
EVM0010259	553.03	2070.81	-1.90	5.00E-05	4.28E-04	--	Domain of unknown function (DUF427)
EVM0009651	3.21	12.01	-1.90	5.00E-05	4.28E-04	Energy production and conversion	FAD binding domain
EVM0009399	19.92	74.45	-1.90	5.00E-05	4.28E-04	--	Mediator complex subunit 25 von Willebrand factor type A

EVM0007509	7.64	28.38	-1.89	5.00E-05	4.28E-04	--	--
EVM0011068	30.01	111.26	-1.89	5.00E-05	4.28E-04	Defense mechanisms	Dual specificity phosphatase, catalytic domain
EVM0000361	2.11	7.80	-1.89	5.00E-05	4.28E-04	--	--
EVM0001246	10.24	37.89	-1.89	5.00E-05	4.28E-04	--	--
EVM0002898	18.52	68.49	-1.89	5.00E-05	4.28E-04	Transcription	RNA polymerase Rpb3/RpoA insert domain
EVM0008826	76.50	282.46	-1.88	5.00E-05	4.28E-04	--	--
EVM0006068	2.89	10.66	-1.88	5.00E-05	4.28E-04	--	bZIP transcription factor
EVM0004340	200.70	740.12	-1.88	5.00E-05	4.28E-04	--	Thioesterase superfamily
EVM0003580	6.01	22.15	-1.88	5.00E-05	4.28E-04	--	--
EVM0007281	23.80	87.65	-1.88	5.00E-05	4.28E-04	Signal transduction mechanisms	Protein kinase domain
EVM0006734	218.84	804.37	-1.88	5.00E-05	4.28E-04	--	Complex 1 protein (LYR family)
EVM0005109	3.14	11.52	-1.88	5.00E-05	4.28E-04	--	--
EVM0007084	5.21	19.10	-1.87	5.00E-05	4.28E-04	--	--
EVM0001225	2.32	8.50	-1.87	1.85E-03	9.52E-03	--	CoA binding domain
EVM0004818	11.25	41.23	-1.87	5.00E-05	4.28E-04	#N/A	#N/A
EVM0007300	1.63	0.69	1.24	1.22E-02	4.38E-02	--	--
EVM0011344	9.73	35.54	-1.87	5.00E-05	4.28E-04	RNA processing and modification	Mpp10 protein
EVM0003322	5.49	20.02	-1.87	5.00E-05	4.28E-04	General function prediction only	Sugar (and other) transporter
EVM0001566	2.68	9.77	-1.87	5.00E-05	4.28E-04	General function prediction only	Sugar (and other) transporter

EVM0005665	27.37	99.77	-1.87	5.00E-05	4.28E-04	General function prediction only	Zinc finger, C2H2 type
EVM0006463	14.46	52.66	-1.86	5.00E-05	4.28E-04	--	Lactonase, 7-bladed beta-propeller
EVM0009977	3.18	11.57	-1.86	5.00E-05	4.28E-04	--	Ubiquitin 3 binding protein But2 C-terminal domain
EVM0004238	22.13	80.54	-1.86	5.00E-05	4.28E-04	--	SMI1 / KNR4 family (SUKH-1)
EVM0003844	9.48	34.29	-1.85	5.00E-05	4.28E-04	Function unknown	--
EVM0002302	18.03	65.06	-1.85	5.00E-05	4.28E-04	--	--
EVM0000150	91.56	328.27	-1.84	5.00E-05	4.28E-04	--	CFEM domain
EVM0005613	14.14	50.67	-1.84	5.00E-05	4.28E-04	--	--
EVM0007225	72.33	258.85	-1.84	5.00E-05	4.28E-04	--	Nuclear pore complex subunit Nro1
EVM0008999	8.53	30.49	-1.84	5.00E-05	4.28E-04	General function prediction only	Zinc finger, C2H2 type
EVM0008761	13.32	47.49	-1.83	5.00E-05	4.28E-04	--	Calcineurin-like phosphoesterase
EVM0002317	33.56	119.60	-1.83	5.00E-05	4.28E-04	--	--
EVM0006454	6.47	23.04	-1.83	5.00E-05	4.28E-04	--	--
EVM0004758	5.12	18.23	-1.83	5.00E-05	4.28E-04	--	Tryptophan halogenase
EVM0006946	18.89	67.22	-1.83	5.00E-05	4.28E-04	--	--
EVM0005691	0.55	1.94	-1.83	1.05E-03	5.94E-03	--	Cytochrome P450
EVM0003416	22.42	79.59	-1.83	5.00E-05	4.28E-04	--	--
EVM0009214	135.72	481.27	-1.83	5.00E-05	4.28E-04	General function prediction only	Casein kinase substrate phosphoprotein PP28
EVM0010935	21.44	76.02	-1.83	5.00E-05	4.28E-04	General function prediction only	U3-containing 90S pre-ribosomal complex subunit

EVM0007535	1.02	3.60	-1.82	1.40E-03	7.55E-03	--	--
EVM0008156	408.87	1448.07	-1.82	5.00E-05	4.28E-04	--	--
EVM0003616	51.99	184.12	-1.82	5.00E-05	4.28E-04	Transcription	HSF-type DNA-binding
EVM0011379	14.39	50.94	-1.82	5.00E-05	4.28E-04	--	non-haem dioxygenase in morphine synthesis N-terminal
EVM0000883	4.16	14.72	-1.82	5.00E-05	4.28E-04	Transcription	HMG (high mobility group) box
EVM0009678	4.30	15.21	-1.82	5.00E-05	4.28E-04	Coenzyme transport and metabolism	Aminotransferase class-V
EVM0009486	0.59	2.10	-1.82	1.29E-02	4.57E-02	--	--
EVM0000538	1.71	6.02	-1.82	5.00E-05	4.28E-04	--	Putative FMN-binding domain
EVM0010921	10.80	38.09	-1.82	5.00E-05	4.28E-04	--	--
EVM0003944	2.24	7.87	-1.82	5.00E-05	4.28E-04	--	--
EVM0007958	1.73	6.09	-1.81	5.00E-05	4.28E-04	Secondary metabolites biosynthesis, transport and catabolism	Multicopper oxidase
EVM0000762	166.04	582.58	-1.81	5.00E-05	4.28E-04	General function prediction only	Protein of unknown function (DUF1295)
EVM0008201	1.77	6.21	-1.81	3.00E-04	2.07E-03	--	--
EVM0011937	2.19	7.68	-1.81	5.00E-05	4.28E-04	--	--
EVM0004770	11.94	41.81	-1.81	5.00E-05	4.28E-04	--	WH1 domain
EVM0003548	9.49	33.24	-1.81	5.00E-05	4.28E-04	Signal transduction mechanisms	--
EVM0000593	97.57	341.37	-1.81	5.00E-05	4.28E-04	--	Protein of unknown function (DUF952)
EVM0007842	54.16	189.39	-1.81	5.00E-05	4.28E-04	--	Hus1-like protein

EVM0006117	11.59	40.49	-1.80	5.00E-05	4.28E-04	General function prediction only	Aldo/keto reductase family
EVM0008889	0.60	2.09	-1.80	1.55E-03	8.22E-03	--	Cytochrome P450
EVM0002548	15.32	53.40	-1.80	5.00E-05	4.28E-04	--	--
EVM0004054	399.42	1389.50	-1.80	5.00E-05	4.28E-04	Posttranslational modification, protein turnover, chaperones	Hsp20/alpha crystallin family
EVM0007045	33.05	114.95	-1.80	5.00E-05	4.28E-04	--	--
EVM0007616	18.76	65.25	-1.80	5.00E-05	4.28E-04	Transcription	MT-A70
EVM0002358	18.38	63.88	-1.80	5.00E-05	4.28E-04	--	--
EVM0010819	2.13	7.40	-1.80	5.00E-05	4.28E-04	--	--
EVM0003936	43.31	150.05	-1.79	5.00E-05	4.28E-04	General function prediction only	TPR repeat
EVM0002723	6.08	21.03	-1.79	5.00E-05	4.28E-04	--	--
EVM0007740	6.19	21.39	-1.79	5.00E-05	4.28E-04	--	--
EVM0002692	0.98	3.37	-1.79	2.00E-04	1.46E-03	Secondary metabolites biosynthesis, transport and catabolism	Cytochrome P450
EVM0006427	106.14	366.14	-1.79	5.00E-05	4.28E-04	Amino acid transport and metabolism	Amino acid permease
EVM0002722	3.38	11.67	-1.79	5.00E-05	4.28E-04	Secondary metabolites biosynthesis, transport and catabolism	Multicopper oxidase
EVM0006228	74.43	256.50	-1.78	5.00E-05	4.28E-04	Posttranslational modification, protein turnover, chaperones	Ubiquitin carboxyl-terminal hydrolase, family 1

EVM0005225	10.08	34.54	-1.78	5.00E-05	4.28E-04	--	Gti1/Pac2 family
EVM0005312	0.97	3.34	-1.78	1.50E-04	1.14E-03	--	DNA polymerase subunit Cdc27
EVM0000069	8.07	27.63	-1.78	5.00E-05	4.28E-04	--	--
EVM0004405	1.28	4.38	-1.77	1.40E-03	7.55E-03	--	--
EVM0010316	34.67	118.50	-1.77	5.00E-05	4.28E-04	--	Fungal fruit body lectin
EVM0006290	3.79	12.96	-1.77	5.00E-05	4.28E-04	General function prediction only	GMC oxidoreductase
EVM0006914	2.53	8.65	-1.77	5.00E-05	4.28E-04	--	--
EVM0000151	253.39	865.86	-1.77	5.00E-05	4.28E-04	Lipid transport and metabolism	Cytochrome b5-like Heme/Steroid binding domain
EVM0012239	2.11	7.20	-1.77	5.00E-05	4.28E-04	--	--
EVM0005335	7.90	26.98	-1.77	5.00E-05	4.28E-04	--	F-box-like
EVM0008324	2.39	8.16	-1.77	5.00E-05	4.28E-04	Secondary metabolites biosynthesis, transport and catabolism	Cytochrome P450
EVM0005952	6.02	20.55	-1.77	5.00E-05	4.28E-04	--	RNA ligase
EVM0008051	197.12	673.01	-1.77	5.00E-05	4.28E-04	--	SUR7/PalI family
EVM0008452	6.52	22.24	-1.77	5.00E-05	4.28E-04	--	--
EVM0011033	2.83	9.66	-1.77	5.00E-05	4.28E-04	--	--
EVM0010885	17.74	60.37	-1.77	5.00E-05	4.28E-04	Posttranslational modification, protein turnover, chaperones	BCS1 N terminal
EVM0010265	33.25	113.06	-1.77	5.00E-05	4.28E-04	General function prediction only	Conserved hypothetical ATP binding protein

EVM0011950	601.16	2042.67	-1.76	5.00E-05	4.28E-04	Posttranslational modification, protein turnover, chaperones	CS domain
EVM0011048	3.13	10.63	-1.76	2.65E-03	1.28E-02	--	--
EVM0004305	0.67	2.27	-1.76	3.50E-04	2.36E-03	General function prediction only	GMC oxidoreductase
EVM0005902	10.21	34.59	-1.76	5.00E-05	4.28E-04	--	--
EVM0000869	161.53	545.40	-1.76	5.00E-05	4.28E-04	--	--
EVM0005044	7.86	26.51	-1.75	5.00E-05	4.28E-04	--	SAC3/GANP/Nin1/mts3/eIF-3 p25 family
EVM0009757	152.11	512.66	-1.75	5.00E-05	4.28E-04	--	--
EVM0002413	3.62	12.17	-1.75	5.00E-05	4.28E-04	--	Cellulase (glycosyl hydrolase family 5)
EVM0004513	60.65	204.10	-1.75	5.00E-05	4.28E-04	--	pfkB family carbohydrate kinase
EVM0007562	20.87	70.15	-1.75	5.00E-05	4.28E-04	--	--
EVM0007241	0.75	2.51	-1.75	1.75E-03	9.08E-03	--	--
EVM0010148	191.45	643.25	-1.75	5.00E-05	4.28E-04	--	--
EVM0008404	13.39	44.93	-1.75	5.00E-05	4.28E-04	RNA processing and modification	BING4CT (NUC141) domain
EVM0008819	1.91	6.41	-1.74	5.00E-05	4.28E-04	--	--
EVM0011527	1186.58	3967.40	-1.74	5.00E-05	4.28E-04	Signal transduction mechanisms	EF hand
EVM0011961	104.37	348.96	-1.74	5.00E-05	4.28E-04	General function prediction only	Zinc finger C-x8-C-x5-C-x3-H type (and similar)
EVM0002923	5995.82	20036.90	-1.74	5.00E-05	4.28E-04	--	CsbD-like
EVM0004611	4.62	15.44	-1.74	5.00E-05	4.28E-04	--	Ferritin-like domain

EVM0006867	13.46	44.95	-1.74	5.00E-05	4.28E-04	--	--
EVM0001681	258.91	863.46	-1.74	5.00E-05	4.28E-04	Intracellular trafficking, secretion, and vesicular transport	Microsomal signal peptidase 12 kDa subunit (SPC12)
EVM0004358	181.56	604.80	-1.74	5.00E-05	4.28E-04	--	Heat shock factor binding protein 1
EVM0010929	9.89	32.94	-1.74	5.00E-05	4.28E-04	--	--
EVM0007445	2.78	9.26	-1.73	5.00E-05	4.28E-04	--	--
EVM0000893	13.64	45.39	-1.73	5.00E-05	4.28E-04	--	--
EVM0008490	1.34	4.45	-1.73	2.00E-03	1.01E-02	--	--
EVM0009934	8.28	27.52	-1.73	5.00E-05	4.28E-04	--	Uncharacterized conserved protein (DUF2183)
EVM0010493	7.85	26.03	-1.73	2.50E-04	1.77E-03	--	--
EVM0000946	24.77	82.14	-1.73	5.00E-05	4.28E-04	Function unknown	Rgp1
EVM0011971	10.24	33.93	-1.73	5.00E-05	4.28E-04	Transcription	HMG (high mobility group) box
EVM0004944	2.89	9.58	-1.73	2.50E-04	1.77E-03	--	F-box-like
EVM0003453	156.96	519.42	-1.73	5.00E-05	4.28E-04	--	--
EVM0000189	4.32	14.29	-1.73	1.00E-04	7.96E-04	--	Glycosyl hydrolases family 43
EVM0003951	36.10	119.44	-1.73	5.00E-05	4.28E-04	Function unknown	RWD domain
EVM0003855	4.12	13.64	-1.73	5.00E-05	4.28E-04	Signal transduction mechanisms	Protein kinase domain
EVM0009807	2341.01	7739.50	-1.73	5.00E-05	4.28E-04	--	Mitochondrial F1-F0 ATP synthase subunit F of fungi
EVM0006896	412.41	1361.32	-1.72	7.50E-04	4.49E-03	--	Protein of unknown function (DUF4449)
EVM0004746	1.19	3.90	-1.72	9.75E-03	3.67E-02	--	--

EVM0000423	141.39	464.75	-1.72	5.00E-05	4.28E-04	#N/A	#N/A
EVM0005253	643.40	2114.47	-1.72	5.00E-05	4.28E-04	--	--
EVM0001355	262.49	861.95	-1.72	5.00E-05	4.28E-04	--	--
EVM0003403	1.91	6.27	-1.71	2.05E-03	1.03E-02	--	MYND finger
EVM0003549	2.98	9.78	-1.71	3.00E-04	2.07E-03	--	--
EVM0003569	2.34	7.69	-1.71	5.00E-05	4.28E-04	--	--
EVM0010137	3.24	10.59	-1.71	5.00E-05	4.28E-04	--	--
EVM0007361	17.76	57.99	-1.71	5.00E-05	4.28E-04	RNA processing and modification	DEAD/DEAH box helicase
EVM0000381	13.58	44.33	-1.71	5.00E-05	4.28E-04	Function unknown	Folate-sensitive fragile site protein Fra10Ac1
EVM0007086	70.66	230.49	-1.71	5.00E-05	4.28E-04	--	--
EVM0000419	56.33	183.64	-1.70	5.00E-05	4.28E-04	Function unknown	Complex 1 protein (LYR family)
EVM0006136	157.27	512.60	-1.70	5.00E-05	4.28E-04	--	--
EVM0001178	20.65	67.30	-1.70	5.00E-05	4.28E-04	--	--
EVM0003558	38.60	125.61	-1.70	5.00E-05	4.28E-04	--	Methyltransferase domain
EVM0007803	5.11	16.64	-1.70	3.50E-04	2.36E-03	--	--
EVM0001559	11.63	37.81	-1.70	5.00E-05	4.28E-04	Function unknown	Cysteine-rich secretory protein family
EVM0008798	1.18	3.82	-1.70	5.00E-05	4.28E-04	--	Gti1/Pac2 family
EVM0006869	426.64	1383.50	-1.70	5.00E-05	4.28E-04	General function prediction only	Ras family
EVM0009813	52.58	170.09	-1.69	5.00E-05	4.28E-04	--	--
EVM0003678	2.80	9.07	-1.69	5.00E-05	4.28E-04	--	FAD binding domain
EVM0006402	133.80	432.17	-1.69	5.00E-05	4.28E-04	--	--

EVM0004502	14.05	45.37	-1.69	5.00E-05	4.28E-04	RNA processing and modification	SUZ domain
EVM0003465	15.24	49.19	-1.69	5.00E-05	4.28E-04	--	--
EVM0011270	10.79	34.83	-1.69	5.00E-05	4.28E-04	--	cAMP phosphodiesterases class-II
EVM0008562	182.17	586.17	-1.69	5.00E-05	4.28E-04	--	--
EVM0008828	5.15	16.56	-1.68	5.00E-05	4.28E-04	--	--
EVM0001442	1.06	3.42	-1.68	6.50E-04	3.98E-03	--	Phosphotransferase enzyme family
EVM0006753	4.27	13.69	-1.68	5.00E-05	4.28E-04	General function prediction only	Family of unknown function (DUF706)
EVM0010219	10.43	33.39	-1.68	1.85E-03	9.52E-03	--	--
EVM0006432	2.37	7.56	-1.67	5.00E-05	4.28E-04	--	Fatty acid desaturase
EVM0004134	52.49	167.16	-1.67	5.00E-05	4.28E-04	General function prediction only	RNA recognition motif. (a.k.a. RRM, RBD, or RNP domain)
EVM0005121	147.61	469.78	-1.67	5.00E-05	4.28E-04	General function prediction only	Ras family
EVM0005503	0.99	3.15	-1.67	9.50E-04	5.46E-03	General function prediction only	Major Facilitator Superfamily
EVM0009374	16.62	52.75	-1.67	5.00E-05	4.28E-04	Carbohydrate transport and metabolism	Major Facilitator Superfamily
EVM0000209	7.71	24.46	-1.67	5.00E-05	4.28E-04	Translation, ribosomal structure and biogenesis	KRI1-like family C-terminal
EVM0005190	14.88	47.20	-1.67	5.00E-05	4.28E-04	--	--
EVM0006648	3.63	11.48	-1.66	5.00E-05	4.28E-04	--	--

EVM0004201	81.26	257.07	-1.66	5.00E-05	4.28E-04	--	Glutathione-dependent formaldehyde-activating enzyme
EVM0008825	18.95	59.88	-1.66	2.50E-04	1.77E-03	--	--
EVM0009339	46.72	147.61	-1.66	5.00E-05	4.28E-04	Lipid transport and metabolism	Emopamil binding protein
EVM0001525	32.01	100.94	-1.66	5.00E-05	4.28E-04	--	--
EVM0011057	0.45	1.43	-1.66	1.37E-02	4.81E-02	Signal transduction mechanisms	Protein kinase domain
EVM0009442	25.57	80.48	-1.65	5.00E-05	4.28E-04	--	--
EVM0006390	47.58	149.68	-1.65	5.00E-05	4.28E-04	Inorganic ion transport and metabolism	Heme oxygenase
EVM0008343	11.12	34.89	-1.65	5.00E-05	4.28E-04	--	--
EVM0009885	4.27	13.38	-1.65	2.00E-04	1.46E-03	--	--
EVM0007566	1914.58	5997.38	-1.65	5.00E-05	4.28E-04	--	--
EVM0007465	1.81	5.66	-1.65	2.15E-03	1.08E-02	--	Glycosyl hydrolase family 61
EVM0012095	7.11	22.21	-1.64	5.00E-05	4.28E-04	#N/A	#N/A
EVM0009292	4.05	12.64	-1.64	5.00E-05	4.28E-04	--	--
EVM0009340	39.90	124.43	-1.64	5.00E-05	4.28E-04	--	--
EVM0007132	0.86	2.66	-1.64	1.00E-03	5.70E-03	--	--
EVM0005650	12.37	38.49	-1.64	5.00E-05	4.28E-04	--	--
EVM0008305	10.53	32.76	-1.64	5.00E-05	4.28E-04	General function prediction only	U3 small nucleolar RNA-associated protein 6
EVM0009204	10.20	31.67	-1.63	5.00E-05	4.28E-04	RNA processing and modification	--
EVM0007974	27.03	83.86	-1.63	5.00E-05	4.28E-04	--	--
EVM0006020	33.57	103.92	-1.63	5.00E-05	4.28E-04	--	--

EVM0007872	34.34	106.21	-1.63	5.00E-05	4.28E-04	--	--
EVM0008217	10.18	31.47	-1.63	5.00E-05	4.28E-04	--	Acetyltransferase (GNAT) family
EVM0006662	26.41	81.59	-1.63	5.00E-05	4.28E-04	Lipid transport and metabolism	Hydroxymethylglutaryl-coenzyme A reductase
EVM0011409	8.52	26.32	-1.63	5.00E-05	4.28E-04	--	Ergosterol biosynthesis ERG4/ERG24 family
EVM0009944	7.36	22.75	-1.63	5.00E-05	4.28E-04	--	--
EVM0001647	62.00	191.21	-1.62	5.00E-05	4.28E-04	General function prediction only	Zinc-finger double-stranded RNA-binding
EVM0001573	1.30	4.00	-1.62	1.40E-03	7.55E-03	--	--
EVM0000606	3.15	9.70	-1.62	5.00E-05	4.28E-04	--	Protein of unknown function (DUF1212)
EVM0002154	0.68	2.08	-1.62	4.45E-03	1.96E-02	--	--
EVM0001738	1379.82	4240.67	-1.62	1.30E-03	7.10E-03	Lipid transport and metabolism	Phophatidylserine decarboxylase
EVM0011573	7.88	24.20	-1.62	5.00E-05	4.28E-04	Function unknown	Transcriptional repressor TCF25
EVM0010113	33.98	104.28	-1.62	5.00E-05	4.28E-04	--	--
EVM0002475	28.10	86.22	-1.62	5.00E-05	4.28E-04	--	--
EVM0011042	159.57	487.46	-1.61	5.00E-05	4.28E-04	Signal transduction mechanisms	Inhibitor of apoptosis-promoting Bax1
EVM0010669	117.55	359.05	-1.61	5.00E-05	4.28E-04	--	--
EVM0008633	28.94	88.35	-1.61	5.00E-05	4.28E-04	Intracellular trafficking, secretion, and vesicular transport	Autophagy-related protein 13
EVM0011887	15.54	47.44	-1.61	5.00E-05	4.28E-04	--	--
EVM0002361	15.60	47.58	-1.61	5.00E-05	4.28E-04	Transcription	GATA zinc finger

EVM0005785	1.75	5.33	-1.61	6.50E-04	3.98E-03	--	Variant SH3 domain
EVM0011416	138.19	419.61	-1.60	5.00E-05	4.28E-04	Cell cycle control, cell division, chromosome partitioning	Cdc37 N terminal kinase binding
EVM0012052	183.20	556.16	-1.60	5.00E-05	4.28E-04	--	Cerato-platanin
EVM0004971	12.96	39.33	-1.60	5.00E-05	4.28E-04	#N/A	#N/A
EVM0009851	11.76	35.66	-1.60	5.00E-05	4.28E-04	Function unknown	WD domain, G-beta repeat
EVM0009908	413.33	1253.82	-1.60	5.00E-05	4.28E-04	--	--
EVM0004626	15.54	47.05	-1.60	5.00E-05	4.28E-04	--	--
EVM0001928	13.96	42.24	-1.60	5.00E-05	4.28E-04	--	Ricin-type beta-trefoil lectin domain
EVM0002558	11.74	35.51	-1.60	5.00E-05	4.28E-04	Cell cycle control, cell division, chromosome partitioning	Protein kinase domain
EVM0002672	38.45	116.29	-1.60	5.00E-05	4.28E-04	--	--
EVM0003410	33.43	101.06	-1.60	5.00E-05	4.28E-04	--	--
EVM0005464	15.28	46.17	-1.60	5.00E-05	4.28E-04	Amino acid transport and metabolism	Gamma-glutamyltranspeptidase
EVM0009733	8.69	26.23	-1.59	5.00E-05	4.28E-04	--	--
EVM0006281	14.08	42.47	-1.59	5.00E-05	4.28E-04	Amino acid transport and metabolism	Hydantoinase B/oxoprolinase
EVM0005731	6.11	18.43	-1.59	5.00E-05	4.28E-04	--	--
EVM0004379	196.64	591.88	-1.59	5.00E-05	4.28E-04	Intracellular trafficking, secretion, and vesicular transport	Synaptobrevin
EVM0001403	5.48	16.50	-1.59	5.00E-05	4.28E-04	--	--

EVM0002681	11.19	33.61	-1.59	5.00E-05	4.28E-04	General function prediction only	Glycosyl transferase family 90
EVM0010558	19.86	59.63	-1.59	5.00E-05	4.28E-04	--	--
EVM0007828	14.81	44.43	-1.59	4.70E-03	2.05E-02	Transcription	LAG1, DNA binding
EVM0004866	303.52	910.53	-1.58	5.00E-05	4.28E-04	Function unknown	Uncharacterised protein family (UPF0121)
EVM0011165	39.96	119.75	-1.58	5.00E-05	4.28E-04	Energy production and conversion	Mitochondrial carrier protein
EVM0000390	10.65	31.92	-1.58	5.00E-05	4.28E-04	Amino acid transport and metabolism	Cys/Met metabolism PLP-dependent enzyme
EVM0007307	55.38	165.88	-1.58	5.00E-05	4.28E-04	--	--
EVM0010363	19.22	57.55	-1.58	5.00E-05	4.28E-04	--	Alpha/beta hydrolase family
EVM0005095	8.65	25.87	-1.58	2.50E-04	1.77E-03	--	--
EVM0000240	12.62	37.72	-1.58	5.00E-05	4.28E-04	Transcription	MED6 mediator sub complex component
EVM0008951	27.26	81.36	-1.58	5.00E-05	4.28E-04	Intracellular trafficking, secretion, and vesicular transport	Tetratricopeptide repeat
EVM0004301	13.59	40.53	-1.58	5.00E-05	4.28E-04	Secondary metabolites biosynthesis, transport and catabolism	Pyridine nucleotide-disulphide oxidoreductase
EVM0005034	2143.16	6384.31	-1.57	5.50E-04	3.46E-03	--	--
EVM0011555	68.84	205.03	-1.57	5.00E-05	4.28E-04	--	Sel1 repeat
EVM0007953	187.94	559.39	-1.57	5.00E-05	4.28E-04	--	--

EVM0008702	188.20	560.05	-1.57	5.00E-05	4.28E-04	Intracellular trafficking, secretion, and vesicular transport	PRELI-like family
EVM0004101	41.49	123.30	-1.57	5.00E-05	4.28E-04	Carbohydrate transport and metabolism	Phosphomannose isomerase type I
EVM0009791	8.42	25.01	-1.57	5.00E-05	4.28E-04	Transcription	RNA polymerase Rpb1, domain 5
EVM0008394	31.54	93.64	-1.57	5.00E-05	4.28E-04	General function prediction only	Ankyrin repeats (3 copies)
EVM0006314	2.66	7.89	-1.57	5.00E-05	4.28E-04	--	--
EVM0002980	3.04	9.00	-1.57	5.00E-05	4.28E-04	Amino acid transport and metabolism	Amino acid kinase family
EVM0001816	11.08	32.78	-1.57	1.00E-04	7.96E-04	Posttranslational modification, protein turnover, chaperones	Cyclophilin type peptidyl-prolyl cis-trans isomerase/CLD
EVM0009341	9.53	28.18	-1.56	5.00E-05	4.28E-04	RNA processing and modification	DEAD/DEAH box helicase
EVM0011255	1.87	5.51	-1.56	5.00E-05	4.28E-04	--	--
EVM0005703	18.56	54.75	-1.56	5.00E-05	4.28E-04	Function unknown	Utp14 protein
EVM0006521	6.15	18.14	-1.56	5.00E-05	4.28E-04	--	--
EVM0009038	13.95	41.12	-1.56	5.00E-05	4.28E-04	--	--
EVM0007596	20.18	59.46	-1.56	5.00E-05	4.28E-04	General function prediction only	RNA recognition motif. (a.k.a. RRM, RBD, or RNP domain)
EVM0003493	77.43	228.09	-1.56	5.00E-05	4.28E-04	Cytoskeleton	Calponin homology (CH) domain
EVM0007410	2.72	8.02	-1.56	5.00E-05	4.28E-04	#N/A	#N/A

EVM0007323	699.26	2059.06	-1.56	5.00E-05	4.28E-04	Function unknown	Domain of unknown function (DUF543)
EVM0007568	178.71	526.23	-1.56	5.00E-05	4.28E-04	--	ACT domain
EVM0009077	46.33	136.40	-1.56	5.00E-05	4.28E-04	--	--
EVM0008327	6.79	19.98	-1.56	5.00E-05	4.28E-04	--	--
EVM0001103	3.54	10.43	-1.56	2.00E-04	1.46E-03	--	Glycosyl hydrolases family 16
EVM0003129	3.16	9.28	-1.55	5.00E-05	4.28E-04	--	--
EVM0001002	30.47	89.32	-1.55	5.00E-05	4.28E-04	--	Heterokaryon incompatibility protein Het-C
EVM0010061	78.77	230.53	-1.55	5.00E-05	4.28E-04	--	SH3 domain
EVM0003364	2.87	8.38	-1.55	6.50E-04	3.98E-03	--	--
EVM0004809	43.74	127.43	-1.54	5.00E-05	4.28E-04	Posttranslational modification, protein turnover, chaperones	Myb-like DNA-binding domain
EVM0002948	39.63	115.44	-1.54	5.00E-05	4.28E-04	--	Protein of unknown function (Ytp1)
EVM0003992	15.22	44.33	-1.54	5.00E-05	4.28E-04	--	--
EVM0011145	34.20	99.56	-1.54	5.00E-05	4.28E-04	--	WD domain, G-beta repeat
EVM0008198	40.47	117.60	-1.54	5.00E-05	4.28E-04	Transcription	Transcription initiation factor IIF, beta subunit
EVM0003896	74.37	215.96	-1.54	5.00E-05	4.28E-04	--	Protein of unknown function (DUF2420)
EVM0006099	83.14	241.37	-1.54	5.00E-05	4.28E-04	Lipid transport and metabolism	Enoyl-CoA hydratase/isomerase family
EVM0004056	12.01	34.85	-1.54	1.00E-04	7.96E-04	--	--

EVM0005581	4.93	14.30	-1.54	2.00E-04	1.46E-03	--	Lysine-specific metallo-endopeptidase
EVM0001778	124.96	362.45	-1.54	5.00E-05	4.28E-04	Coenzyme transport and metabolism	Pyridoxamine 5'-phosphate oxidase
EVM0010141	72.42	209.87	-1.54	5.00E-05	4.28E-04	General function prediction only	Prp19/Pso4-like
EVM0012060	4.21	12.19	-1.53	5.00E-05	4.28E-04	--	--
EVM0012112	4.01	11.59	-1.53	7.50E-04	4.49E-03	General function prediction only	--
EVM0007006	80.38	232.63	-1.53	5.00E-05	4.28E-04	Signal transduction mechanisms	CS domain
EVM0009732	14.88	42.98	-1.53	5.00E-05	4.28E-04	--	--
EVM0000380	6.72	19.41	-1.53	5.00E-05	4.28E-04	--	--
EVM0012018	28.35	81.64	-1.53	5.00E-05	4.28E-04	--	--
EVM0004582	1.95	5.63	-1.53	3.80E-03	1.72E-02	--	--
EVM0006433	1.30	3.73	-1.52	1.95E-03	9.93E-03	Carbohydrate transport and metabolism	Major intrinsic protein
EVM0006148	23.28	66.96	-1.52	5.00E-05	4.28E-04	--	--
EVM0006114	36.94	106.15	-1.52	5.00E-05	4.28E-04	--	Thiamine pyrophosphate enzyme, N-terminal TPP binding domain
EVM0005099	0.80	2.29	-1.52	1.08E-02	3.97E-02	Lipid transport and metabolism	Lipase (class 3)
EVM0001593	4.66	13.38	-1.52	5.00E-05	4.28E-04	--	Survival protein SurE
EVM0005950	34.57	99.21	-1.52	5.00E-05	4.28E-04	Carbohydrate transport and metabolism	Major Facilitator Superfamily
EVM0009009	9.55	27.40	-1.52	5.00E-05	4.28E-04	--	--

EVM0002432	3.83	10.99	-1.52	1.20E-03	6.64E-03	--	Cytochrome P450
EVM0003276	0.58	1.67	-1.52	6.65E-03	2.70E-02	General function prediction only	Major Facilitator Superfamily
EVM0005861	157.11	450.22	-1.52	1.50E-04	1.14E-03	Inorganic ion transport and metabolism	Catalase
EVM0003008	72.60	208.02	-1.52	5.00E-05	4.28E-04	--	--
EVM0011391	22.54	64.51	-1.52	5.00E-05	4.28E-04	Translation, ribosomal structure and biogenesis	Noc2p family
EVM0006933	810.11	2318.58	-1.52	1.00E-04	7.96E-04	Nucleotide transport and metabolism	Nucleoside diphosphate kinase
EVM0004316	10.03	28.72	-1.52	8.50E-04	4.98E-03	--	--
EVM0012070	1404.56	4015.74	-1.52	5.00E-04	3.19E-03	--	RNA recognition motif. (a.k.a. RRM, RBD, or RNP domain)
EVM0000093	4.22	12.05	-1.51	1.00E-04	7.96E-04	General function prediction only	HEAT repeats
EVM0001857	3.60	10.28	-1.51	5.00E-05	4.28E-04	--	--
EVM0009103	7.65	21.83	-1.51	5.00E-05	4.28E-04	--	Pheromone A receptor
EVM0002743	16.42	46.85	-1.51	5.00E-05	4.28E-04	--	--
EVM0001320	23.40	66.77	-1.51	5.00E-05	4.28E-04	--	--
EVM0000567	4.16	11.85	-1.51	5.00E-05	4.28E-04	--	Peptidase family M28
EVM0002099	7.65	21.78	-1.51	5.00E-05	4.28E-04	--	--
EVM0004522	16.18	46.08	-1.51	5.00E-05	4.28E-04	--	--
EVM0006606	67.01	190.72	-1.51	5.00E-05	4.28E-04	--	--
EVM0001700	54.42	154.68	-1.51	5.00E-05	4.28E-04	--	Cobalamin-independent synthase, Catalytic domain

EVM0000890	200.00	568.19	-1.51	5.00E-05	4.28E-04	Translation, ribosomal structure and biogenesis	PUA domain
EVM0011691	9.28	26.36	-1.51	5.00E-05	4.28E-04	Function unknown	Regulator of chromosome condensation (RCC1) repeat
EVM0004577	27.55	78.20	-1.51	5.00E-05	4.28E-04	Cell cycle control, cell division, chromosome partitioning	Cid1 family poly A polymerase
EVM0002694	4.83	13.70	-1.50	5.00E-05	4.28E-04	--	DNA polymerase subunit Cdc27
EVM0008173	1.14	3.25	-1.50	4.00E-04	2.65E-03	--	--
EVM0004217	18.35	52.01	-1.50	5.00E-05	4.28E-04	Replication, recombination and repair	--
EVM0010700	13.62	38.60	-1.50	5.00E-05	4.28E-04	--	Glycosyl hydrolase family 76
EVM0002328	19.25	54.51	-1.50	5.00E-05	4.28E-04	--	--
EVM0002245	29.50	83.48	-1.50	5.00E-05	4.28E-04	Function unknown	Leo1-like protein
EVM0009029	77.41	218.90	-1.50	5.00E-05	4.28E-04	Function unknown	Protein of unknown function (DUF1014)
EVM0004698	81.53	230.51	-1.50	5.00E-05	4.28E-04	General function prediction only	Hypoxia induced protein conserved region
EVM0005079	66.24	187.21	-1.50	5.00E-05	4.28E-04	Coenzyme transport and metabolism	Pantoate-beta-alanine ligase
EVM0001171	26.17	73.92	-1.50	5.00E-05	4.28E-04	Signal transduction mechanisms	ERG2 and Sigma1 receptor like protein
EVM0009819	25.17	71.06	-1.50	5.00E-05	4.28E-04	--	CBS domain
EVM0005515	2.40	6.77	-1.49	5.00E-05	4.28E-04	Secondary metabolites biosynthesis, transport and catabolism	Cytochrome P450

EVM0007645	7.01	19.73	-1.49	5.00E-05	4.28E-04	--	Uncharacterised protein family (UPF0014)
EVM0003384	4.06	11.43	-1.49	4.50E-04	2.92E-03	--	Glycosyl hydrolase family 61
EVM0001583	35.72	100.57	-1.49	5.00E-05	4.28E-04	Intracellular trafficking, secretion, and vesicular transport	Ras family
EVM0005154	53.18	149.70	-1.49	5.00E-05	4.28E-04	--	--
EVM0009955	11.05	31.05	-1.49	5.00E-05	4.28E-04	--	--
EVM0000204	803.35	2257.79	-1.49	3.50E-04	2.36E-03	--	short chain dehydrogenase
EVM0008111	1.53	4.30	-1.49	2.00E-03	1.01E-02	--	--
EVM0003431	1.97	5.55	-1.49	6.00E-04	3.72E-03	--	--
EVM0006697	16.67	46.81	-1.49	5.00E-05	4.28E-04	--	--
EVM0011239	91.93	257.86	-1.49	5.00E-05	4.28E-04	--	50S ribosome-binding GTPase
EVM0009523	56.63	158.70	-1.49	5.00E-05	4.28E-04	--	--
EVM0011181	3.76	10.52	-1.48	5.00E-05	4.28E-04	General function prediction only	GMC oxidoreductase
EVM0002044	53.36	149.08	-1.48	5.00E-05	4.28E-04	Translation, ribosomal structure and biogenesis	Initiation factor 2 subunit family
EVM0006994	1.31	3.67	-1.48	1.65E-03	8.65E-03	--	--
EVM0005298	19.56	54.63	-1.48	5.00E-05	4.28E-04	--	--
EVM0000794	677.62	1891.66	-1.48	5.00E-05	4.28E-04	Posttranslational modification, protein turnover, chaperones	Redoxin
EVM0007801	3.63	10.13	-1.48	5.00E-05	4.28E-04	--	--
EVM0012007	20.42	56.96	-1.48	5.00E-05	4.28E-04	--	--
EVM0005710	36.40	101.49	-1.48	5.00E-05	4.28E-04	--	--

EVM0001651	6.84	19.02	-1.48	5.00E-05	4.28E-04	Defense mechanisms	alpha/beta hydrolase fold
EVM0005822	3.17	8.82	-1.48	5.00E-05	4.28E-04	--	Phage lysozyme
EVM0002542	145.03	403.02	-1.47	5.00E-05	4.28E-04	Lipid transport and metabolism	AMP-binding enzyme
EVM0006257	162.43	451.02	-1.47	5.00E-05	4.28E-04	--	--
EVM0005726	7.67	21.28	-1.47	5.00E-05	4.28E-04	--	--
EVM0011587	121.39	336.73	-1.47	5.00E-05	4.28E-04	--	--
EVM0009698	1577.37	4373.42	-1.47	5.00E-05	4.28E-04	Translation, ribosomal structure and biogenesis	Ribosomal L38e protein family
EVM0009206	16.19	44.90	-1.47	5.00E-05	4.28E-04	--	Protein of unknown function (DUF3129)
EVM0004618	3.94	10.91	-1.47	5.00E-05	4.28E-04	Carbohydrate transport and metabolism	Glycosyl hydrolases family 31
EVM0008519	150.41	416.66	-1.47	5.00E-05	4.28E-04	RNA processing and modification	LUC7 N_terminus
EVM0005177	109.00	301.85	-1.47	5.00E-05	4.28E-04	Lipid transport and metabolism	--
EVM0000639	6.21	17.18	-1.47	1.00E-04	7.96E-04	--	--
EVM0011608	1106.38	3057.75	-1.47	5.00E-05	4.28E-04	Translation, ribosomal structure and biogenesis	Ribosomal protein L24e
EVM0010318	5.40	14.91	-1.47	1.50E-04	1.14E-03	--	--
EVM0000905	32.00	88.32	-1.46	5.00E-05	4.28E-04	--	--
EVM0001782	50.75	139.93	-1.46	5.00E-05	4.28E-04	--	Lipase (class 3)
EVM0004771	26.06	71.83	-1.46	5.00E-05	4.28E-04	--	--
EVM0008759	9.36	25.79	-1.46	6.85E-03	2.77E-02	--	--
EVM0000932	98.37	271.11	-1.46	5.00E-05	4.28E-04	--	--

EVM0012234	123.05	338.95	-1.46	5.00E-05	4.28E-04	RNA processing and modification	TAP C-terminal domain
EVM0011531	21.46	59.11	-1.46	5.00E-05	4.28E-04	--	--
EVM0006451	2.25	6.19	-1.46	4.00E-03	1.79E-02	Posttranslational modification, protein turnover, chaperones	Isoprenylcysteine carboxyl methyltransferase (ICMT) family
EVM0011056	57.40	157.83	-1.46	5.00E-05	4.28E-04	--	--
EVM0003863	2.54	6.98	-1.46	3.00E-04	2.07E-03	--	Trichodiene synthase (TRI5)
EVM0002551	20.53	56.42	-1.46	5.00E-05	4.28E-04	Translation, ribosomal structure and biogenesis	Nop14-like family
EVM0008637	12.47	34.25	-1.46	5.00E-05	4.28E-04	RNA processing and modification	WD domain, G-beta repeat
EVM0002778	6.66	18.29	-1.46	1.50E-04	1.14E-03	Chromatin structure and dynamics	Paired amphipathic helix repeat
EVM0010774	1585.17	4344.57	-1.45	5.00E-05	4.28E-04	Posttranslational modification, protein turnover, chaperones	Cyclophilin type peptidyl-prolyl cis-trans isomerase/CLD
EVM0003335	29.73	81.43	-1.45	5.00E-05	4.28E-04	--	Eukaryotic protein of unknown function (DUF1764)
EVM0011601	146.90	401.96	-1.45	5.00E-05	4.28E-04	--	--
EVM0002657	350.18	956.58	-1.45	1.00E-04	7.96E-04	General function prediction only	short chain dehydrogenase
EVM0010039	12.66	34.58	-1.45	5.00E-05	4.28E-04	--	Fungal specific transcription factor domain
EVM0006316	5.93	16.19	-1.45	5.00E-05	4.28E-04	Function unknown	WW domain binding protein 11

EVM0010969	2.11	5.75	-1.45	7.50E-04	4.49E-03	General function prediction only	Zinc-binding dehydrogenase
EVM0007196	310.01	845.81	-1.45	5.00E-05	4.28E-04	RNA processing and modification	LSM domain
EVM0008803	28.64	78.11	-1.45	5.00E-05	4.28E-04	--	FAD binding domain
EVM0001335	9.27	25.29	-1.45	5.00E-05	4.28E-04	RNA processing and modification	Ribosomal L28e protein family
EVM0011446	2630.22	7170.90	-1.45	5.00E-05	4.28E-04	--	--
EVM0012150	31.60	86.14	-1.45	5.00E-05	4.28E-04	--	--
EVM0010983	5.20	14.17	-1.45	5.00E-05	4.28E-04	--	--
EVM0003143	87.67	238.79	-1.45	5.00E-05	4.28E-04	--	Senescence-associated protein
EVM0004038	1552.61	4225.45	-1.44	8.00E-04	4.73E-03	Translation, ribosomal structure and biogenesis	Ribosomal protein L6
EVM0007627	1342.81	3654.10	-1.44	5.00E-05	4.28E-04	Posttranslational modification, protein turnover, chaperones	Hsp20/alpha crystallin family
EVM0006414	20.45	55.64	-1.44	5.00E-05	4.28E-04	General function prediction only	Homeobox domain
EVM0005072	388.60	1056.92	-1.44	5.00E-05	4.28E-04	--	DAHP synthetase I family
EVM0006198	28.01	76.11	-1.44	5.00E-05	4.28E-04	General function prediction only	Protein kinase domain
EVM0002940	127.22	345.69	-1.44	5.00E-05	4.28E-04	General function prediction only	Zinc finger, C2H2 type
EVM0001369	9.46	25.69	-1.44	3.40E-03	1.57E-02	--	--
EVM0000435	51.56	139.96	-1.44	5.00E-05	4.28E-04	General function prediction only	Glycosyl transferase family 2

EVM0001076	8.68	23.56	-1.44	5.00E-05	4.28E-04	--	--
EVM0012187	8.45	22.91	-1.44	5.00E-05	4.28E-04	--	Fungal cellulose binding domain
EVM0009692	29.71	80.48	-1.44	5.00E-05	4.28E-04	General function prediction only	Aldo/keto reductase family
EVM0004846	12.93	35.02	-1.44	5.00E-05	4.28E-04	Posttranslational modification, protein turnover, chaperones	Zinc knuckle
EVM0003386	4.47	12.11	-1.44	1.50E-04	1.14E-03	--	bZIP transcription factor
EVM0007418	54.66	147.90	-1.44	5.00E-05	4.28E-04	Carbohydrate transport and metabolism	Protein kinase domain
EVM0003296	214.70	580.61	-1.44	5.50E-04	3.46E-03	General function prediction only	Domain of unknown function DUF221
EVM0004085	201.70	545.36	-1.43	3.50E-04	2.36E-03	Posttranslational modification, protein turnover, chaperones	FKBP-type peptidyl-prolyl cis-trans isomerase
EVM0008292	16.07	43.43	-1.43	5.00E-05	4.28E-04	Posttranslational modification, protein turnover, chaperones	IBR domain
EVM0003663	1.33	3.58	-1.43	7.50E-04	4.49E-03	Carbohydrate transport and metabolism	O-Glycosyl hydrolase family 30
EVM0009894	40.64	109.70	-1.43	5.00E-05	4.28E-04	--	Fungal specific transcription factor domain
EVM0005821	6.21	16.77	-1.43	5.00E-05	4.28E-04	--	--
EVM0007729	2.53	6.83	-1.43	4.50E-04	2.92E-03	--	--
EVM0003820	20.35	54.91	-1.43	5.00E-05	4.28E-04	--	--
EVM0003368	3.74	10.07	-1.43	1.80E-03	9.29E-03	--	--

EVM0006624	19.39	52.21	-1.43	5.00E-05	4.28E-04	Amino acid transport and metabolism	Pyridoxal-phosphate dependent enzyme
EVM0010878	6.84	18.41	-1.43	5.00E-05	4.28E-04	--	--
EVM0005649	178.28	479.93	-1.43	5.00E-05	4.28E-04	Transcription	Basic region leucine zipper
EVM0004256	6.23	16.77	-1.43	4.20E-03	1.87E-02	--	--
EVM0003346	88.20	236.93	-1.43	1.00E-04	7.96E-04	--	Nitronate monooxygenase
EVM0011568	401.96	1079.51	-1.43	5.00E-05	4.28E-04	--	--
EVM0008872	1.78	4.78	-1.43	6.05E-03	2.51E-02	Lipid transport and metabolism	Fatty acid hydroxylase superfamily
EVM0004977	3.75	10.07	-1.42	8.50E-04	4.98E-03	--	Alpha/beta hydrolase family
EVM0005318	34.68	92.99	-1.42	5.00E-05	4.28E-04	RNA processing and modification	Met-10+ like-protein
EVM0007377	10.04	26.93	-1.42	5.00E-05	4.28E-04	RNA processing and modification	RNA pseudouridylate synthase
EVM0011621	51.54	138.09	-1.42	5.00E-05	4.28E-04	Function unknown	Low temperature viability protein
EVM0004345	18.26	48.94	-1.42	5.00E-05	4.28E-04	--	Taurine catabolism dioxygenase TauD, TfdA family
EVM0006041	230.90	618.32	-1.42	5.00E-05	4.28E-04	Intracellular trafficking, secretion, and vesicular transport	SNARE domain
EVM0004179	4.69	12.55	-1.42	7.50E-04	4.49E-03	Lipid transport and metabolism	short chain dehydrogenase
EVM0002217	160.56	429.31	-1.42	5.00E-05	4.28E-04	--	--
EVM0008316	1.50	4.02	-1.42	3.00E-04	2.07E-03	--	--

EVM0008946	64.11	171.21	-1.42	5.00E-05	4.28E-04	Posttranslational modification, protein turnover, chaperones	Ring finger domain
EVM0003745	4.61	12.31	-1.42	5.00E-05	4.28E-04	--	--
EVM0005073	11.11	29.62	-1.42	5.00E-05	4.28E-04	--	Nitronate monooxygenase
EVM0006998	198.46	529.05	-1.41	5.00E-05	4.28E-04	Carbohydrate transport and metabolism	Aldose 1-epimerase
EVM0003882	60.24	160.58	-1.41	5.00E-05	4.28E-04	Energy production and conversion	Pyridine nucleotide-disulphide oxidoreductase
EVM0009498	1.55	4.12	-1.41	1.42E-02	4.94E-02	General function prediction only	Zinc-finger double domain
EVM0008163	4.77	12.71	-1.41	3.50E-04	2.36E-03	--	--
EVM0005090	7.83	20.83	-1.41	3.00E-04	2.07E-03	--	--
EVM0012061	28.87	76.80	-1.41	5.00E-05	4.28E-04	RNA processing and modification	PIN domain
EVM0008373	13.56	36.05	-1.41	5.00E-05	4.28E-04	General function prediction only	YL1 nuclear protein
EVM0003630	37.70	100.15	-1.41	5.00E-05	4.28E-04	General function prediction only	NGP1NT (NUC091) domain
EVM0000768	749.93	1991.57	-1.41	5.00E-05	4.28E-04	--	--
EVM0001057	25.75	68.32	-1.41	5.00E-05	4.28E-04	Translation, ribosomal structure and biogenesis	tRNA pseudouridine synthase
EVM0007602	11.40	30.24	-1.41	5.00E-05	4.28E-04	Lipid transport and metabolism	PLD-like domain
EVM0009531	2.87	7.60	-1.41	5.00E-05	4.28E-04	--	Polysaccharide lyase family 8, super-sandwich domain

EVM0010169	4.60	12.19	-1.41	5.00E-05	4.28E-04	--	--
EVM0001494	11.14	29.53	-1.41	5.00E-05	4.28E-04	--	Domain of unknown function (DUF4451)
EVM0009378	28.25	74.89	-1.41	5.00E-05	4.28E-04	Signal transduction mechanisms	Pleckstrin homology domain
EVM0000037	1.75	4.63	-1.40	3.70E-03	1.68E-02	Defense mechanisms	alpha/beta hydrolase fold
EVM0009748	22.57	59.69	-1.40	5.00E-05	4.28E-04	--	Endoplasmic reticulum-based factor for assembly of V-ATPase
EVM0011268	21.81	57.66	-1.40	5.00E-05	4.28E-04	--	--
EVM0004288	17.22	45.50	-1.40	2.00E-04	1.46E-03	--	OHCU decarboxylase
EVM0005306	44.42	117.29	-1.40	5.00E-05	4.28E-04	Cell wall/membrane/envelope biogenesis	Glycosyl transferases group 1
EVM0003609	366.16	966.62	-1.40	5.00E-05	4.28E-04	--	Cytochrome oxidase c subunit VIb
EVM0003509	47.08	124.26	-1.40	5.00E-05	4.28E-04	--	--
EVM0005039	192.83	508.02	-1.40	5.00E-05	4.28E-04	RNA processing and modification	DEAD/DEAH box helicase
EVM0011833	28.52	75.11	-1.40	5.00E-05	4.28E-04	--	--
EVM0000400	2.70	7.11	-1.40	5.00E-04	3.19E-03	Lipid transport and metabolism	Lipase (class 3)
EVM0008791	14.59	38.42	-1.40	5.00E-05	4.28E-04	Coenzyme transport and metabolism	Dephospho-CoA kinase
EVM0006610	15.44	40.65	-1.40	5.00E-05	4.28E-04	--	--
EVM0009183	19.28	50.76	-1.40	5.00E-05	4.28E-04	--	BTB/POZ domain
EVM0010602	75.13	197.54	-1.39	5.00E-05	4.28E-04	--	--

EVM0000369	0.64	1.67	-1.39	5.55E-03	2.34E-02	General function prediction only	GMC oxidoreductase
EVM0012015	15.29	40.16	-1.39	5.00E-05	4.28E-04	Translation, ribosomal structure and biogenesis	BOP1NT (NUC169) domain
EVM0006997	8.79	23.07	-1.39	5.00E-05	4.28E-04	--	Glycosyl hydrolases family 28
EVM0004321	53.70	140.64	-1.39	5.00E-05	4.28E-04	Inorganic ion transport and metabolism	Ctr copper transporter family
EVM0006115	2.89	7.56	-1.39	7.50E-04	4.49E-03	Carbohydrate transport and metabolism	Glycosyl transferase family 8
EVM0005431	7.25	18.99	-1.39	5.00E-05	4.28E-04	Cell cycle control, cell division, chromosome partitioning	HEC/Ndc80p family
EVM0006749	24.96	65.23	-1.39	5.00E-05	4.28E-04	--	Membrane bound O-acyl transferase family
EVM0005915	24.78	64.70	-1.38	5.00E-05	4.28E-04	--	Lipase (class 3)
EVM0005340	4.96	12.95	-1.38	9.50E-04	5.46E-03	--	Alpha/beta hydrolase family
EVM0007232	19.82	51.70	-1.38	5.00E-05	4.28E-04	Nucleotide transport and metabolism	Cytidine and deoxycytidylate deaminase zinc-binding region
EVM0007826	24.23	63.15	-1.38	5.00E-05	4.28E-04	General function prediction only	RNA recognition motif. (a.k.a. RRM, RBD, or RNP domain)
EVM0000927	201.97	526.19	-1.38	5.00E-05	4.28E-04	Secondary metabolites biosynthesis, transport and catabolism	Pyridine nucleotide-disulphide oxidoreductase
EVM0011808	2.90	7.56	-1.38	2.45E-03	1.20E-02	--	Lysine-specific metallo-endopeptidase
EVM0005924	1.53	3.99	-1.38	7.75E-03	3.06E-02	--	Glycosyl hydrolase family 61

EVM0002713	13.35	34.76	-1.38	5.00E-05	4.28E-04	Chromatin structure and dynamics	Sin3 family co-repressor
EVM0001160	9.94	25.86	-1.38	1.00E-04	7.96E-04	--	--
EVM0004608	18.87	49.09	-1.38	5.00E-05	4.28E-04	--	--
EVM0009867	39.66	103.14	-1.38	5.00E-05	4.28E-04	--	SNF2 family N-terminal domain
EVM0008232	1.55	4.03	-1.38	2.70E-03	1.30E-02	--	Glycosyl hydrolases family 28
EVM0012002	24.88	64.69	-1.38	5.00E-05	4.28E-04	General function prediction only	WD domain, G-beta repeat
EVM0010331	14.48	37.60	-1.38	5.00E-05	4.28E-04	--	--
EVM0001135	9.87	25.62	-1.38	5.00E-05	4.28E-04	RNA processing and modification	DEAD/DEAH box helicase
EVM0010816	5.26	13.66	-1.38	5.00E-05	4.28E-04	--	alpha/beta hydrolase fold
EVM0005506	4.46	11.58	-1.38	3.50E-04	2.36E-03	#N/A	#N/A
EVM0009843	26.14	67.76	-1.37	5.00E-05	4.28E-04	--	Caspase domain
EVM0001419	184.77	478.79	-1.37	5.00E-05	4.28E-04	Signal transduction mechanisms	SGS domain
EVM0008368	222.78	577.18	-1.37	5.00E-05	4.28E-04	Cell cycle control, cell division, chromosome partitioning	Double-stranded DNA-binding domain
EVM0007634	3.05	7.90	-1.37	4.50E-04	2.92E-03	--	--
EVM0008622	99.12	256.65	-1.37	5.00E-05	4.28E-04	--	50S ribosome-binding GTPase
EVM0006301	28.63	74.11	-1.37	5.00E-05	4.28E-04	Carbohydrate transport and metabolism	Amidohydrolase family
EVM0005200	46.03	119.08	-1.37	5.00E-05	4.28E-04	Lipid transport and metabolism	Alpha/beta hydrolase family

EVM0009112	4.96	12.83	-1.37	6.00E-04	3.72E-03	Defense mechanisms	NAD dependent epimerase/dehydratase family
EVM0011107	137.61	355.39	-1.37	5.00E-05	4.28E-04	--	--
EVM0010981	1041.63	2689.99	-1.37	8.00E-04	4.73E-03	Function unknown	Activator of Hsp90 ATPase, N-terminal
EVM0007524	8.54	22.04	-1.37	5.00E-05	4.28E-04	--	Domain of unknown function (DUF4451)
EVM0007343	27.56	71.12	-1.37	5.00E-05	4.28E-04	--	--
EVM0002076	325.41	839.77	-1.37	5.00E-05	4.28E-04	--	CFEM domain
EVM0010051	3.30	8.52	-1.37	5.00E-05	4.28E-04	--	Glycosyl hydrolase family 3 N terminal domain
EVM0010226	332.37	857.56	-1.37	5.00E-05	4.28E-04	Inorganic ion transport and metabolism	Iron/manganese superoxide dismutases, C-terminal domain
EVM0000675	11.46	29.52	-1.37	5.00E-05	4.28E-04	--	Eukaryotic aspartyl protease
EVM0007858	0.63	1.63	-1.37	1.22E-02	4.38E-02	--	--
EVM0008883	33.08	85.23	-1.37	5.00E-05	4.28E-04	--	--
EVM0002597	8.26	21.26	-1.36	5.00E-05	4.28E-04	--	Flavin reductase like domain
EVM0011352	1.71	4.41	-1.36	2.85E-03	1.36E-02	--	--
EVM0004408	17.94	46.08	-1.36	5.00E-05	4.28E-04	--	Protein of unknown function (DUF563)
EVM0012233	1.25	3.21	-1.36	1.08E-02	3.98E-02	--	--
EVM0005619	16.97	43.57	-1.36	5.00E-05	4.28E-04	--	60Kd inner membrane protein
EVM0004607	6.27	16.09	-1.36	1.00E-04	7.96E-04	--	--
EVM0000375	213.86	548.25	-1.36	5.00E-05	4.28E-04	Lipid transport and metabolism	Fatty acid hydroxylase superfamily
EVM0010743	4.11	10.52	-1.36	2.00E-04	1.46E-03	--	F-box-like

EVM0000212	75.57	193.65	-1.36	5.00E-05	4.28E-04	Posttranslational modification, protein turnover, chaperones	Zinc finger, C3HC4 type (RING finger)
EVM0005019	87.65	224.47	-1.36	5.00E-05	4.28E-04	General function prediction only	Protein kinase domain
EVM0005202	0.97	2.48	-1.36	2.80E-03	1.34E-02	--	Glycosyl hydrolase family 61
EVM0005743	20.51	52.48	-1.36	5.00E-05	4.28E-04	--	--
EVM0008600	29.62	75.77	-1.35	5.00E-05	4.28E-04	--	--
EVM0001556	3.57	9.12	-1.35	2.00E-04	1.46E-03	Amino acid transport and metabolism	Aminotransferase class I and II
EVM0006609	1.43	3.64	-1.35	2.95E-03	1.40E-02	--	G-protein alpha subunit
EVM0006578	103.07	263.57	-1.35	5.00E-05	4.28E-04	Intracellular trafficking, secretion, and vesicular transport	Syntaxin
EVM0006443	2.06	5.27	-1.35	1.10E-03	6.17E-03	General function prediction only	RNA recognition motif. (a.k.a. RRM, RBD, or RNP domain)
EVM0010933	2.24	5.71	-1.35	1.75E-03	9.08E-03	--	--
EVM0003824	1.67	4.26	-1.35	7.15E-03	2.86E-02	--	--
EVM0008917	21.73	55.42	-1.35	3.00E-04	2.07E-03	General function prediction only	Fcf1
EVM0004272	364.07	928.57	-1.35	5.00E-05	4.28E-04	--	Translationally controlled tumour protein
EVM0000041	1.37	3.49	-1.35	1.50E-04	1.14E-03	#N/A	#N/A
EVM0009425	1.85	4.72	-1.35	1.55E-03	8.22E-03	--	--
EVM0007011	66.10	168.24	-1.35	5.00E-05	4.28E-04	Function unknown	Family of unknown function (DUF500)

EVM0004433	8.55	21.72	-1.35	5.00E-05	4.28E-04	--	Lactonase, 7-bladed beta-propeller
EVM0011376	0.60	1.53	-1.35	3.75E-03	1.70E-02	--	--
EVM0004027	2105.78	5348.73	-1.34	5.00E-05	4.28E-04	Translation, ribosomal structure and biogenesis	Ribosomal L27e protein family
EVM0006843	2.46	6.25	-1.34	7.00E-04	4.23E-03	--	Glycosyl hydrolases family 6
EVM0009729	25.37	64.36	-1.34	1.00E-04	7.96E-04	Nucleotide transport and metabolism	Phosphoribosyl transferase domain
EVM0008497	577.43	1463.92	-1.34	9.00E-04	5.22E-03	Lipid transport and metabolism	MaoC like domain
EVM0006496	1.95	4.94	-1.34	5.00E-05	4.28E-04	Replication, recombination and repair	DEAD/DEAH box helicase
EVM0009840	14.42	36.54	-1.34	5.00E-05	4.28E-04	--	BUD22
EVM0005275	517.79	1311.61	-1.34	4.80E-03	2.08E-02	Energy production and conversion	FAD binding domain
EVM0008516	2.13	5.41	-1.34	3.75E-03	1.70E-02	--	Glycosyl hydrolase family 45
EVM0008645	67.91	171.91	-1.34	3.55E-03	1.62E-02	--	--
EVM0005236	393.07	994.81	-1.34	5.00E-05	4.28E-04	RNA processing and modification	LSM domain
EVM0007065	120.18	304.16	-1.34	5.00E-05	4.28E-04	--	--
EVM0008504	39.76	100.53	-1.34	5.00E-05	4.28E-04	--	F-box-like
EVM0002142	54.18	136.91	-1.34	5.00E-05	4.28E-04	General function prediction only	Cyclin
EVM0001250	12.47	31.46	-1.34	5.00E-05	4.28E-04	Inorganic ion transport and metabolism	E1-E2 ATPase

EVM0010158	41.47	104.62	-1.34	5.00E-05	4.28E-04	Function unknown	Pre-rRNA-processing protein TSR2
EVM0006028	235.04	592.51	-1.33	5.00E-05	4.28E-04	--	--
EVM0001608	3.84	9.69	-1.33	5.50E-04	3.46E-03	--	Glycosyl hydrolase family 61
EVM0009830	1.11	2.78	-1.33	7.50E-03	2.98E-02	--	--
EVM0004461	16.00	40.31	-1.33	5.00E-05	4.28E-04	--	Putative oxidoreductase C terminal
EVM0008537	8018.90	20193.20	-1.33	4.60E-03	2.01E-02	Posttranslational modification, protein turnover, chaperones	Hsp20/alpha crystallin family
EVM0007375	14.81	37.29	-1.33	5.00E-05	4.28E-04	General function prediction only	HIT zinc finger
EVM0002182	16.77	42.21	-1.33	5.00E-05	4.28E-04	--	RIO1 family
EVM0007672	3.88	9.77	-1.33	5.50E-04	3.46E-03	Defense mechanisms	NAD dependent epimerase/dehydratase family
EVM0005483	22.26	55.89	-1.33	5.00E-05	4.28E-04	Posttranslational modification, protein turnover, chaperones	ATPase family associated with various cellular activities (AAA)
EVM0011888	0.93	2.35	-1.33	1.09E-02	4.01E-02	--	Peroxidase
EVM0003399	2.21	5.55	-1.33	1.90E-03	9.73E-03	--	--
EVM0004689	4.53	11.37	-1.33	5.00E-05	4.28E-04	--	HAUS augmin-like complex subunit 6 N-terminus
EVM0004830	2.82	7.05	-1.33	1.40E-03	7.55E-03	--	YqcI/YcgG family
EVM0003606	18.41	46.12	-1.33	5.00E-05	4.28E-04	--	--
EVM0008340	16.23	40.59	-1.32	1.50E-04	1.14E-03	General function prediction only	Methyltransferase domain

EVM0010339	23.88	59.74	-1.32	5.00E-05	4.28E-04	--	WW domain
EVM0003803	1.80	4.50	-1.32	2.80E-03	1.34E-02	--	NADH:flavin oxidoreductase / NADH oxidase family
EVM0010881	23.69	59.14	-1.32	5.00E-05	4.28E-04	--	Inositol polyphosphate kinase
EVM0001587	13.52	33.73	-1.32	5.00E-05	4.28E-04	--	--
EVM0005891	32.76	81.71	-1.32	5.00E-05	4.28E-04	Energy production and conversion	Pyridine nucleotide-disulphide oxidoreductase
EVM0001521	15.55	38.77	-1.32	1.50E-04	1.14E-03	--	--
EVM0007328	1928.08	4802.84	-1.32	5.00E-05	4.28E-04	Translation, ribosomal structure and biogenesis	60s Acidic ribosomal protein
EVM0008609	19.64	48.92	-1.32	3.35E-03	1.55E-02	General function prediction only	Ankyrin repeats (3 copies)
EVM0006159	40.98	102.02	-1.32	5.00E-05	4.28E-04	--	--
EVM0000986	8.59	21.37	-1.32	5.00E-05	4.28E-04	--	--
EVM0008995	26.65	66.32	-1.32	5.00E-05	4.28E-04	--	Arrestin (or S-antigen), C-terminal domain
EVM0010646	29.60	73.64	-1.31	5.00E-05	4.28E-04	Cell wall/membrane/envelope biogenesis	Mechanosensitive ion channel
EVM0002993	7.18	17.86	-1.31	5.00E-05	4.28E-04	--	Spb1 C-terminal domain
EVM0007514	33.18	82.54	-1.31	5.00E-05	4.28E-04	--	RTA1 like protein
EVM0009405	12.99	32.30	-1.31	5.55E-03	2.34E-02	General function prediction only	Yippee zinc-binding/DNA-binding /Mis18, centromere assembly
EVM0009615	3.93	9.76	-1.31	1.10E-03	6.17E-03	--	Pectate lyase

EVM0002208	25.41	63.02	-1.31	5.00E-05	4.28E-04	Lipid transport and metabolism	CRAL/TRIO domain
EVM0003943	2.54	6.30	-1.31	5.00E-05	4.28E-04	--	--
EVM0007351	6.78	16.81	-1.31	5.00E-05	4.28E-04	--	--
EVM0011602	2270.95	5628.31	-1.31	5.00E-05	4.28E-04	Translation, ribosomal structure and biogenesis	Translation initiation factor SUI1
EVM0008835	35.82	88.70	-1.31	5.00E-05	4.28E-04	Carbohydrate transport and metabolism	--
EVM0011261	25.40	62.85	-1.31	5.00E-05	4.28E-04	General function prediction only	WD domain, G-beta repeat
EVM0011343	60.87	150.59	-1.31	5.00E-05	4.28E-04	Posttranslational modification, protein turnover, chaperones	Glutaredoxin
EVM0010336	59.89	147.96	-1.30	5.00E-05	4.28E-04	--	--
EVM0010845	0.99	2.45	-1.30	1.27E-02	4.52E-02	--	Domain of unknown function (DUF3415)
EVM0011499	10.11	24.96	-1.30	2.00E-04	1.46E-03	--	--
EVM0003922	20.43	50.37	-1.30	5.00E-05	4.28E-04	Function unknown	--
EVM0010985	28.80	70.99	-1.30	1.00E-04	7.96E-04	Carbohydrate transport and metabolism	Transaldolase
EVM0008211	14.80	36.44	-1.30	5.00E-05	4.28E-04	Signal transduction mechanisms	Protein tyrosine kinase
EVM0002753	17.65	43.44	-1.30	5.00E-05	4.28E-04	--	G protein-coupled glucose receptor regulating Gpa2
EVM0011734	5.99	14.73	-1.30	3.50E-04	2.36E-03	--	--
EVM0011492	260.14	639.74	-1.30	5.00E-05	4.28E-04	--	--

EVM0003818	15.18	37.33	-1.30	5.00E-05	4.28E-04	RNA processing and modification	RNA recognition motif. (a.k.a. RRM, RBD, or RNP domain)
EVM0008910	12.39	30.47	-1.30	1.00E-04	7.96E-04	--	--
EVM0011552	35.30	86.70	-1.30	5.00E-05	4.28E-04	Lipid transport and metabolism	Fatty acid desaturase
EVM0011840	15.75	38.66	-1.30	5.00E-05	4.28E-04	--	--
EVM0007512	200.47	491.17	-1.29	1.00E-04	7.96E-04	Cell cycle control, cell division, chromosome partitioning	Protein kinase domain
EVM0008044	14.30	35.04	-1.29	5.00E-04	3.19E-03	--	--
EVM0001473	24.18	59.25	-1.29	5.00E-05	4.28E-04	Intracellular trafficking, secretion, and vesicular transport	Transport protein particle (TRAPP) component
EVM0006895	2.87	7.03	-1.29	5.95E-03	2.47E-02	--	Protein of unknown function (DUF3129)
EVM0004556	8.30	20.31	-1.29	5.00E-05	4.28E-04	--	NACHT domain
EVM0001584	35.33	86.44	-1.29	5.00E-05	4.28E-04	--	Voltage-dependent anion channel
EVM0001421	43.49	106.35	-1.29	5.00E-05	4.28E-04	Cell cycle control, cell division, chromosome partitioning	Protein kinase domain
EVM0000264	0.97	2.37	-1.29	2.35E-03	1.16E-02	--	--
EVM0008142	0.61	1.49	-1.29	5.05E-03	2.17E-02	--	--
EVM0000653	48.09	117.54	-1.29	5.00E-05	4.28E-04	Function unknown	Domain of unknown function (DUF947)
EVM0011522	8.83	21.55	-1.29	2.50E-04	1.77E-03	Defense mechanisms	NAD dependent epimerase/dehydratase family

EVM0004623	9.64	23.52	-1.29	5.00E-05	4.28E-04	--	F-box-like
EVM0007930	11.07	26.99	-1.29	5.00E-05	4.28E-04	--	RNA recognition motif (a.k.a. RRM, RBD, or RNP domain)
EVM0005026	4.13	10.06	-1.29	7.00E-04	4.23E-03	--	--
EVM0005245	26.13	63.68	-1.29	5.00E-05	4.28E-04	--	Ubiquitin-2 like Rad60 SUMO-like
EVM0000121	44.85	109.18	-1.28	5.00E-05	4.28E-04	General function prediction only	Haloacid dehalogenase-like hydrolase
EVM0005338	187.89	457.18	-1.28	5.00E-05	4.28E-04	General function prediction only	Zinc-binding dehydrogenase
EVM0006924	17.43	42.33	-1.28	2.50E-04	1.77E-03	--	--
EVM0012121	19.61	47.60	-1.28	5.00E-05	4.28E-04	--	Glioma tumor suppressor candidate region
EVM0009034	12.09	29.34	-1.28	2.00E-04	1.46E-03	--	--
EVM0008481	11.72	28.45	-1.28	5.00E-05	4.28E-04	Inorganic ion transport and metabolism	CorA-like Mg2+ transporter protein
EVM0001809	129.36	313.62	-1.28	5.00E-05	4.28E-04	--	--
EVM0012201	4.88	11.81	-1.28	5.00E-04	3.19E-03	--	--
EVM0001227	39.10	94.70	-1.28	5.00E-05	4.28E-04	Function unknown	Pre-mRNA-splicing factor of RES complex
EVM0003283	17.04	41.24	-1.27	5.00E-05	4.28E-04	--	--
EVM0011843	2.14	5.17	-1.27	9.00E-04	5.22E-03	--	--
EVM0010862	95.69	231.41	-1.27	5.00E-05	4.28E-04	Translation, ribosomal structure and biogenesis	Ribosomal protein L24e
EVM0007013	24.72	59.78	-1.27	5.00E-05	4.28E-04	--	Protein of unknown function (DUF1275)

EVM0009750	114.66	277.19	-1.27	5.00E-05	4.28E-04	Lipid transport and metabolism	PAP2 superfamily
EVM0011085	39.91	96.40	-1.27	5.00E-05	4.28E-04	--	Translation initiation factor IF-3, C-terminal domain
EVM0002721	101.31	244.62	-1.27	5.00E-05	4.28E-04	Carbohydrate transport and metabolism	Glycosyl hydrolases family 18
EVM0004090	16.49	39.80	-1.27	5.00E-05	4.28E-04	--	--
EVM0002495	443.19	1068.61	-1.27	2.00E-04	1.46E-03	Posttranslational modification, protein turnover, chaperones	Cyclophilin type peptidyl-prolyl cis-trans isomerase/CLD
EVM0009839	190.50	458.94	-1.27	3.50E-04	2.36E-03	Carbohydrate transport and metabolism	Glycosyltransferase family 20
EVM0007485	90.33	217.59	-1.27	5.00E-05	4.28E-04	--	Fasciclin domain
EVM0001538	14.28	34.39	-1.27	5.00E-05	4.28E-04	--	F-box-like
EVM0003807	2.35	5.65	-1.27	5.95E-03	2.47E-02	Amino acid transport and metabolism	--
EVM0006468	15.66	37.70	-1.27	1.00E-04	7.96E-04	Signal transduction mechanisms	RED-like protein N-terminal region
EVM0008224	180.18	433.73	-1.27	2.00E-04	1.46E-03	Amino acid transport and metabolism	Protein of unknown function (DUF619)
EVM0003669	1.83	4.41	-1.27	5.00E-03	2.15E-02	--	--
EVM0008927	47.33	113.79	-1.27	5.00E-05	4.28E-04	--	Velvet factor
EVM0003527	58.28	140.07	-1.27	5.00E-05	4.28E-04	Transcription	RNA polymerase Rpb1, domain 5
EVM0007629	63.87	153.46	-1.26	5.00E-05	4.28E-04	General function prediction only	Protein of unknown function (DUF933)

EVM0006166	78.61	188.90	-1.26	5.00E-05	4.28E-04	Nucleotide transport and metabolism	SAICAR synthetase
EVM0005262	5.10	12.26	-1.26	4.50E-03	1.97E-02	--	--
EVM0001821	2.63	6.32	-1.26	9.00E-04	5.22E-03	Transcription	HMG (high mobility group) box
EVM0007133	210.60	505.75	-1.26	5.00E-05	4.28E-04	Function unknown	SNARE associated Golgi protein
EVM0003487	65.97	158.40	-1.26	5.00E-05	4.28E-04	--	Eukaryotic cytochrome b561
EVM0006637	7.81	18.76	-1.26	5.00E-05	4.28E-04	Posttranslational modification, protein turnover, chaperones	Prolyl oligopeptidase family
EVM0004668	200.52	481.45	-1.26	5.00E-05	4.28E-04	General function prediction only	Proteolipid membrane potential modulator
EVM0010500	36.53	87.67	-1.26	5.00E-05	4.28E-04	Transcription	RNA polymerase II transcription factor SIII (Elongin) subunit A
EVM0010843	48.38	116.02	-1.26	5.00E-05	4.28E-04	Coenzyme transport and metabolism	Dihydroneopterin aldolase
EVM0003002	14.42	34.57	-1.26	8.40E-03	3.26E-02	Transcription	Transcription factor S-II (TFIIS)
EVM0006795	1494.96	3582.30	-1.26	6.60E-03	2.69E-02	--	--
EVM0005770	3.66	8.78	-1.26	1.55E-03	8.22E-03	--	--
EVM0004878	173.45	415.14	-1.26	5.00E-05	4.28E-04	--	--
EVM0007137	8.29	19.84	-1.26	4.50E-04	2.92E-03	--	Glycosyl hydrolase family 61
EVM0000784	9.86	23.58	-1.26	2.00E-04	1.46E-03	--	--
EVM0000666	103.37	247.26	-1.26	5.00E-05	4.28E-04	--	--
EVM0006908	21.32	50.97	-1.26	1.00E-04	7.96E-04	--	FAD binding domain
EVM0010038	3.09	7.37	-1.26	1.41E-02	4.91E-02	--	--
EVM0003967	141.19	337.15	-1.26	5.00E-05	4.28E-04	Function unknown	Mago binding
EVM0003061	26.38	62.92	-1.25	5.00E-05	4.28E-04	--	--

EVM0002998	64.45	153.63	-1.25	5.00E-05	4.28E-04	Defense mechanisms	NAD dependent epimerase/dehydratase family
EVM0003340	15.80	37.62	-1.25	5.00E-05	4.28E-04	--	Nuclear fragile X mental retardation-interacting protein 1 (NUFIP1)
EVM0006033	18.27	43.50	-1.25	5.00E-05	4.28E-04	--	--
EVM0009289	10.97	26.11	-1.25	5.00E-05	4.28E-04	--	--
EVM0011738	27.34	65.06	-1.25	5.00E-05	4.28E-04	--	--
EVM0004042	56.20	133.72	-1.25	5.00E-05	4.28E-04	--	--
EVM0009458	2.51	5.98	-1.25	9.00E-04	5.22E-03	--	--
EVM0009693	21.39	50.86	-1.25	5.00E-05	4.28E-04	Function unknown	--
EVM0009858	33.46	79.52	-1.25	5.00E-05	4.28E-04	Translation, ribosomal structure and biogenesis	CPL (NUC119) domain
EVM0010680	11.47	27.25	-1.25	7.00E-04	4.23E-03	General function prediction only	Ras family
EVM0010670	3.37	8.02	-1.25	2.35E-03	1.16E-02	--	--
EVM0008719	11.15	26.49	-1.25	5.00E-05	4.28E-04	--	--
EVM0001019	1150.50	2732.13	-1.25	2.00E-04	1.46E-03	--	--
EVM0005896	33.67	79.95	-1.25	1.00E-04	7.96E-04	--	Alpha/beta hydrolase family
EVM0010800	99.16	235.42	-1.25	1.00E-04	7.96E-04	Carbohydrate transport and metabolism	Formyl transferase
EVM0002482	12.20	28.94	-1.25	2.00E-04	1.46E-03	--	--
EVM0007683	4.06	9.63	-1.25	5.00E-05	4.28E-04	Posttranslational modification, protein turnover, chaperones	--
EVM0007044	37.64	89.15	-1.24	5.00E-05	4.28E-04	--	Salt tolerance down-regulator

EVM0006845	17.01	40.29	-1.24	5.00E-05	4.28E-04	--	SNF5 / SMARCB1 / INI1
EVM0009931	63.75	150.96	-1.24	5.00E-05	4.28E-04	--	--
EVM0006480	8.30	19.65	-1.24	1.00E-04	7.96E-04	--	Membrane transport protein
EVM0009948	4.41	10.45	-1.24	4.00E-04	2.65E-03	--	--
EVM0005584	1.86	4.39	-1.24	5.05E-03	2.17E-02	--	--
EVM0001922	8.14	19.26	-1.24	1.00E-04	7.96E-04	--	Fungal specific transcription factor domain
EVM0010988	28.44	67.27	-1.24	5.00E-05	4.28E-04	Secondary metabolites biosynthesis, transport and catabolism	Alcohol dehydrogenase GroES-like domain
EVM0011177	22.62	53.50	-1.24	5.00E-05	4.28E-04	RNA processing and modification	Helicase associated domain (HA2)
EVM0004008	141.58	334.73	-1.24	9.00E-04	5.22E-03	Lipid transport and metabolism	Choline/Carnitine o-acyltransferase
EVM0004966	2.64	6.24	-1.24	5.00E-05	4.28E-04	--	--
EVM0007135	2.88	6.81	-1.24	2.80E-03	1.34E-02	--	Metallo-beta-lactamase superfamily
EVM0010575	22.57	53.30	-1.24	5.00E-05	4.28E-04	--	--
EVM0009504	9.34	22.06	-1.24	5.00E-05	4.28E-04	--	--
EVM0002104	48.63	114.79	-1.24	5.00E-05	4.28E-04	Signal transduction mechanisms	Protein kinase domain
EVM0003294	4.61	10.87	-1.24	3.00E-04	2.07E-03	--	Pro-kumamolisin, activation domain
EVM0000799	2.55	6.01	-1.24	1.85E-03	9.52E-03	--	Membrane bound O-acyl transferase family

EVM0003839	801.48	1889.09	-1.24	5.00E-05	4.28E-04	Translation, ribosomal structure and biogenesis	Ribosomal S13/S15 N-terminal domain
EVM0005541	33.03	77.83	-1.24	5.00E-05	4.28E-04	--	Aminotransferase class-V
EVM0001684	30.95	72.93	-1.24	4.00E-04	2.65E-03	General function prediction only	AAA domain (dynein-related subfamily)
EVM0003868	24.54	57.79	-1.24	6.50E-04	3.98E-03	--	Peptidase inhibitor I9
EVM0005811	1.96	4.61	-1.24	3.70E-03	1.68E-02	--	--
EVM0011129	11.85	27.90	-1.23	2.50E-04	1.77E-03	--	--
EVM0003373	3.94	9.27	-1.23	3.20E-03	1.49E-02	--	--
EVM0007363	6.89	16.21	-1.23	1.60E-03	8.44E-03	--	--
EVM0000075	5.28	12.41	-1.23	1.00E-04	7.96E-04	--	F-box domain
EVM0007890	192.20	451.96	-1.23	5.00E-05	4.28E-04	--	--
EVM0002580	7.01	16.47	-1.23	1.70E-03	8.87E-03	--	Acetyltransferase (GNAT) family
EVM0006458	13.83	32.50	-1.23	5.00E-05	4.28E-04	--	Cytochrome P450
EVM0010567	7.89	18.53	-1.23	2.00E-04	1.46E-03	--	Putative serine dehydratase domain
EVM0009477	10.32	24.23	-1.23	1.00E-04	7.96E-04	--	F-box-like
EVM0010633	1350.09	3170.56	-1.23	5.00E-05	4.28E-04	Energy production and conversion	--
EVM0010989	57.69	135.41	-1.23	2.00E-04	1.46E-03	--	NADH:flavin oxidoreductase / NADH oxidase family
EVM0003721	254.23	596.65	-1.23	5.00E-05	4.28E-04	--	--
EVM0010517	243.58	571.41	-1.23	5.00E-05	4.28E-04	Posttranslational modification, protein turnover, chaperones	SelR domain
EVM0006126	24.83	58.23	-1.23	5.00E-05	4.28E-04	--	--

EVM0011784	30.44	71.32	-1.23		5.00E-05	4.28E-04	Inorganic ion transport and metabolism	Sodium/hydrogen exchanger family
EVM0011823	7.10	16.63	-1.23		1.00E-04	7.96E-04	Inorganic ion transport and metabolism	Natural resistance-associated macrophage protein
EVM0006717	16.65	39.01	-1.23		3.50E-04	2.36E-03	--	--
EVM0010749	1.31	3.07	-1.23		2.95E-03	1.40E-02	Secondary metabolites biosynthesis, transport and catabolism	Cytochrome P450
EVM0002996	26.17	61.25	-1.23		5.00E-05	4.28E-04	Transcription	Sin3 associated polypeptide p18 (SAP18)
EVM0000851	392.38	918.17	-1.23		1.85E-03	9.52E-03	--	Eisosome component PIL1
EVM0011423	2.94	6.87	-1.23		7.50E-04	4.49E-03	General function prediction only	Sugar (and other) transporter
EVM0011661	12.71	29.70	-1.22		5.00E-05	4.28E-04	Signal transduction mechanisms	Protein kinase domain
EVM0003203	23.85	55.75	-1.22		5.00E-05	4.28E-04	--	--
EVM0002190	20.64	48.21	-1.22		5.00E-05	4.28E-04	--	N-terminal C2 in EEIG1 and EHBP1 proteins
EVM0007585	11.21	26.20	-1.22		1.00E-04	7.96E-04	Amino acid transport and metabolism	Amino acid permease
EVM0002120	3.02	7.06	-1.22		7.50E-04	4.49E-03	Energy production and conversion	FAD binding domain
EVM0006013	1.62	3.77	-1.22		3.10E-03	1.45E-02	--	--
EVM0000483	176.73	412.13	-1.22		1.00E-04	7.96E-04	Amino acid transport and metabolism	Chorismate mutase type II
EVM0011383	78.03	181.97	-1.22		1.00E-04	7.96E-04	Function unknown	Zinc finger, ZZ type

EVM0001620	4.76	11.11	-1.22	1.70E-03	8.87E-03	--	--
EVM0006910	3.59	8.36	-1.22	4.00E-04	2.65E-03	--	--
EVM0008438	16.67	38.81	-1.22	5.00E-05	4.28E-04	--	Apoptosis-antagonizing transcription factor, C-terminal
EVM0000557	23.01	53.55	-1.22	1.00E-04	7.96E-04	--	--
EVM0008439	11.76	27.36	-1.22	2.00E-04	1.46E-03	--	Uncharacterized alpha/beta hydrolase domain (DUF2235)
EVM0004198	145.75	338.52	-1.22	5.00E-05	4.28E-04	--	--
EVM0005216	46.67	108.37	-1.22	5.00E-05	4.28E-04	--	Whi5 like
EVM0010692	23.40	54.34	-1.22	1.00E-04	7.96E-04	--	--
EVM0005171	16.83	39.07	-1.21	5.00E-05	4.28E-04	Replication, recombination and repair	DNA mismatch repair protein, C-terminal domain
EVM0001479	2326.67	5399.71	-1.21	5.00E-05	4.28E-04	Translation, ribosomal structure and biogenesis	Ribosomal protein L18e/L15
EVM0003660	61.80	143.39	-1.21	5.00E-05	4.28E-04	--	--
EVM0012102	17.50	40.60	-1.21	3.50E-04	2.36E-03	--	--
EVM0003224	65.35	151.56	-1.21	5.00E-05	4.28E-04	General function prediction only	Phosphatidylethanolamine-binding protein
EVM0006065	16.59	38.46	-1.21	5.00E-05	4.28E-04	--	--
EVM0006060	5.47	12.67	-1.21	3.00E-04	2.07E-03	--	PP-loop family
EVM0001211	12.21	28.29	-1.21	5.00E-05	4.28E-04	--	--
EVM0009657	1.26	2.92	-1.21	4.90E-03	2.12E-02	Secondary metabolites biosynthesis, transport and catabolism	Cytochrome P450
EVM0001905	79.00	182.92	-1.21	1.00E-04	7.96E-04	General function prediction only	GMC oxidoreductase

EVM0007506	269.85	624.50	-1.21	5.00E-05	4.28E-04	Intracellular trafficking, secretion, and vesicular transport	Regulated-SNARE-like domain
EVM0008847	4.53	10.47	-1.21	2.50E-04	1.77E-03	Posttranslational modification, protein turnover, chaperones	Cyclophilin type peptidyl-prolyl cis-trans isomerase/CLD
EVM0007305	349.97	808.63	-1.21	5.00E-05	4.28E-04	RNA processing and modification	U1 zinc finger
EVM0003531	7.56	17.46	-1.21	5.00E-05	4.28E-04	--	Fungal specific transcription factor domain
EVM0002787	119.17	275.13	-1.21	2.50E-04	1.77E-03	Posttranslational modification, protein turnover, chaperones	Thioredoxin
EVM0001027	63.67	146.99	-1.21	5.00E-05	4.28E-04	Signal transduction mechanisms	HIT domain
EVM0010310	17.67	40.78	-1.21	8.90E-03	3.41E-02	--	RNA recognition motif. (a.k.a. RRM, RBD, or RNP domain)
EVM0009586	48.27	111.26	-1.20	5.00E-05	4.28E-04	Lipid transport and metabolism	Fatty acid hydroxylase superfamily
EVM0004240	9.31	21.45	-1.20	2.30E-03	1.14E-02	#N/A	#N/A
EVM0004868	20.24	46.64	-1.20	5.00E-05	4.28E-04	--	F-box-like
EVM0010071	4.55	10.49	-1.20	4.50E-04	2.92E-03	--	--
EVM0010605	962.90	2217.45	-1.20	5.00E-05	4.28E-04	Translation, ribosomal structure and biogenesis	Ribosomal protein L34e
EVM0001483	20.38	46.92	-1.20	5.00E-05	4.28E-04	Transcription	Transcription factor S-II (TFIIS), central domain

EVM0012042	4.92	11.31	-1.20	1.40E-03	7.55E-03	--	--
EVM0010101	5.21	11.98	-1.20	1.85E-03	9.52E-03	--	--
EVM0005126	2.58	5.93	-1.20	2.10E-03	1.06E-02	--	--
EVM0012211	28.44	65.38	-1.20	1.00E-04	7.96E-04	--	--
EVM0007660	16.37	37.64	-1.20	2.50E-04	1.77E-03	--	Pregnancy-associated plasma protein-A
EVM0004831	16.00	36.77	-1.20	2.00E-04	1.46E-03	Lipid transport and metabolism	CRAL/TRIO domain
EVM0011757	4.27	9.82	-1.20	5.40E-03	2.29E-02	--	--
EVM0006288	22.63	51.99	-1.20	5.00E-05	4.28E-04	--	--
EVM0005282	310.68	713.30	-1.20	1.50E-04	1.14E-03	Posttranslational modification, protein turnover, chaperones	Nucleotide exchange factor Fes1
EVM0005135	8.93	20.49	-1.20	2.00E-04	1.46E-03	Secondary metabolites biosynthesis, transport and catabolism	Cytochrome P450
EVM0010327	16.55	37.95	-1.20	1.35E-03	7.33E-03	--	--
EVM0004661	6.04	13.84	-1.20	3.00E-04	2.07E-03	--	Cytochrome P450
EVM0007121	8.33	19.08	-1.20	5.00E-04	3.19E-03	--	--
EVM0000696	3.76	8.60	-1.20	7.00E-04	4.23E-03	Inorganic ion transport and metabolism	Cation transport protein
EVM0007942	81.46	186.36	-1.19	1.50E-04	1.14E-03	Function unknown	Protein of unknown function DUF89
EVM0000245	51.25	117.18	-1.19	5.00E-05	4.28E-04	Translation, ribosomal structure and biogenesis	--
EVM0004978	4.69	10.73	-1.19	1.11E-02	4.07E-02	--	--

EVM0001611	2.70	6.18	-1.19	1.75E-03	9.08E-03	--	Glycosyl hydrolase family 61
EVM0005518	1.19	2.71	-1.19	1.31E-02	4.63E-02	--	non-haem dioxygenase in morphine synthesis N-terminal
EVM0001437	19.76	45.14	-1.19	5.00E-05	4.28E-04	RNA processing and modification	pre-mRNA processing factor 3 (PRP3)
EVM0002590	22.23	50.76	-1.19	5.00E-05	4.28E-04	Carbohydrate transport and metabolism	Glycosyl hydrolase family 47
EVM0011949	37.42	85.46	-1.19	5.00E-05	4.28E-04	--	Protein of unknown function (DUF3245)
EVM0011546	3.68	8.41	-1.19	5.00E-04	3.19E-03	--	--
EVM0006532	52.38	119.47	-1.19	5.00E-05	4.28E-04	--	--
EVM0003316	490.45	1118.51	-1.19	5.00E-05	4.28E-04	--	--
EVM0005758	66.90	152.55	-1.19	5.00E-05	4.28E-04	Lipid transport and metabolism	Putative undecaprenyl diphosphate synthase
EVM0003024	47.56	108.45	-1.19	1.00E-04	7.96E-04	Signal transduction mechanisms	Rhomboid family
EVM0005467	135.33	308.53	-1.19	5.00E-05	4.28E-04	Energy production and conversion	Mitochondrial carrier protein
EVM0004220	8.05	18.34	-1.19	1.50E-04	1.14E-03	--	--
EVM0005188	134.10	305.72	-1.19	2.50E-04	1.77E-03	Inorganic ion transport and metabolism	Carbonic anhydrase
EVM0007837	2567.46	5847.03	-1.19	8.50E-04	4.98E-03	Translation, ribosomal structure and biogenesis	Ribosomal protein S17
EVM0005692	15.00	34.13	-1.19	3.00E-04	2.07E-03	--	--
EVM0000201	31.61	71.89	-1.19	5.00E-05	4.28E-04	--	Fungal specific transcription factor domain

EVM0000051	1.05	2.38	-1.18	8.05E-03	3.15E-02	--	--
EVM0000030	8.93	20.28	-1.18	2.00E-04	1.46E-03	Inorganic ion transport and metabolism	Sodium/calcium exchanger protein
EVM0010108	16.10	36.54	-1.18	5.00E-05	4.28E-04	Intracellular trafficking, secretion, and vesicular transport	Sec7 domain
EVM0010704	49.46	112.22	-1.18	5.00E-05	4.28E-04	Posttranslational modification, protein turnover, chaperones	--
EVM0006176	29.02	65.84	-1.18	2.50E-04	1.77E-03	--	--
EVM0011758	3.94	8.93	-1.18	2.80E-03	1.34E-02	General function prediction only	THUMP domain
EVM0000596	6.54	14.83	-1.18	7.00E-04	4.23E-03	Replication, recombination and repair	--
EVM0004195	1.69	3.82	-1.18	7.25E-03	2.90E-02	--	--
EVM0005593	21.47	48.65	-1.18	5.00E-05	4.28E-04	General function prediction only	TPR repeat
EVM0003685	7.80	17.68	-1.18	8.00E-04	4.73E-03	--	--
EVM0001189	44.76	101.31	-1.18	5.00E-05	4.28E-04	Chromatin structure and dynamics	Histone deacetylase domain
EVM0011097	378.34	855.96	-1.18	5.00E-05	4.28E-04	--	RNA recognition motif. (a.k.a. RRM, RBD, or RNP domain)
EVM0009094	9.60	21.71	-1.18	3.00E-04	2.07E-03	--	--
EVM0011743	5.63	12.72	-1.18	5.20E-03	2.22E-02	--	--
EVM0001308	1.34	3.02	-1.18	2.60E-03	1.26E-02	General function prediction only	short chain dehydrogenase

EVM0012010	48.69	110.02	-1.18	5.00E-05	4.28E-04	--	--
EVM0007631	21.63	48.81	-1.17	5.00E-05	4.28E-04	--	--
EVM0004782	29.95	67.55	-1.17	1.50E-04	1.14E-03	--	Tyrosine phosphatase family
EVM0004495	299.00	674.21	-1.17	5.00E-05	4.28E-04	Translation, ribosomal structure and biogenesis	Ribosomal protein L7Ae/L30e/S12e/Gadd45 family
EVM0007476	4.94	11.14	-1.17	1.75E-03	9.08E-03	--	Protein of unknown function (DUF1399)
EVM0005046	1.38	3.11	-1.17	8.35E-03	3.24E-02	--	Glycosyl Hydrolase Family 88
EVM0004026	32.57	73.39	-1.17	2.00E-04	1.46E-03	Secondary metabolites biosynthesis, transport and catabolism	short chain dehydrogenase
EVM0008172	31.59	71.18	-1.17	5.00E-05	4.28E-04	--	Alpha/beta hydrolase family
EVM0010867	1471.16	3313.53	-1.17	1.45E-03	7.77E-03	--	--
EVM0002339	62.86	141.57	-1.17	5.00E-05	4.28E-04	--	--
EVM0004503	3.20	7.20	-1.17	2.75E-03	1.32E-02	--	--
EVM0004108	3.02	6.80	-1.17	1.15E-03	6.41E-03	Secondary metabolites biosynthesis, transport and catabolism	Cytochrome P450
EVM0010258	45.83	103.17	-1.17	1.00E-04	7.96E-04	--	--
EVM0007278	81.72	183.92	-1.17	5.00E-05	4.28E-04	Coenzyme transport and metabolism	GTP cyclohydrolase II
EVM0002786	1913.05	4304.47	-1.17	3.55E-03	1.62E-02	--	--
EVM0004710	164.60	370.26	-1.17	5.00E-05	4.28E-04	--	Cgr1 family
EVM0005694	26.91	60.54	-1.17	5.00E-05	4.28E-04	Energy production and conversion	FMN-dependent dehydrogenase

EVM0003646	6.36	14.29	-1.17	2.40E-03	1.18E-02	Translation, ribosomal structure and biogenesis	Methyltransferase small domain
EVM0000837	2.32	5.21	-1.17	4.75E-03	2.06E-02	--	--
EVM0001689	296.64	665.84	-1.17	5.00E-05	4.28E-04	General function prediction only	Ras family
EVM0002411	5.34	11.99	-1.17	2.00E-04	1.46E-03	--	--
EVM0000527	19.05	42.72	-1.17	3.50E-04	2.36E-03	General function prediction only	Methyltransferase domain
EVM0004088	17.22	38.61	-1.16	2.50E-04	1.77E-03	General function prediction only	TPR repeat
EVM0012262	13.02	29.18	-1.16	5.50E-04	3.46E-03	Transcription	SHS2 domain found in N terminus of Rpb7p/Rpc25p/MJ0397
EVM0002860	15.78	35.35	-1.16	5.00E-05	4.28E-04	Secondary metabolites biosynthesis, transport and catabolism	AMP-binding enzyme
EVM0011857	42.81	95.92	-1.16	5.00E-05	4.28E-04	RNA processing and modification	Pescadillo N-terminus
EVM0007607	175.16	392.48	-1.16	5.00E-05	4.28E-04	General function prediction only	TPR repeat
EVM0011559	8.87	19.87	-1.16	3.00E-04	2.07E-03	Energy production and conversion	FAD linked oxidases, C-terminal domain
EVM0007466	18.64	41.73	-1.16	5.00E-05	4.28E-04	General function prediction only	Pentatricopeptide repeat domain
EVM0007916	7.74	17.32	-1.16	2.85E-03	1.36E-02	#N/A	#N/A
EVM0002903	91.91	205.62	-1.16	5.00E-05	4.28E-04	--	--

EVM0007567	3791.38	8481.37	-1.16	2.60E-03	1.26E-02	Posttranslational modification, protein turnover, chaperones	Hsp90 protein
EVM0008422	26.51	59.30	-1.16	2.00E-04	1.46E-03	--	AAA-ATPase Vps4-associated protein 1
EVM0000185	3.01	6.74	-1.16	2.15E-03	1.08E-02	--	Zinc-binding dehydrogenase
EVM0002535	34.20	76.41	-1.16	2.00E-04	1.46E-03	--	--
EVM0011879	38.22	85.35	-1.16	1.00E-04	7.96E-04	General function prediction only	--
EVM0009356	37.28	83.25	-1.16	1.00E-04	7.96E-04	--	TPR repeat
EVM0010715	73.74	164.65	-1.16	5.00E-05	4.28E-04	RNA processing and modification	Isy1-like splicing family
EVM0003417	23.03	51.40	-1.16	2.00E-04	1.46E-03	Translation, ribosomal structure and biogenesis	Ribosome biogenesis protein Nop16
EVM0002183	203.09	453.22	-1.16	5.00E-05	4.28E-04	--	Tetraspanin family
EVM0008178	19.31	43.09	-1.16	1.55E-03	8.22E-03	--	--
EVM0005227	61.43	137.05	-1.16	1.50E-04	1.14E-03	Function unknown	Bromodomain
EVM0005617	249.56	556.44	-1.16	1.50E-04	1.14E-03	--	--
EVM0010946	1.04	2.33	-1.16	1.31E-02	4.63E-02	Energy production and conversion	Pyridine nucleotide-disulphide oxidoreductase
EVM0011592	88.10	196.29	-1.16	3.00E-04	2.07E-03	--	--
EVM0006878	4.36	9.70	-1.15	9.00E-04	5.22E-03	Carbohydrate transport and metabolism	Major Facilitator Superfamily
EVM0008205	7.72	17.17	-1.15	7.00E-04	4.23E-03	Defense mechanisms	NADH(P)-binding
EVM0011062	156.83	348.43	-1.15	3.00E-04	2.07E-03	Energy production and conversion	lactate/malate dehydrogenase, NAD binding domain

EVM0007565	26.17	58.13	-1.15	5.00E-05	4.28E-04	Function unknown	Uncharacterized protein family UPF0029
EVM0007587	16.97	37.70	-1.15	4.00E-04	2.65E-03	--	--
EVM0005633	50.69	112.58	-1.15	1.00E-04	7.96E-04	--	--
EVM0004686	1.24	2.76	-1.15	8.60E-03	3.32E-02	General function prediction only	Major Facilitator Superfamily
EVM0000647	12.35	27.42	-1.15	7.50E-04	4.49E-03	#N/A	#N/A
EVM0001063	10.10	22.42	-1.15	3.00E-04	2.07E-03	--	G protein-coupled glucose receptor regulating Gpa2
EVM0012134	15.64	34.69	-1.15	1.00E-04	7.96E-04	Energy production and conversion	Phosphoenolpyruvate phosphomutase
EVM0003812	20.17	44.74	-1.15	5.00E-05	4.28E-04	--	--
EVM0012166	6.01	13.32	-1.15	5.00E-04	3.19E-03	--	Glyoxal oxidase N-terminus
EVM0001303	207.17	459.15	-1.15	5.00E-05	4.28E-04	--	--
EVM0006165	7.99	17.70	-1.15	5.50E-04	3.46E-03	--	--
EVM0007796	29.71	65.68	-1.14	8.65E-03	3.34E-02	--	--
EVM0007553	8.17	18.07	-1.14	1.00E-03	5.70E-03	RNA processing and modification	Putative methyltransferase
EVM0007009	381.12	842.21	-1.14	3.50E-04	2.36E-03	--	Calcipressin
EVM0011366	12.21	26.99	-1.14	5.00E-05	4.28E-04	--	SDA1
EVM0002755	5.80	12.81	-1.14	2.45E-03	1.20E-02	--	--
EVM0011919	29.00	64.03	-1.14	2.50E-04	1.77E-03	--	--
EVM0000357	3.96	8.74	-1.14	2.55E-03	1.24E-02	Function unknown	AhpC/TSA antioxidant enzyme
EVM0009738	11.76	25.96	-1.14	5.00E-05	4.28E-04	Carbohydrate transport and metabolism	Major Facilitator Superfamily
EVM0001158	40.04	88.33	-1.14	1.00E-04	7.96E-04	--	--

EVM0008736	11.03	24.32	-1.14	1.45E-03	7.77E-03	Secondary metabolites biosynthesis, transport and catabolism	Alcohol dehydrogenase GroES-like domain
EVM0006502	184.02	405.65	-1.14	5.00E-05	4.28E-04	Secondary metabolites biosynthesis, transport and catabolism	Alcohol dehydrogenase GroES-like domain
EVM0006064	4.45	9.81	-1.14	1.00E-04	7.96E-04	--	--
EVM0006652	13.63	30.04	-1.14	1.50E-04	1.14E-03	--	Cytochrome P450
EVM0004838	20.06	44.19	-1.14	5.00E-04	3.19E-03	Function unknown	Uncharacterized protein family UPF0029
EVM0011287	8.39	18.49	-1.14	4.50E-04	2.92E-03	Secondary metabolites biosynthesis, transport and catabolism	Cytochrome P450
EVM0007905	18.04	39.74	-1.14	7.00E-04	4.23E-03	--	Spc19
EVM0004444	14.36	31.61	-1.14	2.50E-04	1.77E-03	--	--
EVM0007747	29.35	64.57	-1.14	4.00E-04	2.65E-03	Function unknown	Domain of unknown function DUF59
EVM0005233	22.61	49.70	-1.14	5.00E-05	4.28E-04	Lipid transport and metabolism	Alpha/beta hydrolase family
EVM0002340	23.42	51.41	-1.13	1.50E-04	1.14E-03	RNA processing and modification	Ribonuclease T2 family
EVM0006440	120.79	265.04	-1.13	2.50E-04	1.77E-03	--	LysM domain
EVM0001623	11.78	25.85	-1.13	5.50E-04	3.46E-03	--	--
EVM0004616	928.46	2036.91	-1.13	9.40E-03	3.57E-02	--	--
EVM0008127	107.17	234.89	-1.13	5.00E-05	4.28E-04	--	SOH1

EVM0006962	35.79	78.44	-1.13	1.50E-04	1.14E-03	Intracellular trafficking, secretion, and vesicular transport	ABC transporter
EVM0001878	35.93	78.74	-1.13	5.00E-05	4.28E-04	--	--
EVM0011693	4.91	10.76	-1.13	2.05E-03	1.03E-02	--	F-box-like
EVM0006860	47.99	105.10	-1.13	3.50E-04	2.36E-03	Function unknown	AMMECR1
EVM0011500	12.11	26.51	-1.13	2.00E-03	1.01E-02	General function prediction only	Putative esterase
EVM0002814	132.04	289.05	-1.13	2.50E-04	1.77E-03	Intracellular trafficking, secretion, and vesicular transport	Peroxisomal membrane protein (Pex16)
EVM0004396	11.85	25.94	-1.13	1.50E-04	1.14E-03	--	--
EVM0004844	7.74	16.93	-1.13	3.50E-04	2.36E-03	--	Domain of unknown function (DUF4139)
EVM0005913	108.71	237.80	-1.13	1.50E-04	1.14E-03	--	Phosphoribosyl transferase domain
EVM0007277	769.92	1683.76	-1.13	2.00E-04	1.46E-03	--	--
EVM0009027	41.30	90.32	-1.13	5.00E-05	4.28E-04	--	F-box-like
EVM0009459	3.79	8.28	-1.13	7.00E-04	4.23E-03	--	--
EVM0007802	205.03	448.20	-1.13	3.00E-04	2.07E-03	--	Protein of unknown function (DUF952)
EVM0011750	6.16	13.44	-1.13	1.40E-03	7.55E-03	Defense mechanisms	NAD dependent epimerase/dehydratase family
EVM0011689	3.03	6.62	-1.13	6.00E-04	3.72E-03	--	non-haem dioxygenase in morphine synthesis N-terminal

EVM0004323	15.17	33.09	-1.12	1.50E-04	1.14E-03	Signal transduction mechanisms	Protein kinase domain
EVM0001028	15.43	33.64	-1.12	4.00E-04	2.65E-03	--	--
EVM0006461	7.64	16.65	-1.12	9.50E-04	5.46E-03	Lipid transport and metabolism	Acyltransferase
EVM0002136	755.55	1646.10	-1.12	5.00E-05	4.28E-04	Translation, ribosomal structure and biogenesis	Ribosomal protein S4/S9 N-terminal domain
EVM0011692	20.42	44.46	-1.12	5.00E-05	4.28E-04	Cell motility	Dynactin p62 family
EVM0010024	2.42	5.27	-1.12	8.85E-03	3.40E-02	--	--
EVM0007043	17.88	38.85	-1.12	5.00E-05	4.28E-04	General function prediction only	Zinc finger, C2H2 type
EVM0005876	7.33	15.94	-1.12	1.15E-03	6.41E-03	--	--
EVM0011335	17.69	38.44	-1.12	5.00E-05	4.28E-04	--	--
EVM0010095	6.01	13.04	-1.12	3.50E-04	2.36E-03	Replication, recombination and repair	DNA polymerase alpha/epsilon subunit B
EVM0005623	15.14	32.85	-1.12	2.00E-04	1.46E-03	--	Glycosyl hydrolases family 11
EVM0002871	13.75	29.81	-1.12	5.00E-05	4.28E-04	--	--
EVM0000005	33.92	73.56	-1.12	4.05E-03	1.81E-02	--	--
EVM0006695	23.69	51.34	-1.12	3.00E-04	2.07E-03	--	Ankyrin repeats (3 copies)
EVM0006793	36.71	79.55	-1.12	2.00E-04	1.46E-03	--	--
EVM0002268	18.32	39.69	-1.11	4.90E-03	2.12E-02	--	--
EVM0007593	128.29	277.65	-1.11	5.00E-05	4.28E-04	RNA processing and modification	Zinc knuckle
EVM0003207	50.97	110.17	-1.11	1.00E-04	7.96E-04	--	--
EVM0007309	8.15	17.62	-1.11	3.00E-04	2.07E-03	Amino acid transport and metabolism	Amino acid permease

EVM0001068	6.07	13.11	-1.11	2.05E-03	1.03E-02	--	Isochorismatase family
EVM0011600	19.24	41.55	-1.11	1.50E-04	1.14E-03	Function unknown	WD domain, G-beta repeat
EVM0009256	35.71	77.09	-1.11	5.00E-05	4.28E-04	Function unknown	Vta1 like
EVM0011563	73.51	158.66	-1.11	1.50E-04	1.14E-03	Posttranslational modification, protein turnover, chaperones	X-domain of DnaJ-containing
EVM0005272	811.62	1751.54	-1.11	8.00E-04	4.73E-03	--	Ubiquinol-cytochrome-c reductase complex subunit (QCR10)
EVM0002387	133.02	286.90	-1.11	1.00E-04	7.96E-04	--	Coiled-coil domain containing protein (DUF2052)
EVM0001635	139.31	300.37	-1.11	1.00E-04	7.96E-04	Posttranslational modification, protein turnover, chaperones	GrpE
EVM0002307	41.78	90.05	-1.11	1.40E-03	7.55E-03	--	--
EVM0001156	167.02	359.98	-1.11	2.00E-04	1.46E-03	--	--
EVM0006907	5.80	12.50	-1.11	2.00E-03	1.01E-02	--	RTA1 like protein
EVM0006559	48.80	105.03	-1.11	5.00E-04	3.19E-03	--	--
EVM0002587	99.13	213.26	-1.11	1.50E-04	1.14E-03	Translation, ribosomal structure and biogenesis	Translation initiation factor eIF3 subunit
EVM0003984	109.50	235.46	-1.10	5.50E-04	3.46E-03	--	Protein of unknown function (DUF3421)
EVM0005472	32.64	70.19	-1.10	5.00E-05	4.28E-04	--	PCI domain
EVM0004813	77.39	166.32	-1.10	4.85E-03	2.10E-02	Inorganic ion transport and metabolism	E1-E2 ATPase

EVM0003574	48.42	103.96	-1.10	5.00E-05	4.28E-04	General function prediction only	PCI domain
EVM0011363	1.07	2.29	-1.10	5.80E-03	2.42E-02	RNA processing and modification	AAA domain
EVM0003670	43.61	93.63	-1.10	1.00E-04	7.96E-04	Chromatin structure and dynamics	ATP-utilising chromatin assembly and remodelling N-terminal
EVM0008302	76.36	163.78	-1.10	3.00E-04	2.07E-03	--	--
EVM0006667	20.61	44.20	-1.10	3.00E-04	2.07E-03	--	--
EVM0006499	14.30	30.67	-1.10	3.50E-04	2.36E-03	--	--
EVM0005941	105.49	226.10	-1.10	5.00E-05	4.28E-04	--	--
EVM0006953	4.79	10.26	-1.10	1.50E-03	8.00E-03	Cytoskeleton	Autophagy protein Atg8 ubiquitin like
EVM0001548	1370.75	2937.56	-1.10	7.50E-04	4.49E-03	Translation, ribosomal structure and biogenesis	Ribosomal protein S19e
EVM0002124	26.15	56.04	-1.10	1.50E-04	1.14E-03	Amino acid transport and metabolism	Dehydratase family
EVM0008208	117.60	251.94	-1.10	1.50E-04	1.14E-03	General function prediction only	DHHC palmitoyltransferase
EVM0012177	23.32	49.96	-1.10	1.00E-04	7.96E-04	General function prediction only	Nuclear protein Es2
EVM0004869	195.04	417.73	-1.10	4.50E-04	2.92E-03	Lipid transport and metabolism	Myo-inositol-1-phosphate synthase
EVM0001429	28.68	61.42	-1.10	2.50E-04	1.77E-03	Inorganic ion transport and metabolism	E1-E2 ATPase
EVM0002826	117.04	250.62	-1.10	1.50E-04	1.14E-03	General function prediction only	Cell differentiation family, Rcd1-like

EVM0004168	4.91	10.51	-1.10	1.45E-03	7.77E-03	Carbohydrate transport and metabolism	FGGY family of carbohydrate kinases, C-terminal domain
EVM0003042	7.64	16.33	-1.10	7.00E-04	4.23E-03	--	--
EVM0010344	1807.48	3862.04	-1.10	2.00E-04	1.46E-03	--	ATP synthase complex subunit h
EVM0011479	121.53	259.64	-1.10	1.00E-04	7.96E-04	Posttranslational modification, protein turnover, chaperones	PI31 proteasome regulator N-terminal
EVM0006354	51.55	110.11	-1.09	6.75E-03	2.74E-02	RNA processing and modification	RNA recognition motif. (a.k.a. RRM, RBD, or RNP domain)
EVM0006685	46.84	99.99	-1.09	4.50E-04	2.92E-03	Amino acid transport and metabolism	Amino acid kinase family
EVM0008724	6.59	14.07	-1.09	5.50E-04	3.46E-03	General function prediction only	Major Facilitator Superfamily
EVM0000211	29.99	64.00	-1.09	3.50E-04	2.36E-03	RNA processing and modification	WD domain, G-beta repeat
EVM0010511	5.93	12.64	-1.09	7.50E-04	4.49E-03	--	WD domain, G-beta repeat
EVM0002578	1.66	3.54	-1.09	3.15E-03	1.47E-02	General function prediction only	Glycosyl transferase family 90
EVM0010235	2.28	4.86	-1.09	4.65E-03	2.03E-02	--	--
EVM0006976	19.35	41.25	-1.09	4.50E-04	2.92E-03	Inorganic ion transport and metabolism	Regulator of volume decrease after cellular swelling
EVM0001036	75.92	161.76	-1.09	4.00E-04	2.65E-03	--	--
EVM0011220	39.24	83.55	-1.09	4.00E-04	2.65E-03	--	Glyoxalase-like domain
EVM0004432	7.40	15.73	-1.09	5.50E-04	3.46E-03	Secondary metabolites biosynthesis, transport and catabolism	Pyridine nucleotide-disulphide oxidoreductase

EVM0006511	4.86	10.33	-1.09	4.00E-04	2.65E-03	General function prediction only	Glycosyl transferase family 90
EVM0001108	21.96	46.71	-1.09	2.00E-04	1.46E-03	Translation, ribosomal structure and biogenesis	Putative diphthamide synthesis protein
EVM0004930	9.52	20.24	-1.09	3.50E-04	2.36E-03	--	Flavin containing amine oxidoreductase
EVM0002825	59.93	127.40	-1.09	1.50E-04	1.14E-03	--	--
EVM0005366	22.53	47.83	-1.09	2.50E-04	1.77E-03	Function unknown	pre-RNA processing PIH1/Nop17
EVM0007349	219.77	466.55	-1.09	1.50E-04	1.14E-03	Signal transduction mechanisms	EF hand
EVM0012075	8.57	18.19	-1.09	1.70E-03	8.87E-03	--	N-acetyltransferase
EVM0001205	34.79	73.84	-1.09	2.00E-04	1.46E-03	Function unknown	Ribosomal protein L1p/L10e family
EVM0011226	140.90	298.96	-1.09	4.50E-04	2.92E-03	RNA processing and modification	Zinc-finger double-stranded RNA-binding
EVM0001902	58.73	124.62	-1.09	1.50E-04	1.14E-03	Energy production and conversion	Lyase
EVM0008552	6.58	13.96	-1.08	1.45E-03	7.77E-03	--	--
EVM0011908	5.87	12.44	-1.08	5.45E-03	2.31E-02	Function unknown	2OG-Fe(II) oxygenase superfamily
EVM0002991	30.04	63.69	-1.08	7.50E-04	4.49E-03	--	--
EVM0008796	25.88	54.84	-1.08	5.00E-05	4.28E-04	Defense mechanisms	Dual specificity phosphatase, catalytic domain
EVM0006154	33.57	71.12	-1.08	3.00E-04	2.07E-03	Carbohydrate transport and metabolism	Fructose-bisphosphate aldolase class-I

EVM0008693	21.23	44.96	-1.08	7.50E-04	4.49E-03	Lipid transport and metabolism	short chain dehydrogenase
EVM0003382	16.69	35.33	-1.08	1.50E-04	1.14E-03	Translation, ribosomal structure and biogenesis	Dihydrouridine synthase (Dus)
EVM0007932	57.02	120.65	-1.08	2.50E-04	1.77E-03	Posttranslational modification, protein turnover, chaperones	Protein-L-isoaspartate(D-aspartate) O-methyltransferase (PCMT)
EVM0000800	428.60	906.54	-1.08	3.75E-03	1.70E-02	--	Protein of unknown function (DUF4449)
EVM0009850	4.07	8.61	-1.08	4.50E-04	2.92E-03	Replication, recombination and repair	MutS domain V
EVM0008397	197.98	418.43	-1.08	5.50E-04	3.46E-03	--	--
EVM0009133	10.19	21.53	-1.08	5.00E-04	3.19E-03	--	Peptidase family M28
EVM0010176	40.75	86.02	-1.08	1.00E-04	7.96E-04	Posttranslational modification, protein turnover, chaperones	Dynamin family
EVM0009047	17.25	36.39	-1.08	3.50E-04	2.36E-03	General function prediction only	WD domain, G-beta repeat
EVM0009512	25.84	54.50	-1.08	3.00E-04	2.07E-03	Lipid transport and metabolism	CoA-transferase family III
EVM0003425	7.21	15.18	-1.07	5.00E-05	4.28E-04	--	Formin Homology 2 Domain
EVM0008014	1525.28	3211.64	-1.07	1.50E-04	1.14E-03	Translation, ribosomal structure and biogenesis	Ribosomal protein L35Ae
EVM0005754	7.03	14.80	-1.07	1.31E-02	4.64E-02	--	--

EVM0002212	14.52	30.55	-1.07	1.50E-03	8.00E-03	Posttranslational modification, protein turnover, chaperones	--
EVM0009084	64.15	134.98	-1.07	5.00E-05	4.28E-04	Translation, ribosomal structure and biogenesis	Histidyl-tRNA synthetase
EVM0008008	6.82	14.34	-1.07	9.00E-04	5.22E-03	--	Heterokaryon incompatibility protein (HET)
EVM0011714	96.69	203.25	-1.07	1.50E-04	1.14E-03	Function unknown	Protein of unknown function (DUF1279)
EVM0008615	209.09	439.47	-1.07	4.00E-04	2.65E-03	General function prediction only	Thi4 family
EVM0000289	31.95	67.13	-1.07	1.50E-04	1.14E-03	RNA processing and modification	RNA recognition motif. (a.k.a. RRM, RBD, or RNP domain)
EVM0000096	1.57	3.30	-1.07	3.55E-03	1.62E-02	--	--
EVM0002576	15.71	33.00	-1.07	5.00E-05	4.28E-04	--	MYND finger
EVM0007337	22.56	47.38	-1.07	7.00E-04	4.23E-03	Carbohydrate transport and metabolism	Class II Aldolase and Adducin N-terminal domain
EVM0004284	5.08	10.66	-1.07	5.00E-04	3.19E-03	--	WSC domain
EVM0004735	7.15	15.00	-1.07	2.00E-04	1.46E-03	--	50S ribosome-binding GTPase
EVM0006851	25.64	53.80	-1.07	5.00E-04	3.19E-03	--	--
EVM0000774	41.07	86.18	-1.07	5.00E-05	4.28E-04	--	--
EVM0006313	4.40	9.24	-1.07	1.05E-02	3.88E-02	General function prediction only	C2H2-type zinc finger
EVM0000642	26.67	55.95	-1.07	1.00E-04	7.96E-04	--	--
EVM0001741	2.66	5.57	-1.07	1.50E-03	8.00E-03	--	RNA recognition motif. (a.k.a. RRM, RBD, or RNP domain)

EVM0006493	123.41	258.75	-1.07	1.50E-04	1.14E-03	--	N-acetylglucosaminyl transferase component (Gpi1)
EVM0006380	3.84	8.06	-1.07	3.00E-03	1.42E-02	--	Glyoxal oxidase N-terminus
EVM0000560	3.98	8.34	-1.07	2.25E-03	1.12E-02	--	Ferric reductase like transmembrane component
EVM0004244	13.39	28.03	-1.07	4.00E-04	2.65E-03	--	F-box-like
EVM0010713	16.16	33.82	-1.07	3.50E-04	2.36E-03	Function unknown	Protein of unknown function (DUF890)
EVM0004606	45.25	94.67	-1.07	1.50E-04	1.14E-03	--	Sel1 repeat
EVM0002257	28.03	58.63	-1.06	3.50E-04	2.36E-03	--	--
EVM0003798	4.06	8.48	-1.06	8.40E-03	3.26E-02	--	Thaumatin family
EVM0002854	44.72	93.53	-1.06	3.00E-04	2.07E-03	Secondary metabolites biosynthesis, transport and catabolism	Multicopper oxidase
EVM0008213	1093.56	2286.68	-1.06	1.50E-04	1.14E-03	RNA processing and modification	LSM domain
EVM0008930	78.58	164.28	-1.06	6.00E-04	3.72E-03	--	--
EVM0002777	125.23	261.73	-1.06	5.50E-04	3.46E-03	--	--
EVM0005540	37.24	77.79	-1.06	1.70E-03	8.87E-03	--	--
EVM0002843	20.14	42.04	-1.06	2.50E-04	1.77E-03	--	FAD binding domain
EVM0006400	3.44	7.19	-1.06	5.15E-03	2.20E-02	--	--
EVM0010109	8.69	18.14	-1.06	9.50E-04	5.46E-03	Replication, recombination and repair	Eukaryotic and archaeal DNA primase, large subunit
EVM0008011	47.05	98.22	-1.06	3.50E-04	2.36E-03	--	--
EVM0007046	5.36	11.19	-1.06	3.05E-03	1.44E-02	--	--

EVM0010556	7.18	14.97	-1.06	1.75E-03	9.08E-03	General function prediction only	Major Facilitator Superfamily
EVM0001163	107.11	223.33	-1.06	4.50E-04	2.92E-03	--	Protein kinase domain
EVM0005895	30.66	63.91	-1.06	4.00E-04	2.65E-03	--	--
EVM0011785	4.26	8.87	-1.06	4.00E-04	2.65E-03	--	Fungal specific transcription factor domain
EVM0007252	56.37	117.46	-1.06	2.50E-04	1.77E-03	Secondary metabolites biosynthesis, transport and catabolism	Pyridine nucleotide-disulphide oxidoreductase
EVM0007781	2.77	5.76	-1.06	1.11E-02	4.06E-02	Amino acid transport and metabolism	Carbon-nitrogen hydrolase
EVM0009119	11.83	24.63	-1.06	1.00E-03	5.70E-03	--	--
EVM0009780	34.23	71.27	-1.06	2.50E-04	1.77E-03	Translation, ribosomal structure and biogenesis	3' exoribonuclease family, domain 1
EVM0005500	16.81	34.99	-1.06	8.00E-04	4.73E-03	Function unknown	XAP5, circadian clock regulator
EVM0001475	12.14	25.27	-1.06	3.50E-04	2.36E-03	--	--
EVM0002127	55.73	115.98	-1.06	1.50E-04	1.14E-03	RNA processing and modification	RNA recognition motif. (a.k.a. RRM, RBD, or RNP domain)
EVM0002623	12.13	25.21	-1.06	1.50E-04	1.14E-03	--	WD domain, G-beta repeat
EVM0008843	14.89	30.96	-1.06	2.00E-04	1.46E-03	--	--
EVM0010675	15.43	32.07	-1.06	3.00E-04	2.07E-03	General function prediction only	MatE
EVM0006677	24.68	51.24	-1.05	8.00E-04	4.73E-03	--	--
EVM0000516	39.43	81.84	-1.05	3.50E-04	2.36E-03	--	--
EVM0007866	47.43	98.12	-1.05	3.50E-04	2.36E-03	--	Peptidase M50B-like
EVM0007883	184.74	382.04	-1.05	1.50E-04	1.14E-03	--	--

EVM0008605	13.83	28.61	-1.05	5.00E-04	3.19E-03	Defense mechanisms	NAD dependent epimerase/dehydratase family
EVM0006618	86.81	179.49	-1.05	2.50E-04	1.77E-03	--	--
EVM0008262	55.05	113.83	-1.05	2.50E-04	1.77E-03	General function prediction only	NAD binding domain of 6-phosphogluconate dehydrogenase
EVM0004911	537.24	1110.72	-1.05	3.00E-04	2.07E-03	--	--
EVM0001770	6.78	14.01	-1.05	2.00E-03	1.01E-02	--	Lactonase, 7-bladed beta-propeller
EVM0007582	4.73	9.77	-1.05	2.25E-03	1.12E-02	Carbohydrate transport and metabolism	Glycosyl hydrolase family 47
EVM0000771	8.17	16.87	-1.05	1.10E-03	6.17E-03	--	--
EVM0004801	33.45	69.09	-1.05	5.00E-05	4.28E-04	--	--
EVM0005561	65.72	135.72	-1.05	5.00E-05	4.28E-04	Transcription	Transcription factor TFIIB repeat
EVM0008064	17.02	35.15	-1.05	3.50E-04	2.36E-03	--	Shugoshin C terminus
EVM0006335	118.87	245.18	-1.04	1.38E-02	4.83E-02	--	--
EVM0003787	76.12	157.01	-1.04	1.00E-04	7.96E-04	Translation, ribosomal structure and biogenesis	Eukaryotic translation initiation factor 3 subunit 7 (eIF-3)
EVM0008186	121.03	249.60	-1.04	6.50E-04	3.98E-03	General function prediction only	Translation initiation factor SUI1
EVM0003484	14.96	30.84	-1.04	7.50E-04	4.49E-03	--	Major Facilitator Superfamily
EVM0000528	256.48	528.79	-1.04	7.90E-03	3.10E-02	Lipid transport and metabolism	Acyl-CoA dehydrogenase, C-terminal domain
EVM0001528	42.72	88.05	-1.04	2.00E-04	1.46E-03	RNA processing and modification	DEAD/DEAH box helicase
EVM0009691	36.99	76.22	-1.04	2.50E-04	1.77E-03	Carbohydrate transport and metabolism	pfkB family carbohydrate kinase

EVM0005748	42.07	86.68	-1.04	2.00E-04	1.46E-03	--	--
EVM0001487	56.18	115.73	-1.04	2.00E-04	1.46E-03	--	Transcriptional regulator of RNA polII, SAGA, subunit
EVM0011835	78.81	162.34	-1.04	3.50E-04	2.36E-03	Posttranslational modification, protein turnover, chaperones	Ring finger domain
EVM0012127	23.24	47.84	-1.04	2.50E-04	1.77E-03	--	PXA domain
EVM0004852	185.42	381.61	-1.04	1.90E-03	9.73E-03	RNA processing and modification	LSM domain
EVM0004518	4.97	10.22	-1.04	2.15E-03	1.08E-02	Energy production and conversion	Aldehyde dehydrogenase family
EVM0005978	37.96	78.09	-1.04	1.50E-04	1.14E-03	--	Rab-GTPase-TBC domain
EVM0001385	25.85	53.16	-1.04	3.00E-04	2.07E-03	--	G-protein alpha subunit
EVM0001725	24.27	49.91	-1.04	3.00E-04	2.07E-03	RNA processing and modification	AAA domain
EVM0008651	112.98	232.33	-1.04	2.50E-04	1.77E-03	Inorganic ion transport and metabolism	Copper/zinc superoxide dismutase (SODC)
EVM0004948	19.19	39.36	-1.04	2.20E-03	1.10E-02	--	--
EVM0006887	6.91	14.17	-1.04	2.00E-03	1.01E-02	--	--
EVM0003428	48.39	99.23	-1.04	2.00E-04	1.46E-03	Extracellular structures	Cofilin/tropomyosin-type actin-binding protein
EVM0003811	4.68	9.61	-1.04	1.20E-03	6.64E-03	--	--
EVM0011083	8.76	17.95	-1.04	6.00E-04	3.72E-03	--	--
EVM0009958	13.74	28.17	-1.04	3.05E-03	1.44E-02	--	--
EVM0009777	36.81	75.42	-1.03	3.00E-04	2.07E-03	General function prediction only	Putative methyltransferase

EVM0007937	44.66	91.49	-1.03	3.00E-04	2.07E-03	Posttranslational modification, protein turnover, chaperones	Zinc finger, C3HC4 type (RING finger)
EVM0000938	23.74	48.64	-1.03	2.00E-04	1.46E-03	Posttranslational modification, protein turnover, chaperones	Cyclophilin type peptidyl-prolyl cis-trans isomerase/CLD
EVM0001935	22.42	45.90	-1.03	3.00E-04	2.07E-03	--	Myosin-like coiled-coil protein
EVM0000379	5.57	11.40	-1.03	1.90E-03	9.73E-03	--	HNH endonuclease
EVM0010974	11.49	23.50	-1.03	6.50E-04	3.98E-03	Signal transduction mechanisms	OPT oligopeptide transporter protein
EVM0006096	23.67	48.41	-1.03	2.00E-04	1.46E-03	--	PH domain
EVM0002921	9.49	19.40	-1.03	6.00E-04	3.72E-03	Lipid transport and metabolism	AMP-binding enzyme
EVM0005087	137.39	280.83	-1.03	3.00E-04	2.07E-03	Intracellular trafficking, secretion, and vesicular transport	Tim17/Tim22/Tim23/Pmp24 family
EVM0005207	4.99	10.19	-1.03	2.55E-03	1.24E-02	--	Ubiquitin family
EVM0006139	3.30	6.75	-1.03	4.80E-03	2.08E-02	Transcription	HMG (high mobility group) box
EVM0008671	14.93	30.48	-1.03	8.50E-04	4.98E-03	--	--
EVM0009434	9.40	19.18	-1.03	2.75E-03	1.32E-02	General function prediction only	Ankyrin repeats (3 copies)
EVM0011210	280.69	572.05	-1.03	3.50E-04	2.36E-03	--	DASH complex subunit Dad2
EVM0011535	183.89	374.69	-1.03	5.50E-04	3.46E-03	--	Protein of unknown function (DUF3632)
EVM0002894	15.66	31.89	-1.03	2.00E-04	1.46E-03	Transcription	Protein kinase domain

EVM0001380	5.63	11.46	-1.03	2.00E-03	1.01E-02	General function prediction only	Major Facilitator Superfamily
EVM0010850	67.11	136.69	-1.03	7.00E-04	4.23E-03	Secondary metabolites biosynthesis, transport and catabolism	short chain dehydrogenase
EVM0005091	30.98	63.09	-1.03	6.50E-04	3.98E-03	--	TEA/ATTS domain family
EVM0003753	31.01	63.14	-1.03	7.00E-04	4.23E-03	Amino acid transport and metabolism	Aminotransferase class-V
EVM0004303	15.95	32.46	-1.03	2.00E-04	1.46E-03	Secondary metabolites biosynthesis, transport and catabolism	ABC transporter
EVM0010536	2.15	4.37	-1.03	9.85E-03	3.70E-02	--	--
EVM0009990	9.20	18.72	-1.03	9.00E-04	5.22E-03	--	Arrestin (or S-antigen), C-terminal domain
EVM0003625	181.86	369.87	-1.02	8.00E-04	4.73E-03	Carbohydrate transport and metabolism	Alpha amylase, catalytic domain
EVM0006366	2.69	5.46	-1.02	7.05E-03	2.83E-02	--	--
EVM0002321	157.75	320.70	-1.02	5.95E-03	2.47E-02	Energy production and conversion	Mitochondrial carrier protein
EVM0010583	443.78	902.05	-1.02	6.00E-04	3.72E-03	--	--
EVM0001247	90.27	183.43	-1.02	1.80E-03	9.29E-03	--	MAC/Perforin domain
EVM0003414	6.24	12.69	-1.02	2.65E-03	1.28E-02	--	--
EVM0008363	46.52	94.51	-1.02	2.00E-04	1.46E-03	--	--
EVM0007250	4.05	8.22	-1.02	5.50E-03	2.32E-02	--	Methyltransferase domain

EVM0007907	23.10	46.91	-1.02	3.00E-04	2.07E-03	Function unknown	Rix1 complex component involved in 60S ribosome maturation
EVM0011322	41.54	84.22	-1.02	1.95E-03	9.93E-03	Intracellular trafficking, secretion, and vesicular transport	N-terminal region of Chorein, a TM vesicle-mediated sorter
EVM0005197	9.63	19.52	-1.02	1.20E-03	6.64E-03	General function prediction only	Major Facilitator Superfamily
EVM0011060	51.45	104.27	-1.02	1.00E-04	7.96E-04	Translation, ribosomal structure and biogenesis	tRNA synthetase class II core domain (G, H, P, S and T)
EVM0007270	1.05	2.14	-1.02	8.75E-03	3.37E-02	--	--
EVM0004319	36.64	74.19	-1.02	4.00E-04	2.65E-03	--	Protein of unknown function (DUF2962)
EVM0000252	18.24	36.90	-1.02	7.00E-04	4.23E-03	--	--
EVM0002524	8.64	17.47	-1.02	1.05E-03	5.94E-03	--	Pectate lyase
EVM0010382	67.33	136.15	-1.02	6.50E-04	3.98E-03	--	--
EVM0000277	7.97	16.11	-1.02	1.30E-03	7.10E-03	Posttranslational modification, protein turnover, chaperones	Eukaryotic aspartyl protease
EVM0010218	21.73	43.92	-1.02	2.50E-04	1.77E-03	--	Methyltransferase domain
EVM0011153	189.73	383.39	-1.01	6.50E-04	3.98E-03	--	Tim17/Tim22/Tim23/Pmp24 family
EVM0010232	577.71	1167.18	-1.01	7.50E-04	4.49E-03	Intracellular trafficking, secretion, and vesicular transport	Nuclear transport factor 2 (NTF2) domain
EVM0009614	73.49	148.46	-1.01	2.50E-04	1.77E-03	--	--

EVM0005970	36.07	72.85	-1.01	2.65E-03	1.28E-02	Amino acid transport and metabolism	3-hydroxyanthranilic acid dioxygenase
EVM0002686	66.96	135.23	-1.01	3.50E-04	2.36E-03	--	--
EVM0007223	35.15	70.99	-1.01	3.00E-04	2.07E-03	Translation, ribosomal structure and biogenesis	Poly A polymerase head domain
EVM0000491	24.45	49.38	-1.01	2.50E-04	1.77E-03	--	Ferric reductase NAD binding domain
EVM0009482	50.90	102.78	-1.01	4.00E-04	2.65E-03	General function prediction only	Putative serine esterase (DUF676)
EVM0004386	21.99	44.40	-1.01	4.00E-04	2.65E-03	RNA processing and modification	DEAD/DEAH box helicase
EVM0008501	6.12	12.34	-1.01	2.15E-03	1.08E-02	Function unknown	--
EVM0000097	3.63	7.32	-1.01	2.75E-03	1.32E-02	--	--
EVM0008241	67.52	136.12	-1.01	1.50E-04	1.14E-03	Amino acid transport and metabolism	Pyridoxal-dependent decarboxylase conserved domain
EVM0010572	5.44	10.96	-1.01	1.65E-03	8.65E-03	Signal transduction mechanisms	Carboxylesterase family
EVM0011381	43.85	88.38	-1.01	6.00E-04	3.72E-03	--	--
EVM0002805	149.26	300.83	-1.01	5.50E-04	3.46E-03	Intracellular trafficking, secretion, and vesicular transport	Translocation protein Sec62
EVM0000600	108.52	218.54	-1.01	7.00E-04	4.23E-03	--	Nitronate monooxygenase
EVM0004430	86.55	174.17	-1.01	3.50E-04	2.36E-03	Signal transduction mechanisms	Stage II sporulation protein E (SpoIIE)
EVM0002082	171.89	345.89	-1.01	7.50E-04	4.49E-03	Carbohydrate transport and metabolism	Triosephosphate isomerase

EVM0005419	45.82	92.16	-1.01	4.00E-04	2.65E-03	--	--
EVM0009125	120.09	241.50	-1.01	4.00E-04	2.65E-03	General function prediction only	Dienelactone hydrolase family
EVM0000152	3.40	6.84	-1.01	4.65E-03	2.03E-02	--	--
EVM0009051	454.36	913.61	-1.01	3.50E-04	2.36E-03	Translation, ribosomal structure and biogenesis	Ribosomal protein L34
EVM0007795	13.44	27.02	-1.01	5.50E-04	3.46E-03	Secondary metabolites biosynthesis, transport and catabolism	Cytochrome P450
EVM0011806	977.12	1963.32	-1.01	1.15E-03	6.41E-03	Translation, ribosomal structure and biogenesis	Ribosomal protein L18e/L15
EVM0006775	26.74	53.71	-1.01	4.50E-04	2.92E-03	--	--
EVM0005389	1092.62	2194.80	-1.01	1.20E-03	6.64E-03	--	Cerato-platanin
EVM0002230	13.41	26.94	-1.01	1.00E-03	5.70E-03	--	Lactonase, 7-bladed beta-propeller
EVM0000935	45.88	92.11	-1.01	8.00E-04	4.73E-03	--	--
EVM0003011	2.53	5.07	-1.01	6.80E-03	2.75E-02	General function prediction only	Zinc finger, C2H2 type
EVM0010312	23.60	47.37	-1.01	2.50E-04	1.77E-03	Cell cycle control, cell division, chromosome partitioning	Protein kinase domain
EVM0009225	17.51	35.10	-1.00	5.50E-04	3.46E-03	General function prediction only	Ankyrin repeats (many copies)
EVM0011952	151.13	302.69	-1.00	5.50E-04	3.46E-03	Transcription	Helix-loop-helix DNA-binding domain
EVM0009227	12.72	25.47	-1.00	1.70E-03	8.87E-03	--	CrcB-like protein

EVM0008788	11.23	22.49	-1.00	2.40E-03	1.18E-02	--	Protein of unknown function (DUF1479)
EVM0001226	36.02	72.13	-1.00	4.50E-04	2.92E-03	Energy production and conversion	Transketolase, pyrimidine binding domain
EVM0008939	42.64	85.38	-1.00	5.00E-04	3.19E-03	RNA processing and modification	Hypothetical methyltransferase
EVM0000637	15.80	31.63	-1.00	1.55E-03	8.22E-03	--	Cytochrome P450
EVM0000668	147.27	294.81	-1.00	5.05E-03	2.17E-02	--	--
EVM0007048	1.94	3.89	-1.00	7.35E-03	2.93E-02	--	--
EVM0000562	39.98	80.01	-1.00	3.50E-04	2.36E-03	Intracellular trafficking, secretion, and vesicular transport	Signal recognition particle, alpha subunit, N-terminal
EVM0004028	92.88	185.86	-1.00	6.00E-04	3.72E-03	Cell cycle control, cell division, chromosome partitioning	--
EVM0005550	195.08	390.22	-1.00	1.00E-03	5.70E-03	--	50S ribosome-binding GTPase
EVM0010163	515.27	257.56	1.00	2.25E-03	1.12E-02	--	F-box-like
EVM0001489	52.74	26.35	1.00	8.00E-04	4.73E-03	General function prediction only	Enoyl-(Acyl carrier protein) reductase
EVM0002143	44.76	22.34	1.00	6.50E-04	3.98E-03	Cytoskeleton	Kinesin motor domain
EVM0005647	14.66	7.32	1.00	6.85E-03	2.77E-02	--	Alpha/beta hydrolase family
EVM0012029	100.94	50.31	1.00	2.00E-03	1.01E-02	--	PLAC8 family
EVM0002785	37.30	18.59	1.00	4.00E-04	2.65E-03	Inorganic ion transport and metabolism	Sodium:sulfate symporter transmembrane region
EVM0000108	44.65	22.23	1.01	4.15E-03	1.85E-02	--	Amidohydrolase
EVM0001781	35.27	17.54	1.01	3.50E-04	2.36E-03	--	--

EVM0007746	19.35	9.62	1.01	1.15E-03	6.41E-03	--	JmjC domain, hydroxylase
EVM0010927	41.23	20.49	1.01	1.60E-03	8.44E-03	--	BTB/POZ domain
EVM0000749	211.41	105.01	1.01	3.00E-04	2.07E-03	General function prediction only	Ras family
EVM0009149	143.42	71.22	1.01	2.00E-04	1.46E-03	--	--
EVM0000304	78.68	39.06	1.01	5.00E-04	3.19E-03	--	Sel1 repeat
EVM0012125	33.28	16.51	1.01	5.00E-04	3.19E-03	--	Protein of unknown function (DUF563)
EVM0012100	142.10	70.50	1.01	7.50E-04	4.49E-03	General function prediction only	Carboxylesterase family
EVM0008890	11.81	5.85	1.01	8.50E-04	4.98E-03	RNA processing and modification	Sec63 Brl domain
EVM0008784	23.12	11.46	1.01	4.00E-04	2.65E-03	Cytoskeleton	Kinesin motor domain
EVM0000751	25.51	12.63	1.01	9.00E-04	5.22E-03	Replication, recombination and repair	Ku70/Ku80 beta-barrel domain
EVM0010762	37.35	18.49	1.01	7.00E-04	4.23E-03	--	SHNi-TPR
EVM0001119	40.49	20.03	1.02	4.50E-04	2.92E-03	Signal transduction mechanisms	Response regulator receiver domain
EVM0005900	25.10	12.40	1.02	8.50E-04	4.98E-03	Posttranslational modification, protein turnover, chaperones	BCS1 N terminal
EVM0006412	443.80	219.32	1.02	7.00E-04	4.23E-03	--	Bin/amphiphysin/Rvs domain for vesicular trafficking
EVM0002222	8.09	4.00	1.02	7.15E-03	2.86E-02	--	Cytochrome P450
EVM0004780	7.22	3.56	1.02	2.45E-03	1.20E-02	Replication, recombination and repair	DEAD/DEAH box helicase

EVM0004931	73.24	36.10	1.02	4.50E-04	2.92E-03	--	Munc13 (mammalian uncoordinated) homology domain
EVM0000011	237.20	116.84	1.02	8.00E-04	4.73E-03	Posttranslational modification, protein turnover, chaperones	Peptidase family M3
EVM0004776	43.15	21.25	1.02	8.00E-04	4.73E-03	Intracellular trafficking, secretion, and vesicular transport	Vps5 C terminal like
EVM0002790	20.24	9.96	1.02	4.45E-03	1.96E-02	--	--
EVM0012178	20.78	10.22	1.02	1.00E-03	5.70E-03	Defense mechanisms	NADH(P)-binding
EVM0008401	28.07	13.80	1.02	1.00E-04	7.96E-04	Posttranslational modification, protein turnover, chaperones	Ubiquitin carboxyl-terminal hydrolase
EVM0008398	54.87	26.95	1.03	3.00E-04	2.07E-03	--	--
EVM0009108	2367.33	1161.26	1.03	1.02E-02	3.80E-02	--	Septin
EVM0006883	32.11	15.75	1.03	3.00E-04	2.07E-03	Secondary metabolites biosynthesis, transport and catabolism	ABC transporter
EVM0006534	14.98	7.34	1.03	1.35E-03	7.33E-03	Replication, recombination and repair	Origin recognition complex (ORC) subunit 5 C-terminus
EVM0008192	156.48	76.59	1.03	7.00E-04	4.23E-03	Posttranslational modification, protein turnover, chaperones	ThiF family
EVM0001797	12.30	6.01	1.03	6.40E-03	2.62E-02	--	--
EVM0006410	336.54	164.42	1.03	2.05E-03	1.03E-02	--	Importin-beta N-terminal domain

EVM0011665	48.52	23.71	1.03	1.00E-04	7.96E-04	Signal transduction mechanisms	Protein kinase domain
EVM0004207	576.82	281.40	1.04	8.50E-04	4.98E-03	--	NAD dependent epimerase/dehydratase family
EVM0002885	130.51	63.65	1.04	4.00E-04	2.65E-03	Transcription	G10 protein
EVM0006334	26.39	12.87	1.04	5.00E-04	3.19E-03	--	--
EVM0000993	3071.48	1494.52	1.04	1.16E-02	4.22E-02	--	--
EVM0009846	20.45	9.94	1.04	8.00E-04	4.73E-03	General function prediction only	Sugar (and other) transporter
EVM0011666	10.73	5.21	1.04	1.65E-03	8.65E-03	--	MYND finger
EVM0012087	136.85	66.30	1.05	8.50E-04	4.98E-03	General function prediction only	Animal haem peroxidase
EVM0012149	59.55	28.84	1.05	4.50E-04	2.92E-03	--	Beta-lactamase superfamily domain
EVM0007994	36.59	17.69	1.05	6.50E-04	3.98E-03	--	--
EVM0009638	8.14	3.94	1.05	1.75E-03	9.08E-03	--	--
EVM0010723	153.49	74.20	1.05	5.00E-04	3.19E-03	Intracellular trafficking, secretion, and vesicular transport	Coatomer WD associated region
EVM0006771	235.40	113.78	1.05	2.50E-04	1.77E-03	Carbohydrate transport and metabolism	Alpha amylase, catalytic domain
EVM0010223	32.65	15.78	1.05	3.00E-04	2.07E-03	--	Permease family
EVM0007015	44.83	14.14	1.66	5.00E-05	4.28E-04	--	Acyl transferase domain
EVM0000926	150.75	72.77	1.05	1.50E-03	8.00E-03	--	--
EVM0008039	347.46	167.53	1.05	1.65E-03	8.65E-03	--	--
EVM0000512	6.92	3.34	1.05	1.05E-02	3.89E-02	--	--

EVM0003731	143.83	69.16	1.06	7.50E-04	4.49E-03	General function prediction only	RNA recognition motif. (a.k.a. RRM, RBD, or RNP domain)
EVM0011572	416.72	199.92	1.06	7.50E-04	4.49E-03	--	Beta-glucan synthesis-associated protein (SKN1)
EVM0008222	18.30	8.77	1.06	4.50E-04	2.92E-03	Cell cycle control, cell division, chromosome partitioning	Domain of unknown function (DUF3635)
EVM0005404	24.27	11.64	1.06	1.00E-04	7.96E-04	--	RecF/RecN/SMC N terminal domain
EVM0004203	13.00	6.23	1.06	1.00E-04	7.96E-04	RNA processing and modification	SEN1 N terminal
EVM0000520	42.09	20.17	1.06	3.00E-04	2.07E-03	General function prediction only	Josephin
EVM0004568	273.05	130.74	1.06	8.00E-04	4.73E-03	--	Cellulase (glycosyl hydrolase family 5)
EVM0003688	39.63	18.97	1.06	5.00E-05	4.28E-04	Cell cycle control, cell division, chromosome partitioning	Mad3/BUB1 homology region 1
EVM0006759	95.45	45.63	1.06	1.50E-04	1.14E-03	Intracellular trafficking, secretion, and vesicular transport	ABC transporter transmembrane region
EVM0009500	100.93	48.23	1.07	3.50E-04	2.36E-03	--	Domain of unknown function (DUF1708)
EVM0004100	207.69	99.22	1.07	5.00E-05	4.28E-04	--	F-box domain
EVM0003463	1.96	0.93	1.07	1.02E-02	3.81E-02	--	--

EVM0008313	21.42	10.18	1.07	3.50E-04	2.36E-03	Secondary metabolites biosynthesis, transport and catabolism	ABC transporter transmembrane region
EVM0001336	53.25	25.30	1.07	5.40E-03	2.29E-02	Signal transduction mechanisms	HIT domain
EVM0001629	11.58	5.49	1.08	1.70E-03	8.87E-03	--	--
EVM0003437	16.24	7.70	1.08	5.00E-04	3.19E-03	Posttranslational modification, protein turnover, chaperones	Tubulin folding cofactor D C terminal
EVM0007014	90.12	42.71	1.08	4.00E-04	2.65E-03	General function prediction only	Acetyltransferase (GNAT) family
EVM0008722	195.10	92.39	1.08	4.50E-04	2.92E-03	Inorganic ion transport and metabolism	Alkaline phosphatase
EVM0001532	1195.11	565.38	1.08	6.00E-04	3.72E-03	--	Septin
EVM0003658	7.01	3.31	1.08	3.40E-03	1.57E-02	--	--
EVM0009761	224.83	106.26	1.08	1.00E-04	7.96E-04	Posttranslational modification, protein turnover, chaperones	PCI domain
EVM0002371	1390.32	656.79	1.08	3.00E-04	2.07E-03	Chromatin structure and dynamics	Core histone H2A/H2B/H3/H4
EVM0003151	21.52	10.16	1.08	1.30E-03	7.10E-03	--	Phosphotyrosyl phosphate activator (PTPA) protein
EVM0008332	13.68	6.45	1.08	2.05E-03	1.03E-02	--	--
EVM0011537	141.11	66.51	1.09	2.00E-04	1.46E-03	Transcription	Histone-like transcription factor (CBF/NF-Y) and archaeal histone

EVM0000618	28.71	13.52	1.09	2.00E-04	1.46E-03	RNA processing and modification	Suppressor of forked protein (Suf)
EVM0003945	216.14	101.73	1.09	1.35E-03	7.33E-03	Cell wall/membrane/envelope biogenesis	Chitin synthase
EVM0009514	208.38	98.05	1.09	1.00E-04	7.96E-04	Carbohydrate transport and metabolism	Glyceraldehyde 3-phosphate dehydrogenase, C-terminal domain
EVM0008496	20.49	9.64	1.09	3.50E-04	2.36E-03	Amino acid transport and metabolism	Aminotransferase class-III
EVM0007972	6.27	2.94	1.09	1.30E-03	7.10E-03	RNA processing and modification	RNA dependent RNA polymerase
EVM0006573	20.01	9.38	1.09	4.80E-03	2.08E-02	--	--
EVM0008453	22.80	10.69	1.09	3.00E-04	2.07E-03	--	F-box-like
EVM0009159	7.09	3.32	1.09	9.50E-04	5.46E-03	--	--
EVM0004585	49.29	23.09	1.09	5.00E-05	4.28E-04	General function prediction only	GMC oxidoreductase
EVM0001289	91.63	42.81	1.10	5.00E-05	4.28E-04	--	--
EVM0011598	38.71	18.09	1.10	1.00E-04	7.96E-04	--	--
EVM0008097	90.78	42.37	1.10	2.25E-03	1.12E-02	--	--
EVM0004383	1175.83	548.42	1.10	1.09E-02	4.01E-02	--	Pro-kumamolisin, activation domain
EVM0006210	17.73	8.26	1.10	2.00E-04	1.46E-03	--	Nuclear condensing complex subunits, C-term domain
EVM0000711	119.23	55.45	1.10	1.50E-04	1.14E-03	--	Cytochrome P450

EVM0002430	746.46	347.11	1.10		5.00E-04	3.19E-03	Energy production and conversion	Dehydrogenase E1 component
EVM0000585	118.50	55.07	1.11		5.00E-05	4.28E-04	Secondary metabolites biosynthesis, transport and catabolism	Cytochrome P450
EVM0011276	131.05	60.82	1.11		5.00E-05	4.28E-04	Translation, ribosomal structure and biogenesis	Pumilio-family RNA binding repeat
EVM0000788	40.74	18.87	1.11		9.00E-04	5.22E-03	--	PH domain
EVM0006726	302.99	140.31	1.11		3.50E-04	2.36E-03	Posttranslational modification, protein turnover, chaperones	ATPase family associated with various cellular activities (AAA)
EVM0001345	24.42	11.26	1.12		5.00E-05	4.28E-04	Cell cycle control, cell division, chromosome partitioning	SIT4 phosphatase-associated protein
EVM0000598	87.02	40.01	1.12		5.00E-05	4.28E-04	Cell cycle control, cell division, chromosome partitioning	N terminus of Rad21 / Rec8 like protein
EVM0000295	719.86	331.02	1.12		1.40E-03	7.55E-03	Inorganic ion transport and metabolism	Catalase
EVM0004423	50.49	23.21	1.12		2.05E-03	1.03E-02	Posttranslational modification, protein turnover, chaperones	Peptidase S24-like
EVM0010842	132.66	60.94	1.12		9.00E-04	5.22E-03	--	Allophanate hydrolase subunit 2
EVM0006789	107.49	44.24	1.28		5.00E-05	4.28E-04	Posttranslational modification, protein turnover, chaperones	Eukaryotic aspartyl protease

EVM0002080	3.84	1.75	1.13	8.15E-03	3.18E-02	--	--
EVM0002999	48.51	22.10	1.13	1.00E-04	7.96E-04	Intracellular trafficking, secretion, and vesicular transport	ATG C terminal domain
EVM0009169	97.58	44.41	1.14	5.50E-04	3.46E-03	--	--
EVM0008682	47.42	21.57	1.14	1.00E-04	7.96E-04	General function prediction only	EamA-like transporter family
EVM0007345	174.29	79.23	1.14	1.00E-04	7.96E-04	Amino acid transport and metabolism	POT family
EVM0007019	151.81	69.00	1.14	1.00E-04	7.96E-04	--	Dynamin central region
EVM0007035	54.56	24.77	1.14	5.00E-05	4.28E-04	--	Transferrin receptor-like dimerisation domain
EVM0009222	12.54	5.68	1.14	6.00E-04	3.72E-03	--	Uncharacterized protein conserved in bacteria (DUF2264)
EVM0005286	403.44	182.66	1.14	5.00E-05	4.28E-04	--	--
EVM0002962	1185.33	536.59	1.14	7.50E-04	4.49E-03	--	--
EVM0009044	73.68	33.29	1.15	1.00E-04	7.96E-04	--	--
EVM0009274	27.59	12.46	1.15	5.00E-04	3.19E-03	--	CLASP N terminal
EVM0001663	2.75	1.24	1.15	1.27E-02	4.52E-02	Energy production and conversion	Pyridine nucleotide-disulphide oxidoreductase
EVM0002782	27.83	12.54	1.15	6.50E-04	3.98E-03	--	--
EVM0011907	18.74	8.44	1.15	1.00E-03	5.70E-03	Coenzyme transport and metabolism	Polyprenyl synthetase
EVM0000723	29.96	13.48	1.15	5.00E-05	4.28E-04	Translation, ribosomal structure and biogenesis	tRNA synthetases class I (I, L, M and V)

EVM0007128	179.99	80.68	1.16	3.00E-04	2.07E-03	Posttranslational modification, protein turnover, chaperones	ATPase family associated with various cellular activities (AAA)
EVM0005320	147.56	66.13	1.16	5.00E-05	4.28E-04	General function prediction only	Carbamoyl-phosphate synthase L chain, ATP binding domain
EVM0000040	48.39	21.67	1.16	1.00E-04	7.96E-04	Secondary metabolites biosynthesis, transport and catabolism	Flavin-binding monooxygenase-like
EVM0002137	55.49	24.81	1.16	1.00E-03	5.70E-03	Defense mechanisms	D-arabinono-1,4-lactone oxidase
EVM0010128	15.48	6.92	1.16	1.35E-03	7.33E-03	--	--
EVM0011002	21.80	9.74	1.16	5.00E-05	4.28E-04	Function unknown	Domain of unknown function (DUF3337)
EVM0003794	13.24	5.91	1.16	1.50E-04	1.14E-03	--	Calpain large subunit, domain III
EVM0006627	16.14	7.20	1.16	1.50E-04	1.14E-03	Chromatin structure and dynamics	Chromatin assembly factor 1 subunit A
EVM0010568	2.94	1.31	1.16	1.03E-02	3.82E-02	General function prediction only	Histidine phosphatase superfamily (branch 2)
EVM0008131	146.03	65.12	1.17	5.00E-05	4.28E-04	--	Cellulase (glycosyl hydrolase family 5)
EVM0006508	15.87	7.08	1.17	5.00E-05	4.28E-04	Function unknown	SprT-like family
EVM0007516	92.79	41.34	1.17	1.00E-04	7.96E-04	Carbohydrate transport and metabolism	Glycosyl hydrolases family 38 N-terminal domain
EVM0004887	2.66	1.18	1.17	4.90E-03	2.12E-02	--	Protein of unknown function (DUF4246)
EVM0006505	35.79	15.92	1.17	5.00E-05	4.28E-04	Signal transduction mechanisms	OPT oligopeptide transporter protein

EVM0007102	67.72	30.10	1.17	5.00E-04	3.19E-03	--	--
EVM0005016	63.79	28.35	1.17	1.00E-04	7.96E-04	Secondary metabolites biosynthesis, transport and catabolism	Copper amine oxidase, enzyme domain
EVM0010042	14.55	6.46	1.17	3.50E-04	2.36E-03	Transcription	RNA polymerase Rpb5, C-terminal domain
EVM0001686	2.98	1.32	1.17	5.60E-03	2.36E-02	--	Bestrophin, RFP-TM, chloride channel
EVM0000127	2964.39	1313.97	1.17	5.75E-03	2.41E-02	--	--
EVM0010882	230.74	102.20	1.17	5.00E-05	4.28E-04	General function prediction only	Histidine phosphatase superfamily (branch 2)
EVM0011238	83.17	36.82	1.18	1.00E-04	7.96E-04	Secondary metabolites biosynthesis, transport and catabolism	Cytochrome P450
EVM0003998	26.33	11.65	1.18	8.50E-04	4.98E-03	Posttranslational modification, protein turnover, chaperones	Proteasome activator pa28 beta subunit
EVM0009431	177.84	78.62	1.18	1.00E-04	7.96E-04	--	Mycolic acid cyclopropane synthetase
EVM0006594	269.00	118.87	1.18	2.00E-04	1.46E-03	Amino acid transport and metabolism	Serine hydroxymethyltransferase
EVM0010136	49.72	21.94	1.18	5.00E-05	4.28E-04	Carbohydrate transport and metabolism	Major Facilitator Superfamily
EVM0007160	5.87	2.59	1.18	6.50E-04	3.98E-03	--	--
EVM0001971	11.67	5.14	1.18	4.70E-03	2.05E-02	--	--

EVM0006450	87.10	38.33	1.18	5.00E-05	4.28E-04	General function prediction only	Alpha/beta hydrolase family
EVM0009661	305.78	134.20	1.19	5.00E-05	4.28E-04	--	--
EVM0004320	141.78	62.00	1.19	1.00E-04	7.96E-04	General function prediction only	Aldo/keto reductase family
EVM0002952	34.73	15.18	1.19	5.00E-05	4.28E-04	Extracellular structures	Tropomyosin
EVM0007366	42.46	18.53	1.20	5.00E-05	4.28E-04	--	--
EVM0003280	11.43	4.98	1.20	6.15E-03	2.54E-02	--	--
EVM0011387	65.19	28.38	1.20	5.00E-05	4.28E-04	Translation, ribosomal structure and biogenesis	tRNA synthetases class I (E and Q), catalytic domain
EVM0010099	308.50	134.30	1.20	1.00E-04	7.96E-04	--	Mycolic acid cyclopropane synthetase
EVM0003016	27.75	12.08	1.20	5.00E-05	4.28E-04	--	--
EVM0008931	53.49	23.27	1.20	5.00E-05	4.28E-04	--	Growth-Arrest-Specific Protein 2 Domain
EVM0011910	45.62	19.83	1.20	5.00E-05	4.28E-04	Intracellular trafficking, secretion, and vesicular transport	VID27 cytoplasmic protein
EVM0004174	37.21	16.16	1.20	5.00E-05	4.28E-04	Intracellular trafficking, secretion, and vesicular transport	COG4 transport protein
EVM0007504	3.76	1.63	1.20	6.40E-03	2.62E-02	--	--
EVM0002032	10.47	4.54	1.21	4.50E-04	2.92E-03	--	--
EVM0005679	23.45	10.16	1.21	3.00E-04	2.07E-03	--	--
EVM0006489	251.65	108.98	1.21	5.00E-05	4.28E-04	--	--
EVM0009273	3.79	1.64	1.21	1.65E-03	8.65E-03	--	--

EVM0002025	65.69	28.42	1.21	5.00E-05	4.28E-04	--	Major Facilitator Superfamily
EVM0001810	290.27	124.20	1.22	1.50E-04	1.14E-03	Carbohydrate transport and metabolism	Major intrinsic protein
EVM0010686	5.29	2.29	1.21	6.45E-03	2.64E-02	--	--
EVM0011240	257.40	111.20	1.21	1.00E-04	7.96E-04	--	--
EVM0007091	7.24	3.13	1.21	1.11E-02	4.06E-02	--	--
EVM0008774	23.46	10.13	1.21	5.00E-05	4.28E-04	Chromatin structure and dynamics	DNA gyrase/topoisomerase IV, subunit A
EVM0005574	19.45	8.39	1.21	5.00E-05	4.28E-04	RNA processing and modification	RNA dependent RNA polymerase
EVM0010980	1.45	0.63	1.21	1.65E-03	8.65E-03	--	--
EVM0009229	27.55	11.87	1.21	1.00E-04	7.96E-04	--	--
EVM0003903	18.22	7.84	1.22	5.00E-05	4.28E-04	--	--
EVM0004640	6.79	2.92	1.22	1.12E-02	4.10E-02	Signal transduction mechanisms	Phosphatidylinositolglycan class N (PIG-N)
EVM0001919	27.55	11.81	1.22	5.00E-05	4.28E-04	Replication, recombination and repair	DNA polymerase family B
EVM0005755	76.65	32.65	1.23	5.00E-05	4.28E-04	--	Glycosyl hydrolases family 16
EVM0010247	33.91	14.40	1.24	1.00E-04	7.96E-04	Secondary metabolites biosynthesis, transport and catabolism	Cytochrome P450
EVM0003492	87.17	37.01	1.24	5.00E-05	4.28E-04	--	Phospholipid methyltransferase
EVM0000868	27.85	11.81	1.24	5.00E-05	4.28E-04	--	Aconitase family (aconitate hydratase)
EVM0008035	27.70	11.75	1.24	5.00E-05	4.28E-04	General function prediction only	SNF2 family N-terminal domain

EVM0006748	105.07	44.52	1.24	5.00E-05	4.28E-04	Function unknown	TPR/MLP1/MLP2-like protein
EVM0011982	81.83	34.60	1.24	4.80E-03	2.08E-02	Chromatin structure and dynamics	Core histone H2A/H2B/H3/H4
EVM0008430	30.04	12.70	1.24	5.00E-05	4.28E-04	Replication, recombination and repair	DNA polymerase family B
EVM0002582	321.38	135.71	1.24	2.00E-04	1.46E-03	Translation, ribosomal structure and biogenesis	RNB domain
EVM0004575	99.93	42.19	1.24	2.00E-04	1.46E-03	General function prediction only	GMC oxidoreductase
EVM0005629	74.51	31.40	1.25	1.00E-04	7.96E-04	Translation, ribosomal structure and biogenesis	Ribosomal protein L7/L12 C-terminal domain
EVM0001758	21.48	9.01	1.25	7.00E-04	4.23E-03	--	--
EVM0010197	51.64	21.63	1.26	5.00E-05	4.28E-04	Secondary metabolites biosynthesis, transport and catabolism	ABC transporter transmembrane region
EVM0000333	20.83	8.72	1.26	5.00E-05	4.28E-04	Posttranslational modification, protein turnover, chaperones	Poly(ADP-ribose) polymerase catalytic domain
EVM0005868	145.04	60.61	1.26	1.50E-04	1.14E-03	--	--
EVM0008160	59.02	24.64	1.26	2.50E-04	1.77E-03	General function prediction only	RNA recognition motif. (a.k.a. RRM, RBD, or RNP domain)
EVM0005521	102.86	42.74	1.27	1.50E-04	1.14E-03	--	GPR1/FUN34/yaaH family
EVM0000497	2.92	1.21	1.27	1.85E-03	9.52E-03	--	--
EVM0011974	100.91	41.92	1.27	1.00E-04	7.96E-04	Cell cycle control, cell division, chromosome partitioning	Protein kinase domain

EVM0011018	42.35	17.58	1.27	2.00E-04	1.46E-03	Replication, recombination and repair	Rad51
EVM0001888	8.68	3.60	1.27	2.90E-03	1.38E-02	--	--
EVM0009544	81.36	33.60	1.28	5.00E-05	4.28E-04	Posttranslational modification, protein turnover, chaperones	IBR domain
EVM0006727	42.96	17.73	1.28	1.50E-04	1.14E-03	--	--
EVM0001477	438.78	180.80	1.28	5.00E-05	4.28E-04	--	Sulfatase-modifying factor enzyme 1
EVM0007667	162.90	67.05	1.28	5.00E-05	4.28E-04	--	Ubiquitin-2 like Rad60 SUMO-like
EVM0004127	16.42	6.74	1.28	1.00E-04	7.96E-04	Replication, recombination and repair	Endonuclease/Exonuclease/phosphatase family
EVM0001877	6.85	2.81	1.28	6.50E-04	3.98E-03	--	Voltage-dependent anion channel
EVM0011646	71.98	29.48	1.29	1.00E-04	7.96E-04	--	Pheromone A receptor
EVM0005980	46.60	19.03	1.29	5.00E-05	4.28E-04	General function prediction only	Animal haem peroxidase
EVM0010853	16.98	6.93	1.29	2.65E-03	1.28E-02	--	Flavin reductase like domain
EVM0008071	89.98	36.64	1.30	5.00E-05	4.28E-04	--	--
EVM0002498	116.02	47.08	1.30	5.00E-05	4.28E-04	Transcription	GATA zinc finger
EVM0001706	68.83	27.92	1.30	5.00E-05	4.28E-04	--	Oxidoreductase family, NAD-binding Rossmann fold
EVM0000398	38.60	15.62	1.31	5.00E-05	4.28E-04	Replication, recombination and repair	ATP dependent DNA ligase domain

EVM0010144	314.62	127.14	1.31	4.00E-04	2.65E-03	Posttranslational modification, protein turnover, chaperones	Ubiquitin carboxyl-terminal hydrolase
EVM0011123	62.48	25.21	1.31	5.00E-05	4.28E-04	Posttranslational modification, protein turnover, chaperones	Insulinase (Peptidase family M16)
EVM0008486	250.52	100.93	1.31	5.00E-05	4.28E-04	--	HEAT-like repeat
EVM0004840	490.75	197.64	1.31	5.00E-05	4.28E-04	--	--
EVM0004126	24.66	9.93	1.31	5.00E-05	4.28E-04	Cell cycle control, cell division, chromosome partitioning	RecF/RecN/SMC N terminal domain
EVM0010045	67.34	27.03	1.32	5.00E-05	4.28E-04	General function prediction only	Homeobox domain
EVM0002944	23.42	9.39	1.32	5.00E-05	4.28E-04	--	WD domain, G-beta repeat
EVM0007446	1162.29	466.01	1.32	1.25E-03	6.87E-03	--	Fungalysin metallopeptidase (M36)
EVM0001672	13.37	5.35	1.32	5.00E-04	3.19E-03	Secondary metabolites biosynthesis, transport and catabolism	Alcohol dehydrogenase GroES-like domain
EVM0004326	272.02	108.53	1.33	5.00E-05	4.28E-04	Posttranslational modification, protein turnover, chaperones	Eukaryotic aspartyl protease
EVM0006048	32.20	12.83	1.33	1.50E-04	1.14E-03	Function unknown	GINS complex protein
EVM0003051	99.99	39.84	1.33	5.00E-05	4.28E-04	--	--

EVM0001405	143.68	57.08	1.33	5.00E-05	4.28E-04	Intracellular trafficking, secretion, and vesicular transport	Sybindin-like family
EVM0007927	32.12	12.72	1.34	5.00E-05	4.28E-04	--	Condensin complex subunit 2
EVM0000862	30.14	11.93	1.34	5.00E-05	4.28E-04	Replication, recombination and repair	MCM2/3/5 family
EVM0008720	251.03	99.23	1.34	5.00E-05	4.28E-04	Nucleotide transport and metabolism	Ribonucleotide reductase, barrel domain
EVM0002963	54.85	21.60	1.34	5.00E-05	4.28E-04	--	--
EVM0001687	17.05	6.70	1.35	5.00E-05	4.28E-04	--	RecF/RecN/SMC N terminal domain
EVM0009963	1834.71	720.70	1.35	5.00E-05	4.28E-04	--	--
EVM0010285	15.43	6.05	1.35	5.00E-05	4.28E-04	--	Domain of unknown function (DUF1917)
EVM0004564	477.29	186.20	1.36	5.00E-05	4.28E-04	Lipid transport and metabolism	AMP-binding enzyme
EVM0007248	863.33	336.68	1.36	4.50E-04	2.92E-03	--	RNA recognition motif. (a.k.a. RRM, RBD, or RNP domain)
EVM0008886	58.76	22.90	1.36	5.00E-05	4.28E-04	--	SAD/SRA domain
EVM0006868	746.83	290.39	1.36	5.00E-05	4.28E-04	General function prediction only	RNA recognition motif. (a.k.a. RRM, RBD, or RNP domain)
EVM0002629	45.92	17.85	1.36	5.00E-05	4.28E-04	--	FAD binding domain
EVM0002732	51.09	19.86	1.36	2.00E-04	1.46E-03	Function unknown	Cysteine-rich secretory protein family
EVM0011327	15.28	5.94	1.36	9.50E-04	5.46E-03	Replication, recombination and repair	AAA domain

EVM0011859	10.80	4.18	1.37	2.50E-04	1.77E-03	Energy production and conversion	Phosphoenolpyruvate phosphomutase
EVM0000427	5.76	2.23	1.37	2.00E-04	1.46E-03	--	--
EVM0011640	67.98	26.22	1.37	1.00E-04	7.96E-04	--	--
EVM0011507	58.74	22.60	1.38	5.00E-05	4.28E-04	--	Nuclear pore component
EVM0005696	6.83	2.62	1.38	2.50E-03	1.22E-02	--	--
EVM0010293	28.32	10.86	1.38	5.00E-05	4.28E-04	Chromatin structure and dynamics	Histone deacetylase domain
EVM0009910	92.39	35.31	1.39	5.00E-05	4.28E-04	Cytoskeleton	Kinesin motor domain
EVM0010917	50.23	19.15	1.39	5.00E-05	4.28E-04	Defense mechanisms	Protein kinase domain
EVM0011948	45.34	17.28	1.39	1.00E-04	7.96E-04	Posttranslational modification, protein turnover, chaperones	Zinc knuckle
EVM0002530	4.23	1.61	1.39	6.00E-03	2.49E-02	--	50S ribosome-binding GTPase
EVM0000251	29.61	11.23	1.40	5.00E-05	4.28E-04	--	--
EVM0011035	2106.60	797.44	1.40	5.50E-04	3.46E-03	General function prediction only	RNA recognition motif. (a.k.a. RRM, RBD, or RNP domain)
EVM0011884	579.56	218.78	1.41	1.50E-04	1.14E-03	Signal transduction mechanisms	OPT oligopeptide transporter protein
EVM0009297	127.29	47.98	1.41	5.00E-05	4.28E-04	Amino acid transport and metabolism	Amino acid permease
EVM0005372	11.46	4.32	1.41	5.00E-04	3.19E-03	--	Alpha/beta hydrolase family
EVM0006268	78.69	29.59	1.41	5.00E-05	4.28E-04	--	--
EVM0007989	232.41	86.98	1.42	5.00E-05	4.28E-04	--	--
EVM0002464	88.49	33.10	1.42	5.00E-05	4.28E-04	Amino acid transport and metabolism	Aminotransferase class I and II

EVM0001436	64.87	24.25	1.42	5.00E-05	4.28E-04	--	WD domain, G-beta repeat
EVM0002514	86.13	32.19	1.42	5.00E-05	4.28E-04	--	Uncharacterised protein family (UPF0014)
EVM0001578	95.27	35.57	1.42	5.00E-05	4.28E-04	--	--
EVM0009234	46.42	17.32	1.42	5.00E-05	4.28E-04	--	Enolase C-terminal domain-like
EVM0009335	30.68	11.44	1.42	1.00E-04	7.96E-04	--	--
EVM0007131	10.81	4.01	1.43	1.25E-03	6.87E-03	--	--
EVM0010856	46.63	17.30	1.43	5.00E-05	4.28E-04	--	--
EVM0009427	12.77	4.73	1.43	1.50E-04	1.14E-03	--	Staphylococcal nuclease homologue
EVM0002441	17.79	6.58	1.43	1.00E-04	7.96E-04	General function prediction only	3'-5' exonuclease
EVM0000195	12.77	4.71	1.44	5.00E-05	4.28E-04	--	Serine carboxypeptidase S28
EVM0005872	495.49	182.71	1.44	5.00E-05	4.28E-04	--	--
EVM0004414	19.64	7.24	1.44	5.00E-05	4.28E-04	--	Polysaccharide deacetylase
EVM0002758	8.99	3.31	1.44	7.15E-03	2.86E-02	--	--
EVM0003073	9.86	3.63	1.44	9.50E-04	5.46E-03	--	--
EVM0003020	51.20	18.83	1.44	2.50E-04	1.77E-03	--	--
EVM0010940	3.68	1.35	1.45	9.95E-03	3.74E-02	--	--
EVM0007527	271.98	99.60	1.45	5.00E-05	4.28E-04	Intracellular trafficking, secretion, and vesicular transport	Region in Clathrin and VPS
EVM0009456	77.43	28.33	1.45	5.00E-05	4.28E-04	--	--
EVM0008782	21.38	7.82	1.45	5.00E-05	4.28E-04	Replication, recombination and repair	MCM2/3/5 family

EVM0007192	60.56	22.11	1.45	5.00E-05	4.28E-04	Cell cycle control, cell division, chromosome partitioning	Protein kinase domain
EVM0010231	58.52	21.28	1.46	5.00E-05	4.28E-04	--	NPL4 family
EVM0006788	199.54	72.05	1.47	5.00E-05	4.28E-04	--	--
EVM0003473	117.28	42.34	1.47	5.00E-05	4.28E-04	Amino acid transport and metabolism	POT family
EVM0006057	34.64	12.47	1.47	4.00E-04	2.65E-03	Intracellular trafficking, secretion, and vesicular transport	Clathrin adaptor complex small chain
EVM0000709	144.09	51.84	1.47	2.55E-03	1.24E-02	--	--
EVM0010611	153.17	54.99	1.48	5.00E-05	4.28E-04	--	Protein kinase domain
EVM0002114	223.28	80.09	1.48	5.00E-05	4.28E-04	Posttranslational modification, protein turnover, chaperones	Proteasome/cyclosome repeat
EVM0003304	6.83	2.44	1.48	5.40E-03	2.29E-02	Secondary metabolites biosynthesis, transport and catabolism	Cytochrome P450
EVM0011756	321.37	114.78	1.49	5.00E-05	4.28E-04	--	--
EVM0002138	123.74	44.14	1.49	1.00E-04	7.96E-04	--	--
EVM0002687	21.60	7.66	1.50	5.30E-03	2.26E-02	Transcription	HSF-type DNA-binding
EVM0005761	33.73	11.95	1.50	5.00E-05	4.28E-04	--	MRC1-like domain
EVM0004822	75.84	26.78	1.50	5.00E-05	4.28E-04	--	--
EVM0007451	105.78	37.32	1.50	5.00E-05	4.28E-04	--	Beta-glucan synthesis-associated protein (SKN1)
EVM0006103	107.14	37.75	1.50	5.00E-05	4.28E-04	--	--

EVM0000412	141.15	49.65	1.51	5.00E-05	4.28E-04	--	Thiamine pyrophosphate enzyme, N-terminal TPP binding domain
EVM0005905	24.21	8.51	1.51	5.00E-05	4.28E-04	Signal transduction mechanisms	Protein kinase domain
EVM0001718	10.11	3.54	1.51	1.00E-04	7.96E-04	--	--
EVM0011525	154.40	54.08	1.51	5.00E-05	4.28E-04	Cell cycle control, cell division, chromosome partitioning	Cyclin, N-terminal domain
EVM0002121	25.61	8.95	1.52	5.00E-05	4.28E-04	Replication, recombination and repair	AAA domain
EVM0003122	41.25	14.40	1.52	5.00E-05	4.28E-04	Translation, ribosomal structure and biogenesis	Domain of unknown function (DUF3554)
EVM0005845	45.97	16.02	1.52	5.00E-05	4.28E-04	--	Domain of unknown function (DUF4470)
EVM0000372	2.12	0.74	1.52	1.20E-02	4.33E-02	Posttranslational modification, protein turnover, chaperones	Eukaryotic aspartyl protease
EVM0005118	57.35	19.84	1.53	5.00E-05	4.28E-04	--	Urease alpha-subunit, N-terminal domain
EVM0005203	45.67	15.76	1.54	5.00E-05	4.28E-04	Intracellular trafficking, secretion, and vesicular transport	Pep3/Vps18/deep orange family
EVM0008335	59.07	20.26	1.54	5.00E-05	4.28E-04	Function unknown	Protein of unknown function (DUF3414)
EVM0009751	2.19	0.75	1.54	2.00E-04	1.46E-03	--	--
EVM0002937	148.90	50.94	1.55	5.00E-05	4.28E-04	--	--

EVM0000347	285.86	97.62	1.55	5.00E-05	4.28E-04	--	--
EVM0010784	22.61	7.72	1.55	5.00E-05	4.28E-04	--	--
EVM0000530	33.76	11.51	1.55	5.00E-05	4.28E-04	Function unknown	Ribosome 60S biogenesis N-terminal
EVM0010721	5.31	1.81	1.56	8.55E-03	3.31E-02	--	--
EVM0007836	8.30	2.82	1.56	8.50E-04	4.98E-03	--	--
EVM0008522	63.57	21.55	1.56	5.00E-05	4.28E-04	Energy production and conversion	FAD dependent oxidoreductase
EVM0006409	9.70	3.28	1.56	5.00E-05	4.28E-04	--	HNH endonuclease
EVM0002986	66.42	22.45	1.56	5.00E-05	4.28E-04	Cytoskeleton	Kinesin motor domain
EVM0000884	165.55	55.95	1.57	5.00E-05	4.28E-04	Lipid transport and metabolism	AMP-binding enzyme
EVM0011719	8.28	2.80	1.57	5.00E-05	4.28E-04	General function prediction only	WD domain, G-beta repeat
EVM0006669	251.79	84.91	1.57	5.00E-05	4.28E-04	General function prediction only	HMG (high mobility group) box
EVM0011466	33.08	11.14	1.57	5.00E-05	4.28E-04	--	--
EVM0012194	158.51	53.35	1.57	5.00E-05	4.28E-04	--	Amidohydrolase
EVM0008994	2.48	0.83	1.57	4.90E-03	2.12E-02	--	--
EVM0008059	238.02	79.59	1.58	5.00E-05	4.28E-04	Carbohydrate transport and metabolism	Domain of unknown function (DUF1793)
EVM0010213	7709.96	2576.26	1.58	2.55E-03	1.24E-02	--	--
EVM0001466	13.98	4.67	1.58	5.00E-05	4.28E-04	Posttranslational modification, protein turnover, chaperones	Peptidase family M48

EVM0001750	10.95	3.65	1.58	5.00E-05	4.28E-04	Amino acid transport and metabolism	Amino acid permease
EVM0010994	105.60	35.10	1.59	5.00E-05	4.28E-04	--	Pro-kumamolisin, activation domain
EVM0005775	30.09	10.00	1.59	5.00E-05	4.28E-04	--	FHA domain
EVM0011821	710.80	235.83	1.59	5.00E-05	4.28E-04	--	Glycosyl hydrolase family 71
EVM0007336	235.59	78.15	1.59	5.00E-05	4.28E-04	Posttranslational modification, protein turnover, chaperones	AhpC/TSA family
EVM0001317	30.70	10.10	1.60	5.00E-05	4.28E-04	--	SNF2 family N-terminal domain
EVM0006660	601.05	197.09	1.61	5.00E-05	4.28E-04	--	--
EVM0002736	103.75	34.02	1.61	5.00E-05	4.28E-04	Cytoskeleton	Myosin head (motor domain)
EVM0006506	20.27	6.61	1.62	8.00E-04	4.73E-03	#N/A	#N/A
EVM0004599	29.91	9.75	1.62	5.00E-05	4.28E-04	Lipid transport and metabolism	Phosphatidylserine decarboxylase
EVM0011882	66.19	21.58	1.62	5.00E-05	4.28E-04	Cytoskeleton	Variant SH3 domain
EVM0007579	196.92	63.66	1.63	5.00E-05	4.28E-04	--	HEAT repeat
EVM0002904	103.97	33.61	1.63	5.00E-05	4.28E-04	--	--
EVM0003583	661.53	213.60	1.63	2.00E-04	1.46E-03	--	--
EVM0005111	1740.83	561.42	1.63	2.00E-04	1.46E-03	--	Cellulase (glycosyl hydrolase family 5)
EVM0008739	374.83	120.64	1.64	5.00E-05	4.28E-04	Energy production and conversion	Carbamoyl-phosphate synthase L chain, ATP binding domain
EVM0012202	1206.18	388.20	1.64	5.00E-05	4.28E-04	--	--
EVM0009418	62.33	20.04	1.64	5.00E-05	4.28E-04	--	--
EVM0006985	51.97	16.63	1.64	5.00E-05	4.28E-04	--	--

EVM0004642	65.86	21.05	1.65	5.00E-05	4.28E-04	--	Diaphanous GTPase-binding Domain
EVM0010778	10.98	3.50	1.65	5.00E-05	4.28E-04	--	SNF2 family N-terminal domain
EVM0000921	7.20	2.29	1.65	5.00E-04	3.19E-03	--	--
EVM0010470	198.15	62.94	1.65	5.00E-05	4.28E-04	Posttranslational modification, protein turnover, chaperones	Proteasome/cyclosome repeat
EVM0008259	791.35	251.28	1.66	5.00E-05	4.28E-04	--	Putative oxidoreductase C terminal
EVM0000202	148.92	47.18	1.66	5.00E-05	4.28E-04	--	Glycosyl transferase family group 2
EVM0002727	1259.94	397.01	1.67	5.00E-05	4.28E-04	--	Cupin
EVM0007570	26.15	8.24	1.67	5.00E-05	4.28E-04	--	Poly(ADP-ribose) polymerase catalytic domain
EVM0005588	55.71	17.53	1.67	5.00E-05	4.28E-04	Function unknown	Cysteine-rich secretory protein family
EVM0001424	2085.62	648.00	1.69	5.00E-05	4.28E-04	Cytoskeleton	Dynein light chain type 1
EVM0009616	46.58	14.45	1.69	2.00E-04	1.46E-03	--	Ribosomal protein S21
EVM0005530	40.54	12.54	1.69	5.00E-05	4.28E-04	--	XFP N-terminal domain
EVM0008321	260.36	80.37	1.70	5.00E-05	4.28E-04	General function prediction only	Major Facilitator Superfamily
EVM0006122	32.58	10.04	1.70	6.20E-03	2.56E-02	--	--
EVM0000617	32.73	10.08	1.70	5.00E-05	4.28E-04	Cytoskeleton	Kinesin motor domain
EVM0004411	24.56	7.56	1.70	5.00E-05	4.28E-04	--	Spc7 kinetochore protein
EVM0002125	8.30	2.55	1.71	5.00E-05	4.28E-04	General function prediction only	Major Facilitator Superfamily

EVM0004972	13.31	4.06	1.71	5.00E-05	4.28E-04	--	--
EVM0011205	378.12	115.24	1.71	5.00E-05	4.28E-04	--	Glycosyl hydrolase catalytic core
EVM0007580	380.76	115.15	1.73	5.00E-05	4.28E-04	Cell wall/membrane/envelope biogenesis	Glycosyl transferases group 1
EVM0011006	4.98	1.50	1.73	5.00E-05	4.28E-04	--	Protein of unknown function (DUF1524)
EVM0002859	52.56	15.76	1.74	5.00E-05	4.28E-04	--	non-SMC mitotic condensation complex subunit 1
EVM0008227	41.23	12.26	1.75	5.00E-05	4.28E-04	Replication, recombination and repair	MCM2/3/5 family
EVM0006381	33.01	9.81	1.75	5.00E-05	4.28E-04	Cell cycle control, cell division, chromosome partitioning	PIF1-like helicase
EVM0003976	145.42	43.18	1.75	5.00E-05	4.28E-04	Amino acid transport and metabolism	Conserved region in glutamate synthase
EVM0002409	30.62	9.02	1.76	5.00E-05	4.28E-04	--	--
EVM0004677	131.80	38.71	1.77	5.00E-05	4.28E-04	--	Serine carboxypeptidase S28
EVM0001331	27.81	8.17	1.77	5.00E-05	4.28E-04	--	Ankyrin repeats (many copies)
EVM0005614	92.97	27.28	1.77	5.00E-05	4.28E-04	--	--
EVM0011462	34.17	10.00	1.77	5.00E-05	4.28E-04	Intracellular trafficking, secretion, and vesicular transport	Exocyst complex subunit Sec15-like
EVM0008827	3.30	0.96	1.78	1.20E-02	4.32E-02	--	--
EVM0011687	37.08	10.72	1.79	5.00E-05	4.28E-04	Function unknown	Domain of unknown function (DUF3437)

EVM0008766	19.94	5.71	1.80	5.25E-03	2.24E-02	--	--
EVM0005006	71.38	20.25	1.82	5.00E-05	4.28E-04	--	--
EVM0010840	13.56	3.84	1.82	5.00E-05	4.28E-04	--	--
EVM0006629	43.15	12.20	1.82	5.00E-05	4.28E-04	Secondary metabolites biosynthesis, transport and catabolism	ABC transporter
EVM0008881	231.32	65.30	1.82	5.00E-05	4.28E-04	--	Glycosyl hydrolase family 3 N terminal domain
EVM0001947	5.52	1.55	1.83	2.25E-03	1.12E-02	--	--
EVM0008628	32.71	9.17	1.83	5.00E-05	4.28E-04	--	--
EVM0011867	37.29	10.42	1.84	5.00E-05	4.28E-04	General function prediction only	Haloacid dehalogenase-like hydrolase
EVM0003134	58.00	16.17	1.84	5.00E-05	4.28E-04	--	--
EVM0003929	11.97	3.34	1.84	5.00E-05	4.28E-04	--	Taurine catabolism dioxygenase TauD, TfdA family
EVM0004082	2.07	0.58	1.84	4.45E-03	1.96E-02	--	NADH:flavin oxidoreductase / NADH oxidase family
EVM0012261	7.22	2.00	1.85	5.00E-05	4.28E-04	Replication, recombination and repair	MutS domain V
EVM0005557	73.60	20.38	1.85	5.00E-05	4.28E-04	--	Nucleoporin subcomplex protein binding to Pom34
EVM0008413	62.43	17.20	1.86	5.00E-05	4.28E-04	General function prediction only	ABC1 family
EVM0009035	69.82	19.15	1.87	5.00E-05	4.28E-04	--	Non-repetitive/WGA-negative nucleoporin C-terminal
EVM0003987	25.53	7.00	1.87	6.15E-03	2.54E-02	--	--

EVM0005101	4.27	1.17	1.87	7.00E-04	4.23E-03	Replication, recombination and repair	HhH-GPD superfamily base excision DNA repair protein
EVM0006078	64.61	17.70	1.87	8.50E-04	4.98E-03	--	Cytochrome c oxidase assembly protein COX16
EVM0001207	16.38	4.45	1.88	1.50E-04	1.14E-03	--	--
EVM0001579	37.82	10.27	1.88	5.00E-05	4.28E-04	General function prediction only	Family of unknown function (DUF706)
EVM0007751	100.21	27.07	1.89	5.00E-05	4.28E-04	--	--
EVM0009974	84.98	22.85	1.90	5.00E-05	4.28E-04	Signal transduction mechanisms	GTPase-activator protein for Ras-like GTPase
EVM0004725	89.52	23.99	1.90	5.00E-05	4.28E-04	Chromatin structure and dynamics	linker histone H1 and H5 family
EVM0001562	143.45	38.42	1.90	5.00E-05	4.28E-04	--	Alpha amylase, catalytic domain
EVM0003796	20.10	5.38	1.90	5.00E-05	4.28E-04	--	--
EVM0005846	6.02	1.61	1.90	7.25E-03	2.90E-02	--	--
EVM0003022	7.83	2.09	1.91	5.00E-05	4.28E-04	Amino acid transport and metabolism	FAD dependent oxidoreductase
EVM0001015	8.14	2.17	1.91	5.00E-05	4.28E-04	--	--
EVM0005567	391.68	103.70	1.92	5.00E-05	4.28E-04	Nucleotide transport and metabolism	Ribonucleotide reductase, small chain
EVM0002796	90.71	23.78	1.93	5.00E-05	4.28E-04	--	Permease for cytosine/purines, uracil, thiamine, allantoin
EVM0007397	18.02	4.72	1.93	5.00E-05	4.28E-04	Function unknown	WD domain, G-beta repeat
EVM0010588	40.66	10.64	1.93	1.00E-04	7.96E-04	--	--
EVM0009073	1.86	0.48	1.94	1.10E-02	4.04E-02	--	--
EVM0001707	52.27	13.66	1.94	5.00E-05	4.28E-04	--	Nucleoporin Nup120/160

EVM0005021	6.93	1.79	1.95	5.00E-05	4.28E-04	--	--
EVM0008746	49.40	12.72	1.96	5.00E-05	4.28E-04	--	UvrD-like helicase C-terminal domain
EVM0000515	61.62	15.86	1.96	5.00E-05	4.28E-04	--	--
EVM0011435	56.24	14.19	1.99	5.00E-05	4.28E-04	Cytoskeleton	Myosin head (motor domain)
EVM0011675	37.64	9.43	2.00	5.00E-05	4.28E-04	Cytoskeleton	Dynein heavy chain and region D6 of dynein motor
EVM0009298	65.31	16.29	2.00	5.00E-05	4.28E-04	--	--
EVM0008341	53.66	13.36	2.01	7.00E-03	2.82E-02	--	Anaphase-promoting complex subunit 11 RING-H2 finger
EVM0003928	2.72	0.67	2.02	3.95E-03	1.77E-02	Carbohydrate transport and metabolism	Major Facilitator Superfamily
EVM0001737	64.51	15.72	2.04	5.00E-05	4.28E-04	Secondary metabolites biosynthesis, transport and catabolism	Cytochrome P450
EVM0006249	158.85	38.60	2.04	3.65E-03	1.66E-02	--	Predicted coiled-coil protein (DUF2205)
EVM0006340	78.81	19.07	2.05	5.00E-05	4.28E-04	--	Nup53/35/40-type RNA recognition motif
EVM0006417	2.33	0.56	2.06	4.95E-03	2.13E-02	--	--
EVM0006959	40.19	9.63	2.06	5.00E-05	4.28E-04	--	Cytochrome oxidase complex assembly protein 1
EVM0008978	23.38	5.54	2.08	5.00E-05	4.28E-04	Transcription	SNF2 family N-terminal domain
EVM0012190	3.47	0.82	2.09	5.00E-05	4.28E-04	Secondary metabolites biosynthesis, transport and catabolism	ABC transporter transmembrane region

EVM0008911	12.80	2.99	2.10	5.00E-05	4.28E-04	--	--
EVM0000478	45.23	10.54	2.10	5.00E-05	4.28E-04	Signal transduction mechanisms	Protein kinase domain
EVM0002287	93.86	21.71	2.11	3.25E-03	1.51E-02	--	Cytochrome P450
EVM0008140	48.80	11.29	2.11	5.00E-05	4.28E-04	--	--
EVM0002953	14.48	3.32	2.12	5.00E-05	4.28E-04	--	Fungalysin metallopeptidase (M36)
EVM0002414	53.22	12.09	2.14	5.00E-05	4.28E-04	--	--
EVM0006225	138.68	31.39	2.14	5.00E-05	4.28E-04	--	--
EVM0007573	5359.43	1206.28	2.15	5.00E-05	4.28E-04	--	Fungal hydrophobin
EVM0011658	7.13	1.60	2.15	2.00E-04	1.46E-03	Lipid transport and metabolism	Enoyl-CoA hydratase/isomerase family
EVM0004073	28.42	6.24	2.19	7.90E-03	3.10E-02	--	SCP-2 sterol transfer family
EVM0002281	68.12	14.92	2.19	5.00E-05	4.28E-04	--	--
EVM0001058	20.50	4.49	2.19	5.00E-05	4.28E-04	--	--
EVM0011790	49.97	10.85	2.20	8.90E-03	3.41E-02	General function prediction only	Ras family
EVM0011093	42.57	9.08	2.23	2.00E-03	1.01E-02	Intracellular trafficking, secretion, and vesicular transport	SNARE domain
EVM0009599	5.50	1.16	2.25	5.00E-05	4.28E-04	--	TPR repeat
EVM0006476	546.56	112.61	2.28	5.00E-05	4.28E-04	--	LVIVD repeat
EVM0010488	49.03	10.05	2.29	5.00E-05	4.28E-04	--	--
EVM0003467	597.63	121.97	2.29	5.00E-05	4.28E-04	--	--
EVM0000453	711.90	141.29	2.33	5.00E-05	4.28E-04	--	--
EVM0004723	16.45	3.12	2.40	4.00E-04	2.65E-03	--	--

EVM0007392	78.61	14.88	2.40	5.00E-05	4.28E-04	--	WSC domain
EVM0006479	40.01	7.56	2.40	5.00E-05	4.28E-04	--	RecF/RecN/SMC N terminal domain
EVM0007712	48.98	9.10	2.43	5.00E-05	4.28E-04	General function prediction only	Protein kinase domain
EVM0009049	16.16	3.00	2.43	5.00E-05	4.28E-04	--	--
EVM0007684	199.30	36.61	2.44	5.00E-05	4.28E-04	General function prediction only	Major Facilitator Superfamily
EVM0008898	1826.72	325.60	2.49	5.00E-05	4.28E-04	--	--
EVM0001811	1.24	0.21	2.53	1.13E-02	4.11E-02	General function prediction only	Major Facilitator Superfamily
EVM0010018	118.90	20.46	2.54	5.00E-05	4.28E-04	--	Fungal hydrophobin
EVM0007764	23.91	4.11	2.54	5.00E-05	4.28E-04	--	SNF2 family N-terminal domain
EVM0006645	485.89	82.89	2.55	5.00E-05	4.28E-04	--	--
EVM0009189	4.10	0.70	2.56	2.30E-03	1.14E-02	--	Pheromone A receptor
EVM0005744	12.23	2.07	2.56	5.00E-05	4.28E-04	RNA processing and modification	AAA domain
EVM0008758	1.28	0.21	2.58	1.75E-03	9.08E-03	--	--
EVM0005145	30.58	4.98	2.62	1.60E-03	8.44E-03	--	--
EVM0000235	173.82	28.32	2.62	5.00E-05	4.28E-04	--	Voltage-dependent anion channel
EVM0002747	1.62	0.25	2.67	2.00E-04	1.46E-03	RNA processing and modification	DEAD/DEAH box helicase
EVM0002674	9.74	1.52	2.68	5.00E-05	4.28E-04	Secondary metabolites biosynthesis, transport and catabolism	short chain dehydrogenase
EVM0002033	6.62	1.03	2.68	2.35E-03	1.16E-02	#N/A	#N/A

EVM0003743	7.10	1.10	2.69	5.00E-05	4.28E-04	Carbohydrate transport and metabolism	Major Facilitator Superfamily
EVM0008010	4.63	0.71	2.70	4.25E-03	1.89E-02	--	--
EVM0005169	1355.12	206.52	2.71	5.00E-05	4.28E-04	--	Peptidase family M28
EVM0000187	11.97	1.75	2.77	5.00E-05	4.28E-04	--	--
EVM0008755	63.02	8.99	2.81	5.00E-05	4.28E-04	--	Cupin domain
EVM0011914	35.25	4.96	2.83	5.00E-05	4.28E-04	--	Cerato-platanin
EVM0003089	18.34	2.50	2.87	1.00E-04	7.96E-04	General function prediction only	Uncharacterised protein family (UPF0172)
EVM0008112	670.65	90.26	2.89	5.00E-05	4.28E-04	Intracellular trafficking, secretion, and vesicular transport	ER lumen protein retaining receptor
EVM0011853	5.25	0.70	2.90	2.00E-04	1.46E-03	--	--
EVM0009981	3.71	0.49	2.91	4.50E-04	2.92E-03	--	--
EVM0003369	2.62	0.34	2.94	4.00E-04	2.65E-03	General function prediction only	GMC oxidoreductase
EVM0000318	5.06	0.63	3.01	2.45E-03	1.20E-02	--	--
EVM0000933	5.16	0.63	3.04	5.00E-05	4.28E-04	Energy production and conversion	Malic enzyme, NAD binding domain
EVM0000184	110.04	12.76	3.11	5.00E-05	4.28E-04	Signal transduction mechanisms	OPT oligopeptide transporter protein
EVM0002115	30.58	3.36	3.19	5.00E-05	4.28E-04	--	Pregnancy-associated plasma protein-A
EVM0008976	3.27	0.35	3.24	3.95E-03	1.77E-02	--	--
EVM0006874	30.80	2.54	3.60	5.00E-05	4.28E-04	--	--
EVM0009153	56.94	3.85	3.89	5.00E-05	4.28E-04	--	--

EVM0004142	1008.37	56.52	4.16		5.00E-05	4.28E-04	Intracellular trafficking, secretion, and vesicular transport	Vacuolar sorting protein 39 domain 1
EVM0001256	98.71	5.30	4.22		5.00E-05	4.28E-04	--	LVIVD repeat
EVM0007642	279.57	12.38	4.50		5.00E-05	4.28E-04	--	Pregnancy-associated plasma protein-A
EVM0010621	41.11	1.54	4.74		5.00E-05	4.28E-04	--	Deuterolysin metalloprotease (M35) family
EVM0008710	157.51	5.11	4.95		5.00E-05	4.28E-04	Posttranslational modification, protein turnover, chaperones	Subtilase family
EVM0007266	1958.60	24.78	6.30		5.00E-05	4.28E-04	--	--

Data Set S4 Information of DEGs in R2 vs Z2

gene ID	R2 value	Z2 value	log2(fold change)	p value	q value	KOG class	Pfam annotation
EVM0007042	54.21	1542.16	-4.83	5.00E-05	4.28E-04	--	Hemerythrin HHE cation binding domain
EVM0004233	14.27	375.05	-4.72	5.00E-05	4.28E-04	--	--
EVM0004083	1.94	48.99	-4.66	4.90E-03	2.12E-02	--	--
EVM0002148	32.23	669.23	-4.38	5.00E-05	4.28E-04	--	Uncharacterised protein family (UPF0197)
EVM0005865	229.42	3627.32	-3.98	5.00E-05	4.28E-04	General function prediction only	Proteolipid membrane potential modulator
EVM0003167	13.76	215.43	-3.97	5.00E-05	4.28E-04	--	--
EVM0012049	2.62	39.34	-3.91	5.00E-05	4.28E-04	--	--
EVM0001104	6.02	90.23	-3.91	5.00E-05	4.28E-04	--	--
EVM0011248	10.86	143.39	-3.72	5.00E-04	3.19E-03	--	--
EVM0001350	145.73	1864.80	-3.68	5.00E-05	4.28E-04	--	CsbD-like
EVM0006251	3.49	41.06	-3.56	5.00E-05	4.28E-04	Function unknown	Surfeit locus protein 6

EVM0004031	10.64	124.33	-3.55	5.00E-05	4.28E-04	RNA processing and modification	Eukaryotic rRNA processing protein EBP2
EVM0005648	0.36	4.23	-3.54	2.95E-03	1.40E-02	--	--
EVM0010264	19.41	215.88	-3.48	5.00E-05	4.28E-04	Function unknown	Thioesterase superfamily
EVM0009090	25.19	266.06	-3.40	5.00E-05	4.28E-04	--	--
EVM0002926	93.62	956.06	-3.35	5.00E-05	4.28E-04	General function prediction only	Enoyl-(Acyl carrier protein) reductase
EVM0008465	29.83	304.46	-3.35	5.00E-05	4.28E-04	RNA processing and modification	DEAD/DEAH box helicase
EVM0003732	0.71	7.19	-3.35	5.00E-05	4.28E-04	--	--
EVM0010786	12.59	126.97	-3.33	5.00E-05	4.28E-04	--	Zinc finger, C3HC4 type (RING finger)
EVM0010693	36.22	363.99	-3.33	5.00E-05	4.28E-04	--	--
EVM0009709	8.36	83.39	-3.32	5.00E-05	4.28E-04	--	--
EVM0001661	6.71	65.06	-3.28	5.00E-05	4.28E-04	General function prediction only	Kinase phosphorylation protein
EVM0002081	2.03	18.86	-3.21	5.00E-05	4.28E-04	Signal transduction mechanisms	FHA domain

EVM0010422	4.63	42.69	-3.21	5.00E-05	4.28E-04	Posttranslational modification, protein turnover, chaperones	DnaJ domain
EVM0005078	1.14	10.08	-3.14	5.00E-05	4.28E-04	Cell cycle control, cell division, chromosome partitioning	HORMA domain
EVM0008966	22.04	194.61	-3.14	5.00E-05	4.28E-04	--	--
EVM0005937	48.94	402.75	-3.04	5.00E-05	4.28E-04	--	--
EVM0002682	47.46	389.54	-3.04	5.00E-05	4.28E-04	Transcription	RNA polymerase Rpb6
EVM0005127	87.46	713.49	-3.03	5.00E-05	4.28E-04	--	Gamma interferon inducible lysosomal thiol reductase (GILT)
EVM0007507	13.98	112.47	-3.01	5.00E-05	4.28E-04	--	--
EVM0003543	45.44	365.30	-3.01	5.00E-05	4.28E-04	--	--
EVM0009623	26.53	208.31	-2.97	5.00E-05	4.28E-04	--	--
EVM0006981	15.64	122.70	-2.97	5.00E-05	4.28E-04	--	Rrp15p
EVM0011068	37.34	291.05	-2.96	5.00E-05	4.28E-04	Defense mechanisms	Dual specificity phosphatase, catalytic domain
EVM0007225	74.26	570.70	-2.94	5.00E-05	4.28E-04	--	Nuclear pore complex subunit Nro1

EVM0011230	12.94	98.61	-2.93	5.00E-05	4.28E-04	--	SNARE associated Golgi protein
EVM0001863	0.24	1.78	-2.92	6.75E-03	2.74E-02	--	--
EVM0011786	10.99	81.86	-2.90	5.00E-05	4.28E-04	--	--
EVM0006622	6.66	49.23	-2.89	5.00E-05	4.28E-04	--	--
EVM0000282	157.30	1159.63	-2.88	5.00E-05	4.28E-04	--	--
EVM0004965	2.58	18.91	-2.88	5.00E-05	4.28E-04	Function unknown	Tho complex subunit 7
EVM0008830	59.83	434.89	-2.86	5.00E-05	4.28E-04	Defense mechanisms	Dual specificity phosphatase, catalytic domain
EVM0002451	2.84	20.22	-2.83	5.00E-05	4.28E-04	--	--
EVM0004346	363.40	2552.93	-2.81	5.00E-05	4.28E-04	--	--
EVM0005353	2.27	15.84	-2.80	5.00E-05	4.28E-04	Posttranslational modification, protein turnover, chaperones	Peptidase family M50
EVM0008889	0.27	1.84	-2.79	9.00E-04	5.22E-03	--	Cytochrome P450
EVM0006626	45.70	312.50	-2.77	5.00E-05	4.28E-04	--	--

EVM0002754	10.25	69.76	-2.77	5.00E-05	4.28E-04	General function prediction only	Ankyrin repeats (3 copies)
EVM0007670	22.98	153.28	-2.74	5.00E-05	4.28E-04	--	Domain of unknown function (DUF4112)
EVM0008891	7.44	49.34	-2.73	5.00E-05	4.28E-04	Amino acid transport and metabolism	Histidine biosynthesis protein
EVM0006174	4.08	26.94	-2.72	5.00E-05	4.28E-04	--	--
EVM0001964	7.02	46.30	-2.72	5.00E-05	4.28E-04	--	--
EVM0009876	8.31	54.78	-2.72	5.00E-05	4.28E-04	Translation, ribosomal structure and biogenesis	eRF1 domain 1
EVM0006028	88.99	581.35	-2.71	5.00E-05	4.28E-04	--	--
EVM0001246	8.49	55.45	-2.71	5.00E-05	4.28E-04	--	--
EVM0000819	3.21	20.74	-2.69	5.00E-05	4.28E-04	Transcription	SNF2 family N-terminal domain
EVM0008113	12.51	80.57	-2.69	5.00E-05	4.28E-04	--	FAD binding domain
EVM0003361	9.42	59.91	-2.67	5.00E-05	4.28E-04	--	Lipoxygenase
EVM0009807	1276.43	7974.27	-2.64	5.00E-05	4.28E-04	--	Mitochondrial F1-F0 ATP synthase subunit F of fungi
EVM0004307	0.69	4.27	-2.63	7.95E-03	3.12E-02	Carbohydrate transport and metabolism	Glycosyl hydrolase family 47

EVM0009589	23.21	143.12	-2.62	5.00E-05	4.28E-04	--	--
EVM0004635	75.47	464.42	-2.62	5.00E-05	4.28E-04	--	--
EVM0000142	4.07	25.04	-2.62	5.00E-05	4.28E-04	--	Putative phosphatase regulatory subunit
EVM0010870	14.22	87.23	-2.62	5.00E-05	4.28E-04	--	Conidiation protein 6
EVM0004767	103.26	633.44	-2.62	1.60E-03	8.44E-03	--	4F5 protein family
EVM0008562	142.27	865.07	-2.60	5.00E-05	4.28E-04	--	--
EVM0004053	97.38	589.80	-2.60	5.00E-05	4.28E-04	--	--
EVM0004085	4.00	24.19	-2.59	7.00E-04	4.23E-03	Posttranslational modification, protein turnover, chaperones	FKBP-type peptidyl-prolyl cis-trans isomerase
EVM0004297	21.23	128.12	-2.59	5.00E-05	4.28E-04	--	BTG family
EVM0011879	18.02	107.65	-2.58	5.00E-05	4.28E-04	General function prediction only	--
EVM0007146	732.45	4368.04	-2.58	5.00E-05	4.28E-04	--	--
EVM0006195	13.98	82.97	-2.57	5.00E-05	4.28E-04	--	pre-mRNA splicing factor component

EVM0009852	10.78	63.41	-2.56	5.00E-05	4.28E-04	Transcription	Homeobox domain
EVM0004221	6.81	40.02	-2.56	5.00E-05	4.28E-04	--	--
EVM0000467	2.39	14.03	-2.55	5.00E-05	4.28E-04	--	--
EVM0006068	2.92	17.08	-2.55	5.00E-05	4.28E-04	--	bZIP transcription factor
EVM0003318	27.53	159.23	-2.53	5.00E-05	4.28E-04	--	Autophagy protein 16 (ATG16)
EVM0008586	12.87	74.08	-2.53	5.00E-05	4.28E-04	--	Chromatin remodelling complex Rsc7/Swp82 subunit
EVM0003528	80.61	456.82	-2.50	5.00E-05	4.28E-04	--	--
EVM0011013	11.45	64.79	-2.50	3.60E-03	1.64E-02	Function unknown	COQ9
EVM0005600	41.83	236.25	-2.50	5.00E-05	4.28E-04	--	Acetokinase family
EVM0007894	2.34	13.22	-2.50	7.50E-04	4.49E-03	General function prediction only	Zinc finger, C2H2 type
EVM0001814	5417.96	30257.30	-2.48	5.00E-05	4.28E-04	--	Ricin-type beta-trefoil lectin domain-like
EVM0002391	207.59	1159.11	-2.48	5.00E-05	4.28E-04	--	--
EVM0003241	30.80	171.23	-2.47	5.00E-05	4.28E-04	--	Helix-loop-helix DNA-binding domain

EVM0004958	9.86	54.76	-2.47	5.00E-05	4.28E-04	--	Regulator of G protein signaling domain
EVM0009404	12.49	69.28	-2.47	5.00E-05	4.28E-04	General function prediction only	Dip2/Utp12 Family
EVM0001738	348.42	1928.15	-2.47	5.00E-05	4.28E-04	Lipid transport and metabolism	Phophatidylserine decarboxylase
EVM0009103	5.56	30.53	-2.46	5.00E-05	4.28E-04	--	Pheromone A receptor
EVM0006335	68.22	372.66	-2.45	5.00E-05	4.28E-04	--	--
EVM0001319	2.47	13.51	-2.45	5.00E-05	4.28E-04	--	--
EVM0005139	160.92	877.27	-2.45	5.00E-05	4.28E-04	--	--
EVM0000151	186.42	1015.80	-2.45	5.00E-05	4.28E-04	Lipid transport and metabolism	Cytochrome b5-like Heme/Steroid binding domain
EVM0007132	0.60	3.26	-2.45	5.00E-05	4.28E-04	--	--
EVM0000062	20.77	113.10	-2.45	5.00E-05	4.28E-04	--	--
EVM0011432	9.06	49.27	-2.44	5.00E-05	4.28E-04	Lipid transport and metabolism	SacI homology domain
EVM0003828	9.87	53.54	-2.44	5.00E-05	4.28E-04	--	Membrane bound O-acyl transferase family
EVM0005254	26.67	144.42	-2.44	5.00E-05	4.28E-04	--	--

EVM0009995	4.67	25.30	-2.44	5.00E-05	4.28E-04	RNA processing and modification	WD domain, G-beta repeat
EVM0001452	6.49	35.11	-2.43	5.00E-05	4.28E-04	--	--
EVM0010446	18.81	101.30	-2.43	5.00E-05	4.28E-04	Transcription	Homeobox KN domain
EVM0010589	85.97	462.72	-2.43	5.00E-05	4.28E-04	--	bZIP transcription factor
EVM0000264	1.10	5.88	-2.41	5.00E-05	4.28E-04	--	--
EVM0004461	14.63	77.34	-2.40	5.00E-05	4.28E-04	--	Putative oxidoreductase C terminal
EVM0007517	55.71	294.22	-2.40	5.00E-05	4.28E-04	--	--
EVM0009031	3.81	20.03	-2.40	5.00E-05	4.28E-04	--	Glycosyl hydrolases family 43
EVM0002711	17.89	93.88	-2.39	5.00E-05	4.28E-04	General function prediction only	La domain
EVM0006820	19.02	99.54	-2.39	5.00E-05	4.28E-04	--	--
EVM0009591	9.10	47.61	-2.39	5.00E-05	4.28E-04	Amino acid transport and metabolism	Homocysteine S-methyltransferase
EVM0006723	135.41	704.37	-2.38	5.00E-05	4.28E-04	--	Conidiation protein 6
EVM0003267	7123.21	36948.10	-2.37	5.00E-05	4.28E-04	--	Conidiation protein 6

EVM0000628	17.00	88.10	-2.37	5.00E-05	4.28E-04	--	--
EVM0003306	37.08	191.95	-2.37	5.00E-05	4.28E-04	Intracellular trafficking, secretion, and vesicular transport	Snf7
EVM0000393	461.63	2377.79	-2.36	5.00E-05	4.28E-04	--	--
EVM0002561	3.46	17.79	-2.36	5.00E-05	4.28E-04	--	--
EVM0006187	1.96	10.02	-2.36	5.00E-05	4.28E-04	Transcription	HMG (high mobility group) box
EVM0011239	62.13	317.03	-2.35	5.00E-05	4.28E-04	--	50S ribosome-binding GTPase
EVM0001277	4.91	25.03	-2.35	5.00E-05	4.28E-04	--	--
EVM0011815	20.87	106.09	-2.35	5.00E-05	4.28E-04	--	Membrane-associating domain
EVM0001122	27.42	139.35	-2.35	5.00E-05	4.28E-04	RNA processing and modification	Fibrillarin
EVM0004549	324.29	1646.46	-2.34	5.00E-05	4.28E-04	Lipid transport and metabolism	HIUase/Transthyretin family
EVM0003029	31.14	156.58	-2.33	5.00E-05	4.28E-04	Signal transduction mechanisms	Protein-tyrosine phosphatase
EVM0003631	4.22	21.16	-2.33	6.00E-04	3.72E-03	--	--

EVM0001591	1.17	5.80	-2.31	5.00E-05	4.28E-04	--	--
EVM0001076	6.27	30.99	-2.31	5.00E-05	4.28E-04	--	--
EVM0006881	16.18	79.95	-2.30	5.00E-05	4.28E-04	--	NUDIX domain
EVM0005036	18.21	89.13	-2.29	5.00E-05	4.28E-04	--	--
EVM0011804	4.39	21.51	-2.29	5.00E-05	4.28E-04	--	--
EVM0012165	3.25	15.87	-2.29	1.04E-02	3.87E-02	--	--
EVM0006689	14.87	72.27	-2.28	5.00E-05	4.28E-04	General function prediction only	--
EVM0005265	47.44	230.58	-2.28	5.00E-05	4.28E-04	--	--
EVM0004866	158.25	768.83	-2.28	5.00E-05	4.28E-04	Function unknown	Uncharacterised protein family (UPF0121)
EVM0007661	8.98	43.54	-2.28	5.00E-05	4.28E-04	--	C2 domain
EVM0005795	6.06	29.33	-2.28	5.00E-05	4.28E-04	Chromatin structure and dynamics	Core histone H2A/H2B/H3/H4
EVM0010440	55.33	267.07	-2.27	5.00E-05	4.28E-04	--	Cytochrome c/c1 heme lyase
EVM0007572	3.94	18.95	-2.27	5.00E-05	4.28E-04	--	--

EVM0004071	17.57	84.50	-2.27	5.00E-05	4.28E-04	Transcription	GATA zinc finger
EVM0005110	30.25	145.03	-2.26	5.00E-05	4.28E-04	Transcription	--
EVM0002898	13.73	65.76	-2.26	5.00E-05	4.28E-04	Transcription	RNA polymerase Rpb3/RpoA insert domain
EVM0006542	52.74	251.58	-2.25	5.00E-05	4.28E-04	--	Ricin-type beta-trefoil lectin domain
EVM0003700	0.27	1.27	-2.25	5.00E-05	4.28E-04	Replication, recombination and repair	DEAD/DEAH box helicase
EVM0012086	56.64	269.53	-2.25	5.00E-05	4.28E-04	Carbohydrate transport and metabolism	Major Facilitator Superfamily
EVM0010113	29.57	139.57	-2.24	5.00E-05	4.28E-04	--	--
EVM0005861	76.15	358.24	-2.23	5.00E-05	4.28E-04	Inorganic ion transport and metabolism	Catalase
EVM0001519	8.33	39.11	-2.23	5.00E-05	4.28E-04	--	--
EVM0008791	7.34	34.32	-2.23	5.00E-05	4.28E-04	Coenzyme transport and metabolism	Dephospho-CoA kinase
EVM0001342	31.02	144.47	-2.22	5.00E-05	4.28E-04	Defense mechanisms	TB2/DP1, HVA22 family
EVM0006749	19.04	88.66	-2.22	5.00E-05	4.28E-04	--	Membrane bound O-acyl transferase family
EVM0009757	77.40	360.03	-2.22	5.00E-05	4.28E-04	--	--

EVM0007852	799.16	3705.76	-2.21	5.00E-05	4.28E-04	Posttranslational modification, protein turnover, chaperones	DnaJ C terminal domain
EVM0001566	2.46	11.30	-2.20	5.00E-05	4.28E-04	General function prediction only	Sugar (and other) transporter
EVM0010855	7.44	34.24	-2.20	5.00E-05	4.28E-04	--	--
EVM0007476	4.35	19.97	-2.20	5.00E-05	4.28E-04	--	Protein of unknown function (DUF1399)
EVM0002798	3.75	17.23	-2.20	5.00E-05	4.28E-04	--	F-box-like
EVM0007261	230.85	1057.83	-2.20	5.00E-05	4.28E-04	--	--
EVM0004228	10.08	46.11	-2.19	5.00E-05	4.28E-04	Translation, ribosomal structure and biogenesis	Brix domain
EVM0012010	20.73	94.44	-2.19	5.00E-05	4.28E-04	--	--
EVM0007187	5.62	25.43	-2.18	5.00E-05	4.28E-04	--	Pregnancy-associated plasma protein-A
EVM0002910	16.37	73.90	-2.17	5.00E-05	4.28E-04	--	--
EVM0002505	200.98	906.49	-2.17	5.00E-05	4.28E-04	--	--
EVM0001127	30.20	135.73	-2.17	5.00E-05	4.28E-04	General function prediction only	Ras family

EVM0008343	6.92	31.11	-2.17	5.00E-05	4.28E-04	--	--
EVM0009027	34.24	153.59	-2.17	5.00E-05	4.28E-04	--	F-box-like
EVM0002076	184.01	823.43	-2.16	5.00E-05	4.28E-04	--	CFEM domain
EVM0000746	17.86	79.61	-2.16	5.00E-05	4.28E-04	--	--
EVM0006282	2.76	12.24	-2.15	5.00E-05	4.28E-04	--	--
EVM0010633	739.01	3281.28	-2.15	5.00E-05	4.28E-04	Energy production and conversion	--
EVM0005054	18.28	81.05	-2.15	5.00E-05	4.28E-04	General function prediction only	C2 domain
EVM0002723	11.98	53.00	-2.15	5.00E-05	4.28E-04	--	--
EVM0004341	99.35	438.56	-2.14	5.00E-05	4.28E-04	Function unknown	CSL zinc finger
EVM0010935	16.37	72.16	-2.14	5.00E-05	4.28E-04	General function prediction only	U3-containing 90S pre-ribosomal complex subunit
EVM0004778	62.74	276.37	-2.14	5.00E-05	4.28E-04	General function prediction only	Thioesterase superfamily
EVM0001996	39.85	175.41	-2.14	5.00E-05	4.28E-04	Translation, ribosomal structure and biogenesis	SBDS protein C-terminal domain
EVM0010318	3.61	15.87	-2.14	5.00E-05	4.28E-04	--	--

EVM0005285	1034.32	4536.14	-2.13	5.00E-05	4.28E-04	--	--
EVM0007323	429.64	1877.24	-2.13	5.00E-05	4.28E-04	Function unknown	Domain of unknown function (DUF543)
EVM0009720	8.79	38.33	-2.12	5.00E-05	4.28E-04	Cell cycle control, cell division, chromosome partitioning	LYAR-type C2HC zinc finger
EVM0008871	56.73	246.60	-2.12	5.00E-05	4.28E-04	General function prediction only	Ras family
EVM0002707	0.92	4.00	-2.11	8.05E-03	3.15E-02	--	zinc-RING finger domain
EVM0008242	1.48	6.41	-2.11	3.00E-04	2.07E-03	--	Glycosyl hydrolase family 12
EVM0009699	59.57	257.35	-2.11	5.00E-05	4.28E-04	Posttranslational modification, protein turnover, chaperones	Proteasome subunit
EVM0004368	1.93	8.35	-2.11	4.45E-03	1.96E-02	--	--
EVM0006136	96.87	417.01	-2.11	5.00E-05	4.28E-04	--	--
EVM0001218	35.45	151.91	-2.10	5.00E-05	4.28E-04	--	--
EVM0002358	11.05	47.36	-2.10	5.00E-05	4.28E-04	--	--
EVM0002995	9.37	40.07	-2.10	5.00E-05	4.28E-04	RNA processing and modification	FF domain

EVM0009733	8.04	34.16	-2.09	5.00E-05	4.28E-04	--	--
EVM0006917	4.18	17.73	-2.08	5.00E-05	4.28E-04	--	--
EVM0004716	9.84	41.61	-2.08	5.00E-05	4.28E-04	Replication, recombination and repair	Exonuclease
EVM0006717	12.48	52.79	-2.08	5.00E-05	4.28E-04	--	--
EVM0008153	28.18	119.13	-2.08	5.00E-05	4.28E-04	--	--
EVM0010921	12.13	50.96	-2.07	5.00E-05	4.28E-04	--	--
EVM0010403	4.09	17.08	-2.06	5.00E-05	4.28E-04	--	--
EVM0005968	5.69	23.71	-2.06	5.00E-05	4.28E-04	--	--
EVM0000557	12.73	52.98	-2.06	5.00E-05	4.28E-04	--	--
EVM0001604	198.42	820.81	-2.05	5.00E-05	4.28E-04	General function prediction only	Enoyl-(Acyl carrier protein) reductase
EVM0010332	2.64	10.84	-2.04	5.00E-05	4.28E-04	--	--
EVM0000363	9.96	40.60	-2.03	5.00E-05	4.28E-04	Posttranslational modification, protein turnover, chaperones	Zinc knuckle

EVM0010419	4.29	17.40	-2.02	5.00E-05	4.28E-04	--	Mis12-Mtw1 protein family
EVM0010548	23.11	93.75	-2.02	5.00E-05	4.28E-04	--	--
EVM0002270	2.93	11.86	-2.02	6.00E-03	2.49E-02	--	--
EVM0008877	17.03	68.85	-2.02	5.00E-05	4.28E-04	--	--
EVM0010306	16.65	67.06	-2.01	5.00E-05	4.28E-04	--	--
EVM0008356	12.11	48.78	-2.01	5.00E-05	4.28E-04	Function unknown	Utp11 protein
EVM0001552	13.94	55.87	-2.00	5.00E-05	4.28E-04	#N/A	#N/A
EVM0011343	28.02	111.97	-2.00	5.00E-05	4.28E-04	Posttranslational modification, protein turnover, chaperones	Glutaredoxin
EVM0012244	1.18	4.69	-2.00	6.50E-04	3.98E-03	--	--
EVM0004499	22.37	89.13	-1.99	5.00E-05	4.28E-04	Transcription	HSF-type DNA-binding
EVM0006648	4.23	16.81	-1.99	5.00E-05	4.28E-04	--	--
EVM0004775	19.25	75.95	-1.98	5.00E-05	4.28E-04	--	Conidiation protein 6

EVM0004698	65.13	256.36	-1.98	5.00E-05	4.28E-04	General function prediction only	Hypoxia induced protein conserved region
EVM0011088	14.00	55.00	-1.97	5.00E-05	4.28E-04	Translation, ribosomal structure and biogenesis	Gar1/Naf1 RNA binding region
EVM0002548	13.79	54.15	-1.97	5.00E-05	4.28E-04	--	--
EVM0002743	12.34	48.46	-1.97	5.00E-05	4.28E-04	--	--
EVM0006700	162.97	638.54	-1.97	5.00E-05	4.28E-04	--	Aconitase family (aconitate hydratase)
EVM0004688	23.99	93.72	-1.97	5.00E-05	4.28E-04	--	--
EVM0005272	408.78	1587.89	-1.96	5.00E-05	4.28E-04	--	Ubiquinol-cytochrome-c reductase complex subunit (QCR10)
EVM0007254	6323.28	24518.90	-1.96	5.00E-05	4.28E-04	--	Ribosomal protein S30
EVM0005497	60.73	235.18	-1.95	5.00E-05	4.28E-04	--	--
EVM0003129	2.64	10.21	-1.95	5.00E-05	4.28E-04	--	--
EVM0002150	170.20	654.75	-1.94	5.00E-05	4.28E-04	General function prediction only	RNA recognition motif. (a.k.a. RRM, RBD, or RNP domain)
EVM0009023	9.60	36.89	-1.94	5.00E-05	4.28E-04	RNA processing and modification	Brix domain

EVM0006570	718.07	2755.72	-1.94	5.00E-05	4.28E-04	--	Pyridoxamine 5'-phosphate oxidase
EVM0003560	19.19	73.30	-1.93	8.85E-03	3.40E-02	--	G-patch domain
EVM0007566	1114.51	4249.04	-1.93	5.00E-05	4.28E-04	--	--
EVM0011052	0.74	2.80	-1.93	9.45E-03	3.58E-02	--	Glycosyl hydrolase family 12
EVM0005881	4.08	15.48	-1.92	5.00E-05	4.28E-04	--	Small nuclear RNA activating complex (SNAPc), subunit SNAP43
EVM0008721	1.68	6.36	-1.92	5.00E-05	4.28E-04	Lipid transport and metabolism	Hydroxymethylglutaryl-coenzyme A synthase N terminal
EVM0003548	8.36	31.64	-1.92	5.00E-05	4.28E-04	Signal transduction mechanisms	--
EVM0007199	107.14	404.82	-1.92	5.00E-05	4.28E-04	General function prediction only	Yippee zinc-binding/DNA-binding /Mis18, centromere assembly
EVM0012226	1.89	7.10	-1.91	4.50E-04	2.92E-03	--	--
EVM0005367	1.61	6.05	-1.91	5.00E-05	4.28E-04	--	--
EVM0008224	126.92	476.01	-1.91	5.00E-05	4.28E-04	Amino acid transport and metabolism	Protein of unknown function (DUF619)
EVM0012020	0.72	2.68	-1.91	3.90E-03	1.76E-02	--	--

EVM0009798	47.15	176.51	-1.90	5.00E-05	4.28E-04	RNA processing and modification	RNA recognition motif. (a.k.a. RRM, RBD, or RNP domain)
EVM0010344	1055.50	3949.47	-1.90	5.00E-05	4.28E-04	--	ATP synthase complex subunit h
EVM0002660	8.84	33.00	-1.90	5.00E-05	4.28E-04	Chromatin structure and dynamics	MOZ/SAS family
EVM0005619	9.47	35.23	-1.89	5.00E-05	4.28E-04	--	60Kd inner membrane protein
EVM0006946	10.95	40.71	-1.89	5.00E-05	4.28E-04	--	--
EVM0005014	4.72	17.51	-1.89	5.00E-05	4.28E-04	--	--
EVM0010141	48.19	178.63	-1.89	5.00E-05	4.28E-04	General function prediction only	Prp19/Pso4-like
EVM0011950	577.86	2141.59	-1.89	5.00E-05	4.28E-04	Posttranslational modification, protein turnover, chaperones	CS domain
EVM0005691	0.43	1.60	-1.89	3.15E-03	1.47E-02	--	Cytochrome P450
EVM0010820	1553.60	5752.51	-1.89	5.00E-05	4.28E-04	Energy production and conversion	Cytochrome oxidase c subunit VIb
EVM0010530	57.40	212.30	-1.89	5.00E-05	4.28E-04	--	--
EVM0005530	34.95	129.27	-1.89	5.00E-05	4.28E-04	--	XFP N-terminal domain

EVM0003373	2.09	7.70	-1.88	2.00E-04	1.46E-03	--	--
EVM0006992	12.70	46.74	-1.88	5.00E-05	4.28E-04	--	Cytochrome P450
EVM0001520	4.32	15.87	-1.88	5.00E-05	4.28E-04	--	--
EVM0006915	28.66	105.38	-1.88	5.00E-05	4.28E-04	--	Velvet factor
EVM0001178	21.49	78.95	-1.88	5.00E-05	4.28E-04	--	--
EVM0005891	23.59	86.60	-1.88	5.00E-05	4.28E-04	Energy production and conversion	Pyridine nucleotide-disulphide oxidoreductase
EVM0003817	5.59	20.50	-1.87	5.00E-05	4.28E-04	--	--
EVM0011475	6.49	23.79	-1.87	5.00E-05	4.28E-04	--	--
EVM0009527	6.09	22.17	-1.87	5.00E-05	4.28E-04	Replication, recombination and repair	Cid1 family poly A polymerase
EVM0007162	14.24	51.71	-1.86	5.00E-05	4.28E-04	RNA processing and modification	Helicase associated domain (HA2)
EVM0003249	70.45	255.26	-1.86	5.00E-05	4.28E-04	--	--
EVM0005429	10.17	36.83	-1.86	5.00E-05	4.28E-04	Transcription	CCAAT-binding transcription factor (CBF-B/NF-YA) subunit B

EVM0005972	772.87	2795.82	-1.85	5.00E-05	4.28E-04	--	--
EVM0005079	59.35	214.60	-1.85	5.00E-05	4.28E-04	Coenzyme transport and metabolism	Pantoate-beta-alanine ligase
EVM0008058	11.63	42.04	-1.85	5.00E-05	4.28E-04	--	--
EVM0011482	22.08	79.69	-1.85	5.00E-05	4.28E-04	Function unknown	WD domain, G-beta repeat
EVM0011852	45.43	163.64	-1.85	5.00E-05	4.28E-04	--	--
EVM0001573	1.66	5.95	-1.84	1.50E-04	1.14E-03	--	--
EVM0003103	58.14	208.62	-1.84	5.00E-05	4.28E-04	--	--
EVM0004358	154.92	554.44	-1.84	5.00E-05	4.28E-04	--	Heat shock factor binding protein 1
EVM0001259	10.45	37.34	-1.84	5.00E-05	4.28E-04	General function prediction only	WD domain, G-beta repeat
EVM0007388	14.33	51.11	-1.83	5.00E-05	4.28E-04	--	--
EVM0009908	352.00	1255.26	-1.83	5.00E-05	4.28E-04	--	--
EVM0009041	10.37	36.94	-1.83	5.00E-05	4.28E-04	--	--
EVM0004911	415.95	1480.83	-1.83	5.00E-05	4.28E-04	--	--

EVM0008074	2.34	8.31	-1.83	5.30E-03	2.26E-02	Chromatin structure and dynamics	Core histone H2A/H2B/H3/H4
EVM0006020	40.69	144.13	-1.82	5.00E-05	4.28E-04	--	--
EVM0011517	15.58	54.91	-1.82	5.00E-05	4.28E-04	--	RNA12 protein
EVM0008717	53.99	190.08	-1.82	5.00E-05	4.28E-04	--	Seed maturation protein
EVM0010336	37.24	131.06	-1.82	5.00E-05	4.28E-04	--	--
EVM0002297	237.19	834.10	-1.81	5.00E-05	4.28E-04	--	--
EVM0008982	3.01	10.57	-1.81	5.00E-05	4.28E-04	--	--
EVM0001990	1164.43	4087.02	-1.81	5.00E-05	4.28E-04	Posttranslational modification, protein turnover, chaperones	Hsp20/alpha crystallin family
EVM0000869	219.77	768.15	-1.81	5.00E-05	4.28E-04	--	--
EVM0003609	221.00	771.77	-1.80	5.00E-05	4.28E-04	--	Cytochrome oxidase c subunit VIb
EVM0007627	2314.50	8066.33	-1.80	5.00E-05	4.28E-04	Posttranslational modification, protein turnover, chaperones	Hsp20/alpha crystallin family
EVM0011971	8.85	30.81	-1.80	5.00E-05	4.28E-04	Transcription	HMG (high mobility group) box

EVM0008237	19.35	67.33	-1.80	5.00E-05	4.28E-04	--	RNA ligase
EVM0000147	3.35	11.64	-1.80	5.00E-05	4.28E-04	--	--
EVM0000932	62.30	216.15	-1.79	5.00E-05	4.28E-04	--	--
EVM0005976	107.64	373.40	-1.79	5.00E-05	4.28E-04	General function prediction only	Sugar (and other) transporter
EVM0000435	45.83	158.90	-1.79	5.00E-05	4.28E-04	General function prediction only	Glycosyl transferase family 2
EVM0007798	2.19	7.59	-1.79	5.00E-05	4.28E-04	Transcription	TEA/ATTS domain family
EVM0000419	47.31	163.41	-1.79	5.00E-05	4.28E-04	Function unknown	Complex 1 protein (LYR family)
EVM0002677	0.43	1.48	-1.79	7.50E-03	2.98E-02	--	--
EVM0002912	156.77	540.40	-1.79	5.00E-05	4.28E-04	Function unknown	Telomere stability and silencing
EVM0003951	36.31	124.85	-1.78	5.00E-05	4.28E-04	Function unknown	RWD domain
EVM0007958	2.34	8.05	-1.78	5.00E-05	4.28E-04	Secondary metabolites biosynthesis, transport and catabolism	Multicopper oxidase
EVM0001827	3.26	11.16	-1.78	5.00E-05	4.28E-04	Replication, recombination and repair	Helicase conserved C-terminal domain

EVM0012268	52.13	178.54	-1.78	5.00E-05	4.28E-04	Function unknown	Pam16
EVM0008059	33.09	113.28	-1.78	5.00E-05	4.28E-04	Carbohydrate transport and metabolism	Domain of unknown function (DUF1793)
EVM0010061	48.69	166.45	-1.77	5.00E-05	4.28E-04	--	SH3 domain
EVM0012007	17.93	61.23	-1.77	5.00E-05	4.28E-04	--	--
EVM0000905	27.14	92.62	-1.77	5.00E-05	4.28E-04	--	--
EVM0001192	105.27	359.10	-1.77	5.00E-05	4.28E-04	Transcription	RNA polymerase Rpb3/Rpb11 dimerisation domain
EVM0011516	11.72	39.93	-1.77	5.00E-05	4.28E-04	--	C2H2-type zinc finger
EVM0004446	5.38	18.32	-1.77	5.00E-05	4.28E-04	Posttranslational modification, protein turnover, chaperones	Isoprenylcysteine carboxyl methyltransferase (ICMT) family
EVM0006839	2.38	8.10	-1.76	5.00E-05	4.28E-04	--	--
EVM0002867	34.56	117.39	-1.76	5.00E-05	4.28E-04	Lipid transport and metabolism	MBOAT, membrane-bound O-acyltransferase family
EVM0008230	17.37	58.98	-1.76	5.00E-05	4.28E-04	Transcription	Maf1 regulator
EVM0009322	8.65	29.37	-1.76	5.00E-05	4.28E-04	--	--

EVM0010609	159.06	538.91	-1.76	5.00E-05	4.28E-04	Intracellular trafficking, secretion, and vesicular transport	Translocation protein Sec62
EVM0007013	35.93	121.57	-1.76	5.00E-05	4.28E-04	--	Protein of unknown function (DUF1275)
EVM0001016	5.47	18.51	-1.76	5.00E-05	4.28E-04	--	--
EVM0001816	10.76	36.39	-1.76	5.00E-05	4.28E-04	Posttranslational modification, protein turnover, chaperones	Cyclophilin type peptidyl-prolyl cis-trans isomerase/CLD
EVM0001788	15.23	51.37	-1.75	5.00E-05	4.28E-04	--	--
EVM0009797	6.60	22.25	-1.75	5.00E-05	4.28E-04	--	Pheromone A receptor
EVM0004042	63.23	213.16	-1.75	5.00E-05	4.28E-04	--	--
EVM0006896	214.29	721.71	-1.75	1.00E-04	7.96E-04	--	Protein of unknown function (DUF4449)
EVM0009214	152.31	512.95	-1.75	5.00E-05	4.28E-04	General function prediction only	Casein kinase substrate phosphoprotein PP28
EVM0003453	113.79	383.15	-1.75	5.00E-05	4.28E-04	--	--
EVM0011734	6.18	20.81	-1.75	5.00E-05	4.28E-04	--	--
EVM0001247	34.90	117.26	-1.75	5.00E-05	4.28E-04	--	MAC/Perforin domain

EVM0005279	619.87	2080.40	-1.75	5.00E-05	4.28E-04	--	Stress responsive A/B Barrel Domain
EVM0007796	54.09	181.52	-1.75	5.00E-05	4.28E-04	--	--
EVM0011033	3.52	11.80	-1.74	5.00E-05	4.28E-04	--	--
EVM0007196	234.14	783.54	-1.74	5.00E-05	4.28E-04	RNA processing and modification	LSM domain
EVM0010029	135.10	451.64	-1.74	5.00E-05	4.28E-04	--	Microsomal signal peptidase 25 kDa subunit (SPC25)
EVM0008461	10.87	36.33	-1.74	5.00E-05	4.28E-04	--	Putative snoRNA binding domain
EVM0008272	0.86	2.88	-1.74	4.50E-04	2.92E-03	--	--
EVM0000829	4.19	14.00	-1.74	2.10E-03	1.06E-02	--	--
EVM0004843	10.29	34.31	-1.74	5.00E-05	4.28E-04	--	Ricin-type beta-trefoil lectin domain-like
EVM0005234	0.92	3.06	-1.74	5.50E-04	3.46E-03	--	--
EVM0011587	159.68	530.01	-1.73	5.00E-05	4.28E-04	--	--
EVM0011107	80.25	266.21	-1.73	5.00E-05	4.28E-04	--	--
EVM0009051	296.43	981.43	-1.73	5.00E-05	4.28E-04	Translation, ribosomal structure and biogenesis	Ribosomal protein L34

EVM0004357	162.42	536.82	-1.72	1.60E-03	8.44E-03	--	--
EVM0004818	13.48	44.44	-1.72	5.00E-05	4.28E-04	#N/A	#N/A
EVM0010805	14.96	49.24	-1.72	5.00E-05	4.28E-04	General function prediction only	GMC oxidoreductase
EVM0000406	4.56	14.95	-1.71	5.00E-05	4.28E-04	--	Protein kinase domain
EVM0009546	19.42	63.68	-1.71	5.00E-05	4.28E-04	--	Fungal specific transcription factor domain
EVM0002786	1247.12	4081.63	-1.71	5.00E-05	4.28E-04	--	--
EVM0005796	1012.35	3312.97	-1.71	5.00E-05	4.28E-04	Translation, ribosomal structure and biogenesis	60s Acidic ribosomal protein
EVM0004028	62.79	205.30	-1.71	5.00E-05	4.28E-04	Cell cycle control, cell division, chromosome partitioning	--
EVM0007173	53.73	175.29	-1.71	5.00E-05	4.28E-04	Replication, recombination and repair	Nucleosome assembly protein (NAP)
EVM0007752	0.40	1.29	-1.71	8.75E-03	3.37E-02	--	--
EVM0009923	621.51	2026.31	-1.71	5.00E-05	4.28E-04	#N/A	#N/A
EVM0001318	3334.00	10865.60	-1.70	3.50E-04	2.36E-03	Posttranslational modification, protein turnover, chaperones	Hsp70 protein

EVM0009347	16.22	52.85	-1.70	5.00E-05	4.28E-04	--	WH2 motif
EVM0006606	52.24	170.16	-1.70	5.00E-05	4.28E-04	--	--
EVM0004482	1.59	5.19	-1.70	3.30E-03	1.53E-02	--	--
EVM0006132	57.10	185.61	-1.70	5.00E-05	4.28E-04	Intracellular trafficking, secretion, and vesicular transport	Peroxisomal biogenesis factor 11 (PEX11)
EVM0011165	29.59	96.16	-1.70	5.00E-05	4.28E-04	Energy production and conversion	Mitochondrial carrier protein
EVM0009732	18.80	60.80	-1.69	5.00E-05	4.28E-04	--	--
EVM0011891	10.00	32.25	-1.69	5.00E-05	4.28E-04	Posttranslational modification, protein turnover, chaperones	Glutathione S-transferase, N-terminal domain
EVM0004933	397.74	1280.81	-1.69	5.00E-05	4.28E-04	Posttranslational modification, protein turnover, chaperones	Sec61beta family
EVM0005500	11.98	38.59	-1.69	5.00E-05	4.28E-04	Function unknown	XAP5, circadian clock regulator
EVM0010779	259.74	835.69	-1.69	5.00E-05	4.28E-04	Intracellular trafficking, secretion, and vesicular transport	SURF4 family
EVM0005453	49.09	157.89	-1.69	5.00E-05	4.28E-04	--	--

EVM0000220	18.80	60.21	-1.68	8.60E-03	3.32E-02	Carbohydrate transport and metabolism	UAA transporter family
EVM0011758	3.84	12.30	-1.68	5.00E-05	4.28E-04	General function prediction only	THUMP domain
EVM0005185	136.66	437.06	-1.68	5.00E-05	4.28E-04	Amino acid transport and metabolism	Aminotransferase class-V
EVM0009813	49.94	159.55	-1.68	5.00E-05	4.28E-04	--	--
EVM0005073	10.32	32.96	-1.68	5.00E-05	4.28E-04	--	Nitronate monooxygenase
EVM0007331	3.46	11.04	-1.67	5.00E-05	4.28E-04	--	--
EVM0005717	39.57	126.03	-1.67	5.00E-05	4.28E-04	--	--
EVM0010331	18.01	57.26	-1.67	5.00E-05	4.28E-04	--	--
EVM0010883	201.12	639.22	-1.67	5.00E-05	4.28E-04	Cell wall/membrane/envelope biogenesis	Acetyltransferase (GNAT) family
EVM0002372	8.81	27.99	-1.67	5.00E-05	4.28E-04	--	Cytochrome P450
EVM0003772	3.79	12.03	-1.67	5.00E-05	4.28E-04	--	--
EVM0003992	14.16	44.92	-1.67	5.00E-05	4.28E-04	--	--

EVM0009819	23.61	74.88	-1.67	5.00E-05	4.28E-04	--	CBS domain
EVM0008730	16.51	52.29	-1.66	5.00E-05	4.28E-04	--	--
EVM0001421	51.01	161.48	-1.66	5.00E-05	4.28E-04	Cell cycle control, cell division, chromosome partitioning	Protein kinase domain
EVM0001320	22.62	71.50	-1.66	5.00E-05	4.28E-04	--	--
EVM0007843	28.52	89.98	-1.66	5.00E-05	4.28E-04	--	Dyp-type peroxidase family
EVM0011097	303.47	956.27	-1.66	5.00E-05	4.28E-04	--	RNA recognition motif. (a.k.a. RRM, RBD, or RNP domain)
EVM0003616	29.22	92.05	-1.66	5.00E-05	4.28E-04	Transcription	HSF-type DNA-binding
EVM0005253	425.02	1332.13	-1.65	5.00E-05	4.28E-04	--	--
EVM0000666	55.68	174.32	-1.65	5.00E-05	4.28E-04	--	--
EVM0007514	137.65	429.80	-1.64	5.00E-05	4.28E-04	--	RTA1 like protein
EVM0004582	2.35	7.33	-1.64	2.30E-03	1.14E-02	--	--
EVM0006949	68.14	212.58	-1.64	5.00E-05	4.28E-04	--	N-terminal domain of ribose phosphate pyrophosphokinase

EVM0000927	140.16	437.11	-1.64	5.00E-05	4.28E-04	Secondary metabolites biosynthesis, transport and catabolism	Pyridine nucleotide-disulphide oxidoreductase
EVM0000390	8.67	27.02	-1.64	5.00E-05	4.28E-04	Amino acid transport and metabolism	Cys/Met metabolism PLP-dependent enzyme
EVM0007872	31.65	98.52	-1.64	5.00E-05	4.28E-04	--	--
EVM0009085	584.49	1816.44	-1.64	5.00E-05	4.28E-04	--	--
EVM0008537	9774.36	30297.40	-1.63	2.35E-03	1.16E-02	Posttranslational modification, protein turnover, chaperones	Hsp20/alpha crystallin family
EVM0005625	2.42	7.48	-1.63	5.00E-05	4.28E-04	--	--
EVM0003837	603.06	1864.50	-1.63	5.00E-05	4.28E-04	--	--
EVM0002338	13.22	40.84	-1.63	5.00E-05	4.28E-04	Lipid transport and metabolism	PAP2 superfamily
EVM0012239	1.76	5.43	-1.63	5.00E-05	4.28E-04	--	--
EVM0007949	14.57	44.95	-1.63	5.00E-05	4.28E-04	RNA processing and modification	WD domain, G-beta repeat
EVM0010521	41.02	126.46	-1.62	5.00E-05	4.28E-04	--	--
EVM0009399	19.78	60.91	-1.62	5.00E-05	4.28E-04	--	Mediator complex subunit 25 von Willebrand factor type A

EVM0003466	23.04	70.85	-1.62	5.00E-05	4.28E-04	--	NYN domain
EVM0002644	1484.48	4563.43	-1.62	8.50E-04	4.98E-03	--	--
EVM0001510	80.14	246.20	-1.62	5.00E-05	4.28E-04	Posttranslational modification, protein turnover, chaperones	Eukaryotic aspartyl protease
EVM0004770	11.36	34.83	-1.62	5.00E-05	4.28E-04	--	WH1 domain
EVM0004238	23.97	73.47	-1.62	5.00E-05	4.28E-04	--	SMI1 / KNR4 family (SUKH-1)
EVM0008511	36.18	110.86	-1.62	5.00E-05	4.28E-04	RNA processing and modification	Ribosomal RNA-processing protein 7 (RRP7)
EVM0010252	43.86	134.34	-1.61	5.00E-05	4.28E-04	--	PX domain
EVM0000794	547.80	1675.77	-1.61	5.00E-05	4.28E-04	Posttranslational modification, protein turnover, chaperones	Redoxin
EVM0002465	4.56	13.95	-1.61	2.50E-04	1.77E-03	--	50S ribosome-binding GTPase
EVM0011344	9.71	29.66	-1.61	5.00E-05	4.28E-04	RNA processing and modification	Mpp10 protein
EVM0001778	135.45	413.61	-1.61	5.00E-05	4.28E-04	Coenzyme transport and metabolism	Pyridoxamine 5'-phosphate oxidase
EVM0006301	31.86	97.07	-1.61	5.00E-05	4.28E-04	Carbohydrate transport and metabolism	Amidohydrolase family

EVM0001614	3.34	10.17	-1.61	5.00E-05	4.28E-04	--	--
EVM0002694	4.22	12.82	-1.60	5.00E-05	4.28E-04	--	DNA polymerase subunit Cdc27
EVM0010702	11.60	35.03	-1.59	5.00E-05	4.28E-04	--	--
EVM0006754	209.05	631.22	-1.59	5.00E-05	4.28E-04	Coenzyme transport and metabolism	Coproporphyrinogen III oxidase
EVM0009791	7.67	23.09	-1.59	5.00E-05	4.28E-04	Transcription	RNA polymerase Rpb1, domain 5
EVM0002264	90.73	272.80	-1.59	5.00E-05	4.28E-04	--	--
EVM0010015	0.66	1.98	-1.59	8.90E-03	3.41E-02	#N/A	#N/A
EVM0003296	142.35	427.41	-1.59	5.00E-05	4.28E-04	General function prediction only	Domain of unknown function DUF221
EVM0005402	2.39	7.16	-1.58	5.00E-05	4.28E-04	--	F-box domain
EVM0003866	80.48	241.18	-1.58	5.00E-05	4.28E-04	--	DAD family
EVM0008406	26.64	79.82	-1.58	5.00E-05	4.28E-04	Lipid transport and metabolism	AMP-binding enzyme
EVM0010558	18.17	54.39	-1.58	5.00E-05	4.28E-04	--	--
EVM0005758	58.97	176.41	-1.58	5.00E-05	4.28E-04	Lipid transport and metabolism	Putative undecaprenyl diphosphate synthase

EVM0007305	247.84	740.78	-1.58	5.00E-05	4.28E-04	RNA processing and modification	U1 zinc finger
EVM0001647	48.79	145.80	-1.58	5.00E-05	4.28E-04	General function prediction only	Zinc-finger double-stranded RNA-binding
EVM0009705	76.45	228.23	-1.58	5.00E-05	4.28E-04	--	--
EVM0000266	22.86	68.24	-1.58	5.00E-05	4.28E-04	--	--
EVM0007445	3.29	9.80	-1.58	5.00E-05	4.28E-04	--	--
EVM0008804	57.59	171.23	-1.57	5.00E-05	4.28E-04	--	--
EVM0009711	125.87	373.42	-1.57	5.00E-05	4.28E-04	--	Fungal hydrophobin
EVM0008198	39.94	118.44	-1.57	5.00E-05	4.28E-04	Transcription	Transcription initiation factor IIF, beta subunit
EVM0004809	40.01	118.60	-1.57	5.00E-05	4.28E-04	Posttranslational modification, protein turnover, chaperones	Myb-like DNA-binding domain
EVM0008622	75.43	223.50	-1.57	5.00E-05	4.28E-04	--	50S ribosome-binding GTPase
EVM0006976	13.80	40.89	-1.57	5.00E-05	4.28E-04	Inorganic ion transport and metabolism	Regulator of volume decrease after cellular swelling
EVM0002302	22.38	66.30	-1.57	5.00E-05	4.28E-04	--	--

EVM0000986	9.84	29.16	-1.57	5.00E-05	4.28E-04	--	--
EVM0001355	215.77	638.75	-1.57	1.50E-04	1.14E-03	--	--
EVM0008504	30.44	90.04	-1.56	5.00E-05	4.28E-04	--	F-box-like
EVM0011270	9.23	27.27	-1.56	5.00E-05	4.28E-04	--	cAMP phosphodiesterases class-II
EVM0011757	3.67	10.83	-1.56	9.50E-04	5.46E-03	--	--
EVM0010583	329.63	973.19	-1.56	5.00E-05	4.28E-04	--	--
EVM0010158	53.50	157.86	-1.56	5.00E-05	4.28E-04	Function unknown	Pre-rRNA-processing protein TSR2
EVM0004617	575.75	1693.60	-1.56	5.00E-05	4.28E-04	--	ESSS subunit of NADH:ubiquinone oxidoreductase (complex I)
EVM0000784	9.47	27.84	-1.56	5.00E-05	4.28E-04	--	--
EVM0009146	1.57	4.62	-1.56	6.20E-03	2.56E-02	--	Glycosyl hydrolase family 61
EVM0000883	3.80	11.15	-1.55	5.00E-05	4.28E-04	Transcription	HMG (high mobility group) box
EVM0000240	10.82	31.75	-1.55	1.00E-04	7.96E-04	Transcription	MED6 mediator sub complex component

EVM0009204	10.50	30.76	-1.55	5.00E-05	4.28E-04	RNA processing and modification	--
EVM0003326	34.24	100.26	-1.55	5.00E-05	4.28E-04	General function prediction only	R3H domain
EVM0003558	31.97	93.52	-1.55	5.00E-05	4.28E-04	--	Methyltransferase domain
EVM0005508	103.77	303.32	-1.55	5.00E-05	4.28E-04	Posttranslational modification, protein turnover, chaperones	OST3 / OST6 family
EVM0008305	9.82	28.69	-1.55	5.00E-05	4.28E-04	General function prediction only	U3 small nucleolar RNA-associated protein 6
EVM0009845	13.77	40.20	-1.55	2.00E-04	1.46E-03	--	--
EVM0005613	18.39	53.56	-1.54	5.00E-05	4.28E-04	--	--
EVM0008163	4.18	12.15	-1.54	1.00E-04	7.96E-04	--	--
EVM0004215	453.48	1317.55	-1.54	5.00E-05	4.28E-04	Energy production and conversion	Cytochrome c oxidase subunit Va
EVM0002596	0.82	2.38	-1.54	3.85E-03	1.74E-02	--	Phosphotransferase enzyme family
EVM0000527	21.10	61.27	-1.54	5.00E-05	4.28E-04	General function prediction only	Methyltransferase domain
EVM0009955	11.02	32.01	-1.54	5.00E-05	4.28E-04	--	--

EVM0002266	300.51	872.36	-1.54	5.00E-05	4.28E-04	--	Protein of unknown function (DUF3429)
EVM0004837	100.55	291.48	-1.54	5.00E-05	4.28E-04	General function prediction only	Ras family
EVM0003322	9.08	26.32	-1.54	5.00E-05	4.28E-04	General function prediction only	Sugar (and other) transporter
EVM0003224	61.67	178.72	-1.54	5.00E-05	4.28E-04	General function prediction only	Phosphatidylethanolamine-binding protein
EVM0009206	26.38	76.45	-1.53	5.00E-05	4.28E-04	--	Protein of unknown function (DUF3129)
EVM0005583	16.59	47.99	-1.53	5.00E-05	4.28E-04	--	--
EVM0007890	184.25	532.65	-1.53	5.00E-05	4.28E-04	--	--
EVM0008702	157.43	455.02	-1.53	5.00E-05	4.28E-04	Intracellular trafficking, secretion, and vesicular transport	PRELI-like family
EVM0000890	159.05	459.47	-1.53	5.00E-05	4.28E-04	Translation, ribosomal structure and biogenesis	PUA domain
EVM0010645	9.87	28.49	-1.53	5.00E-05	4.28E-04	Cytoskeleton	Splicing factor, Prp19-binding domain
EVM0004272	379.07	1094.18	-1.53	5.00E-05	4.28E-04	--	Translationally controlled tumour protein
EVM0009625	11.69	33.73	-1.53	5.00E-05	4.28E-04	--	AIG1 family

EVM0005280	1.47	4.24	-1.53	1.20E-03	6.64E-03	--	Phosphotransferase enzyme family
EVM0006159	47.41	136.47	-1.53	5.00E-05	4.28E-04	--	--
EVM0002923	4250.88	12227.00	-1.52	5.00E-05	4.28E-04	--	CsbD-like
EVM0007878	19.81	56.94	-1.52	5.00E-05	4.28E-04	Posttranslational modification, protein turnover, chaperones	BCS1 N terminal
EVM0008481	10.28	29.48	-1.52	5.00E-05	4.28E-04	Inorganic ion transport and metabolism	CorA-like Mg2+ transporter protein
EVM0002697	4.88	13.99	-1.52	8.00E-04	4.73E-03	--	--
EVM0005991	266.57	763.68	-1.52	5.00E-05	4.28E-04	--	SAP domain
EVM0006292	5.14	14.73	-1.52	5.00E-05	4.28E-04	--	Fungal Zn(2)-Cys(6) binuclear cluster domain
EVM0007771	8.61	24.57	-1.51	3.00E-04	2.07E-03	--	--
EVM0011887	9.38	26.73	-1.51	1.00E-04	7.96E-04	--	--
EVM0004134	51.54	146.79	-1.51	5.00E-05	4.28E-04	General function prediction only	RNA recognition motif. (a.k.a. RRM, RBD, or RNP domain)
EVM0005227	55.84	158.73	-1.51	5.00E-05	4.28E-04	Function unknown	Bromodomain

EVM0005831	12.92	36.71	-1.51	5.00E-05	4.28E-04	General function prediction only	3'-5' exonuclease
EVM0004691	19.14	54.33	-1.50	5.00E-05	4.28E-04	--	--
EVM0001809	98.29	278.85	-1.50	5.00E-05	4.28E-04	--	--
EVM0005630	145.42	411.85	-1.50	5.00E-05	4.28E-04	Translation, ribosomal structure and biogenesis	Mitochondrial ribosomal protein L51 / S25 / CI-B8 domain
EVM0008851	252.09	713.58	-1.50	5.00E-05	4.28E-04	Intracellular trafficking, secretion, and vesicular transport	Preprotein translocase subunit Sec66
EVM0002331	198.80	561.31	-1.50	5.00E-05	4.28E-04	Posttranslational modification, protein turnover, chaperones	14-3-3 protein
EVM0006561	7.62	21.48	-1.50	5.00E-05	4.28E-04	--	--
EVM0008650	73.28	206.52	-1.49	5.00E-05	4.28E-04	--	--
EVM0004953	61.60	173.46	-1.49	5.00E-05	4.28E-04	--	--
EVM0000218	102.05	287.31	-1.49	5.00E-05	4.28E-04	--	50S ribosome-binding GTPase
EVM0003855	4.75	13.38	-1.49	1.50E-04	1.14E-03	Signal transduction mechanisms	Protein kinase domain
EVM0008213	778.98	2190.78	-1.49	5.00E-05	4.28E-04	RNA processing and modification	LSM domain

EVM0007629	60.68	170.63	-1.49	5.00E-05	4.28E-04	General function prediction only	Protein of unknown function (DUF933)
EVM0009884	171.44	481.58	-1.49	5.00E-05	4.28E-04	--	Glycosyl hydrolase catalytic core
EVM0001583	29.75	83.56	-1.49	5.00E-05	4.28E-04	Intracellular trafficking, secretion, and vesicular transport	Ras family
EVM0006099	130.16	365.45	-1.49	5.00E-05	4.28E-04	Lipid transport and metabolism	Enoyl-CoA hydratase/isomerase family
EVM0010258	37.36	104.84	-1.49	5.00E-05	4.28E-04	--	--
EVM0011446	1632.45	4570.86	-1.49	5.00E-05	4.28E-04	--	--
EVM0002673	20.51	57.42	-1.49	5.00E-05	4.28E-04	--	--
EVM0000423	152.25	425.44	-1.48	5.00E-05	4.28E-04	#N/A	#N/A
EVM0006494	15.86	44.29	-1.48	5.00E-05	4.28E-04	Chromatin structure and dynamics	Sas10 C-terminal domain
EVM0008519	124.61	347.64	-1.48	5.00E-05	4.28E-04	RNA processing and modification	LUC7 N_terminus
EVM0002899	187.88	524.05	-1.48	5.00E-05	4.28E-04	--	Fatty acid desaturase
EVM0005109	4.25	11.83	-1.48	5.00E-05	4.28E-04	--	--

EVM0000031	410.77	1143.85	-1.48	5.00E-05	4.28E-04	--	--
EVM0010038	2.91	8.09	-1.48	5.40E-03	2.29E-02	--	--
EVM0007361	17.90	49.81	-1.48	5.00E-05	4.28E-04	RNA processing and modification	DEAD/DEAH box helicase
EVM0009999	8.29	23.05	-1.47	3.45E-03	1.59E-02	--	Rare lipoprotein A (RlpA)-like double-psi beta-barrel
EVM0003599	4.78	13.27	-1.47	5.05E-03	2.17E-02	Carbohydrate transport and metabolism	LysM domain
EVM0003574	36.69	101.76	-1.47	5.00E-05	4.28E-04	General function prediction only	PCI domain
EVM0007410	3.82	10.57	-1.47	5.00E-05	4.28E-04	#N/A	#N/A
EVM0003721	166.50	461.01	-1.47	5.00E-05	4.28E-04	--	--
EVM0005703	21.51	59.51	-1.47	5.00E-05	4.28E-04	Function unknown	Utp14 protein
EVM0002607	57.03	157.71	-1.47	5.00E-05	4.28E-04	Lipid transport and metabolism	Cupin-like domain
EVM0000019	31.20	86.28	-1.47	5.00E-05	4.28E-04	--	--
EVM0000219	58.89	162.71	-1.47	5.00E-05	4.28E-04	General function prediction only	Cyclin

EVM0004762	144.37	398.71	-1.47	5.00E-05	4.28E-04	Intracellular trafficking, secretion, and vesicular transport	Clathrin adaptor complex small chain
EVM0008002	130.81	361.22	-1.47	5.00E-05	4.28E-04	General function prediction only	Flavodoxin
EVM0007506	189.76	522.92	-1.46	5.00E-05	4.28E-04	Intracellular trafficking, secretion, and vesicular transport	Regulated-SNARE-like domain
EVM0002218	170.01	468.10	-1.46	5.00E-05	4.28E-04	--	--
EVM0008604	3.08	8.46	-1.46	1.00E-04	7.96E-04	--	--
EVM0002141	5.35	14.73	-1.46	4.00E-04	2.65E-03	--	--
EVM0007826	23.42	64.42	-1.46	5.00E-05	4.28E-04	General function prediction only	RNA recognition motif. (a.k.a. RRM, RBD, or RNP domain)
EVM0000209	8.50	23.39	-1.46	5.00E-05	4.28E-04	Translation, ribosomal structure and biogenesis	KRI1-like family C-terminal
EVM0008447	49.59	136.13	-1.46	5.00E-05	4.28E-04	--	--
EVM0009119	10.08	27.64	-1.46	5.00E-05	4.28E-04	--	--
EVM0001825	79.11	216.79	-1.45	5.00E-05	4.28E-04	Transcription	HSF-type DNA-binding
EVM0005377	6.19	16.93	-1.45	5.00E-05	4.28E-04	--	Cellulase (glycosyl hydrolase family 5)

EVM0008051	146.44	400.63	-1.45	5.00E-05	4.28E-04	--	SUR7/PalI family
EVM0009614	79.69	217.79	-1.45	5.00E-05	4.28E-04	--	--
EVM0005134	84.43	230.66	-1.45	5.00E-05	4.28E-04	Function unknown	COP9 signalosome, subunit CSN8
EVM0011134	19.38	52.92	-1.45	5.00E-05	4.28E-04	Function unknown	NRDE-2, necessary for RNA interference
EVM0010109	7.58	20.68	-1.45	5.00E-05	4.28E-04	Replication, recombination and repair	Eukaryotic and archaeal DNA primase, large subunit
EVM0010363	22.93	62.45	-1.45	5.00E-05	4.28E-04	--	Alpha/beta hydrolase family
EVM0000377	40.53	110.05	-1.44	5.00E-05	4.28E-04	--	--
EVM0003168	9.60	26.01	-1.44	1.25E-03	6.87E-03	--	--
EVM0004156	0.96	2.60	-1.44	2.00E-04	1.46E-03	--	--
EVM0010715	53.79	145.51	-1.44	5.00E-05	4.28E-04	RNA processing and modification	Isy1-like splicing family
EVM0007930	11.43	30.89	-1.43	5.00E-05	4.28E-04	--	RNA recognition motif (a.k.a. RRM, RBD, or RNP domain)
EVM0011808	3.05	8.22	-1.43	1.35E-03	7.33E-03	--	Lysine-specific metallo-endopeptidase
EVM0002948	33.72	90.82	-1.43	5.00E-05	4.28E-04	--	Protein of unknown function (Ytp1)

EVM0011851	5.54	14.89	-1.43	4.00E-04	2.65E-03	--	Domain of unknown function (DUF3328)
EVM0006795	860.74	2314.38	-1.43	3.90E-03	1.76E-02	--	--
EVM0000165	48.84	131.27	-1.43	5.00E-05	4.28E-04	Cell cycle control, cell division, chromosome partitioning	Cyclin, N-terminal domain
EVM0002127	38.35	103.06	-1.43	5.00E-05	4.28E-04	RNA processing and modification	RNA recognition motif. (a.k.a. RRM, RBD, or RNP domain)
EVM0004954	57.78	154.96	-1.42	5.00E-05	4.28E-04	--	--
EVM0000370	155.94	418.22	-1.42	5.00E-05	4.28E-04	--	Fungal fruit body lectin
EVM0001681	268.76	719.84	-1.42	5.00E-05	4.28E-04	Intracellular trafficking, secretion, and vesicular transport	Microsomal signal peptidase 12 kDa subunit (SPC12)
EVM0006400	4.27	11.42	-1.42	5.00E-05	4.28E-04	--	--
EVM0008835	55.75	149.26	-1.42	5.00E-05	4.28E-04	Carbohydrate transport and metabolism	--
EVM0002821	86.52	231.48	-1.42	4.00E-04	2.65E-03	--	--
EVM0001632	36.49	97.53	-1.42	5.00E-05	4.28E-04	--	--
EVM0003818	15.05	40.20	-1.42	5.00E-05	4.28E-04	RNA processing and modification	RNA recognition motif. (a.k.a. RRM, RBD, or RNP domain)

EVM0002245	24.28	64.74	-1.41	5.00E-05	4.28E-04	Function unknown	Leo1-like protein
EVM0008283	44.15	117.57	-1.41	5.00E-05	4.28E-04	--	Fungal hydrophobin
EVM0005649	153.19	407.26	-1.41	5.00E-05	4.28E-04	Transcription	Basic region leucine zipper
EVM0001419	216.66	575.26	-1.41	5.00E-05	4.28E-04	Signal transduction mechanisms	SGS domain
EVM0003839	516.29	1370.46	-1.41	5.00E-05	4.28E-04	Translation, ribosomal structure and biogenesis	Ribosomal S13/S15 N-terminal domain
EVM0005821	6.11	16.20	-1.41	5.00E-05	4.28E-04	--	--
EVM0004708	144.71	383.86	-1.41	5.00E-05	4.28E-04	--	SecY translocase
EVM0006082	2.37	6.28	-1.41	9.05E-03	3.46E-02	--	--
EVM0005245	24.45	64.79	-1.41	5.00E-05	4.28E-04	--	Ubiquitin-2 like Rad60 SUMO-like
EVM0011391	30.67	81.24	-1.41	5.00E-05	4.28E-04	Translation, ribosomal structure and biogenesis	Noc2p family
EVM0000381	15.88	42.04	-1.40	5.00E-05	4.28E-04	Function unknown	Folate-sensitive fragile site protein Fra10Ac1
EVM0000651	40.25	106.52	-1.40	5.00E-03	2.15E-02	--	Transient receptor potential (TRP) ion channel
EVM0006403	294.76	778.81	-1.40	5.00E-05	4.28E-04	Inorganic ion transport and metabolism	Anion-transporting ATPase

EVM0000472	1331.09	3510.98	-1.40	5.00E-05	4.28E-04	--	--
EVM0011042	149.02	391.77	-1.39	5.00E-05	4.28E-04	Signal transduction mechanisms	Inhibitor of apoptosis-promoting Bax1
EVM0003745	5.02	13.20	-1.39	5.00E-05	4.28E-04	--	--
EVM0007065	74.32	195.25	-1.39	5.00E-05	4.28E-04	--	--
EVM0005323	5.98	15.72	-1.39	1.00E-04	7.96E-04	--	--
EVM0001935	17.61	46.24	-1.39	5.00E-05	4.28E-04	--	Myosin-like coiled-coil protein
EVM0010981	1381.82	3628.46	-1.39	1.55E-03	8.22E-03	Function unknown	Activator of Hsp90 ATPase, N-terminal
EVM0010500	36.63	96.06	-1.39	5.00E-05	4.28E-04	Transcription	RNA polymerase II transcription factor SIII (Elongin) subunit A
EVM0011226	121.74	318.86	-1.39	5.00E-05	4.28E-04	RNA processing and modification	Zinc-finger double-stranded RNA-binding
EVM0003499	605.32	1583.75	-1.39	5.00E-05	4.28E-04	--	Rare lipoprotein A (RlpA)-like double-psi beta-barrel
EVM0004433	10.36	27.08	-1.39	5.00E-05	4.28E-04	--	Lactonase, 7-bladed beta-propeller
EVM0002996	22.09	57.76	-1.39	5.00E-05	4.28E-04	Transcription	Sin3 associated polypeptide p18 (SAP18)
EVM0009944	8.88	23.15	-1.38	5.00E-05	4.28E-04	--	--

EVM0009653	22.76	59.31	-1.38	5.00E-05	4.28E-04	--	Cellulase (glycosyl hydrolase family 5)
EVM0004924	10.49	27.32	-1.38	1.40E-02	4.90E-02	--	--
EVM0000593	100.34	261.18	-1.38	5.00E-05	4.28E-04	--	Protein of unknown function (DUF952)
EVM0009691	31.92	83.08	-1.38	5.00E-05	4.28E-04	Carbohydrate transport and metabolism	pfkB family carbohydrate kinase
EVM0010758	17.78	46.25	-1.38	5.00E-05	4.28E-04	RNA processing and modification	RNA 3'-terminal phosphate cyclase
EVM0006402	164.77	428.53	-1.38	5.00E-05	4.28E-04	--	--
EVM0008637	14.47	37.61	-1.38	5.00E-05	4.28E-04	RNA processing and modification	WD domain, G-beta repeat
EVM0006319	1.55	4.02	-1.38	5.45E-03	2.31E-02	--	--
EVM0012074	18.71	48.60	-1.38	5.00E-05	4.28E-04	Lipid transport and metabolism	CDP-alcohol phosphatidyltransferase
EVM0007190	8.15	21.15	-1.38	5.00E-05	4.28E-04	--	50S ribosome-binding GTPase
EVM0002154	1.66	4.29	-1.37	1.85E-03	9.52E-03	--	--
EVM0008088	5.56	14.42	-1.37	5.50E-04	3.46E-03	--	--
EVM0008651	86.60	224.47	-1.37	5.00E-05	4.28E-04	Inorganic ion transport and metabolism	Copper/zinc superoxide dismutase (SODC)

EVM0000204	890.32	2303.93	-1.37	4.50E-04	2.92E-03	--	short chain dehydrogenase
EVM0008011	65.93	170.62	-1.37	5.00E-05	4.28E-04	--	--
EVM0004173	22.04	57.02	-1.37	5.00E-05	4.28E-04	--	--
EVM0003008	55.18	142.78	-1.37	5.00E-05	4.28E-04	--	--
EVM0007861	87.11	225.36	-1.37	5.00E-05	4.28E-04	--	Protein of unknown function (DUF1183)
EVM0010095	7.47	19.32	-1.37	5.00E-05	4.28E-04	Replication, recombination and repair	DNA polymerase alpha/epsilon subunit B
EVM0002317	49.27	127.29	-1.37	3.30E-03	1.53E-02	--	--
EVM0006575	4.16	10.73	-1.37	5.00E-05	4.28E-04	--	--
EVM0008064	12.24	31.56	-1.37	5.00E-05	4.28E-04	--	Shugoshin C terminus
EVM0012095	7.23	18.64	-1.37	2.00E-04	1.46E-03	#N/A	#N/A
EVM0011261	21.47	55.30	-1.36	5.00E-05	4.28E-04	General function prediction only	WD domain, G-beta repeat
EVM0007650	120.79	310.96	-1.36	5.00E-05	4.28E-04	--	--
EVM0003518	21.87	56.29	-1.36	1.00E-04	7.96E-04	--	--

EVM0007616	24.00	61.74	-1.36	5.00E-05	4.28E-04	Transcription	MT-A70
EVM0001772	176.34	453.19	-1.36	5.00E-05	4.28E-04	Translation, ribosomal structure and biogenesis	Ribosomal protein L36
EVM0009084	52.12	133.86	-1.36	5.00E-05	4.28E-04	Translation, ribosomal structure and biogenesis	Histidyl-tRNA synthetase
EVM0004477	24.13	61.91	-1.36	5.00E-05	4.28E-04	--	--
EVM0010867	928.29	2378.57	-1.36	1.00E-04	7.96E-04	--	--
EVM0009646	5.93	15.19	-1.36	7.50E-03	2.98E-02	--	Pheromone A receptor
EVM0005915	11.51	29.42	-1.35	5.00E-05	4.28E-04	--	Lipase (class 3)
EVM0004869	130.86	334.16	-1.35	5.00E-05	4.28E-04	Lipid transport and metabolism	Myo-inositol-1-phosphate synthase
EVM0000850	17.06	43.54	-1.35	1.00E-04	7.96E-04	--	--
EVM0006126	28.63	72.95	-1.35	5.00E-05	4.28E-04	--	--
EVM0006060	5.94	15.14	-1.35	1.00E-04	7.96E-04	--	PP-loop family
EVM0006316	5.94	15.11	-1.35	5.00E-05	4.28E-04	Function unknown	WW domain binding protein 11
EVM0011059	36.64	93.14	-1.35	5.00E-05	4.28E-04	--	--

EVM0007279	28.45	72.32	-1.35	1.00E-04	7.96E-04	--	--
EVM0006922	37.48	95.24	-1.35	1.00E-04	7.96E-04	--	--
EVM0001689	250.94	636.22	-1.34	5.00E-05	4.28E-04	General function prediction only	Ras family
EVM0005087	91.68	232.42	-1.34	5.00E-05	4.28E-04	Intracellular trafficking, secretion, and vesicular transport	Tim17/Tim22/Tim23/Pmp24 family
EVM0011499	13.28	33.64	-1.34	5.00E-05	4.28E-04	--	--
EVM0009851	14.31	36.24	-1.34	5.00E-05	4.28E-04	Function unknown	WD domain, G-beta repeat
EVM0010669	96.29	243.59	-1.34	5.00E-05	4.28E-04	--	--
EVM0000212	79.58	201.28	-1.34	5.00E-05	4.28E-04	Posttranslational modification, protein turnover, chaperones	Zinc finger, C3HC4 type (RING finger)
EVM0011085	32.62	82.46	-1.34	1.00E-04	7.96E-04	--	Translation initiation factor IF-3, C-terminal domain
EVM0007358	13.04	32.96	-1.34	5.00E-05	4.28E-04	--	--
EVM0004502	15.24	38.44	-1.34	5.00E-05	4.28E-04	RNA processing and modification	SUZ domain
EVM0005121	166.37	418.83	-1.33	5.00E-05	4.28E-04	General function prediction only	Ras family

EVM0004946	1.02	2.56	-1.33	1.02E-02	3.81E-02	--	--
EVM0002522	6.83	17.19	-1.33	1.00E-04	7.96E-04	--	Protein of unknown function (DUF1399)
EVM0008302	33.75	84.87	-1.33	5.00E-05	4.28E-04	--	--
EVM0007669	1.10	2.76	-1.33	4.95E-03	2.13E-02	General function prediction only	Zinc-finger double domain
EVM0010265	43.39	108.98	-1.33	5.00E-05	4.28E-04	General function prediction only	Conserved hypothetical ATP binding protein
EVM0010273	2.54	6.38	-1.33	3.00E-04	2.07E-03	--	--
EVM0004408	19.97	50.09	-1.33	5.00E-05	4.28E-04	--	Protein of unknown function (DUF563)
EVM0007912	6.46	16.16	-1.32	1.20E-03	6.64E-03	--	--
EVM0001156	118.61	296.49	-1.32	5.00E-05	4.28E-04	--	--
EVM0010557	6.14	15.34	-1.32	1.00E-04	7.96E-04	--	Repair protein Rad1/Rec1/Rad17
EVM0004661	5.54	13.84	-1.32	2.50E-04	1.77E-03	--	Cytochrome P450
EVM0012116	100.74	251.74	-1.32	5.00E-05	4.28E-04	Posttranslational modification, protein turnover, chaperones	Redoxin

EVM0012015	17.15	42.76	-1.32	5.00E-05	4.28E-04	Translation, ribosomal structure and biogenesis	BOP1NT (NUC169) domain
EVM0005216	44.31	110.45	-1.32	5.00E-05	4.28E-04	--	Whi5 like
EVM0011460	245.66	611.87	-1.32	5.00E-05	4.28E-04	--	--
EVM0001152	162.96	405.40	-1.31	5.00E-05	4.28E-04	General function prediction only	Mpv17 / PMP22 family
EVM0009800	219.06	544.69	-1.31	5.00E-05	4.28E-04	--	Mitochondrial ribosomal protein L31
EVM0003778	5.97	14.85	-1.31	1.25E-03	6.87E-03	--	--
EVM0002708	24.06	59.80	-1.31	5.00E-05	4.28E-04	Function unknown	Ima1 N-terminal domain
EVM0004959	166.73	413.55	-1.31	5.00E-05	4.28E-04	General function prediction only	PQ loop repeat
EVM0007827	96.01	238.04	-1.31	5.00E-05	4.28E-04	--	Lipase (class 3)
EVM0005244	144.94	359.20	-1.31	5.00E-05	4.28E-04	Function unknown	Polysaccharide biosynthesis
EVM0005693	36.13	89.55	-1.31	5.00E-05	4.28E-04	--	--
EVM0011601	125.16	310.07	-1.31	5.00E-05	4.28E-04	--	--
EVM0002871	17.01	42.12	-1.31	5.00E-05	4.28E-04	--	--

EVM0007897	0.84	2.09	-1.31	1.32E-02	4.67E-02	--	F-box-like
EVM0000773	426.10	1051.07	-1.30	1.00E-04	7.96E-04	Energy production and conversion	NADH-ubiquinone oxidoreductase B18 subunit (NDUFB7)
EVM0011359	203.43	501.15	-1.30	5.00E-05	4.28E-04	Energy production and conversion	ETC complex I subunit conserved region
EVM0008632	6.89	16.97	-1.30	9.50E-04	5.46E-03	Posttranslational modification, protein turnover, chaperones	Isoprenylcysteine carboxyl methyltransferase (ICMT) family
EVM0010389	75.11	184.74	-1.30	1.90E-03	9.73E-03	Intracellular trafficking, secretion, and vesicular transport	Tim10/DDP family zinc finger
EVM0008917	19.92	48.96	-1.30	4.50E-04	2.92E-03	General function prediction only	Fcf1
EVM0000800	309.62	760.97	-1.30	5.50E-04	3.46E-03	--	Protein of unknown function (DUF4449)
EVM0001054	21.78	53.52	-1.30	5.00E-05	4.28E-04	--	--
EVM0005431	8.59	21.08	-1.30	5.00E-05	4.28E-04	Cell cycle control, cell division, chromosome partitioning	HEC/Ndc80p family
EVM0002558	11.05	27.09	-1.29	5.00E-05	4.28E-04	Cell cycle control, cell division, chromosome partitioning	Protein kinase domain

EVM0006314	4.06	9.95	-1.29	2.50E-04	1.77E-03	--	--
EVM0010229	28.14	68.91	-1.29	5.00E-05	4.28E-04	General function prediction only	Zinc-binding dehydrogenase
EVM0004513	76.28	186.79	-1.29	2.50E-04	1.77E-03	--	pfkB family carbohydrate kinase
EVM0007883	170.76	417.69	-1.29	5.00E-05	4.28E-04	--	--
EVM0002542	183.23	448.04	-1.29	5.00E-05	4.28E-04	Lipid transport and metabolism	AMP-binding enzyme
EVM0012070	1068.93	2610.87	-1.29	7.00E-04	4.23E-03	--	RNA recognition motif. (a.k.a. RRM, RBD, or RNP domain)
EVM0005236	407.49	994.97	-1.29	5.00E-05	4.28E-04	RNA processing and modification	LSM domain
EVM0004626	15.47	37.76	-1.29	1.00E-04	7.96E-04	--	--
EVM0007325	5.68	13.86	-1.29	7.50E-04	4.49E-03	General function prediction only	3'-5' exonuclease
EVM0000313	13.11	31.99	-1.29	1.00E-04	7.96E-04	Translation, ribosomal structure and biogenesis	RF-1 domain
EVM0007121	10.59	25.83	-1.29	1.00E-04	7.96E-04	--	--
EVM0005913	104.35	254.38	-1.29	5.00E-05	4.28E-04	--	Phosphoribosyl transferase domain
EVM0004405	1.91	4.66	-1.29	6.40E-03	2.62E-02	--	--

EVM0006666	48.95	119.20	-1.28	5.00E-05	4.28E-04	General function prediction only	Homeobox domain
EVM0011169	57.94	140.33	-1.28	5.00E-05	4.28E-04	Posttranslational modification, protein turnover, chaperones	ATPase family associated with various cellular activities (AAA)
EVM0004319	32.83	79.47	-1.28	5.00E-05	4.28E-04	--	Protein of unknown function (DUF2962)
EVM0008404	15.89	38.43	-1.27	5.00E-05	4.28E-04	RNA processing and modification	BING4CT (NUC141) domain
EVM0005282	514.01	1242.20	-1.27	3.50E-04	2.36E-03	Posttranslational modification, protein turnover, chaperones	Nucleotide exchange factor Fes1
EVM0000907	2.79	6.73	-1.27	2.25E-03	1.12E-02	--	--
EVM0002826	98.65	237.84	-1.27	5.00E-05	4.28E-04	General function prediction only	Cell differentiation family, Rcd1-like
EVM0012262	10.72	25.85	-1.27	5.50E-04	3.46E-03	Transcription	SHS2 domain found in N terminus of Rpb7p/Rpc25p/MJ0397
EVM0004038	1024.34	2468.78	-1.27	2.00E-03	1.01E-02	Translation, ribosomal structure and biogenesis	Ribosomal protein L6
EVM0000630	17.98	43.33	-1.27	5.00E-05	4.28E-04	--	--
EVM0007834	40.39	97.29	-1.27	5.00E-05	4.28E-04	--	ADP-ribosylglycohydrolase

EVM0000425	777.91	1873.10	-1.27	5.00E-05	4.28E-04	Intracellular trafficking, secretion, and vesicular transport	TB2/DP1, HVA22 family
EVM0002402	3.95	9.51	-1.27	3.20E-03	1.49E-02	--	Domain of unknown function (DUF3328)
EVM0008872	2.84	6.82	-1.27	5.15E-03	2.20E-02	Lipid transport and metabolism	Fatty acid hydroxylase superfamily
EVM0003967	119.12	286.42	-1.27	1.00E-04	7.96E-04	Function unknown	Mago binding
EVM0007965	22.80	54.81	-1.27	1.00E-04	7.96E-04	Energy production and conversion	Mitochondrial carrier protein
EVM0007303	215.46	517.68	-1.26	5.00E-05	4.28E-04	Intracellular trafficking, secretion, and vesicular transport	PRELI-like family
EVM0009047	17.78	42.69	-1.26	1.00E-04	7.96E-04	General function prediction only	WD domain, G-beta repeat
EVM0001074	75.66	181.69	-1.26	5.00E-05	4.28E-04	Function unknown	Membrane transport protein
EVM0008759	21.69	52.08	-1.26	7.30E-03	2.91E-02	--	--
EVM0000029	14.67	35.22	-1.26	1.00E-04	7.96E-04	Transcription	RNA polymerase Rpb3/RpoA insert domain
EVM0007555	23.12	55.51	-1.26	5.00E-05	4.28E-04	--	--
EVM0011492	336.75	808.34	-1.26	5.00E-05	4.28E-04	--	--

EVM0002416	136.15	326.79	-1.26	1.50E-04	1.14E-03	Translation, ribosomal structure and biogenesis	Ribosomal protein S16
EVM0006106	17.77	42.66	-1.26	5.00E-05	4.28E-04	--	--
EVM0009334	15.69	37.67	-1.26	5.00E-05	4.28E-04	--	--
EVM0000705	630.14	1512.21	-1.26	5.00E-05	4.28E-04	Inorganic ion transport and metabolism	Iron/manganese superoxide dismutases, C-terminal domain
EVM0007607	140.17	336.34	-1.26	5.00E-05	4.28E-04	General function prediction only	TPR repeat
EVM0001314	79.24	190.04	-1.26	5.00E-05	4.28E-04	--	--
EVM0002044	94.18	225.88	-1.26	5.00E-05	4.28E-04	Translation, ribosomal structure and biogenesis	Initiation factor 2 subunit family
EVM0001141	8.15	19.54	-1.26	2.20E-03	1.10E-02	--	--
EVM0010493	10.22	24.50	-1.26	3.20E-03	1.49E-02	--	--
EVM0011608	874.64	2092.67	-1.26	1.50E-04	1.14E-03	Translation, ribosomal structure and biogenesis	Ribosomal protein L24e
EVM0006616	1026.24	2454.15	-1.26	6.00E-04	3.72E-03	Energy production and conversion	Mitochondrial carrier protein
EVM0007953	326.55	780.62	-1.26	2.00E-04	1.46E-03	--	--
EVM0010259	825.94	1973.39	-1.26	1.00E-04	7.96E-04	--	Domain of unknown function (DUF427)

EVM0011272	2.35	5.61	-1.26	2.65E-03	1.28E-02	--	--
EVM0008626	40.43	96.42	-1.25	5.00E-05	4.28E-04	--	--
EVM0009512	32.12	76.60	-1.25	5.00E-05	4.28E-04	Lipid transport and metabolism	CoA-transferase family III
EVM0007418	43.76	104.37	-1.25	5.00E-05	4.28E-04	Carbohydrate transport and metabolism	Protein kinase domain
EVM0009341	10.40	24.81	-1.25	1.50E-04	1.14E-03	RNA processing and modification	DEAD/DEAH box helicase
EVM0001538	13.36	31.86	-1.25	5.00E-05	4.28E-04	--	F-box-like
EVM0010217	48.18	114.86	-1.25	1.50E-04	1.14E-03	--	Domain of unknown function (DUF4139)
EVM0009858	33.92	80.81	-1.25	5.00E-05	4.28E-04	Translation, ribosomal structure and biogenesis	CPL (NUC119) domain
EVM0011612	35.03	83.44	-1.25	5.00E-05	4.28E-04	--	--
EVM0005496	390.23	929.14	-1.25	2.00E-04	1.46E-03	--	--
EVM0002811	323.94	770.52	-1.25	5.50E-04	3.46E-03	Posttranslational modification, protein turnover, chaperones	Thioredoxin
EVM0000189	6.27	14.90	-1.25	1.20E-03	6.64E-03	--	Glycosyl hydrolases family 43

EVM0007568	127.56	303.20	-1.25	5.00E-05	4.28E-04	--	ACT domain
EVM0001635	109.76	260.62	-1.25	5.00E-05	4.28E-04	Posttranslational modification, protein turnover, chaperones	GrpE
EVM0006578	98.13	233.00	-1.25	5.00E-05	4.28E-04	Intracellular trafficking, secretion, and vesicular transport	Syntaxin
EVM0006768	12.12	28.76	-1.25	5.00E-05	4.28E-04	Secondary metabolites biosynthesis, transport and catabolism	Cytochrome P450
EVM0003042	10.81	25.63	-1.25	1.00E-04	7.96E-04	--	--
EVM0004668	149.04	353.19	-1.24	2.50E-04	1.77E-03	General function prediction only	Proteolipid membrane potential modulator
EVM0008633	34.63	82.00	-1.24	5.00E-05	4.28E-04	Intracellular trafficking, secretion, and vesicular transport	Autophagy-related protein 13
EVM0001657	546.77	1294.13	-1.24	3.00E-04	2.07E-03	--	--
EVM0000762	150.34	355.64	-1.24	5.00E-05	4.28E-04	General function prediction only	Protein of unknown function (DUF1295)
EVM0000913	29.90	70.72	-1.24	1.50E-04	1.14E-03	--	--
EVM0011593	7.13	16.86	-1.24	3.00E-04	2.07E-03	Carbohydrate transport and metabolism	Major Facilitator Superfamily

EVM0003932	90.21	213.30	-1.24	1.00E-04	7.96E-04	--	--
EVM0007596	21.58	51.00	-1.24	5.00E-05	4.28E-04	General function prediction only	RNA recognition motif. (a.k.a. RRM, RBD, or RNP domain)
EVM0011840	17.63	41.60	-1.24	5.00E-05	4.28E-04	--	--
EVM0005550	115.76	272.77	-1.24	5.00E-05	4.28E-04	--	50S ribosome-binding GTPase
EVM0008995	28.86	67.95	-1.24	5.00E-05	4.28E-04	--	Arrestin (or S-antigen), C-terminal domain
EVM0005197	35.23	82.94	-1.24	5.00E-05	4.28E-04	General function prediction only	Major Facilitator Superfamily
EVM0005877	682.84	1607.28	-1.24	5.00E-05	4.28E-04	Energy production and conversion	Ubiquinol-cytochrome C reductase complex 14kD subunit
EVM0006994	2.05	4.83	-1.23	2.70E-03	1.30E-02	--	--
EVM0011577	86.47	203.24	-1.23	5.00E-05	4.28E-04	--	ER membrane protein SH3
EVM0005200	51.03	119.93	-1.23	5.00E-05	4.28E-04	Lipid transport and metabolism	Alpha/beta hydrolase family
EVM0008327	7.67	18.01	-1.23	1.00E-04	7.96E-04	--	--
EVM0005644	43.81	102.81	-1.23	5.00E-05	4.28E-04	Lipid transport and metabolism	Phospholipid methyltransferase
EVM0011536	52.19	122.39	-1.23	5.00E-05	4.28E-04	--	Protein of unknown function (DUF3445)

EVM0009748	20.75	48.64	-1.23	4.50E-04	2.92E-03	--	Endoplasmic reticulum-based factor for assembly of V-ATPase
EVM0004503	2.96	6.94	-1.23	2.55E-03	1.24E-02	--	--
EVM0004135	14.64	34.31	-1.23	5.00E-05	4.28E-04	--	Aconitase family (aconitate hydratase)
EVM0004782	28.37	66.49	-1.23	1.00E-04	7.96E-04	--	Tyrosine phosphatase family
EVM0002657	408.67	957.32	-1.23	1.00E-04	7.96E-04	General function prediction only	short chain dehydrogenase
EVM0004345	20.90	48.94	-1.23	1.50E-04	1.14E-03	--	Taurine catabolism dioxygenase TauD, TfdA family
EVM0000893	18.30	42.83	-1.23	7.00E-04	4.23E-03	--	--
EVM0010862	90.28	211.19	-1.23	5.00E-05	4.28E-04	Translation, ribosomal structure and biogenesis	Ribosomal protein L24e
EVM0009651	4.96	11.60	-1.23	2.50E-04	1.77E-03	Energy production and conversion	FAD binding domain
EVM0011153	189.03	441.33	-1.22	5.00E-05	4.28E-04	--	Tim17/Tim22/Tim23/Pmp24 family
EVM0000143	5.32	12.41	-1.22	1.00E-04	7.96E-04	--	Pectate lyase
EVM0012211	24.74	57.71	-1.22	5.00E-05	4.28E-04	--	--
EVM0006852	41.99	97.85	-1.22	5.00E-04	3.19E-03	--	Sen15 protein

EVM0006461	6.93	16.14	-1.22	8.50E-04	4.98E-03	Lipid transport and metabolism	Acyltransferase
EVM0010213	1148.45	2672.67	-1.22	5.70E-03	2.39E-02	--	--
EVM0002814	131.54	305.86	-1.22	1.00E-04	7.96E-04	Intracellular trafficking, secretion, and vesicular transport	Peroxisomal membrane protein (Pex16)
EVM0003201	1421.95	3304.21	-1.22	1.00E-04	7.96E-04	--	Mitochondrial ATP synthase epsilon chain
EVM0009471	17.90	41.55	-1.22	9.00E-04	5.22E-03	--	--
EVM0008951	29.06	67.44	-1.21	5.00E-05	4.28E-04	Intracellular trafficking, secretion, and vesicular transport	Tetratricopeptide repeat
EVM0004301	22.59	52.40	-1.21	5.00E-05	4.28E-04	Secondary metabolites biosynthesis, transport and catabolism	Pyridine nucleotide-disulphide oxidoreductase
EVM0002975	522.13	1211.01	-1.21	2.35E-03	1.16E-02	--	Putative stress-responsive nuclear envelope protein
EVM0011618	111.48	258.49	-1.21	2.00E-04	1.46E-03	--	--
EVM0000116	5.76	13.35	-1.21	5.10E-03	2.19E-02	--	--
EVM0009698	1174.02	2720.55	-1.21	5.00E-05	4.28E-04	Translation, ribosomal structure and biogenesis	Ribosomal L38e protein family

EVM0012235	479.41	1110.66	-1.21	2.00E-04	1.46E-03	Posttranslational modification, protein turnover, chaperones	Cyclophilin type peptidyl-prolyl cis-trans isomerase/CLD
EVM0007682	61.28	141.89	-1.21	1.10E-03	6.17E-03	--	--
EVM0010549	13.82	31.98	-1.21	5.00E-05	4.28E-04	--	--
EVM0002205	1.74	4.03	-1.21	1.70E-03	8.87E-03	--	--
EVM0001189	42.53	98.39	-1.21	5.00E-05	4.28E-04	Chromatin structure and dynamics	Histone deacetylase domain
EVM0005130	13.69	31.65	-1.21	2.00E-04	1.46E-03	--	--
EVM0000714	287.33	664.41	-1.21	5.00E-05	4.28E-04	Translation, ribosomal structure and biogenesis	Ribosomal protein S7p/S5e
EVM0008508	7.71	17.83	-1.21	1.80E-03	9.29E-03	--	--
EVM0007602	10.24	23.67	-1.21	1.50E-04	1.14E-03	Lipid transport and metabolism	PLD-like domain
EVM0010922	220.18	508.81	-1.21	1.00E-04	7.96E-04	--	Nitronate monooxygenase
EVM0009839	185.50	428.35	-1.21	1.15E-03	6.41E-03	Carbohydrate transport and metabolism	Glycosyltransferase family 20
EVM0011785	5.92	13.65	-1.21	5.00E-05	4.28E-04	--	Fungal specific transcription factor domain

EVM0007562	22.06	50.89	-1.21	5.50E-04	3.46E-03	--	--
EVM0007412	330.14	760.69	-1.20	2.00E-04	1.46E-03	--	Domain of unknown function (DUF202)
EVM0004906	162.63	374.56	-1.20	1.50E-04	1.14E-03	--	Fungal specific transcription factor domain
EVM0009107	2.30	5.30	-1.20	8.90E-03	3.41E-02	--	--
EVM0001013	1.27	2.91	-1.20	1.27E-02	4.53E-02	--	--
EVM0005135	11.96	27.52	-1.20	5.00E-05	4.28E-04	Secondary metabolites biosynthesis, transport and catabolism	Cytochrome P450
EVM0010148	173.26	398.39	-1.20	1.00E-04	7.96E-04	--	--
EVM0011121	14.51	33.34	-1.20	1.15E-03	6.41E-03	--	--
EVM0002805	128.92	295.79	-1.20	1.00E-04	7.96E-04	Intracellular trafficking, secretion, and vesicular transport	Translocation protein Sec62
EVM0003106	20.47	46.90	-1.20	5.00E-05	4.28E-04	--	Spt20 family
EVM0008959	218.04	499.13	-1.19	5.00E-05	4.28E-04	General function prediction only	Phosphatidylethanolamine-binding protein

EVM0011835	78.75	180.24	-1.19	5.00E-05	4.28E-04	Posttranslational modification, protein turnover, chaperones	Ring finger domain
EVM0001453	2.06	4.72	-1.19	7.45E-03	2.96E-02	--	--
EVM0002160	4.11	9.40	-1.19	1.28E-02	4.54E-02	--	--
EVM0002217	170.46	390.05	-1.19	5.00E-05	4.28E-04	--	--
EVM0002681	12.43	28.42	-1.19	5.00E-05	4.28E-04	General function prediction only	Glycosyl transferase family 90
EVM0005012	4.61	10.52	-1.19	1.00E-03	5.70E-03	General function prediction only	Calcineurin-like phosphoesterase
EVM0003649	56.42	128.87	-1.19	5.00E-05	4.28E-04	General function prediction only	Thioesterase superfamily
EVM0006430	6.17	14.10	-1.19	8.50E-04	4.98E-03	--	--
EVM0004971	13.07	29.85	-1.19	1.50E-04	1.14E-03	#N/A	#N/A
EVM0007693	21.44	48.95	-1.19	2.50E-04	1.77E-03	--	--
EVM0003055	220.55	503.41	-1.19	1.00E-04	7.96E-04	Function unknown	NUDIX domain
EVM0007328	1376.96	3142.71	-1.19	5.00E-05	4.28E-04	Translation, ribosomal structure and biogenesis	60s Acidic ribosomal protein

EVM0002176	41.57	94.85	-1.19	5.00E-05	4.28E-04	--	--
EVM0001875	8.80	20.09	-1.19	7.00E-04	4.23E-03	--	Sgf11 (transcriptional regulation protein)
EVM0006624	21.18	48.23	-1.19	5.00E-05	4.28E-04	Amino acid transport and metabolism	Pyridoxal-phosphate dependent enzyme
EVM0007006	135.54	308.61	-1.19	2.00E-04	1.46E-03	Signal transduction mechanisms	CS domain
EVM0006543	3.63	8.27	-1.19	4.00E-04	2.65E-03	--	--
EVM0003844	12.89	29.31	-1.19	1.50E-04	1.14E-03	Function unknown	--
EVM0007377	11.94	27.15	-1.18	1.00E-04	7.96E-04	RNA processing and modification	RNA pseudouridylate synthase
EVM0010407	10.81	24.57	-1.18	1.50E-04	1.14E-03	--	Putative S-adenosyl-L-methionine-dependent methyltransferase
EVM0010348	13.48	30.63	-1.18	1.15E-03	6.41E-03	--	--
EVM0009717	975.88	2217.50	-1.18	7.65E-03	3.02E-02	--	--
EVM0011555	79.25	179.98	-1.18	5.00E-05	4.28E-04	--	Sel1 repeat
EVM0011056	58.60	133.05	-1.18	1.00E-04	7.96E-04	--	--

EVM0009100	19.30	43.82	-1.18	5.00E-05	4.28E-04	--	--
EVM0000616	98.02	222.47	-1.18	3.00E-04	2.07E-03	--	SNARE domain
EVM0011007	9.02	20.47	-1.18	6.00E-04	3.72E-03	--	--
EVM0002693	57.78	131.06	-1.18	7.50E-04	4.49E-03	--	--
EVM0006895	2.96	6.71	-1.18	1.37E-02	4.80E-02	--	Protein of unknown function (DUF3129)
EVM0011806	609.02	1380.14	-1.18	1.50E-04	1.14E-03	Translation, ribosomal structure and biogenesis	Ribosomal protein L18e/L15
EVM0009693	23.34	52.87	-1.18	5.00E-05	4.28E-04	Function unknown	--
EVM0006869	465.42	1054.10	-1.18	5.00E-05	4.28E-04	General function prediction only	Ras family
EVM0007905	19.62	44.43	-1.18	2.50E-04	1.77E-03	--	Spc19
EVM0005126	3.47	7.86	-1.18	1.25E-03	6.87E-03	--	--
EVM0009339	40.77	92.24	-1.18	2.00E-04	1.46E-03	Lipid transport and metabolism	Emopamil binding protein
EVM0012102	18.46	41.74	-1.18	2.00E-04	1.46E-03	--	--
EVM0003678	4.71	10.64	-1.18	8.00E-04	4.73E-03	--	FAD binding domain

EVM0007593	108.89	246.16	-1.18	1.00E-04	7.96E-04	RNA processing and modification	Zinc knuckle
EVM0004621	425.01	959.86	-1.18	7.00E-04	4.23E-03	--	BTB/POZ domain
EVM0001584	39.66	89.56	-1.18	5.00E-05	4.28E-04	--	Voltage-dependent anion channel
EVM0001050	47.15	106.31	-1.17	5.00E-05	4.28E-04	--	CybS
EVM0009451	8.84	19.92	-1.17	5.00E-05	4.28E-04	--	NYN domain
EVM0001437	18.61	41.96	-1.17	1.00E-04	7.96E-04	RNA processing and modification	pre-mRNA processing factor 3 (PRP3)
EVM0010485	20.49	46.17	-1.17	5.00E-05	4.28E-04	--	Cytochrome P450
EVM0000405	170.34	383.71	-1.17	2.50E-04	1.77E-03	--	50S ribosome-binding GTPase
EVM0009183	20.56	46.32	-1.17	5.00E-05	4.28E-04	--	BTB/POZ domain
EVM0010534	61.68	138.88	-1.17	5.00E-05	4.28E-04	--	--
EVM0003262	32.19	72.49	-1.17	5.00E-05	4.28E-04	--	Ribonuclease-III-like
EVM0008219	158.43	356.68	-1.17	5.00E-05	4.28E-04	--	Hypothetical protein FLILHELTA
EVM0005225	12.95	29.16	-1.17	1.00E-04	7.96E-04	--	Gti1/Pac2 family

EVM0009840	17.24	38.79	-1.17	5.00E-05	4.28E-04	--	BUD22
EVM0011144	64.29	144.61	-1.17	5.00E-05	4.28E-04	--	Protein of unknown function (DUF2034)
EVM0000836	149.61	336.46	-1.17	1.00E-04	7.96E-04	Intracellular trafficking, secretion, and vesicular transport	WD domain, G-beta repeat
EVM0000596	6.95	15.62	-1.17	8.00E-04	4.73E-03	Replication, recombination and repair	--
EVM0012127	18.30	41.10	-1.17	5.00E-05	4.28E-04	--	PXA domain
EVM0000042	11.08	24.86	-1.17	3.20E-03	1.49E-02	--	--
EVM0008789	1922.75	4314.63	-1.17	3.00E-04	2.07E-03	Energy production and conversion	Cytochrome c oxidase subunit VIa
EVM0008292	16.60	37.24	-1.17	5.00E-05	4.28E-04	Posttranslational modification, protein turnover, chaperones	IBR domain
EVM0006361	43.55	97.72	-1.17	2.00E-04	1.46E-03	--	--
EVM0006257	158.73	355.70	-1.16	5.00E-05	4.28E-04	--	--
EVM0006015	36.17	80.99	-1.16	5.00E-05	4.28E-04	--	MYND finger
EVM0001571	17.76	39.75	-1.16	1.50E-04	1.14E-03	--	--

EVM0008946	83.52	186.91	-1.16	1.00E-04	7.96E-04	Posttranslational modification, protein turnover, chaperones	Ring finger domain
EVM0011691	12.75	28.52	-1.16	2.00E-04	1.46E-03	Function unknown	Regulator of chromosome condensation (RCC1) repeat
EVM0011563	73.05	163.37	-1.16	5.00E-05	4.28E-04	Posttranslational modification, protein turnover, chaperones	X-domain of DnaJ-containing
EVM0012134	16.86	37.64	-1.16	5.00E-05	4.28E-04	Energy production and conversion	Phosphoenolpyruvate phosphomutase
EVM0007136	30.35	67.77	-1.16	5.00E-05	4.28E-04	--	--
EVM0006468	16.56	36.95	-1.16	1.50E-04	1.14E-03	Signal transduction mechanisms	RED-like protein N-terminal region
EVM0003787	67.47	150.51	-1.16	1.00E-04	7.96E-04	Translation, ribosomal structure and biogenesis	Eukaryotic translation initiation factor 3 subunit 7 (eIF-3)
EVM0010785	57.46	128.11	-1.16	1.00E-04	7.96E-04	--	Rpp20 subunit of nuclear RNase MRP and P
EVM0006446	124.25	276.59	-1.15	1.00E-04	7.96E-04	--	FR47-like protein
EVM0005171	14.80	32.94	-1.15	5.00E-05	4.28E-04	Replication, recombination and repair	DNA mismatch repair protein, C-terminal domain
EVM0000274	23.30	51.81	-1.15	5.00E-05	4.28E-04	General function prediction only	Protein kinase domain
EVM0007735	0.82	1.81	-1.15	4.40E-03	1.94E-02	--	--

EVM0009577	23.71	52.69	-1.15	5.00E-05	4.28E-04	General function prediction only	GTP1/OBG
EVM0002709	4.53	10.07	-1.15	9.50E-04	5.46E-03	--	--
EVM0005506	6.00	13.32	-1.15	1.80E-03	9.29E-03	#N/A	#N/A
EVM0005034	745.33	1655.45	-1.15	6.50E-04	3.98E-03	--	--
EVM0005665	31.55	70.07	-1.15	5.00E-05	4.28E-04	General function prediction only	Zinc finger, C2H2 type
EVM0003335	36.17	80.20	-1.15	4.50E-04	2.92E-03	--	Eukaryotic protein of unknown function (DUF1764)
EVM0007292	5.42	12.00	-1.15	7.00E-04	4.23E-03	General function prediction only	Domain of unknown function (DUF4110)
EVM0011416	180.47	399.53	-1.15	2.00E-04	1.46E-03	Cell cycle control, cell division, chromosome partitioning	Cdc37 N terminal kinase binding
EVM0005357	5.85	12.96	-1.15	1.25E-03	6.87E-03	--	--
EVM0005470	7.89	17.45	-1.15	1.00E-04	7.96E-04	--	F-box-like
EVM0000575	80.89	178.99	-1.15	4.50E-04	2.92E-03	--	--
EVM0008621	9.11	20.16	-1.15	1.60E-03	8.44E-03	--	--

EVM0006672	33.15	73.26	-1.14	5.00E-05	4.28E-04	--	Peroxidase, family 2
EVM0002128	159.69	352.68	-1.14	2.50E-04	1.77E-03	Translation, ribosomal structure and biogenesis	Domain found in IF2B/IF5
EVM0000424	1026.55	2266.44	-1.14	3.90E-03	1.76E-02	--	Protein of unknown function (DUF3421)
EVM0004054	437.54	965.46	-1.14	3.50E-04	2.36E-03	Posttranslational modification, protein turnover, chaperones	Hsp20/alpha crystallin family
EVM0006805	133.31	294.00	-1.14	5.00E-05	4.28E-04	Lipid transport and metabolism	Alpha/beta hydrolase family
EVM0006502	191.15	421.30	-1.14	1.00E-04	7.96E-04	Secondary metabolites biosynthesis, transport and catabolism	Alcohol dehydrogenase GroES-like domain
EVM0008653	23.03	50.74	-1.14	2.50E-04	1.77E-03	--	--
EVM0008337	25.78	56.76	-1.14	1.00E-04	7.96E-04	General function prediction only	--
EVM0004636	91.57	201.60	-1.14	5.00E-04	3.19E-03	General function prediction only	RNA recognition motif. (a.k.a. RRM, RBD, or RNP domain)
EVM0000880	20.12	44.27	-1.14	3.50E-04	2.36E-03	--	Glycosyl hydrolases family 15
EVM0003015	7.65	16.82	-1.14	1.50E-03	8.00E-03	--	RTA1 like protein
EVM0007304	38.00	83.57	-1.14	1.00E-04	7.96E-04	Carbohydrate transport and metabolism	UAA transporter family

EVM0011509	69.60	153.01	-1.14	5.00E-05	4.28E-04	--	--
EVM0009609	47.68	104.61	-1.13	3.00E-04	2.07E-03	Signal transduction mechanisms	RhoGEF domain
EVM0006633	4.50	9.87	-1.13	1.00E-04	7.96E-04	--	--
EVM0010495	50.38	110.41	-1.13	1.50E-04	1.14E-03	--	Protein kinase domain
EVM0005366	24.96	54.67	-1.13	2.50E-04	1.77E-03	Function unknown	pre-RNA processing PIH1/Nop17
EVM0000547	6.32	13.83	-1.13	6.50E-04	3.98E-03	--	--
EVM0010071	3.72	8.15	-1.13	2.50E-03	1.22E-02	--	--
EVM0002024	5.96	13.05	-1.13	2.05E-03	1.03E-02	Secondary metabolites biosynthesis, transport and catabolism	short chain dehydrogenase
EVM0010774	1934.73	4233.52	-1.13	8.50E-04	4.98E-03	Posttranslational modification, protein turnover, chaperones	Cyclophilin type peptidyl-prolyl cis-trans isomerase/CLD
EVM0005546	21.20	46.39	-1.13	3.00E-04	2.07E-03	General function prediction only	MatE
EVM0006308	28.10	61.37	-1.13	3.45E-03	1.59E-02	--	--
EVM0007375	16.76	36.60	-1.13	1.00E-04	7.96E-04	General function prediction only	HIT zinc finger

EVM0004217	20.64	45.04	-1.13	5.00E-04	3.19E-03	Replication, recombination and repair	--
EVM0004398	338.58	738.53	-1.13	1.50E-04	1.14E-03	General function prediction only	Proteolipid membrane potential modulator
EVM0010575	20.51	44.72	-1.12	5.00E-05	4.28E-04	--	--
EVM0001181	220.10	479.56	-1.12	1.00E-04	7.96E-04	Intracellular trafficking, secretion, and vesicular transport	Regulated-SNARE-like domain
EVM0007991	70.78	154.07	-1.12	5.00E-05	4.28E-04	Transcription	SAC3/GANP/Nin1/mts3/eIF-3 p25 family
EVM0005306	52.40	114.02	-1.12	1.00E-04	7.96E-04	Cell wall/membrane/envelope biogenesis	Glycosyl transferases group 1
EVM0002257	30.87	67.12	-1.12	1.00E-04	7.96E-04	--	--
EVM0009686	163.03	354.07	-1.12	5.00E-05	4.28E-04	Transcription	Spt4/RpoE2 zinc finger
EVM0003157	547.66	1189.32	-1.12	2.50E-04	1.77E-03	Defense mechanisms	B-cell receptor-associated protein 31-like
EVM0004197	4.84	10.50	-1.12	9.05E-03	3.46E-02	--	MYND finger
EVM0006677	23.91	51.92	-1.12	1.65E-03	8.65E-03	--	--
EVM0002618	60.53	131.33	-1.12	1.50E-04	1.14E-03	Translation, ribosomal structure and biogenesis	TruB family pseudouridylate synthase (N terminal domain)

EVM0002834	326.36	707.76	-1.12	1.40E-03	7.55E-03	--	Zinc-binding dehydrogenase
EVM0002551	24.13	52.33	-1.12	1.00E-04	7.96E-04	Translation, ribosomal structure and biogenesis	Nop14-like family
EVM0010966	10.39	22.51	-1.12	5.50E-04	3.46E-03	Replication, recombination and repair	ATPase family associated with various cellular activities (AAA)
EVM0007623	34.93	75.70	-1.12	8.00E-04	4.73E-03	--	ribonuclease
EVM0011961	145.44	315.05	-1.12	5.00E-05	4.28E-04	General function prediction only	Zinc finger C-x8-C-x5-C-x3-H type (and similar)
EVM0003805	3.36	7.27	-1.12	1.20E-03	6.64E-03	Transcription	HMG (high mobility group) box
EVM0007576	23.10	50.04	-1.12	1.00E-04	7.96E-04	Transcription	NLI interacting factor-like phosphatase
EVM0003465	23.72	51.36	-1.11	7.50E-04	4.49E-03	--	--
EVM0010315	5.62	12.16	-1.11	2.00E-03	1.01E-02	--	50S ribosome-binding GTPase
EVM0000366	3.30	7.14	-1.11	5.65E-03	2.37E-02	--	--
EVM0011527	1670.30	3615.71	-1.11	7.50E-04	4.49E-03	Signal transduction mechanisms	EF hand
EVM0008532	125.24	271.09	-1.11	3.50E-04	2.36E-03	Translation, ribosomal structure and biogenesis	Ribosomal L27 protein
EVM0006117	14.98	32.42	-1.11	6.00E-04	3.72E-03	General function prediction only	Aldo/keto reductase family

EVM0001473	25.08	54.27	-1.11	5.50E-04	3.46E-03	Intracellular trafficking, secretion, and vesicular transport	Transport protein particle (TRAPP) component
EVM0006601	18.95	40.99	-1.11	5.00E-05	4.28E-04	General function prediction only	Zinc finger, C2H2 type
EVM0002908	640.34	1384.52	-1.11	2.00E-04	1.46E-03	Posttranslational modification, protein turnover, chaperones	Ubiquitin-conjugating enzyme
EVM0008156	787.31	1701.22	-1.11	4.20E-03	1.87E-02	--	--
EVM0005849	216.66	468.03	-1.11	2.00E-04	1.46E-03	--	--
EVM0006493	122.28	264.14	-1.11	5.00E-05	4.28E-04	--	N-acetylglucosaminyl transferase component (Gpi1)
EVM0011115	5.03	10.87	-1.11	4.00E-03	1.79E-02	--	--
EVM0006692	2.21	4.77	-1.11	1.00E-04	7.96E-04	--	Variant SH3 domain
EVM0005827	33.80	72.93	-1.11	1.00E-04	7.96E-04	--	--
EVM0003684	3.65	7.88	-1.11	6.50E-04	3.98E-03	#N/A	#N/A
EVM0011422	343.49	740.98	-1.11	3.50E-04	2.36E-03	Secondary metabolites biosynthesis, transport and catabolism	Alcohol dehydrogenase GroES-like domain

EVM0002841	114.90	247.68	-1.11	2.50E-04	1.77E-03	General function prediction only	Cyclin
EVM0006547	112.73	242.92	-1.11	5.00E-05	4.28E-04	Translation, ribosomal structure and biogenesis	Ribosomal protein L23
EVM0000928	158.82	342.11	-1.11	1.00E-04	7.96E-04	Function unknown	Der1-like family
EVM0006875	70.15	151.06	-1.11	5.50E-04	3.46E-03	--	Polysaccharide deacetylase
EVM0004577	24.45	52.61	-1.11	2.50E-04	1.77E-03	Cell cycle control, cell division, chromosome partitioning	Cid1 family poly A polymerase
EVM0010742	38.69	83.22	-1.11	5.00E-05	4.28E-04	Posttranslational modification, protein turnover, chaperones	ATP10 protein
EVM0011573	9.89	21.26	-1.10	5.00E-05	4.28E-04	Function unknown	Transcriptional repressor TCF25
EVM0006845	15.45	33.19	-1.10	5.00E-04	3.19E-03	--	SNF5 / SMARCB1 / INI1
EVM0005464	19.25	41.34	-1.10	1.50E-04	1.14E-03	Amino acid transport and metabolism	Gamma-glutamyltranspeptidase
EVM0007966	157.27	337.77	-1.10	4.50E-04	2.92E-03	--	Na+ dependent nucleoside transporter C-terminus
EVM0010581	59.75	128.29	-1.10	3.50E-04	2.36E-03	--	--
EVM0007144	31.92	68.53	-1.10	2.00E-04	1.46E-03	--	F-box-like

EVM0000005	34.87	74.87	-1.10	4.05E-03	1.81E-02	--	--
EVM0004966	3.33	7.14	-1.10	7.00E-04	4.23E-03	--	--
EVM0005401	14.37	30.82	-1.10	2.00E-04	1.46E-03	--	Poly(ADP-ribose) polymerase catalytic domain
EVM0000354	723.39	1550.96	-1.10	1.50E-04	1.14E-03	Translation, ribosomal structure and biogenesis	Ribosomal L29 protein
EVM0007977	313.54	672.08	-1.10	4.00E-04	2.65E-03	--	--
EVM0005544	96.69	207.23	-1.10	1.00E-04	7.96E-04	Translation, ribosomal structure and biogenesis	Ribosomal protein L18e/L15
EVM0000483	165.09	353.73	-1.10	5.00E-05	4.28E-04	Amino acid transport and metabolism	Chorismate mutase type II
EVM0002695	100.88	216.03	-1.10	1.00E-04	7.96E-04	--	SYF2 splicing factor
EVM0001019	1060.48	2270.15	-1.10	1.10E-03	6.17E-03	--	--
EVM0010106	60.11	128.56	-1.10	1.00E-04	7.96E-04	Cell cycle control, cell division, chromosome partitioning	Maf-like protein
EVM0002772	22.54	48.21	-1.10	1.75E-03	9.08E-03	--	--
EVM0008986	109.61	234.29	-1.10	1.50E-04	1.14E-03	Intracellular trafficking, secretion, and vesicular transport	Endoplasmic reticulum vesicle transporter

EVM0001622	251.23	536.99	-1.10	3.00E-04	2.07E-03	--	Ricin-type beta-trefoil lectin domain-like
EVM0011337	2.46	5.26	-1.10	9.90E-03	3.72E-02	--	--
EVM0009958	13.22	28.26	-1.10	1.40E-03	7.55E-03	--	--
EVM0005232	48.20	102.99	-1.10	1.50E-04	1.14E-03	RNA processing and modification	Brix domain
EVM0009904	39.79	84.99	-1.09	2.00E-04	1.46E-03	General function prediction only	Methyltransferase involved in Williams-Beuren syndrome
EVM0004101	58.60	125.16	-1.09	1.50E-04	1.14E-03	Carbohydrate transport and metabolism	Phosphomannose isomerase type I
EVM0004707	31.70	67.70	-1.09	1.00E-04	7.96E-04	--	Alpha-L-rhamnosidase N-terminal domain
EVM0004046	128.45	274.23	-1.09	5.00E-05	4.28E-04	Amino acid transport and metabolism	Pyridoxal-dependent decarboxylase, pyridoxal binding domain
EVM0003024	37.16	79.28	-1.09	5.00E-05	4.28E-04	Signal transduction mechanisms	Rhomboid family
EVM0011858	83.43	177.95	-1.09	2.50E-04	1.77E-03	RNA processing and modification	Ribosomal protein L10
EVM0004495	260.54	555.58	-1.09	2.00E-04	1.46E-03	Translation, ribosomal structure and biogenesis	Ribosomal protein L7Ae/L30e/S12e/Gadd45 family
EVM0010022	190.17	405.42	-1.09	5.00E-04	3.19E-03	--	--

EVM0011621	60.74	129.47	-1.09	3.50E-04	2.36E-03	Function unknown	Low temperature viability protein
EVM0004063	45.47	96.89	-1.09	2.50E-04	1.77E-03	Transcription	RNA recognition motif. (a.k.a. RRM, RBD, or RNP domain)
EVM0000755	137.37	292.54	-1.09	1.50E-04	1.14E-03	--	--
EVM0010288	54.08	115.09	-1.09	5.00E-05	4.28E-04	Inorganic ion transport and metabolism	Cation efflux family
EVM0008206	114.94	244.54	-1.09	5.00E-05	4.28E-04	RNA processing and modification	RNA recognition motif. (a.k.a. RRM, RBD, or RNP domain)
EVM0009109	53.80	114.39	-1.09	1.00E-04	7.96E-04	--	Glycosyl hydrolases family 43
EVM0007564	9.42	20.02	-1.09	3.50E-04	2.36E-03	Transcription	Fork head domain
EVM0009937	312.92	664.17	-1.09	9.50E-04	5.46E-03	Cell wall/membrane/envelope biogenesis	UTP--glucose-1-phosphate uridylyltransferase
EVM0010005	32.97	69.97	-1.09	3.50E-04	2.36E-03	--	PIG-X / PBN1
EVM0008829	32.77	69.49	-1.08	2.00E-04	1.46E-03	--	--
EVM0000690	45.10	95.59	-1.08	1.50E-04	1.14E-03	--	Protein of unknown function (DUF3433)
EVM0004379	228.26	483.53	-1.08	1.50E-04	1.14E-03	Intracellular trafficking, secretion, and vesicular transport	Synaptobrevin

EVM0009036	154.34	326.91	-1.08	5.00E-05	4.28E-04	RNA processing and modification	Ribosomal protein L7Ae/L30e/S12e/Gadd45 family
EVM0002623	10.92	23.11	-1.08	5.00E-05	4.28E-04	--	WD domain, G-beta repeat
EVM0010727	30.48	64.51	-1.08	3.50E-04	2.36E-03	General function prediction only	Alpha/beta hydrolase family
EVM0005044	10.90	23.07	-1.08	2.50E-04	1.77E-03	--	SAC3/GANP/Nin1/mts3/eIF-3 p25 family
EVM0003820	27.23	57.62	-1.08	2.50E-04	1.77E-03	--	--
EVM0009601	7.25	15.34	-1.08	2.95E-03	1.40E-02	--	GDSL-like Lipase/Acylhydrolase
EVM0003386	12.67	26.78	-1.08	1.00E-03	5.70E-03	--	bZIP transcription factor
EVM0008320	4.30	9.09	-1.08	4.50E-04	2.92E-03	--	Nuclear condensing complex subunits, C-term domain
EVM0001335	13.23	27.96	-1.08	1.00E-03	5.70E-03	RNA processing and modification	Ribosomal L28e protein family
EVM0004541	73.84	156.05	-1.08	3.50E-04	2.36E-03	--	--
EVM0010299	14.34	30.28	-1.08	5.00E-05	4.28E-04	--	XRN 5'-3' exonuclease N-terminus
EVM0008485	4.96	10.48	-1.08	5.50E-04	3.46E-03	--	--
EVM0011366	14.56	30.73	-1.08	2.00E-04	1.46E-03	--	SDA1

EVM0003406	118.46	250.02	-1.08	3.00E-04	2.07E-03	--	--
EVM0010983	5.99	12.64	-1.08	4.60E-03	2.01E-02	--	--
EVM0007524	7.41	15.64	-1.08	8.50E-04	4.98E-03	--	Domain of unknown function (DUF4451)
EVM0001586	252.18	532.04	-1.08	5.00E-05	4.28E-04	Function unknown	Eukaryotic family of unknown function (DUF1754)
EVM0000201	27.19	57.34	-1.08	1.00E-04	7.96E-04	--	Fungal specific transcription factor domain
EVM0000289	27.95	58.88	-1.08	5.00E-05	4.28E-04	RNA processing and modification	RNA recognition motif. (a.k.a. RRM, RBD, or RNP domain)
EVM0000682	130.39	274.63	-1.07	1.00E-04	7.96E-04	Carbohydrate transport and metabolism	Glycolipid transfer protein (GLTP)
EVM0002933	52.91	111.39	-1.07	1.00E-04	7.96E-04	--	50S ribosome-binding GTPase
EVM0011112	14.30	30.11	-1.07	1.50E-03	8.00E-03	--	--
EVM0010562	59.23	124.64	-1.07	1.00E-04	7.96E-04	Posttranslational modification, protein turnover, chaperones	DnaJ domain
EVM0000131	5.59	11.76	-1.07	1.02E-02	3.81E-02	--	--
EVM0004589	55.41	116.56	-1.07	2.50E-04	1.77E-03	--	--

EVM0010153	49.57	104.26	-1.07	5.00E-05	4.28E-04	Lipid transport and metabolism	--
EVM0005335	17.23	36.23	-1.07	3.00E-04	2.07E-03	--	F-box-like
EVM0012187	10.51	22.08	-1.07	6.50E-04	3.98E-03	--	Fungal cellulose binding domain
EVM0008989	141.93	298.19	-1.07	2.00E-04	1.46E-03	--	--
EVM0006637	10.70	22.49	-1.07	6.00E-04	3.72E-03	Posttranslational modification, protein turnover, chaperones	Prolyl oligopeptidase family
EVM0001651	13.01	27.31	-1.07	1.35E-03	7.33E-03	Defense mechanisms	alpha/beta hydrolase fold
EVM0011567	407.42	855.25	-1.07	1.20E-03	6.64E-03	--	--
EVM0001135	10.43	21.89	-1.07	5.50E-04	3.46E-03	RNA processing and modification	DEAD/DEAH box helicase
EVM0009815	87.45	183.43	-1.07	3.00E-04	2.07E-03	--	--
EVM0001766	158.59	332.67	-1.07	1.00E-04	7.96E-04	Intracellular trafficking, secretion, and vesicular transport	Signal recognition particle receptor beta subunit
EVM0000207	55.85	117.08	-1.07	1.50E-04	1.14E-03	Energy production and conversion	Mitochondrial carrier protein

EVM0011727	156.17	327.36	-1.07	6.00E-04	3.72E-03	Posttranslational modification, protein turnover, chaperones	Ubiquitin-2 like Rad60 SUMO-like
EVM0011062	195.65	410.00	-1.07	2.00E-04	1.46E-03	Energy production and conversion	lactate/malate dehydrogenase, NAD binding domain
EVM0006114	44.97	94.19	-1.07	3.50E-04	2.36E-03	--	Thiamine pyrophosphate enzyme, N-terminal TPP binding domain
EVM0011604	3.15	6.60	-1.07	4.45E-03	1.96E-02	--	Cellulase (glycosyl hydrolase family 5)
EVM0007837	1876.37	3928.94	-1.07	1.10E-03	6.17E-03	Translation, ribosomal structure and biogenesis	Ribosomal protein S17
EVM0008363	54.23	113.56	-1.07	3.00E-04	2.07E-03	--	--
EVM0004710	186.95	391.17	-1.07	3.00E-04	2.07E-03	--	Cgr1 family
EVM0008761	21.69	45.37	-1.06	2.00E-04	1.46E-03	--	Calcineurin-like phosphoesterase
EVM0007277	590.66	1235.39	-1.06	7.00E-04	4.23E-03	--	--
EVM0003850	93.57	195.53	-1.06	5.00E-05	4.28E-04	--	--
EVM0001494	11.22	23.44	-1.06	5.00E-04	3.19E-03	--	Domain of unknown function (DUF4451)
EVM0001424	459.03	958.71	-1.06	1.80E-03	9.29E-03	Cytoskeleton	Dynein light chain type 1

EVM0001725	16.94	35.37	-1.06	1.50E-04	1.14E-03	RNA processing and modification	AAA domain
EVM0011101	17.55	36.59	-1.06	2.00E-04	1.46E-03	--	Bromodomain
EVM0008497	701.39	1462.32	-1.06	2.30E-03	1.14E-02	Lipid transport and metabolism	MaoC like domain
EVM0000597	40.02	83.42	-1.06	3.50E-04	2.36E-03	General function prediction only	short chain dehydrogenase
EVM0004055	74.59	155.50	-1.06	5.00E-04	3.19E-03	Translation, ribosomal structure and biogenesis	Ribosomal protein S13/S18
EVM0006198	31.93	66.55	-1.06	1.50E-04	1.14E-03	General function prediction only	Protein kinase domain
EVM0004868	18.65	38.84	-1.06	6.50E-04	3.98E-03	--	F-box-like
EVM0011467	52.11	108.53	-1.06	5.00E-05	4.28E-04	Function unknown	YIF1
EVM0010012	11.95	24.89	-1.06	8.70E-03	3.35E-02	--	Formin Homology Region 1
EVM0007916	11.82	24.61	-1.06	3.15E-03	1.47E-02	#N/A	#N/A
EVM0004807	7.43	15.46	-1.06	2.25E-03	1.12E-02	Chromatin structure and dynamics	Histone deacetylase domain
EVM0004689	5.47	11.38	-1.06	1.05E-03	5.94E-03	--	HAUS augmin-like complex subunit 6 N-terminus
EVM0008211	17.24	35.84	-1.06	2.00E-04	1.46E-03	Signal transduction mechanisms	Protein tyrosine kinase

EVM0002262	92.98	193.32	-1.06	4.20E-03	1.87E-02	Chromatin structure and dynamics	Oxoglutarate and iron-dependent oxygenase degradation C-term
EVM0012170	28.71	59.68	-1.06	5.00E-05	4.28E-04	--	--
EVM0004616	794.46	1650.49	-1.05	8.70E-03	3.35E-02	--	--
EVM0002993	10.03	20.82	-1.05	4.50E-04	2.92E-03	--	Spb1 C-terminal domain
EVM0009366	9.21	19.10	-1.05	4.00E-04	2.65E-03	--	--
EVM0005312	1.12	2.31	-1.05	1.23E-02	4.42E-02	--	DNA polymerase subunit Cdc27
EVM0003892	22.33	46.21	-1.05	2.50E-04	1.77E-03	--	--
EVM0011090	463.04	958.32	-1.05	6.00E-04	3.72E-03	Intracellular trafficking, secretion, and vesicular transport	Tim10/DDP family zinc finger
EVM0003936	77.86	161.09	-1.05	1.00E-04	7.96E-04	General function prediction only	TPR repeat
EVM0000531	25.20	52.11	-1.05	1.50E-04	1.14E-03	Intracellular trafficking, secretion, and vesicular transport	Signal recognition particle 9 kDa protein (SRP9)
EVM0010097	2369.14	4899.33	-1.05	6.05E-03	2.51E-02	Posttranslational modification, protein turnover, chaperones	Hsp20/alpha crystallin family

EVM0005019	121.82	251.66	-1.05	1.40E-03	7.55E-03	General function prediction only	Protein kinase domain
EVM0006288	22.48	46.42	-1.05	1.00E-04	7.96E-04	--	--
EVM0007198	210.26	434.22	-1.05	5.00E-04	3.19E-03	--	--
EVM0001525	33.65	69.49	-1.05	1.50E-04	1.14E-03	--	--
EVM0003663	2.20	4.54	-1.04	6.65E-03	2.70E-02	Carbohydrate transport and metabolism	O-Glycosyl hydrolase family 30
EVM0010347	1101.57	2271.49	-1.04	1.50E-04	1.14E-03	--	Hyaluronan / mRNA binding family
EVM0000938	21.00	43.30	-1.04	6.00E-04	3.72E-03	Posttranslational modification, protein turnover, chaperones	Cyclophilin type peptidyl-prolyl cis-trans isomerase/CLD
EVM0001821	4.23	8.73	-1.04	2.30E-03	1.14E-02	Transcription	HMG (high mobility group) box
EVM0002950	3.45	7.10	-1.04	1.04E-02	3.85E-02	--	--
EVM0007339	30.48	62.74	-1.04	7.00E-04	4.23E-03	--	SMP-30/Gluconolaconase/LRE-like region
EVM0007046	6.70	13.80	-1.04	2.80E-03	1.34E-02	--	--
EVM0005710	37.44	77.06	-1.04	2.00E-04	1.46E-03	--	--

EVM0006933	1246.88	2565.92	-1.04	2.45E-03	1.20E-02	Nucleotide transport and metabolism	Nucleoside diphosphate kinase
EVM0004516	20.43	42.01	-1.04	1.45E-03	7.77E-03	--	--
EVM0010781	119.29	245.21	-1.04	3.00E-04	2.07E-03	General function prediction only	Ribosomal protein S8e
EVM0001265	84.23	173.12	-1.04	1.80E-03	9.29E-03	General function prediction only	Sugar (and other) transporter
EVM0009820	121.56	249.75	-1.04	2.00E-04	1.46E-03	--	Acetyltransferase (GNAT) domain
EVM0000252	16.06	32.99	-1.04	1.15E-03	6.41E-03	--	--
EVM0004057	202.66	416.26	-1.04	1.50E-04	1.14E-03	--	Ser-Thr-rich glycosyl-phosphatidyl-inositol-anchored membrane family
EVM0011378	772.08	1585.11	-1.04	4.00E-04	2.65E-03	Translation, ribosomal structure and biogenesis	Eukaryotic elongation factor 5A hypusine, DNA-binding OB fold
EVM0006914	5.67	11.63	-1.04	3.00E-03	1.42E-02	--	--
EVM0009894	52.99	108.71	-1.04	4.00E-04	2.65E-03	--	Fungal specific transcription factor domain
EVM0009777	42.50	87.11	-1.04	3.00E-04	2.07E-03	General function prediction only	Putative methyltransferase
EVM0000820	44.66	91.47	-1.03	6.50E-04	3.98E-03	Posttranslational modification, protein turnover, chaperones	Ubiquitin-conjugating enzyme

EVM0003606	22.52	46.13	-1.03	2.50E-04	1.77E-03	--	--
EVM0007631	22.14	45.32	-1.03	1.50E-04	1.14E-03	--	--
EVM0006843	3.08	6.30	-1.03	5.50E-03	2.32E-02	--	Glycosyl hydrolases family 6
EVM0007556	395.63	809.72	-1.03	1.00E-03	5.70E-03	--	Ser-Thr-rich glycosyl-phosphatidyl-inositol-anchored membrane family
EVM0010682	117.07	239.34	-1.03	4.20E-03	1.87E-02	--	Mitochondrial ribosomal protein L27
EVM0005393	2.12	4.33	-1.03	3.95E-03	1.77E-02	--	--
EVM0002991	34.86	71.17	-1.03	7.00E-04	4.23E-03	--	--
EVM0010235	3.00	6.12	-1.03	5.20E-03	2.22E-02	--	--
EVM0003416	25.30	51.63	-1.03	3.00E-04	2.07E-03	--	--
EVM0011926	18.85	38.47	-1.03	7.00E-04	4.23E-03	--	--
EVM0007668	317.29	647.34	-1.03	7.50E-04	4.49E-03	Energy production and conversion	CHCH domain
EVM0001024	45.88	93.60	-1.03	1.50E-04	1.14E-03	--	Hinge domain of cleavage stimulation factor subunit 2

EVM0002619	23.07	47.06	-1.03	6.50E-04	3.98E-03	Function unknown	Signal peptide peptidase
EVM0011600	19.64	40.06	-1.03	4.50E-04	2.92E-03	Function unknown	WD domain, G-beta repeat
EVM0009946	30.92	63.06	-1.03	3.00E-04	2.07E-03	Transcription	A49-like RNA polymerase I associated factor
EVM0002328	21.40	43.57	-1.03	1.50E-04	1.14E-03	--	--
EVM0007810	5.88	11.98	-1.03	3.60E-03	1.64E-02	--	--
EVM0004828	631.41	1284.93	-1.03	4.00E-04	2.65E-03	Translation, ribosomal structure and biogenesis	Plectin/S10 domain
EVM0007937	53.31	108.35	-1.02	6.50E-04	3.98E-03	Posttranslational modification, protein turnover, chaperones	Zinc finger, C3HC4 type (RING finger)
EVM0000388	40.02	81.32	-1.02	1.50E-03	8.00E-03	--	--
EVM0007840	540.95	1098.84	-1.02	5.50E-04	3.46E-03	Energy production and conversion	Mitochondrial ribosomal protein L51 / S25 / CI-B8 domain
EVM0010936	208.38	423.24	-1.02	4.50E-04	2.92E-03	--	KH domain
EVM0008552	7.70	15.63	-1.02	2.00E-03	1.01E-02	--	--
EVM0005840	28.59	58.06	-1.02	5.40E-03	2.29E-02	--	Sen15 protein

EVM0008697	10.49	21.31	-1.02	3.20E-03	1.49E-02	--	--
EVM0009006	13.97	28.36	-1.02	2.00E-04	1.46E-03	Replication, recombination and repair	FAT domain
EVM0002613	275.98	560.28	-1.02	3.00E-04	2.07E-03	General function prediction only	RNA recognition motif. (a.k.a. RRM, RBD, or RNP domain)
EVM0012234	139.90	283.93	-1.02	5.50E-04	3.46E-03	RNA processing and modification	TAP C-terminal domain
EVM0001831	539.85	1095.62	-1.02	6.00E-04	3.72E-03	Energy production and conversion	ATP synthase, Delta/Epsilon chain, beta-sandwich domain
EVM0006281	24.55	49.82	-1.02	3.00E-04	2.07E-03	Amino acid transport and metabolism	Hydantoinase B/oxoprolinase
EVM0007792	21.30	43.17	-1.02	1.10E-03	6.17E-03	--	--
EVM0010256	1.39	2.82	-1.02	6.55E-03	2.67E-02	General function prediction only	GMC oxidoreductase
EVM0009247	92.83	187.93	-1.02	2.00E-04	1.46E-03	Transcription	NLI interacting factor-like phosphatase
EVM0010552	30.68	62.10	-1.02	1.50E-04	1.14E-03	--	--
EVM0001367	80.41	162.74	-1.02	1.35E-03	7.33E-03	--	--
EVM0006247	96.49	195.23	-1.02	4.00E-04	2.65E-03	General function prediction only	Zinc finger, C2H2 type
EVM0008894	28.54	57.75	-1.02	8.50E-04	4.98E-03	--	Pterin 4 alpha carbinolamine dehydratase

EVM0000602	1309.36	2648.71	-1.02	2.05E-03	1.03E-02	General function prediction only	Ras family
EVM0007184	42.99	86.91	-1.02	5.50E-04	3.46E-03	Function unknown	YjeF-related protein N-terminus
EVM0006702	20.54	41.52	-1.02	6.50E-04	3.98E-03	--	--
EVM0010605	754.98	1525.74	-1.02	1.50E-04	1.14E-03	Translation, ribosomal structure and biogenesis	Ribosomal protein L34e
EVM0011919	27.01	54.58	-1.01	2.30E-03	1.14E-02	--	--
EVM0011630	6.34	12.81	-1.01	2.00E-03	1.01E-02	--	--
EVM0005039	180.88	365.31	-1.01	1.55E-03	8.22E-03	RNA processing and modification	DEAD/DEAH box helicase
EVM0007023	2946.66	5950.91	-1.01	3.00E-04	2.07E-03	--	NADH-ubiquinone reductase complex 1 MLRQ subunit
EVM0005623	18.46	37.26	-1.01	1.00E-03	5.70E-03	--	Glycosyl hydrolases family 11
EVM0002597	8.82	17.80	-1.01	3.60E-03	1.64E-02	--	Flavin reductase like domain
EVM0012144	38.02	76.72	-1.01	3.00E-04	2.07E-03	Function unknown	Eukaryotic protein of unknown function (DUF829)
EVM0012121	19.26	38.86	-1.01	8.50E-04	4.98E-03	--	Glioma tumor suppressor candidate region
EVM0004149	17.82	35.94	-1.01	6.00E-04	3.72E-03	--	--

EVM0011255	3.28	6.61	-1.01	3.30E-03	1.53E-02	--	--
EVM0006908	35.78	72.14	-1.01	3.50E-04	2.36E-03	--	FAD binding domain
EVM0003316	552.91	1114.40	-1.01	8.00E-04	4.73E-03	--	--
EVM0004270	3.64	7.33	-1.01	3.60E-03	1.64E-02	--	SelR domain
EVM0007073	62.48	125.88	-1.01	3.00E-04	2.07E-03	Nucleotide transport and metabolism	Phosphorylase superfamily
EVM0010957	37.69	75.88	-1.01	5.50E-04	3.46E-03	Transcription	Myb-like DNA-binding domain
EVM0005032	76.66	154.32	-1.01	4.50E-04	2.92E-03	Signal transduction mechanisms	NUDIX domain
EVM0009586	49.10	98.82	-1.01	2.50E-04	1.77E-03	Lipid transport and metabolism	Fatty acid hydroxylase superfamily
EVM0002294	11.65	23.45	-1.01	1.60E-03	8.44E-03	General function prediction only	Alpha/beta hydrolase family
EVM0005707	18.28	36.77	-1.01	2.00E-03	1.01E-02	--	--
EVM0007589	664.77	1336.94	-1.01	4.40E-03	1.94E-02	General function prediction only	Protein of unknown function (DUF1295)
EVM0006910	3.21	6.46	-1.01	3.05E-03	1.44E-02	--	--
EVM0006891	12.59	25.29	-1.01	2.95E-03	1.40E-02	--	--

EVM0007696	8.52	17.12	-1.01	2.05E-03	1.03E-02	--	Domain of unknown function (DUF4139)
EVM0000586	7.63	15.33	-1.01	4.45E-03	1.96E-02	--	Pheromone A receptor
EVM0003220	6.19	12.43	-1.01	7.20E-03	2.88E-02	--	--
EVM0008748	14.50	29.11	-1.01	2.90E-03	1.38E-02	--	--
EVM0004735	8.49	17.04	-1.01	7.50E-04	4.49E-03	--	50S ribosome-binding GTPase
EVM0008438	19.82	39.78	-1.01	4.50E-04	2.92E-03	--	Apoptosis-antagonizing transcription factor, C-terminal
EVM0010937	10.96	22.00	-1.00	1.65E-03	8.65E-03	--	--
EVM0008173	1.71	3.44	-1.00	9.10E-03	3.48E-02	--	--
EVM0003532	39.83	79.87	-1.00	5.50E-04	3.46E-03	General function prediction only	Arrestin (or S-antigen), C-terminal domain
EVM0001759	54.43	109.15	-1.00	2.00E-04	1.46E-03	Energy production and conversion	Mitochondrial carrier protein
EVM0011463	86.93	174.24	-1.00	4.00E-04	2.65E-03	General function prediction only	Ras family
EVM0001105	3.44	6.90	-1.00	5.20E-03	2.22E-02	Carbohydrate transport and metabolism	Glycosyl hydrolase family 47
EVM0012150	60.01	120.20	-1.00	2.50E-04	1.77E-03	--	--

EVM0006647	158.10	316.65	-1.00	5.00E-04	3.19E-03	Amino acid transport and metabolism	Aminotransferase class-V
EVM0011632	64.71	129.55	-1.00	6.00E-04	3.72E-03	--	--
EVM0002524	9.83	19.67	-1.00	1.10E-03	6.17E-03	--	Pectate lyase
EVM0003942	27.11	54.24	-1.00	4.95E-03	2.13E-02	--	Domain of unknown function (DUF202)
EVM0004593	29.98	59.98	-1.00	2.80E-03	1.34E-02	Signal transduction mechanisms	Protein kinase domain
EVM0008908	70.44	140.92	-1.00	5.00E-04	3.19E-03	Translation, ribosomal structure and biogenesis	Brix domain
EVM0000211	31.24	62.49	-1.00	3.50E-04	2.36E-03	RNA processing and modification	WD domain, G-beta repeat
EVM0005980	41.85	20.92	1.00	9.50E-04	5.46E-03	General function prediction only	Animal haem peroxidase
EVM0004069	9.45	4.72	1.00	5.50E-04	3.46E-03	Energy production and conversion	FAD linked oxidases, C-terminal domain
EVM0000703	28.10	14.03	1.00	6.85E-03	2.77E-02	--	--
EVM0008890	17.09	8.53	1.00	2.50E-04	1.77E-03	RNA processing and modification	Sec63 Brl domain
EVM0008138	85.80	42.81	1.00	6.50E-04	3.98E-03	Inorganic ion transport and metabolism	Sodium/hydrogen exchanger family
EVM0008744	51.35	25.58	1.01	4.00E-04	2.65E-03	RNA processing and modification	MIF4G domain

EVM0000862	24.58	12.24	1.01	5.00E-04	3.19E-03	Replication, recombination and repair	MCM2/3/5 family
EVM0005381	62.59	31.16	1.01	4.00E-04	2.65E-03	--	--
EVM0007667	147.00	73.16	1.01	6.00E-04	3.72E-03	--	Ubiquitin-2 like Rad60 SUMO-like
EVM0000205	14.89	7.41	1.01	1.30E-03	7.10E-03	Transcription	SET domain
EVM0008417	8.43	4.19	1.01	2.70E-03	1.30E-02	Secondary metabolites biosynthesis, transport and catabolism	ABC transporter
EVM0001345	24.80	12.32	1.01	5.50E-04	3.46E-03	Cell cycle control, cell division, chromosome partitioning	SIT4 phosphatase-associated protein
EVM0010324	119.57	59.38	1.01	2.50E-04	1.77E-03	--	--
EVM0011018	30.70	15.24	1.01	5.50E-03	2.32E-02	Replication, recombination and repair	Rad51
EVM0002907	59.36	29.46	1.01	1.40E-03	7.55E-03	Defense mechanisms	NAD dependent epimerase/dehydratase family
EVM0012016	234.85	116.45	1.01	2.20E-03	1.10E-02	Amino acid transport and metabolism	Cys/Met metabolism PLP-dependent enzyme
EVM0007248	580.67	287.76	1.01	2.95E-03	1.40E-02	--	RNA recognition motif. (a.k.a. RRM, RBD, or RNP domain)
EVM0001744	12.53	6.20	1.01	1.25E-03	6.87E-03	Signal transduction mechanisms	Protein kinase domain

EVM0002952	29.64	14.68	1.01	6.00E-04	3.72E-03	Extracellular structures	Tropomyosin
EVM0001683	75.96	37.55	1.02	3.00E-04	2.07E-03	Coenzyme transport and metabolism	Probable molybdopterin binding domain
EVM0002941	15.00	7.41	1.02	5.25E-03	2.24E-02	Nucleotide transport and metabolism	Ham1 family
EVM0009790	24.63	12.15	1.02	2.50E-04	1.77E-03	Secondary metabolites biosynthesis, transport and catabolism	ABC transporter
EVM0005867	133.30	65.58	1.02	2.50E-04	1.77E-03	--	--
EVM0000598	66.97	32.93	1.02	4.00E-04	2.65E-03	Cell cycle control, cell division, chromosome partitioning	N terminus of Rad21 / Rec8 like protein
EVM0005161	24.89	12.24	1.02	2.00E-04	1.46E-03	Secondary metabolites biosynthesis, transport and catabolism	ABC transporter
EVM0011116	7.53	3.70	1.03	4.10E-03	1.83E-02	--	Acetamidase/Formamidase family
EVM0006573	13.98	6.86	1.03	1.01E-02	3.76E-02	--	--
EVM0002374	28.64	13.96	1.04	1.00E-04	7.96E-04	--	Alpha/beta hydrolase family
EVM0010842	133.19	64.84	1.04	2.15E-03	1.08E-02	--	Allophanate hydrolase subunit 2

EVM0009401	39.66	19.30	1.04	1.45E-03	7.77E-03	Transcription	--
EVM0011910	57.76	28.09	1.04	1.00E-04	7.96E-04	Intracellular trafficking, secretion, and vesicular transport	VID27 cytoplasmic protein
EVM0000333	24.34	11.84	1.04	2.50E-04	1.77E-03	Posttranslational modification, protein turnover, chaperones	Poly(ADP-ribose) polymerase catalytic domain
EVM0004887	3.64	1.77	1.04	5.35E-03	2.27E-02	--	Protein of unknown function (DUF4246)
EVM0000281	13.40	6.51	1.04	1.20E-03	6.64E-03	--	--
EVM0005732	8.57	4.16	1.04	4.65E-03	2.03E-02	--	NADH:flavin oxidoreductase / NADH oxidase family
EVM0004931	69.14	33.47	1.05	3.00E-04	2.07E-03	--	Munc13 (mammalian uncoordinated) homology domain
EVM0008452	37.88	18.33	1.05	4.80E-03	2.08E-02	--	--
EVM0006262	94.68	45.77	1.05	1.00E-04	7.96E-04	--	Peptidase family M1
EVM0008437	6.13	2.96	1.05	4.65E-03	2.03E-02	Energy production and conversion	Aldehyde dehydrogenase family
EVM0009297	118.76	57.30	1.05	2.50E-04	1.77E-03	Amino acid transport and metabolism	Amino acid permease

EVM0007019	99.16	47.80	1.05	3.00E-04	2.07E-03	--	Dynamin central region
EVM0011884	539.46	259.92	1.05	2.00E-03	1.01E-02	Signal transduction mechanisms	OPT oligopeptide transporter protein
EVM0003920	229.00	110.21	1.06	6.50E-04	3.98E-03	Lipid transport and metabolism	AMP-binding enzyme
EVM0006816	197.10	94.82	1.06	3.00E-04	2.07E-03	--	--
EVM0001031	43.52	20.93	1.06	7.50E-04	4.49E-03	Carbohydrate transport and metabolism	Major Facilitator Superfamily
EVM0003703	40.32	19.37	1.06	1.10E-03	6.17E-03	Function unknown	Ribosome biogenesis protein SLX9
EVM0000369	5.73	2.75	1.06	1.35E-02	4.74E-02	General function prediction only	GMC oxidoreductase
EVM0009536	109.03	52.36	1.06	5.00E-04	3.19E-03	Amino acid transport and metabolism	Cys/Met metabolism PLP-dependent enzyme
EVM0001664	2.17	1.04	1.06	1.15E-02	4.19E-02	Function unknown	Major Facilitator Superfamily
EVM0001497	27.05	12.98	1.06	7.00E-04	4.23E-03	--	NADH(P)-binding
EVM0011905	99.10	47.51	1.06	4.50E-04	2.92E-03	--	Iron permease FTR1 family
EVM0011151	504.48	241.66	1.06	6.50E-04	3.98E-03	Secondary metabolites biosynthesis, transport and catabolism	Cytochrome P450

EVM0002077	23.96	11.47	1.06	3.00E-03	1.42E-02	Posttranslational modification, protein turnover, chaperones	Ankyrin repeat
EVM0000779	143.69	68.76	1.06	8.50E-04	4.98E-03	--	--
EVM0002918	54.49	26.07	1.06	2.50E-04	1.77E-03	--	Cytochrome P450
EVM0000114	9.83	4.70	1.06	1.25E-03	6.87E-03	Posttranslational modification, protein turnover, chaperones	ATPase family associated with various cellular activities (AAA)
EVM0008943	2.36	1.13	1.06	1.30E-02	4.62E-02	Signal transduction mechanisms	Protein kinase domain
EVM0006450	69.31	33.11	1.07	2.00E-04	1.46E-03	General function prediction only	Alpha/beta hydrolase family
EVM0002233	23.83	11.38	1.07	1.03E-02	3.84E-02	--	Dopa 4,5-dioxygenase family
EVM0004972	14.02	6.69	1.07	5.50E-03	2.32E-02	--	--
EVM0000553	117.93	56.19	1.07	2.00E-04	1.46E-03	--	Esterase-like activity of phytase
EVM0002585	30.14	14.35	1.07	2.00E-04	1.46E-03	--	Phosphoinositide 3-kinase family, accessory domain (PIK domain)
EVM0005854	131.76	62.70	1.07	8.00E-04	4.73E-03	Cell wall/membrane/envelope biogenesis	Chitin synthase

EVM0010519	8.28	3.94	1.07	2.80E-03	1.34E-02	--	--
EVM0010882	209.62	99.71	1.07	7.00E-04	4.23E-03	General function prediction only	Histidine phosphatase superfamily (branch 2)
EVM0003014	115.44	54.87	1.07	2.00E-03	1.01E-02	Amino acid transport and metabolism	Methylenetetrahydrofolate reductase
EVM0006386	44.83	21.26	1.08	2.50E-03	1.22E-02	--	--
EVM0000996	109.13	51.58	1.08	8.00E-04	4.73E-03	Lipid transport and metabolism	short chain dehydrogenase
EVM0007160	5.77	2.73	1.08	2.15E-03	1.08E-02	--	--
EVM0009035	45.72	21.58	1.08	1.00E-04	7.96E-04	--	Non-repetitive/WGA-negative nucleoporin C-terminal
EVM0000439	70.92	33.47	1.08	1.50E-04	1.14E-03	--	Kelch motif
EVM0009335	21.15	9.97	1.08	2.70E-03	1.30E-02	--	--
EVM0010160	101.30	47.75	1.09	1.00E-04	7.96E-04	Carbohydrate transport and metabolism	Major Facilitator Superfamily
EVM0007366	59.74	28.14	1.09	1.50E-04	1.14E-03	--	--
EVM0002944	23.88	11.24	1.09	1.40E-03	7.55E-03	--	WD domain, G-beta repeat
EVM0011507	42.35	19.90	1.09	2.00E-04	1.46E-03	--	Nuclear pore component

EVM0004480	16.69	7.83	1.09	6.50E-04	3.98E-03	--	Glycosyl hydrolases family 15
EVM0005478	8.54	3.99	1.10	2.20E-03	1.10E-02	General function prediction only	WD domain, G-beta repeat
EVM0006229	421.19	196.42	1.10	6.00E-04	3.72E-03	Inorganic ion transport and metabolism	Sugar (and other) transporter
EVM0006013	4.64	2.16	1.10	4.25E-03	1.89E-02	--	--
EVM0001008	7.99	3.72	1.10	4.30E-03	1.90E-02	--	--
EVM0012125	20.80	9.65	1.11	2.00E-04	1.46E-03	--	Protein of unknown function (DUF563)
EVM0010840	7.40	3.43	1.11	5.00E-04	3.19E-03	--	--
EVM0005773	100.26	46.45	1.11	5.00E-05	4.28E-04	Secondary metabolites biosynthesis, transport and catabolism	Cytochrome P450
EVM0004652	10.85	5.01	1.11	9.50E-04	5.46E-03	--	Heterokaryon incompatibility protein (HET)
EVM0009121	20.66	9.54	1.11	1.00E-04	7.96E-04	--	C-5 cytosine-specific DNA methylase
EVM0008160	41.36	19.10	1.11	9.50E-04	5.46E-03	General function prediction only	RNA recognition motif. (a.k.a. RRM, RBD, or RNP domain)
EVM0005603	3.50	1.61	1.12	6.30E-03	2.59E-02	--	PhoD-like phosphatase

EVM0006055	11.26	5.17	1.12	7.70E-03	3.04E-02	General function prediction only	short chain dehydrogenase
EVM0002221	24.68	11.31	1.13	7.50E-04	4.49E-03	Amino acid transport and metabolism	Cys/Met metabolism PLP-dependent enzyme
EVM0001336	37.80	17.30	1.13	5.65E-03	2.37E-02	Signal transduction mechanisms	HIT domain
EVM0004123	3.63	1.66	1.13	1.95E-03	9.93E-03	--	Telomerase ribonucleoprotein complex - RNA binding domain
EVM0004768	45.99	21.01	1.13	1.00E-04	7.96E-04	--	Major Facilitator Superfamily
EVM0001841	43.56	19.89	1.13	2.50E-04	1.77E-03	--	--
EVM0001119	43.58	19.90	1.13	5.00E-05	4.28E-04	Signal transduction mechanisms	Response regulator receiver domain
EVM0000108	60.50	27.61	1.13	5.50E-04	3.46E-03	--	Amidohydrolase
EVM0000347	249.76	113.76	1.13	5.00E-05	4.28E-04	--	--
EVM0004356	6.16	2.80	1.14	1.37E-02	4.81E-02	General function prediction only	Dienelactone hydrolase family
EVM0003763	5.03	2.29	1.14	5.00E-04	3.19E-03	Signal transduction mechanisms	Protein kinase domain
EVM0011389	125.73	57.10	1.14	2.00E-04	1.46E-03	--	FAD binding domain
EVM0004067	50.64	22.99	1.14	1.00E-04	7.96E-04	General function prediction only	HMG (high mobility group) box

EVM0000119	26.76	12.14	1.14	2.50E-04	1.77E-03	--	3-beta hydroxysteroid dehydrogenase/isomerase family
EVM0003946	472.85	214.26	1.14	1.25E-03	6.87E-03	--	--
EVM0006381	22.21	10.04	1.15	1.50E-04	1.14E-03	Cell cycle control, cell division, chromosome partitioning	PIF1-like helicase
EVM0002487	14.15	6.39	1.15	6.00E-04	3.72E-03	Signal transduction mechanisms	OPT oligopeptide transporter protein
EVM0005868	131.16	59.21	1.15	8.50E-04	4.98E-03	--	--
EVM0006071	52.11	23.52	1.15	1.00E-04	7.96E-04	Translation, ribosomal structure and biogenesis	tRNA synthetases class II (D, K and N)
EVM0008628	20.34	9.18	1.15	2.00E-04	1.46E-03	--	--
EVM0010014	183.69	82.89	1.15	2.50E-04	1.77E-03	General function prediction only	Calcineurin-like phosphoesterase
EVM0002909	20.45	9.21	1.15	2.50E-04	1.77E-03	--	NAD(P)-binding Rossmann-like domain
EVM0001213	71.20	32.06	1.15	3.85E-03	1.74E-02	General function prediction only	Thioesterase superfamily
EVM0001011	77.08	34.69	1.15	1.50E-04	1.14E-03	--	Cytochrome P450
EVM0011663	117.26	52.62	1.16	2.50E-04	1.77E-03	Posttranslational modification, protein turnover, chaperones	Prefoldin subunit

EVM0000530	32.20	14.44	1.16	5.00E-05	4.28E-04	Function unknown	Ribosome 60S biogenesis N-terminal
EVM0010598	4.70	2.11	1.16	7.75E-03	3.06E-02	--	F-box-like
EVM0004840	299.94	134.47	1.16	1.50E-04	1.14E-03	--	--
EVM0007450	55.67	24.92	1.16	2.50E-04	1.77E-03	Amino acid transport and metabolism	Beta-eliminating lyase
EVM0007477	41.72	18.66	1.16	2.00E-04	1.46E-03	General function prediction only	Metallo-beta-lactamase superfamily
EVM0004619	45.90	20.52	1.16	1.00E-04	7.96E-04	Nucleotide transport and metabolism	Amidohydrolase
EVM0004153	229.27	102.30	1.16	3.00E-04	2.07E-03	--	--
EVM0008035	24.86	11.08	1.17	5.00E-05	4.28E-04	General function prediction only	SNF2 family N-terminal domain
EVM0008257	56.78	25.30	1.17	1.30E-03	7.10E-03	--	--
EVM0002937	118.24	52.67	1.17	5.00E-05	4.28E-04	--	--
EVM0001562	135.12	60.05	1.17	4.50E-04	2.92E-03	--	Alpha amylase, catalytic domain
EVM0003369	1.82	0.81	1.17	1.34E-02	4.73E-02	General function prediction only	GMC oxidoreductase

EVM0002999	53.51	23.77	1.17	1.00E-04	7.96E-04	Intracellular trafficking, secretion, and vesicular transport	ATG C terminal domain
EVM0005203	38.75	17.21	1.17	1.50E-04	1.14E-03	Intracellular trafficking, secretion, and vesicular transport	Pep3/Vps18/deep orange family
EVM0010728	53.05	23.55	1.17	5.00E-05	4.28E-04	--	--
EVM0001015	6.89	3.06	1.17	3.90E-03	1.76E-02	--	--
EVM0007942	113.13	50.15	1.17	1.50E-04	1.14E-03	Function unknown	Protein of unknown function DUF89
EVM0007734	104.81	46.42	1.18	5.00E-05	4.28E-04	Secondary metabolites biosynthesis, transport and catabolism	Pyridine nucleotide-disulphide oxidoreductase
EVM0007527	227.68	100.76	1.18	9.00E-04	5.22E-03	Intracellular trafficking, secretion, and vesicular transport	Region in Clathrin and VPS
EVM0008911	8.40	3.71	1.18	3.00E-04	2.07E-03	--	--
EVM0003020	49.81	21.99	1.18	1.15E-03	6.41E-03	--	--
EVM0011948	47.96	21.16	1.18	5.50E-04	3.46E-03	Posttranslational modification, protein turnover, chaperones	Zinc knuckle

EVM0000617	24.09	10.61	1.18	1.00E-04	7.96E-04	Cytoskeleton	Kinesin motor domain
EVM0000645	775.42	340.85	1.19	1.35E-02	4.74E-02	--	Hemopexin
EVM0003003	89.85	39.47	1.19	5.00E-05	4.28E-04	Coenzyme transport and metabolism	Glutamate-cysteine ligase
EVM0005845	32.84	14.43	1.19	5.00E-05	4.28E-04	--	Domain of unknown function (DUF4470)
EVM0000265	181.11	79.43	1.19	9.50E-04	5.46E-03	Cell wall/membrane/envelope biogenesis	Chitin synthase
EVM0004112	187.54	82.22	1.19	5.00E-05	4.28E-04	--	--
EVM0010927	52.09	22.84	1.19	3.50E-04	2.36E-03	--	BTB/POZ domain
EVM0009544	58.48	25.57	1.19	1.00E-04	7.96E-04	Posttranslational modification, protein turnover, chaperones	IBR domain
EVM0001822	4.39	1.92	1.19	5.10E-03	2.19E-02	--	--
EVM0010994	82.75	36.16	1.19	5.00E-05	4.28E-04	--	Pro-kumamolisin, activation domain
EVM0008720	239.89	104.78	1.19	1.50E-04	1.14E-03	Nucleotide transport and metabolism	Ribonucleotide reductase, barrel domain

EVM0002114	154.53	67.49	1.20	1.50E-04	1.14E-03	Posttranslational modification, protein turnover, chaperones	Proteasome/cyclosome repeat
EVM0004023	91.45	39.91	1.20	5.00E-05	4.28E-04	Secondary metabolites biosynthesis, transport and catabolism	Pyridine nucleotide-disulphide oxidoreductase
EVM0011704	26.06	11.37	1.20	8.50E-04	4.98E-03	Inorganic ion transport and metabolism	ZIP Zinc transporter
EVM0008192	232.76	101.56	1.20	3.50E-04	2.36E-03	Posttranslational modification, protein turnover, chaperones	ThiF family
EVM0011483	145.00	63.26	1.20	3.00E-04	2.07E-03	--	--
EVM0005159	86.07	37.55	1.20	1.00E-04	7.96E-04	--	NADH:flavin oxidoreductase / NADH oxidase family
EVM0010477	7.66	3.34	1.20	6.00E-04	3.72E-03	Posttranslational modification, protein turnover, chaperones	Prolyl oligopeptidase family
EVM0000473	57.49	25.04	1.20	6.00E-04	3.72E-03	--	--
EVM0005286	425.68	184.96	1.20	5.00E-05	4.28E-04	--	--
EVM0009688	13.16	5.71	1.20	1.30E-03	7.10E-03	--	Zinc-binding dehydrogenase
EVM0003473	161.61	70.13	1.20	5.00E-05	4.28E-04	Amino acid transport and metabolism	POT family

EVM0005738	710.86	307.03	1.21	2.00E-04	1.46E-03	RNA processing and modification	LSM domain
EVM0012111	24.57	10.60	1.21	1.26E-02	4.49E-02	--	--
EVM0011560	18.36	7.92	1.21	6.50E-04	3.98E-03	--	Membrane bound O-acyl transferase family
EVM0007397	15.13	6.52	1.21	2.50E-04	1.77E-03	Function unknown	WD domain, G-beta repeat
EVM0008335	49.06	21.15	1.21	5.00E-05	4.28E-04	Function unknown	Protein of unknown function (DUF3414)
EVM0005679	18.98	8.17	1.21	5.00E-04	3.19E-03	--	--
EVM0002528	69.71	29.97	1.22	5.00E-05	4.28E-04	Cytoskeleton	WD domain, G-beta repeat
EVM0003132	166.24	71.42	1.22	5.00E-05	4.28E-04	--	Histidine-specific methyltransferase, SAM-dependent
EVM0004177	93.81	40.26	1.22	6.50E-04	3.98E-03	Inorganic ion transport and metabolism	Nitrite and sulphite reductase 4Fe-4S domain
EVM0011713	9.22	3.95	1.22	7.50E-04	4.49E-03	Secondary metabolites biosynthesis, transport and catabolism	Cytochrome P450
EVM0006793	325.86	139.46	1.22	7.85E-03	3.09E-02	--	--
EVM0005557	49.62	21.20	1.23	5.00E-05	4.28E-04	--	Nucleoporin subcomplex protein binding to Pom34

EVM0000098	11.76	5.02	1.23	3.50E-04	2.36E-03	--	Glycosyl hydrolase family 3 N terminal domain
EVM0006748	96.81	41.16	1.23	1.50E-04	1.14E-03	Function unknown	TPR/MLP1/MLP2-like protein
EVM0005178	7.52	3.19	1.23	4.95E-03	2.13E-02	--	--
EVM0005776	238.64	101.39	1.23	5.00E-05	4.28E-04	Posttranslational modification, protein turnover, chaperones	Glutathione S-transferase, N-terminal domain
EVM0007924	261.54	110.85	1.24	5.00E-05	4.28E-04	Carbohydrate transport and metabolism	Protein of unknown function (DUF791)
EVM0001663	2.80	1.18	1.24	9.05E-03	3.46E-02	Energy production and conversion	Pyridine nucleotide-disulphide oxidoreductase
EVM0008259	1280.14	541.81	1.24	7.00E-04	4.23E-03	--	Putative oxidoreductase C terminal
EVM0006449	152.41	64.48	1.24	5.00E-05	4.28E-04	--	Ricin-type beta-trefoil lectin domain
EVM0009922	116.75	49.39	1.24	5.00E-05	4.28E-04	--	Protein kinase domain
EVM0006696	36.96	15.63	1.24	6.25E-03	2.57E-02	--	Domain of unknown function DUF77
EVM0010847	296.83	125.53	1.24	5.00E-05	4.28E-04	--	Leucine Rich repeats (2 copies)
EVM0007960	3.19	1.35	1.25	1.90E-03	9.73E-03	--	Heterokaryon incompatibility protein (HET)

EVM0003998	28.42	11.97	1.25	7.00E-04	4.23E-03	Posttranslational modification, protein turnover, chaperones	Proteasome activator pa28 beta subunit
EVM0009500	121.52	51.03	1.25	5.00E-05	4.28E-04	--	Domain of unknown function (DUF1708)
EVM0007131	10.37	4.35	1.25	4.15E-03	1.85E-02	--	--
EVM0006718	47.56	19.94	1.25	7.00E-04	4.23E-03	Amino acid transport and metabolism	FAD dependent oxidoreductase
EVM0005326	5.03	2.11	1.25	8.00E-04	4.73E-03	General function prediction only	GMC oxidoreductase
EVM0002000	42.12	17.63	1.26	5.00E-05	4.28E-04	Posttranslational modification, protein turnover, chaperones	Prolyl oligopeptidase, N-terminal beta-propeller domain
EVM0007222	4.11	1.72	1.26	1.90E-03	9.73E-03	--	Protein of unknown function (DUF4246)
EVM0006789	83.87	35.06	1.26	5.00E-05	4.28E-04	Posttranslational modification, protein turnover, chaperones	Eukaryotic aspartyl protease
EVM0004481	7.50	3.13	1.26	3.90E-03	1.76E-02	--	--
EVM0004594	108.53	45.20	1.26	5.00E-05	4.28E-04	Amino acid transport and metabolism	Amino acid permease
EVM0000790	13.69	5.70	1.27	1.00E-04	7.96E-04	Translation, ribosomal structure and biogenesis	Piwi domain

EVM0003945	205.26	85.30	1.27	4.50E-04	2.92E-03	Cell wall/membrane/envelope biogenesis	Chitin synthase
EVM0000466	51.44	21.36	1.27	5.00E-05	4.28E-04	General function prediction only	Peptidase family M49
EVM0008931	52.62	21.83	1.27	5.00E-05	4.28E-04	--	Growth-Arrest-Specific Protein 2 Domain
EVM0012149	61.11	25.29	1.27	5.00E-05	4.28E-04	--	Beta-lactamase superfamily domain
EVM0010488	29.72	12.30	1.27	1.20E-03	6.64E-03	--	--
EVM0008962	28.57	11.78	1.28	3.50E-04	2.36E-03	General function prediction only	MOSC domain
EVM0008061	87.73	36.14	1.28	5.00E-05	4.28E-04	General function prediction only	GMC oxidoreductase
EVM0009152	5.95	2.45	1.28	2.50E-04	1.77E-03	--	Bacterial alpha-L-rhamnosidase
EVM0011225	36.71	15.08	1.28	1.00E-04	7.96E-04	RNA processing and modification	Ribonuclease III domain
EVM0004082	2.45	1.01	1.28	1.43E-02	4.97E-02	--	NADH:flavin oxidoreductase / NADH oxidase family
EVM0000724	49.48	20.27	1.29	5.00E-05	4.28E-04	Translation, ribosomal structure and biogenesis	Piwi domain
EVM0000838	216.34	88.48	1.29	5.00E-05	4.28E-04	--	Glycosyl Hydrolase Family 88

EVM0001687	16.11	6.58	1.29	5.00E-05	4.28E-04	--	RecF/RecN/SMC N terminal domain
EVM0001466	14.05	5.73	1.29	1.50E-04	1.14E-03	Posttranslational modification, protein turnover, chaperones	Peptidase family M48
EVM0003437	22.07	8.98	1.30	5.00E-05	4.28E-04	Posttranslational modification, protein turnover, chaperones	Tubulin folding cofactor D C terminal
EVM0003467	483.21	196.61	1.30	5.00E-05	4.28E-04	--	--
EVM0006877	21.93	8.90	1.30	4.50E-04	2.92E-03	--	Membrane bound O-acyl transferase family
EVM0004984	129.22	52.46	1.30	5.00E-05	4.28E-04	--	--
EVM0006959	20.84	8.45	1.30	3.00E-03	1.42E-02	--	Cytochrome oxidase complex assembly protein 1
EVM0008057	3.42	1.38	1.30	4.25E-03	1.89E-02	Energy production and conversion	Aldehyde dehydrogenase family
EVM0002760	39.53	15.99	1.31	7.00E-04	4.23E-03	--	--
EVM0007194	50.62	20.46	1.31	5.00E-05	4.28E-04	--	Major Facilitator Superfamily
EVM0007451	84.78	34.11	1.31	5.00E-05	4.28E-04	--	Beta-glucan synthesis-associated protein (SKN1)
EVM0010231	76.04	30.59	1.31	5.00E-05	4.28E-04	--	NPL4 family

EVM0007272	43.04	17.31	1.31	5.00E-05	4.28E-04	--	Pro-kumamolisin, activation domain
EVM0012119	6.10	2.45	1.32	4.00E-04	2.65E-03	--	Ankyrin repeats (3 copies)
EVM0002146	6.66	2.67	1.32	9.00E-04	5.22E-03	--	Pectinesterase
EVM0005137	6.31	2.53	1.32	1.03E-02	3.84E-02	#N/A	#N/A
EVM0010942	29.63	11.87	1.32	5.00E-05	4.28E-04	--	--
EVM0007516	88.32	35.37	1.32	5.00E-05	4.28E-04	Carbohydrate transport and metabolism	Glycosyl hydrolases family 38 N-terminal domain
EVM0009062	3.11	1.25	1.32	2.70E-03	1.30E-02	--	Domain of unknown function (DUF4139)
EVM0008666	33.70	13.49	1.32	5.00E-05	4.28E-04	--	Dioxygenase
EVM0000179	3.22	1.29	1.32	3.45E-03	1.59E-02	Secondary metabolites biosynthesis, transport and catabolism	Cytochrome P450
EVM0004320	149.65	59.82	1.32	5.00E-05	4.28E-04	General function prediction only	Aldo/keto reductase family
EVM0006268	77.30	30.87	1.32	5.00E-05	4.28E-04	--	--
EVM0007570	19.30	7.70	1.33	5.00E-05	4.28E-04	--	Poly(ADP-ribose) polymerase catalytic domain

EVM0009974	82.68	32.89	1.33	5.00E-05	4.28E-04	Signal transduction mechanisms	GTPase-activator protein for Ras-like GTPase
EVM0005979	5.28	2.10	1.33	3.50E-03	1.61E-02	--	--
EVM0008616	72.25	28.70	1.33	5.00E-05	4.28E-04	Carbohydrate transport and metabolism	Major Facilitator Superfamily
EVM0005231	18.27	7.24	1.33	1.00E-04	7.96E-04	Amino acid transport and metabolism	FAD dependent oxidoreductase
EVM0006160	23.11	9.12	1.34	5.00E-05	4.28E-04	Function unknown	Major Facilitator Superfamily
EVM0007751	78.58	30.98	1.34	5.00E-05	4.28E-04	--	--
EVM0009444	9517.76	3750.32	1.34	9.00E-04	5.22E-03	--	Fungal hydrophobin
EVM0001877	4.63	1.82	1.35	1.95E-03	9.93E-03	--	Voltage-dependent anion channel
EVM0011240	225.96	88.62	1.35	5.00E-05	4.28E-04	--	--
EVM0010629	69.29	27.10	1.35	2.45E-03	1.20E-02	--	Glycosyl hydrolase family 92
EVM0001405	143.94	56.30	1.35	5.00E-05	4.28E-04	Intracellular trafficking, secretion, and vesicular transport	Sybindin-like family
EVM0003929	17.66	6.90	1.36	1.50E-04	1.14E-03	--	Taurine catabolism dioxygenase TauD, TfdA family

EVM0006295	48.19	18.80	1.36	5.00E-05	4.28E-04	General function prediction only	Dienelactone hydrolase family
EVM0003122	43.47	16.92	1.36	5.00E-05	4.28E-04	Translation, ribosomal structure and biogenesis	Domain of unknown function (DUF3554)
EVM0008150	41.66	16.20	1.36	7.00E-04	4.23E-03	Energy production and conversion	Aldo/keto reductase family
EVM0011231	3.25	1.26	1.36	2.65E-03	1.28E-02	--	Serine carboxypeptidase
EVM0009509	35.80	13.88	1.37	2.00E-04	1.46E-03	General function prediction only	GMC oxidoreductase
EVM0011805	2.41	0.93	1.37	2.75E-03	1.32E-02	--	Tht1-like nuclear fusion protein
EVM0002563	9.23	3.57	1.37	3.50E-04	2.36E-03	--	Protein of unknown function (DUF1399)
EVM0002782	25.36	9.81	1.37	1.50E-04	1.14E-03	--	--
EVM0011882	43.81	16.90	1.37	1.50E-04	1.14E-03	Cytoskeleton	Variant SH3 domain
EVM0009019	3.42	1.32	1.37	1.34E-02	4.73E-02	--	Survival protein SurE
EVM0006352	148.47	57.25	1.37	5.00E-05	4.28E-04	General function prediction only	short chain dehydrogenase
EVM0010470	134.22	51.70	1.38	5.00E-05	4.28E-04	Posttranslational modification, protein turnover, chaperones	Proteasome/cyclosome repeat

EVM0007276	4.44	1.71	1.38	2.05E-03	1.03E-02	--	Acetyltransferase (GNAT) domain
EVM0009736	31.16	11.99	1.38	5.00E-05	4.28E-04	--	PhoD-like phosphatase
EVM0008271	13.32	5.12	1.38	1.65E-03	8.65E-03	--	--
EVM0000536	8.79	3.38	1.38	4.35E-03	1.92E-02	--	--
EVM0003743	6.24	2.40	1.38	1.00E-03	5.70E-03	Carbohydrate transport and metabolism	Major Facilitator Superfamily
EVM0005149	20.09	7.70	1.38	5.00E-05	4.28E-04	--	--
EVM0005153	17.94	6.87	1.38	1.00E-04	7.96E-04	General function prediction only	Major Facilitator Superfamily
EVM0001489	73.96	28.26	1.39	5.00E-05	4.28E-04	General function prediction only	Enoyl-(Acyl carrier protein) reductase
EVM0001362	11.71	4.47	1.39	1.00E-04	7.96E-04	--	Protein of unknown function (DUF1212)
EVM0002085	260.21	99.33	1.39	1.75E-03	9.08E-03	--	Major Facilitator Superfamily
EVM0011278	744.06	283.63	1.39	5.00E-05	4.28E-04	--	--
EVM0005755	56.14	21.40	1.39	5.00E-05	4.28E-04	--	Glycosyl hydrolases family 16
EVM0005145	31.58	12.04	1.39	6.65E-03	2.70E-02	--	--

EVM0008313	24.55	9.35	1.39	5.00E-05	4.28E-04	Secondary metabolites biosynthesis, transport and catabolism	ABC transporter transmembrane region
EVM0007446	1034.64	393.16	1.40	6.50E-04	3.98E-03	--	Fungalysin metallopeptidase (M36)
EVM0009632	159.39	60.52	1.40	1.00E-04	7.96E-04	Secondary metabolites biosynthesis, transport and catabolism	Copper amine oxidase, enzyme domain
EVM0008081	409.36	155.28	1.40	5.00E-05	4.28E-04	Translation, ribosomal structure and biogenesis	Ribosomal protein S19
EVM0000747	20.03	7.58	1.40	5.00E-04	3.19E-03	General function prediction only	Scavenger mRNA decapping enzyme C-term binding
EVM0006635	12.66	4.79	1.40	5.00E-05	4.28E-04	--	Glutaredoxin-like domain (DUF836)
EVM0006660	559.24	211.01	1.41	5.00E-05	4.28E-04	--	--
EVM0009298	38.01	14.33	1.41	5.00E-05	4.28E-04	--	--
EVM0002859	49.39	18.57	1.41	5.00E-05	4.28E-04	--	non-SMC mitotic condensation complex subunit 1
EVM0002582	320.26	120.20	1.41	5.00E-05	4.28E-04	Translation, ribosomal structure and biogenesis	RNB domain
EVM0002222	8.41	3.16	1.41	3.50E-04	2.36E-03	--	Cytochrome P450

EVM0000680	14.42	5.40	1.42	2.00E-04	1.46E-03	Secondary metabolites biosynthesis, transport and catabolism	Cytochrome P450
EVM0011859	8.71	3.26	1.42	4.00E-04	2.65E-03	Energy production and conversion	Phosphoenolpyruvate phosphomutase
EVM0000187	7.49	2.80	1.42	2.75E-03	1.32E-02	--	--
EVM0005170	9.53	3.55	1.42	7.50E-04	4.49E-03	Secondary metabolites biosynthesis, transport and catabolism	Cytochrome P450
EVM0008923	8.05	3.00	1.42	6.00E-04	3.72E-03	--	--
EVM0007579	204.50	76.15	1.43	5.00E-05	4.28E-04	--	HEAT repeat
EVM0009131	99.84	37.13	1.43	5.00E-05	4.28E-04	Lipid transport and metabolism	AMP-binding enzyme
EVM0005521	98.68	36.68	1.43	5.00E-05	4.28E-04	--	GPR1/FUN34/yaaH family
EVM0009273	5.93	2.20	1.43	1.50E-04	1.14E-03	--	--
EVM0011375	3.54	1.31	1.43	4.20E-03	1.87E-02	--	--
EVM0003471	5.07	1.88	1.43	4.50E-04	2.92E-03	General function prediction only	GMC oxidoreductase

EVM0005515	9.55	3.54	1.43	4.00E-04	2.65E-03	Secondary metabolites biosynthesis, transport and catabolism	Cytochrome P450
EVM0000884	30.51	11.28	1.44	5.00E-05	4.28E-04	Lipid transport and metabolism	AMP-binding enzyme
EVM0008039	641.59	237.16	1.44	1.50E-04	1.14E-03	--	--
EVM0001300	4.44	1.64	1.44	4.30E-03	1.90E-02	--	--
EVM0010856	41.65	15.35	1.44	5.00E-05	4.28E-04	--	--
EVM0004107	5.43	2.00	1.44	3.00E-03	1.42E-02	--	--
EVM0012135	16.50	6.05	1.45	2.00E-04	1.46E-03	--	--
EVM0002960	3.64	1.33	1.45	6.80E-03	2.75E-02	--	--
EVM0004000	53.62	19.64	1.45	5.00E-05	4.28E-04	Carbohydrate transport and metabolism	Glucose-6-phosphate dehydrogenase, C-terminal domain
EVM0006771	207.54	75.95	1.45	5.00E-05	4.28E-04	Carbohydrate transport and metabolism	Alpha amylase, catalytic domain
EVM0006519	51.21	18.71	1.45	5.00E-05	4.28E-04	--	Putative amidotransferase
EVM0008140	28.50	10.38	1.46	5.00E-05	4.28E-04	--	--

EVM0010535	14.15	5.15	1.46	7.55E-03	2.99E-02	--	NmrA-like family
EVM0011462	33.58	12.20	1.46	5.00E-05	4.28E-04	Intracellular trafficking, secretion, and vesicular transport	Exocyst complex subunit Sec15-like
EVM0008739	362.39	131.42	1.46	5.00E-05	4.28E-04	Energy production and conversion	Carbamoyl-phosphate synthase L chain, ATP binding domain
EVM0000512	9.72	3.52	1.46	5.50E-04	3.46E-03	--	--
EVM0011123	59.11	21.40	1.47	5.00E-05	4.28E-04	Posttranslational modification, protein turnover, chaperones	Insulinase (Peptidase family M16)
EVM0004211	193.79	70.03	1.47	5.00E-05	4.28E-04	Replication, recombination and repair	ATPase family associated with various cellular activities (AAA)
EVM0011159	4.15	1.50	1.47	6.50E-04	3.98E-03	Secondary metabolites biosynthesis, transport and catabolism	L-lysine 6-monooxygenase (NADPH-requiring)
EVM0006087	217.43	78.48	1.47	5.00E-05	4.28E-04	Inorganic ion transport and metabolism	Ammonium Transporter Family
EVM0006340	52.61	18.99	1.47	5.00E-05	4.28E-04	--	Nup53/35/40-type RNA recognition motif
EVM0000810	40.81	14.72	1.47	1.44E-02	4.99E-02	General function prediction only	WD domain, G-beta repeat
EVM0008782	19.36	6.98	1.47	5.00E-05	4.28E-04	Replication, recombination and repair	MCM2/3/5 family

EVM0003237	125.74	45.31	1.47	5.00E-05	4.28E-04	Amino acid transport and metabolism	Glutamate/Leucine/Phenylalanine/Valine dehydrogenase
EVM0006267	327.34	117.24	1.48	5.00E-05	4.28E-04	Energy production and conversion	Aldo/keto reductase family
EVM0006868	824.81	295.30	1.48	5.00E-05	4.28E-04	General function prediction only	RNA recognition motif. (a.k.a. RRM, RBD, or RNP domain)
EVM0004436	3.55	1.27	1.48	1.03E-02	3.82E-02	--	Acetamidase/Formamidase family
EVM0011188	13.95	4.98	1.49	5.00E-05	4.28E-04	Carbohydrate transport and metabolism	Major Facilitator Superfamily
EVM0003971	22.83	8.15	1.49	1.00E-04	7.96E-04	Coenzyme transport and metabolism	Dihydroneopterin aldolase
EVM0006629	44.42	15.84	1.49	5.00E-05	4.28E-04	Secondary metabolites biosynthesis, transport and catabolism	ABC transporter
EVM0002696	5.68	2.02	1.49	2.50E-04	1.77E-03	Signal transduction mechanisms	OPT oligopeptide transporter protein
EVM0008679	8.03	2.85	1.50	5.00E-05	4.28E-04	Signal transduction mechanisms	RhoGEF domain
EVM0004575	139.05	49.21	1.50	1.00E-04	7.96E-04	General function prediction only	GMC oxidoreductase
EVM0009784	7.99	2.83	1.50	5.50E-04	3.46E-03	Energy production and conversion	D-isomer specific 2-hydroxyacid dehydrogenase, NAD binding domain

EVM0010467	1.22	0.43	1.50	1.20E-02	4.33E-02	Secondary metabolites biosynthesis, transport and catabolism	Cytochrome P450
EVM0009044	79.78	28.18	1.50	5.00E-05	4.28E-04	--	--
EVM0008864	173.27	61.12	1.50	5.00E-05	4.28E-04	Energy production and conversion	Acetyl-CoA hydrolase/transferase N-terminal domain
EVM0003583	822.18	289.96	1.50	3.50E-04	2.36E-03	--	--
EVM0009751	2.30	0.81	1.50	1.00E-04	7.96E-04	--	--
EVM0005610	92.60	32.60	1.51	5.00E-05	4.28E-04	--	Acetamidase/Formamidase family
EVM0006727	43.81	15.40	1.51	5.00E-05	4.28E-04	--	--
EVM0011299	171.44	60.19	1.51	5.00E-05	4.28E-04	General function prediction only	Sugar (and other) transporter
EVM0004388	2.41	0.84	1.51	5.00E-05	4.28E-04	--	--
EVM0010473	46.57	16.33	1.51	5.00E-05	4.28E-04	Secondary metabolites biosynthesis, transport and catabolism	Multicopper oxidase
EVM0011675	31.01	10.86	1.51	5.00E-05	4.28E-04	Cytoskeleton	Dynein heavy chain and region D6 of dynein motor

EVM0002736	88.78	31.00	1.52	5.00E-05	4.28E-04	Cytoskeleton	Myosin head (motor domain)
EVM0001358	6.31	2.20	1.52	1.00E-04	7.96E-04	General function prediction only	GMC oxidoreductase
EVM0007746	28.10	9.75	1.53	5.00E-05	4.28E-04	--	JmjC domain, hydroxylase
EVM0008413	43.83	15.19	1.53	5.00E-05	4.28E-04	General function prediction only	ABC1 family
EVM0001128	10.95	3.79	1.53	5.00E-05	4.28E-04	--	Cytochrome P450
EVM0011387	77.10	26.65	1.53	5.00E-05	4.28E-04	Translation, ribosomal structure and biogenesis	tRNA synthetases class I (E and Q), catalytic domain
EVM0003698	127.33	44.00	1.53	5.00E-05	4.28E-04	--	Alginate lyase
EVM0010784	22.55	7.79	1.53	5.00E-05	4.28E-04	--	--
EVM0012202	1409.84	485.96	1.54	2.00E-04	1.46E-03	--	--
EVM0008462	5.65	1.94	1.54	4.50E-04	2.92E-03	--	NADH:flavin oxidoreductase / NADH oxidase family
EVM0007192	40.32	13.86	1.54	5.00E-05	4.28E-04	Cell cycle control, cell division, chromosome partitioning	Protein kinase domain
EVM0004677	170.33	58.57	1.54	5.00E-05	4.28E-04	--	Serine carboxypeptidase S28

EVM0006788	204.59	70.28	1.54	5.00E-05	4.28E-04	--	--
EVM0011825	5.86	2.01	1.54	5.00E-05	4.28E-04	Carbohydrate transport and metabolism	Glycosyl hydrolases family 35
EVM0009138	8.14	2.78	1.55	1.00E-04	7.96E-04	--	--
EVM0001810	190.23	64.65	1.56	5.00E-05	4.28E-04	Carbohydrate transport and metabolism	Major intrinsic protein
EVM0004285	33.40	11.28	1.57	5.00E-05	4.28E-04	Secondary metabolites biosynthesis, transport and catabolism	Multicopper oxidase
EVM0011488	12.84	4.33	1.57	5.00E-05	4.28E-04	--	--
EVM0007035	68.03	22.81	1.58	5.00E-05	4.28E-04	--	Transferrin receptor-like dimerisation domain
EVM0010980	1.75	0.58	1.58	2.50E-04	1.77E-03	--	--
EVM0007317	3.26	1.08	1.59	8.00E-04	4.73E-03	--	Protein of unknown function (DUF1399)
EVM0003788	1.97	0.65	1.59	4.65E-03	2.03E-02	--	--
EVM0006864	90.08	29.83	1.59	4.50E-04	2.92E-03	--	Glyoxal oxidase N-terminus
EVM0002121	22.65	7.49	1.60	5.00E-05	4.28E-04	Replication, recombination and repair	AAA domain

EVM0003469	2.31	0.76	1.60	3.25E-03	1.51E-02	Carbohydrate transport and metabolism	Glycosyl-transferase for dystroglycan
EVM0010991	17.57	5.78	1.60	1.00E-04	7.96E-04	Carbohydrate transport and metabolism	Histidine phosphatase superfamily (branch 1)
EVM0010340	10.47	3.44	1.61	5.00E-05	4.28E-04	Energy production and conversion	Pyridine nucleotide-disulphide oxidoreductase
EVM0008227	34.98	11.47	1.61	5.00E-05	4.28E-04	Replication, recombination and repair	MCM2/3/5 family
EVM0006552	11.35	3.72	1.61	5.00E-05	4.28E-04	Secondary metabolites biosynthesis, transport and catabolism	Male sterility protein
EVM0003051	150.00	49.02	1.61	5.00E-05	4.28E-04	--	--
EVM0000912	6.36	2.07	1.62	1.23E-02	4.40E-02	#N/A	#N/A
EVM0006965	38.85	12.64	1.62	5.00E-05	4.28E-04	Inorganic ion transport and metabolism	Phosphate transporter family
EVM0006675	19.93	6.48	1.62	7.65E-03	3.02E-02	--	--
EVM0002787	1001.29	323.55	1.63	5.00E-05	4.28E-04	Posttranslational modification, protein turnover, chaperones	Thioredoxin
EVM0004830	30.62	9.88	1.63	1.50E-04	1.14E-03	--	YqcI/YcgG family
EVM0006225	85.30	27.53	1.63	5.00E-05	4.28E-04	--	--

EVM0011867	24.90	8.04	1.63	5.00E-05	4.28E-04	General function prediction only	Haloacid dehalogenase-like hydrolase
EVM0002414	33.58	10.83	1.63	5.00E-05	4.28E-04	--	--
EVM0012100	495.34	159.46	1.64	5.00E-05	4.28E-04	General function prediction only	Carboxylesterase family
EVM0005595	3.16	1.02	1.64	5.50E-04	3.46E-03	--	Serine carboxypeptidase
EVM0009488	6.57	2.11	1.64	2.20E-03	1.10E-02	--	--
EVM0000706	84.78	27.16	1.64	5.00E-05	4.28E-04	--	Alginate lyase
EVM0011907	51.08	16.32	1.65	5.00E-05	4.28E-04	Coenzyme transport and metabolism	Polyprenyl synthetase
EVM0010778	8.19	2.62	1.65	5.00E-05	4.28E-04	--	SNF2 family N-terminal domain
EVM0011846	177.26	56.11	1.66	5.00E-05	4.28E-04	Posttranslational modification, protein turnover, chaperones	Glutathione S-transferase, N-terminal domain
EVM0001971	15.63	4.94	1.66	5.00E-04	3.19E-03	--	--
EVM0002115	16.32	5.15	1.66	5.00E-05	4.28E-04	--	Pregnancy-associated plasma protein-A
EVM0000101	23.98	7.56	1.67	2.00E-04	1.46E-03	#N/A	#N/A

EVM0011327	18.95	5.97	1.67	1.00E-04	7.96E-04	Replication, recombination and repair	AAA domain
EVM0001061	1.30	0.41	1.67	1.75E-03	9.08E-03	Posttranslational modification, protein turnover, chaperones	BCS1 N terminal
EVM0002963	100.39	31.23	1.68	5.00E-05	4.28E-04	--	--
EVM0001672	17.06	5.30	1.69	5.00E-05	4.28E-04	Secondary metabolites biosynthesis, transport and catabolism	Alcohol dehydrogenase GroES-like domain
EVM0000594	15.52	4.82	1.69	5.00E-05	4.28E-04	--	NmrA-like family
EVM0011428	24.49	7.60	1.69	5.00E-05	4.28E-04	Inorganic ion transport and metabolism	Sulfate transporter family
EVM0008766	25.03	7.76	1.69	2.30E-03	1.14E-02	--	--
EVM0002253	2.69	0.83	1.69	1.65E-03	8.65E-03	--	--
EVM0008881	197.99	60.98	1.70	5.00E-05	4.28E-04	--	Glycosyl hydrolase family 3 N terminal domain
EVM0001331	36.49	11.22	1.70	5.00E-05	4.28E-04	--	Ankyrin repeats (many copies)
EVM0011451	3.47	1.07	1.71	1.21E-02	4.36E-02	--	SH3-binding, glutamic acid-rich protein
EVM0000646	7.84	2.38	1.72	8.40E-03	3.26E-02	--	--

EVM0008978	19.12	5.81	1.72	5.00E-05	4.28E-04	Transcription	SNF2 family N-terminal domain
EVM0010171	25.83	7.83	1.72	5.00E-05	4.28E-04	Energy production and conversion	Aldo/keto reductase family
EVM0001895	12.48	3.78	1.72	1.50E-04	1.14E-03	--	--
EVM0001305	1.59	0.48	1.72	1.34E-02	4.72E-02	--	Domain of unknown function (DUF3328)
EVM0010291	32.34	9.78	1.72	2.00E-04	1.46E-03	--	MAPEG family
EVM0012190	3.58	1.08	1.73	2.50E-04	1.77E-03	Secondary metabolites biosynthesis, transport and catabolism	ABC transporter transmembrane region
EVM0003849	66.96	20.19	1.73	5.00E-05	4.28E-04	--	--
EVM0010242	6.79	2.05	1.73	1.50E-04	1.14E-03	Function unknown	WD domain, G-beta repeat
EVM0005614	93.07	27.97	1.73	5.00E-05	4.28E-04	--	--
EVM0003633	23.87	7.10	1.75	5.00E-05	4.28E-04	--	Pectate lyase superfamily protein
EVM0005680	12.19	3.61	1.76	5.00E-05	4.28E-04	Carbohydrate transport and metabolism	Major Facilitator Superfamily
EVM0002173	1.74	0.51	1.76	1.35E-02	4.76E-02	--	Rad51

EVM0009020	13.30	3.88	1.78	5.00E-05	4.28E-04	--	--
EVM0011477	29.16	8.49	1.78	5.00E-05	4.28E-04	Posttranslational modification, protein turnover, chaperones	Glutathione S-transferase, N-terminal domain
EVM0009637	38.46	11.18	1.78	5.00E-05	4.28E-04	General function prediction only	Epoxide hydrolase N terminus
EVM0005805	4.67	1.35	1.79	1.00E-03	5.70E-03	--	--
EVM0010323	71.21	20.60	1.79	5.00E-05	4.28E-04	Inorganic ion transport and metabolism	ATP-sulfurylase
EVM0010721	6.47	1.87	1.79	2.25E-03	1.12E-02	--	--
EVM0005713	1.33	0.38	1.80	1.43E-02	4.97E-02	--	Serine carboxypeptidase
EVM0008970	56.30	16.17	1.80	5.00E-05	4.28E-04	--	Cytochrome P450
EVM0003089	11.28	3.23	1.80	1.80E-03	9.29E-03	General function prediction only	Uncharacterised protein family (UPF0172)
EVM0003976	195.19	55.88	1.80	5.00E-05	4.28E-04	Amino acid transport and metabolism	Conserved region in glutamate synthase
EVM0010136	97.45	27.89	1.80	5.00E-05	4.28E-04	Carbohydrate transport and metabolism	Major Facilitator Superfamily
EVM0005194	2.02	0.58	1.81	5.90E-03	2.46E-02	--	Glycosyl hydrolases family 28

EVM0006726	35.85	10.17	1.82	5.00E-05	4.28E-04	Posttranslational modification, protein turnover, chaperones	ATPase family associated with various cellular activities (AAA)
EVM0007544	5.55	1.57	1.82	5.00E-05	4.28E-04	Posttranslational modification, protein turnover, chaperones	Eukaryotic aspartyl protease
EVM0011213	15.31	4.32	1.83	4.95E-03	2.13E-02	--	--
EVM0001872	6.38	1.80	1.83	4.00E-03	1.79E-02	--	--
EVM0005724	89.82	25.30	1.83	5.00E-05	4.28E-04	--	Protein of unknown function (Ytp1)
EVM0002854	96.47	27.12	1.83	5.00E-05	4.28E-04	Secondary metabolites biosynthesis, transport and catabolism	Multicopper oxidase
EVM0007881	2.86	0.80	1.83	4.60E-03	2.01E-02	--	Ubiquitin 3 binding protein But2 C-terminal domain
EVM0008303	3.71	1.04	1.83	4.45E-03	1.96E-02	Lipid transport and metabolism	short chain dehydrogenase
EVM0000934	2.46	0.69	1.84	7.35E-03	2.93E-02	--	Alpha/beta hydrolase family
EVM0003956	1.40	0.39	1.84	8.15E-03	3.18E-02	--	Glycosyl hydrolase family 7
EVM0005925	37.48	10.37	1.85	5.00E-05	4.28E-04	--	Cytochrome P450

EVM0006594	421.38	115.37	1.87	5.00E-05	4.28E-04	Amino acid transport and metabolism	Serine hydroxymethyltransferase
EVM0012269	442.14	120.91	1.87	5.00E-05	4.28E-04	Cell wall/membrane/envelope biogenesis	SIS domain
EVM0001482	3.51	0.95	1.88	3.20E-03	1.49E-02	Lipid transport and metabolism	short chain dehydrogenase
EVM0006030	41.62	11.29	1.88	5.00E-05	4.28E-04	--	--
EVM0002050	159.91	43.37	1.88	1.26E-02	4.49E-02	--	--
EVM0006479	34.65	9.23	1.91	5.00E-05	4.28E-04	--	RecF/RecN/SMC N terminal domain
EVM0009154	62.07	16.51	1.91	5.00E-05	4.28E-04	--	--
EVM0004904	16.97	4.49	1.92	5.00E-05	4.28E-04	--	FAD binding domain
EVM0007712	37.77	9.99	1.92	5.00E-05	4.28E-04	General function prediction only	Protein kinase domain
EVM0002674	8.85	2.34	1.92	5.00E-05	4.28E-04	Secondary metabolites biosynthesis, transport and catabolism	short chain dehydrogenase
EVM0005310	577.96	152.52	1.92	5.00E-05	4.28E-04	General function prediction only	Enoyl-(Acyl carrier protein) reductase
EVM0011435	55.96	14.72	1.93	5.00E-05	4.28E-04	Cytoskeleton	Myosin head (motor domain)

EVM0004571	1.17	0.31	1.93	4.45E-03	1.96E-02	--	Pectate lyase
EVM0002793	33.32	8.68	1.94	4.00E-04	2.65E-03	--	Cerato-platanin
EVM0000933	3.88	1.01	1.94	1.00E-04	7.96E-04	Energy production and conversion	Malic enzyme, NAD binding domain
EVM0009846	22.15	5.72	1.95	5.00E-05	4.28E-04	General function prediction only	Sugar (and other) transporter
EVM0006476	593.32	152.82	1.96	5.00E-05	4.28E-04	--	LVIVD repeat
EVM0009616	43.31	11.11	1.96	5.00E-05	4.28E-04	--	Ribosomal protein S21
EVM0005846	9.38	2.40	1.97	1.85E-03	9.52E-03	--	--
EVM0001888	15.78	4.04	1.97	5.00E-05	4.28E-04	--	--
EVM0012233	12.95	3.31	1.97	5.00E-05	4.28E-04	--	--
EVM0008308	2.22	0.57	1.97	1.85E-03	9.52E-03	--	--
EVM0008614	19.15	4.87	1.98	5.00E-05	4.28E-04	--	--
EVM0006744	7.97	2.02	1.98	5.00E-05	4.28E-04	General function prediction only	Carboxylesterase family
EVM0006489	267.22	67.64	1.98	5.00E-05	4.28E-04	--	--

EVM0003996	109.01	27.53	1.99	5.00E-05	4.28E-04	--	--
EVM0000637	86.49	21.67	2.00	5.00E-05	4.28E-04	--	Cytochrome P450
EVM0002953	38.71	9.67	2.00	5.00E-05	4.28E-04	--	Fungalysin metallopeptidase (M36)
EVM0005188	166.95	41.40	2.01	5.00E-05	4.28E-04	Inorganic ion transport and metabolism	Carbonic anhydrase
EVM0008197	49.64	12.24	2.02	5.00E-05	4.28E-04	Amino acid transport and metabolism	Carbon-nitrogen hydrolase
EVM0000202	149.45	36.66	2.03	5.00E-05	4.28E-04	--	Glycosyl transferase family group 2
EVM0010083	2.22	0.54	2.03	9.10E-03	3.48E-02	--	Domain of unknown function (DUF3328)
EVM0004219	256.53	62.65	2.03	5.00E-05	4.28E-04	--	NADH:flavin oxidoreductase / NADH oxidase family
EVM0001750	15.06	3.66	2.04	5.00E-05	4.28E-04	Amino acid transport and metabolism	Amino acid permease
EVM0007684	137.09	33.25	2.04	5.00E-05	4.28E-04	General function prediction only	Major Facilitator Superfamily
EVM0008827	3.91	0.95	2.05	4.05E-03	1.81E-02	--	--
EVM0005744	9.94	2.37	2.07	5.00E-05	4.28E-04	RNA processing and modification	AAA domain
EVM0009981	3.40	0.81	2.07	6.50E-04	3.98E-03	--	--

EVM0002420	19.64	4.64	2.08	1.07E-02	3.94E-02	--	--
EVM0006122	39.58	9.33	2.09	1.40E-03	7.55E-03	--	--
EVM0007573	3633.65	847.04	2.10	5.00E-05	4.28E-04	--	Fungal hydrophobin
EVM0001569	11.74	2.73	2.10	5.00E-05	4.28E-04	General function prediction only	Major Facilitator Superfamily
EVM0003690	10.87	2.50	2.12	5.00E-05	4.28E-04	--	Caspase domain
EVM0009599	5.52	1.27	2.12	5.00E-05	4.28E-04	--	TPR repeat
EVM0007764	19.32	4.41	2.13	5.00E-05	4.28E-04	--	SNF2 family N-terminal domain
EVM0002710	47.07	10.71	2.14	5.00E-05	4.28E-04	--	Aldo/keto reductase family
EVM0011356	5.26	1.19	2.15	5.00E-05	4.28E-04	--	Glyoxal oxidase N-terminus
EVM0011562	151.91	34.28	2.15	5.00E-05	4.28E-04	--	--
EVM0010117	8.23	1.85	2.16	5.00E-05	4.28E-04	--	--
EVM0009436	3.79	0.85	2.16	5.45E-03	2.31E-02	--	NADH:flavin oxidoreductase / NADH oxidase family
EVM0002747	1.73	0.38	2.18	4.00E-04	2.65E-03	RNA processing and modification	DEAD/DEAH box helicase

EVM0009153	26.15	5.75	2.18	5.00E-05	4.28E-04	--	--
EVM0008746	59.21	12.92	2.20	5.00E-05	4.28E-04	--	UvrD-like helicase C-terminal domain
EVM0005372	22.57	4.92	2.20	5.00E-05	4.28E-04	--	Alpha/beta hydrolase family
EVM0007492	233.52	50.77	2.20	3.50E-04	2.36E-03	Amino acid transport and metabolism	Cobalamin-independent synthase, Catalytic domain
EVM0003142	1.86	0.40	2.23	2.65E-03	1.28E-02	Carbohydrate transport and metabolism	Major Facilitator Superfamily
EVM0011658	8.49	1.80	2.24	1.50E-04	1.14E-03	Lipid transport and metabolism	Enoyl-CoA hydratase/isomerase family
EVM0002737	7.89	1.67	2.24	2.00E-04	1.46E-03	--	--
EVM0001737	97.00	20.42	2.25	5.00E-05	4.28E-04	Secondary metabolites biosynthesis, transport and catabolism	Cytochrome P450
EVM0011790	13.12	2.76	2.25	4.00E-04	2.65E-03	General function prediction only	Ras family
EVM0000478	56.73	11.75	2.27	5.00E-05	4.28E-04	Signal transduction mechanisms	Protein kinase domain
EVM0011687	46.34	9.58	2.27	5.00E-05	4.28E-04	Function unknown	Domain of unknown function (DUF3437)
EVM0007836	10.03	2.05	2.29	1.50E-04	1.14E-03	--	--

EVM0008735	635.35	129.24	2.30	5.00E-05	4.28E-04	--	NmrA-like family
EVM0007392	73.79	14.80	2.32	5.00E-05	4.28E-04	--	WSC domain
EVM0010471	10.43	2.05	2.35	5.00E-05	4.28E-04	--	Cytochrome P450
EVM0009169	589.43	115.82	2.35	5.00E-05	4.28E-04	--	--
EVM0009561	5.91	1.16	2.35	1.02E-02	3.80E-02	--	--
EVM0008408	7.88	1.54	2.36	3.45E-03	1.59E-02	--	--
EVM0001579	175.78	33.77	2.38	5.00E-05	4.28E-04	General function prediction only	Family of unknown function (DUF706)
EVM0007578	3.30	0.63	2.38	2.50E-04	1.77E-03	General function prediction only	GMC oxidoreductase
EVM0008758	1.13	0.21	2.40	2.40E-03	1.18E-02	--	--
EVM0011853	2.13	0.40	2.40	7.55E-03	2.99E-02	--	--
EVM0003903	39.03	7.38	2.40	5.00E-05	4.28E-04	--	--
EVM0010699	3005.31	563.67	2.41	2.45E-03	1.20E-02	Energy production and conversion	Aldo/keto reductase family
EVM0008722	340.50	62.89	2.44	5.00E-05	4.28E-04	Inorganic ion transport and metabolism	Alkaline phosphatase

EVM0008249	29.07	5.31	2.45	5.00E-05	4.28E-04	--	--
EVM0000318	3.51	0.64	2.46	4.10E-03	1.83E-02	--	--
EVM0002732	68.12	12.32	2.47	5.00E-05	4.28E-04	Function unknown	Cysteine-rich secretory protein family
EVM0009073	2.04	0.36	2.49	8.85E-03	3.40E-02	--	--
EVM0008569	15.71	2.79	2.49	3.55E-03	1.62E-02	--	--
EVM0000446	38.04	6.70	2.51	5.00E-05	4.28E-04	Signal transduction mechanisms	Carboxylesterase family
EVM0009588	51.43	9.03	2.51	5.00E-05	4.28E-04	Secondary metabolites biosynthesis, transport and catabolism	Cytochrome P450
EVM0008919	3.08	0.54	2.52	6.00E-04	3.72E-03	--	--
EVM0008117	18.49	3.19	2.54	5.00E-05	4.28E-04	--	--
EVM0005069	6.01	1.00	2.58	5.00E-05	4.28E-04	Energy production and conversion	FAD binding domain
EVM0009217	24.61	4.11	2.58	5.00E-05	4.28E-04	Secondary metabolites biosynthesis, transport and catabolism	Cytochrome P450
EVM0004536	18.84	3.09	2.61	1.00E-04	7.96E-04	--	Hydrophobic surface binding protein A

EVM0002733	34.79	5.51	2.66	5.00E-05	4.28E-04	Lipid transport and metabolism	Calcineurin-like phosphoesterase
EVM0007992	4.70	0.74	2.66	1.16E-02	4.20E-02	Carbohydrate transport and metabolism	Major intrinsic protein
EVM0008898	1620.69	255.57	2.66	5.00E-05	4.28E-04	--	--
EVM0006645	372.93	58.00	2.68	5.00E-05	4.28E-04	--	--
EVM0011914	105.82	16.29	2.70	5.00E-05	4.28E-04	--	Cerato-platanin
EVM0003422	67.28	10.35	2.70	5.00E-05	4.28E-04	General function prediction only	Zinc-binding dehydrogenase
EVM0010018	86.16	13.21	2.71	5.00E-05	4.28E-04	--	Fungal hydrophobin
EVM0000195	46.72	7.06	2.73	5.00E-05	4.28E-04	--	Serine carboxypeptidase S28
EVM0002281	47.04	7.08	2.73	5.00E-05	4.28E-04	--	--
EVM0009326	3.23	0.48	2.74	3.00E-04	2.07E-03	--	SMP-30/Gluconolaconase/LRE-like region
EVM0005169	1103.72	164.25	2.75	5.00E-05	4.28E-04	--	Peptidase family M28
EVM0004978	23.68	3.48	2.77	5.00E-05	4.28E-04	--	--
EVM0002125	20.91	3.05	2.78	5.00E-05	4.28E-04	General function prediction only	Major Facilitator Superfamily

EVM0001981	72.41	10.40	2.80	5.00E-05	4.28E-04	--	HD domain
EVM0007990	30.74	4.40	2.80	5.00E-05	4.28E-04	--	Carbonic anhydrase
EVM0002401	21.10	2.96	2.83	5.00E-05	4.28E-04	General function prediction only	O-methyltransferase
EVM0003134	60.76	8.07	2.91	5.00E-05	4.28E-04	--	--
EVM0009189	2.59	0.34	2.92	1.12E-02	4.08E-02	--	Pheromone A receptor
EVM0008755	71.14	9.38	2.92	5.00E-05	4.28E-04	--	Cupin domain
EVM0011006	18.23	2.40	2.93	5.00E-05	4.28E-04	--	Protein of unknown function (DUF1524)
EVM0002399	134.22	16.43	3.03	5.00E-05	4.28E-04	--	NADH(P)-binding
EVM0009049	11.25	1.37	3.04	5.00E-05	4.28E-04	--	--
EVM0002629	803.85	96.36	3.06	5.50E-04	3.46E-03	--	FAD binding domain
EVM0004350	2640.24	313.04	3.08	5.00E-05	4.28E-04	Energy production and conversion	Aldo/keto reductase family
EVM0005375	15.40	1.76	3.13	5.00E-05	4.28E-04	--	--

EVM0008112	718.63	81.43	3.14	5.00E-05	4.28E-04	Intracellular trafficking, secretion, and vesicular transport	ER lumen protein retaining receptor
EVM0010863	66.70	7.05	3.24	5.00E-05	4.28E-04	General function prediction only	O-methyltransferase
EVM0011267	374.31	39.37	3.25	5.00E-05	4.28E-04	Energy production and conversion	Aldo/keto reductase family
EVM0006935	10.54	1.08	3.28	5.00E-05	4.28E-04	--	UbiA prenyltransferase family
EVM0004142	565.17	57.81	3.29	5.00E-05	4.28E-04	Intracellular trafficking, secretion, and vesicular transport	Vacuolar sorting protein 39 domain 1
EVM0004026	921.67	92.37	3.32	5.00E-05	4.28E-04	Secondary metabolites biosynthesis, transport and catabolism	short chain dehydrogenase
EVM0002861	1.38	0.14	3.35	6.00E-04	3.72E-03	--	Transposase family tnp2
EVM0002202	13.30	1.30	3.36	5.00E-05	4.28E-04	--	Putative glycosyl hydrolase of unknown function (DUF1680)
EVM0000184	152.63	14.70	3.38	5.00E-05	4.28E-04	Signal transduction mechanisms	OPT oligopeptide transporter protein
EVM0011894	271.14	24.46	3.47	5.00E-05	4.28E-04	Secondary metabolites biosynthesis, transport and catabolism	Cytochrome P450
EVM0006874	40.47	3.64	3.48	5.00E-05	4.28E-04	--	--

EVM0009915	131.45	11.52	3.51	5.00E-05	4.28E-04	Secondary metabolites biosynthesis, transport and catabolism	Cytochrome P450
EVM0010798	436.15	36.00	3.60	5.00E-05	4.28E-04	--	Stress responsive A/B Barrel Domain
EVM0004300	55.30	4.50	3.62	5.00E-05	4.28E-04	Secondary metabolites biosynthesis, transport and catabolism	Cytochrome P450
EVM0009612	34.41	2.36	3.86	5.00E-05	4.28E-04	--	--
EVM0007642	294.85	19.17	3.94	5.00E-05	4.28E-04	--	Pregnancy-associated plasma protein-A
EVM0010247	268.99	17.39	3.95	5.00E-05	4.28E-04	Secondary metabolites biosynthesis, transport and catabolism	Cytochrome P450
EVM0010621	29.42	1.85	3.99	5.00E-05	4.28E-04	--	Deuterolysin metalloprotease (M35) family
EVM0008010	4.56	0.28	4.03	2.65E-03	1.28E-02	--	--
EVM0006001	83.19	4.72	4.14	5.00E-05	4.28E-04	--	--
EVM0005984	43.90	1.67	4.71	5.00E-05	4.28E-04	--	CVNH domain
EVM0003375	46.43	1.65	4.82	1.50E-04	1.14E-03	--	--

EVM0001256	107.28	3.62	4.89	5.00E-05	4.28E-04	--	LVIVD repeat
EVM0007243	71.61	2.20	5.02	5.00E-05	4.28E-04	Secondary metabolites biosynthesis, transport and catabolism	Pyridine nucleotide-disulphide oxidoreductase
EVM0003814	7.65	0.23	5.08	5.00E-05	4.28E-04	General function prediction only	GMC oxidoreductase
EVM0008710	275.29	7.98	5.11	5.00E-05	4.28E-04	Posttranslational modification, protein turnover, chaperones	Subtilase family
EVM0007266	1631.18	39.90	5.35	5.00E-05	4.28E-04	--	--
EVM0012169	227.05	4.80	5.56	5.00E-05	4.28E-04	--	CVNH domain
EVM0003022	448.11	7.75	5.85	5.00E-05	4.28E-04	Amino acid transport and metabolism	FAD dependent oxidoreductase
EVM0009739	700.28	9.20	6.25	5.00E-05	4.28E-04	Energy production and conversion	Aldo/keto reductase family
EVM0005722	492.55	6.35	6.28	5.00E-05	4.28E-04	General function prediction only	O-methyltransferase

Data Set S5 Information of DEGs in R3 vs Z3

gene ID	R3 value	Z3 value	log2(fold change)	p value	q value	KOG class	Pfam annotation
EVM0011351	56.96	132.95	-1.22	5.00E-05	4.28E-04	--	--
EVM0004614	3.68	2575.92	-9.45	2.60E-03	1.26E-02	--	--
EVM0004221	1.70	207.12	-6.93	1.00E-04	7.96E-04	--	--
EVM0004083	7.40	899.59	-6.92	5.00E-05	4.28E-04	--	--
EVM0002644	53.67	5739.08	-6.74	5.00E-05	4.28E-04	--	--
EVM0000746	20.17	1635.09	-6.34	5.00E-05	4.28E-04	--	--
EVM0003786	2.50	161.00	-6.01	5.00E-05	4.28E-04	General function prediction only	Major Facilitator Superfamily
EVM0002673	4.93	315.29	-6.00	5.00E-05	4.28E-04	--	--
EVM0004440	0.99	59.51	-5.91	2.20E-03	1.10E-02	--	ribonuclease
EVM0006723	29.57	1458.58	-5.62	5.00E-05	4.28E-04	--	Conidiation protein 6
EVM0002148	18.94	844.41	-5.48	5.00E-05	4.28E-04	--	Uncharacterised protein family (UPF0197)
EVM0002607	13.49	383.22	-4.83	5.00E-05	4.28E-04	Lipid transport and metabolism	Cupin-like domain

EVM0003267	1260.86	34941.10	-4.79	5.00E-05	4.28E-04	--	Conidiation protein 6
EVM0007042	38.39	1037.88	-4.76	5.00E-05	4.28E-04	--	Hemerythrin HHE cation binding domain
EVM0004233	16.74	447.19	-4.74	5.00E-05	4.28E-04	--	--
EVM0008153	19.61	516.67	-4.72	5.00E-05	4.28E-04	--	--
EVM0001604	106.89	2660.18	-4.64	5.00E-05	4.28E-04	General function prediction only	Enoyl-(Acyl carrier protein) reductase
EVM0003583	56.60	1326.27	-4.55	5.00E-05	4.28E-04	--	--
EVM0003588	7.36	159.95	-4.44	5.00E-05	4.28E-04	Secondary metabolites biosynthesis, transport and catabolism	Cytochrome P450
EVM0012086	29.78	636.01	-4.42	5.00E-05	4.28E-04	Carbohydrate transport and metabolism	Major Facilitator Superfamily
EVM0005861	9.29	197.13	-4.41	5.00E-05	4.28E-04	Inorganic ion transport and metabolism	Catalase
EVM0002926	34.17	725.08	-4.41	5.00E-05	4.28E-04	General function prediction only	Enoyl-(Acyl carrier protein) reductase
EVM0011108	1.21	25.37	-4.39	3.10E-03	1.45E-02	--	Fungal hydrophobin
EVM0009711	51.83	1034.42	-4.32	5.00E-05	4.28E-04	--	Fungal hydrophobin

EVM0010264	12.22	226.65	-4.21	5.00E-05	4.28E-04	Function unknown	Thioesterase superfamily
EVM0006402	24.68	454.50	-4.20	5.00E-05	4.28E-04	--	--
EVM0012049	3.32	59.99	-4.17	5.00E-05	4.28E-04	--	--
EVM0009623	30.69	548.84	-4.16	5.00E-05	4.28E-04	--	--
EVM0000056	2050.62	34018.20	-4.05	5.00E-05	4.28E-04	--	Aegerolysin
EVM0011343	19.88	328.37	-4.05	5.00E-05	4.28E-04	Posttranslational modification, protein turnover, chaperones	Glutaredoxin
EVM0005139	37.04	591.64	-4.00	5.00E-05	4.28E-04	--	--
EVM0007399	3.78	60.16	-3.99	5.00E-05	4.28E-04	--	Common central domain of tyrosinase
EVM0007843	5.15	80.42	-3.97	5.00E-05	4.28E-04	--	Dyp-type peroxidase family
EVM0009923	177.06	2728.98	-3.95	5.00E-05	4.28E-04	#N/A	#N/A
EVM0003700	0.23	3.47	-3.93	5.00E-05	4.28E-04	Replication, recombination and repair	DEAD/DEAH box helicase
EVM0006875	12.58	191.09	-3.93	5.00E-05	4.28E-04	--	Polysaccharide deacetylase

EVM0004031	9.34	141.70	-3.92	5.00E-05	4.28E-04	RNA processing and modification	Eukaryotic rRNA processing protein EBP2
EVM0001738	145.06	2200.62	-3.92	5.00E-05	4.28E-04	Lipid transport and metabolism	Phophatidylserine decarboxylase
EVM0000901	1.62	24.44	-3.92	5.00E-05	4.28E-04	Carbohydrate transport and metabolism	Starch binding domain
EVM0007517	15.36	224.72	-3.87	5.00E-05	4.28E-04	--	--
EVM0007912	1.45	21.13	-3.86	5.00E-05	4.28E-04	--	--
EVM0009499	0.94	13.58	-3.86	8.00E-04	4.73E-03	General function prediction only	Phosphatidylethanolamine-binding protein
EVM0003373	0.93	13.38	-3.84	1.50E-04	1.14E-03	--	--
EVM0008982	1.22	17.36	-3.83	5.00E-05	4.28E-04	--	--
EVM0004346	314.22	4460.27	-3.83	5.00E-05	4.28E-04	--	--
EVM0003167	37.21	516.16	-3.79	5.00E-05	4.28E-04	--	--
EVM0003528	21.72	301.01	-3.79	5.00E-05	4.28E-04	--	--
EVM0001520	2.40	33.19	-3.79	5.00E-05	4.28E-04	--	--

EVM0004488	12.19	166.23	-3.77	5.00E-05	4.28E-04	Secondary metabolites biosynthesis, transport and catabolism	Multicopper oxidase
EVM0006981	11.02	147.01	-3.74	5.00E-05	4.28E-04	--	Rrp15p
EVM0004778	27.45	352.76	-3.68	5.00E-05	4.28E-04	General function prediction only	Thioesterase superfamily
EVM0005127	51.92	657.87	-3.66	5.00E-05	4.28E-04	--	Gamma interferon inducible lysosomal thiol reductase (GILT)
EVM0002081	1.38	17.51	-3.66	5.00E-05	4.28E-04	Signal transduction mechanisms	FHA domain
EVM0004302	6.99	87.03	-3.64	5.00E-05	4.28E-04	--	--
EVM0000062	12.99	160.27	-3.63	5.00E-05	4.28E-04	--	--
EVM0007098	4.91	59.59	-3.60	5.00E-05	4.28E-04	--	FAD binding domain of DNA photolyase
EVM0006053	0.36	4.29	-3.58	1.00E-04	7.96E-04	--	--
EVM0008167	14.51	172.95	-3.57	5.00E-05	4.28E-04	General function prediction only	Animal haem peroxidase
EVM0005865	356.73	4175.63	-3.55	5.00E-05	4.28E-04	General function prediction only	Proteolipid membrane potential modulator
EVM0000829	28.77	330.89	-3.52	5.00E-05	4.28E-04	--	--

EVM0010422	4.02	45.67	-3.51	5.00E-05	4.28E-04	Posttranslational modification, protein turnover, chaperones	DnaJ domain
EVM0006251	5.01	56.48	-3.50	5.00E-05	4.28E-04	Function unknown	Surfeit locus protein 6
EVM0002391	123.60	1386.81	-3.49	5.00E-05	4.28E-04	--	--
EVM0007514	18.82	210.86	-3.49	5.00E-05	4.28E-04	--	RTA1 like protein
EVM0011052	0.40	4.39	-3.47	9.80E-03	3.69E-02	--	Glycosyl hydrolase family 12
EVM0004767	66.02	720.49	-3.45	1.50E-04	1.14E-03	--	4F5 protein family
EVM0000880	11.15	121.55	-3.45	5.00E-05	4.28E-04	--	Glycosyl hydrolases family 15
EVM0007566	404.06	4360.24	-3.43	5.00E-05	4.28E-04	--	--
EVM0001141	64.72	692.55	-3.42	5.00E-05	4.28E-04	--	--
EVM0004818	10.11	107.07	-3.41	5.00E-05	4.28E-04	#N/A	#N/A
EVM0002176	7.61	80.43	-3.40	5.00E-05	4.28E-04	--	--
EVM0002270	4.45	46.89	-3.40	5.00E-05	4.28E-04	--	--

EVM0009090	14.37	149.64	-3.38	5.00E-05	4.28E-04	--	--
EVM0009999	3.21	33.16	-3.37	3.00E-04	2.07E-03	--	Rare lipoprotein A (RlpA)-like double-psi beta-barrel
EVM0003866	40.50	415.20	-3.36	5.00E-05	4.28E-04	--	DAD family
EVM0003008	23.24	237.88	-3.36	5.00E-05	4.28E-04	--	--
EVM0008891	7.95	81.18	-3.35	5.00E-05	4.28E-04	Amino acid transport and metabolism	Histidine biosynthesis protein
EVM0011587	73.31	746.54	-3.35	5.00E-05	4.28E-04	--	--
EVM0004953	34.63	348.15	-3.33	5.00E-05	4.28E-04	--	--
EVM0010633	518.20	5169.30	-3.32	5.00E-05	4.28E-04	Energy production and conversion	--
EVM0005881	2.01	19.68	-3.29	5.00E-05	4.28E-04	--	Small nuclear RNA activating complex (SNAPc), subunit SNAP43
EVM0012010	22.49	218.63	-3.28	5.00E-05	4.28E-04	--	--
EVM0007894	1.30	12.65	-3.28	7.50E-04	4.49E-03	General function prediction only	Zinc finger, C2H2 type
EVM0003732	0.65	6.26	-3.26	5.00E-05	4.28E-04	--	--

EVM0005546	12.12	116.02	-3.26	5.00E-05	4.28E-04	General function prediction only	MatE
EVM0001104	4.48	41.99	-3.23	5.00E-05	4.28E-04	--	--
EVM0010306	7.12	66.62	-3.23	5.00E-05	4.28E-04	--	--
EVM0004307	0.42	3.89	-3.20	1.10E-02	4.04E-02	Carbohydrate transport and metabolism	Glycosyl hydrolase family 47
EVM0001246	11.08	101.35	-3.19	5.00E-05	4.28E-04	--	--
EVM0004958	11.61	106.06	-3.19	5.00E-05	4.28E-04	--	Regulator of G protein signaling domain
EVM0006028	26.35	235.43	-3.16	5.00E-05	4.28E-04	--	--
EVM0008242	0.81	7.17	-3.15	1.00E-03	5.70E-03	--	Glycosyl hydrolase family 12
EVM0007445	2.65	23.57	-3.15	5.00E-05	4.28E-04	--	--
EVM0008171	0.60	5.31	-3.15	5.65E-03	2.37E-02	--	Cerato-platanin
EVM0005968	2.09	18.53	-3.15	5.00E-05	4.28E-04	--	--
EVM0000435	26.09	230.91	-3.15	5.00E-05	4.28E-04	General function prediction only	Glycosyl transferase family 2
EVM0010210	196.82	1731.08	-3.14	5.00E-05	4.28E-04	--	--

EVM0007958	2.44	21.40	-3.13	5.00E-05	4.28E-04	Secondary metabolites biosynthesis, transport and catabolism	Multicopper oxidase
EVM0007507	11.64	101.72	-3.13	5.00E-05	4.28E-04	--	--
EVM0007339	11.51	100.46	-3.13	5.00E-05	4.28E-04	--	SMP-30/Gluconolaconase/LRE-like region
EVM0011013	6.39	55.62	-3.12	2.15E-03	1.08E-02	Function unknown	COQ9
EVM0001661	6.38	55.31	-3.12	5.00E-05	4.28E-04	General function prediction only	Kinase phosphorylation protein
EVM0009807	1330.92	11531.60	-3.12	5.00E-05	4.28E-04	--	Mitochondrial F1-F0 ATP synthase subunit F of fungi
EVM0002451	2.95	25.34	-3.10	5.00E-05	4.28E-04	--	--
EVM0009425	9.72	82.79	-3.09	5.00E-05	4.28E-04	--	--
EVM0000681	6.75	57.35	-3.09	5.00E-05	4.28E-04	--	--
EVM0000282	213.49	1807.70	-3.08	5.00E-05	4.28E-04	--	--
EVM0004965	2.64	22.29	-3.08	5.00E-05	4.28E-04	Function unknown	Tho complex subunit 7
EVM0009798	18.72	156.91	-3.07	5.00E-05	4.28E-04	RNA processing and modification	RNA recognition motif. (a.k.a. RRM, RBD, or RNP domain)

EVM0010440	40.98	343.16	-3.07	5.00E-05	4.28E-04	--	Cytochrome c/c1 heme lyase
EVM0010609	92.23	771.31	-3.06	5.00E-05	4.28E-04	Intracellular trafficking, secretion, and vesicular transport	Translocation protein Sec62
EVM0005915	12.02	100.28	-3.06	5.00E-05	4.28E-04	--	Lipase (class 3)
EVM0010015	0.37	3.03	-3.05	6.00E-04	3.72E-03	#N/A	#N/A
EVM0008621	1.65	13.59	-3.04	5.00E-05	4.28E-04	--	--
EVM0005647	9.18	75.61	-3.04	5.00E-05	4.28E-04	--	Alpha/beta hydrolase family
EVM0001452	3.30	26.78	-3.02	5.00E-05	4.28E-04	--	--
EVM0007975	0.69	5.54	-3.01	5.00E-05	4.28E-04	--	GDSL-like Lipase/Acylhydrolase
EVM0008487	0.15	1.20	-3.01	5.65E-03	2.37E-02	--	--
EVM0001814	3962.69	31870.50	-3.01	5.00E-05	4.28E-04	--	Ricin-type beta-trefoil lectin domain-like
EVM0010253	8.77	70.12	-3.00	5.00E-05	4.28E-04	Replication, recombination and repair	Uracil DNA glycosylase superfamily
EVM0002307	53.31	424.82	-2.99	5.00E-05	4.28E-04	--	--

EVM0005795	5.19	41.33	-2.99	5.00E-05	4.28E-04	Chromatin structure and dynamics	Core histone H2A/H2B/H3/H4
EVM0005630	80.09	632.92	-2.98	5.00E-05	4.28E-04	Translation, ribosomal structure and biogenesis	Mitochondrial ribosomal protein L51 / S25 / CI-B8 domain
EVM0007323	388.35	3065.78	-2.98	5.00E-05	4.28E-04	Function unknown	Domain of unknown function (DUF543)
EVM0008586	8.70	68.23	-2.97	5.00E-05	4.28E-04	--	Chromatin remodelling complex Rsc7/Swp82 subunit
EVM0003649	32.91	258.01	-2.97	5.00E-05	4.28E-04	General function prediction only	Thioesterase superfamily
EVM0009041	5.27	40.96	-2.96	5.00E-05	4.28E-04	--	--
EVM0007470	1.99	15.18	-2.93	5.00E-05	4.28E-04	--	--
EVM0007261	165.82	1267.30	-2.93	5.00E-05	4.28E-04	--	--
EVM0009031	4.37	33.21	-2.93	5.00E-05	4.28E-04	--	Glycosyl hydrolases family 43
EVM0010219	6.43	48.55	-2.92	1.00E-04	7.96E-04	--	--
EVM0006917	1.68	12.58	-2.91	5.00E-05	4.28E-04	--	--
EVM0005367	0.91	6.84	-2.91	5.00E-05	4.28E-04	--	--
EVM0002697	4.74	35.45	-2.90	5.00E-05	4.28E-04	--	--

EVM0000461	28.01	208.83	-2.90	5.00E-05	4.28E-04	--	--
EVM0005079	33.60	246.26	-2.87	5.00E-05	4.28E-04	Coenzyme transport and metabolism	Pantoate-beta-alanine ligase
EVM0000390	4.02	29.31	-2.87	5.00E-05	4.28E-04	Amino acid transport and metabolism	Cys/Met metabolism PLP-dependent enzyme
EVM0010785	21.95	159.97	-2.87	5.00E-05	4.28E-04	--	Rpp20 subunit of nuclear RNase MRP and P
EVM0005285	755.82	5491.75	-2.86	5.00E-05	4.28E-04	--	--
EVM0006195	13.10	94.70	-2.85	5.00E-05	4.28E-04	--	pre-mRNA splicing factor component
EVM0000780	112.49	810.30	-2.85	5.00E-05	4.28E-04	--	--
EVM0007187	3.62	26.03	-2.85	5.00E-05	4.28E-04	--	Pregnancy-associated plasma protein-A
EVM0011536	15.82	112.12	-2.83	5.00E-05	4.28E-04	--	Protein of unknown function (DUF3445)
EVM0008407	4.02	28.42	-2.82	1.15E-02	4.19E-02	--	--
EVM0000393	281.92	1992.78	-2.82	5.00E-05	4.28E-04	--	--
EVM0005036	14.34	101.37	-2.82	5.00E-05	4.28E-04	--	--

EVM0005353	1.36	9.61	-2.82	5.00E-05	4.28E-04	Posttranslational modification, protein turnover, chaperones	Peptidase family M50
EVM0008632	5.35	37.76	-2.82	5.00E-05	4.28E-04	Posttranslational modification, protein turnover, chaperones	Isoprenylcysteine carboxyl methyltransferase (ICMT) family
EVM0006543	2.71	19.06	-2.81	5.00E-05	4.28E-04	--	--
EVM0004368	1.98	13.84	-2.81	6.50E-04	3.98E-03	--	--
EVM0004446	6.20	43.19	-2.80	5.00E-05	4.28E-04	Posttranslational modification, protein turnover, chaperones	Isoprenylcysteine carboxyl methyltransferase (ICMT) family
EVM0000999	2.18	15.15	-2.80	5.00E-05	4.28E-04	Signal transduction mechanisms	Protein kinase domain
EVM0001122	25.05	172.64	-2.79	5.00E-05	4.28E-04	RNA processing and modification	Fibrillarin
EVM0004549	201.58	1386.51	-2.78	5.00E-05	4.28E-04	Lipid transport and metabolism	HIUase/Transthyretin family
EVM0003609	122.45	841.86	-2.78	5.00E-05	4.28E-04	--	Cytochrome oxidase c subunit VIb
EVM0008465	46.77	319.96	-2.77	5.00E-05	4.28E-04	RNA processing and modification	DEAD/DEAH box helicase
EVM0000819	2.35	15.96	-2.77	5.00E-05	4.28E-04	Transcription	SNF2 family N-terminal domain

EVM0000515	3.16	21.43	-2.76	5.00E-05	4.28E-04	--	--
EVM0008626	27.36	185.52	-2.76	5.00E-05	4.28E-04	--	--
EVM0006749	14.17	95.01	-2.75	5.00E-05	4.28E-04	--	Membrane bound O-acyl transferase family
EVM0002711	15.39	103.19	-2.75	5.00E-05	4.28E-04	General function prediction only	La domain
EVM0010273	1.44	9.64	-2.74	5.00E-05	4.28E-04	--	--
EVM0002682	65.55	435.15	-2.73	5.00E-05	4.28E-04	Transcription	RNA polymerase Rpb6
EVM0012109	3.66	24.24	-2.73	2.50E-04	1.77E-03	--	Rare lipoprotein A (RlpA)-like double-psi beta-barrel
EVM0005453	23.36	154.28	-2.72	5.00E-05	4.28E-04	--	--
EVM0004771	16.36	107.80	-2.72	5.00E-05	4.28E-04	--	--
EVM0010419	2.25	14.74	-2.71	5.00E-05	4.28E-04	--	Mis12-Mtw1 protein family
EVM0005254	36.33	238.27	-2.71	5.00E-05	4.28E-04	--	--
EVM0009591	6.85	44.76	-2.71	5.00E-05	4.28E-04	Amino acid transport and metabolism	Homocysteine S-methyltransferase
EVM0007540	3.90	25.42	-2.70	5.00E-05	4.28E-04	--	--

EVM0008163	1.87	12.12	-2.70	5.00E-05	4.28E-04	--	--
EVM0008966	23.39	151.06	-2.69	5.00E-05	4.28E-04	--	--
EVM0006896	66.84	430.23	-2.69	5.00E-05	4.28E-04	--	Protein of unknown function (DUF4449)
EVM0006637	2.27	14.62	-2.68	5.00E-05	4.28E-04	Posttranslational modification, protein turnover, chaperones	Prolyl oligopeptidase family
EVM0012244	0.73	4.64	-2.68	7.00E-04	4.23E-03	--	--
EVM0001964	4.59	29.32	-2.68	5.00E-05	4.28E-04	--	--
EVM0010982	7.81	49.80	-2.67	5.00E-05	4.28E-04	--	--
EVM0010935	16.67	106.17	-2.67	5.00E-05	4.28E-04	General function prediction only	U3-containing 90S pre-ribosomal complex subunit
EVM0005758	38.11	241.58	-2.66	5.00E-05	4.28E-04	Lipid transport and metabolism	Putative undecaprenyl diphosphate synthase
EVM0004866	125.43	793.16	-2.66	5.00E-05	4.28E-04	Function unknown	Uncharacterised protein family (UPF0121)
EVM0010855	10.87	68.21	-2.65	5.00E-05	4.28E-04	--	--
EVM0005272	404.12	2535.69	-2.65	5.00E-05	4.28E-04	--	Ubiquinol-cytochrome-c reductase complex subunit (QCR10)

EVM0009146	0.76	4.78	-2.65	1.35E-03	7.33E-03	--	Glycosyl hydrolase family 61
EVM0006314	1.77	11.01	-2.63	5.00E-05	4.28E-04	--	--
EVM0010779	201.51	1248.67	-2.63	5.00E-05	4.28E-04	Intracellular trafficking, secretion, and vesicular transport	SURF4 family
EVM0005691	0.56	3.42	-2.61	2.00E-04	1.46E-03	--	Cytochrome P450
EVM0008537	2311.19	14034.10	-2.60	5.00E-05	4.28E-04	Posttranslational modification, protein turnover, chaperones	Hsp20/alpha crystallin family
EVM0011432	7.16	43.40	-2.60	5.00E-05	4.28E-04	Lipid transport and metabolism	SacI homology domain
EVM0007668	174.19	1051.10	-2.59	5.00E-05	4.28E-04	Energy production and conversion	CHCH domain
EVM0004405	1.28	7.75	-2.59	5.00E-05	4.28E-04	--	--
EVM0004582	1.40	8.42	-2.59	2.00E-04	1.46E-03	--	--
EVM0000430	24.59	147.56	-2.59	5.00E-05	4.28E-04	--	Polyketide cyclase / dehydrase and lipid transport
EVM0005980	5.06	30.28	-2.58	5.00E-05	4.28E-04	General function prediction only	Animal haem peroxidase
EVM0010820	1394.39	8339.69	-2.58	5.00E-05	4.28E-04	Energy production and conversion	Cytochrome oxidase c subunit VIb

EVM0007319	3.68	21.83	-2.57	5.00E-05	4.28E-04	--	--
EVM0005717	29.56	175.06	-2.57	5.00E-05	4.28E-04	--	--
EVM0005279	307.74	1820.55	-2.56	5.00E-05	4.28E-04	--	Stress responsive A/B Barrel Domain
EVM0002416	76.01	447.18	-2.56	5.00E-05	4.28E-04	Translation, ribosomal structure and biogenesis	Ribosomal protein S16
EVM0007964	20.41	119.85	-2.55	5.00E-05	4.28E-04	Lipid transport and metabolism	Calcineurin-like phosphoesterase
EVM0001355	149.31	872.19	-2.55	5.00E-05	4.28E-04	--	--
EVM0002257	26.14	152.50	-2.54	5.00E-05	4.28E-04	--	--
EVM0004341	65.39	380.43	-2.54	5.00E-05	4.28E-04	Function unknown	CSL zinc finger
EVM0010213	681.01	3955.13	-2.54	5.00E-05	4.28E-04	--	--
EVM0007597	11.28	64.84	-2.52	5.00E-05	4.28E-04	--	--
EVM0006670	1.40	8.05	-2.52	1.43E-02	4.98E-02	Secondary metabolites biosynthesis, transport and catabolism	Cytochrome P450
EVM0008283	23.64	135.66	-2.52	5.00E-05	4.28E-04	--	Fungal hydrophobin

EVM0011270	6.95	39.84	-2.52	5.00E-05	4.28E-04	--	cAMP phosphodiesterases class-II
EVM0001693	1.82	10.39	-2.52	5.00E-05	4.28E-04	--	--
EVM0002898	14.77	84.06	-2.51	5.00E-05	4.28E-04	Transcription	RNA polymerase Rpb3/RpoA insert domain
EVM0007198	147.39	838.01	-2.51	5.00E-05	4.28E-04	--	--
EVM0004911	288.88	1632.91	-2.50	5.00E-05	4.28E-04	--	--
EVM0002821	56.05	316.44	-2.50	5.00E-05	4.28E-04	--	--
EVM0003955	1.06	5.94	-2.49	6.50E-04	3.98E-03	--	--
EVM0002923	2245.12	12605.60	-2.49	5.00E-05	4.28E-04	--	CsbD-like
EVM0008645	25.22	141.57	-2.49	5.00E-05	4.28E-04	--	--
EVM0010141	40.37	226.56	-2.49	5.00E-05	4.28E-04	General function prediction only	Prp19/Pso4-like
EVM0001054	19.35	107.97	-2.48	5.00E-05	4.28E-04	--	--
EVM0007670	19.18	106.90	-2.48	5.00E-05	4.28E-04	--	Domain of unknown function (DUF4112)
EVM0002071	19377.00	107959.00	-2.48	5.00E-05	4.28E-04	--	Aegerolysin

EVM0011968	1.96	10.89	-2.48	5.00E-05	4.28E-04	--	Pectate lyase
EVM0006247	63.68	351.91	-2.47	5.00E-05	4.28E-04	General function prediction only	Zinc finger, C2H2 type
EVM0000423	89.05	491.73	-2.47	5.00E-05	4.28E-04	#N/A	#N/A
EVM0003258	29.24	161.41	-2.46	5.00E-05	4.28E-04	Posttranslational modification, protein turnover, chaperones	ThiF family
EVM0000069	7.73	42.63	-2.46	5.00E-05	4.28E-04	--	--
EVM0005130	4.19	23.09	-2.46	5.00E-05	4.28E-04	--	--
EVM0008730	17.63	96.77	-2.46	5.00E-05	4.28E-04	--	--
EVM0000406	3.54	19.43	-2.46	5.00E-05	4.28E-04	--	Protein kinase domain
EVM0010485	6.31	34.44	-2.45	5.00E-05	4.28E-04	--	Cytochrome P450
EVM0004698	60.10	326.74	-2.44	5.00E-05	4.28E-04	General function prediction only	Hypoxia induced protein conserved region
EVM0005508	79.05	428.98	-2.44	5.00E-05	4.28E-04	Posttranslational modification, protein turnover, chaperones	OST3 / OST6 family
EVM0012239	0.99	5.37	-2.44	5.00E-05	4.28E-04	--	--

EVM0006881	14.67	79.43	-2.44	5.00E-05	4.28E-04	--	NUDIX domain
EVM0000983	1.81	9.78	-2.43	5.00E-03	2.15E-02	--	--
EVM0008481	7.22	38.97	-2.43	5.00E-05	4.28E-04	Inorganic ion transport and metabolism	CorA-like Mg2+ transporter protein
EVM0007852	384.44	2074.10	-2.43	5.00E-05	4.28E-04	Posttranslational modification, protein turnover, chaperones	DnaJ C terminal domain
EVM0001411	0.86	4.62	-2.43	5.00E-05	4.28E-04	--	Cellulase (glycosyl hydrolase family 5)
EVM0006622	6.54	35.26	-2.43	5.00E-05	4.28E-04	--	--
EVM0008252	5.30	28.53	-2.43	5.00E-05	4.28E-04	--	--
EVM0001314	42.21	226.53	-2.42	5.00E-05	4.28E-04	--	--
EVM0011097	183.48	981.53	-2.42	5.00E-05	4.28E-04	--	RNA recognition motif. (a.k.a. RRM, RBD, or RNP domain)
EVM0001031	5.23	27.96	-2.42	5.00E-05	4.28E-04	Carbohydrate transport and metabolism	Major Facilitator Superfamily
EVM0009398	1.90	10.13	-2.41	7.60E-03	3.01E-02	--	--
EVM0000486	1.52	8.06	-2.41	5.00E-05	4.28E-04	Posttranslational modification, protein turnover, chaperones	ATPase family associated with various cellular activities (AAA)

EVM0012235	314.74	1671.98	-2.41	5.00E-05	4.28E-04	Posttranslational modification, protein turnover, chaperones	Cyclophilin type peptidyl-prolyl cis-trans isomerase/CLD
EVM0009733	12.02	63.75	-2.41	5.00E-05	4.28E-04	--	--
EVM0001647	35.86	189.11	-2.40	5.00E-05	4.28E-04	General function prediction only	Zinc-finger double-stranded RNA-binding
EVM0003560	13.70	72.15	-2.40	1.50E-03	8.00E-03	--	G-patch domain
EVM0004933	467.93	2452.18	-2.39	5.00E-05	4.28E-04	Posttranslational modification, protein turnover, chaperones	Sec61beta family
EVM0002358	13.68	71.67	-2.39	5.00E-05	4.28E-04	--	--
EVM0002266	177.80	930.24	-2.39	5.00E-05	4.28E-04	--	Protein of unknown function (DUF3429)
EVM0010981	290.73	1516.73	-2.38	5.00E-05	4.28E-04	Function unknown	Activator of Hsp90 ATPase, N-terminal
EVM0003855	3.50	18.23	-2.38	5.00E-05	4.28E-04	Signal transduction mechanisms	Protein kinase domain
EVM0006689	12.72	66.24	-2.38	5.00E-05	4.28E-04	General function prediction only	--
EVM0003721	84.33	438.78	-2.38	5.00E-05	4.28E-04	--	--
EVM0001192	64.77	336.77	-2.38	5.00E-05	4.28E-04	Transcription	RNA polymerase Rpb3/Rpb11 dimerisation domain

EVM0004228	12.13	62.28	-2.36	5.00E-05	4.28E-04	Translation, ribosomal structure and biogenesis	Brix domain
EVM0005497	53.46	274.54	-2.36	5.00E-05	4.28E-04	--	--
EVM0011102	4.21	21.56	-2.36	5.00E-05	4.28E-04	--	--
EVM0009280	0.52	2.68	-2.36	4.65E-03	2.03E-02	#N/A	#N/A
EVM0006570	502.56	2569.12	-2.35	5.00E-05	4.28E-04	--	Pyridoxamine 5'-phosphate oxidase
EVM0009404	15.36	78.43	-2.35	5.00E-05	4.28E-04	General function prediction only	Dip2/Utp12 Family
EVM0010867	382.18	1948.93	-2.35	5.00E-05	4.28E-04	--	--
EVM0010336	35.49	180.14	-2.34	5.00E-05	4.28E-04	--	--
EVM0003318	39.89	202.36	-2.34	5.00E-05	4.28E-04	--	Autophagy protein 16 (ATG16)
EVM0000162	303.03	1537.06	-2.34	5.00E-05	4.28E-04	--	Uncharacterized alpha/beta hydrolase domain (DUF2235)
EVM0002910	11.60	58.76	-2.34	5.00E-05	4.28E-04	--	--
EVM0002798	3.13	15.83	-2.34	5.00E-05	4.28E-04	--	F-box-like
EVM0007796	23.12	116.93	-2.34	5.00E-05	4.28E-04	--	--

EVM0007949	10.48	53.00	-2.34	5.00E-05	4.28E-04	RNA processing and modification	WD domain, G-beta repeat
EVM0005054	9.91	50.06	-2.34	5.00E-05	4.28E-04	General function prediction only	C2 domain
EVM0011731	274.29	1384.55	-2.34	5.00E-05	4.28E-04	--	Peroxidase, family 2
EVM0004617	475.63	2396.32	-2.33	5.00E-05	4.28E-04	--	ESSS subunit of NADH:ubiquinone oxidoreductase (complex I)
EVM0002660	7.30	36.65	-2.33	5.00E-05	4.28E-04	Chromatin structure and dynamics	MOZ/SAS family
EVM0001284	3.30	16.49	-2.32	5.00E-05	4.28E-04	--	Protein of unknown function (DUF3129)
EVM0005012	3.68	18.32	-2.32	5.00E-05	4.28E-04	General function prediction only	Calcineurin-like phosphoesterase
EVM0005506	4.39	21.88	-2.32	5.00E-05	4.28E-04	#N/A	#N/A
EVM0002561	3.91	19.47	-2.32	5.00E-05	4.28E-04	--	--
EVM0010344	1120.67	5556.24	-2.31	5.00E-05	4.28E-04	--	ATP synthase complex subunit h
EVM0008650	15.28	75.60	-2.31	5.00E-05	4.28E-04	--	--
EVM0003837	503.20	2484.11	-2.30	5.00E-05	4.28E-04	--	--

EVM0010786	22.46	110.84	-2.30	5.00E-05	4.28E-04	--	Zinc finger, C3HC4 type (RING finger)
EVM0011055	50.83	250.21	-2.30	5.00E-05	4.28E-04	Posttranslational modification, protein turnover, chaperones	Thioredoxin
EVM0011206	0.34	1.69	-2.29	1.70E-03	8.87E-03	--	Protein of unknown function (DUF3505)
EVM0009103	3.60	17.60	-2.29	5.00E-05	4.28E-04	--	Pheromone A receptor
EVM0006740	0.75	3.65	-2.29	2.50E-03	1.22E-02	--	--
EVM0008960	6.74	32.92	-2.29	5.00E-05	4.28E-04	--	--
EVM0011446	1369.67	6690.14	-2.29	5.00E-05	4.28E-04	--	--
EVM0007279	19.98	97.54	-2.29	5.00E-05	4.28E-04	--	--
EVM0011882	28.65	139.63	-2.29	5.00E-05	4.28E-04	Cytoskeleton	Variant SH3 domain
EVM0002073	4.45	21.67	-2.28	5.00E-05	4.28E-04	Defense mechanisms	alpha/beta hydrolase fold
EVM0007225	121.76	592.66	-2.28	5.00E-05	4.28E-04	--	Nuclear pore complex subunit Nro1
EVM0005253	213.46	1036.20	-2.28	5.00E-05	4.28E-04	--	--

EVM0006173	187.12	907.89	-2.28	5.00E-05	4.28E-04	--	Membrane-associating domain
EVM0006136	84.59	410.10	-2.28	5.00E-05	4.28E-04	--	--
EVM0001681	199.69	965.94	-2.27	5.00E-05	4.28E-04	Intracellular trafficking, secretion, and vesicular transport	Microsomal signal peptidase 12 kDa subunit (SPC12)
EVM0007771	1.29	6.20	-2.27	5.00E-05	4.28E-04	--	--
EVM0003453	69.03	332.90	-2.27	5.00E-05	4.28E-04	--	--
EVM0009773	16.51	79.53	-2.27	5.00E-05	4.28E-04	--	GPI-GlcNAc transferase complex, PIG-H component
EVM0006820	40.31	193.40	-2.26	5.00E-05	4.28E-04	--	--
EVM0010870	18.91	90.66	-2.26	5.00E-05	4.28E-04	--	Conidiation protein 6
EVM0009720	10.12	48.42	-2.26	5.00E-05	4.28E-04	Cell cycle control, cell division, chromosome partitioning	LYAR-type C2HC zinc finger
EVM0004954	40.60	194.04	-2.26	5.00E-05	4.28E-04	--	--
EVM0000357	7.37	34.97	-2.25	5.00E-05	4.28E-04	Function unknown	AhpC/TSA antioxidant enzyme
EVM0009498	0.94	4.46	-2.24	2.25E-03	1.12E-02	General function prediction only	Zinc-finger double domain

EVM0011088	20.87	98.79	-2.24	5.00E-05	4.28E-04	Translation, ribosomal structure and biogenesis	Gar1/Naf1 RNA binding region
EVM0000019	21.72	102.79	-2.24	5.00E-05	4.28E-04	--	--
EVM0001076	4.96	23.45	-2.24	5.00E-05	4.28E-04	--	--
EVM0010590	265.59	1253.85	-2.24	5.00E-05	4.28E-04	--	--
EVM0003086	73.49	346.62	-2.24	5.00E-05	4.28E-04	--	Fungal specific transcription factor domain
EVM0007572	3.48	16.41	-2.24	5.00E-05	4.28E-04	--	--
EVM0003466	14.74	69.10	-2.23	5.00E-05	4.28E-04	--	NYN domain
EVM0009876	11.28	52.85	-2.23	5.00E-05	4.28E-04	Translation, ribosomal structure and biogenesis	eRF1 domain 1
EVM0011068	76.82	359.63	-2.23	5.00E-05	4.28E-04	Defense mechanisms	Dual specificity phosphatase, catalytic domain
EVM0009051	283.50	1325.40	-2.23	5.00E-05	4.28E-04	Translation, ribosomal structure and biogenesis	Ribosomal protein L34
EVM0000147	2.92	13.65	-2.22	5.00E-05	4.28E-04	--	--
EVM0003157	291.81	1358.37	-2.22	5.00E-05	4.28E-04	Defense mechanisms	B-cell receptor-associated protein 31-like
EVM0002337	16.14	75.01	-2.22	5.00E-05	4.28E-04	--	Ribonuclease H2 non-catalytic subunit (Ylr154p-like)

EVM0001519	8.66	40.24	-2.22	5.00E-05	4.28E-04	--	--
EVM0007779	2.49	11.57	-2.21	5.00E-05	4.28E-04	--	--
EVM0008622	60.22	278.68	-2.21	5.00E-05	4.28E-04	--	50S ribosome-binding GTPase
EVM0008830	36.90	170.55	-2.21	5.00E-05	4.28E-04	Defense mechanisms	Dual specificity phosphatase, catalytic domain
EVM0000936	203.44	938.69	-2.21	5.00E-05	4.28E-04	--	--
EVM0011567	251.49	1159.67	-2.21	5.00E-05	4.28E-04	--	--
EVM0008030	0.33	1.50	-2.20	9.95E-03	3.74E-02	Transcription	Homeobox domain
EVM0006126	21.17	97.29	-2.20	5.00E-05	4.28E-04	--	--
EVM0003548	6.31	28.99	-2.20	5.00E-05	4.28E-04	Signal transduction mechanisms	--
EVM0011359	125.91	578.41	-2.20	5.00E-05	4.28E-04	Energy production and conversion	ETC complex I subunit conserved region
EVM0005543	3.39	15.55	-2.20	5.00E-05	4.28E-04	--	Glycosyltransferase family 18
EVM0000720	32.06	146.85	-2.20	1.05E-03	5.94E-03	--	Carboxyl transferase domain
EVM0003103	52.01	237.94	-2.19	5.00E-05	4.28E-04	--	--

EVM0011107	91.06	416.56	-2.19	5.00E-05	4.28E-04	--	--
EVM0010348	17.53	80.03	-2.19	5.00E-05	4.28E-04	--	--
EVM0009583	24.66	112.47	-2.19	5.00E-05	4.28E-04	--	EF hand
EVM0008872	1.63	7.39	-2.18	5.00E-04	3.19E-03	Lipid transport and metabolism	Fatty acid hydroxylase superfamily
EVM0011757	1.95	8.88	-2.18	2.50E-04	1.77E-03	--	--
EVM0003549	1.73	7.84	-2.18	1.55E-03	8.22E-03	--	--
EVM0010029	141.73	640.64	-2.18	5.00E-05	4.28E-04	--	Microsomal signal peptidase 25 kDa subunit (SPC25)
EVM0006020	62.17	280.92	-2.18	5.00E-05	4.28E-04	--	--
EVM0004708	119.43	538.56	-2.17	5.00E-05	4.28E-04	--	SecY translocase
EVM0007196	182.61	823.45	-2.17	5.00E-05	4.28E-04	RNA processing and modification	LSM domain
EVM0004959	114.88	517.55	-2.17	5.00E-05	4.28E-04	General function prediction only	PQ loop repeat
EVM0002640	37.02	166.27	-2.17	5.00E-05	4.28E-04	Chromatin structure and dynamics	SET domain
EVM0009206	42.48	190.71	-2.17	5.00E-05	4.28E-04	--	Protein of unknown function (DUF3129)

EVM0001197	18.22	81.77	-2.17	5.00E-05	4.28E-04	--	--
EVM0000572	13.31	59.69	-2.17	5.00E-05	4.28E-04	--	Nucleolar complex-associated protein
EVM0005244	93.50	419.30	-2.17	5.00E-05	4.28E-04	Function unknown	Polysaccharide biosynthesis
EVM0009884	438.62	1965.97	-2.16	5.00E-05	4.28E-04	--	Glycosyl hydrolase catalytic core
EVM0004319	26.02	116.50	-2.16	5.00E-05	4.28E-04	--	Protein of unknown function (DUF2962)
EVM0011612	35.43	158.51	-2.16	5.00E-05	4.28E-04	--	--
EVM0008791	6.50	29.07	-2.16	5.00E-05	4.28E-04	Coenzyme transport and metabolism	Dephospho-CoA kinase
EVM0005157	21.63	96.71	-2.16	5.00E-05	4.28E-04	Secondary metabolites biosynthesis, transport and catabolism	short chain dehydrogenase
EVM0007662	855.61	3824.11	-2.16	5.00E-05	4.28E-04	--	--
EVM0002729	62.99	281.26	-2.16	5.00E-05	4.28E-04	--	Ergosterol biosynthesis ERG4/ERG24 family
EVM0010583	268.20	1196.77	-2.16	5.00E-05	4.28E-04	--	--
EVM0002402	0.86	3.84	-2.16	1.95E-03	9.93E-03	--	Domain of unknown function (DUF3328)

EVM0009547	43.63	194.21	-2.15	5.00E-05	4.28E-04	--	Cytochrome P450
EVM0008851	205.74	913.84	-2.15	5.00E-05	4.28E-04	Intracellular trafficking, secretion, and vesicular transport	Preprotein translocase subunit Sec66
EVM0000932	56.84	252.19	-2.15	5.00E-05	4.28E-04	--	--
EVM0005108	2.67	11.82	-2.15	5.00E-05	4.28E-04	--	GCC2 and GCC3
EVM0008511	24.25	107.30	-2.15	5.00E-05	4.28E-04	RNA processing and modification	Ribosomal RNA-processing protein 7 (RRP7)
EVM0002331	73.10	322.40	-2.14	5.00E-05	4.28E-04	Posttranslational modification, protein turnover, chaperones	14-3-3 protein
EVM0002338	10.13	44.68	-2.14	5.00E-05	4.28E-04	Lipid transport and metabolism	PAP2 superfamily
EVM0006021	1.45	6.38	-2.14	6.15E-03	2.54E-02	--	--
EVM0011879	42.12	185.59	-2.14	5.00E-05	4.28E-04	General function prediction only	--
EVM0006561	4.00	17.61	-2.14	1.50E-04	1.14E-03	--	--
EVM0008761	13.60	59.88	-2.14	5.00E-05	4.28E-04	--	Calcineurin-like phosphoesterase
EVM0001700	52.76	232.20	-2.14	5.00E-05	4.28E-04	--	Cobalamin-independent synthase, Catalytic domain

EVM0010983	2.80	12.29	-2.14	5.00E-05	4.28E-04	--	--
EVM0007254	7240.86	31828.00	-2.14	5.00E-05	4.28E-04	--	Ribosomal protein S30
EVM0002292	45.23	197.87	-2.13	5.00E-05	4.28E-04	--	--
EVM0009353	60.32	263.27	-2.13	5.00E-05	4.28E-04	--	--
EVM0004297	24.02	104.81	-2.13	5.00E-05	4.28E-04	--	BTG family
EVM0003876	2.13	9.29	-2.13	5.00E-05	4.28E-04	--	Hemerythrin HHE cation binding domain
EVM0009908	220.37	960.96	-2.12	5.00E-05	4.28E-04	--	--
EVM0002491	286.10	1247.39	-2.12	5.00E-05	4.28E-04	--	YABBY protein
EVM0008587	15.14	65.97	-2.12	5.00E-05	4.28E-04	--	--
EVM0007388	14.44	62.90	-2.12	5.00E-05	4.28E-04	--	--
EVM0012268	49.08	213.17	-2.12	5.00E-05	4.28E-04	Function unknown	Pam16
EVM0000363	10.99	47.64	-2.12	5.00E-05	4.28E-04	Posttranslational modification, protein turnover, chaperones	Zinc knuckle

EVM0011090	331.40	1436.10	-2.12	5.00E-05	4.28E-04	Intracellular trafficking, secretion, and vesicular transport	Tim10/DDP family zinc finger
EVM0011982	18.44	79.87	-2.12	5.00E-05	4.28E-04	Chromatin structure and dynamics	Core histone H2A/H2B/H3/H4
EVM0007621	37.23	160.70	-2.11	5.00E-05	4.28E-04	Replication, recombination and repair	NUDIX domain
EVM0004736	0.47	2.02	-2.11	9.75E-03	3.67E-02	--	--
EVM0009653	21.53	92.73	-2.11	5.00E-05	4.28E-04	--	Cellulase (glycosyl hydrolase family 5)
EVM0008356	9.76	42.01	-2.11	5.00E-05	4.28E-04	Function unknown	Utp11 protein
EVM0007939	2.44	10.51	-2.10	3.50E-04	2.36E-03	General function prediction only	Ras family
EVM0002967	3.65	15.64	-2.10	4.85E-03	2.10E-02	--	--
EVM0000467	4.37	18.75	-2.10	5.00E-05	4.28E-04	--	--
EVM0007770	3.48	14.91	-2.10	9.00E-04	5.22E-03	--	--
EVM0010617	126.98	544.50	-2.10	5.00E-05	4.28E-04	--	DASH complex subunit Dad3
EVM0010158	53.16	227.91	-2.10	5.00E-05	4.28E-04	Function unknown	Pre-rRNA-processing protein TSR2

EVM0005022	20.13	86.26	-2.10	5.00E-05	4.28E-04	Function unknown	Protein of unknown function (DUF788)
EVM0009205	1.46	6.26	-2.10	1.90E-03	9.73E-03	--	--
EVM0006099	92.45	395.05	-2.10	5.00E-05	4.28E-04	Lipid transport and metabolism	Enoyl-CoA hydratase/isomerase family
EVM0002811	243.51	1037.48	-2.09	5.00E-05	4.28E-04	Posttranslational modification, protein turnover, chaperones	Thioredoxin
EVM0008173	1.48	6.30	-2.09	5.00E-05	4.28E-04	--	--
EVM0004762	115.33	491.14	-2.09	5.00E-05	4.28E-04	Intracellular trafficking, secretion, and vesicular transport	Clathrin adaptor complex small chain
EVM0005049	34.18	145.47	-2.09	5.00E-05	4.28E-04	Posttranslational modification, protein turnover, chaperones	DnaJ domain
EVM0003625	77.04	327.28	-2.09	5.00E-05	4.28E-04	Carbohydrate transport and metabolism	Alpha amylase, catalytic domain
EVM0010897	31.53	133.93	-2.09	5.00E-05	4.28E-04	--	DASH complex subunit Dad2
EVM0005369	156.14	662.51	-2.09	5.00E-05	4.28E-04	Intracellular trafficking, secretion, and vesicular transport	emp24/gp25L/p24 family/GOLD
EVM0008562	205.37	869.38	-2.08	5.00E-05	4.28E-04	--	--

EVM0005697	26.01	109.83	-2.08	5.00E-05	4.28E-04	--	Peroxin-3
EVM0004410	16.72	70.58	-2.08	5.00E-05	4.28E-04	--	PAPA-1-like conserved region
EVM0010921	11.95	50.39	-2.08	5.00E-05	4.28E-04	--	--
EVM0011153	135.74	570.89	-2.07	5.00E-05	4.28E-04	--	Tim17/Tim22/Tim23/Pmp24 family
EVM0000706	16.56	69.61	-2.07	5.00E-05	4.28E-04	--	Alginate lyase
EVM0001259	8.53	35.87	-2.07	5.00E-05	4.28E-04	General function prediction only	WD domain, G-beta repeat
EVM0011746	7.92	33.23	-2.07	5.00E-05	4.28E-04	--	--
EVM0008485	5.10	21.30	-2.06	5.00E-05	4.28E-04	--	--
EVM0005110	29.61	123.58	-2.06	5.00E-05	4.28E-04	Transcription	--
EVM0010702	12.09	50.43	-2.06	5.00E-05	4.28E-04	--	--
EVM0011577	69.27	288.87	-2.06	5.00E-05	4.28E-04	--	ER membrane protein SH3
EVM0008320	2.30	9.58	-2.06	5.00E-05	4.28E-04	--	Nuclear condensing complex subunits, C-term domain
EVM0002743	9.03	37.61	-2.06	5.00E-05	4.28E-04	--	--

EVM0008058	9.76	40.64	-2.06	5.00E-05	4.28E-04	--	--
EVM0008835	32.71	136.13	-2.06	5.00E-05	4.28E-04	Carbohydrate transport and metabolism	--
EVM0008406	47.92	199.36	-2.06	5.00E-05	4.28E-04	Lipid transport and metabolism	AMP-binding enzyme
EVM0001342	26.47	110.10	-2.06	5.00E-05	4.28E-04	Defense mechanisms	TB2/DP1, HVA22 family
EVM0009527	5.81	24.15	-2.05	5.00E-05	4.28E-04	Replication, recombination and repair	Cid1 family poly A polymerase
EVM0011085	23.83	98.97	-2.05	5.00E-05	4.28E-04	--	Translation initiation factor IF-3, C-terminal domain
EVM0006734	179.11	743.70	-2.05	5.00E-05	4.28E-04	--	Complex 1 protein (LYR family)
EVM0002517	138.70	575.78	-2.05	5.00E-05	4.28E-04	Defense mechanisms	Tetratricopeptide repeat
EVM0003684	2.37	9.85	-2.05	5.00E-05	4.28E-04	#N/A	#N/A
EVM0011239	74.61	309.36	-2.05	5.00E-05	4.28E-04	--	50S ribosome-binding GTPase
EVM0002230	13.58	56.14	-2.05	5.00E-05	4.28E-04	--	Lactonase, 7-bladed beta-propeller
EVM0004837	109.85	454.07	-2.05	5.00E-05	4.28E-04	General function prediction only	Ras family
EVM0009586	39.13	161.55	-2.05	5.00E-05	4.28E-04	Lipid transport and metabolism	Fatty acid hydroxylase superfamily

EVM0002154	0.84	3.47	-2.04	8.00E-04	4.73E-03	--	--
EVM0011632	36.71	150.92	-2.04	5.00E-05	4.28E-04	--	--
EVM0008343	7.63	31.31	-2.04	5.00E-05	4.28E-04	--	--
EVM0009995	6.51	26.74	-2.04	5.00E-05	4.28E-04	RNA processing and modification	WD domain, G-beta repeat
EVM0007132	0.99	4.05	-2.04	1.00E-04	7.96E-04	--	--
EVM0002285	1.83	7.52	-2.04	5.00E-05	4.28E-04	--	--
EVM0005544	64.15	262.82	-2.03	5.00E-05	4.28E-04	Translation, ribosomal structure and biogenesis	Ribosomal protein L18e/L15
EVM0001016	5.53	22.66	-2.03	5.00E-05	4.28E-04	--	--
EVM0004688	18.59	75.99	-2.03	5.00E-05	4.28E-04	--	--
EVM0011165	21.32	87.15	-2.03	5.00E-05	4.28E-04	Energy production and conversion	Mitochondrial carrier protein
EVM0005264	38.02	155.28	-2.03	5.00E-05	4.28E-04	General function prediction only	Major Facilitator Superfamily
EVM0000773	359.29	1466.01	-2.03	5.00E-05	4.28E-04	Energy production and conversion	NADH-ubiquinone oxidoreductase B18 subunit (NDUFB7)

EVM0002656	73.68	300.17	-2.03	5.00E-05	4.28E-04	Function unknown	Erg28 like protein
EVM0002198	4.40	17.91	-2.03	5.00E-05	4.28E-04	--	Phosphotransferase enzyme family
EVM0003931	96.54	392.37	-2.02	5.00E-05	4.28E-04	--	--
EVM0011838	201.68	819.46	-2.02	5.00E-05	4.28E-04	--	Ribosomal protein S8
EVM0002024	6.25	25.39	-2.02	5.00E-05	4.28E-04	Secondary metabolites biosynthesis, transport and catabolism	short chain dehydrogenase
EVM0002372	12.43	50.32	-2.02	5.00E-05	4.28E-04	--	Cytochrome P450
EVM0000472	1992.42	8035.73	-2.01	5.00E-05	4.28E-04	--	--
EVM0000076	0.47	1.88	-2.01	7.90E-03	3.10E-02	--	Zinc-binding dehydrogenase
EVM0005931	1.65	6.63	-2.01	7.00E-04	4.23E-03	--	--
EVM0011475	5.42	21.76	-2.01	5.00E-05	4.28E-04	--	--
EVM0010715	38.90	155.98	-2.00	5.00E-05	4.28E-04	RNA processing and modification	Isy1-like splicing family
EVM0003567	14.09	56.35	-2.00	5.00E-05	4.28E-04	--	Tryptophan halogenase

EVM0010407	7.65	30.57	-2.00	5.00E-05	4.28E-04	--	Putative S-adenosyl-L-methionine-dependent methyltransferase
EVM0001591	1.57	6.29	-2.00	5.00E-05	4.28E-04	--	--
EVM0005185	78.03	311.32	-2.00	5.00E-05	4.28E-04	Amino acid transport and metabolism	Aminotransferase class-V
EVM0000905	23.39	93.07	-1.99	5.00E-05	4.28E-04	--	--
EVM0011144	46.12	183.45	-1.99	5.00E-05	4.28E-04	--	Protein of unknown function (DUF2034)
EVM0006060	4.96	19.72	-1.99	5.00E-05	4.28E-04	--	PP-loop family
EVM0007905	13.87	55.09	-1.99	5.00E-05	4.28E-04	--	Spc19
EVM0006949	69.91	277.54	-1.99	5.00E-05	4.28E-04	--	N-terminal domain of ribose phosphate pyrophosphokinase
EVM0001268	1.71	6.80	-1.99	5.00E-05	4.28E-04	--	--
EVM0002754	17.95	71.17	-1.99	5.00E-05	4.28E-04	General function prediction only	Ankyrin repeats (3 copies)
EVM0008871	64.60	256.09	-1.99	5.00E-05	4.28E-04	General function prediction only	Ras family
EVM0002628	225.30	892.85	-1.99	5.00E-05	4.28E-04	Translation, ribosomal structure and biogenesis	Mitochondrial ribosomal subunit S27

EVM0008497	460.74	1821.91	-1.98	5.00E-05	4.28E-04	Lipid transport and metabolism	MaoC like domain
EVM0006547	82.72	326.88	-1.98	5.00E-05	4.28E-04	Translation, ribosomal structure and biogenesis	Ribosomal protein L23
EVM0004398	260.23	1027.78	-1.98	5.00E-05	4.28E-04	General function prediction only	Proteolipid membrane potential modulator
EVM0003817	4.77	18.81	-1.98	5.00E-05	4.28E-04	--	--
EVM0005078	2.05	8.10	-1.98	4.50E-04	2.92E-03	Cell cycle control, cell division, chromosome partitioning	HORMA domain
EVM0000419	44.43	175.18	-1.98	5.00E-05	4.28E-04	Function unknown	Complex 1 protein (LYR family)
EVM0005619	10.10	39.74	-1.98	5.00E-05	4.28E-04	--	60Kd inner membrane protein
EVM0003822	6.03	23.71	-1.98	5.00E-05	4.28E-04	Translation, ribosomal structure and biogenesis	Formyl transferase
EVM0005613	19.51	76.71	-1.98	5.00E-05	4.28E-04	--	--
EVM0002758	1.39	5.44	-1.97	4.00E-03	1.79E-02	--	--
EVM0004710	129.07	506.83	-1.97	5.00E-05	4.28E-04	--	Cgr1 family
EVM0008258	42.75	167.71	-1.97	5.00E-05	4.28E-04	--	--

EVM0010354	0.78	3.05	-1.97	7.50E-04	4.49E-03	--	F-box-like
EVM0010012	11.00	42.92	-1.96	5.00E-05	4.28E-04	--	Formin Homology Region 1
EVM0006285	67.10	261.71	-1.96	5.00E-05	4.28E-04	Lipid transport and metabolism	Emopamil binding protein
EVM0006529	16.45	64.07	-1.96	5.00E-05	4.28E-04	--	--
EVM0012211	19.84	77.25	-1.96	5.00E-05	4.28E-04	--	--
EVM0001883	9.81	38.20	-1.96	5.00E-05	4.28E-04	--	LIM domain
EVM0000204	629.60	2450.84	-1.96	5.00E-05	4.28E-04	--	short chain dehydrogenase
EVM0007556	190.49	740.02	-1.96	5.00E-05	4.28E-04	--	Ser-Thr-rich glycosyl-phosphatidyl-inositol-anchored membrane family
EVM0006296	4.04	15.69	-1.96	5.00E-05	4.28E-04	--	--
EVM0009852	8.91	34.59	-1.96	5.00E-05	4.28E-04	Transcription	Homeobox domain
EVM0001421	50.04	193.70	-1.95	5.00E-05	4.28E-04	Cell cycle control, cell division, chromosome partitioning	Protein kinase domain
EVM0001194	7.35	28.44	-1.95	5.00E-05	4.28E-04	--	--

EVM0001584	34.37	132.95	-1.95	5.00E-05	4.28E-04	--	Voltage-dependent anion channel
EVM0009036	127.04	490.95	-1.95	5.00E-05	4.28E-04	RNA processing and modification	Ribosomal protein L7Ae/L30e/S12e/Gadd45 family
EVM0009625	10.47	40.43	-1.95	5.00E-05	4.28E-04	--	AIG1 family
EVM0011691	12.58	48.57	-1.95	5.00E-05	4.28E-04	Function unknown	Regulator of chromosome condensation (RCC1) repeat
EVM0006200	4.31	16.63	-1.95	5.00E-05	4.28E-04	Posttranslational modification, protein turnover, chaperones	Eukaryotic aspartyl protease
EVM0002948	30.61	118.09	-1.95	5.00E-05	4.28E-04	--	Protein of unknown function (Ytp1)
EVM0010252	40.89	157.55	-1.95	5.00E-05	4.28E-04	--	PX domain
EVM0001566	2.00	7.70	-1.95	5.00E-05	4.28E-04	General function prediction only	Sugar (and other) transporter
EVM0006292	2.78	10.69	-1.94	5.00E-05	4.28E-04	--	Fungal Zn(2)-Cys(6) binuclear cluster domain
EVM0003828	14.93	57.41	-1.94	5.00E-05	4.28E-04	--	Membrane bound O-acyl transferase family
EVM0008088	7.71	29.64	-1.94	5.00E-05	4.28E-04	--	--
EVM0001511	19.95	76.55	-1.94	5.00E-05	4.28E-04	Translation, ribosomal structure and biogenesis	GTP-binding protein TrmE N-terminus

EVM0000442	53.85	206.56	-1.94	5.00E-05	4.28E-04	--	--
EVM0010303	2.33	8.95	-1.94	5.00E-05	4.28E-04	--	Helix-turn-helix domain
EVM0002264	77.82	297.99	-1.94	5.00E-05	4.28E-04	--	--
EVM0003886	174.99	668.14	-1.93	5.00E-05	4.28E-04	--	Ribosomal prokaryotic L21 protein
EVM0005939	6.87	26.21	-1.93	5.00E-05	4.28E-04	--	--
EVM0010495	32.20	122.87	-1.93	5.00E-05	4.28E-04	--	Protein kinase domain
EVM0000836	109.52	417.02	-1.93	5.00E-05	4.28E-04	Intracellular trafficking, secretion, and vesicular transport	WD domain, G-beta repeat
EVM0001382	10.07	38.33	-1.93	5.00E-05	4.28E-04	--	--
EVM0005032	30.02	114.27	-1.93	5.00E-05	4.28E-04	Signal transduction mechanisms	NUDIX domain
EVM0007173	70.31	267.38	-1.93	5.00E-05	4.28E-04	Replication, recombination and repair	Nucleosome assembly protein (NAP)
EVM0003322	5.71	21.72	-1.93	5.00E-05	4.28E-04	General function prediction only	Sugar (and other) transporter
EVM0010446	36.52	138.75	-1.93	5.00E-05	4.28E-04	Transcription	Homeobox KN domain

EVM0009699	59.94	227.55	-1.92	5.00E-05	4.28E-04	Posttranslational modification, protein turnover, chaperones	Proteasome subunit
EVM0002423	48.62	184.06	-1.92	4.40E-03	1.94E-02	Energy production and conversion	Carbamoyl-phosphate synthase L chain, ATP binding domain
EVM0004635	101.07	382.58	-1.92	5.00E-05	4.28E-04	--	--
EVM0011804	5.19	19.64	-1.92	5.00E-05	4.28E-04	--	--
EVM0000031	299.51	1131.39	-1.92	5.00E-05	4.28E-04	--	--
EVM0011230	17.92	67.68	-1.92	6.10E-03	2.52E-02	--	SNARE associated Golgi protein
EVM0011786	22.04	83.21	-1.92	5.00E-05	4.28E-04	--	--
EVM0011619	21.36	80.50	-1.91	5.00E-05	4.28E-04	--	F-box-like
EVM0005809	11.30	42.50	-1.91	5.00E-05	4.28E-04	Replication, recombination and repair	RNase H
EVM0008383	403.50	1513.02	-1.91	5.00E-05	4.28E-04	Energy production and conversion	NADH ubiquinone oxidoreductase subunit NDUFA12
EVM0004134	46.71	174.72	-1.90	5.00E-05	4.28E-04	General function prediction only	RNA recognition motif. (a.k.a. RRM, RBD, or RNP domain)
EVM0004256	5.68	21.23	-1.90	3.50E-04	2.36E-03	--	--

EVM0005705	11.28	42.10	-1.90	5.00E-05	4.28E-04	--	--
EVM0010165	53.31	198.82	-1.90	5.00E-05	4.28E-04	Function unknown	AhpC/TSA antioxidant enzyme
EVM0000794	532.17	1984.75	-1.90	5.00E-05	4.28E-04	Posttranslational modification, protein turnover, chaperones	Redoxin
EVM0010743	4.31	16.08	-1.90	5.00E-05	4.28E-04	--	F-box-like
EVM0000029	11.07	41.25	-1.90	5.00E-05	4.28E-04	Transcription	RNA polymerase Rpb3/RpoA insert domain
EVM0009698	1049.89	3909.93	-1.90	5.00E-05	4.28E-04	Translation, ribosomal structure and biogenesis	Ribosomal L38e protein family
EVM0008889	1.35	5.04	-1.90	3.50E-04	2.36E-03	--	Cytochrome P450
EVM0003580	5.37	19.98	-1.89	5.00E-05	4.28E-04	--	--
EVM0001247	25.31	93.81	-1.89	5.00E-05	4.28E-04	--	MAC/Perforin domain
EVM0008461	12.87	47.64	-1.89	5.00E-05	4.28E-04	--	Putative snoRNA binding domain
EVM0001277	5.83	21.51	-1.88	5.00E-05	4.28E-04	--	--
EVM0005073	11.11	40.97	-1.88	5.00E-05	4.28E-04	--	Nitronate monooxygenase

EVM0002382	1.17	4.30	-1.88	1.01E-02	3.78E-02	--	--
EVM0005402	2.38	8.72	-1.88	5.00E-05	4.28E-04	--	F-box domain
EVM0006468	13.60	49.85	-1.87	5.00E-05	4.28E-04	Signal transduction mechanisms	RED-like protein N-terminal region
EVM0000560	3.71	13.59	-1.87	5.00E-05	4.28E-04	--	Ferric reductase like transmembrane component
EVM0006499	6.16	22.58	-1.87	5.00E-05	4.28E-04	--	--
EVM0008399	0.33	1.20	-1.87	8.05E-03	3.15E-02	--	--
EVM0001058	6.07	22.17	-1.87	5.00E-05	4.28E-04	--	--
EVM0006282	2.91	10.63	-1.87	5.00E-05	4.28E-04	--	--
EVM0006795	461.49	1683.99	-1.87	5.00E-05	4.28E-04	--	--
EVM0007872	63.49	231.38	-1.87	5.00E-05	4.28E-04	--	--
EVM0003954	34.43	125.45	-1.87	5.00E-05	4.28E-04	Intracellular trafficking, secretion, and vesicular transport	--
EVM0008504	58.39	212.76	-1.87	5.00E-05	4.28E-04	--	F-box-like

EVM0007568	104.78	381.71	-1.87	5.00E-05	4.28E-04	--	ACT domain
EVM0006498	1.24	4.53	-1.86	3.15E-03	1.47E-02	--	--
EVM0007304	29.89	108.80	-1.86	5.00E-05	4.28E-04	Carbohydrate transport and metabolism	UAA transporter family
EVM0004063	43.87	159.60	-1.86	5.00E-05	4.28E-04	Transcription	RNA recognition motif. (a.k.a. RRM, RBD, or RNP domain)
EVM0008519	122.32	444.91	-1.86	5.00E-05	4.28E-04	RNA processing and modification	LUC7 N_terminus
EVM0000596	3.89	14.13	-1.86	5.00E-05	4.28E-04	Replication, recombination and repair	--
EVM0008224	138.01	501.58	-1.86	5.00E-05	4.28E-04	Amino acid transport and metabolism	Protein of unknown function (DUF619)
EVM0003106	18.49	67.20	-1.86	5.00E-05	4.28E-04	--	Spt20 family
EVM0001816	8.27	29.99	-1.86	5.00E-05	4.28E-04	Posttranslational modification, protein turnover, chaperones	Cyclophilin type peptidyl-prolyl cis-trans isomerase/CLD
EVM0009709	32.49	117.72	-1.86	5.00E-05	4.28E-04	--	--
EVM0011276	65.66	237.87	-1.86	5.00E-05	4.28E-04	Translation, ribosomal structure and biogenesis	Pumilio-family RNA binding repeat
EVM0004707	38.03	137.60	-1.86	5.00E-05	4.28E-04	--	Alpha-L-rhamnosidase N-terminal domain

EVM0009463	140.06	506.58	-1.85	5.00E-05	4.28E-04	--	Ribosomal protein L35
EVM0011238	27.17	98.25	-1.85	5.00E-05	4.28E-04	Secondary metabolites biosynthesis, transport and catabolism	Cytochrome P450
EVM0006335	116.74	422.08	-1.85	5.00E-05	4.28E-04	--	--
EVM0011646	13.15	47.41	-1.85	5.00E-05	4.28E-04	--	Pheromone A receptor
EVM0004053	185.07	667.10	-1.85	5.00E-05	4.28E-04	--	--
EVM0007826	19.64	70.79	-1.85	5.00E-05	4.28E-04	General function prediction only	RNA recognition motif. (a.k.a. RRM, RBD, or RNP domain)
EVM0007539	235.10	847.27	-1.85	5.00E-05	4.28E-04	--	SUR7/PalI family
EVM0004055	58.80	211.49	-1.85	5.00E-05	4.28E-04	Translation, ribosomal structure and biogenesis	Ribosomal protein S13/S18
EVM0001657	112.63	405.01	-1.85	5.00E-05	4.28E-04	--	--
EVM0004716	11.90	42.79	-1.85	5.00E-05	4.28E-04	Replication, recombination and repair	Exonuclease
EVM0005431	7.22	25.93	-1.85	5.00E-05	4.28E-04	Cell cycle control, cell division, chromosome partitioning	HEC/Ndc80p family
EVM0000310	3.59	12.89	-1.85	4.50E-04	2.92E-03	--	--

EVM0002996	17.46	62.69	-1.84	5.00E-05	4.28E-04	Transcription	Sin3 associated polypeptide p18 (SAP18)
EVM0008789	1753.47	6293.03	-1.84	5.00E-05	4.28E-04	Energy production and conversion	Cytochrome c oxidase subunit VIa
EVM0000547	3.31	11.86	-1.84	5.00E-05	4.28E-04	--	--
EVM0003364	4.03	14.46	-1.84	5.00E-05	4.28E-04	--	--
EVM0005267	23.65	84.75	-1.84	4.90E-03	2.12E-02	--	--
EVM0002076	300.25	1075.35	-1.84	5.00E-05	4.28E-04	--	CFEM domain
EVM0009466	4.12	14.74	-1.84	5.00E-05	4.28E-04	--	UreF
EVM0005232	46.86	167.66	-1.84	5.00E-05	4.28E-04	RNA processing and modification	Brix domain
EVM0009065	6.83	24.43	-1.84	5.00E-05	4.28E-04	--	--
EVM0006231	99.80	356.39	-1.84	5.00E-05	4.28E-04	--	--
EVM0000755	118.50	422.69	-1.83	5.00E-05	4.28E-04	--	--
EVM0007113	2.76	9.82	-1.83	9.35E-03	3.55E-02	--	--
EVM0007046	4.31	15.36	-1.83	5.00E-05	4.28E-04	--	--

EVM0008232	1.58	5.64	-1.83	1.00E-04	7.96E-04	--	Glycosyl hydrolases family 28
EVM0003029	30.01	106.91	-1.83	5.00E-05	4.28E-04	Signal transduction mechanisms	Protein-tyrosine phosphatase
EVM0005991	184.07	654.91	-1.83	5.00E-05	4.28E-04	--	SAP domain
EVM0005206	5.52	19.62	-1.83	5.00E-05	4.28E-04	--	--
EVM0009840	11.08	39.39	-1.83	5.00E-05	4.28E-04	--	BUD22
EVM0004042	54.48	193.43	-1.83	5.00E-05	4.28E-04	--	--
EVM0005926	67.34	239.08	-1.83	5.00E-05	4.28E-04	General function prediction only	Peptidase family M48
EVM0010482	226.69	804.18	-1.83	5.00E-05	4.28E-04	--	--
EVM0008781	59.91	212.40	-1.83	5.00E-05	4.28E-04	--	--
EVM0012038	82.50	292.47	-1.83	5.00E-05	4.28E-04	--	3-beta hydroxysteroid dehydrogenase/isomerase family
EVM0002995	8.67	30.74	-1.83	5.00E-05	4.28E-04	RNA processing and modification	FF domain
EVM0002465	4.08	14.46	-1.83	2.50E-04	1.77E-03	--	50S ribosome-binding GTPase
EVM0004919	5.95	21.06	-1.82	5.00E-05	4.28E-04	Cytoskeleton	Tetratricopeptide repeat

EVM0005906	0.63	2.22	-1.82	2.05E-03	1.03E-02	--	Glycosyl hydrolase family 7
EVM0005644	33.24	117.36	-1.82	5.00E-05	4.28E-04	Lipid transport and metabolism	Phospholipid methyltransferase
EVM0010966	6.07	21.43	-1.82	5.00E-05	4.28E-04	Replication, recombination and repair	ATPase family associated with various cellular activities (AAA)
EVM0001716	1.90	6.70	-1.82	5.00E-05	4.28E-04	--	Pregnancy-associated plasma protein-A
EVM0001438	1.12	3.96	-1.82	3.50E-04	2.36E-03	--	--
EVM0008986	82.45	290.49	-1.82	5.00E-05	4.28E-04	Intracellular trafficking, secretion, and vesicular transport	Endoplasmic reticulum vesicle transporter
EVM0008169	526.70	1855.38	-1.82	5.00E-05	4.28E-04	--	Cornichon protein
EVM0005796	1292.18	4545.87	-1.81	5.00E-05	4.28E-04	Translation, ribosomal structure and biogenesis	60s Acidic ribosomal protein
EVM0003262	24.81	87.23	-1.81	5.00E-05	4.28E-04	--	Ribonuclease-III-like
EVM0011344	9.92	34.83	-1.81	5.00E-05	4.28E-04	RNA processing and modification	Mpp10 protein
EVM0004028	64.29	225.73	-1.81	5.00E-05	4.28E-04	Cell cycle control, cell division, chromosome partitioning	--
EVM0002694	3.97	13.92	-1.81	5.00E-05	4.28E-04	--	DNA polymerase subunit Cdc27

EVM0002056	120.50	422.21	-1.81	5.00E-05	4.28E-04	--	--
EVM0006056	1.03	3.62	-1.81	2.25E-03	1.12E-02	--	--
EVM0009797	3.98	13.95	-1.81	5.00E-05	4.28E-04	--	Pheromone A receptor
EVM0006681	75.34	263.64	-1.81	5.00E-05	4.28E-04	--	--
EVM0002551	15.15	53.00	-1.81	5.00E-05	4.28E-04	Translation, ribosomal structure and biogenesis	Nop14-like family
EVM0006915	21.29	74.44	-1.81	5.00E-05	4.28E-04	--	Velvet factor
EVM0003543	58.24	203.55	-1.81	5.00E-05	4.28E-04	--	--
EVM0000373	2.09	7.31	-1.80	2.50E-04	1.77E-03	--	Terpene synthase family, metal binding domain
EVM0006805	107.23	374.56	-1.80	5.00E-05	4.28E-04	Lipid transport and metabolism	Alpha/beta hydrolase family
EVM0010513	5.00	17.47	-1.80	5.00E-05	4.28E-04	General function prediction only	Fumarylacetoacetate (FAA) hydrolase family
EVM0003306	48.58	169.53	-1.80	5.00E-05	4.28E-04	Intracellular trafficking, secretion, and vesicular transport	Snf7
EVM0005649	123.43	430.64	-1.80	5.00E-05	4.28E-04	Transcription	Basic region leucine zipper

EVM0001778	110.15	383.48	-1.80	5.00E-05	4.28E-04	Coenzyme transport and metabolism	Pyridoxamine 5'-phosphate oxidase
EVM0008213	798.91	2780.10	-1.80	5.00E-05	4.28E-04	RNA processing and modification	LSM domain
EVM0003608	20.67	71.91	-1.80	5.00E-05	4.28E-04	--	--
EVM0007386	6.61	23.00	-1.80	5.00E-05	4.28E-04	--	--
EVM0006092	107.53	372.98	-1.79	5.00E-05	4.28E-04	--	Complex1_LYR-like
EVM0005696	1.27	4.39	-1.79	1.70E-03	8.87E-03	--	--
EVM0007682	38.28	132.45	-1.79	1.50E-04	1.14E-03	--	--
EVM0010005	18.40	63.67	-1.79	5.00E-05	4.28E-04	--	PIG-X / PBN1
EVM0004809	39.76	137.53	-1.79	5.00E-05	4.28E-04	Posttranslational modification, protein turnover, chaperones	Myb-like DNA-binding domain
EVM0009109	30.09	104.04	-1.79	5.00E-05	4.28E-04	--	Glycosyl hydrolases family 43
EVM0004647	2.20	7.59	-1.79	5.00E-04	3.19E-03	--	--
EVM0011608	717.36	2478.86	-1.79	5.00E-05	4.28E-04	Translation, ribosomal structure and biogenesis	Ribosomal protein L24e

EVM0006565	142.82	493.51	-1.79	5.00E-05	4.28E-04	Lipid transport and metabolism	Enoyl-CoA hydratase/isomerase family
EVM0002572	56.62	195.39	-1.79	5.00E-05	4.28E-04	Transcription	Transcriptional Coactivator p15 (PC4)
EVM0001039	22.13	76.35	-1.79	8.50E-04	4.98E-03	--	--
EVM0010070	119.60	412.28	-1.79	5.00E-05	4.28E-04	--	--
EVM0003725	46.71	160.99	-1.79	5.00E-05	4.28E-04	--	--
EVM0003892	19.57	67.38	-1.78	5.00E-05	4.28E-04	--	--
EVM0000705	450.72	1551.46	-1.78	5.00E-05	4.28E-04	Inorganic ion transport and metabolism	Iron/manganese superoxide dismutases, C-terminal domain
EVM0005583	16.56	56.97	-1.78	5.00E-05	4.28E-04	--	--
EVM0010427	14.67	50.46	-1.78	5.00E-05	4.28E-04	--	--
EVM0008404	11.35	39.03	-1.78	5.00E-05	4.28E-04	RNA processing and modification	BING4CT (NUC141) domain
EVM0009718	3.24	11.15	-1.78	5.00E-05	4.28E-04	--	--
EVM0012166	7.91	27.18	-1.78	5.00E-05	4.28E-04	--	Glyoxal oxidase N-terminus

EVM0009437	50.74	174.36	-1.78	5.00E-05	4.28E-04	Posttranslational modification, protein turnover, chaperones	Hsp90 protein
EVM0006678	26.13	89.80	-1.78	5.00E-05	4.28E-04	Intracellular trafficking, secretion, and vesicular transport	SNARE-complex protein Syntaxin-18 N-terminus
EVM0008917	21.10	72.47	-1.78	5.00E-05	4.28E-04	General function prediction only	Fcf1
EVM0004439	39.84	136.78	-1.78	5.00E-05	4.28E-04	General function prediction only	RNA cap guanine-N2 methyltransferase
EVM0005042	64.49	221.35	-1.78	5.00E-05	4.28E-04	Posttranslational modification, protein turnover, chaperones	Subtilase family
EVM0011505	0.40	1.35	-1.78	1.07E-02	3.95E-02	--	Cytochrome P450
EVM0010235	1.38	4.71	-1.77	5.00E-05	4.28E-04	--	--
EVM0006433	0.81	2.77	-1.77	2.50E-03	1.22E-02	Carbohydrate transport and metabolism	Major intrinsic protein
EVM0001527	5.48	18.73	-1.77	8.25E-03	3.21E-02	--	--
EVM0012122	8.78	30.00	-1.77	5.00E-05	4.28E-04	--	--
EVM0005631	0.97	3.30	-1.77	2.15E-03	1.08E-02	--	Domain of unknown function (DUF3415)

EVM0004988	3.41	11.65	-1.77	1.50E-04	1.14E-03	--	--
EVM0005087	91.19	311.07	-1.77	5.00E-05	4.28E-04	Intracellular trafficking, secretion, and vesicular transport	Tim17/Tim22/Tim23/Pmp24 family
EVM0011674	100.12	341.51	-1.77	6.50E-04	3.98E-03	--	--
EVM0002805	117.02	398.44	-1.77	5.00E-05	4.28E-04	Intracellular trafficking, secretion, and vesicular transport	Translocation protein Sec62
EVM0001552	12.27	41.72	-1.77	5.00E-05	4.28E-04	#N/A	#N/A
EVM0010587	60.18	204.48	-1.76	5.00E-05	4.28E-04	--	Cupin
EVM0004999	3.32	11.29	-1.76	5.00E-05	4.28E-04	Secondary metabolites biosynthesis, transport and catabolism	short chain dehydrogenase
EVM0006976	13.03	44.26	-1.76	5.00E-05	4.28E-04	Inorganic ion transport and metabolism	Regulator of volume decrease after cellular swelling
EVM0001956	46.58	158.16	-1.76	5.00E-05	4.28E-04	--	Gti1/Pac2 family
EVM0009386	8.19	27.81	-1.76	7.50E-04	4.49E-03	--	Phosphotransferase enzyme family
EVM0006067	344.09	1166.27	-1.76	5.00E-05	4.28E-04	Posttranslational modification, protein turnover, chaperones	Calreticulin family

EVM0007576	19.25	65.21	-1.76	5.00E-05	4.28E-04	Transcription	NLI interacting factor-like phosphatase
EVM0009577	17.05	57.76	-1.76	5.00E-05	4.28E-04	General function prediction only	GTP1/OBG
EVM0010421	19.62	66.44	-1.76	5.00E-05	4.28E-04	Function unknown	MBOAT, membrane-bound O-acyltransferase family
EVM0005227	39.94	135.16	-1.76	5.00E-05	4.28E-04	Function unknown	Bromodomain
EVM0008951	24.36	82.19	-1.75	5.00E-05	4.28E-04	Intracellular trafficking, secretion, and vesicular transport	Tetratricopeptide repeat
EVM0010581	51.04	172.17	-1.75	5.00E-05	4.28E-04	--	--
EVM0011734	2.25	7.59	-1.75	5.00E-05	4.28E-04	--	--
EVM0010645	8.74	29.48	-1.75	5.00E-05	4.28E-04	Cytoskeleton	Splicing factor, Prp19-binding domain
EVM0000041	1.24	4.18	-1.75	5.00E-05	4.28E-04	#N/A	#N/A
EVM0005323	6.86	23.13	-1.75	5.00E-05	4.28E-04	--	--
EVM0004358	195.63	658.94	-1.75	5.00E-05	4.28E-04	--	Heat shock factor binding protein 1
EVM0010862	89.85	302.39	-1.75	5.00E-05	4.28E-04	Translation, ribosomal structure and biogenesis	Ribosomal protein L24e

EVM0009691	22.42	75.39	-1.75	5.00E-05	4.28E-04	Carbohydrate transport and metabolism	pfkB family carbohydrate kinase
EVM0007258	1.60	5.37	-1.75	1.55E-03	8.22E-03	--	--
EVM0010575	14.56	48.88	-1.75	5.00E-05	4.28E-04	--	--
EVM0009512	21.62	72.49	-1.75	5.00E-05	4.28E-04	Lipid transport and metabolism	CoA-transferase family III
EVM0001725	9.80	32.87	-1.75	5.00E-05	4.28E-04	RNA processing and modification	AAA domain
EVM0009732	10.68	35.72	-1.74	5.00E-05	4.28E-04	--	--
EVM0000344	146.36	489.47	-1.74	5.00E-05	4.28E-04	--	SAM domain (Sterile alpha motif)
EVM0005236	327.58	1092.18	-1.74	5.00E-05	4.28E-04	RNA processing and modification	LSM domain
EVM0000915	33.14	110.24	-1.73	5.00E-05	4.28E-04	Translation, ribosomal structure and biogenesis	Ribosomal protein S5, C-terminal domain
EVM0003129	2.58	8.56	-1.73	5.00E-05	4.28E-04	--	--
EVM0002127	36.60	121.66	-1.73	5.00E-05	4.28E-04	RNA processing and modification	RNA recognition motif. (a.k.a. RRM, RBD, or RNP domain)
EVM0000557	20.43	67.81	-1.73	5.00E-05	4.28E-04	--	--
EVM0011852	46.96	155.72	-1.73	5.00E-05	4.28E-04	--	--

EVM0009023	10.77	35.70	-1.73	5.00E-05	4.28E-04	RNA processing and modification	Brix domain
EVM0009303	1.33	4.42	-1.73	5.00E-04	3.19E-03	--	--
EVM0010661	28.64	94.85	-1.73	5.00E-05	4.28E-04	Nucleotide transport and metabolism	AAA domain
EVM0001827	3.41	11.29	-1.73	5.00E-05	4.28E-04	Replication, recombination and repair	Helicase conserved C-terminal domain
EVM0005216	46.06	152.40	-1.73	5.00E-05	4.28E-04	--	Whi5 like
EVM0003691	144.98	479.42	-1.73	5.00E-05	4.28E-04	Translation, ribosomal structure and biogenesis	Ribosomal protein L4/L1 family
EVM0007146	14.37	47.50	-1.73	6.50E-04	3.98E-03	--	--
EVM0006271	41.40	136.85	-1.72	5.00E-05	4.28E-04	--	--
EVM0002912	152.57	503.86	-1.72	5.00E-05	4.28E-04	Function unknown	Telomere stability and silencing
EVM0010805	46.70	154.13	-1.72	5.00E-05	4.28E-04	General function prediction only	GMC oxidoreductase
EVM0010622	12.22	40.32	-1.72	5.00E-05	4.28E-04	--	--
EVM0003327	83.77	276.10	-1.72	5.00E-05	4.28E-04	--	Mitosis protein DIM1
EVM0010288	59.08	194.66	-1.72	5.00E-05	4.28E-04	Inorganic ion transport and metabolism	Cation efflux family

EVM0007930	8.61	28.34	-1.72	5.00E-05	4.28E-04	--	RNA recognition motif (a.k.a. RRM, RBD, or RNP domain)
EVM0010300	1.05	3.47	-1.72	3.00E-04	2.07E-03	--	F-box-like
EVM0004340	276.58	909.70	-1.72	5.00E-05	4.28E-04	--	Thioesterase superfamily
EVM0002613	202.01	664.20	-1.72	5.00E-05	4.28E-04	General function prediction only	RNA recognition motif. (a.k.a. RRM, RBD, or RNP domain)
EVM0008255	5.92	19.47	-1.72	5.00E-05	4.28E-04	--	--
EVM0012007	21.95	72.15	-1.72	5.00E-05	4.28E-04	--	--
EVM0012262	9.14	30.04	-1.72	5.00E-05	4.28E-04	Transcription	SHS2 domain found in N terminus of Rpb7p/Rpc25p/MJ0397
EVM0004872	137.22	450.71	-1.72	5.00E-05	4.28E-04	--	--
EVM0010113	61.21	201.03	-1.72	5.00E-05	4.28E-04	--	--
EVM0000628	34.15	112.13	-1.72	5.00E-05	4.28E-04	--	--
EVM0002542	138.25	453.78	-1.71	5.00E-05	4.28E-04	Lipid transport and metabolism	AMP-binding enzyme
EVM0001181	210.45	690.68	-1.71	5.00E-05	4.28E-04	Intracellular trafficking, secretion, and vesicular transport	Regulated-SNARE-like domain

EVM0006293	0.34	1.12	-1.71	2.80E-03	1.34E-02	--	--
EVM0007861	83.01	271.56	-1.71	5.00E-05	4.28E-04	--	Protein of unknown function (DUF1183)
EVM0002177	15.96	52.22	-1.71	5.00E-05	4.28E-04	General function prediction only	short chain dehydrogenase
EVM0002681	10.79	35.29	-1.71	5.00E-05	4.28E-04	General function prediction only	Glycosyl transferase family 90
EVM0007376	1.65	5.38	-1.71	7.00E-04	4.23E-03	--	--
EVM0000930	1.90	6.20	-1.71	5.00E-04	3.19E-03	--	--
EVM0004456	0.63	2.07	-1.71	7.40E-03	2.94E-02	Secondary metabolites biosynthesis, transport and catabolism	Alcohol dehydrogenase GroES-like domain
EVM0010163	3.79	12.37	-1.71	5.00E-05	4.28E-04	--	F-box-like
EVM0000220	21.49	70.03	-1.70	4.00E-04	2.65E-03	Carbohydrate transport and metabolism	UAA transporter family
EVM0012120	63.21	205.96	-1.70	5.00E-05	4.28E-04	Lipid transport and metabolism	3-hydroxyacyl-CoA dehydrogenase, NAD binding domain
EVM0009858	27.87	90.71	-1.70	5.00E-05	4.28E-04	Translation, ribosomal structure and biogenesis	CPL (NUC119) domain
EVM0000313	15.01	48.76	-1.70	5.00E-05	4.28E-04	Translation, ribosomal structure and biogenesis	RF-1 domain

EVM0005152	10.92	35.49	-1.70	5.00E-05	4.28E-04	--	--
EVM0009684	76.49	248.47	-1.70	5.00E-05	4.28E-04	Translation, ribosomal structure and biogenesis	Mitochondrial ribosomal protein L28
EVM0002623	8.06	26.15	-1.70	5.00E-05	4.28E-04	--	WD domain, G-beta repeat
EVM0003701	33.54	108.81	-1.70	5.00E-05	4.28E-04	Lipid transport and metabolism	Histidine phosphatase superfamily (branch 2)
EVM0007965	20.60	66.75	-1.70	5.00E-05	4.28E-04	Energy production and conversion	Mitochondrial carrier protein
EVM0002509	140.94	456.48	-1.70	5.00E-05	4.28E-04	--	--
EVM0011693	3.46	11.21	-1.69	5.00E-05	4.28E-04	--	F-box-like
EVM0001809	81.04	261.96	-1.69	5.00E-05	4.28E-04	--	--
EVM0007602	5.84	18.86	-1.69	5.00E-05	4.28E-04	Lipid transport and metabolism	PLD-like domain
EVM0000907	1.60	5.17	-1.69	4.00E-04	2.65E-03	--	--
EVM0011950	418.92	1347.21	-1.69	5.00E-05	4.28E-04	Posttranslational modification, protein turnover, chaperones	CS domain
EVM0008064	10.34	33.26	-1.69	5.00E-05	4.28E-04	--	Shugoshin C terminus

EVM0001772	171.87	552.27	-1.68	5.00E-05	4.28E-04	Translation, ribosomal structure and biogenesis	Ribosomal protein L36
EVM0001304	30.64	98.30	-1.68	5.00E-05	4.28E-04	Transcription	RNA recognition motif (a.k.a. RRM, RBD, or RNP domain)
EVM0003462	119.94	384.32	-1.68	5.00E-05	4.28E-04	Intracellular trafficking, secretion, and vesicular transport	Eukaryotic porin
EVM0001935	16.53	52.97	-1.68	5.00E-05	4.28E-04	--	Myosin-like coiled-coil protein
EVM0003796	5.54	17.73	-1.68	5.00E-05	4.28E-04	--	--
EVM0001752	1.93	6.17	-1.68	2.50E-04	1.77E-03	--	--
EVM0011785	4.52	14.45	-1.68	5.00E-05	4.28E-04	--	Fungal specific transcription factor domain
EVM0001766	120.57	385.02	-1.68	5.00E-05	4.28E-04	Intracellular trafficking, secretion, and vesicular transport	Signal recognition particle receptor beta subunit
EVM0009519	121.85	389.03	-1.67	5.00E-05	4.28E-04	Transcription	RNA polymerase Rpb3/Rpb11 dimerisation domain
EVM0009755	83.90	267.71	-1.67	5.00E-05	4.28E-04	Secondary metabolites biosynthesis, transport and catabolism	Cytochrome P450
EVM0004049	0.80	2.56	-1.67	1.90E-03	9.73E-03	General function prediction only	GMC oxidoreductase

EVM0004637	230.19	731.96	-1.67	5.00E-05	4.28E-04	--	--
EVM0007361	19.72	62.71	-1.67	5.00E-05	4.28E-04	RNA processing and modification	DEAD/DEAH box helicase
EVM0000804	92.88	295.06	-1.67	5.00E-05	4.28E-04	Lipid transport and metabolism	Acyl-CoA dehydrogenase, C-terminal domain
EVM0005225	14.60	46.36	-1.67	5.00E-05	4.28E-04	--	Gti1/Pac2 family
EVM0007358	8.84	28.05	-1.67	5.00E-05	4.28E-04	--	--
EVM0005754	4.58	14.48	-1.66	2.00E-03	1.01E-02	--	--
EVM0006354	37.98	120.09	-1.66	5.00E-05	4.28E-04	RNA processing and modification	RNA recognition motif. (a.k.a. RRM, RBD, or RNP domain)
EVM0012020	0.94	2.96	-1.66	6.85E-03	2.77E-02	--	--
EVM0009845	12.95	40.87	-1.66	5.00E-05	4.28E-04	--	--
EVM0004898	137.88	435.01	-1.66	5.00E-05	4.28E-04	Function unknown	Uncharacterized protein family UPF0016
EVM0007328	1407.82	4431.20	-1.65	5.00E-05	4.28E-04	Translation, ribosomal structure and biogenesis	60s Acidic ribosomal protein
EVM0010682	84.22	264.90	-1.65	1.50E-04	1.14E-03	--	Mitochondrial ribosomal protein L27
EVM0000447	27.97	87.96	-1.65	5.00E-05	4.28E-04	--	--

EVM0006974	1.33	4.17	-1.65	2.90E-03	1.38E-02	--	--
EVM0011509	54.64	171.68	-1.65	5.00E-05	4.28E-04	--	--
EVM0000165	46.51	145.88	-1.65	5.00E-05	4.28E-04	Cell cycle control, cell division, chromosome partitioning	Cyclin, N-terminal domain
EVM0005711	93.24	292.45	-1.65	5.00E-05	4.28E-04	Function unknown	Yip1 domain
EVM0010704	42.31	132.66	-1.65	5.00E-05	4.28E-04	Posttranslational modification, protein turnover, chaperones	--
EVM0008220	23.33	73.16	-1.65	5.00E-05	4.28E-04	Function unknown	Protein of unknown function (DUF1168)
EVM0006845	10.33	32.38	-1.65	5.00E-05	4.28E-04	--	SNF5 / SMARCB1 / INI1
EVM0004215	488.38	1530.46	-1.65	5.00E-05	4.28E-04	Energy production and conversion	Cytochrome c oxidase subunit Va
EVM0003574	38.63	121.03	-1.65	5.00E-05	4.28E-04	General function prediction only	PCI domain
EVM0010389	57.02	178.60	-1.65	1.00E-04	7.96E-04	Intracellular trafficking, secretion, and vesicular transport	Tim10/DDP family zinc finger
EVM0009757	125.51	392.95	-1.65	5.00E-05	4.28E-04	--	--

EVM0001548	827.17	2588.51	-1.65	5.00E-05	4.28E-04	Translation, ribosomal structure and biogenesis	Ribosomal protein S19e
EVM0005344	5.42	16.94	-1.64	5.00E-05	4.28E-04	--	Glycosyl hydrolases family 28
EVM0007176	33.04	103.14	-1.64	5.00E-05	4.28E-04	Energy production and conversion	D-isomer specific 2-hydroxyacid dehydrogenase, NAD binding domain
EVM0010758	18.86	58.89	-1.64	5.00E-05	4.28E-04	RNA processing and modification	RNA 3'-terminal phosphate cyclase
EVM0009680	135.46	422.52	-1.64	5.00E-05	4.28E-04	Translation, ribosomal structure and biogenesis	Ribosomal protein L16p/L10e
EVM0006626	65.78	205.11	-1.64	5.00E-05	4.28E-04	--	--
EVM0005217	86.05	268.10	-1.64	5.00E-05	4.28E-04	--	Glycosyl hydrolases family 16
EVM0007719	1.61	5.02	-1.64	8.50E-04	4.98E-03	--	--
EVM0004245	13.63	42.43	-1.64	5.00E-05	4.28E-04	Carbohydrate transport and metabolism	Glycosyl transferase family 4
EVM0007803	4.82	14.94	-1.63	2.00E-04	1.46E-03	--	--
EVM0001630	20.79	64.52	-1.63	5.00E-05	4.28E-04	Replication, recombination and repair	ESCO1/2 acetyl-transferase
EVM0005888	233.16	723.37	-1.63	5.00E-05	4.28E-04	Energy production and conversion	Cytochrome C1 family

EVM0005500	13.73	42.56	-1.63	5.00E-05	4.28E-04	Function unknown	XAP5, circadian clock regulator
EVM0007772	28.67	88.79	-1.63	5.00E-05	4.28E-04	--	Thioredoxin
EVM0002324	15.06	46.64	-1.63	5.00E-05	4.28E-04	Lipid transport and metabolism	Cytochrome b5-like Heme/Steroid binding domain
EVM0007428	4.51	13.98	-1.63	5.00E-05	4.28E-04	--	--
EVM0010106	40.49	125.22	-1.63	5.00E-05	4.28E-04	Cell cycle control, cell division, chromosome partitioning	Maf-like protein
EVM0006403	132.04	408.31	-1.63	5.00E-05	4.28E-04	Inorganic ion transport and metabolism	Anion-transporting ATPase
EVM0001435	68.91	212.91	-1.63	5.00E-05	4.28E-04	--	Mycolic acid cyclopropane synthetase
EVM0003224	67.85	209.58	-1.63	5.00E-05	4.28E-04	General function prediction only	Phosphatidylethanolamine-binding protein
EVM0001103	4.16	12.83	-1.63	1.00E-04	7.96E-04	--	Glycosyl hydrolases family 16
EVM0004815	50.52	155.66	-1.62	5.00E-05	4.28E-04	RNA processing and modification	LSM domain
EVM0007696	7.57	23.32	-1.62	5.00E-05	4.28E-04	--	Domain of unknown function (DUF4139)
EVM0002263	1.93	5.94	-1.62	2.50E-04	1.77E-03	--	--

EVM0007331	4.19	12.89	-1.62	5.00E-05	4.28E-04	--	--
EVM0003839	537.50	1654.23	-1.62	5.00E-05	4.28E-04	Translation, ribosomal structure and biogenesis	Ribosomal S13/S15 N-terminal domain
EVM0011083	14.73	45.31	-1.62	5.00E-05	4.28E-04	--	--
EVM0000240	10.98	33.76	-1.62	5.00E-05	4.28E-04	Transcription	MED6 mediator sub complex component
EVM0004659	0.74	2.27	-1.62	9.60E-03	3.63E-02	--	G protein-coupled glucose receptor regulating Gpa2
EVM0010641	24.32	74.69	-1.62	5.00E-05	4.28E-04	Signal transduction mechanisms	ERG2 and Sigma1 receptor like protein
EVM0007720	53.76	165.13	-1.62	5.00E-05	4.28E-04	Posttranslational modification, protein turnover, chaperones	Glutathione S-transferase, N-terminal domain
EVM0009851	12.02	36.93	-1.62	5.00E-05	4.28E-04	Function unknown	WD domain, G-beta repeat
EVM0008230	24.18	74.24	-1.62	5.00E-05	4.28E-04	Transcription	Maf1 regulator
EVM0006946	9.90	30.38	-1.62	5.00E-05	4.28E-04	--	--
EVM0001624	5.44	16.67	-1.62	1.15E-03	6.41E-03	--	--
EVM0010207	157.23	482.14	-1.62	5.00E-05	4.28E-04	Intracellular trafficking, secretion, and vesicular transport	emp24/gp25L/p24 family/GOLD

EVM0011300	2.84	8.69	-1.62	3.00E-04	2.07E-03	--	--
EVM0003532	31.68	97.00	-1.61	5.00E-05	4.28E-04	General function prediction only	Arrestin (or S-antigen), C-terminal domain
EVM0007379	32.01	97.94	-1.61	5.00E-05	4.28E-04	--	--
EVM0000717	2.75	8.41	-1.61	2.00E-04	1.46E-03	--	TEA/ATTS domain family
EVM0003992	17.66	53.99	-1.61	5.00E-05	4.28E-04	--	--
EVM0002603	0.55	1.68	-1.61	1.40E-03	7.55E-03	--	--
EVM0010657	553.41	1689.22	-1.61	4.00E-04	2.65E-03	Posttranslational modification, protein turnover, chaperones	Hsp70 protein
EVM0002218	106.71	325.66	-1.61	5.00E-05	4.28E-04	--	--
EVM0005403	15.88	48.46	-1.61	5.00E-05	4.28E-04	Translation, ribosomal structure and biogenesis	Elongation factor TS
EVM0002008	2.30	7.02	-1.61	1.00E-04	7.96E-04	--	--
EVM0003419	0.99	3.01	-1.61	1.00E-04	7.96E-04	--	--
EVM0007433	4.69	14.29	-1.61	5.00E-05	4.28E-04	--	--

EVM0006443	1.61	4.89	-1.61	2.00E-04	1.46E-03	General function prediction only	RNA recognition motif. (a.k.a. RRM, RBD, or RNP domain)
EVM0005885	159.00	484.05	-1.61	5.00E-05	4.28E-04	Intracellular trafficking, secretion, and vesicular transport	emp24/gp25L/p24 family/GOLD
EVM0003399	2.56	7.79	-1.61	4.00E-04	2.65E-03	--	--
EVM0009247	83.67	254.56	-1.61	5.00E-05	4.28E-04	Transcription	NLI interacting factor-like phosphatase
EVM0001091	1.32	4.02	-1.60	5.00E-05	4.28E-04	--	Glycoside hydrolase family 44
EVM0008532	108.90	330.67	-1.60	5.00E-05	4.28E-04	Translation, ribosomal structure and biogenesis	Ribosomal L27 protein
EVM0001586	232.64	706.04	-1.60	5.00E-05	4.28E-04	Function unknown	Eukaryotic family of unknown function (DUF1754)
EVM0005151	432.59	1312.82	-1.60	5.00E-05	4.28E-04	--	--
EVM0009204	11.51	34.94	-1.60	5.00E-05	4.28E-04	RNA processing and modification	--
EVM0001878	12.76	38.69	-1.60	5.00E-05	4.28E-04	--	--
EVM0007904	1.47	4.46	-1.60	2.00E-04	1.46E-03	--	--
EVM0004418	33.29	100.70	-1.60	5.00E-05	4.28E-04	--	--

EVM0000820	42.52	128.52	-1.60	5.00E-05	4.28E-04	Posttranslational modification, protein turnover, chaperones	Ubiquitin-conjugating enzyme
EVM0010393	9.18	27.73	-1.60	5.00E-05	4.28E-04	--	Lactonase, 7-bladed beta-propeller
EVM0000847	3.51	10.60	-1.60	1.10E-03	6.17E-03	--	--
EVM0001632	37.05	111.93	-1.60	5.00E-05	4.28E-04	--	--
EVM0005634	6.84	20.65	-1.59	5.00E-05	4.28E-04	--	--
EVM0011727	175.33	529.11	-1.59	5.00E-05	4.28E-04	Posttranslational modification, protein turnover, chaperones	Ubiquitin-2 like Rad60 SUMO-like
EVM0011467	40.43	121.99	-1.59	5.00E-05	4.28E-04	Function unknown	YIF1
EVM0007837	1756.10	5291.10	-1.59	5.00E-05	4.28E-04	Translation, ribosomal structure and biogenesis	Ribosomal protein S17
EVM0005707	7.84	23.62	-1.59	5.00E-05	4.28E-04	--	--
EVM0000539	52.57	158.22	-1.59	5.00E-05	4.28E-04	Function unknown	Zinc carboxypeptidase
EVM0005112	0.93	2.79	-1.59	5.00E-04	3.19E-03	Amino acid transport and metabolism	Peptidase family M20/M25/M40
EVM0010727	27.14	81.60	-1.59	5.00E-05	4.28E-04	General function prediction only	Alpha/beta hydrolase family

EVM0000209	7.53	22.63	-1.59	5.00E-05	4.28E-04	Translation, ribosomal structure and biogenesis	KRI1-like family C-terminal
EVM0000653	42.40	127.30	-1.59	5.00E-05	4.28E-04	Function unknown	Domain of unknown function (DUF947)
EVM0006687	7.49	22.49	-1.59	5.00E-05	4.28E-04	--	--
EVM0009646	4.63	13.88	-1.58	9.70E-03	3.66E-02	--	Pheromone A receptor
EVM0000044	297.23	890.64	-1.58	5.00E-05	4.28E-04	Intracellular trafficking, secretion, and vesicular transport	Tim10/DDP family zinc finger
EVM0011482	32.31	96.74	-1.58	5.00E-05	4.28E-04	Function unknown	WD domain, G-beta repeat
EVM0006207	8.37	25.05	-1.58	1.00E-04	7.96E-04	--	--
EVM0009735	199.24	595.94	-1.58	5.00E-05	4.28E-04	--	Oxidoreductase FAD-binding domain
EVM0011042	107.56	321.64	-1.58	5.00E-05	4.28E-04	Signal transduction mechanisms	Inhibitor of apoptosis-promoting Bax1
EVM0005109	2.85	8.53	-1.58	1.00E-04	7.96E-04	--	--
EVM0002381	58.63	175.31	-1.58	5.00E-05	4.28E-04	--	Nucleoporin complex subunit 54
EVM0000616	86.45	258.49	-1.58	5.00E-05	4.28E-04	--	SNARE domain

EVM0008292	12.39	37.02	-1.58	5.00E-05	4.28E-04	Posttranslational modification, protein turnover, chaperones	IBR domain
EVM0011745	17.29	51.67	-1.58	5.00E-05	4.28E-04	--	--
EVM0006119	5.19	15.48	-1.58	5.00E-05	4.28E-04	Secondary metabolites biosynthesis, transport and catabolism	short chain dehydrogenase
EVM0011744	19.76	58.99	-1.58	1.35E-02	4.74E-02	Signal transduction mechanisms	TGS domain
EVM0003303	302.61	902.69	-1.58	5.00E-05	4.28E-04	--	--
EVM0008367	1.73	5.16	-1.58	2.50E-04	1.77E-03	--	Protein kinase domain
EVM0010051	4.77	14.23	-1.58	5.00E-05	4.28E-04	--	Glycosyl hydrolase family 3 N terminal domain
EVM0000483	103.27	307.65	-1.57	5.00E-05	4.28E-04	Amino acid transport and metabolism	Chorismate mutase type II
EVM0004156	0.74	2.20	-1.57	5.00E-05	4.28E-04	--	--
EVM0005014	8.10	24.13	-1.57	5.00E-05	4.28E-04	--	--
EVM0010922	161.05	479.68	-1.57	5.00E-05	4.28E-04	--	Nitronate monooxygenase
EVM0005981	4.50	13.38	-1.57	5.00E-05	4.28E-04	--	--

EVM0007859	5.80	17.23	-1.57	5.00E-05	4.28E-04	--	--
EVM0010410	13.65	40.56	-1.57	5.00E-05	4.28E-04	Defense mechanisms	Isochorismatase family
EVM0003501	24.06	71.45	-1.57	5.00E-05	4.28E-04	--	Flavin containing amine oxidoreductase
EVM0002323	1.01	2.99	-1.57	1.25E-03	6.87E-03	General function prediction only	Sugar (and other) transporter
EVM0005875	54.24	160.89	-1.57	5.00E-05	4.28E-04	--	--
EVM0002330	182.48	540.42	-1.57	5.00E-05	4.28E-04	--	GGL domain
EVM0006072	4.78	14.15	-1.57	4.25E-03	1.89E-02	--	--
EVM0001178	27.67	81.84	-1.56	5.00E-05	4.28E-04	--	--
EVM0000583	121.22	358.26	-1.56	5.00E-05	4.28E-04	Translation, ribosomal structure and biogenesis	Ribosomal protein L1p/L10e family
EVM0006475	24.09	71.18	-1.56	5.00E-05	4.28E-04	--	AAA domain
EVM0001105	2.52	7.45	-1.56	1.00E-04	7.96E-04	Carbohydrate transport and metabolism	Glycosyl hydrolase family 47
EVM0004611	4.30	12.68	-1.56	1.00E-04	7.96E-04	--	Ferritin-like domain
EVM0011815	38.87	114.71	-1.56	1.00E-04	7.96E-04	--	Membrane-associating domain

EVM0006494	15.29	45.11	-1.56	5.00E-05	4.28E-04	Chromatin structure and dynamics	Sas10 C-terminal domain
EVM0008434	47.16	139.17	-1.56	5.00E-05	4.28E-04	--	--
EVM0008578	4.71	13.88	-1.56	5.00E-05	4.28E-04	--	Glycosyl hydrolase family 71
EVM0009662	32.04	94.45	-1.56	5.00E-05	4.28E-04	Inorganic ion transport and metabolism	Cation efflux family
EVM0000189	4.27	12.57	-1.56	5.00E-05	4.28E-04	--	Glycosyl hydrolases family 43
EVM0011601	76.61	225.49	-1.56	5.00E-05	4.28E-04	--	--
EVM0005496	345.96	1017.61	-1.56	5.00E-05	4.28E-04	--	--
EVM0010403	7.81	22.98	-1.56	5.00E-05	4.28E-04	--	--
EVM0002657	362.68	1066.26	-1.56	5.00E-05	4.28E-04	General function prediction only	short chain dehydrogenase
EVM0009199	60.83	178.67	-1.55	5.00E-05	4.28E-04	Signal transduction mechanisms	Protein phosphatase 2C
EVM0004868	13.62	39.98	-1.55	5.00E-05	4.28E-04	--	F-box-like
EVM0005966	20.45	60.03	-1.55	5.00E-05	4.28E-04	General function prediction only	PPR repeat family

EVM0004301	27.42	80.38	-1.55	5.00E-05	4.28E-04	Secondary metabolites biosynthesis, transport and catabolism	Pyridine nucleotide-disulphide oxidoreductase
EVM0001371	87.74	257.04	-1.55	5.00E-05	4.28E-04	--	Mitochondrial ribosomal protein S25
EVM0004111	72.45	212.12	-1.55	5.00E-05	4.28E-04	Signal transduction mechanisms	Glucosidase II beta subunit-like
EVM0004939	22.97	67.20	-1.55	5.00E-05	4.28E-04	RNA processing and modification	RNAse P Rpr2/Rpp21/SNM1 subunit domain
EVM0006461	6.25	18.29	-1.55	1.00E-04	7.96E-04	Lipid transport and metabolism	Acyltransferase
EVM0009944	6.66	19.45	-1.55	5.00E-05	4.28E-04	--	--
EVM0010781	114.60	334.44	-1.55	5.00E-05	4.28E-04	General function prediction only	Ribosomal protein S8e
EVM0006895	2.86	8.34	-1.55	2.35E-03	1.16E-02	--	Protein of unknown function (DUF3129)
EVM0005306	41.70	121.68	-1.54	5.00E-05	4.28E-04	Cell wall/membrane/envelope biogenesis	Glycosyl transferases group 1
EVM0006578	93.35	272.30	-1.54	5.00E-05	4.28E-04	Intracellular trafficking, secretion, and vesicular transport	Syntaxin
EVM0009689	118.71	346.16	-1.54	5.00E-05	4.28E-04	Translation, ribosomal structure and biogenesis	Ribosomal protein S17

EVM0008363	43.74	127.52	-1.54	5.00E-05	4.28E-04	--	--
EVM0005442	42.58	123.96	-1.54	5.00E-05	4.28E-04	General function prediction only	TPR repeat
EVM0003398	0.62	1.79	-1.54	1.11E-02	4.07E-02	Secondary metabolites biosynthesis, transport and catabolism	Cytochrome P450
EVM0002287	38.57	111.94	-1.54	1.90E-03	9.73E-03	--	Cytochrome P450
EVM0006140	13.64	39.58	-1.54	5.00E-05	4.28E-04	--	--
EVM0011958	0.60	1.74	-1.54	5.85E-03	2.44E-02	--	--
EVM0011214	38.16	110.70	-1.54	6.50E-04	3.98E-03	Posttranslational modification, protein turnover, chaperones	Pex2 / Pex12 amino terminal region
EVM0005335	11.32	32.83	-1.54	5.00E-05	4.28E-04	--	F-box-like
EVM0010591	27.21	78.91	-1.54	5.00E-05	4.28E-04	--	--
EVM0006349	8.70	25.24	-1.54	5.00E-05	4.28E-04	Replication, recombination and repair	Sas10/Utp3/C1D family
EVM0012035	113.44	328.86	-1.54	5.00E-05	4.28E-04	--	Yqey-like protein
EVM0008977	2.01	5.83	-1.53	2.15E-03	1.08E-02	--	--

EVM0008651	93.46	270.68	-1.53	5.00E-05	4.28E-04	Inorganic ion transport and metabolism	Copper/zinc superoxide dismutase (SODC)
EVM0005247	38.72	112.13	-1.53	5.00E-05	4.28E-04	Function unknown	Breast carcinoma amplified sequence 2 (BCAS2)
EVM0008198	38.19	110.46	-1.53	5.00E-05	4.28E-04	Transcription	Transcription initiation factor IIF, beta subunit
EVM0010777	39.56	114.34	-1.53	5.00E-05	4.28E-04	Energy production and conversion	Mitochondrial carrier protein
EVM0000792	36.01	104.00	-1.53	5.00E-05	4.28E-04	RNA processing and modification	U1 zinc finger
EVM0000533	84.73	244.61	-1.53	5.00E-05	4.28E-04	Translation, ribosomal structure and biogenesis	Ribosomal protein L11, RNA binding domain
EVM0006914	5.04	14.55	-1.53	1.00E-04	7.96E-04	--	--
EVM0001473	20.28	58.50	-1.53	5.00E-05	4.28E-04	Intracellular trafficking, secretion, and vesicular transport	Transport protein particle (TRAPP) component
EVM0006044	7.47	21.53	-1.53	5.00E-05	4.28E-04	--	--
EVM0006170	7.57	21.81	-1.53	5.00E-05	4.28E-04	--	--
EVM0000381	11.44	32.95	-1.53	5.00E-05	4.28E-04	Function unknown	Folate-sensitive fragile site protein Fra10Ac1
EVM0006165	7.53	21.70	-1.53	5.00E-05	4.28E-04	--	--

EVM0008279	2.60	7.47	-1.53	6.15E-03	2.54E-02	--	--
EVM0007413	38.63	111.17	-1.53	5.00E-05	4.28E-04	General function prediction only	Haloacid dehalogenase-like hydrolase
EVM0009740	60.61	174.29	-1.52	5.00E-05	4.28E-04	Function unknown	Uncharacterised protein family (UPF0203)
EVM0003055	116.10	333.77	-1.52	5.00E-05	4.28E-04	Function unknown	NUDIX domain
EVM0010179	121.57	349.47	-1.52	5.00E-05	4.28E-04	RNA processing and modification	Complex1_LYR-like
EVM0009477	9.72	27.92	-1.52	5.00E-05	4.28E-04	--	F-box-like
EVM0012003	6.17	17.71	-1.52	5.00E-05	4.28E-04	--	--
EVM0001084	42.04	120.71	-1.52	5.00E-05	4.28E-04	--	Uncharacterised conserved protein (DUF2315)
EVM0001335	10.05	28.83	-1.52	5.00E-05	4.28E-04	RNA processing and modification	Ribosomal L28e protein family
EVM0003278	1.46	4.19	-1.52	1.27E-02	4.53E-02	--	--
EVM0010557	4.86	13.92	-1.52	5.00E-05	4.28E-04	--	Repair protein Rad1/Rec1/Rad17
EVM0005814	19.63	56.15	-1.52	5.00E-05	4.28E-04	General function prediction only	MORN repeat
EVM0011177	19.53	55.81	-1.51	5.00E-05	4.28E-04	RNA processing and modification	Helicase associated domain (HA2)

EVM0004661	5.29	15.12	-1.51	5.00E-05	4.28E-04	--	Cytochrome P450
EVM0011442	105.64	301.74	-1.51	5.00E-05	4.28E-04	Translation, ribosomal structure and biogenesis	Eukaryotic translation initiation factor 3 subunit G
EVM0003096	153.07	436.71	-1.51	5.00E-05	4.28E-04	--	--
EVM0002273	14.28	40.73	-1.51	5.00E-05	4.28E-04	RNA processing and modification	--
EVM0011802	44.02	125.51	-1.51	5.00E-05	4.28E-04	Energy production and conversion	Glycerophosphoryl diester phosphodiesterase family
EVM0002693	49.79	141.93	-1.51	1.00E-04	7.96E-04	--	--
EVM0004912	57.99	165.24	-1.51	5.00E-05	4.28E-04	--	--
EVM0005134	108.41	308.82	-1.51	5.00E-05	4.28E-04	Function unknown	COP9 signalosome, subunit CSN8
EVM0003487	61.69	175.58	-1.51	5.00E-05	4.28E-04	--	Eukaryotic cytochrome b561
EVM0002011	1.08	3.08	-1.51	7.25E-03	2.90E-02	--	--
EVM0001937	4.58	13.01	-1.51	5.00E-05	4.28E-04	--	--
EVM0010312	16.05	45.61	-1.51	5.00E-05	4.28E-04	Cell cycle control, cell division, chromosome partitioning	Protein kinase domain

EVM0002296	44.72	127.09	-1.51	5.00E-05	4.28E-04	Posttranslational modification, protein turnover, chaperones	DnaJ C terminal domain
EVM0002648	2.05	5.82	-1.51	5.00E-05	4.28E-04	--	Glycosyl hydrolase family 7
EVM0007842	52.11	147.89	-1.50	5.00E-05	4.28E-04	--	Hus1-like protein
EVM0002302	21.43	60.80	-1.50	5.00E-05	4.28E-04	--	--
EVM0009800	217.08	615.42	-1.50	5.00E-05	4.28E-04	--	Mitochondrial ribosomal protein L31
EVM0003787	57.92	164.19	-1.50	5.00E-05	4.28E-04	Translation, ribosomal structure and biogenesis	Eukaryotic translation initiation factor 3 subunit 7 (eIF-3)
EVM0003274	31.80	90.13	-1.50	5.00E-05	4.28E-04	RNA processing and modification	Domain of unknown function (DUF3449)
EVM0008237	24.44	69.26	-1.50	5.00E-05	4.28E-04	--	RNA ligase
EVM0000072	3.59	10.16	-1.50	4.00E-04	2.65E-03	Posttranslational modification, protein turnover, chaperones	Eukaryotic aspartyl protease
EVM0000654	126.44	357.56	-1.50	5.00E-05	4.28E-04	General function prediction only	Enoyl-(Acyl carrier protein) reductase
EVM0000005	18.74	52.99	-1.50	2.50E-04	1.77E-03	--	--
EVM0009341	10.72	30.32	-1.50	5.00E-05	4.28E-04	RNA processing and modification	DEAD/DEAH box helicase

EVM0007418	44.52	125.75	-1.50	5.00E-05	4.28E-04	Carbohydrate transport and metabolism	Protein kinase domain
EVM0002012	3963.77	11195.70	-1.50	2.00E-04	1.46E-03	--	Ser-Thr-rich glycosyl-phosphatidyl-inositol-anchored membrane family
EVM0000992	65.15	183.90	-1.50	5.00E-05	4.28E-04	--	Carbonic anhydrase
EVM0010693	184.39	520.36	-1.50	5.00E-05	4.28E-04	--	--
EVM0001127	44.96	126.62	-1.49	5.00E-05	4.28E-04	General function prediction only	Ras family
EVM0007840	512.12	1442.06	-1.49	5.00E-05	4.28E-04	Energy production and conversion	Mitochondrial ribosomal protein L51 / S25 / CI-B8 domain
EVM0010552	18.02	50.75	-1.49	5.00E-05	4.28E-04	--	--
EVM0004171	16.21	45.62	-1.49	5.00E-05	4.28E-04	--	Histone-like transcription factor (CBF/NF-Y) and archaeal histone
EVM0004576	67.71	190.51	-1.49	5.00E-05	4.28E-04	General function prediction only	Acetyltransferase (GNAT) family
EVM0001419	179.59	505.13	-1.49	5.00E-05	4.28E-04	Signal transduction mechanisms	SGS domain
EVM0006355	160.18	450.33	-1.49	5.00E-05	4.28E-04	Intracellular trafficking, secretion, and vesicular transport	Microsomal signal peptidase 12 kDa subunit (SPC12)

EVM0010543	2.19	6.14	-1.49	2.00E-04	1.46E-03	--	--
EVM0004513	82.31	230.98	-1.49	2.95E-03	1.40E-02	--	pfkB family carbohydrate kinase
EVM0002548	18.97	53.22	-1.49	5.00E-05	4.28E-04	--	--
EVM0000131	3.95	11.08	-1.49	1.75E-03	9.08E-03	--	--
EVM0008273	11.10	31.09	-1.49	5.00E-05	4.28E-04	--	Glycosyl hydrolase family 3 N terminal domain
EVM0011440	1.08	3.02	-1.48	4.00E-04	2.65E-03	--	--
EVM0010019	2.50	7.00	-1.48	5.00E-05	4.28E-04	--	F-box-like
EVM0000377	53.36	149.25	-1.48	5.00E-05	4.28E-04	--	--
EVM0004148	59.82	167.18	-1.48	5.00E-05	4.28E-04	--	--
EVM0009904	44.22	123.57	-1.48	5.00E-05	4.28E-04	General function prediction only	Methyltransferase involved in Williams-Beuren syndrome
EVM0009025	34.63	96.68	-1.48	5.00E-05	4.28E-04	--	--
EVM0001444	32.04	89.47	-1.48	5.00E-05	4.28E-04	--	--
EVM0000883	3.86	10.79	-1.48	5.00E-05	4.28E-04	Transcription	HMG (high mobility group) box

EVM0005158	6.53	18.23	-1.48	5.00E-05	4.28E-04	--	Mitochondrial carrier protein
EVM0009946	26.63	74.29	-1.48	5.00E-05	4.28E-04	Transcription	A49-like RNA polymerase I associated factor
EVM0001024	41.84	116.63	-1.48	5.00E-05	4.28E-04	--	Hinge domain of cleavage stimulation factor subunit 2
EVM0007991	79.25	220.62	-1.48	5.00E-05	4.28E-04	Transcription	SAC3/GANP/Nin1/mts3/eIF-3 p25 family
EVM0007810	4.18	11.64	-1.48	1.00E-04	7.96E-04	--	--
EVM0001207	8.37	23.26	-1.48	1.50E-04	1.14E-03	--	--
EVM0001831	487.13	1353.48	-1.47	5.00E-05	4.28E-04	Energy production and conversion	ATP synthase, Delta/Epsilon chain, beta-sandwich domain
EVM0009011	0.76	2.10	-1.47	5.00E-03	2.15E-02	--	F-box-like
EVM0007377	9.47	26.23	-1.47	5.00E-05	4.28E-04	RNA processing and modification	RNA pseudouridylate synthase
EVM0000869	474.64	1314.67	-1.47	2.50E-04	1.77E-03	--	--
EVM0007065	45.00	124.25	-1.47	5.00E-05	4.28E-04	--	--
EVM0008014	971.59	2683.00	-1.47	5.00E-05	4.28E-04	Translation, ribosomal structure and biogenesis	Ribosomal protein L35Ae

EVM0009270	772.11	2130.61	-1.46	5.00E-05	4.28E-04	Posttranslational modification, protein turnover, chaperones	Chaperonin 10 Kd subunit
EVM0003630	30.34	83.71	-1.46	5.00E-05	4.28E-04	General function prediction only	NGP1NT (NUC091) domain
EVM0007726	6.85	18.90	-1.46	5.00E-05	4.28E-04	Signal transduction mechanisms	Protein tyrosine kinase
EVM0011961	114.49	315.80	-1.46	5.00E-05	4.28E-04	General function prediction only	Zinc finger C-x8-C-x5-C-x3-H type (and similar)
EVM0007748	66.42	183.17	-1.46	1.75E-03	9.08E-03	--	--
EVM0000417	13.87	38.24	-1.46	5.00E-05	4.28E-04	Posttranslational modification, protein turnover, chaperones	DnaJ domain
EVM0008299	208.07	573.04	-1.46	5.00E-05	4.28E-04	Intracellular trafficking, secretion, and vesicular transport	emp24/gp25L/p24 family/GOLD
EVM0002619	22.05	60.64	-1.46	5.00E-05	4.28E-04	Function unknown	Signal peptide peptidase
EVM0008121	125.90	345.92	-1.46	5.00E-05	4.28E-04	--	RNA polymerase II transcription mediator complex subunit 9
EVM0008389	437.37	1201.25	-1.46	5.00E-05	4.28E-04	Energy production and conversion	Complex 1 protein (LYR family)
EVM0008637	14.82	40.67	-1.46	5.00E-05	4.28E-04	RNA processing and modification	WD domain, G-beta repeat

EVM0007616	23.95	65.73	-1.46	5.00E-05	4.28E-04	Transcription	MT-A70
EVM0008206	98.65	270.25	-1.45	5.00E-05	4.28E-04	RNA processing and modification	RNA recognition motif. (a.k.a. RRM, RBD, or RNP domain)
EVM0002482	9.36	25.63	-1.45	5.00E-05	4.28E-04	--	--
EVM0000647	46.75	127.92	-1.45	1.00E-04	7.96E-04	#N/A	#N/A
EVM0007650	114.01	311.92	-1.45	5.00E-05	4.28E-04	--	--
EVM0003400	5.86	16.03	-1.45	5.00E-05	4.28E-04	--	Centromere protein Scm3
EVM0001350	376.65	1027.81	-1.45	1.50E-04	1.14E-03	--	CsbD-like
EVM0011888	0.84	2.30	-1.45	7.00E-03	2.82E-02	--	Peroxidase
EVM0012015	20.09	54.81	-1.45	5.00E-05	4.28E-04	Translation, ribosomal structure and biogenesis	BOP1NT (NUC169) domain
EVM0003340	12.68	34.57	-1.45	5.00E-05	4.28E-04	--	Nuclear fragile X mental retardation-interacting protein 1 (NUFIP1)
EVM0010318	4.94	13.46	-1.45	1.50E-04	1.14E-03	--	--
EVM0000425	767.73	2091.15	-1.45	5.00E-05	4.28E-04	Intracellular trafficking, secretion, and vesicular transport	TB2/DP1, HVA22 family

EVM0008908	53.48	145.61	-1.45	5.00E-05	4.28E-04	Translation, ribosomal structure and biogenesis	Brix domain
EVM0012226	2.60	7.07	-1.44	2.05E-03	1.03E-02	--	--
EVM0009933	76.63	208.48	-1.44	5.00E-05	4.28E-04	Intracellular trafficking, secretion, and vesicular transport	Peptidase S24-like
EVM0006452	5933.64	16138.20	-1.44	5.00E-05	4.28E-04	--	--
EVM0000264	0.51	1.37	-1.44	5.05E-03	2.17E-02	--	--
EVM0004038	1161.22	3149.55	-1.44	5.00E-04	3.19E-03	Translation, ribosomal structure and biogenesis	Ribosomal protein L6
EVM0004951	1.73	4.68	-1.44	2.50E-04	1.77E-03	--	--
EVM0005028	28.68	77.69	-1.44	5.00E-05	4.28E-04	Inorganic ion transport and metabolism	Acetyl-coenzyme A transporter 1
EVM0008305	14.15	38.34	-1.44	5.00E-05	4.28E-04	General function prediction only	U3 small nucleolar RNA-associated protein 6
EVM0003326	32.29	87.47	-1.44	5.00E-05	4.28E-04	General function prediction only	R3H domain
EVM0003685	8.04	21.78	-1.44	5.00E-05	4.28E-04	--	--
EVM0006691	1.15	3.10	-1.44	2.30E-03	1.14E-02	--	--

EVM0000211	24.18	65.38	-1.44	5.00E-05	4.28E-04	RNA processing and modification	WD domain, G-beta repeat
EVM0007596	20.25	54.73	-1.43	5.00E-05	4.28E-04	General function prediction only	RNA recognition motif. (a.k.a. RRM, RBD, or RNP domain)
EVM0006014	118.97	320.98	-1.43	5.00E-05	4.28E-04	--	--
EVM0000252	15.85	42.69	-1.43	5.00E-05	4.28E-04	--	--
EVM0000081	80.72	217.34	-1.43	5.00E-05	4.28E-04	--	WW domain
EVM0008740	19.07	51.34	-1.43	5.00E-05	4.28E-04	--	Putative amidotransferase
EVM0008159	92.95	250.20	-1.43	5.00E-05	4.28E-04	Posttranslational modification, protein turnover, chaperones	Hsp70 protein
EVM0000121	48.80	131.33	-1.43	5.00E-05	4.28E-04	General function prediction only	Haloacid dehalogenase-like hydrolase
EVM0008276	56.54	152.13	-1.43	5.00E-05	4.28E-04	Inorganic ion transport and metabolism	Ion channel
EVM0009271	35.95	96.68	-1.43	5.00E-05	4.28E-04	--	Protein kinase domain
EVM0004796	45.32	121.87	-1.43	5.00E-05	4.28E-04	Function unknown	Fcf2 pre-rRNA processing
EVM0008074	142.26	382.42	-1.43	5.00E-05	4.28E-04	Chromatin structure and dynamics	Core histone H2A/H2B/H3/H4

EVM0006924	14.14	38.01	-1.43	5.00E-05	4.28E-04	--	--
EVM0000928	140.07	376.37	-1.43	5.00E-05	4.28E-04	Function unknown	Der1-like family
EVM0009294	151.75	407.56	-1.43	5.00E-05	4.28E-04	Lipid transport and metabolism	Fatty acid hydroxylase superfamily
EVM0002100	16.20	43.51	-1.43	5.00E-05	4.28E-04	--	Helix-loop-helix DNA-binding domain
EVM0006480	9.12	24.49	-1.43	5.00E-05	4.28E-04	--	Membrane transport protein
EVM0006700	195.99	526.28	-1.43	1.50E-04	1.14E-03	--	Aconitase family (aconitate hydratase)
EVM0012187	10.73	28.82	-1.42	5.00E-05	4.28E-04	--	Fungal cellulose binding domain
EVM0000784	7.75	20.82	-1.42	5.00E-05	4.28E-04	--	--
EVM0002505	684.07	1835.01	-1.42	2.00E-04	1.46E-03	--	--
EVM0000600	152.36	407.67	-1.42	5.00E-05	4.28E-04	--	Nitronate monooxygenase
EVM0011341	180.98	483.99	-1.42	5.00E-05	4.28E-04	--	CDP-alcohol phosphatidyltransferase
EVM0000593	135.73	362.89	-1.42	5.00E-05	4.28E-04	--	Protein of unknown function (DUF952)
EVM0012074	13.16	35.18	-1.42	5.00E-05	4.28E-04	Lipid transport and metabolism	CDP-alcohol phosphatidyltransferase

EVM0002814	94.54	252.69	-1.42	5.00E-05	4.28E-04	Intracellular trafficking, secretion, and vesicular transport	Peroxisomal membrane protein (Pex16)
EVM0008368	210.92	563.69	-1.42	5.00E-05	4.28E-04	Cell cycle control, cell division, chromosome partitioning	Double-stranded DNA-binding domain
EVM0006117	9.64	25.75	-1.42	5.00E-05	4.28E-04	General function prediction only	Aldo/keto reductase family
EVM0004386	19.36	51.68	-1.42	5.00E-05	4.28E-04	RNA processing and modification	DEAD/DEAH box helicase
EVM0001244	2.75	7.33	-1.42	5.00E-04	3.19E-03	--	Dyp-type peroxidase family
EVM0006132	85.94	229.13	-1.41	5.00E-05	4.28E-04	Intracellular trafficking, secretion, and vesicular transport	Peroxisomal biogenesis factor 11 (PEX11)
EVM0004396	14.12	37.63	-1.41	5.00E-05	4.28E-04	--	--
EVM0004027	1690.05	4500.14	-1.41	5.00E-05	4.28E-04	Translation, ribosomal structure and biogenesis	Ribosomal L27e protein family
EVM0006511	5.54	14.75	-1.41	5.00E-05	4.28E-04	General function prediction only	Glycosyl transferase family 90
EVM0006813	103.99	276.68	-1.41	5.00E-05	4.28E-04	General function prediction only	Sugar (and other) transporter
EVM0010558	13.17	35.02	-1.41	5.00E-05	4.28E-04	--	--

EVM0009958	11.49	30.55	-1.41	2.00E-04	1.46E-03	--	--
EVM0010741	5.71	15.19	-1.41	1.50E-04	1.14E-03	Secondary metabolites biosynthesis, transport and catabolism	short chain dehydrogenase
EVM0008554	63.07	167.59	-1.41	5.00E-05	4.28E-04	Cell cycle control, cell division, chromosome partitioning	WD domain, G-beta repeat
EVM0012170	20.27	53.76	-1.41	5.00E-05	4.28E-04	--	--
EVM0003576	5.25	13.91	-1.41	5.00E-05	4.28E-04	--	--
EVM0012212	15.10	40.03	-1.41	5.00E-05	4.28E-04	Chromatin structure and dynamics	SET domain
EVM0011226	128.12	339.63	-1.41	5.00E-05	4.28E-04	RNA processing and modification	Zinc-finger double-stranded RNA-binding
EVM0002708	20.52	54.38	-1.41	5.00E-05	4.28E-04	Function unknown	Ima1 N-terminal domain
EVM0006852	49.08	129.92	-1.40	5.00E-05	4.28E-04	--	Sen15 protein
EVM0002695	102.16	270.29	-1.40	5.00E-05	4.28E-04	--	SYF2 splicing factor
EVM0005762	187.42	495.53	-1.40	5.00E-05	4.28E-04	Posttranslational modification, protein turnover, chaperones	Thioredoxin

EVM0008274	4.84	12.79	-1.40	5.00E-05	4.28E-04	--	--
EVM0003688	9.03	23.87	-1.40	5.00E-05	4.28E-04	Cell cycle control, cell division, chromosome partitioning	Mad3/BUB1 homology region 1
EVM0000538	3.19	8.42	-1.40	8.50E-04	4.98E-03	--	Putative FMN-binding domain
EVM0007613	6.50	17.16	-1.40	5.00E-05	4.28E-04	Replication, recombination and repair	DNA polymerase beta thumb
EVM0000523	4.76	12.57	-1.40	5.00E-05	4.28E-04	--	Glycosyl hydrolase family 10
EVM0006536	0.74	1.95	-1.40	9.95E-03	3.74E-02	--	Pectate lyase
EVM0009651	4.91	12.97	-1.40	5.00E-05	4.28E-04	Energy production and conversion	FAD binding domain
EVM0007327	82.92	218.74	-1.40	5.00E-05	4.28E-04	Intracellular trafficking, secretion, and vesicular transport	Scd6-like Sm domain
EVM0011639	1293.03	3409.19	-1.40	1.00E-04	7.96E-04	--	--
EVM0006989	4.50	11.85	-1.40	5.00E-05	4.28E-04	Replication, recombination and repair	CDC45-like protein
EVM0011784	27.17	71.56	-1.40	5.00E-05	4.28E-04	Inorganic ion transport and metabolism	Sodium/hydrogen exchanger family
EVM0012251	0.71	1.87	-1.40	1.08E-02	3.98E-02	--	F-box-like

EVM0003967	102.09	268.77	-1.40	5.00E-05	4.28E-04	Function unknown	Mago binding
EVM0010500	30.57	80.45	-1.40	5.00E-05	4.28E-04	Transcription	RNA polymerase II transcription factor SIII (Elongin) subunit A
EVM0000666	56.27	148.08	-1.40	5.00E-05	4.28E-04	--	--
EVM0011858	89.70	235.89	-1.39	5.00E-05	4.28E-04	RNA processing and modification	Ribosomal protein L10
EVM0009027	71.92	189.06	-1.39	5.00E-05	4.28E-04	--	F-box-like
EVM0006644	72.01	189.23	-1.39	5.00E-05	4.28E-04	Function unknown	Mitochondrial large subunit ribosomal protein (Img2)
EVM0000030	11.00	28.89	-1.39	5.00E-05	4.28E-04	Inorganic ion transport and metabolism	Sodium/calcium exchanger protein
EVM0002455	369.64	970.99	-1.39	5.00E-05	4.28E-04	--	Phosphopantetheine attachment site
EVM0001135	10.15	26.67	-1.39	5.00E-05	4.28E-04	RNA processing and modification	DEAD/DEAH box helicase
EVM0009481	1.68	4.41	-1.39	2.75E-03	1.32E-02	--	GDSL-like Lipase/Acylhydrolase
EVM0003249	91.37	239.83	-1.39	5.00E-05	4.28E-04	--	--
EVM0003357	1906.89	5004.89	-1.39	1.85E-03	9.52E-03	--	--
EVM0006392	6.20	16.27	-1.39	5.00E-05	4.28E-04	--	Ferric reductase NAD binding domain

EVM0006496	1.22	3.20	-1.39	5.00E-05	4.28E-04	Replication, recombination and repair	DEAD/DEAH box helicase
EVM0007233	1.17	3.06	-1.39	6.50E-04	3.98E-03	--	--
EVM0004420	11.65	30.49	-1.39	5.00E-05	4.28E-04	--	--
EVM0010189	49.51	129.57	-1.39	5.00E-05	4.28E-04	--	Bacterial transferase hexapeptide (six repeats)
EVM0008576	4.30	11.24	-1.39	5.40E-03	2.29E-02	--	Ctf8
EVM0004161	85.37	223.38	-1.39	5.00E-05	4.28E-04	--	--
EVM0009145	2.56	6.70	-1.39	3.50E-04	2.36E-03	--	--
EVM0003989	11.13	29.11	-1.39	5.00E-05	4.28E-04	--	--
EVM0003535	8.46	22.11	-1.39	5.00E-05	4.28E-04	--	--
EVM0007915	59.79	156.32	-1.39	5.00E-05	4.28E-04	RNA processing and modification	Zinc-finger of C2H2 type
EVM0008531	57.51	150.27	-1.39	5.00E-05	4.28E-04	Posttranslational modification, protein turnover, chaperones	Oligosaccharyl transferase STT3 subunit
EVM0001263	6.02	15.73	-1.39	1.50E-04	1.14E-03	--	--

EVM0005384	49.87	130.32	-1.39	5.00E-05	4.28E-04	--	--
EVM0001995	5.93	15.48	-1.38	1.50E-04	1.14E-03	--	--
EVM0004684	13.91	36.29	-1.38	5.00E-05	4.28E-04	--	--
EVM0008642	26.47	69.08	-1.38	5.00E-05	4.28E-04	--	--
EVM0010605	717.39	1870.07	-1.38	5.00E-05	4.28E-04	Translation, ribosomal structure and biogenesis	Ribosomal protein L34e
EVM0007871	5.89	15.34	-1.38	2.50E-04	1.77E-03	--	--
EVM0005712	10.21	26.59	-1.38	5.00E-05	4.28E-04	--	--
EVM0005789	106.87	278.08	-1.38	5.00E-05	4.28E-04	--	--
EVM0003292	82.12	213.62	-1.38	5.00E-05	4.28E-04	--	Sel1 repeat
EVM0009292	6.56	17.07	-1.38	5.00E-05	4.28E-04	--	--
EVM0010081	86.48	224.95	-1.38	1.50E-04	1.14E-03	--	Protein of unknown function (DUF2462)
EVM0000857	1.43	3.73	-1.38	1.08E-02	3.97E-02	--	--
EVM0002350	35.46	92.21	-1.38	5.00E-05	4.28E-04	--	--

EVM0002618	47.12	122.51	-1.38	5.00E-05	4.28E-04	Translation, ribosomal structure and biogenesis	TruB family pseudouridylate synthase (N terminal domain)
EVM0000648	77.12	200.50	-1.38	5.00E-05	4.28E-04	Lipid transport and metabolism	Eukaryotic phosphomannomutase
EVM0007138	69.78	181.42	-1.38	5.00E-05	4.28E-04	--	--
EVM0006717	19.89	51.66	-1.38	5.00E-05	4.28E-04	--	--
EVM0002128	119.34	309.70	-1.38	5.00E-05	4.28E-04	Translation, ribosomal structure and biogenesis	Domain found in IF2B/IF5
EVM0010824	15.94	41.35	-1.38	5.00E-05	4.28E-04	General function prediction only	WD domain, G-beta repeat
EVM0003139	59.43	154.11	-1.37	5.00E-05	4.28E-04	Posttranslational modification, protein turnover, chaperones	Oligosaccharyltransferase 48 kDa subunit beta
EVM0009402	86.01	223.03	-1.37	5.00E-05	4.28E-04	Inorganic ion transport and metabolism	Got1/Sft2-like family
EVM0007355	9.65	25.03	-1.37	5.00E-05	4.28E-04	Coenzyme transport and metabolism	UbiA prenyltransferase family
EVM0003289	18.10	46.92	-1.37	5.00E-05	4.28E-04	Transcription	RNA polymerase III subunit Rpc25
EVM0003936	66.45	172.18	-1.37	5.00E-05	4.28E-04	General function prediction only	TPR repeat
EVM0002080	2.10	5.43	-1.37	6.00E-04	3.72E-03	--	--

EVM0010061	55.99	144.62	-1.37	5.00E-05	4.28E-04	--	SH3 domain
EVM0009042	176.61	455.27	-1.37	5.00E-05	4.28E-04	Posttranslational modification, protein turnover, chaperones	Prefoldin subunit
EVM0007136	26.61	68.48	-1.36	5.00E-05	4.28E-04	--	--
EVM0005405	7.31	18.81	-1.36	1.00E-04	7.96E-04	--	Pericentrin-AKAP-450 domain of centrosomal targeting protein
EVM0006430	7.78	20.00	-1.36	5.00E-05	4.28E-04	--	--
EVM0005831	15.11	38.86	-1.36	1.00E-04	7.96E-04	General function prediction only	3'-5' exonuclease
EVM0005080	34.83	89.47	-1.36	2.00E-04	1.46E-03	--	Fungal hydrophobin
EVM0007186	24.40	62.65	-1.36	5.00E-05	4.28E-04	--	--
EVM0010279	42.78	109.85	-1.36	5.00E-05	4.28E-04	--	Etoposide-induced protein 2.4 (EI24)
EVM0002055	13.11	33.66	-1.36	5.00E-05	4.28E-04	General function prediction only	Major Facilitator Superfamily
EVM0006257	192.83	494.59	-1.36	5.00E-05	4.28E-04	--	--
EVM0000375	91.86	235.54	-1.36	5.00E-05	4.28E-04	Lipid transport and metabolism	Fatty acid hydroxylase superfamily

EVM0001171	11.11	28.47	-1.36	1.00E-04	7.96E-04	Signal transduction mechanisms	ERG2 and Sigma1 receptor like protein
EVM0005349	2.52	6.47	-1.36	1.22E-02	4.39E-02	--	--
EVM0003339	3.40	8.71	-1.36	5.00E-05	4.28E-04	General function prediction only	Glycosyl transferase family 90
EVM0000938	18.63	47.64	-1.35	5.00E-05	4.28E-04	Posttranslational modification, protein turnover, chaperones	Cyclophilin type peptidyl-prolyl cis-trans isomerase/CLD
EVM0011919	27.40	70.06	-1.35	5.00E-05	4.28E-04	--	--
EVM0000602	1394.30	3560.81	-1.35	1.00E-04	7.96E-04	General function prediction only	Ras family
EVM0002709	3.94	10.06	-1.35	1.00E-04	7.96E-04	--	--
EVM0010195	362.36	925.32	-1.35	5.00E-05	4.28E-04	Translation, ribosomal structure and biogenesis	Ribosomal protein L14p/L23e
EVM0002424	25.20	64.34	-1.35	5.00E-05	4.28E-04	--	--
EVM0006608	19.45	49.65	-1.35	5.00E-05	4.28E-04	Carbohydrate transport and metabolism	ALG3 protein
EVM0010589	174.65	445.54	-1.35	5.00E-05	4.28E-04	--	bZIP transcription factor
EVM0006647	125.55	320.25	-1.35	5.00E-05	4.28E-04	Amino acid transport and metabolism	Aminotransferase class-V

EVM0006085	78.41	199.85	-1.35	5.00E-05	4.28E-04	--	--
EVM0011971	9.04	23.05	-1.35	5.00E-05	4.28E-04	Transcription	HMG (high mobility group) box
EVM0001928	22.10	56.31	-1.35	5.00E-05	4.28E-04	--	Ricin-type beta-trefoil lectin domain
EVM0004461	32.27	82.20	-1.35	5.00E-05	4.28E-04	--	Putative oxidoreductase C terminal
EVM0003807	4.04	10.29	-1.35	1.15E-03	6.41E-03	Amino acid transport and metabolism	--
EVM0011916	1048.96	2668.30	-1.35	5.00E-05	4.28E-04	--	Protein of unknown function (DUF2611)
EVM0001287	19.17	48.74	-1.35	5.00E-05	4.28E-04	--	--
EVM0005265	110.83	281.64	-1.35	1.00E-04	7.96E-04	--	--
EVM0005366	16.34	41.49	-1.34	5.00E-05	4.28E-04	Function unknown	pre-RNA processing PIH1/Nop17
EVM0001130	287.49	729.86	-1.34	2.00E-04	1.46E-03	--	Mitotic-spindle organizing gamma-tubulin ring associated
EVM0006992	22.34	56.72	-1.34	5.00E-05	4.28E-04	--	Cytochrome P450
EVM0005866	44.94	114.06	-1.34	3.65E-03	1.66E-02	Function unknown	Transcriptional regulator
EVM0004157	2.99	7.59	-1.34	2.00E-04	1.46E-03	--	--

EVM0004960	7.54	19.11	-1.34	2.00E-04	1.46E-03	--	Complex 1 protein (LYR family)
EVM0002867	34.40	87.22	-1.34	5.00E-05	4.28E-04	Lipid transport and metabolism	MBOAT, membrane-bound O-acyltransferase family
EVM0004379	235.10	595.80	-1.34	5.00E-05	4.28E-04	Intracellular trafficking, secretion, and vesicular transport	Synaptobrevin
EVM0003951	58.59	148.43	-1.34	5.00E-05	4.28E-04	Function unknown	RWD domain
EVM0005429	15.80	39.97	-1.34	5.00E-05	4.28E-04	Transcription	CCAAT-binding transcription factor (CBF-B/NF-YA) subunit B
EVM0011758	4.05	10.24	-1.34	9.00E-04	5.22E-03	General function prediction only	THUMP domain
EVM0009259	168.64	426.46	-1.34	5.00E-05	4.28E-04	--	--
EVM0011808	3.70	9.36	-1.34	1.95E-03	9.93E-03	--	Lysine-specific metallo-endopeptidase
EVM0009590	193.84	489.97	-1.34	5.00E-05	4.28E-04	RNA processing and modification	Zinc finger C-x8-C-x5-C-x3-H type (and similar)
EVM0011570	142.54	360.28	-1.34	5.00E-05	4.28E-04	--	--
EVM0002044	40.93	103.39	-1.34	5.00E-05	4.28E-04	Translation, ribosomal structure and biogenesis	Initiation factor 2 subunit family
EVM0007286	31.43	79.38	-1.34	5.00E-05	4.28E-04	--	--

EVM0003434	12.59	31.80	-1.34	1.50E-04	1.14E-03	--	Protein of unknown function (DUF1691)
EVM0003335	29.19	73.73	-1.34	5.00E-05	4.28E-04	--	Eukaryotic protein of unknown function (DUF1764)
EVM0007300	3.06	7.71	-1.34	4.50E-04	2.92E-03	--	--
EVM0012150	29.86	75.35	-1.34	5.00E-05	4.28E-04	--	--
EVM0007629	63.26	159.57	-1.33	5.00E-05	4.28E-04	General function prediction only	Protein of unknown function (DUF933)
EVM0003348	11.25	28.36	-1.33	4.00E-04	2.65E-03	Function unknown	PIG-P
EVM0007008	19.32	48.68	-1.33	5.00E-05	4.28E-04	--	F-box-like
EVM0011412	2.18	5.50	-1.33	1.03E-02	3.82E-02	--	--
EVM0005561	45.10	113.56	-1.33	5.00E-05	4.28E-04	Transcription	Transcription factor TFIIB repeat
EVM0004238	23.90	60.16	-1.33	5.00E-05	4.28E-04	--	SMI1 / KNR4 family (SUKH-1)
EVM0008438	19.61	49.36	-1.33	5.00E-05	4.28E-04	--	Apoptosis-antagonizing transcription factor, C-terminal
EVM0004387	36.97	93.03	-1.33	5.00E-05	4.28E-04	--	ASF1 like histone chaperone
EVM0005668	67.05	168.65	-1.33	5.00E-05	4.28E-04	--	NmrA-like family

EVM0007661	13.62	34.23	-1.33	5.00E-05	4.28E-04	--	C2 domain
EVM0000146	34.98	87.93	-1.33	5.00E-05	4.28E-04	--	--
EVM0008848	50.60	127.16	-1.33	5.00E-05	4.28E-04	Translation, ribosomal structure and biogenesis	Ribosomal protein S12/S23
EVM0001112	1.20	3.01	-1.33	2.15E-03	1.08E-02	--	Pectate lyase
EVM0005565	0.93	2.34	-1.33	1.44E-02	4.99E-02	--	Glycosyl hydrolases family 16
EVM0001903	209.50	525.92	-1.33	1.00E-04	7.96E-04	Translation, ribosomal structure and biogenesis	Eukaryotic translation initiation factor eIF2A
EVM0004046	127.46	319.86	-1.33	5.00E-05	4.28E-04	Amino acid transport and metabolism	Pyridoxal-dependent decarboxylase, pyridoxal binding domain
EVM0011101	12.25	30.74	-1.33	5.00E-05	4.28E-04	--	Bromodomain
EVM0001367	90.97	228.09	-1.33	5.00E-05	4.28E-04	--	--
EVM0002540	4.42	11.07	-1.33	2.50E-04	1.77E-03	--	Transferase family
EVM0004516	18.14	45.47	-1.33	5.00E-05	4.28E-04	--	--
EVM0004915	0.94	2.35	-1.32	4.95E-03	2.13E-02	Carbohydrate transport and metabolism	Alpha amylase, catalytic domain

EVM0011355	23.53	58.90	-1.32	5.00E-05	4.28E-04	--	Chalcone-flavanone isomerase
EVM0007524	5.23	13.09	-1.32	2.00E-04	1.46E-03	--	Domain of unknown function (DUF4451)
EVM0007162	18.75	46.92	-1.32	5.00E-05	4.28E-04	RNA processing and modification	Helicase associated domain (HA2)
EVM0004554	35.99	89.98	-1.32	5.00E-05	4.28E-04	Cytoskeleton	--
EVM0008702	191.94	479.50	-1.32	5.00E-05	4.28E-04	Intracellular trafficking, secretion, and vesicular transport	PRELI-like family
EVM0004324	184.83	461.70	-1.32	5.00E-05	4.28E-04	--	--
EVM0010825	2.08	5.20	-1.32	9.50E-04	5.46E-03	--	--
EVM0002863	4.64	11.58	-1.32	6.00E-04	3.72E-03	Transcription	Myb-like DNA-binding domain
EVM0011949	35.13	87.63	-1.32	5.00E-05	4.28E-04	--	Protein of unknown function (DUF3245)
EVM0007229	10.63	26.52	-1.32	1.00E-04	7.96E-04	--	Cupin-like domain
EVM0012004	3.53	8.81	-1.32	1.60E-03	8.44E-03	--	--
EVM0006504	28.67	71.42	-1.32	5.00E-05	4.28E-04	--	--

EVM0000228	21.06	52.37	-1.31	5.00E-05	4.28E-04	Function unknown	Di-sulfide bridge nucleocytoplasmic transport domain
EVM0001050	38.32	95.25	-1.31	5.00E-05	4.28E-04	--	CybS
EVM0000490	4.29	10.66	-1.31	4.50E-04	2.92E-03	--	Glycosyl hydrolase family 61
EVM0007494	231.85	575.41	-1.31	5.00E-05	4.28E-04	Posttranslational modification, protein turnover, chaperones	Glutaredoxin
EVM0009347	15.58	38.60	-1.31	5.00E-05	4.28E-04	--	WH2 motif
EVM0006427	202.36	501.12	-1.31	5.00E-05	4.28E-04	Amino acid transport and metabolism	Amino acid permease
EVM0006431	75.01	185.70	-1.31	5.00E-05	4.28E-04	--	--
EVM0011795	25.99	64.29	-1.31	1.00E-04	7.96E-04	--	--
EVM0003465	23.61	58.39	-1.31	5.00E-05	4.28E-04	--	--
EVM0007305	332.47	821.83	-1.31	5.00E-05	4.28E-04	RNA processing and modification	U1 zinc finger
EVM0002015	34.68	85.69	-1.31	5.00E-05	4.28E-04	Function unknown	--
EVM0005233	22.80	56.32	-1.30	5.00E-05	4.28E-04	Lipid transport and metabolism	Alpha/beta hydrolase family

EVM0011396	2.51	6.21	-1.30	6.00E-04	3.72E-03	--	Glycosyl hydrolase family 7
EVM0001699	131.77	325.14	-1.30	5.00E-05	4.28E-04	Translation, ribosomal structure and biogenesis	Ribosomal protein L13
EVM0005976	183.50	452.68	-1.30	5.00E-04	3.19E-03	General function prediction only	Sugar (and other) transporter
EVM0002013	1.54	3.80	-1.30	4.45E-03	1.96E-02	--	--
EVM0011522	12.40	30.56	-1.30	5.00E-05	4.28E-04	Defense mechanisms	NAD dependent epimerase/dehydratase family
EVM0000959	27.08	66.67	-1.30	5.00E-05	4.28E-04	Cytoskeleton	Dynamitin
EVM0000586	4.17	10.27	-1.30	1.10E-03	6.17E-03	--	Pheromone A receptor
EVM0003201	1634.68	4022.57	-1.30	5.00E-05	4.28E-04	--	Mitochondrial ATP synthase epsilon chain
EVM0003190	4.02	9.88	-1.30	7.50E-04	4.49E-03	--	Terpene synthase family, metal binding domain
EVM0008552	5.58	13.72	-1.30	4.00E-04	2.65E-03	--	--
EVM0011618	126.04	309.26	-1.29	5.00E-05	4.28E-04	--	--
EVM0007362	2.04	5.00	-1.29	1.20E-03	6.64E-03	--	Glycosyl hydrolases family 6
EVM0000068	90.16	221.08	-1.29	6.50E-04	3.98E-03	Function unknown	Uncharacterized conserved protein (DUF2346)

EVM0004607	7.39	18.09	-1.29	3.50E-04	2.36E-03	--	--
EVM0006677	12.11	29.64	-1.29	7.50E-04	4.49E-03	--	--
EVM0010899	44.21	108.20	-1.29	5.00E-05	4.28E-04	RNA processing and modification	Ribonuclease T2 family
EVM0007673	43.86	107.31	-1.29	5.00E-05	4.28E-04	Transcription	SWIB/MDM2 domain
EVM0010123	5.99	14.67	-1.29	5.00E-05	4.28E-04	Transcription	Myb-like DNA-binding domain
EVM0004016	161.79	395.80	-1.29	5.00E-05	4.28E-04	--	Protein of unknown function (DUF1275)
EVM0003444	3.52	8.62	-1.29	1.50E-04	1.14E-03	Carbohydrate transport and metabolism	Major Facilitator Superfamily
EVM0005581	7.63	18.67	-1.29	3.00E-04	2.07E-03	--	Lysine-specific metallo-endopeptidase
EVM0001790	725.79	1774.62	-1.29	5.00E-05	4.28E-04	Translation, ribosomal structure and biogenesis	Ribosomal protein L44
EVM0003382	17.28	42.24	-1.29	5.00E-05	4.28E-04	Translation, ribosomal structure and biogenesis	Dihydrouridine synthase (Dus)
EVM0006090	70.19	171.57	-1.29	5.00E-05	4.28E-04	--	Glycosyl transferase family 90
EVM0007699	116.48	284.65	-1.29	5.00E-05	4.28E-04	--	--
EVM0012102	18.41	44.99	-1.29	2.50E-04	1.77E-03	--	--

EVM0001924	9.20	22.44	-1.29	4.50E-04	2.92E-03	--	COMPASS (Complex proteins associated with Set1p) component shg1
EVM0000161	91.32	222.73	-1.29	5.00E-05	4.28E-04	Intracellular trafficking, secretion, and vesicular transport	Coatomer epsilon subunit
EVM0008044	15.30	37.30	-1.29	6.00E-04	3.72E-03	--	--
EVM0007655	23.46	57.19	-1.29	5.00E-05	4.28E-04	RNA processing and modification	DEAD/DEAH box helicase
EVM0002460	154.12	375.56	-1.29	1.18E-02	4.26E-02	General function prediction only	Proteolipid membrane potential modulator
EVM0005659	32.91	80.18	-1.28	5.00E-05	4.28E-04	--	--
EVM0009649	2.48	6.04	-1.28	5.55E-03	2.34E-02	--	--
EVM0001525	27.70	67.45	-1.28	5.00E-05	4.28E-04	--	--
EVM0002152	16.87	41.05	-1.28	1.00E-04	7.96E-04	--	--
EVM0009830	1.53	3.73	-1.28	4.55E-03	1.99E-02	--	--
EVM0007137	9.98	24.27	-1.28	2.00E-04	1.46E-03	--	Glycosyl hydrolase family 61
EVM0012095	7.67	18.66	-1.28	5.00E-04	3.19E-03	#N/A	#N/A

EVM0003241	37.16	90.35	-1.28	5.00E-05	4.28E-04	--	Helix-loop-helix DNA-binding domain
EVM0002387	98.09	238.42	-1.28	5.00E-05	4.28E-04	--	Coiled-coil domain containing protein (DUF2052)
EVM0003871	2.72	6.61	-1.28	1.00E-04	7.96E-04	--	Cytochrome P450
EVM0004125	0.56	1.35	-1.28	3.65E-03	1.66E-02	--	--
EVM0003678	5.28	12.82	-1.28	1.00E-04	7.96E-04	--	FAD binding domain
EVM0002245	30.43	73.87	-1.28	5.00E-05	4.28E-04	Function unknown	Leo1-like protein
EVM0002319	46.36	112.45	-1.28	5.00E-05	4.28E-04	--	Ribosome recycling factor
EVM0003843	104.89	254.35	-1.28	5.00E-05	4.28E-04	--	--
EVM0006953	2.76	6.69	-1.28	7.50E-04	4.49E-03	Cytoskeleton	Autophagy protein Atg8 ubiquitin like
EVM0004819	31.71	76.87	-1.28	5.00E-05	4.28E-04	Intracellular trafficking, secretion, and vesicular transport	Outer mitochondrial membrane transport complex protein
EVM0002675	15.78	38.25	-1.28	7.50E-04	4.49E-03	--	Glutathione-dependent formaldehyde-activating enzyme
EVM0010399	7.06	17.11	-1.28	8.50E-04	4.98E-03	--	Alpha/beta hydrolase family

EVM0008435	10.59	25.62	-1.27	1.00E-04	7.96E-04	--	--
EVM0007564	7.19	17.40	-1.27	5.00E-05	4.28E-04	Transcription	Fork head domain
EVM0007792	16.83	40.73	-1.27	5.00E-05	4.28E-04	--	--
EVM0010912	7.61	18.42	-1.27	3.00E-04	2.07E-03	--	--
EVM0000946	20.23	48.85	-1.27	5.00E-05	4.28E-04	Function unknown	Rgp1
EVM0009929	26.15	63.12	-1.27	5.00E-05	4.28E-04	Translation, ribosomal structure and biogenesis	39S mitochondrial ribosomal protein L46
EVM0010487	40.89	98.68	-1.27	5.00E-05	4.28E-04	--	Helix-loop-helix DNA-binding domain
EVM0009330	25.55	61.63	-1.27	5.00E-05	4.28E-04	--	NADH(P)-binding
EVM0009617	27.27	65.75	-1.27	5.00E-05	4.28E-04	--	Oxidoreductase FAD-binding domain
EVM0000564	220.48	531.46	-1.27	5.00E-05	4.28E-04	Amino acid transport and metabolism	Isocitrate/isopropylmalate dehydrogenase
EVM0000001	26.09	62.82	-1.27	5.00E-05	4.28E-04	Intracellular trafficking, secretion, and vesicular transport	ADP-ribosylation factor family
EVM0008547	2.67	6.42	-1.27	2.95E-03	1.40E-02	--	Common central domain of tyrosinase

EVM0012198	143.50	345.38	-1.27	5.00E-05	4.28E-04	--	Ribosomal protein L30p/L7e
EVM0006827	77.08	185.41	-1.27	1.00E-04	7.96E-04	--	--
EVM0011593	6.21	14.94	-1.27	4.00E-04	2.65E-03	Carbohydrate transport and metabolism	Major Facilitator Superfamily
EVM0007864	3.29	7.92	-1.27	1.50E-03	8.00E-03	--	--
EVM0006420	2.70	6.49	-1.26	3.35E-03	1.55E-02	--	--
EVM0007780	55.76	133.96	-1.26	5.00E-05	4.28E-04	--	--
EVM0003818	14.42	34.63	-1.26	5.00E-05	4.28E-04	RNA processing and modification	RNA recognition motif. (a.k.a. RRM, RBD, or RNP domain)
EVM0011517	20.19	48.48	-1.26	1.00E-04	7.96E-04	--	RNA12 protein
EVM0006062	64.55	154.93	-1.26	5.00E-05	4.28E-04	--	Mitochondrial ribosomal protein subunit
EVM0002500	31.08	74.60	-1.26	5.00E-05	4.28E-04	Posttranslational modification, protein turnover, chaperones	Ribophorin I
EVM0005187	2.21	5.30	-1.26	3.65E-03	1.66E-02	Cell cycle control, cell division, chromosome partitioning	Replication Fork Protection Component Swi3
EVM0011999	3.21	7.71	-1.26	5.05E-03	2.17E-02	--	--

EVM0006516	46.01	110.38	-1.26	5.00E-05	4.28E-04	--	Transcription factor Tfb2
EVM0006456	64.44	154.46	-1.26	2.60E-03	1.26E-02	General function prediction only	RNA recognition motif. (a.k.a. RRM, RBD, or RNP domain)
EVM0004843	17.52	41.95	-1.26	2.00E-04	1.46E-03	--	Ricin-type beta-trefoil lectin domain-like
EVM0003376	29.32	70.14	-1.26	1.00E-04	7.96E-04	--	--
EVM0003305	72.35	173.03	-1.26	5.00E-05	4.28E-04	Nucleotide transport and metabolism	Guanylate kinase
EVM0009226	35.67	85.31	-1.26	5.00E-05	4.28E-04	Carbohydrate transport and metabolism	Glycosyl hydrolase family 47
EVM0000332	92.46	220.97	-1.26	1.50E-04	1.14E-03	Lipid transport and metabolism	Thiolase, N-terminal domain
EVM0011937	3.44	8.22	-1.26	1.50E-04	1.14E-03	--	--
EVM0002882	25.59	61.13	-1.26	5.00E-05	4.28E-04	--	--
EVM0004115	1.44	3.43	-1.26	4.05E-03	1.81E-02	--	GDP-fucose protein O-fucosyltransferase
EVM0007189	172.54	411.62	-1.25	5.00E-05	4.28E-04	--	--
EVM0007966	193.40	461.04	-1.25	1.50E-04	1.14E-03	--	Na+ dependent nucleoside transporter C-terminus
EVM0010650	148.37	353.71	-1.25	5.00E-05	4.28E-04	--	Nnf1

EVM0011480	129.75	309.14	-1.25	5.00E-05	4.28E-04	--	Ion transport protein
EVM0008447	51.60	122.92	-1.25	5.00E-05	4.28E-04	--	--
EVM0011940	2.31	5.49	-1.25	8.40E-03	3.26E-02	General function prediction only	Sugar (and other) transporter
EVM0000324	81.26	193.47	-1.25	1.80E-03	9.29E-03	--	--
EVM0005479	46.75	111.26	-1.25	5.00E-05	4.28E-04	Function unknown	Secretory pathway protein Sec39
EVM0003416	22.70	54.03	-1.25	5.00E-05	4.28E-04	--	--
EVM0009266	18.45	43.89	-1.25	5.00E-05	4.28E-04	Cytoskeleton	Kinesin motor domain
EVM0005706	2.96	7.03	-1.25	4.00E-04	2.65E-03	--	F-box-like
EVM0009176	3.91	9.30	-1.25	1.70E-03	8.87E-03	--	--
EVM0008107	7.54	17.93	-1.25	6.00E-04	3.72E-03	--	--
EVM0008590	281.70	669.69	-1.25	3.50E-04	2.36E-03	Posttranslational modification, protein turnover, chaperones	TCP-1/cpn60 chaperonin family
EVM0010474	91.84	218.31	-1.25	5.00E-05	4.28E-04	--	--

EVM0011806	647.26	1538.05	-1.25	5.00E-05	4.28E-04	Translation, ribosomal structure and biogenesis	Ribosomal protein L18e/L15
EVM0010148	177.16	420.48	-1.25	5.00E-05	4.28E-04	--	--
EVM0012036	11.30	26.81	-1.25	1.55E-03	8.22E-03	--	--
EVM0008396	5.98	14.16	-1.24	5.45E-03	2.31E-02	General function prediction only	--
EVM0005806	141.17	334.52	-1.24	4.50E-04	2.92E-03	Carbohydrate transport and metabolism	Glycosyl hydrolase family 47
EVM0006187	3.53	8.37	-1.24	8.00E-04	4.73E-03	Transcription	HMG (high mobility group) box
EVM0011155	23.60	55.90	-1.24	1.50E-04	1.14E-03	--	PPR repeat family
EVM0012113	34.04	80.57	-1.24	2.00E-04	1.46E-03	--	--
EVM0003050	110.20	260.76	-1.24	5.00E-05	4.28E-04	Translation, ribosomal structure and biogenesis	Elongation factor Tu GTP binding domain
EVM0003533	25.29	59.83	-1.24	5.00E-05	4.28E-04	Posttranslational modification, protein turnover, chaperones	DnaJ domain
EVM0000366	5.23	12.37	-1.24	9.50E-04	5.46E-03	--	--
EVM0001597	23.10	54.63	-1.24	1.00E-04	7.96E-04	--	--

EVM0001395	29.87	70.61	-1.24	5.00E-05	4.28E-04	Nucleotide transport and metabolism	GDA1/CD39 (nucleoside phosphatase) family
EVM0009898	1641.79	3879.65	-1.24	5.00E-05	4.28E-04	Intracellular trafficking, secretion, and vesicular transport	SecE/Sec61-gamma subunits of protein translocation complex
EVM0000610	0.87	2.06	-1.24	9.25E-03	3.52E-02	Amino acid transport and metabolism	Amino acid permease
EVM0006686	191.57	452.51	-1.24	3.00E-04	2.07E-03	--	Protein of unknown function (DUF4449)
EVM0009421	30.45	71.82	-1.24	6.50E-04	3.98E-03	#N/A	#N/A
EVM0012070	1163.47	2743.83	-1.24	6.50E-04	3.98E-03	--	RNA recognition motif. (a.k.a. RRM, RBD, or RNP domain)
EVM0001951	5.79	13.66	-1.24	4.00E-03	1.79E-02	--	--
EVM0004854	205.35	484.06	-1.24	5.00E-05	4.28E-04	--	Mitochondrial import protein Pam17
EVM0010302	7.07	16.66	-1.24	1.85E-03	9.52E-03	--	--
EVM0008681	181.04	426.51	-1.24	5.00E-05	4.28E-04	--	Isochorismatase family
EVM0003613	91.43	215.38	-1.24	5.00E-05	4.28E-04	Energy production and conversion	Mitochondrial carrier protein
EVM0005121	190.59	448.80	-1.24	5.00E-05	4.28E-04	General function prediction only	Ras family

EVM0001485	448.77	1054.55	-1.23	1.00E-04	7.96E-04	General function prediction only	Ras family
EVM0006005	73.89	173.56	-1.23	8.10E-03	3.17E-02	Posttranslational modification, protein turnover, chaperones	Dolichol phosphate-mannose biosynthesis regulatory protein (DPM2)
EVM0010250	21.97	51.56	-1.23	5.00E-05	4.28E-04	General function prediction only	PPR repeat family
EVM0011110	1.39	3.26	-1.23	6.70E-03	2.72E-02	--	CAP-Gly domain
EVM0007529	11.83	27.75	-1.23	1.00E-04	7.96E-04	RNA processing and modification	Hinge domain of cleavage stimulation factor subunit 2
EVM0004251	17.77	41.69	-1.23	5.00E-05	4.28E-04	--	F-box-like
EVM0007280	57.24	134.18	-1.23	7.50E-04	4.49E-03	--	--
EVM0010330	6.51	15.26	-1.23	3.00E-04	2.07E-03	--	--
EVM0010387	98.34	230.34	-1.23	5.00E-05	4.28E-04	Transcription	Transcription initiation factor TFIID 23-30kDa subunit
EVM0010109	7.76	18.17	-1.23	1.50E-04	1.14E-03	Replication, recombination and repair	Eukaryotic and archaeal DNA primase, large subunit
EVM0006960	2.33	5.44	-1.23	1.39E-02	4.86E-02	--	--
EVM0007745	20.47	47.88	-1.23	5.00E-05	4.28E-04	--	F-box-like

EVM0011701	63.02	147.36	-1.23	3.00E-04	2.07E-03	--	Single-strand binding protein family
EVM0008483	19.55	45.71	-1.22	8.00E-04	4.73E-03	Function unknown	PRP38 family
EVM0008277	11.83	27.66	-1.22	2.00E-04	1.46E-03	--	Metallopeptidase family M24
EVM0011393	2.83	6.62	-1.22	1.50E-04	1.14E-03	--	Fungal specific transcription factor domain
EVM0003749	6.15	14.36	-1.22	6.50E-04	3.98E-03	Replication, recombination and repair	8-oxoguanine DNA glycosylase, N-terminal domain
EVM0006115	4.20	9.81	-1.22	3.80E-03	1.72E-02	Carbohydrate transport and metabolism	Glycosyl transferase family 8
EVM0007412	243.37	568.20	-1.22	2.00E-04	1.46E-03	--	Domain of unknown function (DUF202)
EVM0009748	19.92	46.49	-1.22	5.00E-05	4.28E-04	--	Endoplasmic reticulum-based factor for assembly of V-ATPase
EVM0002305	629.93	1469.34	-1.22	2.00E-04	1.46E-03	Chromatin structure and dynamics	Core histone H2A/H2B/H3/H4
EVM0001581	53.97	125.81	-1.22	5.00E-05	4.28E-04	RNA processing and modification	Putative methyltransferase
EVM0000627	20.02	46.66	-1.22	3.50E-04	2.36E-03	General function prediction only	Mpv17 / PMP22 family
EVM0006755	19.01	44.27	-1.22	5.00E-05	4.28E-04	--	--
EVM0010566	8.45	19.65	-1.22	5.00E-05	4.28E-04	Function unknown	WD domain, G-beta repeat

EVM0003921	76.14	177.04	-1.22	1.00E-04	7.96E-04	--	Mitochondrial ribosomal protein subunit L20
EVM0001542	29.86	69.42	-1.22	5.00E-05	4.28E-04	--	--
EVM0011278	478.09	1110.28	-1.22	5.00E-05	4.28E-04	--	--
EVM0007683	5.06	11.76	-1.22	1.50E-04	1.14E-03	Posttranslational modification, protein turnover, chaperones	--
EVM0006702	19.81	46.00	-1.22	5.00E-05	4.28E-04	--	--
EVM0006846	49.23	114.19	-1.21	5.00E-05	4.28E-04	RNA processing and modification	Prp18 domain
EVM0009325	72.98	169.25	-1.21	5.00E-05	4.28E-04	Amino acid transport and metabolism	Transmembrane amino acid transporter protein
EVM0000484	15.65	36.27	-1.21	1.50E-04	1.14E-03	--	--
EVM0003062	14.12	32.73	-1.21	1.50E-04	1.14E-03	--	--
EVM0008020	5.32	12.32	-1.21	1.90E-03	9.73E-03	--	--
EVM0012127	19.67	45.57	-1.21	5.00E-05	4.28E-04	--	PXA domain
EVM0007066	11.40	26.40	-1.21	2.00E-04	1.46E-03	--	--

EVM0009686	179.09	414.48	-1.21	5.00E-05	4.28E-04	Transcription	Spt4/RpoE2 zinc finger
EVM0000630	26.51	61.33	-1.21	2.50E-04	1.77E-03	--	--
EVM0007883	261.91	605.79	-1.21	1.50E-04	1.14E-03	--	--
EVM0004213	134.33	310.59	-1.21	5.00E-05	4.28E-04	Function unknown	Yip1 domain
EVM0010984	772.08	1785.06	-1.21	1.00E-04	7.96E-04	Transcription	NAC domain
EVM0008773	3639.22	8413.37	-1.21	4.55E-03	1.99E-02	--	--
EVM0003316	471.55	1089.32	-1.21	1.00E-04	7.96E-04	--	--
EVM0002007	25.92	59.87	-1.21	3.00E-04	2.07E-03	Energy production and conversion	Cytochrome b5-like Heme/Steroid binding domain
EVM0006616	1284.85	2965.34	-1.21	1.35E-03	7.33E-03	Energy production and conversion	Mitochondrial carrier protein
EVM0011921	512.60	1182.96	-1.21	1.50E-03	8.00E-03	Energy production and conversion	Aldehyde dehydrogenase family
EVM0011762	95.88	221.26	-1.21	5.00E-05	4.28E-04	General function prediction only	--
EVM0003534	5.29	12.21	-1.21	2.50E-04	1.77E-03	--	Major Facilitator Superfamily
EVM0004944	3.94	9.10	-1.21	8.45E-03	3.28E-02	--	F-box-like

EVM0009399	28.13	64.87	-1.21	5.00E-05	4.28E-04	--	Mediator complex subunit 25 von Willebrand factor type A
EVM0008425	1.46	3.37	-1.21	3.45E-03	1.59E-02	--	--
EVM0002309	44.24	101.98	-1.20	5.00E-05	4.28E-04	Energy production and conversion	NAD-dependent glycerol-3-phosphate dehydrogenase C-terminus
EVM0000499	25.93	59.76	-1.20	5.00E-05	4.28E-04	Translation, ribosomal structure and biogenesis	GatB/GatE catalytic domain
EVM0004351	8.45	19.45	-1.20	9.00E-04	5.22E-03	--	--
EVM0009324	86.36	198.79	-1.20	5.00E-05	4.28E-04	--	Transposase family tnp2
EVM0009762	2.84	6.54	-1.20	2.65E-03	1.28E-02	--	--
EVM0001479	1755.29	4040.24	-1.20	1.00E-04	7.96E-04	Translation, ribosomal structure and biogenesis	Ribosomal protein L18e/L15
EVM0011499	8.32	19.14	-1.20	3.50E-04	2.36E-03	--	--
EVM0007589	489.61	1124.30	-1.20	7.00E-04	4.23E-03	General function prediction only	Protein of unknown function (DUF1295)
EVM0010503	25.36	58.19	-1.20	5.00E-05	4.28E-04	Transcription	--
EVM0002352	54.98	126.12	-1.20	1.50E-04	1.14E-03	RNA processing and modification	DEAD/DEAH box helicase

EVM0011926	17.47	40.07	-1.20	1.00E-04	7.96E-04	--	--
EVM0011433	6.78	15.56	-1.20	1.00E-04	7.96E-04	--	F-box-like
EVM0001189	47.14	108.13	-1.20	5.00E-05	4.28E-04	Chromatin structure and dynamics	Histone deacetylase domain
EVM0011112	13.39	30.69	-1.20	6.00E-04	3.72E-03	--	--
EVM0006378	20.81	47.70	-1.20	5.00E-05	4.28E-04	Carbohydrate transport and metabolism	UDP-glucose:Glycoprotein Glucosyltransferase
EVM0008577	94.72	217.04	-1.20	5.00E-05	4.28E-04	Intracellular trafficking, secretion, and vesicular transport	Adaptor complexes medium subunit family
EVM0003908	94.74	217.04	-1.20	5.00E-05	4.28E-04	--	RNA recognition motif. (a.k.a. RRM, RBD, or RNP domain)
EVM0010265	35.36	80.93	-1.19	5.00E-05	4.28E-04	General function prediction only	Conserved hypothetical ATP binding protein
EVM0005768	463.72	1061.07	-1.19	3.00E-04	2.07E-03	--	Peroxidase
EVM0011816	171.01	390.79	-1.19	1.50E-04	1.14E-03	RNA processing and modification	LSM domain
EVM0000987	164.98	376.69	-1.19	5.00E-05	4.28E-04	--	--
EVM0001074	124.72	284.65	-1.19	9.00E-04	5.22E-03	Function unknown	Membrane transport protein

EVM0003745	6.42	14.65	-1.19	1.50E-04	1.14E-03	--	--
EVM0001611	2.69	6.14	-1.19	1.75E-03	9.08E-03	--	Glycosyl hydrolase family 61
EVM0000210	15.91	36.29	-1.19	5.00E-05	4.28E-04	--	--
EVM0009567	3.01	6.87	-1.19	5.50E-03	2.32E-02	Cell cycle control, cell division, chromosome partitioning	Protein kinase domain
EVM0003446	47.25	107.74	-1.19	1.00E-04	7.96E-04	--	Protein of unknown function (DUF2012)
EVM0006175	19.18	43.73	-1.19	5.00E-05	4.28E-04	Coenzyme transport and metabolism	Polyprenyl synthetase
EVM0006954	21.23	48.39	-1.19	5.00E-05	4.28E-04	--	Nucleoporin FG repeat region
EVM0010102	46.90	106.70	-1.19	5.00E-05	4.28E-04	--	--
EVM0005135	13.43	30.55	-1.19	3.00E-04	2.07E-03	Secondary metabolites biosynthesis, transport and catabolism	Cytochrome P450
EVM0008217	26.33	59.89	-1.19	1.00E-04	7.96E-04	--	Acetyltransferase (GNAT) family
EVM0004807	5.73	13.03	-1.19	2.50E-04	1.77E-03	Chromatin structure and dynamics	Histone deacetylase domain
EVM0008662	63.23	143.76	-1.18	5.00E-05	4.28E-04	--	Protein of unknown function (DUF2009)

EVM0003185	2.30	5.22	-1.18	7.50E-04	4.49E-03	--	--
EVM0003105	31.27	71.06	-1.18	7.00E-04	4.23E-03	Function unknown	FUN14 family
EVM0009431	42.85	97.32	-1.18	5.00E-05	4.28E-04	--	Mycolic acid cyclopropane synthetase
EVM0007845	4.03	9.16	-1.18	3.55E-03	1.62E-02	Cell cycle control, cell division, chromosome partitioning	Cyclin, N-terminal domain
EVM0006239	2.39	5.42	-1.18	1.15E-03	6.41E-03	--	UV-endonuclease UvdE
EVM0009865	35.64	80.86	-1.18	5.00E-05	4.28E-04	--	--
EVM0009040	15.53	35.24	-1.18	1.40E-03	7.55E-03	--	--
EVM0009965	6.10	13.82	-1.18	5.50E-04	3.46E-03	Defense mechanisms	NAD dependent epimerase/dehydratase family
EVM0007199	167.20	379.02	-1.18	2.00E-04	1.46E-03	General function prediction only	Yippee zinc-binding/DNA-binding /Mis18, centromere assembly
EVM0007735	0.78	1.78	-1.18	5.95E-03	2.47E-02	--	--
EVM0005126	4.20	9.52	-1.18	1.15E-03	6.41E-03	--	--
EVM0002405	4.82	10.92	-1.18	7.50E-03	2.98E-02	General function prediction only	Acetyltransferase (GNAT) family

EVM0009203	136.43	308.80	-1.18	1.00E-04	7.96E-04	--	OTU-like cysteine protease
EVM0005760	94.99	214.88	-1.18	1.00E-04	7.96E-04	Secondary metabolites biosynthesis, transport and catabolism	short chain dehydrogenase
EVM0005600	208.86	472.37	-1.18	3.35E-03	1.55E-02	--	Acetokinase family
EVM0009700	60.75	137.40	-1.18	5.00E-05	4.28E-04	--	NADH ubiquinone oxidoreductase subunit NDUFA12
EVM0006121	19.03	43.02	-1.18	2.00E-03	1.01E-02	--	FHA domain
EVM0010277	461.27	1042.14	-1.18	4.00E-04	2.65E-03	--	--
EVM0000800	162.68	367.38	-1.18	1.10E-03	6.17E-03	--	Protein of unknown function (DUF4449)
EVM0010961	1.68	3.79	-1.17	5.05E-03	2.17E-02	--	--
EVM0005918	62.55	141.09	-1.17	5.00E-05	4.28E-04	General function prediction only	PQ loop repeat
EVM0005903	6.61	14.92	-1.17	4.50E-04	2.92E-03	Transcription	RNA polymerase III RPC4
EVM0002113	8.73	19.70	-1.17	2.50E-04	1.77E-03	Replication, recombination and repair	Rad51
EVM0005046	1.38	3.10	-1.17	7.30E-03	2.91E-02	--	Glycosyl Hydrolase Family 88

EVM0006624	22.97	51.79	-1.17	1.00E-04	7.96E-04	Amino acid transport and metabolism	Pyridoxal-phosphate dependent enzyme
EVM0003706	13.20	29.74	-1.17	6.75E-03	2.74E-02	--	--
EVM0009875	7.82	17.62	-1.17	5.50E-04	3.46E-03	--	--
EVM0006106	28.39	63.97	-1.17	1.00E-04	7.96E-04	--	--
EVM0007277	708.74	1596.78	-1.17	2.50E-04	1.77E-03	--	--
EVM0001436	18.77	42.27	-1.17	3.00E-04	2.07E-03	--	WD domain, G-beta repeat
EVM0003299	63.29	142.58	-1.17	5.00E-05	4.28E-04	--	Protein kinase domain
EVM0009609	55.15	124.22	-1.17	2.50E-04	1.77E-03	Signal transduction mechanisms	RhoGEF domain
EVM0004559	5.57	12.56	-1.17	2.00E-04	1.46E-03	--	Fungalysin metallopeptidase (M36)
EVM0011169	74.39	167.55	-1.17	5.00E-05	4.28E-04	Posttranslational modification, protein turnover, chaperones	ATPase family associated with various cellular activities (AAA)
EVM0006316	7.60	17.11	-1.17	2.00E-04	1.46E-03	Function unknown	WW domain binding protein 11
EVM0010206	9.45	21.27	-1.17	2.00E-04	1.46E-03	--	--

EVM0011354	47.62	107.13	-1.17	1.00E-04	7.96E-04	Lipid transport and metabolism	Lipase (class 3)
EVM0001874	8.64	19.43	-1.17	2.50E-04	1.77E-03	Translation, ribosomal structure and biogenesis	tRNA synthetases class I (W and Y)
EVM0004039	172.18	387.23	-1.17	1.00E-04	7.96E-04	Energy production and conversion	--
EVM0011261	21.91	49.26	-1.17	5.00E-05	4.28E-04	General function prediction only	WD domain, G-beta repeat
EVM0000626	51.96	116.80	-1.17	1.00E-04	7.96E-04	--	Putative snoRNA binding domain
EVM0008219	228.57	513.77	-1.17	2.00E-04	1.46E-03	--	Hypothetical protein FLILHELTA
EVM0007891	193.38	434.67	-1.17	2.00E-04	1.46E-03	--	Amidase
EVM0002521	159.31	358.08	-1.17	1.00E-04	7.96E-04	Lipid transport and metabolism	Acyl-CoA dehydrogenase, C-terminal domain
EVM0001097	32.54	73.08	-1.17	2.00E-04	1.46E-03	--	Aminotransferase class IV
EVM0001993	66.87	150.11	-1.17	5.00E-05	4.28E-04	Coenzyme transport and metabolism	Coenzyme Q (ubiquinone) biosynthesis protein Coq4
EVM0001560	321.02	720.12	-1.17	2.00E-04	1.46E-03	--	Mitochondrial export protein Som1
EVM0005518	1.89	4.23	-1.17	8.15E-03	3.18E-02	--	non-haem dioxygenase in morphine synthesis N-terminal
EVM0002723	42.95	96.28	-1.16	2.10E-03	1.06E-02	--	--

EVM0010521	159.20	356.79	-1.16	1.05E-03	5.94E-03	--	--
EVM0011026	38.41	86.07	-1.16	5.00E-05	4.28E-04	Amino acid transport and metabolism	Semialdehyde dehydrogenase, dimerisation domain
EVM0009047	19.41	43.44	-1.16	5.00E-05	4.28E-04	General function prediction only	WD domain, G-beta repeat
EVM0008894	34.83	77.90	-1.16	7.50E-04	4.49E-03	--	Pterin 4 alpha carbinolamine dehydratase
EVM0009726	133.61	298.63	-1.16	5.00E-05	4.28E-04	--	--
EVM0007223	35.22	78.68	-1.16	5.00E-05	4.28E-04	Translation, ribosomal structure and biogenesis	Poly A polymerase head domain
EVM0000289	22.98	51.27	-1.16	5.00E-05	4.28E-04	RNA processing and modification	RNA recognition motif. (a.k.a. RRM, RBD, or RNP domain)
EVM0011516	21.39	47.70	-1.16	1.50E-04	1.14E-03	--	C2H2-type zinc finger
EVM0001684	30.54	68.10	-1.16	3.50E-04	2.36E-03	General function prediction only	AAA domain (dynein-related subfamily)
EVM0007438	50.54	112.64	-1.16	5.00E-05	4.28E-04	Cell cycle control, cell division, chromosome partitioning	WD domain, G-beta repeat
EVM0009155	55.38	123.42	-1.16	5.00E-05	4.28E-04	Function unknown	Ras-induced vulval development antagonist
EVM0009214	176.79	393.79	-1.16	5.00E-05	4.28E-04	General function prediction only	Casein kinase substrate phosphoprotein PP28

EVM0009100	21.34	47.51	-1.15	1.00E-04	7.96E-04	--	--
EVM0005312	1.72	3.82	-1.15	2.90E-03	1.38E-02	--	DNA polymerase subunit Cdc27
EVM0010571	41.70	92.77	-1.15	5.00E-05	4.28E-04	Transcription	Paf1
EVM0010669	90.91	202.04	-1.15	5.00E-05	4.28E-04	--	--
EVM0003558	65.17	144.81	-1.15	5.00E-05	4.28E-04	--	Methyltransferase domain
EVM0003112	3.23	7.17	-1.15	4.50E-03	1.97E-02	--	--
EVM0003799	3.74	8.30	-1.15	2.00E-03	1.01E-02	--	--
EVM0008502	4.78	10.60	-1.15	8.50E-04	4.98E-03	--	--
EVM0001957	7.42	16.44	-1.15	8.50E-04	4.98E-03	Secondary metabolites biosynthesis, transport and catabolism	Pyridine nucleotide-disulphide oxidoreductase
EVM0007632	58.12	128.70	-1.15	5.00E-05	4.28E-04	--	Protein kinase domain
EVM0010493	26.31	58.24	-1.15	3.15E-03	1.47E-02	--	--
EVM0004170	20.11	44.50	-1.15	5.00E-05	4.28E-04	--	--

EVM0011391	33.33	73.74	-1.15	5.00E-05	4.28E-04	Translation, ribosomal structure and biogenesis	Noc2p family
EVM0002501	75.90	167.78	-1.14	1.00E-04	7.96E-04	--	--
EVM0009315	268.08	592.01	-1.14	5.50E-04	3.46E-03	RNA processing and modification	LSM domain
EVM0011952	77.55	171.16	-1.14	5.00E-05	4.28E-04	Transcription	Helix-loop-helix DNA-binding domain
EVM0004966	3.63	8.02	-1.14	4.00E-04	2.65E-03	--	--
EVM0011892	2.19	4.82	-1.14	2.75E-03	1.32E-02	--	F-box-like
EVM0003518	23.99	52.89	-1.14	1.50E-04	1.14E-03	--	--
EVM0001765	29.00	63.90	-1.14	2.00E-04	1.46E-03	--	--
EVM0003552	57.99	127.78	-1.14	1.50E-04	1.14E-03	General function prediction only	Enoyl-(Acyl carrier protein) reductase
EVM0005877	892.77	1965.81	-1.14	1.00E-04	7.96E-04	Energy production and conversion	Ubiquinol-cytochrome C reductase complex 14kD subunit
EVM0002894	16.28	35.83	-1.14	1.50E-04	1.14E-03	Transcription	Protein kinase domain
EVM0002136	546.70	1202.24	-1.14	2.00E-04	1.46E-03	Translation, ribosomal structure and biogenesis	Ribosomal protein S4/S9 N-terminal domain
EVM0000700	56.78	124.84	-1.14	5.00E-05	4.28E-04	--	60Kd inner membrane protein

EVM0010096	49.43	108.67	-1.14	2.00E-04	1.46E-03	--	Cupin domain
EVM0007417	96.72	212.62	-1.14	1.50E-04	1.14E-03	Chromatin structure and dynamics	Core histone H2A/H2B/H3/H4
EVM0002318	52.27	114.87	-1.14	5.00E-05	4.28E-04	Replication, recombination and repair	Peptidase M76 family
EVM0001770	7.78	17.11	-1.14	7.00E-04	4.23E-03	--	Lactonase, 7-bladed beta-propeller
EVM0008669	28.59	62.80	-1.14	5.00E-05	4.28E-04	RNA processing and modification	DEAD/DEAH box helicase
EVM0011868	55.67	122.29	-1.14	2.50E-04	1.77E-03	Function unknown	Integral membrane protein DUF106
EVM0007207	4.79	10.51	-1.13	8.00E-04	4.73E-03	Replication, recombination and repair	Origin recognition complex (ORC) subunit 4 C-terminus
EVM0006493	100.54	220.63	-1.13	1.50E-04	1.14E-03	--	N-acetylglucosaminyl transferase component (Gpi1)
EVM0001573	1.69	3.71	-1.13	1.02E-02	3.80E-02	--	--
EVM0001926	45.17	98.96	-1.13	2.50E-04	1.77E-03	Posttranslational modification, protein turnover, chaperones	Dolichyl-phosphate-mannose-protein mannosyltransferase
EVM0011552	32.78	71.81	-1.13	4.00E-04	2.65E-03	Lipid transport and metabolism	Fatty acid desaturase
EVM0008804	68.54	150.05	-1.13	2.00E-04	1.46E-03	--	--

EVM0003425	7.89	17.26	-1.13	5.00E-05	4.28E-04	--	Formin Homology 2 Domain
EVM0009955	10.02	21.91	-1.13	2.50E-04	1.77E-03	--	--
EVM0005298	84.38	184.54	-1.13	2.00E-04	1.46E-03	--	--
EVM0002573	10.72	23.43	-1.13	4.50E-04	2.92E-03	--	RTA1 like protein
EVM0002730	27.40	59.91	-1.13	5.00E-05	4.28E-04	Signal transduction mechanisms	Leucine Rich repeats (2 copies)
EVM0001152	39.83	87.08	-1.13	3.50E-04	2.36E-03	General function prediction only	Mpv17 / PMP22 family
EVM0005703	24.18	52.82	-1.13	1.50E-04	1.14E-03	Function unknown	Utp14 protein
EVM0004423	15.62	34.09	-1.13	2.05E-03	1.03E-02	Posttranslational modification, protein turnover, chaperones	Peptidase S24-like
EVM0002797	108.18	236.09	-1.13	5.00E-05	4.28E-04	--	--
EVM0011621	49.66	108.33	-1.13	1.00E-04	7.96E-04	Function unknown	Low temperature viability protein
EVM0002975	293.69	640.47	-1.12	2.20E-03	1.10E-02	--	Putative stress-responsive nuclear envelope protein
EVM0003571	17.52	38.18	-1.12	3.50E-04	2.36E-03	--	Zinc finger-containing protein

EVM0003411	527.29	1148.21	-1.12	8.00E-04	4.73E-03	--	Zinc-binding
EVM0002703	28.70	62.50	-1.12	2.00E-04	1.46E-03	--	Domain of unknown function DUF221
EVM0002459	10.59	23.06	-1.12	5.00E-05	4.28E-04	Cell cycle control, cell division, chromosome partitioning	N terminus of Rad21 / Rec8 like protein
EVM0010830	257.82	561.11	-1.12	6.45E-03	2.64E-02	--	--
EVM0004694	8.86	19.26	-1.12	1.00E-03	5.70E-03	--	--
EVM0006639	87.80	190.93	-1.12	5.00E-05	4.28E-04	--	BRCA1 C Terminus (BRCT) domain
EVM0010286	4.53	9.85	-1.12	6.50E-04	3.98E-03	--	Ferric reductase like transmembrane component
EVM0002557	15.27	33.20	-1.12	1.50E-04	1.14E-03	Replication, recombination and repair	--
EVM0011493	21.66	47.08	-1.12	9.50E-04	5.46E-03	--	--
EVM0011176	112.12	243.57	-1.12	5.00E-05	4.28E-04	Translation, ribosomal structure and biogenesis	Ribosomal protein L6
EVM0010559	22.13	48.06	-1.12	6.00E-04	3.72E-03	General function prediction only	Mitochondrial PGP phosphatase
EVM0004323	12.70	27.57	-1.12	2.50E-04	1.77E-03	Signal transduction mechanisms	Protein kinase domain

EVM0004600	65.38	141.95	-1.12	5.00E-05	4.28E-04	General function prediction only	Eukaryotic protein of unknown function (DUF846)
EVM0000753	46.09	100.03	-1.12	1.50E-04	1.14E-03	Amino acid transport and metabolism	Amino acid permease
EVM0003296	183.26	397.72	-1.12	2.25E-03	1.12E-02	General function prediction only	Domain of unknown function DUF221
EVM0011479	137.22	297.52	-1.12	5.00E-05	4.28E-04	Posttranslational modification, protein turnover, chaperones	PI31 proteasome regulator N-terminal
EVM0011366	14.13	30.62	-1.12	5.00E-05	4.28E-04	--	SDA1
EVM0010660	20.77	45.02	-1.12	8.30E-03	3.23E-02	--	--
EVM0000793	122.04	264.44	-1.12	5.00E-05	4.28E-04	--	HPP family
EVM0002980	3.39	7.35	-1.12	2.10E-03	1.06E-02	Amino acid transport and metabolism	Amino acid kinase family
EVM0008212	17.56	38.01	-1.11	1.00E-04	7.96E-04	Translation, ribosomal structure and biogenesis	Glucose inhibited division protein A
EVM0011059	69.87	151.25	-1.11	2.50E-04	1.77E-03	--	--
EVM0001565	2.43	5.27	-1.11	2.25E-03	1.12E-02	--	--
EVM0004976	15.89	34.39	-1.11	1.00E-04	7.96E-04	Replication, recombination and repair	ERCC4 domain

EVM0001742	40.62	87.87	-1.11	1.50E-04	1.14E-03	General function prediction only	Alpha/beta hydrolase family
EVM0007593	110.22	237.91	-1.11	2.00E-04	1.46E-03	RNA processing and modification	Zinc knuckle
EVM0001412	3.95	8.53	-1.11	1.04E-02	3.87E-02	--	--
EVM0008609	20.25	43.68	-1.11	6.75E-03	2.74E-02	General function prediction only	Ankyrin repeats (3 copies)
EVM0006784	27.47	59.25	-1.11	4.50E-04	2.92E-03	General function prediction only	Zinc finger, C2H2 type
EVM0000063	249.99	539.17	-1.11	2.00E-04	1.46E-03	Nucleotide transport and metabolism	Adenylate kinase
EVM0004057	244.65	527.48	-1.11	5.00E-05	4.28E-04	--	Ser-Thr-rich glycosyl-phosphatidyl-inositol-anchored membrane family
EVM0005811	2.59	5.58	-1.11	1.01E-02	3.78E-02	--	--
EVM0006665	61.41	132.34	-1.11	5.00E-05	4.28E-04	Lipid transport and metabolism	Thioesterase-like superfamily
EVM0001158	36.87	79.44	-1.11	1.00E-04	7.96E-04	--	--
EVM0009246	28.67	61.76	-1.11	7.50E-04	4.49E-03	--	--
EVM0003806	20.17	43.43	-1.11	8.50E-04	4.98E-03	--	--

EVM0002692	1.62	3.49	-1.11	7.45E-03	2.96E-02	Secondary metabolites biosynthesis, transport and catabolism	Cytochrome P450
EVM0009392	150.82	324.50	-1.11	2.55E-03	1.24E-02	Posttranslational modification, protein turnover, chaperones	Sec63 Brl domain
EVM0008877	34.99	75.27	-1.10	5.00E-05	4.28E-04	--	--
EVM0001621	3.11	6.68	-1.10	1.09E-02	4.01E-02	--	Glycosyl hydrolases family 11
EVM0002403	297.77	640.24	-1.10	1.50E-04	1.14E-03	Posttranslational modification, protein turnover, chaperones	Ubiquitin-conjugating enzyme
EVM0005840	29.96	64.41	-1.10	2.25E-03	1.12E-02	--	Sen15 protein
EVM0000931	30.20	64.93	-1.10	1.40E-03	7.55E-03	--	Protein of unknown function (DUF3128)
EVM0000151	301.75	648.44	-1.10	2.00E-03	1.01E-02	Lipid transport and metabolism	Cytochrome b5-like Heme/Steroid binding domain
EVM0007815	184.20	395.66	-1.10	1.70E-03	8.87E-03	Inorganic ion transport and metabolism	E1-E2 ATPase
EVM0005388	244.08	524.01	-1.10	1.50E-04	1.14E-03	--	--
EVM0008721	2.30	4.94	-1.10	6.00E-03	2.49E-02	Lipid transport and metabolism	Hydroxymethylglutaryl-coenzyme A synthase N terminal

EVM0001193	6.56	14.09	-1.10	4.15E-03	1.85E-02	--	--
EVM0002058	12.34	26.48	-1.10	1.10E-03	6.17E-03	--	--
EVM0002564	25.81	55.38	-1.10	5.00E-05	4.28E-04	--	--
EVM0006853	125.74	269.72	-1.10	1.50E-04	1.14E-03	Energy production and conversion	ATP synthase (C/AC39) subunit
EVM0009366	10.03	21.50	-1.10	3.00E-04	2.07E-03	--	--
EVM0010530	95.47	204.71	-1.10	2.50E-04	1.77E-03	--	--
EVM0010153	56.94	122.09	-1.10	1.00E-04	7.96E-04	Lipid transport and metabolism	--
EVM0009451	8.89	19.07	-1.10	1.00E-04	7.96E-04	--	NYN domain
EVM0000516	26.15	56.04	-1.10	6.50E-04	3.98E-03	--	--
EVM0007521	44.53	95.41	-1.10	5.00E-04	3.19E-03	--	Cytochrome P450
EVM0005477	25.73	55.12	-1.10	1.00E-04	7.96E-04	Secondary metabolites biosynthesis, transport and catabolism	short chain dehydrogenase
EVM0005021	1.52	3.25	-1.10	2.65E-03	1.28E-02	--	--

EVM0003735	40.07	85.85	-1.10	5.00E-05	4.28E-04	Function unknown	Rtf2 RING-finger
EVM0000021	234.83	502.93	-1.10	3.00E-04	2.07E-03	Intracellular trafficking, secretion, and vesicular transport	Syntaxin-like protein
EVM0007756	154.83	331.34	-1.10	3.50E-04	2.36E-03	--	CybS
EVM0003169	81.28	173.93	-1.10	5.00E-05	4.28E-04	--	--
EVM0003937	26.13	55.90	-1.10	2.00E-04	1.46E-03	RNA processing and modification	RNA pseudouridylate synthase
EVM0001571	15.36	32.86	-1.10	1.25E-03	6.87E-03	--	--
EVM0006361	44.73	95.63	-1.10	4.00E-04	2.65E-03	--	--
EVM0004577	24.64	52.64	-1.10	5.00E-05	4.28E-04	Cell cycle control, cell division, chromosome partitioning	Cid1 family poly A polymerase
EVM0009215	300.57	641.79	-1.09	3.00E-04	2.07E-03	Lipid transport and metabolism	GNS1/SUR4 family
EVM0000218	57.90	123.57	-1.09	8.00E-04	4.73E-03	--	50S ribosome-binding GTPase
EVM0002193	35.51	75.75	-1.09	1.00E-04	7.96E-04	--	--
EVM0004159	8.08	17.22	-1.09	1.00E-03	5.70E-03	--	--

EVM0003070	33.65	71.68	-1.09	2.00E-04	1.46E-03	RNA processing and modification	DEAD/DEAH box helicase
EVM0009354	28.60	60.93	-1.09	1.50E-04	1.14E-03	Function unknown	Coiled-coil domain-containing protein 55 (DUF2040)
EVM0002249	15.30	32.58	-1.09	3.50E-04	2.36E-03	Function unknown	NUC153 domain
EVM0005876	9.09	19.33	-1.09	1.15E-03	6.41E-03	--	--
EVM0003681	219.25	466.04	-1.09	5.00E-05	4.28E-04	Intracellular trafficking, secretion, and vesicular transport	Tim17/Tim22/Tim23/Pmp24 family
EVM0008778	8.71	18.52	-1.09	4.50E-04	2.92E-03	--	--
EVM0005633	36.91	78.43	-1.09	5.00E-04	3.19E-03	--	--
EVM0007971	73.11	155.34	-1.09	1.00E-04	7.96E-04	Carbohydrate transport and metabolism	Glycolipid 2-alpha-mannosyltransferase
EVM0003061	25.29	53.71	-1.09	2.00E-04	1.46E-03	--	--
EVM0004955	743.05	1577.63	-1.09	2.50E-04	1.77E-03	Translation, ribosomal structure and biogenesis	Ribosomal protein S10p/S20e
EVM0004758	24.20	51.37	-1.09	3.50E-04	2.36E-03	--	Tryptophan halogenase
EVM0004250	27.66	58.70	-1.09	3.50E-04	2.36E-03	--	--

EVM0009458	2.35	4.99	-1.08	3.50E-03	1.61E-02	--	--
EVM0008302	49.03	103.98	-1.08	3.00E-04	2.07E-03	--	--
EVM0006756	84.30	178.73	-1.08	1.45E-03	7.77E-03	Posttranslational modification, protein turnover, chaperones	Cytochrome c oxidase assembly protein PET191
EVM0004495	251.97	534.01	-1.08	4.00E-04	2.65E-03	Translation, ribosomal structure and biogenesis	Ribosomal protein L7Ae/L30e/S12e/Gadd45 family
EVM0009227	11.35	24.04	-1.08	4.50E-04	2.92E-03	--	CrcB-like protein
EVM0005377	7.43	15.72	-1.08	1.00E-03	5.70E-03	--	Cellulase (glycosyl hydrolase family 5)
EVM0001550	134.28	284.16	-1.08	2.50E-04	1.77E-03	--	--
EVM0009311	129.49	273.89	-1.08	4.00E-04	2.65E-03	--	Etoposide-induced protein 2.4 (EI24)
EVM0002370	22.87	48.32	-1.08	1.50E-04	1.14E-03	RNA processing and modification	WD domain, G-beta repeat
EVM0001463	14.31	30.22	-1.08	5.00E-05	4.28E-04	Signal transduction mechanisms	Protein kinase domain
EVM0010742	38.24	80.61	-1.08	5.00E-04	3.19E-03	Posttranslational modification, protein turnover, chaperones	ATP10 protein
EVM0002326	8.51	17.93	-1.08	2.30E-03	1.14E-02	--	--

EVM0007812	81.09	170.80	-1.07	4.00E-04	2.65E-03	Function unknown	N-terminal glutamine amidase
EVM0002494	68.64	144.50	-1.07	5.00E-05	4.28E-04	--	Peroxisomal membrane anchor protein (Pex14p) conserved region
EVM0007242	6.31	13.29	-1.07	5.50E-04	3.46E-03	--	--
EVM0004550	27.78	58.48	-1.07	3.00E-04	2.07E-03	--	--
EVM0004745	23.13	48.69	-1.07	4.00E-04	2.65E-03	--	--
EVM0004173	66.71	140.40	-1.07	3.35E-03	1.55E-02	--	--
EVM0011339	161.57	339.97	-1.07	1.00E-04	7.96E-04	Translation, ribosomal structure and biogenesis	SMP-30/Gluconolaconase/LRE-like region
EVM0000882	72.56	152.68	-1.07	2.50E-04	1.77E-03	RNA processing and modification	Nucleotide hydrolase
EVM0001975	139.55	293.55	-1.07	5.00E-05	4.28E-04	--	--
EVM0001093	4.56	9.59	-1.07	3.30E-03	1.53E-02	--	Sodium Bile acid symporter family
EVM0007190	12.62	26.53	-1.07	3.00E-04	2.07E-03	--	50S ribosome-binding GTPase
EVM0006612	3.70	7.78	-1.07	2.35E-03	1.16E-02	--	--

EVM0001536	69.72	146.51	-1.07	3.50E-04	2.36E-03	--	PhoD-like phosphatase
EVM0000148	240.74	505.78	-1.07	4.50E-04	2.92E-03	General function prediction only	Zinc-binding dehydrogenase
EVM0007202	2.90	6.10	-1.07	6.20E-03	2.56E-02	--	non-haem dioxygenase in morphine synthesis N-terminal
EVM0004503	4.28	8.99	-1.07	5.30E-03	2.26E-02	--	--
EVM0008762	140.21	294.41	-1.07	3.50E-04	2.36E-03	Carbohydrate transport and metabolism	Glycolipid 2-alpha-mannosyltransferase
EVM0012060	4.58	9.61	-1.07	1.00E-03	5.70E-03	--	--
EVM0011721	103.84	217.98	-1.07	5.00E-05	4.28E-04	Translation, ribosomal structure and biogenesis	Ribosomal L28 family
EVM0000088	319.90	671.49	-1.07	6.55E-03	2.67E-02	--	--
EVM0004907	3.66	7.68	-1.07	6.45E-03	2.64E-02	RNA processing and modification	RNA recognition motif. (a.k.a. RRM, RBD, or RNP domain)
EVM0009940	3.45	7.24	-1.07	1.90E-03	9.73E-03	--	Thiamine pyrophosphate enzyme, N-terminal TPP binding domain
EVM0009631	54.87	114.97	-1.07	3.00E-04	2.07E-03	Coenzyme transport and metabolism	ubiE/COQ5 methyltransferase family
EVM0011592	106.88	223.87	-1.07	5.50E-04	3.46E-03	--	--
EVM0008388	17.36	36.36	-1.07	5.50E-04	3.46E-03	--	--

EVM0009128	1.37	2.87	-1.06	6.70E-03	2.72E-02	--	--
EVM0001528	39.61	82.83	-1.06	2.00E-04	1.46E-03	RNA processing and modification	DEAD/DEAH box helicase
EVM0007700	86.53	180.94	-1.06	2.65E-03	1.28E-02	--	--
EVM0007785	202.62	423.46	-1.06	2.50E-04	1.77E-03	Intracellular trafficking, secretion, and vesicular transport	Mitochondrial import receptor subunit Tom22
EVM0007252	54.57	114.04	-1.06	1.00E-04	7.96E-04	Secondary metabolites biosynthesis, transport and catabolism	Pyridine nucleotide-disulphide oxidoreductase
EVM0000562	38.47	80.39	-1.06	2.00E-04	1.46E-03	Intracellular trafficking, secretion, and vesicular transport	Signal recognition particle, alpha subunit, N-terminal
EVM0003359	177.43	370.28	-1.06	3.50E-04	2.36E-03	Translation, ribosomal structure and biogenesis	JAB1/Mov34/MPN/PAD-1 ubiquitin protease
EVM0002993	13.20	27.54	-1.06	3.00E-04	2.07E-03	--	Spb1 C-terminal domain
EVM0010902	422.35	881.11	-1.06	7.00E-04	4.23E-03	--	Mitochondrial ATPase inhibitor, IATP
EVM0011210	289.65	604.24	-1.06	1.50E-04	1.14E-03	--	DASH complex subunit Dad2
EVM0008211	13.55	28.25	-1.06	2.00E-04	1.46E-03	Signal transduction mechanisms	Protein tyrosine kinase

EVM0002986	19.73	41.15	-1.06	3.00E-04	2.07E-03	Cytoskeleton	Kinesin motor domain
EVM0007474	45.34	94.53	-1.06	1.50E-04	1.14E-03	Transcription	HCNGP-like protein
EVM0010516	1.49	3.11	-1.06	1.35E-02	4.74E-02	--	--
EVM0005390	52.54	109.47	-1.06	1.00E-04	7.96E-04	Lipid transport and metabolism	short chain dehydrogenase
EVM0004273	31.41	65.42	-1.06	3.50E-04	2.36E-03	Signal transduction mechanisms	Mitochondrial branched-chain alpha-ketoacid dehydrogenase kinase
EVM0009487	32.59	67.87	-1.06	1.50E-04	1.14E-03	Function unknown	Sad1 / UNC-like C-terminal
EVM0000714	351.30	731.50	-1.06	6.00E-04	3.72E-03	Translation, ribosomal structure and biogenesis	Ribosomal protein S7p/S5e
EVM0007025	207.07	431.17	-1.06	3.00E-04	2.07E-03	General function prediction only	RNA recognition motif. (a.k.a. RRM, RBD, or RNP domain)
EVM0011851	7.57	15.76	-1.06	6.80E-03	2.75E-02	--	Domain of unknown function (DUF3328)
EVM0002297	271.37	564.48	-1.06	1.55E-03	8.22E-03	--	--
EVM0006870	228.21	474.42	-1.06	4.20E-03	1.87E-02	Function unknown	Cytochrome c oxidase biogenesis protein Cmc1 like
EVM0002558	11.62	24.16	-1.06	2.00E-04	1.46E-03	Cell cycle control, cell division, chromosome partitioning	Protein kinase domain

EVM0009546	29.78	61.87	-1.05	3.00E-04	2.07E-03	--	Fungal specific transcription factor domain
EVM0001784	45.00	93.44	-1.05	3.50E-04	2.36E-03	Transcription	RNA polymerase Rpb4
EVM0006523	8.84	18.35	-1.05	3.15E-03	1.47E-02	--	--
EVM0003982	8.15	16.90	-1.05	1.90E-03	9.73E-03	RNA processing and modification	AAA domain
EVM0011554	189.80	393.58	-1.05	2.50E-04	1.77E-03	--	EF-hand domain
EVM0002786	1521.28	3153.75	-1.05	8.20E-03	3.20E-02	--	--
EVM0002271	14.49	30.03	-1.05	4.50E-04	2.92E-03	Function unknown	Gar1/Naf1 RNA binding region
EVM0011920	56.59	117.22	-1.05	1.50E-04	1.14E-03	Translation, ribosomal structure and biogenesis	Ribosomal protein L3
EVM0006320	244.06	505.34	-1.05	3.50E-04	2.36E-03	--	Protein kinase domain
EVM0007154	37.87	78.40	-1.05	4.50E-04	2.92E-03	--	--
EVM0007802	185.13	383.23	-1.05	1.00E-04	7.96E-04	--	Protein of unknown function (DUF952)
EVM0002598	5.43	11.24	-1.05	8.00E-04	4.73E-03	--	Protein kinase domain
EVM0000354	882.53	1825.59	-1.05	1.50E-04	1.14E-03	Translation, ribosomal structure and biogenesis	Ribosomal L29 protein

EVM0009575	58.77	121.56	-1.05	1.50E-04	1.14E-03	Lipid transport and metabolism	Acyl-CoA dehydrogenase, C-terminal domain
EVM0003529	4.22	8.73	-1.05	3.05E-03	1.44E-02	--	--
EVM0012197	52.21	107.93	-1.05	1.50E-04	1.14E-03	Translation, ribosomal structure and biogenesis	DKCLD (NUC011) domain
EVM0001437	21.06	43.52	-1.05	4.50E-04	2.92E-03	RNA processing and modification	pre-mRNA processing factor 3 (PRP3)
EVM0007184	40.39	83.45	-1.05	4.50E-04	2.92E-03	Function unknown	YjeF-related protein N-terminus
EVM0002265	53.19	109.87	-1.05	2.00E-04	1.46E-03	--	Probable molybdopterin binding domain
EVM0005245	22.88	47.20	-1.05	1.50E-04	1.14E-03	--	Ubiquitin-2 like Rad60 SUMO-like
EVM0000145	60.92	125.65	-1.04	3.50E-04	2.36E-03	--	Glycosyl hydrolase family 71
EVM0002946	10.09	20.80	-1.04	1.70E-03	8.87E-03	Carbohydrate transport and metabolism	Glycolipid 2-alpha-mannosyltransferase
EVM0006765	25.94	53.44	-1.04	6.00E-04	3.72E-03	Energy production and conversion	Mitochondrial carrier protein
EVM0008033	88.91	183.04	-1.04	1.00E-04	7.96E-04	Energy production and conversion	Biotin-requiring enzyme
EVM0008323	15.09	31.07	-1.04	2.50E-04	1.77E-03	--	--
EVM0003158	1079.23	2221.35	-1.04	2.30E-03	1.14E-02	--	--

EVM0000823	9.77	20.12	-1.04	8.00E-04	4.73E-03	General function prediction only	Surface antigen
EVM0005164	132.24	271.93	-1.04	2.00E-04	1.46E-03	Function unknown	--
EVM0005141	52.00	106.90	-1.04	5.00E-05	4.28E-04	General function prediction only	--
EVM0004008	139.12	285.98	-1.04	9.50E-04	5.46E-03	Lipid transport and metabolism	Choline/Carnitine o-acyltransferase
EVM0005539	3.60	7.39	-1.04	3.85E-03	1.74E-02	Posttranslational modification, protein turnover, chaperones	BCS1 N terminal
EVM0002294	7.48	15.37	-1.04	2.10E-03	1.06E-02	General function prediction only	Alpha/beta hydrolase family
EVM0012232	1.60	3.29	-1.04	1.39E-02	4.87E-02	--	--
EVM0012208	74.49	153.01	-1.04	9.00E-04	5.22E-03	--	--
EVM0006068	5.92	12.16	-1.04	2.50E-04	1.77E-03	--	bZIP transcription factor
EVM0012144	37.54	77.05	-1.04	3.00E-04	2.07E-03	Function unknown	Eukaryotic protein of unknown function (DUF829)
EVM0001478	4.77	9.80	-1.04	1.50E-03	8.00E-03	--	Pro-kumamolisin, activation domain
EVM0009338	26.96	55.31	-1.04	3.50E-04	2.36E-03	Carbohydrate transport and metabolism	FGGY family of carbohydrate kinases, C-terminal domain

EVM0001461	20.90	42.84	-1.04	4.50E-04	2.92E-03	Function unknown	WD domain, G-beta repeat
EVM0011812	2.67	5.48	-1.04	6.70E-03	2.72E-02	--	Glycosyl hydrolase family 71
EVM0007209	78.25	160.32	-1.03	1.50E-04	1.14E-03	--	Universal stress protein family
EVM0002920	28.21	57.80	-1.03	6.50E-04	3.98E-03	Intracellular trafficking, secretion, and vesicular transport	ADP-ribosylation factor family
EVM0004775	21.60	44.25	-1.03	1.30E-03	7.10E-03	--	Conidiation protein 6
EVM0010086	18.21	37.24	-1.03	3.50E-04	2.36E-03	Signal transduction mechanisms	Rab-GTPase-TBC domain
EVM0000902	23.98	49.04	-1.03	4.00E-04	2.65E-03	Posttranslational modification, protein turnover, chaperones	DnaJ domain
EVM0002150	312.70	639.48	-1.03	1.50E-04	1.14E-03	General function prediction only	RNA recognition motif. (a.k.a. RRM, RBD, or RNP domain)
EVM0008017	6.51	13.31	-1.03	1.10E-03	6.17E-03	--	ATPase family associated with various cellular activities (AAA)
EVM0007506	224.15	458.19	-1.03	2.00E-04	1.46E-03	Intracellular trafficking, secretion, and vesicular transport	Regulated-SNARE-like domain
EVM0003961	33.88	69.20	-1.03	6.00E-04	3.72E-03	--	--

EVM0009826	33.24	67.82	-1.03	1.85E-03	9.52E-03	General function prediction only	Acetyltransferase (GNAT) family
EVM0007624	7.27	14.83	-1.03	4.90E-03	2.12E-02	--	--
EVM0008967	272.82	556.23	-1.03	1.05E-03	5.94E-03	--	Tic20-like protein
EVM0003421	71.18	145.11	-1.03	5.00E-04	3.19E-03	--	--
EVM0002892	8.78	17.91	-1.03	1.30E-03	7.10E-03	Energy production and conversion	FAD binding domain
EVM0010562	57.36	116.92	-1.03	4.50E-04	2.92E-03	Posttranslational modification, protein turnover, chaperones	DnaJ domain
EVM0006891	16.18	32.98	-1.03	1.30E-03	7.10E-03	--	--
EVM0007892	105.18	214.32	-1.03	6.50E-04	3.98E-03	--	Domain of unknown function (DUF4598)
EVM0003200	106.31	216.57	-1.03	4.00E-04	2.65E-03	Posttranslational modification, protein turnover, chaperones	Cyclophilin type peptidyl-prolyl cis-trans isomerase/CLD
EVM0001034	87.28	177.72	-1.03	2.50E-04	1.77E-03	Inorganic ion transport and metabolism	Ctr copper transporter family
EVM0008633	43.13	87.74	-1.02	3.00E-04	2.07E-03	Intracellular trafficking, secretion, and vesicular transport	Autophagy-related protein 13

EVM0004019	47.99	97.54	-1.02	5.90E-03	2.46E-02	General function prediction only	Domain of unknown function (DUF3336)
EVM0004660	24.15	49.06	-1.02	2.00E-04	1.46E-03	General function prediction only	ABC transporter
EVM0002242	10.67	21.68	-1.02	9.00E-04	5.22E-03	Secondary metabolites biosynthesis, transport and catabolism	Aromatic amino acid lyase
EVM0005636	273.87	556.38	-1.02	9.00E-04	5.22E-03	Energy production and conversion	Mitochondrial glycoprotein
EVM0001803	14.08	28.60	-1.02	2.30E-03	1.14E-02	--	Haloacid dehalogenase-like hydrolase
EVM0001711	48.99	99.41	-1.02	1.50E-04	1.14E-03	Intracellular trafficking, secretion, and vesicular transport	Coatomer WD associated region
EVM0000555	40.55	82.23	-1.02	4.45E-03	1.96E-02	Lipid transport and metabolism	PAP2 superfamily
EVM0010536	2.59	5.26	-1.02	7.15E-03	2.86E-02	--	--
EVM0005171	14.11	28.60	-1.02	2.50E-04	1.77E-03	Replication, recombination and repair	DNA mismatch repair protein, C-terminal domain
EVM0004792	12.94	26.22	-1.02	1.27E-02	4.53E-02	--	--
EVM0007499	34.67	70.17	-1.02	1.10E-03	6.17E-03	--	--
EVM0003386	5.95	12.04	-1.02	5.60E-03	2.36E-02	--	bZIP transcription factor

EVM0011769	7.25	14.65	-1.02	1.11E-02	4.06E-02	--	--
EVM0007727	7.61	15.38	-1.02	4.30E-03	1.90E-02	--	--
EVM0006288	19.08	38.56	-1.02	3.00E-04	2.07E-03	--	--
EVM0008700	1311.59	2650.06	-1.01	6.55E-03	2.67E-02	Energy production and conversion	ATP synthase D chain, mitochondrial (ATP5H)
EVM0004828	668.13	1349.92	-1.01	6.50E-04	3.98E-03	Translation, ribosomal structure and biogenesis	Plectin/S10 domain
EVM0006277	8.87	17.93	-1.01	7.90E-03	3.10E-02	--	--
EVM0000834	5.46	11.03	-1.01	2.20E-03	1.10E-02	--	Glycosyl hydrolases family 28
EVM0005197	14.21	28.70	-1.01	1.80E-03	9.29E-03	General function prediction only	Major Facilitator Superfamily
EVM0004833	11.58	23.37	-1.01	3.00E-04	2.07E-03	--	--
EVM0009620	11.25	22.70	-1.01	1.05E-03	5.94E-03	General function prediction only	Sugar (and other) transporter
EVM0009627	42.78	86.31	-1.01	2.00E-04	1.46E-03	--	Inner membrane protein import complex subunit Tim54
EVM0008106	221.25	446.26	-1.01	3.50E-04	2.36E-03	--	Domain of unknown function (DUF4336)
EVM0004589	47.86	96.47	-1.01	8.50E-04	4.98E-03	--	--

EVM0002315	55.79	112.43	-1.01	2.50E-04	1.77E-03	Amino acid transport and metabolism	Aminotransferase class I and II
EVM0000850	19.18	38.65	-1.01	1.35E-03	7.33E-03	--	--
EVM0005271	11.75	23.65	-1.01	1.35E-03	7.33E-03	--	Zinc finger, C3HC4 type (RING finger)
EVM0002010	53.17	107.05	-1.01	2.50E-04	1.77E-03	--	--
EVM0006503	1800.41	3624.34	-1.01	1.65E-03	8.65E-03	Translation, ribosomal structure and biogenesis	Ribosomal protein S13/S18
EVM0002611	78.11	157.17	-1.01	5.00E-04	3.19E-03	--	Fungal domain of unknown function (DUF1750)
EVM0008251	15.94	32.06	-1.01	1.00E-04	7.96E-04	General function prediction only	Aldo/keto reductase family
EVM0005661	12.40	24.93	-1.01	2.35E-03	1.16E-02	--	--
EVM0004022	19.34	38.89	-1.01	8.50E-04	4.98E-03	General function prediction only	50S ribosome-binding GTPase
EVM0002916	134.56	270.45	-1.01	8.50E-04	4.98E-03	Posttranslational modification, protein turnover, chaperones	FKBP-type peptidyl-prolyl cis-trans isomerase
EVM0002764	99.42	199.73	-1.01	3.50E-04	2.36E-03	Transcription	TFIIE alpha subunit
EVM0008059	14.62	29.38	-1.01	5.50E-04	3.46E-03	Carbohydrate transport and metabolism	Domain of unknown function (DUF1793)

EVM0009675	57.20	114.85	-1.01	2.00E-04	1.46E-03	Posttranslational modification, protein turnover, chaperones	Oligosaccharyltransferase subunit Ribophorin II
EVM0001211	25.73	51.66	-1.01	7.00E-04	4.23E-03	--	--
EVM0006148	21.81	43.75	-1.00	1.07E-02	3.95E-02	--	--
EVM0001205	29.78	59.73	-1.00	9.00E-04	5.22E-03	Function unknown	Ribosomal protein L1p/L10e family
EVM0007899	81.21	162.84	-1.00	3.50E-04	2.36E-03	Intracellular trafficking, secretion, and vesicular transport	Yip1 domain
EVM0010713	20.36	40.82	-1.00	6.50E-04	3.98E-03	Function unknown	Protein of unknown function (DUF890)
EVM0001330	43.70	87.52	-1.00	5.00E-04	3.19E-03	Lipid transport and metabolism	Diacylglycerol acyltransferase
EVM0009094	9.87	19.77	-1.00	1.30E-03	7.10E-03	--	--
EVM0010110	9.91	19.84	-1.00	1.95E-03	9.93E-03	Carbohydrate transport and metabolism	Glycosyl hydrolase family 1
EVM0002102	713.74	1428.73	-1.00	8.10E-03	3.17E-02	Energy production and conversion	DHHA2 domain
EVM0009973	9.24	18.49	-1.00	6.00E-04	3.72E-03	--	DDE superfamily endonuclease

EVM0008505	172.60	345.43	-1.00	1.50E-04	1.14E-03	Posttranslational modification, protein turnover, chaperones	Zinc knuckle
EVM0011840	17.37	34.77	-1.00	5.00E-04	3.19E-03	--	--
EVM0011955	8.90	17.81	-1.00	3.55E-03	1.62E-02	Replication, recombination and repair	Ribonuclease HII
EVM0003013	2.48	4.96	-1.00	6.40E-03	2.62E-02	--	--
EVM0009066	41.21	82.42	-1.00	2.00E-04	1.46E-03	Function unknown	Rubisco LSMT substrate-binding
EVM0007015	16.83	8.41	1.00	6.50E-04	3.98E-03	--	Acyl transferase domain
EVM0009455	5.16	2.58	1.00	3.50E-03	1.61E-02	--	NACHT domain
EVM0002765	22.94	11.45	1.00	7.50E-04	4.49E-03	General function prediction only	Rab-GTPase-TBC domain
EVM0000199	248.93	124.22	1.00	6.00E-04	3.72E-03	Signal transduction mechanisms	HEAT repeat
EVM0001289	80.85	40.34	1.00	3.00E-04	2.07E-03	--	--
EVM0007405	690.26	344.37	1.00	4.50E-04	2.92E-03	Intracellular trafficking, secretion, and vesicular transport	RanBP1 domain
EVM0008123	70.86	35.31	1.00	3.50E-04	2.36E-03	Translation, ribosomal structure and biogenesis	Initiation factor 2 subunit family

EVM0010778	7.32	3.65	1.01	1.50E-03	8.00E-03	--	SNF2 family N-terminal domain
EVM0007126	93.77	46.71	1.01	5.00E-04	3.19E-03	General function prediction only	Autophagocytosis associated protein (Atg3), N-terminal domain
EVM0000270	64.59	32.16	1.01	6.50E-04	3.98E-03	--	--
EVM0011948	54.37	27.06	1.01	1.60E-03	8.44E-03	Posttranslational modification, protein turnover, chaperones	Zinc knuckle
EVM0001740	99.41	49.45	1.01	1.20E-03	6.64E-03	--	2OG-Fe(II) oxygenase superfamily
EVM0003881	8.86	4.41	1.01	4.35E-03	1.92E-02	Replication, recombination and repair	impB/mucB/samB family
EVM0002143	44.34	22.05	1.01	5.00E-04	3.19E-03	Cytoskeleton	Kinesin motor domain
EVM0010722	21.84	10.86	1.01	5.00E-04	3.19E-03	Transcription	IKI3 family
EVM0011360	13.67	6.79	1.01	1.70E-03	8.87E-03	--	--
EVM0004284	42.47	21.09	1.01	4.50E-04	2.92E-03	--	WSC domain
EVM0000621	29.37	14.59	1.01	1.60E-03	8.44E-03	--	Oxidoreductase family, NAD-binding Rossmann fold
EVM0006311	59.02	29.29	1.01	1.00E-02	3.75E-02	Defense mechanisms	Macrophage migration inhibitory factor (MIF)

EVM0001907	60.21	29.88	1.01	3.50E-04	2.36E-03	--	--
EVM0002001	69.71	34.58	1.01	4.00E-04	2.65E-03	General function prediction only	Cyclin
EVM0009097	41.53	20.60	1.01	2.60E-03	1.26E-02	Lipid transport and metabolism	GDSL-like Lipase/Acylhydrolase family
EVM0005381	65.97	32.71	1.01	4.00E-04	2.65E-03	--	--
EVM0004079	66.25	32.84	1.01	2.70E-03	1.30E-02	Amino acid transport and metabolism	Phosphoadenosine phosphosulfate reductase family
EVM0010858	13.92	6.90	1.01	1.15E-03	6.41E-03	--	--
EVM0001397	1172.24	580.88	1.01	1.85E-03	9.52E-03	General function prediction only	Zinc-finger double-stranded RNA-binding
EVM0007333	18.51	9.17	1.01	7.00E-04	4.23E-03	RNA processing and modification	Ribonuclease III domain
EVM0012209	97.00	48.03	1.01	1.00E-03	5.70E-03	Function unknown	Uncharacterised protein family (UPF0183)
EVM0007079	30.67	15.18	1.01	4.00E-04	2.65E-03	Transcription	HMG (high mobility group) box
EVM0009576	4.95	2.45	1.01	3.30E-03	1.53E-02	--	NACHT domain
EVM0001859	670.97	332.02	1.01	3.70E-03	1.68E-02	Amino acid transport and metabolism	D-isomer specific 2-hydroxyacid dehydrogenase, NAD binding domain

EVM0000526	111.15	54.97	1.02	5.50E-04	3.46E-03	Function unknown	Protein of unknown function (DUF726)
EVM0008544	5.42	2.68	1.02	1.04E-02	3.85E-02	--	Major Facilitator Superfamily
EVM0006531	38.32	18.94	1.02	1.39E-02	4.86E-02	--	Protein of unknown function (DUF2423)
EVM0009046	29.26	14.46	1.02	4.00E-04	2.65E-03	Replication, recombination and repair	DNA mismatch repair protein, C-terminal domain
EVM0012227	22.53	11.13	1.02	1.15E-03	6.41E-03	--	--
EVM0006259	23.38	11.54	1.02	7.00E-04	4.23E-03	--	MYND finger
EVM0008313	27.63	13.63	1.02	6.50E-04	3.98E-03	Secondary metabolites biosynthesis, transport and catabolism	ABC transporter transmembrane region
EVM0007923	12.95	6.38	1.02	1.30E-03	7.10E-03	--	Amidase
EVM0004419	52.94	26.09	1.02	1.65E-03	8.65E-03	General function prediction only	Protein kinase domain
EVM0003953	10.72	5.28	1.02	4.35E-03	1.92E-02	--	NACHT domain
EVM0003247	120.39	59.30	1.02	1.00E-03	5.70E-03	Signal transduction mechanisms	Protein kinase domain
EVM0010764	268.55	132.21	1.02	9.50E-04	5.46E-03	Carbohydrate transport and metabolism	Transketolase, thiamine diphosphate binding domain

EVM0001744	11.97	5.89	1.02	7.50E-04	4.49E-03	Signal transduction mechanisms	Protein kinase domain
EVM0006716	23.99	11.81	1.02	3.80E-03	1.72E-02	--	--
EVM0005766	63.65	31.29	1.02	1.50E-04	1.14E-03	--	Adenylate cyclase associated (CAP) N terminal
EVM0004370	24.47	12.02	1.03	1.40E-03	7.55E-03	General function prediction only	Lipopolysaccharide kinase (Kdo/WaaP) family
EVM0005270	30.21	14.84	1.03	8.00E-04	4.73E-03	Transcription	R3H domain
EVM0009823	87.74	43.08	1.03	6.00E-04	3.72E-03	--	HOOK protein
EVM0004470	39.70	19.47	1.03	1.00E-04	7.96E-04	Inorganic ion transport and metabolism	Cation transport protein
EVM0003730	24.00	11.76	1.03	3.65E-03	1.66E-02	--	Uncharacterized alpha/beta hydrolase domain (DUF2235)
EVM0002727	1058.91	519.11	1.03	4.40E-03	1.94E-02	--	Cupin
EVM0011120	1679.46	822.56	1.03	9.65E-03	3.64E-02	Inorganic ion transport and metabolism	Sugar (and other) transporter
EVM0010709	110.42	54.08	1.03	1.50E-04	1.14E-03	Nucleotide transport and metabolism	Phosphorylase superfamily
EVM0001332	32.70	16.01	1.03	8.50E-04	4.98E-03	General function prediction only	Acetyltransferase (GNAT) domain

EVM0001339	1086.00	531.72	1.03	4.15E-03	1.85E-02	Posttranslational modification, protein turnover, chaperones	GDP dissociation inhibitor
EVM0001963	72.35	35.37	1.03	4.00E-04	2.65E-03	--	Alpha/beta hydrolase family
EVM0003280	15.24	7.44	1.03	1.23E-02	4.40E-02	--	--
EVM0011492	1057.93	516.65	1.03	1.05E-02	3.88E-02	--	--
EVM0010906	113.24	55.24	1.04	6.00E-04	3.72E-03	--	CorA-like Mg2+ transporter protein
EVM0008175	22.31	10.88	1.04	5.00E-04	3.19E-03	--	FAT domain
EVM0009032	73.78	35.97	1.04	1.50E-04	1.14E-03	General function prediction only	WD domain, G-beta repeat
EVM0004703	65.28	31.79	1.04	8.00E-04	4.73E-03	Posttranslational modification, protein turnover, chaperones	Leucine carboxyl methyltransferase
EVM0010016	70.23	34.20	1.04	4.00E-04	2.65E-03	General function prediction only	Oxidoreductase family, NAD-binding Rossmann fold
EVM0005203	36.14	17.59	1.04	2.00E-04	1.46E-03	Intracellular trafficking, secretion, and vesicular transport	Pep3/Vps18/deep orange family
EVM0005607	7.37	3.59	1.04	4.90E-03	2.12E-02	Posttranslational modification, protein turnover, chaperones	Prenyltransferase and squalene oxidase repeat

EVM0004132	22.48	10.94	1.04	3.50E-04	2.36E-03	General function prediction only	RAVE protein 1 C terminal
EVM0009994	43.25	21.03	1.04	8.00E-04	4.73E-03	--	--
EVM0006478	169.41	82.34	1.04	1.50E-04	1.14E-03	--	N-terminal domain of ribose phosphate pyrophosphokinase
EVM0010432	140.08	68.07	1.04	2.50E-04	1.77E-03	Posttranslational modification, protein turnover, chaperones	TCP-1/cpn60 chaperonin family
EVM0001504	357.56	173.68	1.04	4.50E-04	2.92E-03	--	--
EVM0005070	328.70	159.60	1.04	2.00E-04	1.46E-03	--	EB1-like C-terminal motif
EVM0006155	40.09	19.46	1.04	3.10E-03	1.45E-02	Cell wall/membrane/envelope biogenesis	Glycosyl hydrolases family 18
EVM0003284	21.60	10.48	1.04	6.50E-04	3.98E-03	--	--
EVM0008024	585.65	283.92	1.04	7.55E-03	2.99E-02	--	--
EVM0006412	396.69	192.24	1.05	5.50E-04	3.46E-03	--	Bin/amphiphysin/Rvs domain for vesicular trafficking
EVM0006229	663.64	321.60	1.05	2.95E-03	1.40E-02	Inorganic ion transport and metabolism	Sugar (and other) transporter

EVM0002077	26.03	12.61	1.05	1.10E-03	6.17E-03	Posttranslational modification, protein turnover, chaperones	Ankyrin repeat
EVM0008486	209.82	101.62	1.05	9.00E-04	5.22E-03	--	HEAT-like repeat
EVM0003260	39.87	19.30	1.05	2.00E-04	1.46E-03	--	Regulator of chromosome condensation (RCC1) repeat
EVM0010705	115.90	56.11	1.05	1.05E-03	5.94E-03	Transcription	SRF-type transcription factor (DNA-binding and dimerisation domain)
EVM0000563	5.30	2.56	1.05	6.30E-03	2.59E-02	RNA processing and modification	Initiator tRNA phosphoribosyl transferase
EVM0010904	21.68	10.48	1.05	3.50E-04	2.36E-03	Inorganic ion transport and metabolism	Sodium/hydrogen exchanger family
EVM0000071	4.34	2.10	1.05	6.10E-03	2.52E-02	--	--
EVM0002141	43.68	21.10	1.05	9.35E-03	3.55E-02	--	--
EVM0001077	15.50	7.48	1.05	8.00E-04	4.73E-03	Signal transduction mechanisms	Phosphatidylinositol-specific phospholipase C, X domain
EVM0011682	52.91	25.55	1.05	2.00E-04	1.46E-03	Signal transduction mechanisms	Frag1/DRAM/Sfk1 family
EVM0003715	132.86	64.00	1.05	2.50E-04	1.77E-03	--	PIN domain
EVM0007345	481.96	232.16	1.05	2.80E-03	1.34E-02	Amino acid transport and metabolism	POT family

EVM0002666	110.19	53.02	1.06	5.50E-04	3.46E-03	Translation, ribosomal structure and biogenesis	tRNA synthetases class I (I, L, M and V)
EVM0002651	484.05	232.85	1.06	3.00E-04	2.07E-03	Translation, ribosomal structure and biogenesis	Endoribonuclease L-PSP
EVM0000238	695.40	334.49	1.06	7.00E-04	4.23E-03	Translation, ribosomal structure and biogenesis	eRF1 domain 2
EVM0008239	622.33	299.26	1.06	5.50E-04	3.46E-03	Carbohydrate transport and metabolism	Transaldolase
EVM0011578	312.28	150.12	1.06	8.50E-04	4.98E-03	--	--
EVM0005878	76.09	36.55	1.06	2.00E-04	1.46E-03	--	--
EVM0011361	70.19	33.69	1.06	1.25E-03	6.87E-03	--	--
EVM0000331	34.73	16.65	1.06	4.85E-03	2.10E-02	--	--
EVM0000242	107.99	51.76	1.06	5.45E-03	2.31E-02	Carbohydrate transport and metabolism	Trehalase
EVM0002699	50.33	24.12	1.06	4.30E-03	1.90E-02	Function unknown	--
EVM0011634	26.83	12.86	1.06	2.00E-04	1.46E-03	Function unknown	Spc97 / Spc98 family
EVM0004970	175.52	84.09	1.06	1.00E-03	5.70E-03	Energy production and conversion	Malic enzyme, NAD binding domain
EVM0006142	12.60	6.03	1.06	5.10E-03	2.19E-02	--	G protein-coupled glucose receptor regulating Gpa2

EVM0001119	40.52	19.39	1.06	3.50E-04	2.36E-03	Signal transduction mechanisms	Response regulator receiver domain
EVM0006880	43.02	20.58	1.06	2.10E-03	1.06E-02	General function prediction only	Glycosyltransferase family 28 C-terminal domain
EVM0009089	12.55	6.00	1.07	2.30E-03	1.14E-02	Inorganic ion transport and metabolism	Carbonic anhydrase
EVM0008027	113.79	54.36	1.07	9.50E-04	5.46E-03	--	--
EVM0011485	129.93	62.05	1.07	2.50E-04	1.77E-03	Amino acid transport and metabolism	Indole-3-glycerol phosphate synthase
EVM0002456	557.03	265.88	1.07	1.10E-03	6.17E-03	Cytoskeleton	Microtubule-associated protein CRIPT
EVM0007895	141.08	67.33	1.07	4.50E-04	2.92E-03	--	CRM1 C terminal
EVM0006748	78.41	37.40	1.07	4.50E-04	2.92E-03	Function unknown	TPR/MLP1/MLP2-like protein
EVM0008990	23.46	11.19	1.07	2.00E-04	1.46E-03	RNA processing and modification	Helicase associated domain (HA2)
EVM0009552	323.36	154.22	1.07	7.50E-04	4.49E-03	--	--
EVM0011140	54.89	26.17	1.07	2.00E-04	1.46E-03	Intracellular trafficking, secretion, and vesicular transport	Lethal giant larvae(Lgl) like, C-terminal
EVM0006968	30.95	14.73	1.07	4.35E-03	1.92E-02	--	--

EVM0001643	358.11	170.37	1.07	1.00E-04	7.96E-04	Intracellular trafficking, secretion, and vesicular transport	Synaptobrevin
EVM0007424	10.51	5.00	1.07	4.10E-03	1.83E-02	Lipid transport and metabolism	C2 domain
EVM0006727	32.36	15.37	1.07	1.45E-03	7.77E-03	--	--
EVM0010125	250.51	118.90	1.08	1.30E-03	7.10E-03	--	--
EVM0007527	220.25	104.46	1.08	1.50E-03	8.00E-03	Intracellular trafficking, secretion, and vesicular transport	Region in Clathrin and VPS
EVM0002952	27.88	13.22	1.08	3.50E-04	2.36E-03	Extracellular structures	Tropomyosin
EVM0009427	13.48	6.38	1.08	3.00E-03	1.42E-02	--	Staphylococcal nuclease homologue
EVM0005015	177.89	84.15	1.08	1.00E-04	7.96E-04	--	Triose-phosphate Transporter family
EVM0005723	53.86	25.47	1.08	3.50E-04	2.36E-03	Transcription	RNA polymerase Rpb1, domain 5
EVM0009342	50.64	23.91	1.08	1.00E-04	7.96E-04	--	von Willebrand factor type A domain
EVM0008508	21.45	10.13	1.08	8.80E-03	3.38E-02	--	--
EVM0007370	12.46	5.87	1.09	1.00E-03	5.70E-03	--	--

EVM0004931	67.18	31.56	1.09	1.00E-04	7.96E-04	--	Munc13 (mammalian uncoordinated) homology domain
EVM0009149	370.51	173.99	1.09	3.50E-04	2.36E-03	--	--
EVM0009549	41.73	19.59	1.09	2.50E-04	1.77E-03	--	Zinc-binding dehydrogenase
EVM0000859	2.78	1.31	1.09	9.95E-03	3.74E-02	--	--
EVM0009161	15.10	7.08	1.09	4.50E-04	2.92E-03	--	Fungal Zn(2)-Cys(6) binuclear cluster domain
EVM0010426	981.69	460.22	1.09	5.50E-04	3.46E-03	--	--
EVM0007608	606.62	284.32	1.09	4.50E-03	1.97E-02	--	Domain of unknown function (DUF4203)
EVM0001912	73.35	34.37	1.09	3.50E-04	2.36E-03	Amino acid transport and metabolism	EPSP synthase (3-phosphoshikimate 1-carboxyvinyltransferase)
EVM0006980	9.20	4.31	1.09	4.15E-03	1.85E-02	--	--
EVM0012077	48.58	22.74	1.10	3.00E-04	2.07E-03	--	Isoprenylcysteine carboxyl methyltransferase (ICMT) family
EVM0005907	25.68	12.02	1.10	6.95E-03	2.80E-02	--	--
EVM0008335	36.68	17.14	1.10	5.00E-05	4.28E-04	Function unknown	Protein of unknown function (DUF3414)

EVM0005769	21.43	10.01	1.10	1.35E-03	7.33E-03	--	Common central domain of tyrosinase
EVM0011534	22.27	10.39	1.10	7.50E-04	4.49E-03	--	--
EVM0005101	3.50	1.63	1.10	1.27E-02	4.53E-02	Replication, recombination and repair	HhH-GPD superfamily base excision DNA repair protein
EVM0002944	21.25	9.90	1.10	1.10E-03	6.17E-03	--	WD domain, G-beta repeat
EVM0006521	27.46	12.79	1.10	2.10E-03	1.06E-02	--	--
EVM0010324	143.74	66.94	1.10	5.00E-05	4.28E-04	--	--
EVM0008988	17.55	8.17	1.10	5.00E-05	4.28E-04	--	--
EVM0004414	65.24	30.35	1.10	1.50E-04	1.14E-03	--	Polysaccharide deacetylase
EVM0001572	15.78	7.34	1.10	4.50E-04	2.92E-03	General function prediction only	O-methyltransferase
EVM0003504	22.13	10.29	1.10	1.50E-03	8.00E-03	General function prediction only	SPRY domain
EVM0001546	222.22	103.33	1.10	2.50E-04	1.77E-03	Cytoskeleton	Domain of unknown function (DUF1900)
EVM0004506	113.34	52.63	1.11	2.50E-04	1.77E-03	Carbohydrate transport and metabolism	Phosphofructokinase
EVM0004193	26.15	12.14	1.11	6.50E-04	3.98E-03	--	NADH:flavin oxidoreductase / NADH oxidase family

EVM0002486	8.42	3.91	1.11	3.40E-03	1.57E-02	Function unknown	Protein of unknown function (DUF544)
EVM0000940	32.79	15.21	1.11	5.00E-05	4.28E-04	Lipid transport and metabolism	AMP-binding enzyme
EVM0008035	18.88	8.75	1.11	2.00E-04	1.46E-03	General function prediction only	SNF2 family N-terminal domain
EVM0004355	82.81	38.38	1.11	5.00E-05	4.28E-04	Signal transduction mechanisms	RasGEF domain
EVM0003043	30.27	14.03	1.11	5.00E-03	2.15E-02	--	Cyanate lyase C-terminal domain
EVM0006564	13.28	6.15	1.11	1.30E-03	7.10E-03	Carbohydrate transport and metabolism	Melibiase
EVM0008825	111.89	51.82	1.11	7.65E-03	3.02E-02	--	--
EVM0007477	40.13	18.58	1.11	3.00E-04	2.07E-03	General function prediction only	Metallo-beta-lactamase superfamily
EVM0008081	395.87	183.19	1.11	1.00E-04	7.96E-04	Translation, ribosomal structure and biogenesis	Ribosomal protein S19
EVM0005901	3.32	1.53	1.11	1.34E-02	4.72E-02	Carbohydrate transport and metabolism	Melibiase
EVM0004320	137.75	63.67	1.11	5.00E-05	4.28E-04	General function prediction only	Aldo/keto reductase family
EVM0003720	179.82	83.12	1.11	5.00E-05	4.28E-04	--	NAD dependent epimerase/dehydratase family
EVM0005612	49.77	23.01	1.11	1.00E-04	7.96E-04	Energy production and conversion	Aldehyde dehydrogenase family

EVM0001598	142.73	65.96	1.11	3.00E-04	2.07E-03	Cytoskeleton	ANTH domain
EVM0009630	75.26	34.78	1.11	8.00E-04	4.73E-03	Posttranslational modification, protein turnover, chaperones	PITH domain
EVM0000748	19.30	8.91	1.12	1.45E-03	7.77E-03	Secondary metabolites biosynthesis, transport and catabolism	Zinc-binding dehydrogenase
EVM0012228	31.21	14.38	1.12	1.00E-04	7.96E-04	--	--
EVM0009461	10.00	4.61	1.12	6.50E-04	3.98E-03	General function prediction only	Zinc-finger of C2H2 type
EVM0001221	88.35	40.71	1.12	1.00E-04	7.96E-04	Function unknown	WD domain, G-beta repeat
EVM0002379	112.92	52.03	1.12	1.50E-04	1.14E-03	Translation, ribosomal structure and biogenesis	tRNA synthetases class I (E and Q), catalytic domain
EVM0011550	19.28	8.88	1.12	3.00E-04	2.07E-03	--	--
EVM0010049	210.10	96.73	1.12	3.00E-04	2.07E-03	Posttranslational modification, protein turnover, chaperones	IBR domain
EVM0010561	87.50	40.28	1.12	5.00E-05	4.28E-04	--	Caspase domain
EVM0009313	58.26	26.81	1.12	5.00E-05	4.28E-04	Intracellular trafficking, secretion, and vesicular transport	Adaptin N terminal region

EVM0008667	14.29	6.57	1.12	7.00E-04	4.23E-03	--	MYND finger
EVM0009405	88.83	40.84	1.12	2.30E-03	1.14E-02	General function prediction only	Yippee zinc-binding/DNA-binding /Mis18, centromere assembly
EVM0004176	675.73	310.50	1.12	9.00E-04	5.22E-03	--	--
EVM0009704	78.21	35.87	1.12	5.00E-05	4.28E-04	Amino acid transport and metabolism	Lyase
EVM0000724	61.85	28.36	1.12	1.50E-04	1.14E-03	Translation, ribosomal structure and biogenesis	Piwi domain
EVM0008503	37.20	17.05	1.13	9.00E-04	5.22E-03	Lipid transport and metabolism	short chain dehydrogenase
EVM0002940	522.39	239.19	1.13	3.00E-04	2.07E-03	General function prediction only	Zinc finger, C2H2 type
EVM0007352	36.15	16.54	1.13	1.00E-04	7.96E-04	General function prediction only	Protein kinase domain
EVM0004309	309.87	141.76	1.13	7.50E-04	4.49E-03	--	Ferric reductase like transmembrane component
EVM0011502	272.25	124.39	1.13	1.50E-04	1.14E-03	Energy production and conversion	Citrate synthase
EVM0006225	57.88	26.43	1.13	4.00E-04	2.65E-03	--	--
EVM0003215	68.08	31.06	1.13	1.50E-04	1.14E-03	Signal transduction mechanisms	Leucine Rich repeats (2 copies)

EVM0003091	117.40	53.56	1.13	1.50E-04	1.14E-03	Posttranslational modification, protein turnover, chaperones	Zinc finger, C3HC4 type (RING finger)
EVM0004348	44.36	20.23	1.13	9.50E-04	5.46E-03	Lipid transport and metabolism	Ceramidase
EVM0009974	66.42	30.25	1.13	7.50E-04	4.49E-03	Signal transduction mechanisms	GTPase-activator protein for Ras-like GTPase
EVM0007827	423.46	192.64	1.14	2.20E-03	1.10E-02	--	Lipase (class 3)
EVM0008727	16.13	7.34	1.14	1.19E-02	4.30E-02	Translation, ribosomal structure and biogenesis	D-Tyr-tRNA(Tyr) deacylase
EVM0006999	166.86	75.88	1.14	1.50E-04	1.14E-03	Coenzyme transport and metabolism	GTP cyclohydrolase N terminal
EVM0004131	49.37	22.44	1.14	5.00E-05	4.28E-04	General function prediction only	TPR repeat
EVM0003517	119.02	54.04	1.14	1.50E-04	1.14E-03	Posttranslational modification, protein turnover, chaperones	Hsp70 protein
EVM0010856	28.50	12.93	1.14	1.55E-03	8.22E-03	--	--
EVM0003378	176.81	80.22	1.14	2.00E-04	1.46E-03	--	Domain of unknown function (DUF427)
EVM0011658	5.66	2.57	1.14	1.06E-02	3.91E-02	Lipid transport and metabolism	Enoyl-CoA hydratase/isomerase family
EVM0005098	1220.76	553.55	1.14	7.50E-04	4.49E-03	--	--

EVM0003885	1194.51	541.34	1.14	6.50E-04	3.98E-03	--	--
EVM0009293	529.11	239.66	1.14	4.50E-04	2.92E-03	--	--
EVM0007183	23.96	10.83	1.15	5.50E-04	3.46E-03	--	Enolase C-terminal domain-like
EVM0003658	7.00	3.16	1.15	2.10E-03	1.06E-02	--	--
EVM0001184	4.97	2.24	1.15	1.42E-02	4.95E-02	--	--
EVM0003687	153.62	68.88	1.16	4.50E-04	2.92E-03	Amino acid transport and metabolism	Chorismate synthase
EVM0006983	102.60	45.87	1.16	1.00E-04	7.96E-04	--	DAHP synthetase I family
EVM0011564	377.19	168.61	1.16	5.00E-05	4.28E-04	General function prediction only	Major Facilitator Superfamily
EVM0004126	19.57	8.74	1.16	5.00E-05	4.28E-04	Cell cycle control, cell division, chromosome partitioning	RecF/RecN/SMC N terminal domain
EVM0003696	10.58	4.72	1.16	9.80E-03	3.69E-02	--	--
EVM0006484	24.27	10.83	1.16	5.00E-04	3.19E-03	Lipid transport and metabolism	Lipase (class 3)
EVM0008184	31.37	14.00	1.16	1.50E-04	1.14E-03	--	Domain of unknown function (DUF4139)

EVM0002393	3.57	1.59	1.16	6.30E-03	2.59E-02	--	--
EVM0007960	3.95	1.76	1.17	1.70E-03	8.87E-03	--	Heterokaryon incompatibility protein (HET)
EVM0010328	21.35	9.51	1.17	2.50E-04	1.77E-03	Replication, recombination and repair	Exonuclease
EVM0012160	11.14	4.96	1.17	5.00E-05	4.28E-04	--	Fungal specific transcription factor domain
EVM0003587	105.49	46.96	1.17	1.00E-04	7.96E-04	Coenzyme transport and metabolism	UbiA prenyltransferase family
EVM0010285	12.17	5.41	1.17	6.50E-04	3.98E-03	--	Domain of unknown function (DUF1917)
EVM0011547	182.71	81.30	1.17	5.00E-05	4.28E-04	Lipid transport and metabolism	Calcineurin-like phosphoesterase
EVM0000895	26.48	11.77	1.17	1.10E-03	6.17E-03	Nucleotide transport and metabolism	Thymidylate kinase
EVM0000594	22.41	9.96	1.17	1.35E-03	7.33E-03	--	NmrA-like family
EVM0002329	476.03	211.26	1.17	3.00E-04	2.07E-03	Carbohydrate transport and metabolism	Pyruvate kinase, barrel domain
EVM0009195	20.83	9.24	1.17	5.00E-04	3.19E-03	--	Survival protein SurE
EVM0010130	52.77	23.40	1.17	1.00E-04	7.96E-04	Signal transduction mechanisms	Putative GTPase activating protein for Arf
EVM0011905	120.34	53.34	1.17	5.00E-05	4.28E-04	--	Iron permease FTR1 family

EVM0008661	65.26	28.88	1.18	1.50E-04	1.14E-03	Posttranslational modification, protein turnover, chaperones	ThiF family
EVM0006159	136.24	60.24	1.18	2.50E-04	1.77E-03	--	--
EVM0001664	1.70	0.75	1.18	9.95E-03	3.74E-02	Function unknown	Major Facilitator Superfamily
EVM0001458	3.61	1.59	1.18	5.10E-03	2.19E-02	--	--
EVM0011654	13.61	6.00	1.18	1.30E-03	7.10E-03	--	--
EVM0000006	18.65	8.22	1.18	1.00E-04	7.96E-04	Secondary metabolites biosynthesis, transport and catabolism	Cytochrome P450
EVM0003493	265.50	117.02	1.18	7.00E-04	4.23E-03	Cytoskeleton	Calponin homology (CH) domain
EVM0000788	38.64	17.03	1.18	7.00E-04	4.23E-03	--	PH domain
EVM0003301	41.44	18.26	1.18	5.00E-05	4.28E-04	Translation, ribosomal structure and biogenesis	Elongation factor Tu GTP binding domain
EVM0002142	143.74	63.33	1.18	5.00E-05	4.28E-04	General function prediction only	Cyclin
EVM0003294	16.25	7.16	1.18	5.00E-04	3.19E-03	--	Pro-kumamolisin, activation domain
EVM0005952	9.39	4.14	1.18	1.10E-03	6.17E-03	--	RNA ligase

EVM0002799	90.20	39.71	1.18	2.00E-04	1.46E-03	General function prediction only	Putative Phosphatase
EVM0008652	65.18	28.69	1.18	7.00E-04	4.23E-03	--	--
EVM0010437	89.44	39.35	1.18	6.50E-04	3.98E-03	--	--
EVM0012216	70.06	30.82	1.18	5.00E-05	4.28E-04	General function prediction only	short chain dehydrogenase
EVM0005569	27.69	12.18	1.18	4.50E-04	2.92E-03	--	--
EVM0001175	18.28	8.03	1.19	1.09E-02	4.01E-02	--	TEA/ATTS domain family
EVM0002290	51.76	22.73	1.19	2.50E-03	1.22E-02	Posttranslational modification, protein turnover, chaperones	Ubiquitin-conjugating enzyme
EVM0006183	27.31	11.99	1.19	5.00E-05	4.28E-04	--	--
EVM0007829	92.00	40.36	1.19	5.00E-05	4.28E-04	Amino acid transport and metabolism	HMGL-like
EVM0004732	78.10	34.25	1.19	5.00E-05	4.28E-04	Amino acid transport and metabolism	Acyl-CoA dehydrogenase, C-terminal domain
EVM0008712	58.54	25.65	1.19	5.00E-05	4.28E-04	Replication, recombination and repair	NUDIX domain
EVM0008440	14.61	6.40	1.19	7.50E-04	4.49E-03	Signal transduction mechanisms	Protein kinase domain

EVM0002090	45.82	20.03	1.19	5.00E-05	4.28E-04	Lipid transport and metabolism	Taurine catabolism dioxygenase TauD, TfdA family
EVM0009132	71.55	31.27	1.19	4.00E-04	2.65E-03	--	--
EVM0007663	410.44	179.35	1.19	5.00E-05	4.28E-04	Lipid transport and metabolism	Alpha/beta hydrolase family
EVM0003972	221.74	96.88	1.19	1.00E-04	7.96E-04	Signal transduction mechanisms	Protein kinase domain
EVM0007249	44.91	19.62	1.19	5.00E-05	4.28E-04	--	RhoGEF domain
EVM0007050	76.48	33.40	1.20	1.00E-04	7.96E-04	General function prediction only	Aminotransferase class-V
EVM0006520	10.62	4.63	1.20	2.00E-04	1.46E-03	--	--
EVM0011443	204.03	88.99	1.20	5.00E-05	4.28E-04	Amino acid transport and metabolism	Saccharopine dehydrogenase
EVM0006655	18.13	7.90	1.20	1.50E-04	1.14E-03	Nucleotide transport and metabolism	Adenosine/AMP deaminase
EVM0001322	388.32	169.21	1.20	3.50E-04	2.36E-03	Secondary metabolites biosynthesis, transport and catabolism	Cytochrome P450
EVM0007926	7.76	3.38	1.20	3.00E-04	2.07E-03	--	--
EVM0005744	8.96	3.90	1.20	1.10E-03	6.17E-03	RNA processing and modification	AAA domain

EVM0006510	178.81	77.77	1.20	1.00E-04	7.96E-04	General function prediction only	Sugar (and other) transporter
EVM0000265	179.93	78.22	1.20	3.50E-04	2.36E-03	Cell wall/membrane/envelope biogenesis	Chitin synthase
EVM0010470	144.34	62.71	1.20	5.00E-05	4.28E-04	Posttranslational modification, protein turnover, chaperones	Proteasome/cyclosome repeat
EVM0009368	66.11	28.71	1.20	5.00E-05	4.28E-04	--	--
EVM0009382	17.37	7.54	1.20	2.00E-04	1.46E-03	General function prediction only	Elongation factor Tu C-terminal domain
EVM0002963	72.68	31.53	1.21	1.50E-04	1.14E-03	--	--
EVM0008164	13.05	5.66	1.21	4.00E-04	2.65E-03	Carbohydrate transport and metabolism	Major Facilitator Superfamily
EVM0010296	25.33	10.97	1.21	1.50E-04	1.14E-03	General function prediction only	50S ribosome-binding GTPase
EVM0001679	365.34	158.13	1.21	4.50E-04	2.92E-03	Carbohydrate transport and metabolism	Alpha amylase, C-terminal all-beta domain
EVM0000891	140.13	60.64	1.21	5.00E-05	4.28E-04	--	Pyridoxamine 5'-phosphate oxidase
EVM0000749	202.33	87.46	1.21	5.00E-05	4.28E-04	General function prediction only	Ras family
EVM0011666	10.55	4.55	1.21	1.00E-04	7.96E-04	--	MYND finger

EVM0005837	36.58	15.80	1.21	5.00E-05	4.28E-04	Translation, ribosomal structure and biogenesis	PUA domain
EVM0011775	98.74	42.63	1.21	5.00E-05	4.28E-04	Posttranslational modification, protein turnover, chaperones	Ubiquitin carboxyl-terminal hydrolase
EVM0004800	234.89	101.39	1.21	5.00E-05	4.28E-04	Coenzyme transport and metabolism	Delta-aminolevulinic acid dehydratase
EVM0008666	53.45	23.05	1.21	1.50E-04	1.14E-03	--	Dioxygenase
EVM0006667	48.14	20.75	1.21	5.00E-05	4.28E-04	--	--
EVM0004493	1036.82	445.89	1.22	6.00E-04	3.72E-03	General function prediction only	Cyclin, N-terminal domain
EVM0012168	25.50	10.96	1.22	5.00E-05	4.28E-04	--	Importin-beta N-terminal domain
EVM0004894	252.71	108.62	1.22	5.00E-05	4.28E-04	Energy production and conversion	FAD binding domain
EVM0009926	26.13	11.23	1.22	1.00E-04	7.96E-04	--	AMP-binding enzyme
EVM0003798	39.42	16.93	1.22	1.30E-03	7.10E-03	--	Thaumatin family
EVM0002676	56.49	24.25	1.22	1.00E-04	7.96E-04	Secondary metabolites biosynthesis, transport and catabolism	Cytochrome P450
EVM0011368	19.30	8.28	1.22	2.50E-04	1.77E-03	--	--

EVM0008401	35.59	15.27	1.22	5.00E-05	4.28E-04	Posttranslational modification, protein turnover, chaperones	Ubiquitin carboxyl-terminal hydrolase
EVM0007654	530.14	227.44	1.22	5.00E-05	4.28E-04	Signal transduction mechanisms	Eukaryotic cytochrome b561
EVM0001845	131.71	56.45	1.22	2.50E-04	1.77E-03	General function prediction only	haloacid dehalogenase-like hydrolase
EVM0004033	86.45	37.03	1.22	5.00E-05	4.28E-04	Posttranslational modification, protein turnover, chaperones	TCP-1/cpn60 chaperonin family
EVM0001998	148.53	63.39	1.23	1.00E-04	7.96E-04	Extracellular structures	--
EVM0009352	69.75	29.76	1.23	5.00E-05	4.28E-04	Energy production and conversion	2-oxoacid dehydrogenases acyltransferase (catalytic domain)
EVM0005286	406.86	173.56	1.23	5.00E-05	4.28E-04	--	--
EVM0003494	71.42	30.45	1.23	5.00E-05	4.28E-04	General function prediction only	Rab-GTPase-TBC domain
EVM0001416	7.56	3.22	1.23	5.80E-03	2.42E-02	--	Thaumatin family
EVM0000144	68.22	29.05	1.23	1.50E-04	1.14E-03	General function prediction only	Phenazine biosynthesis-like protein
EVM0003809	10.49	4.46	1.23	1.00E-03	5.70E-03	--	--
EVM0008931	39.74	16.88	1.24	5.00E-05	4.28E-04	--	Growth-Arrest-Specific Protein 2 Domain

EVM0006340	56.11	23.83	1.24	5.00E-05	4.28E-04	--	Nup53/35/40-type RNA recognition motif
EVM0009106	48.34	20.53	1.24	5.00E-05	4.28E-04	--	--
EVM0006467	44.28	18.80	1.24	1.00E-04	7.96E-04	--	Zinc-binding dehydrogenase
EVM0003945	180.22	76.53	1.24	1.00E-04	7.96E-04	Cell wall/membrane/envelope biogenesis	Chitin synthase
EVM0010408	40.71	17.28	1.24	5.00E-05	4.28E-04	Translation, ribosomal structure and biogenesis	MIF4G domain
EVM0012002	49.17	20.83	1.24	5.00E-05	4.28E-04	General function prediction only	WD domain, G-beta repeat
EVM0003882	147.01	62.29	1.24	1.00E-04	7.96E-04	Energy production and conversion	Pyridine nucleotide-disulphide oxidoreductase
EVM0000399	269.93	114.27	1.24	1.00E-04	7.96E-04	Posttranslational modification, protein turnover, chaperones	Metallo-peptidase family M12
EVM0007902	145.96	61.61	1.24	5.00E-05	4.28E-04	--	Fungal specific transcription factor domain
EVM0007315	203.70	85.90	1.25	5.00E-05	4.28E-04	Amino acid transport and metabolism	Homoserine dehydrogenase
EVM0004656	47.97	20.22	1.25	5.00E-05	4.28E-04	--	ATP adenylyltransferase
EVM0004447	115.94	48.82	1.25	5.00E-05	4.28E-04	--	--

EVM0009453	66.01	27.78	1.25	5.00E-05	4.28E-04	--	Septin
EVM0010439	19.34	8.14	1.25	1.25E-03	6.87E-03	--	--
EVM0000011	229.85	96.69	1.25	5.00E-05	4.28E-04	Posttranslational modification, protein turnover, chaperones	Peptidase family M3
EVM0001930	33.23	13.98	1.25	5.00E-04	3.19E-03	Carbohydrate transport and metabolism	Major Facilitator Superfamily
EVM0012205	15.69	6.60	1.25	1.00E-04	7.96E-04	Function unknown	RHS Repeat
EVM0009531	23.96	10.07	1.25	1.00E-03	5.70E-03	--	Polysaccharide lyase family 8, super-sandwich domain
EVM0005521	142.17	59.70	1.25	2.50E-04	1.77E-03	--	GPR1/FUN34/yaaH family
EVM0009069	808.29	339.37	1.25	1.35E-03	7.33E-03	Carbohydrate transport and metabolism	pfkB family carbohydrate kinase
EVM0009905	137.44	57.65	1.25	5.00E-05	4.28E-04	Carbohydrate transport and metabolism	Metalloenzyme superfamily
EVM0003536	7.72	3.24	1.25	1.20E-03	6.64E-03	--	--
EVM0001774	13.04	5.47	1.25	3.50E-04	2.36E-03	Secondary metabolites biosynthesis, transport and catabolism	Cytochrome P450
EVM0002232	142.20	59.58	1.25	5.75E-03	2.41E-02	Amino acid transport and metabolism	Metallopeptidase family M24

EVM0007171	35.81	15.00	1.26	2.00E-04	1.46E-03	--	--
EVM0001216	109.19	45.71	1.26	5.00E-05	4.28E-04	Energy production and conversion	Citrate synthase
EVM0010340	10.95	4.59	1.26	5.00E-05	4.28E-04	Energy production and conversion	Pyridine nucleotide-disulphide oxidoreductase
EVM0000108	53.59	22.39	1.26	3.50E-04	2.36E-03	--	Amidohydrolase
EVM0011384	89.63	37.42	1.26	5.00E-05	4.28E-04	--	Phosphorylase superfamily
EVM0004548	655.91	273.76	1.26	2.00E-04	1.46E-03	--	Fungal hydrophobin
EVM0001625	226.99	94.73	1.26	1.00E-04	7.96E-04	--	--
EVM0005854	149.62	62.40	1.26	1.50E-04	1.14E-03	Cell wall/membrane/envelope biogenesis	Chitin synthase
EVM0002819	24.17	10.07	1.26	5.00E-05	4.28E-04	Function unknown	Domain of unknown function (DUF3437)
EVM0009169	1408.42	586.51	1.26	2.50E-04	1.77E-03	--	--
EVM0010122	43.17	17.97	1.26	5.00E-05	4.28E-04	Replication, recombination and repair	MutS domain V
EVM0010089	78.42	32.62	1.27	5.00E-05	4.28E-04	--	--

EVM0000340	28.30	11.77	1.27	5.00E-05	4.28E-04	--	PWWP domain
EVM0000100	2461.85	1023.31	1.27	3.00E-04	2.07E-03	Cytoskeleton	Cofilin/tropomyosin-type actin-binding protein
EVM0008628	17.86	7.42	1.27	5.00E-05	4.28E-04	--	--
EVM0011466	33.35	13.86	1.27	5.00E-05	4.28E-04	--	--
EVM0005820	220.36	91.55	1.27	5.00E-05	4.28E-04	General function prediction only	ABC transporter
EVM0006696	52.23	21.69	1.27	3.35E-03	1.55E-02	--	Domain of unknown function DUF77
EVM0007451	78.06	32.40	1.27	5.00E-05	4.28E-04	--	Beta-glucan synthesis-associated protein (SKN1)
EVM0011036	13.71	5.69	1.27	8.00E-04	4.73E-03	--	Polysaccharide deacetylase
EVM0008291	143.56	59.56	1.27	5.00E-05	4.28E-04	Carbohydrate transport and metabolism	NAD dependent epimerase/dehydratase family
EVM0002497	26.46	10.98	1.27	5.00E-05	4.28E-04	--	NACHT domain
EVM0010882	214.64	89.00	1.27	5.00E-05	4.28E-04	General function prediction only	Histidine phosphatase superfamily (branch 2)
EVM0000809	15.23	6.31	1.27	3.50E-04	2.36E-03	General function prediction only	Aldo/keto reductase family
EVM0008146	53.45	22.16	1.27	5.00E-05	4.28E-04	--	--

EVM0008147	9.33	3.87	1.27	3.50E-04	2.36E-03	Nucleotide transport and metabolism	5'-nucleotidase, C-terminal domain
EVM0004729	611.89	253.04	1.27	1.24E-02	4.45E-02	--	Helix-turn-helix
EVM0008140	32.62	13.48	1.28	5.00E-05	4.28E-04	--	--
EVM0007638	870.72	359.34	1.28	1.95E-03	9.93E-03	Coenzyme transport and metabolism	Aminotransferase class I and II
EVM0006684	12.93	5.33	1.28	7.00E-04	4.23E-03	Transcription	C-5 cytosine-specific DNA methylase
EVM0010320	14.34	5.90	1.28	1.50E-04	1.14E-03	--	--
EVM0010314	36.04	14.84	1.28	5.00E-05	4.28E-04	General function prediction only	Major Facilitator Superfamily
EVM0008676	10.03	4.13	1.28	1.50E-04	1.14E-03	--	--
EVM0008860	93.49	38.48	1.28	2.00E-04	1.46E-03	--	Lipase (class 3)
EVM0005732	10.66	4.39	1.28	6.00E-04	3.72E-03	--	NADH:flavin oxidoreductase / NADH oxidase family
EVM0003987	26.91	11.07	1.28	9.60E-03	3.63E-02	--	--
EVM0000613	15.11	6.21	1.28	5.00E-05	4.28E-04	Defense mechanisms	AAA domain

EVM0003191	58.67	24.08	1.28	4.50E-04	2.92E-03	Secondary metabolites biosynthesis, transport and catabolism	Cytochrome P450
EVM0012019	14.39	5.90	1.29	4.50E-04	2.92E-03	--	BTB/POZ domain
EVM0000180	171.85	70.44	1.29	2.00E-04	1.46E-03	--	Protein kinase domain
EVM0004604	96.58	39.58	1.29	2.50E-04	1.77E-03	Signal transduction mechanisms	NUDIX domain
EVM0008945	257.59	105.56	1.29	5.00E-05	4.28E-04	Secondary metabolites biosynthesis, transport and catabolism	Pyridine nucleotide-disulphide oxidoreductase
EVM0005250	32.98	13.51	1.29	5.00E-05	4.28E-04	--	Fungal specific transcription factor domain
EVM0005047	122.55	50.17	1.29	5.00E-05	4.28E-04	--	Protein of unknown function (DUF1688)
EVM0002585	33.80	13.84	1.29	5.00E-05	4.28E-04	--	Phosphoinositide 3-kinase family, accessory domain (PIK domain)
EVM0009712	4.55	1.86	1.29	4.65E-03	2.03E-02	#N/A	#N/A
EVM0001698	53.32	21.82	1.29	1.00E-04	7.96E-04	--	Fatty acid desaturase
EVM0005255	77.57	31.70	1.29	5.00E-05	4.28E-04	Posttranslational modification, protein turnover, chaperones	--

EVM0000743	21.93	8.95	1.29	5.00E-05	4.28E-04	--	FAD binding domain
EVM0010124	93.16	37.99	1.29	5.00E-05	4.28E-04	--	Fungal protein of unknown function (DUF1752)
EVM0004029	115.65	47.02	1.30	5.00E-05	4.28E-04	General function prediction only	TATA-binding protein interacting (TIP20)
EVM0006744	49.64	20.16	1.30	5.00E-05	4.28E-04	General function prediction only	Carboxylesterase family
EVM0006690	55.60	22.58	1.30	5.00E-05	4.28E-04	General function prediction only	Kelch motif
EVM0001336	64.58	26.20	1.30	4.00E-04	2.65E-03	Signal transduction mechanisms	HIT domain
EVM0004175	136.81	55.41	1.30	5.00E-05	4.28E-04	--	Nitronate monooxygenase
EVM0001638	71.60	28.99	1.30	5.00E-05	4.28E-04	Amino acid transport and metabolism	Glutamine synthetase, catalytic domain
EVM0001547	6.86	2.77	1.31	6.00E-04	3.72E-03	--	F-box-like
EVM0005159	101.55	41.01	1.31	5.00E-05	4.28E-04	--	NADH:flavin oxidoreductase / NADH oxidase family
EVM0010194	33.25	13.40	1.31	5.00E-05	4.28E-04	--	Protein of unknown function (DUF4246)
EVM0008466	310.04	124.92	1.31	1.00E-04	7.96E-04	Energy production and conversion	ATP synthase alpha/beta family, nucleotide-binding domain
EVM0010598	4.49	1.81	1.31	2.15E-03	1.08E-02	--	F-box-like

EVM0004594	138.25	55.67	1.31	5.00E-05	4.28E-04	Amino acid transport and metabolism	Amino acid permease
EVM0010923	50.54	20.32	1.31	5.00E-05	4.28E-04	Replication, recombination and repair	FAT domain
EVM0004840	276.31	111.05	1.32	5.00E-05	4.28E-04	--	--
EVM0012163	33.80	13.58	1.32	5.00E-05	4.28E-04	Replication, recombination and repair	DNA replication factor Dna2
EVM0005557	46.12	18.52	1.32	5.00E-05	4.28E-04	--	Nucleoporin subcomplex protein binding to Pom34
EVM0009514	310.89	124.76	1.32	5.00E-05	4.28E-04	Carbohydrate transport and metabolism	Glyceraldehyde 3-phosphate dehydrogenase, C-terminal domain
EVM0003057	30.01	12.02	1.32	3.50E-04	2.36E-03	--	Domain of unknown function (DUF4336)
EVM0000787	31.43	12.59	1.32	2.00E-04	1.46E-03	Posttranslational modification, protein turnover, chaperones	Prenyltransferase and squalene oxidase repeat
EVM0010226	1723.10	689.62	1.32	1.50E-04	1.14E-03	Inorganic ion transport and metabolism	Iron/manganese superoxide dismutases, C-terminal domain
EVM0001562	139.15	55.68	1.32	1.00E-04	7.96E-04	--	Alpha amylase, catalytic domain
EVM0004676	96.48	38.58	1.32	5.00E-05	4.28E-04	Signal transduction mechanisms	RhoGEF domain
EVM0003034	165.29	66.08	1.32	3.00E-04	2.07E-03	Coenzyme transport and metabolism	Formate--tetrahydrofolate ligase

EVM0011178	87.12	34.81	1.32	5.00E-05	4.28E-04	--	--
EVM0003271	2.54	1.02	1.32	4.95E-03	2.13E-02	--	Fungalysin metallopeptidase (M36)
EVM0006280	29.16	11.64	1.32	5.00E-05	4.28E-04	Nucleotide transport and metabolism	5'-nucleotidase, C-terminal domain
EVM0004085	36.84	14.70	1.33	1.26E-02	4.49E-02	Posttranslational modification, protein turnover, chaperones	FKBP-type peptidyl-prolyl cis-trans isomerase
EVM0010728	55.80	22.26	1.33	5.00E-05	4.28E-04	--	--
EVM0000707	109.33	43.60	1.33	5.00E-05	4.28E-04	--	Putative cyclase
EVM0000397	161.44	64.35	1.33	5.00E-05	4.28E-04	--	--
EVM0002286	2.74	1.09	1.33	8.40E-03	3.26E-02	--	Cellulase (glycosyl hydrolase family 5)
EVM0000598	64.58	25.72	1.33	5.00E-05	4.28E-04	Cell cycle control, cell division, chromosome partitioning	N terminus of Rad21 / Rec8 like protein
EVM0002966	213.00	84.82	1.33	5.00E-05	4.28E-04	Lipid transport and metabolism	GHMP kinases N terminal domain
EVM0001850	7.00	2.79	1.33	4.10E-03	1.83E-02	--	--
EVM0009256	59.87	23.82	1.33	5.00E-05	4.28E-04	Function unknown	Vta1 like

EVM0009059	4.28	1.70	1.33	5.70E-03	2.39E-02	General function prediction only	short chain dehydrogenase
EVM0011268	50.07	19.91	1.33	1.00E-04	7.96E-04	--	--
EVM0001151	116.37	46.28	1.33	5.00E-05	4.28E-04	Intracellular trafficking, secretion, and vesicular transport	Exportin 1-like protein
EVM0001683	42.00	16.69	1.33	5.00E-05	4.28E-04	Coenzyme transport and metabolism	Probable molybdopterin binding domain
EVM0011830	32.37	12.86	1.33	4.00E-03	1.79E-02	Intracellular trafficking, secretion, and vesicular transport	Golgi CORVET complex core vacuolar protein 8
EVM0002339	222.02	88.17	1.33	5.00E-05	4.28E-04	--	--
EVM0002146	5.39	2.14	1.33	1.30E-03	7.10E-03	--	Pectinesterase
EVM0009414	127.37	50.54	1.33	5.00E-05	4.28E-04	General function prediction only	Protein kinase domain
EVM0001824	62.13	24.65	1.33	5.00E-05	4.28E-04	General function prediction only	Alpha/beta hydrolase family
EVM0009632	196.77	78.06	1.33	5.00E-05	4.28E-04	Secondary metabolites biosynthesis, transport and catabolism	Copper amine oxidase, enzyme domain
EVM0010885	101.70	40.34	1.33	6.50E-04	3.98E-03	Posttranslational modification, protein turnover, chaperones	BCS1 N terminal

EVM0010842	145.53	57.70	1.33	5.00E-05	4.28E-04	--	Allophanate hydrolase subunit 2
EVM0004671	39.90	15.81	1.34	5.00E-05	4.28E-04	Cytoskeleton	Histidine phosphatase superfamily (branch 2)
EVM0002970	30.67	12.15	1.34	4.00E-04	2.65E-03	--	--
EVM0000767	27.72	10.97	1.34	5.00E-05	4.28E-04	Replication, recombination and repair	MCM2/3/5 family
EVM0002487	23.89	9.46	1.34	5.00E-05	4.28E-04	Signal transduction mechanisms	OPT oligopeptide transporter protein
EVM0004374	318.17	125.80	1.34	5.00E-05	4.28E-04	--	--
EVM0011364	6.37	2.52	1.34	8.50E-04	4.98E-03	--	Jacalin-like lectin domain
EVM0007734	166.16	65.61	1.34	5.00E-05	4.28E-04	Secondary metabolites biosynthesis, transport and catabolism	Pyridine nucleotide-disulphide oxidoreductase
EVM0000512	9.27	3.66	1.34	1.15E-03	6.41E-03	--	--
EVM0006652	57.09	22.49	1.34	1.00E-04	7.96E-04	--	Cytochrome P450
EVM0008895	237.35	93.51	1.34	5.00E-05	4.28E-04	--	Protein kinase domain
EVM0003181	86.97	34.24	1.34	5.00E-05	4.28E-04	--	--

EVM0007738	299.98	118.07	1.35	5.00E-05	4.28E-04	--	Glycosyltransferase sugar-binding region containing DXD motif
EVM0001400	39.64	15.59	1.35	5.00E-05	4.28E-04	--	PAS fold
EVM0006416	115.73	45.48	1.35	5.00E-05	4.28E-04	Secondary metabolites biosynthesis, transport and catabolism	Alcohol dehydrogenase GroES-like domain
EVM0008722	155.69	61.14	1.35	5.00E-05	4.28E-04	Inorganic ion transport and metabolism	Alkaline phosphatase
EVM0000439	83.27	32.69	1.35	5.00E-05	4.28E-04	--	Kelch motif
EVM0001362	5.96	2.34	1.35	2.00E-04	1.46E-03	--	Protein of unknown function (DUF1212)
EVM0006629	45.38	17.78	1.35	5.00E-05	4.28E-04	Secondary metabolites biosynthesis, transport and catabolism	ABC transporter
EVM0002137	46.93	18.39	1.35	1.00E-04	7.96E-04	Defense mechanisms	D-arabinono-1,4-lactone oxidase
EVM0002953	180.59	70.64	1.35	7.00E-04	4.23E-03	--	Fungalysin metallopeptidase (M36)
EVM0001594	51.23	20.01	1.36	5.00E-05	4.28E-04	Secondary metabolites biosynthesis, transport and catabolism	short chain dehydrogenase
EVM0010443	11.39	4.45	1.36	3.50E-04	2.36E-03	--	Zinc finger C-x8-C-x5-C-x3-H type (and similar)

EVM0005161	48.06	18.76	1.36	5.00E-05	4.28E-04	Secondary metabolites biosynthesis, transport and catabolism	ABC transporter
EVM0008808	12.00	4.68	1.36	1.00E-03	5.70E-03	General function prediction only	Haloacid dehalogenase-like hydrolase
EVM0005662	85.91	33.51	1.36	5.00E-05	4.28E-04	Nucleotide transport and metabolism	AICARFT/IMPCHase bienzyme
EVM0000868	32.50	12.68	1.36	5.00E-05	4.28E-04	--	Aconitase family (aconitate hydratase)
EVM0008744	55.28	21.52	1.36	5.00E-05	4.28E-04	RNA processing and modification	MIF4G domain
EVM0004686	6.77	2.63	1.36	2.50E-04	1.77E-03	General function prediction only	Major Facilitator Superfamily
EVM0009644	25.43	9.90	1.36	1.40E-03	7.55E-03	--	--
EVM0000355	937.39	364.70	1.36	1.55E-03	8.22E-03	--	--
EVM0000257	8.31	3.23	1.36	3.05E-03	1.44E-02	--	--
EVM0007372	21.23	8.25	1.36	5.00E-05	4.28E-04	--	--
EVM0009793	5.79	2.25	1.36	3.40E-03	1.57E-02	--	--
EVM0011123	49.31	19.16	1.36	5.00E-05	4.28E-04	Posttranslational modification, protein turnover, chaperones	Insulinase (Peptidase family M16)

EVM0005413	201.98	78.47	1.36	5.00E-05	4.28E-04	--	Possible lysine decarboxylase
EVM0000054	8.73	3.39	1.36	2.00E-04	1.46E-03	Secondary metabolites biosynthesis, transport and catabolism	Cytochrome P450
EVM0004984	70.98	27.55	1.37	5.00E-05	4.28E-04	--	--
EVM0004330	1700.54	660.07	1.37	7.20E-03	2.88E-02	--	--
EVM0001877	4.08	1.58	1.37	2.35E-03	1.16E-02	--	Voltage-dependent anion channel
EVM0009565	1575.72	610.93	1.37	2.75E-03	1.32E-02	Posttranslational modification, protein turnover, chaperones	AAA domain (Cdc48 subfamily)
EVM0011313	7.34	2.84	1.37	5.00E-05	4.28E-04	--	--
EVM0011981	30.89	11.94	1.37	5.00E-05	4.28E-04	Amino acid transport and metabolism	Aldehyde dehydrogenase family
EVM0008948	17.26	6.67	1.37	1.00E-04	7.96E-04	--	BTB/POZ domain
EVM0003929	13.23	5.11	1.37	1.00E-04	7.96E-04	--	Taurine catabolism dioxygenase TauD, TfdA family
EVM0000879	21.91	8.46	1.37	5.00E-05	4.28E-04	Carbohydrate transport and metabolism	GHMP kinases C terminal
EVM0004924	59.94	23.14	1.37	1.29E-02	4.57E-02	--	--

EVM0002506	68.64	26.49	1.37	1.50E-04	1.14E-03	Inorganic ion transport and metabolism	Natural resistance-associated macrophage protein
EVM0007541	68.55	26.40	1.38	5.00E-05	4.28E-04	Signal transduction mechanisms	RhoGAP domain
EVM0011121	145.48	56.01	1.38	1.65E-03	8.65E-03	--	--
EVM0007741	32.26	12.41	1.38	5.00E-05	4.28E-04	--	SNF2 family N-terminal domain
EVM0011967	77.17	29.68	1.38	5.00E-05	4.28E-04	--	--
EVM0009469	72.77	27.95	1.38	5.00E-05	4.28E-04	--	--
EVM0008799	54.39	20.87	1.38	5.00E-05	4.28E-04	--	Right handed beta helix region
EVM0005856	620.84	238.21	1.38	5.00E-05	4.28E-04	Translation, ribosomal structure and biogenesis	eIF-6 family
EVM0011151	643.28	246.79	1.38	5.00E-05	4.28E-04	Secondary metabolites biosynthesis, transport and catabolism	Cytochrome P450
EVM0004822	84.00	32.21	1.38	5.00E-05	4.28E-04	--	--
EVM0007677	32.69	12.53	1.38	9.50E-04	5.46E-03	Secondary metabolites biosynthesis, transport and catabolism	AMP-binding enzyme
EVM0008679	7.51	2.88	1.38	5.00E-05	4.28E-04	Signal transduction mechanisms	RhoGEF domain

EVM0004285	29.82	11.41	1.39	5.00E-05	4.28E-04	Secondary metabolites biosynthesis, transport and catabolism	Multicopper oxidase
EVM0005480	267.81	102.49	1.39	5.00E-05	4.28E-04	--	--
EVM0011143	76.16	29.14	1.39	5.00E-05	4.28E-04	RNA processing and modification	DEAD/DEAH box helicase
EVM0007116	2.45	0.94	1.39	3.65E-03	1.66E-02	Cytoskeleton	Misato Segment II tubulin-like domain
EVM0006017	32.12	12.29	1.39	5.00E-05	4.28E-04	--	Uncharacterized alpha/beta hydrolase domain (DUF2235)
EVM0008073	44.07	16.85	1.39	5.00E-05	4.28E-04	Amino acid transport and metabolism	Glycosyl transferase family, a/b domain
EVM0000955	97.99	37.43	1.39	5.00E-05	4.28E-04	General function prediction only	Ankyrin repeats (many copies)
EVM0011070	9.76	3.73	1.39	5.00E-05	4.28E-04	Function unknown	Domain of unknown function (DUF1741)
EVM0004356	5.31	2.03	1.39	7.00E-03	2.82E-02	General function prediction only	Dienelactone hydrolase family
EVM0001377	336.39	128.40	1.39	3.50E-04	2.36E-03	Cell wall/membrane/envelope biogenesis	1,3-beta-glucan synthase component
EVM0008337	72.85	27.80	1.39	5.00E-05	4.28E-04	General function prediction only	--
EVM0007364	65.71	25.05	1.39	5.00E-05	4.28E-04	Lipid transport and metabolism	alpha/beta hydrolase fold

EVM0008673	64.50	24.57	1.39	5.00E-05	4.28E-04	--	Sir2 family
EVM0005213	39.56	15.07	1.39	5.00E-05	4.28E-04	Nucleotide transport and metabolism	Lyase
EVM0009891	84.77	32.29	1.39	5.00E-05	4.28E-04	--	Fungal Zn(2)-Cys(6) binuclear cluster domain
EVM0006969	128.89	49.07	1.39	5.00E-05	4.28E-04	--	--
EVM0008943	2.14	0.81	1.39	2.70E-03	1.30E-02	Signal transduction mechanisms	Protein kinase domain
EVM0011893	1962.61	745.90	1.40	6.65E-03	2.70E-02	Posttranslational modification, protein turnover, chaperones	Subtilase family
EVM0002766	427.37	162.41	1.40	5.00E-05	4.28E-04	Energy production and conversion	Citrate synthase
EVM0006221	19.23	7.27	1.40	1.50E-04	1.14E-03	Signal transduction mechanisms	Protein kinase domain
EVM0009234	28.85	10.90	1.40	5.00E-05	4.28E-04	--	Enolase C-terminal domain-like
EVM0010136	131.96	49.87	1.40	5.00E-05	4.28E-04	Carbohydrate transport and metabolism	Major Facilitator Superfamily
EVM0008227	34.62	13.08	1.40	5.00E-05	4.28E-04	Replication, recombination and repair	MCM2/3/5 family
EVM0005148	440.96	166.50	1.41	5.00E-05	4.28E-04	Carbohydrate transport and metabolism	6-phosphogluconate dehydrogenase, C-terminal domain

EVM0002033	5.91	2.23	1.41	9.80E-03	3.69E-02	#N/A	#N/A
EVM0004859	36.11	13.61	1.41	5.00E-05	4.28E-04	General function prediction only	short chain dehydrogenase
EVM0007330	189.16	71.27	1.41	5.00E-05	4.28E-04	--	--
EVM0004424	38.60	14.53	1.41	5.00E-05	4.28E-04	Amino acid transport and metabolism	Histidinol dehydrogenase
EVM0005149	64.22	24.17	1.41	2.50E-04	1.77E-03	--	--
EVM0005418	149.78	56.37	1.41	1.05E-02	3.88E-02	Translation, ribosomal structure and biogenesis	tRNA synthetases class I (I, L, M and V)
EVM0007721	42.06	15.82	1.41	2.50E-04	1.77E-03	--	--
EVM0009501	137.46	51.67	1.41	5.00E-05	4.28E-04	Function unknown	Eukaryotic protein of unknown function (DUF914)
EVM0000536	6.66	2.50	1.42	6.10E-03	2.52E-02	--	--
EVM0009861	155.36	58.22	1.42	5.00E-05	4.28E-04	Signal transduction mechanisms	Protein kinase domain
EVM0008382	43.40	16.25	1.42	5.00E-05	4.28E-04	General function prediction only	WD domain, G-beta repeat
EVM0004947	16.52	6.18	1.42	5.00E-05	4.28E-04	--	--
EVM0008890	18.59	6.96	1.42	5.00E-05	4.28E-04	RNA processing and modification	Sec63 Brl domain

EVM0002163	25.71	9.62	1.42	5.00E-05	4.28E-04	--	HEAT repeat
EVM0003788	1.68	0.63	1.42	1.17E-02	4.23E-02	--	--
EVM0002782	34.88	13.02	1.42	5.00E-05	4.28E-04	--	--
EVM0007615	63.74	23.78	1.42	5.00E-05	4.28E-04	Function unknown	HEAT repeats
EVM0008747	43.59	16.24	1.42	5.00E-05	4.28E-04	General function prediction only	Metallo-beta-lactamase superfamily
EVM0005193	42.57	15.85	1.43	1.00E-04	7.96E-04	--	Acetyltransferase (GNAT) domain
EVM0008518	492.11	183.18	1.43	2.00E-04	1.46E-03	--	Carboxymuconolactone decarboxylase family
EVM0007587	145.28	54.07	1.43	1.20E-03	6.64E-03	--	--
EVM0004929	40.10	14.90	1.43	1.50E-04	1.14E-03	General function prediction only	Ras family
EVM0010133	84.68	31.46	1.43	5.00E-05	4.28E-04	Amino acid transport and metabolism	Aminotransferase class I and II
EVM0003868	149.62	55.57	1.43	5.00E-05	4.28E-04	--	Peptidase inhibitor I9
EVM0008570	85.08	31.57	1.43	2.00E-03	1.01E-02	--	--
EVM0000728	41.80	15.51	1.43	5.00E-05	4.28E-04	--	50S ribosome-binding GTPase

EVM0004678	22.30	8.27	1.43	5.00E-05	4.28E-04	--	Cellulase (glycosyl hydrolase family 5)
EVM0001405	169.09	62.70	1.43	5.00E-05	4.28E-04	Intracellular trafficking, secretion, and vesicular transport	Sybindin-like family
EVM0000233	26.50	9.81	1.43	5.00E-05	4.28E-04	Intracellular trafficking, secretion, and vesicular transport	Domain of unknown function in PX-proteins (DUF3818)
EVM0002222	9.31	3.44	1.44	5.00E-04	3.19E-03	--	Cytochrome P450
EVM0004850	10.43	3.86	1.44	5.00E-04	3.19E-03	--	Semialdehyde dehydrogenase, NAD binding domain
EVM0008555	7.10	2.62	1.44	5.00E-05	4.28E-04	--	SNF2 family N-terminal domain
EVM0010372	12.25	4.53	1.44	5.00E-05	4.28E-04	Signal transduction mechanisms	Histidine kinase-, DNA gyrase B-, and HSP90-like ATPase
EVM0000075	23.82	8.80	1.44	5.00E-05	4.28E-04	--	F-box domain
EVM0001004	96.97	35.80	1.44	2.50E-04	1.77E-03	--	TAP-like protein
EVM0010749	8.23	3.04	1.44	4.50E-04	2.92E-03	Secondary metabolites biosynthesis, transport and catabolism	Cytochrome P450
EVM0008878	15.41	5.68	1.44	1.01E-02	3.78E-02	--	--

EVM0003012	120.92	44.56	1.44	5.00E-05	4.28E-04	--	--
EVM0009659	179.58	66.17	1.44	5.00E-05	4.28E-04	Posttranslational modification, protein turnover, chaperones	ICP0-binding domain of Ubiquitin-specific protease 7
EVM0009401	42.30	15.57	1.44	5.00E-05	4.28E-04	Transcription	--
EVM0010001	30.49	11.22	1.44	3.95E-03	1.77E-02	Translation, ribosomal structure and biogenesis	Endoribonuclease L-PSP
EVM0004812	213.61	78.61	1.44	5.00E-05	4.28E-04	--	Arrestin (or S-antigen), N-terminal domain
EVM0001810	283.77	104.23	1.45	5.00E-05	4.28E-04	Carbohydrate transport and metabolism	Major intrinsic protein
EVM0001083	48.97	17.98	1.45	5.00E-05	4.28E-04	Lipid transport and metabolism	short chain dehydrogenase
EVM0008748	48.27	17.70	1.45	5.00E-05	4.28E-04	--	--
EVM0008924	849.90	311.04	1.45	3.50E-04	2.36E-03	Translation, ribosomal structure and biogenesis	Elongation factor Tu GTP binding domain
EVM0000179	3.30	1.21	1.45	1.40E-03	7.55E-03	Secondary metabolites biosynthesis, transport and catabolism	Cytochrome P450
EVM0008805	49.99	18.28	1.45	5.00E-05	4.28E-04	--	--
EVM0004568	309.51	113.16	1.45	5.00E-05	4.28E-04	--	Cellulase (glycosyl hydrolase family 5)

EVM0012111	59.25	21.66	1.45	5.00E-05	4.28E-04	--	--
EVM0011240	299.49	109.41	1.45	5.00E-05	4.28E-04	--	--
EVM0006789	131.04	47.82	1.45	5.00E-05	4.28E-04	Posttranslational modification, protein turnover, chaperones	Eukaryotic aspartyl protease
EVM0012190	6.88	2.51	1.46	1.10E-03	6.17E-03	Secondary metabolites biosynthesis, transport and catabolism	ABC transporter transmembrane region
EVM0011846	159.65	58.16	1.46	5.00E-05	4.28E-04	Posttranslational modification, protein turnover, chaperones	Glutathione S-transferase, N-terminal domain
EVM0004518	26.30	9.58	1.46	5.00E-05	4.28E-04	Energy production and conversion	Aldehyde dehydrogenase family
EVM0000503	486.53	177.15	1.46	5.00E-05	4.28E-04	Energy production and conversion	lactate/malate dehydrogenase, NAD binding domain
EVM0010175	142.28	51.80	1.46	5.00E-05	4.28E-04	Posttranslational modification, protein turnover, chaperones	Eukaryotic aspartyl protease
EVM0009960	4.17	1.52	1.46	9.20E-03	3.51E-02	--	--
EVM0000440	63.54	23.12	1.46	5.00E-05	4.28E-04	--	Glutathione S-transferase, N-terminal domain
EVM0006479	19.38	7.04	1.46	5.00E-05	4.28E-04	--	RecF/RecN/SMC N terminal domain

EVM0002184	1535.71	557.60	1.46	2.00E-04	1.46E-03	General function prediction only	Sugar (and other) transporter
EVM0004235	60.80	22.06	1.46	5.00E-05	4.28E-04	--	--
EVM0006076	925.97	335.99	1.46	3.95E-03	1.77E-02	--	--
EVM0010043	162.64	58.99	1.46	5.00E-05	4.28E-04	Carbohydrate transport and metabolism	Fructose-bisphosphate aldolase class-II
EVM0004098	137.15	49.69	1.46	5.00E-05	4.28E-04	Signal transduction mechanisms	Protein kinase domain
EVM0002114	182.00	65.92	1.47	5.00E-05	4.28E-04	Posttranslational modification, protein turnover, chaperones	Proteasome/cyclosome repeat
EVM0007201	38.52	13.93	1.47	5.00E-05	4.28E-04	--	--
EVM0010942	43.72	15.79	1.47	5.00E-05	4.28E-04	--	--
EVM0002736	87.69	31.56	1.47	5.00E-05	4.28E-04	Cytoskeleton	Myosin head (motor domain)
EVM0005404	22.41	8.05	1.48	5.00E-05	4.28E-04	--	RecF/RecN/SMC N terminal domain
EVM0001842	83.01	29.81	1.48	5.00E-05	4.28E-04	--	--
EVM0010878	35.52	12.74	1.48	5.00E-05	4.28E-04	--	--

EVM0001066	21.88	7.84	1.48		5.00E-05	4.28E-04	--	FAD binding domain
EVM0008834	120.63	43.20	1.48		5.00E-05	4.28E-04	--	Pirin
EVM0001225	23.87	8.55	1.48		1.80E-03	9.29E-03	--	CoA binding domain
EVM0009637	39.19	14.02	1.48		5.00E-05	4.28E-04	General function prediction only	Epoxide hydrolase N terminus
EVM0003184	47.21	16.87	1.48		5.00E-05	4.28E-04	Intracellular trafficking, secretion, and vesicular transport	Sec34-like family
EVM0002464	98.47	35.18	1.48		5.00E-05	4.28E-04	Amino acid transport and metabolism	Aminotransferase class I and II
EVM0002457	34.01	12.14	1.49		5.00E-05	4.28E-04	Coenzyme transport and metabolism	Biotin-protein ligase, N terminal
EVM0002502	375.73	134.15	1.49		5.00E-05	4.28E-04	--	Uncharacterized alpha/beta hydrolase domain (DUF2235)
EVM0006860	158.19	56.47	1.49		5.00E-05	4.28E-04	Function unknown	AMMECR1
EVM0005902	53.38	19.03	1.49		5.00E-05	4.28E-04	--	--
EVM0006566	52.76	18.80	1.49		5.00E-05	4.28E-04	--	Protein of unknown function (DUF3533)
EVM0006594	689.09	245.31	1.49		5.00E-05	4.28E-04	Amino acid transport and metabolism	Serine hydroxymethyltransferase

EVM0008978	13.31	4.73	1.49	5.00E-05	4.28E-04	Transcription	SNF2 family N-terminal domain
EVM0003698	103.03	36.61	1.49	5.00E-05	4.28E-04	--	Alginate lyase
EVM0001895	41.79	14.85	1.49	5.00E-05	4.28E-04	--	--
EVM0001446	203.32	72.08	1.50	5.00E-05	4.28E-04	Carbohydrate transport and metabolism	Phosphoglucose isomerase
EVM0006648	10.86	3.84	1.50	1.00E-04	7.96E-04	--	--
EVM0006693	2.70	0.95	1.50	1.15E-03	6.41E-03	--	O-Glycosyl hydrolase family 30
EVM0010152	24.77	8.75	1.50	3.00E-04	2.07E-03	--	--
EVM0005752	102.09	35.99	1.50	5.00E-05	4.28E-04	--	S1/P1 Nuclease
EVM0011216	106.00	37.30	1.51	5.00E-05	4.28E-04	--	Oxidoreductase family, NAD-binding Rossmann fold
EVM0000713	23.54	8.28	1.51	5.00E-05	4.28E-04	--	ABC transporter
EVM0009500	155.93	54.85	1.51	5.00E-05	4.28E-04	--	Domain of unknown function (DUF1708)
EVM0000530	27.77	9.76	1.51	5.00E-05	4.28E-04	Function unknown	Ribosome 60S biogenesis N-terminal
EVM0009456	97.87	34.40	1.51	5.00E-05	4.28E-04	--	--

EVM0011005	20.73	7.28	1.51	5.00E-05	4.28E-04	--	Polysaccharide deacetylase
EVM0002522	17.13	6.02	1.51	1.00E-04	7.96E-04	--	Protein of unknown function (DUF1399)
EVM0003905	166.20	58.32	1.51	5.00E-05	4.28E-04	--	Domain of unknown function (DUF4646)
EVM0000981	43.74	15.31	1.51	9.50E-04	5.46E-03	--	--
EVM0008259	843.42	294.90	1.52	1.00E-04	7.96E-04	--	Putative oxidoreductase C terminal
EVM0007795	70.79	24.74	1.52	5.00E-05	4.28E-04	Secondary metabolites biosynthesis, transport and catabolism	Cytochrome P450
EVM0004154	5.68	1.99	1.52	4.70E-03	2.05E-02	--	--
EVM0006941	10.17	3.55	1.52	5.00E-05	4.28E-04	Nucleotide transport and metabolism	Inosine-uridine preferring nucleoside hydrolase
EVM0005845	29.53	10.30	1.52	5.00E-05	4.28E-04	--	Domain of unknown function (DUF4470)
EVM0007194	64.63	22.50	1.52	5.00E-05	4.28E-04	--	Major Facilitator Superfamily
EVM0003437	25.10	8.74	1.52	5.00E-05	4.28E-04	Posttranslational modification, protein turnover, chaperones	Tubulin folding cofactor D C terminal
EVM0007887	2305.86	802.44	1.52	5.00E-05	4.28E-04	Cytoskeleton	Actin

EVM0012158	47.59	16.56	1.52	5.00E-05	4.28E-04	--	Importin-beta N-terminal domain
EVM0005468	58.31	20.28	1.52	5.00E-05	4.28E-04	Amino acid transport and metabolism	Pyrroline-5-carboxylate reductase dimerisation
EVM0001712	136.61	47.50	1.52	5.00E-05	4.28E-04	Carbohydrate transport and metabolism	Inositol monophosphatase family
EVM0000751	25.92	9.00	1.53	5.00E-05	4.28E-04	Replication, recombination and repair	Ku70/Ku80 beta-barrel domain
EVM0001822	3.56	1.23	1.53	1.60E-03	8.44E-03	--	--
EVM0010844	43.61	15.14	1.53	5.00E-05	4.28E-04	--	Polysaccharide deacetylase
EVM0011500	41.88	14.53	1.53	5.00E-05	4.28E-04	General function prediction only	Putative esterase
EVM0009790	45.44	15.77	1.53	5.00E-05	4.28E-04	Secondary metabolites biosynthesis, transport and catabolism	ABC transporter
EVM0006262	108.63	37.68	1.53	5.00E-05	4.28E-04	--	Peptidase family M1
EVM0006252	30.18	10.45	1.53	5.00E-05	4.28E-04	--	Protein of unknown function (DUF2454)
EVM0009942	30.02	10.39	1.53	5.00E-05	4.28E-04	Transcription	Domain of unknown function (DUF3535)
EVM0011910	60.21	20.81	1.53	5.00E-05	4.28E-04	Intracellular trafficking, secretion, and vesicular transport	VID27 cytoplasmic protein

EVM0001972	32.24	11.14	1.53	6.25E-03	2.57E-02	--	--
EVM0004570	62.59	21.63	1.53	5.00E-05	4.28E-04	--	Domain of unknown function (DUF4470)
EVM0005478	9.21	3.18	1.53	5.00E-05	4.28E-04	General function prediction only	WD domain, G-beta repeat
EVM0007666	62.22	21.46	1.54	5.00E-05	4.28E-04	Cytoskeleton	Myosin head (motor domain)
EVM0007606	32.98	11.35	1.54	5.00E-05	4.28E-04	Energy production and conversion	Malic enzyme, NAD binding domain
EVM0007757	37.95	13.05	1.54	5.00E-05	4.28E-04	--	ABC transporter
EVM0010801	15.75	5.41	1.54	5.00E-05	4.28E-04	--	--
EVM0009831	33.45	11.45	1.55	5.00E-05	4.28E-04	--	--
EVM0008720	208.73	71.42	1.55	5.00E-05	4.28E-04	Nucleotide transport and metabolism	Ribonucleotide reductase, barrel domain
EVM0010648	58.55	20.01	1.55	5.00E-05	4.28E-04	--	Zinc-binding dehydrogenase
EVM0004575	187.92	64.19	1.55	5.00E-05	4.28E-04	General function prediction only	GMC oxidoreductase
EVM0010884	280.99	95.94	1.55	5.00E-05	4.28E-04	--	F-box-like
EVM0001064	1.49	0.51	1.55	1.42E-02	4.94E-02	--	--

EVM0011306	2.37	0.81	1.55	8.00E-03	3.13E-02	--	--
EVM0002277	6.83	2.33	1.55	5.00E-05	4.28E-04	--	Spc97 / Spc98 family
EVM0007667	273.96	93.46	1.55	5.00E-05	4.28E-04	--	Ubiquitin-2 like Rad60 SUMO-like
EVM0010291	25.83	8.79	1.55	1.05E-03	5.94E-03	--	MAPEG family
EVM0010924	18.69	6.36	1.56	2.00E-04	1.46E-03	--	Putative amidotransferase
EVM0006605	105.69	35.94	1.56	5.00E-05	4.28E-04	Secondary metabolites biosynthesis, transport and catabolism	Alcohol dehydrogenase GroES-like domain
EVM0011665	62.91	21.37	1.56	5.00E-05	4.28E-04	Signal transduction mechanisms	Protein kinase domain
EVM0007035	44.51	15.12	1.56	5.00E-05	4.28E-04	--	Transferrin receptor-like dimerisation domain
EVM0007579	214.68	72.77	1.56	5.00E-05	4.28E-04	--	HEAT repeat
EVM0007599	21.07	7.14	1.56	1.50E-04	1.14E-03	--	--
EVM0005145	38.58	13.03	1.57	2.35E-03	1.16E-02	--	--
EVM0002999	53.99	18.24	1.57	5.00E-05	4.28E-04	Intracellular trafficking, secretion, and vesicular transport	ATG C terminal domain

EVM0004740	49.66	16.77	1.57	5.00E-05	4.28E-04	Energy production and conversion	Iron-containing alcohol dehydrogenase
EVM0002918	78.36	26.44	1.57	5.00E-05	4.28E-04	--	Cytochrome P450
EVM0004056	38.79	13.08	1.57	1.50E-04	1.14E-03	--	--
EVM0005495	4.70	1.58	1.57	1.25E-03	6.87E-03	--	Ketopantoate reductase PanE/ApbA C terminal
EVM0009599	3.50	1.18	1.57	3.50E-04	2.36E-03	--	TPR repeat
EVM0005574	20.69	6.93	1.58	5.00E-05	4.28E-04	RNA processing and modification	RNA dependent RNA polymerase
EVM0001966	44.90	15.03	1.58	5.00E-05	4.28E-04	General function prediction only	Major Facilitator Superfamily
EVM0004436	8.61	2.88	1.58	4.00E-04	2.65E-03	--	Acetamidase/Formamidase family
EVM0003073	32.39	10.81	1.58	5.00E-05	4.28E-04	--	--
EVM0006112	26.01	8.67	1.58	5.00E-05	4.28E-04	Carbohydrate transport and metabolism	Glycosyl hydrolases family 18
EVM0009134	32.73	10.91	1.59	5.00E-05	4.28E-04	--	Domain of unknown function (DUF4139)
EVM0000235	12.89	4.29	1.59	5.00E-05	4.28E-04	--	Voltage-dependent anion channel
EVM0010191	10.36	3.44	1.59	1.00E-04	7.96E-04	--	--

EVM0006223	1.57	0.52	1.59	9.75E-03	3.67E-02	--	--
EVM0012052	643.55	213.61	1.59	5.00E-05	4.28E-04	--	Cerato-platanin
EVM0012202	2781.27	922.93	1.59	2.20E-03	1.10E-02	--	--
EVM0003976	127.76	42.32	1.59	5.00E-05	4.28E-04	Amino acid transport and metabolism	Conserved region in glutamate synthase
EVM0003655	16.88	5.59	1.59	5.00E-05	4.28E-04	--	Uncharacterized alpha/beta hydrolase domain (DUF2235)
EVM0002129	9.68	3.20	1.60	5.00E-05	4.28E-04	Carbohydrate transport and metabolism	Major Facilitator Superfamily
EVM0011347	64.93	21.47	1.60	5.00E-05	4.28E-04	Carbohydrate transport and metabolism	Alpha amylase, catalytic domain
EVM0007366	43.49	14.38	1.60	5.00E-05	4.28E-04	--	--
EVM0004566	40.94	13.49	1.60	3.20E-03	1.49E-02	--	Domain of unknown function (DUF718)
EVM0000460	36.45	12.01	1.60	5.00E-05	4.28E-04	Inorganic ion transport and metabolism	Sodium/calcium exchanger protein
EVM0006562	3531.04	1161.26	1.60	5.00E-05	4.28E-04	Posttranslational modification, protein turnover, chaperones	Glutaredoxin
EVM0007265	5.29	1.74	1.60	2.20E-03	1.10E-02	--	--

EVM0006301	123.07	40.33	1.61	5.00E-05	4.28E-04	Carbohydrate transport and metabolism	Amidohydrolase family
EVM0006122	26.98	8.83	1.61	8.35E-03	3.24E-02	--	--
EVM0006552	7.61	2.49	1.61	5.00E-05	4.28E-04	Secondary metabolites biosynthesis, transport and catabolism	Male sterility protein
EVM0001890	6.00	1.96	1.61	1.25E-02	4.46E-02	--	--
EVM0011292	916.28	298.83	1.62	5.00E-05	4.28E-04	Carbohydrate transport and metabolism	Phosphoglycerate kinase
EVM0000747	12.66	4.13	1.62	1.00E-04	7.96E-04	General function prediction only	Scavenger mRNA decapping enzyme C-term binding
EVM0011675	26.10	8.51	1.62	5.00E-05	4.28E-04	Cytoskeleton	Dynein heavy chain and region D6 of dynein motor
EVM0002643	321.50	104.49	1.62	1.00E-04	7.96E-04	--	--
EVM0005728	2.05	0.67	1.63	5.75E-03	2.41E-02	General function prediction only	Histidine phosphatase superfamily (branch 2)
EVM0011309	1245.33	402.88	1.63	5.00E-05	4.28E-04	--	--
EVM0008061	119.36	38.59	1.63	5.00E-05	4.28E-04	General function prediction only	GMC oxidoreductase
EVM0003014	225.46	72.82	1.63	5.00E-05	4.28E-04	Amino acid transport and metabolism	Methylenetetrahydrofolate reductase

EVM0007298	49.54	16.00	1.63	5.00E-05	4.28E-04	General function prediction only	Major Facilitator Superfamily
EVM0003673	4.11	1.33	1.63	1.75E-03	9.08E-03	--	Terpene synthase family, metal binding domain
EVM0003731	175.04	56.41	1.63	5.00E-05	4.28E-04	General function prediction only	RNA recognition motif. (a.k.a. RRM, RBD, or RNP domain)
EVM0005975	128.60	41.44	1.63	5.00E-05	4.28E-04	Secondary metabolites biosynthesis, transport and catabolism	Flavin-binding monooxygenase-like
EVM0002084	159.28	51.15	1.64	5.00E-05	4.28E-04	--	--
EVM0002124	96.72	31.02	1.64	5.00E-05	4.28E-04	Amino acid transport and metabolism	Dehydratase family
EVM0006345	150.46	48.23	1.64	5.00E-05	4.28E-04	Signal transduction mechanisms	HAMP domain
EVM0011448	71.22	22.81	1.64	5.00E-05	4.28E-04	Carbohydrate transport and metabolism	glucanotransferase domain of human glycogen debranching enzyme
EVM0002201	90.85	29.05	1.65	5.00E-05	4.28E-04	Energy production and conversion	Aldehyde dehydrogenase family
EVM0001466	13.74	4.38	1.65	5.00E-05	4.28E-04	Posttranslational modification, protein turnover, chaperones	Peptidase family M48
EVM0012149	94.14	30.02	1.65	5.00E-05	4.28E-04	--	Beta-lactamase superfamily domain

EVM0001953	8.91	2.84	1.65	5.00E-05	4.28E-04	--	Vacuole effluxer Atg22 like
EVM0000709	69.70	22.17	1.65	1.75E-03	9.08E-03	--	--
EVM0000418	196.30	62.40	1.65	5.00E-05	4.28E-04	Posttranslational modification, protein turnover, chaperones	Isoprenylcysteine carboxyl methyltransferase (ICMT) family
EVM0008782	19.90	6.32	1.65	5.00E-05	4.28E-04	Replication, recombination and repair	MCM2/3/5 family
EVM0007311	65.55	20.78	1.66	5.00E-05	4.28E-04	Secondary metabolites biosynthesis, transport and catabolism	Cytochrome P450
EVM0003090	115.35	36.53	1.66	5.00E-05	4.28E-04	Carbohydrate transport and metabolism	Major Facilitator Superfamily
EVM0008798	7.96	2.52	1.66	5.00E-05	4.28E-04	--	Gti1/Pac2 family
EVM0010929	98.98	31.28	1.66	5.00E-05	4.28E-04	--	--
EVM0010014	237.79	75.12	1.66	5.00E-05	4.28E-04	General function prediction only	Calcineurin-like phosphoesterase
EVM0003134	32.98	10.41	1.66	5.00E-05	4.28E-04	--	--
EVM0009536	160.50	50.51	1.67	5.00E-05	4.28E-04	Amino acid transport and metabolism	Cys/Met metabolism PLP-dependent enzyme

EVM0002052	384.96	121.13	1.67	5.00E-05	4.28E-04	Carbohydrate transport and metabolism	Phosphoglucomutase/phosphomannomutase, alpha/beta/alpha domain I
EVM0005683	3.94	1.24	1.67	1.00E-04	7.96E-04	General function prediction only	Major Facilitator Superfamily
EVM0006582	12.30	3.86	1.67	5.00E-05	4.28E-04	Signal transduction mechanisms	Protein kinase domain
EVM0007996	11.95	3.74	1.68	6.50E-04	3.98E-03	Posttranslational modification, protein turnover, chaperones	Isoprenylcysteine carboxyl methyltransferase (ICMT) family
EVM0004844	34.34	10.72	1.68	5.00E-05	4.28E-04	--	Domain of unknown function (DUF4139)
EVM0005816	209.67	65.46	1.68	5.00E-05	4.28E-04	Posttranslational modification, protein turnover, chaperones	Eukaryotic aspartyl protease
EVM0001482	5.51	1.72	1.68	8.00E-04	4.73E-03	Lipid transport and metabolism	short chain dehydrogenase
EVM0002733	64.63	20.17	1.68	5.00E-05	4.28E-04	Lipid transport and metabolism	Calcineurin-like phosphoesterase
EVM0007160	6.43	2.00	1.68	5.00E-05	4.28E-04	--	--
EVM0010697	247.64	77.11	1.68	4.00E-04	2.65E-03	--	--
EVM0006577	211.87	65.95	1.68	5.00E-05	4.28E-04	Energy production and conversion	Aldo/keto reductase family

EVM0003496	3.24	1.01	1.68	1.10E-03	6.17E-03	--	--
EVM0005291	19.42	6.04	1.69	5.00E-05	4.28E-04	--	--
EVM0006660	711.10	220.73	1.69	1.00E-04	7.96E-04	--	--
EVM0005925	21.96	6.81	1.69	5.00E-05	4.28E-04	--	Cytochrome P450
EVM0006160	150.10	46.51	1.69	5.00E-05	4.28E-04	Function unknown	Major Facilitator Superfamily
EVM0004296	13.35	4.13	1.69	6.95E-03	2.80E-02	--	--
EVM0004968	45.68	14.13	1.69	5.00E-05	4.28E-04	Defense mechanisms	Dual specificity phosphatase, catalytic domain
EVM0002674	4.93	1.53	1.69	1.20E-03	6.64E-03	Secondary metabolites biosynthesis, transport and catabolism	short chain dehydrogenase
EVM0010839	116.55	36.04	1.69	5.00E-05	4.28E-04	--	Heterokaryon incompatibility protein Het-C
EVM0007855	145.70	44.99	1.70	5.00E-05	4.28E-04	--	C2 domain
EVM0008736	43.29	13.31	1.70	5.00E-05	4.28E-04	Secondary metabolites biosynthesis, transport and catabolism	Alcohol dehydrogenase GroES-like domain

EVM0009319	129.20	39.72	1.70	5.00E-05	4.28E-04	Secondary metabolites biosynthesis, transport and catabolism	Cytochrome P450
EVM0004842	66.07	20.26	1.71	5.00E-05	4.28E-04	--	Ribosomal protein L11 methyltransferase (PrmA)
EVM0004652	8.97	2.75	1.71	5.00E-05	4.28E-04	--	Heterokaryon incompatibility protein (HET)
EVM0004847	6.27	1.92	1.71	5.00E-05	4.28E-04	--	--
EVM0005563	19.04	5.82	1.71	5.00E-05	4.28E-04	--	Protein of unknown function (DUF4246)
EVM0010821	1682.01	513.88	1.71	6.50E-04	3.98E-03	--	--
EVM0001273	350.28	106.99	1.71	5.00E-05	4.28E-04	Posttranslational modification, protein turnover, chaperones	Eukaryotic aspartyl protease
EVM0010182	19.82	6.05	1.71	5.00E-05	4.28E-04	--	Peptide N-acetyl-beta-D-glucosaminyl asparaginase amidase A
EVM0003410	100.14	30.58	1.71	5.00E-05	4.28E-04	--	--
EVM0009049	7.76	2.37	1.71	5.00E-04	3.19E-03	--	--
EVM0004076	124.16	37.80	1.72	5.00E-05	4.28E-04	--	--

EVM0010556	93.94	28.56	1.72	5.00E-05	4.28E-04	General function prediction only	Major Facilitator Superfamily
EVM0007712	28.42	8.63	1.72	5.00E-05	4.28E-04	General function prediction only	Protein kinase domain
EVM0000921	4.85	1.47	1.72	1.65E-03	8.65E-03	--	--
EVM0003917	3.80	1.15	1.73	3.50E-04	2.36E-03	Energy production and conversion	Aldehyde dehydrogenase family
EVM0009496	67.24	20.25	1.73	5.00E-05	4.28E-04	--	Sir2 family
EVM0003949	488.94	147.15	1.73	2.00E-04	1.46E-03	Energy production and conversion	Mitochondrial carrier protein
EVM0002121	19.84	5.97	1.73	5.00E-05	4.28E-04	Replication, recombination and repair	AAA domain
EVM0011363	2.22	0.67	1.73	2.00E-04	1.46E-03	RNA processing and modification	AAA domain
EVM0011287	35.08	10.54	1.73	5.00E-05	4.28E-04	Secondary metabolites biosynthesis, transport and catabolism	Cytochrome P450
EVM0002909	19.37	5.79	1.74	5.00E-05	4.28E-04	--	NAD(P)-binding Rossmann-like domain
EVM0011244	16.65	4.97	1.74	5.00E-05	4.28E-04	Carbohydrate transport and metabolism	Major Facilitator Superfamily
EVM0002891	64.35	19.23	1.74	5.00E-05	4.28E-04	--	--

EVM0003971	20.32	6.06	1.75	5.00E-05	4.28E-04	Coenzyme transport and metabolism	Dihydroneopterin aldolase
EVM0000698	78.52	23.39	1.75	5.00E-05	4.28E-04	--	Indoleamine 2,3-dioxygenase
EVM0003151	24.44	7.27	1.75	5.00E-05	4.28E-04	--	Phosphotyrosyl phosphate activator (PTPA) protein
EVM0009384	222.15	65.95	1.75	5.00E-05	4.28E-04	Amino acid transport and metabolism	Isocitrate/isopropylmalate dehydrogenase
EVM0008394	87.97	26.10	1.75	5.00E-05	4.28E-04	General function prediction only	Ankyrin repeats (3 copies)
EVM0000295	3685.04	1093.39	1.75	4.50E-04	2.92E-03	Inorganic ion transport and metabolism	Catalase
EVM0007775	112.31	33.28	1.75	5.00E-05	4.28E-04	--	--
EVM0010711	10.34	3.06	1.76	5.00E-05	4.28E-04	--	Fungal specific transcription factor domain
EVM0008484	91.54	27.11	1.76	5.00E-05	4.28E-04	--	Indoleamine 2,3-dioxygenase
EVM0002233	134.89	39.94	1.76	5.00E-05	4.28E-04	--	Dopa 4,5-dioxygenase family
EVM0003171	70.75	20.92	1.76	5.00E-05	4.28E-04	General function prediction only	Major Facilitator Superfamily
EVM0010379	65.57	19.38	1.76	5.00E-05	4.28E-04	Signal transduction mechanisms	Phosphatidylinositol-specific phospholipase C, X domain
EVM0011213	16.88	4.98	1.76	4.10E-03	1.83E-02	--	--

EVM0001110	61.21	18.06	1.76	5.00E-05	4.28E-04	--	CAP-Gly domain
EVM0009688	14.06	4.14	1.76	5.00E-05	4.28E-04	--	Zinc-binding dehydrogenase
EVM0007729	12.79	3.76	1.77	5.00E-05	4.28E-04	--	--
EVM0000320	87.54	25.73	1.77	5.00E-05	4.28E-04	--	--
EVM0004655	25.29	7.43	1.77	5.00E-05	4.28E-04	Amino acid transport and metabolism	Methylenetetrahydrofolate reductase
EVM0008304	174.99	51.41	1.77	5.00E-05	4.28E-04	Defense mechanisms	Dual specificity phosphatase, catalytic domain
EVM0007222	4.28	1.26	1.77	5.00E-05	4.28E-04	--	Protein of unknown function (DUF4246)
EVM0008717	144.29	42.29	1.77	5.00E-05	4.28E-04	--	Seed maturation protein
EVM0011790	15.12	4.43	1.77	1.95E-03	9.93E-03	General function prediction only	Ras family
EVM0007450	72.92	21.29	1.78	5.00E-05	4.28E-04	Amino acid transport and metabolism	Beta-eliminating lyase
EVM0008846	29.19	8.52	1.78	5.00E-05	4.28E-04	General function prediction only	Major Facilitator Superfamily
EVM0003774	30.62	8.90	1.78	5.00E-05	4.28E-04	Translation, ribosomal structure and biogenesis	tRNA synthetases class I (R)
EVM0011078	7.21	2.09	1.78	5.00E-05	4.28E-04	Carbohydrate transport and metabolism	Glycosyl hydrolases family 18

EVM0011462	34.50	9.99	1.79	5.00E-05	4.28E-04	Intracellular trafficking, secretion, and vesicular transport	Exocyst complex subunit Sec15-like
EVM0002582	326.95	94.50	1.79	5.00E-05	4.28E-04	Translation, ribosomal structure and biogenesis	RNB domain
EVM0006955	70.47	20.36	1.79	5.00E-05	4.28E-04	--	--
EVM0008310	20.15	5.81	1.79	5.00E-05	4.28E-04	--	--
EVM0004128	93.61	27.00	1.79	5.00E-05	4.28E-04	--	Peptidase family M1
EVM0010040	113.18	32.57	1.80	1.00E-04	7.96E-04	--	Polysaccharide lyase family 8, super-sandwich domain
EVM0006920	159.97	46.01	1.80	5.00E-05	4.28E-04	General function prediction only	Leucine Rich repeat
EVM0004630	4.64	1.33	1.80	8.00E-04	4.73E-03	--	--
EVM0006268	101.75	29.19	1.80	5.00E-05	4.28E-04	--	--
EVM0000675	28.95	8.28	1.81	5.00E-05	4.28E-04	--	Eukaryotic aspartyl protease
EVM0004012	1.22	0.35	1.81	6.30E-03	2.59E-02	--	FAD binding domain
EVM0011117	22.67	6.48	1.81	5.00E-05	4.28E-04	Cytoskeleton	Spc97 / Spc98 family

EVM0004494	98.28	28.02	1.81	5.00E-05	4.28E-04	Intracellular trafficking, secretion, and vesicular transport	SacI homology domain
EVM0004035	87.67	24.94	1.81	5.00E-05	4.28E-04	General function prediction only	TPR repeat
EVM0009262	370.00	105.06	1.82	5.00E-05	4.28E-04	General function prediction only	AN1-like Zinc finger
EVM0003363	124.09	35.23	1.82	1.00E-04	7.96E-04	--	--
EVM0006432	31.21	8.86	1.82	5.00E-05	4.28E-04	--	Fatty acid desaturase
EVM0008930	175.08	49.61	1.82	5.00E-05	4.28E-04	--	--
EVM0008780	2.74	0.78	1.82	1.13E-02	4.11E-02	--	Phosphotransferase enzyme family
EVM0000851	930.61	262.79	1.82	5.00E-05	4.28E-04	--	Eisosome component PIL1
EVM0008329	58.45	16.50	1.83	2.00E-04	1.46E-03	--	--
EVM0006290	68.26	19.19	1.83	5.00E-05	4.28E-04	General function prediction only	GMC oxidoreductase
EVM0009326	2.54	0.71	1.83	2.35E-03	1.16E-02	--	SMP-30/Gluconolaconase/LRE-like region
EVM0005785	15.96	4.48	1.83	5.00E-05	4.28E-04	--	Variant SH3 domain

EVM0002962	2322.03	651.05	1.83	2.00E-04	1.46E-03	--	--
EVM0002396	25.95	7.27	1.84	5.00E-05	4.28E-04	--	--
EVM0010629	76.46	21.37	1.84	5.00E-05	4.28E-04	--	Glycosyl hydrolase family 92
EVM0005615	5.33	1.49	1.84	5.00E-05	4.28E-04	Function unknown	Alpha/beta hydrolase family
EVM0008758	2.54	0.71	1.84	3.50E-04	2.36E-03	--	--
EVM0001061	2.44	0.68	1.84	5.00E-05	4.28E-04	Posttranslational modification, protein turnover, chaperones	BCS1 N terminal
EVM0011854	86.88	24.17	1.85	5.00E-05	4.28E-04	General function prediction only	Cyclic nucleotide-binding domain
EVM0000996	213.44	59.36	1.85	5.00E-05	4.28E-04	Lipid transport and metabolism	short chain dehydrogenase
EVM0000797	30.49	8.47	1.85	5.00E-05	4.28E-04	Translation, ribosomal structure and biogenesis	Piwi domain
EVM0006965	44.01	12.21	1.85	5.00E-05	4.28E-04	Inorganic ion transport and metabolism	Phosphate transporter family
EVM0003455	10.20	2.83	1.85	5.00E-05	4.28E-04	--	--
EVM0010770	199.17	55.11	1.85	5.00E-05	4.28E-04	--	--

EVM0010991	14.73	4.08	1.85	5.00E-05	4.28E-04	Carbohydrate transport and metabolism	Histidine phosphatase superfamily (branch 1)
EVM0010721	7.09	1.96	1.85	1.50E-03	8.00E-03	--	--
EVM0005144	32.01	8.84	1.86	5.00E-05	4.28E-04	--	--
EVM0003003	137.07	37.80	1.86	5.00E-05	4.28E-04	Coenzyme transport and metabolism	Glutamate-cysteine ligase
EVM0003622	2.76	0.76	1.86	5.50E-04	3.46E-03	--	Fungal Zn(2)-Cys(6) binuclear cluster domain
EVM0010725	4.72	1.30	1.86	2.50E-04	1.77E-03	--	--
EVM0000790	18.99	5.23	1.86	5.00E-05	4.28E-04	Translation, ribosomal structure and biogenesis	Piwi domain
EVM0006482	6.08	1.67	1.86	5.00E-05	4.28E-04	--	Oxidoreductase family, NAD-binding Rossmann fold
EVM0006788	217.88	59.89	1.86	5.00E-05	4.28E-04	--	--
EVM0005947	72.14	19.82	1.86	5.00E-05	4.28E-04	Signal transduction mechanisms	Carboxylesterase family
EVM0004530	69.86	19.19	1.86	5.00E-05	4.28E-04	--	Methyltransferase domain
EVM0001358	17.06	4.68	1.86	5.00E-05	4.28E-04	General function prediction only	GMC oxidoreductase
EVM0011158	410.29	112.46	1.87	5.00E-05	4.28E-04	--	--

EVM0007751	116.36	31.79	1.87	5.00E-05	4.28E-04	--	--
EVM0003172	373.90	102.16	1.87	5.00E-05	4.28E-04	Secondary metabolites biosynthesis, transport and catabolism	Animal haem peroxidase
EVM0005288	2.01	0.55	1.88	3.10E-03	1.45E-02	General function prediction only	GMC oxidoreductase
EVM0001047	238.30	64.77	1.88	5.00E-05	4.28E-04	--	BTB/POZ domain
EVM0007746	28.42	7.67	1.89	5.00E-05	4.28E-04	--	JmjC domain, hydroxylase
EVM0005970	188.44	50.84	1.89	5.00E-05	4.28E-04	Amino acid transport and metabolism	3-hydroxyanthranilic acid dioxygenase
EVM0000101	20.84	5.62	1.89	5.00E-05	4.28E-04	#N/A	#N/A
EVM0006857	12.34	3.33	1.89	5.00E-05	4.28E-04	Signal transduction mechanisms	Protein phosphatase 2C
EVM0010734	48.38	13.02	1.89	5.00E-05	4.28E-04	Energy production and conversion	Aldo/keto reductase family
EVM0010519	13.40	3.60	1.90	5.00E-05	4.28E-04	--	--
EVM0007749	41.53	11.13	1.90	5.00E-05	4.28E-04	General function prediction only	Major Facilitator Superfamily
EVM0004583	79.39	21.28	1.90	5.00E-05	4.28E-04	--	--

EVM0001702	15.19	4.07	1.90	5.00E-05	4.28E-04	--	--
EVM0008190	107.91	28.89	1.90	5.00E-05	4.28E-04	--	Uncharacterized conserved protein (DUF2156)
EVM0005665	131.64	35.23	1.90	5.00E-05	4.28E-04	General function prediction only	Zinc finger, C2H2 type
EVM0000520	67.32	17.99	1.90	5.00E-05	4.28E-04	General function prediction only	Josephin
EVM0010618	45.16	12.00	1.91	5.00E-05	4.28E-04	--	--
EVM0008733	96.79	25.69	1.91	5.00E-05	4.28E-04	Coenzyme transport and metabolism	ThiF family
EVM0001839	277.42	73.61	1.91	5.00E-05	4.28E-04	--	--
EVM0011628	86.64	22.99	1.91	5.00E-05	4.28E-04	Lipid transport and metabolism	PFU (PLAA family ubiquitin binding)
EVM0003352	17.00	4.50	1.92	5.00E-05	4.28E-04	General function prediction only	Major Facilitator Superfamily
EVM0008026	30.26	8.00	1.92	5.00E-05	4.28E-04	RNA processing and modification	AAA domain
EVM0008742	41.52	10.98	1.92	5.00E-05	4.28E-04	--	Reverse transcriptase (RNA-dependent DNA polymerase)
EVM0005759	30.73	8.12	1.92	5.00E-05	4.28E-04	--	Mycolic acid cyclopropane synthetase
EVM0008353	81.68	21.56	1.92	5.00E-05	4.28E-04	--	--

EVM0009922	183.42	48.38	1.92	5.00E-05	4.28E-04	--	Protein kinase domain
EVM0006559	79.54	20.97	1.92	5.00E-05	4.28E-04	--	--
EVM0000963	35.33	9.30	1.93	5.00E-05	4.28E-04	--	--
EVM0003132	208.76	54.75	1.93	5.00E-05	4.28E-04	--	Histidine-specific methyltransferase, SAM-dependent
EVM0005614	105.86	27.76	1.93	5.00E-05	4.28E-04	--	--
EVM0006913	38.72	10.15	1.93	5.00E-05	4.28E-04	--	--
EVM0005316	3.63	0.95	1.93	9.45E-03	3.58E-02	--	Dioxygenase
EVM0009288	287.11	75.14	1.93	5.00E-05	4.28E-04	--	--
EVM0009977	23.15	6.05	1.94	5.00E-05	4.28E-04	--	Ubiquitin 3 binding protein But2 C-terminal domain
EVM0003531	43.25	11.28	1.94	5.00E-05	4.28E-04	--	Fungal specific transcription factor domain
EVM0002374	58.22	15.18	1.94	5.00E-05	4.28E-04	--	Alpha/beta hydrolase family
EVM0005199	18.29	4.76	1.94	1.20E-03	6.64E-03	--	--

EVM0008199	5.18	1.35	1.94	5.00E-05	4.28E-04	Inorganic ion transport and metabolism	E1-E2 ATPase
EVM0010065	33.05	8.59	1.94	5.00E-05	4.28E-04	--	--
EVM0011687	33.85	8.79	1.95	5.00E-05	4.28E-04	Function unknown	Domain of unknown function (DUF3437)
EVM0006071	74.27	19.19	1.95	5.00E-05	4.28E-04	Translation, ribosomal structure and biogenesis	tRNA synthetases class II (D, K and N)
EVM0007085	24.60	6.34	1.96	5.00E-05	4.28E-04	Cytoskeleton	Regulator of Vps4 activity in the MVB pathway
EVM0001556	31.28	8.06	1.96	5.00E-05	4.28E-04	Amino acid transport and metabolism	Aminotransferase class I and II
EVM0008739	394.01	101.13	1.96	5.00E-05	4.28E-04	Energy production and conversion	Carbamoyl-phosphate synthase L chain, ATP binding domain
EVM0010940	3.23	0.83	1.96	3.00E-03	1.42E-02	--	--
EVM0008582	91.06	23.35	1.96	5.00E-05	4.28E-04	General function prediction only	short chain dehydrogenase
EVM0005538	30.31	7.76	1.96	5.00E-05	4.28E-04	--	--
EVM0005867	281.06	71.99	1.97	5.00E-05	4.28E-04	--	--
EVM0009164	213.21	54.60	1.97	4.60E-03	2.01E-02	Amino acid transport and metabolism	Aldehyde dehydrogenase family

EVM0001672	22.45	5.74	1.97	5.00E-05	4.28E-04	Secondary metabolites biosynthesis, transport and catabolism	Alcohol dehydrogenase GroES-like domain
EVM0004021	42.66	10.90	1.97	5.00E-05	4.28E-04	--	Cytochrome P450
EVM0004441	54.76	13.95	1.97	5.00E-05	4.28E-04	Lipid transport and metabolism	Squalene/phytoene synthase
EVM0002115	25.02	6.37	1.97	5.00E-05	4.28E-04	--	Pregnancy-associated plasma protein-A
EVM0007759	91.39	23.26	1.97	5.00E-05	4.28E-04	--	F-box-like
EVM0005969	143.32	36.42	1.98	5.00E-05	4.28E-04	Function unknown	Major Facilitator Superfamily
EVM0006888	53.66	13.62	1.98	5.00E-05	4.28E-04	--	Methyltransferase domain
EVM0003122	53.18	13.46	1.98	5.00E-05	4.28E-04	Translation, ribosomal structure and biogenesis	Domain of unknown function (DUF3554)
EVM0002275	9.41	2.38	1.98	5.00E-05	4.28E-04	--	--
EVM0011935	245.69	61.91	1.99	5.00E-05	4.28E-04	Lipid transport and metabolism	Beta-ketoacyl synthase, N-terminal domain
EVM0009444	12118.40	3051.67	1.99	3.50E-03	1.61E-02	--	Fungal hydrophobin
EVM0008881	317.37	79.47	2.00	5.00E-05	4.28E-04	--	Glycosyl hydrolase family 3 N terminal domain

EVM0005243	14.65	3.67	2.00	5.00E-04	3.19E-03	--	--
EVM0004316	84.39	21.10	2.00	5.00E-05	4.28E-04	--	--
EVM0010231	92.02	23.00	2.00	5.00E-05	4.28E-04	--	NPL4 family
EVM0008827	6.39	1.59	2.01	1.30E-03	7.10E-03	--	--
EVM0003331	20.11	5.00	2.01	5.00E-05	4.28E-04	--	Trehalose utilisation
EVM0006787	1.94	0.48	2.01	1.23E-02	4.42E-02	--	Membrane bound O-acyl transferase family
EVM0008131	455.01	112.93	2.01	5.00E-05	4.28E-04	--	Cellulase (glycosyl hydrolase family 5)
EVM0003669	16.84	4.18	2.01	5.00E-05	4.28E-04	--	--
EVM0004619	33.45	8.28	2.01	5.00E-05	4.28E-04	Nucleotide transport and metabolism	Amidohydrolase
EVM0011824	10.89	2.69	2.02	5.00E-05	4.28E-04	Carbohydrate transport and metabolism	Glycosyl hydrolase family 1
EVM0007351	55.42	13.69	2.02	5.00E-05	4.28E-04	--	--
EVM0006868	762.13	188.25	2.02	5.00E-05	4.28E-04	General function prediction only	RNA recognition motif. (a.k.a. RRM, RBD, or RNP domain)
EVM0003765	881.70	217.76	2.02	5.00E-05	4.28E-04	Amino acid transport and metabolism	Amino acid permease

EVM0004492	8.89	2.19	2.02	5.00E-05	4.28E-04	--	Ricin-type beta-trefoil lectin domain
EVM0009770	75.27	18.53	2.02	5.00E-05	4.28E-04	Secondary metabolites biosynthesis, transport and catabolism	short chain dehydrogenase
EVM0004547	24.94	6.13	2.02	5.00E-05	4.28E-04	--	--
EVM0006858	372.66	91.66	2.02	5.00E-05	4.28E-04	Energy production and conversion	Aldehyde dehydrogenase family
EVM0007374	62.94	15.48	2.02	5.00E-05	4.28E-04	--	--
EVM0009595	57.87	14.20	2.03	5.00E-05	4.28E-04	--	--
EVM0005310	2602.87	638.49	2.03	5.00E-05	4.28E-04	General function prediction only	Enoyl-(Acyl carrier protein) reductase
EVM0011184	162.54	39.80	2.03	5.00E-05	4.28E-04	--	NADH:flavin oxidoreductase / NADH oxidase family
EVM0006615	6.89	1.68	2.04	5.00E-05	4.28E-04	--	S1/P1 Nuclease
EVM0011389	177.93	43.33	2.04	5.00E-05	4.28E-04	--	FAD binding domain
EVM0009298	56.13	13.66	2.04	5.00E-05	4.28E-04	--	--
EVM0011865	412.44	100.20	2.04	5.00E-05	4.28E-04	Energy production and conversion	ATP synthase alpha/beta family, nucleotide-binding domain

EVM0009917	3.07	0.75	2.04	5.00E-05	4.28E-04	--	--
EVM0002907	130.17	31.58	2.04	5.00E-05	4.28E-04	Defense mechanisms	NAD dependent epimerase/dehydratase family
EVM0008764	103.90	25.18	2.05	5.00E-05	4.28E-04	Amino acid transport and metabolism	Asparagine synthase
EVM0012016	688.67	166.76	2.05	5.00E-05	4.28E-04	Amino acid transport and metabolism	Cys/Met metabolism PLP-dependent enzyme
EVM0011421	64.06	15.51	2.05	5.00E-05	4.28E-04	#N/A	#N/A
EVM0002652	4.95	1.20	2.05	5.60E-03	2.36E-02	--	--
EVM0007101	91.69	22.14	2.05	5.00E-05	4.28E-04	--	50S ribosome-binding GTPase
EVM0000119	27.07	6.52	2.05	5.00E-05	4.28E-04	--	3-beta hydroxysteroid dehydrogenase/isomerase family
EVM0010091	38.51	9.27	2.05	5.00E-05	4.28E-04	--	--
EVM0009683	72.92	17.55	2.06	5.00E-05	4.28E-04	--	Cytochrome P450
EVM0005295	33.17	7.97	2.06	1.00E-04	7.96E-04	--	--
EVM0008439	48.09	11.52	2.06	5.00E-05	4.28E-04	--	Uncharacterized alpha/beta hydrolase domain (DUF2235)
EVM0000347	292.07	69.67	2.07	5.00E-05	4.28E-04	--	--

EVM0011460	458.83	109.41	2.07	1.00E-04	7.96E-04	--	--
EVM0004481	35.06	8.35	2.07	5.00E-05	4.28E-04	--	--
EVM0009044	102.53	24.39	2.07	5.00E-05	4.28E-04	--	--
EVM0006505	117.78	27.98	2.07	5.00E-05	4.28E-04	Signal transduction mechanisms	OPT oligopeptide transporter protein
EVM0004153	274.66	65.22	2.07	5.00E-05	4.28E-04	--	--
EVM0004544	19.58	4.65	2.07	5.00E-05	4.28E-04	--	Ricin-type beta-trefoil lectin domain-like
EVM0008715	44.99	10.63	2.08	5.00E-05	4.28E-04	--	Zinc-binding dehydrogenase
EVM0001999	42.16	9.93	2.09	5.00E-05	4.28E-04	--	Alpha/beta hydrolase family
EVM0001569	30.77	7.22	2.09	5.00E-05	4.28E-04	General function prediction only	Major Facilitator Superfamily
EVM0012021	7.64	1.79	2.10	6.70E-03	2.72E-02	--	--
EVM0011011	13.85	3.23	2.10	1.50E-04	1.14E-03	--	--
EVM0000333	29.16	6.81	2.10	5.00E-05	4.28E-04	Posttranslational modification, protein turnover, chaperones	Poly(ADP-ribose) polymerase catalytic domain

EVM0002778	14.98	3.49	2.10	5.00E-05	4.28E-04	Chromatin structure and dynamics	Paired amphipathic helix repeat
EVM0003460	15.76	3.67	2.10	1.50E-04	1.14E-03	--	--
EVM0002221	45.03	10.48	2.10	5.00E-05	4.28E-04	Amino acid transport and metabolism	Cys/Met metabolism PLP-dependent enzyme
EVM0010242	6.89	1.60	2.10	5.00E-05	4.28E-04	Function unknown	WD domain, G-beta repeat
EVM0005441	70.03	16.27	2.11	5.00E-05	4.28E-04	General function prediction only	Major Facilitator Superfamily
EVM0001497	39.21	9.10	2.11	5.00E-05	4.28E-04	--	NADH(P)-binding
EVM0002888	791.05	183.33	2.11	5.00E-05	4.28E-04	--	--
EVM0000082	11.75	2.69	2.12	2.00E-04	1.46E-03	--	WSC domain
EVM0000559	3.33	0.76	2.12	8.25E-03	3.21E-02	--	Glycosyl hydrolase family 61
EVM0000944	1823.73	417.90	2.13	5.00E-05	4.28E-04	General function prediction only	Aldo/keto reductase family
EVM0006901	587.01	134.32	2.13	5.00E-05	4.28E-04	--	Acetohydroxy acid isomeroreductase, catalytic domain
EVM0007212	25.83	5.90	2.13	5.00E-05	4.28E-04	--	Cytochrome P450

EVM0001331	48.73	11.13	2.13	5.00E-05	4.28E-04	--	Ankyrin repeats (many copies)
EVM0008410	591.24	134.90	2.13	5.00E-05	4.28E-04	Cytoskeleton	Autophagy protein Atg8 ubiquitin like
EVM0003064	8.93	2.03	2.14	4.10E-03	1.83E-02	--	--
EVM0005776	353.10	79.92	2.14	5.00E-05	4.28E-04	Posttranslational modification, protein turnover, chaperones	Glutathione S-transferase, N-terminal domain
EVM0007214	17.17	3.88	2.14	3.50E-04	2.36E-03	--	--
EVM0001285	180.20	40.73	2.15	5.00E-05	4.28E-04	--	Alternative oxidase
EVM0007446	1647.58	371.57	2.15	5.00E-05	4.28E-04	--	Fungalysin metallopeptidase (M36)
EVM0008349	218.66	49.28	2.15	5.00E-05	4.28E-04	Secondary metabolites biosynthesis, transport and catabolism	Cytochrome P450
EVM0011116	10.46	2.36	2.15	5.00E-05	4.28E-04	--	Acetamidase/Formamidase family
EVM0010159	469.44	105.61	2.15	5.00E-05	4.28E-04	Amino acid transport and metabolism	Aconitase family (aconitate hydratase)
EVM0001296	35.24	7.92	2.15	5.00E-05	4.28E-04	--	FR47-like protein
EVM0009509	164.33	36.72	2.16	5.00E-05	4.28E-04	General function prediction only	GMC oxidoreductase

EVM0005650	98.84	22.03	2.17	5.00E-05	4.28E-04	--	--
EVM0008479	29.16	6.49	2.17	5.00E-05	4.28E-04	--	Whi5 like
EVM0009222	20.99	4.67	2.17	5.00E-05	4.28E-04	--	Uncharacterized protein conserved in bacteria (DUF2264)
EVM0004179	14.19	3.15	2.17	5.00E-05	4.28E-04	Lipid transport and metabolism	short chain dehydrogenase
EVM0004887	7.47	1.66	2.17	5.00E-05	4.28E-04	--	Protein of unknown function (DUF4246)
EVM0010798	994.61	220.18	2.18	5.00E-05	4.28E-04	--	Stress responsive A/B Barrel Domain
EVM0011073	57.72	12.76	2.18	5.00E-05	4.28E-04	Amino acid transport and metabolism	Aminotransferase class-III
EVM0011946	4.14	0.91	2.18	5.15E-03	2.20E-02	--	SUR7/PalI family
EVM0002807	157.05	34.65	2.18	5.00E-05	4.28E-04	--	--
EVM0003320	28.79	6.34	2.18	5.00E-05	4.28E-04	--	--
EVM0000781	91.72	20.21	2.18	5.00E-05	4.28E-04	Amino acid transport and metabolism	FAD dependent oxidoreductase
EVM0000028	29.19	6.43	2.18	5.00E-05	4.28E-04	Nucleotide transport and metabolism	AAA domain

EVM0003697	7.70	1.69	2.18	5.50E-04	3.46E-03	--	Polyketide cyclase / dehydrase and lipid transport
EVM0005515	17.78	3.90	2.19	5.00E-05	4.28E-04	Secondary metabolites biosynthesis, transport and catabolism	Cytochrome P450
EVM0003226	345.15	75.37	2.20	5.00E-05	4.28E-04	--	Protein of unknown function (DUF3602)
EVM0010499	1.90	0.41	2.21	1.34E-02	4.73E-02	--	Protein of unknown function (DUF4246)
EVM0001166	145.86	31.44	2.21	5.00E-05	4.28E-04	Secondary metabolites biosynthesis, transport and catabolism	Cytochrome P450
EVM0012096	3684.23	793.58	2.21	5.00E-05	4.28E-04	Carbohydrate transport and metabolism	Glyceraldehyde 3-phosphate dehydrogenase, C-terminal domain
EVM0000411	4.95	1.06	2.22	5.00E-05	4.28E-04	Signal transduction mechanisms	Protein tyrosine kinase
EVM0003849	168.92	36.25	2.22	5.00E-05	4.28E-04	--	--
EVM0006542	2175.70	466.37	2.22	5.00E-05	4.28E-04	--	Ricin-type beta-trefoil lectin domain
EVM0007990	21.09	4.52	2.22	5.00E-05	4.28E-04	--	Carbonic anhydrase
EVM0003801	52.49	11.23	2.23	5.00E-05	4.28E-04	--	--

EVM0005160	1044.83	223.14	2.23	5.00E-05	4.28E-04	Lipid transport and metabolism	Phosphatidylserine decarboxylase
EVM0001737	43.05	9.19	2.23	5.00E-05	4.28E-04	Secondary metabolites biosynthesis, transport and catabolism	Cytochrome P450
EVM0011640	24.87	5.31	2.23	5.00E-05	4.28E-04	--	--
EVM0011233	137.22	29.21	2.23	5.00E-05	4.28E-04	--	--
EVM0009853	229.00	48.73	2.23	5.00E-05	4.28E-04	--	--
EVM0001947	4.69	1.00	2.23	3.10E-03	1.45E-02	--	--
EVM0000117	158.56	33.67	2.24	5.00E-05	4.28E-04	--	Peroxidase, family 2
EVM0006769	67.66	14.30	2.24	5.00E-05	4.28E-04	Translation, ribosomal structure and biogenesis	tRNA methyltransferase complex GCD14 subunit
EVM0008687	55.66	11.74	2.25	5.00E-05	4.28E-04	Signal transduction mechanisms	Low molecular weight phosphotyrosine protein phosphatase
EVM0002398	17.31	3.63	2.25	5.00E-05	4.28E-04	--	--
EVM0007713	17.05	3.57	2.25	1.30E-03	7.10E-03	--	PLAC8 family
EVM0005281	10.26	2.14	2.26	5.00E-05	4.28E-04	Amino acid transport and metabolism	Gamma-glutamyltranspeptidase

EVM0003118	48.35	10.08	2.26	5.00E-05	4.28E-04	--	--
EVM0006645	360.08	74.72	2.27	5.00E-05	4.28E-04	--	--
EVM0005718	92.98	19.27	2.27	5.00E-05	4.28E-04	General function prediction only	WD domain, G-beta repeat
EVM0004219	348.33	71.97	2.28	5.00E-05	4.28E-04	--	NADH:flavin oxidoreductase / NADH oxidase family
EVM0005770	47.75	9.86	2.28	5.00E-05	4.28E-04	--	--
EVM0008708	40.70	8.34	2.29	5.00E-05	4.28E-04	--	--
EVM0007588	11.51	2.34	2.30	5.00E-05	4.28E-04	--	--
EVM0002941	32.98	6.70	2.30	5.00E-05	4.28E-04	Nucleotide transport and metabolism	Ham1 family
EVM0007684	183.12	36.89	2.31	5.00E-05	4.28E-04	General function prediction only	Major Facilitator Superfamily
EVM0007260	71.34	14.33	2.32	5.00E-05	4.28E-04	--	Domain of unknown function (DUF3328)
EVM0007480	6.09	1.22	2.32	5.00E-05	4.28E-04	--	Protein of unknown function (DUF3712)
EVM0012240	3833.40	767.07	2.32	5.00E-05	4.28E-04	--	--
EVM0004243	2.92	0.58	2.32	1.40E-03	7.55E-03	Signal transduction mechanisms	Protein tyrosine kinase

EVM0012129	40.16	8.03	2.32	5.00E-05	4.28E-04	--	--
EVM0000318	3.10	0.62	2.33	4.80E-03	2.08E-02	--	--
EVM0010628	22.86	4.56	2.33	5.00E-05	4.28E-04	--	Taurine catabolism dioxygenase TauD, TfdA family
EVM0001060	68.36	13.62	2.33	5.00E-05	4.28E-04	--	--
EVM0005693	562.69	112.15	2.33	5.00E-05	4.28E-04	--	--
EVM0005948	4337.93	862.63	2.33	5.00E-05	4.28E-04	--	--
EVM0010637	10.79	2.14	2.34	5.00E-05	4.28E-04	--	Enolase C-terminal domain-like
EVM0008011	979.97	193.57	2.34	1.00E-04	7.96E-04	--	--
EVM0006786	460.08	90.57	2.34	5.00E-05	4.28E-04	Defense mechanisms	alpha/beta hydrolase fold
EVM0004529	1.34	0.26	2.35	7.95E-03	3.12E-02	--	--
EVM0004712	99.22	19.50	2.35	5.00E-05	4.28E-04	Secondary metabolites biosynthesis, transport and catabolism	short chain dehydrogenase
EVM0000105	89.99	17.69	2.35	5.00E-05	4.28E-04	--	--

EVM0004764	28.01	5.49	2.35	5.00E-05	4.28E-04	--	--
EVM0012137	93.51	18.32	2.35	5.00E-05	4.28E-04	--	--
EVM0007692	128.46	25.07	2.36	5.00E-05	4.28E-04	--	--
EVM0005324	26.37	5.13	2.36	5.00E-05	4.28E-04	--	--
EVM0000973	15.49	3.01	2.36	5.00E-05	4.28E-04	Energy production and conversion	FMN-dependent dehydrogenase
EVM0004672	139.39	27.08	2.36	5.00E-05	4.28E-04	--	--
EVM0004917	34.59	6.70	2.37	7.70E-03	3.04E-02	--	--
EVM0007307	151.07	29.17	2.37	5.00E-05	4.28E-04	--	--
EVM0002528	100.20	19.34	2.37	5.00E-05	4.28E-04	Cytoskeleton	WD domain, G-beta repeat
EVM0002737	27.64	5.33	2.38	5.00E-05	4.28E-04	--	--
EVM0011531	102.31	19.67	2.38	5.00E-05	4.28E-04	--	--
EVM0002562	867.94	166.65	2.38	5.00E-05	4.28E-04	Posttranslational modification, protein turnover, chaperones	AhpC/TSA family

EVM0003243	36.65	7.03	2.38	5.00E-05	4.28E-04	Secondary metabolites biosynthesis, transport and catabolism	AMP-binding enzyme
EVM0008150	80.30	15.36	2.39	5.00E-05	4.28E-04	Energy production and conversion	Aldo/keto reductase family
EVM0011825	5.33	1.02	2.39	5.00E-05	4.28E-04	Carbohydrate transport and metabolism	Glycosyl hydrolases family 35
EVM0005389	1978.18	376.35	2.39	5.00E-05	4.28E-04	--	Cerato-platanin
EVM0005326	9.75	1.85	2.39	5.00E-05	4.28E-04	General function prediction only	GMC oxidoreductase
EVM0001114	500.98	95.22	2.40	1.00E-04	7.96E-04	General function prediction only	GMC oxidoreductase
EVM0010790	1068.99	202.77	2.40	5.00E-05	4.28E-04	--	Cellulase (glycosyl hydrolase family 5)
EVM0004054	2923.38	554.24	2.40	4.50E-04	2.92E-03	Posttranslational modification, protein turnover, chaperones	Hsp20/alpha crystallin family
EVM0000933	5.96	1.13	2.40	5.00E-05	4.28E-04	Energy production and conversion	Malic enzyme, NAD binding domain
EVM0012050	214.40	40.47	2.41	5.00E-05	4.28E-04	General function prediction only	Zinc finger, C2H2 type
EVM0002202	31.72	5.94	2.42	5.00E-05	4.28E-04	--	Putative glycosyl hydrolase of unknown function (DUF1680)
EVM0005095	61.60	11.51	2.42	5.00E-05	4.28E-04	--	--

EVM0009612	45.77	8.55	2.42	5.00E-05	4.28E-04	--	--
EVM0004717	12.53	2.34	2.42	5.00E-05	4.28E-04	--	Protein tyrosine kinase
EVM0008826	918.99	171.42	2.42	5.00E-05	4.28E-04	--	--
EVM0002235	27.45	5.12	2.42	5.00E-05	4.28E-04	--	Pectinesterase
EVM0007950	237.85	44.18	2.43	5.00E-05	4.28E-04	Carbohydrate transport and metabolism	Carbohydrate phosphorylase
EVM0008109	518.90	96.17	2.43	5.00E-05	4.28E-04	Carbohydrate transport and metabolism	Fructose-1-6-bisphosphatase
EVM0002796	87.16	16.13	2.43	5.00E-05	4.28E-04	--	Permease for cytosine/purines, uracil, thiamine, allantoin
EVM0008828	77.66	14.31	2.44	5.00E-05	4.28E-04	--	--
EVM0007764	19.91	3.66	2.44	5.00E-05	4.28E-04	--	SNF2 family N-terminal domain
EVM0005372	21.08	3.86	2.45	5.00E-05	4.28E-04	--	Alpha/beta hydrolase family
EVM0003021	101.78	18.61	2.45	5.00E-05	4.28E-04	--	AIG1 family
EVM0000156	2.40	0.44	2.45	8.15E-03	3.18E-02	--	--
EVM0004741	365.17	66.63	2.45	5.00E-05	4.28E-04	--	Fungal Zn(2)-Cys(6) binuclear cluster domain

EVM0000184	59.07	10.70	2.46	5.00E-05	4.28E-04	Signal transduction mechanisms	OPT oligopeptide transporter protein
EVM0003997	66.75	12.08	2.47	5.00E-05	4.28E-04	General function prediction only	Haloacid dehalogenase-like hydrolase
EVM0009135	12.27	2.21	2.47	5.00E-05	4.28E-04	--	--
EVM0007942	399.70	71.75	2.48	5.00E-05	4.28E-04	Function unknown	Protein of unknown function DUF89
EVM0010831	1593.33	285.08	2.48	5.00E-05	4.28E-04	--	Phosphoenolpyruvate carboxykinase
EVM0002269	91.48	16.30	2.49	5.00E-05	4.28E-04	General function prediction only	Sugar (and other) transporter
EVM0010699	7420.86	1322.16	2.49	5.00E-05	4.28E-04	Energy production and conversion	Aldo/keto reductase family
EVM0011225	42.77	7.61	2.49	5.00E-05	4.28E-04	RNA processing and modification	Ribonuclease III domain
EVM0004082	3.29	0.58	2.49	1.10E-03	6.17E-03	--	NADH:flavin oxidoreductase / NADH oxidase family
EVM0009634	22.24	3.95	2.49	2.45E-03	1.20E-02	--	--
EVM0005170	15.33	2.72	2.50	5.00E-05	4.28E-04	Secondary metabolites biosynthesis, transport and catabolism	Cytochrome P450
EVM0006519	163.55	28.88	2.50	5.00E-05	4.28E-04	--	Putative amidotransferase

EVM0003927	114.64	20.18	2.51	9.25E-03	3.52E-02	--	Complex 1 protein (LYR family)
EVM0008616	57.78	10.17	2.51	5.00E-05	4.28E-04	Carbohydrate transport and metabolism	Major Facilitator Superfamily
EVM0003920	129.63	22.78	2.51	5.00E-05	4.28E-04	Lipid transport and metabolism	AMP-binding enzyme
EVM0009588	140.93	24.74	2.51	5.00E-05	4.28E-04	Secondary metabolites biosynthesis, transport and catabolism	Cytochrome P450
EVM0011095	433.43	76.08	2.51	5.00E-05	4.28E-04	Secondary metabolites biosynthesis, transport and catabolism	AMP-binding enzyme
EVM0009967	272.20	47.66	2.51	5.00E-05	4.28E-04	--	Dynamin family
EVM0009915	73.29	12.75	2.52	5.00E-05	4.28E-04	Secondary metabolites biosynthesis, transport and catabolism	Cytochrome P450
EVM0007275	506.14	87.96	2.52	5.00E-05	4.28E-04	Amino acid transport and metabolism	Aminotransferase class I and II
EVM0005755	121.21	21.02	2.53	5.00E-05	4.28E-04	--	Glycosyl hydrolases family 16
EVM0005547	479.80	83.13	2.53	5.00E-05	4.28E-04	Secondary metabolites biosynthesis, transport and catabolism	Aromatic amino acid lyase
EVM0000317	52.84	9.14	2.53	5.00E-05	4.28E-04	--	--

EVM0010773	6.36	1.10	2.53	5.00E-05	4.28E-04	General function prediction only	Major Facilitator Superfamily
EVM0002104	130.05	22.48	2.53	5.00E-05	4.28E-04	Signal transduction mechanisms	Protein kinase domain
EVM0000703	165.05	28.51	2.53	5.00E-05	4.28E-04	--	--
EVM0011732	21.58	3.72	2.54	5.00E-05	4.28E-04	Amino acid transport and metabolism	homogentisate 1,2-dioxygenase
EVM0006399	54.41	9.31	2.55	5.00E-05	4.28E-04	General function prediction only	short chain dehydrogenase
EVM0010985	194.44	33.19	2.55	5.00E-05	4.28E-04	Carbohydrate transport and metabolism	Transaldolase
EVM0003077	461.71	78.71	2.55	1.00E-04	7.96E-04	--	50S ribosome-binding GTPase
EVM0006190	303.73	51.62	2.56	5.00E-05	4.28E-04	Amino acid transport and metabolism	Amino acid permease
EVM0011562	227.88	38.71	2.56	5.00E-05	4.28E-04	--	--
EVM0011387	110.47	18.67	2.56	5.00E-05	4.28E-04	Translation, ribosomal structure and biogenesis	tRNA synthetases class I (E and Q), catalytic domain
EVM0001750	29.30	4.95	2.57	5.00E-05	4.28E-04	Amino acid transport and metabolism	Amino acid permease
EVM0001905	264.19	44.53	2.57	5.00E-05	4.28E-04	General function prediction only	GMC oxidoreductase

EVM0011252	28.25	4.76	2.57	5.00E-05	4.28E-04	Secondary metabolites biosynthesis, transport and catabolism	ABC-2 type transporter
EVM0002738	44.75	7.51	2.58	5.00E-05	4.28E-04	--	--
EVM0005194	4.52	0.75	2.59	1.00E-04	7.96E-04	--	Glycosyl hydrolases family 28
EVM0009885	49.61	8.21	2.59	5.00E-05	4.28E-04	--	--
EVM0011299	298.60	49.39	2.60	5.00E-05	4.28E-04	General function prediction only	Sugar (and other) transporter
EVM0007276	9.58	1.58	2.60	5.00E-05	4.28E-04	--	Acetyltransferase (GNAT) domain
EVM0008760	12.80	2.11	2.60	5.00E-05	4.28E-04	--	--
EVM0002399	219.76	36.15	2.60	5.00E-05	4.28E-04	--	NADH(P)-binding
EVM0000412	2401.74	395.08	2.60	2.50E-04	1.77E-03	--	Thiamine pyrophosphate enzyme, N-terminal TPP binding domain
EVM0005155	14.17	2.32	2.61	5.00E-05	4.28E-04	--	--
EVM0002050	246.63	40.07	2.62	3.20E-03	1.49E-02	--	--
EVM0007831	18.99	3.08	2.63	5.00E-05	4.28E-04	Lipid transport and metabolism	Lipase (class 3)

EVM0008919	3.92	0.63	2.63	1.00E-04	7.96E-04	--	--
EVM0007580	506.80	81.76	2.63	5.00E-05	4.28E-04	Cell wall/membrane/envelope biogenesis	Glycosyl transferases group 1
EVM0002732	128.68	20.56	2.65	5.00E-05	4.28E-04	Function unknown	Cysteine-rich secretory protein family
EVM0005680	56.01	8.93	2.65	5.00E-05	4.28E-04	Carbohydrate transport and metabolism	Major Facilitator Superfamily
EVM0004089	112.78	17.97	2.65	5.00E-05	4.28E-04	--	MAPEG family
EVM0008238	52.78	8.35	2.66	5.00E-05	4.28E-04	--	Cytochrome P450
EVM0004177	313.65	49.55	2.66	5.00E-05	4.28E-04	Inorganic ion transport and metabolism	Nitrite and sulphite reductase 4Fe-4S domain
EVM0008569	21.48	3.39	2.66	8.50E-04	4.98E-03	--	--
EVM0009544	87.78	13.78	2.67	5.00E-05	4.28E-04	Posttranslational modification, protein turnover, chaperones	IBR domain
EVM0008705	7.18	1.12	2.68	5.00E-05	4.28E-04	General function prediction only	Zinc-binding dehydrogenase
EVM0001379	38.80	6.07	2.68	5.00E-05	4.28E-04	--	FHA domain
EVM0000202	271.29	42.39	2.68	5.00E-05	4.28E-04	--	Glycosyl transferase family group 2

EVM0007642	1836.29	286.69	2.68	5.00E-05	4.28E-04	--	Pregnancy-associated plasma protein-A
EVM0008970	96.88	15.10	2.68	5.00E-05	4.28E-04	--	Cytochrome P450
EVM0005169	4332.32	674.83	2.68	5.00E-05	4.28E-04	--	Peptidase family M28
EVM0007492	381.81	59.46	2.68	5.00E-05	4.28E-04	Amino acid transport and metabolism	Cobalamin-independent synthase, Catalytic domain
EVM0011104	82.89	12.90	2.68	5.00E-05	4.28E-04	Inorganic ion transport and metabolism	Sodium/hydrogen exchanger family
EVM0001169	3.48	0.54	2.69	1.25E-03	6.87E-03	--	--
EVM0008397	2285.68	354.04	2.69	5.00E-05	4.28E-04	--	--
EVM0011435	70.56	10.88	2.70	5.00E-05	4.28E-04	Cytoskeleton	Myosin head (motor domain)
EVM0010184	139.00	21.38	2.70	5.00E-05	4.28E-04	Cell wall/membrane/envelope biogenesis	Chitin synthase
EVM0000639	110.80	17.00	2.70	5.00E-05	4.28E-04	--	--
EVM0000466	116.74	17.87	2.71	5.00E-05	4.28E-04	General function prediction only	Peptidase family M49
EVM0000263	99.65	15.22	2.71	2.00E-04	1.46E-03	--	--

EVM0002563	30.58	4.67	2.71	5.00E-05	4.28E-04	--	Protein of unknown function (DUF1399)
EVM0004067	176.03	26.58	2.73	5.00E-05	4.28E-04	General function prediction only	HMG (high mobility group) box
EVM0012169	159.01	23.73	2.74	5.00E-05	4.28E-04	--	CVNH domain
EVM0009717	616.32	91.64	2.75	5.00E-05	4.28E-04	--	--
EVM0009749	166.45	24.67	2.75	5.00E-05	4.28E-04	--	--
EVM0005835	4.79	0.71	2.76	5.00E-05	4.28E-04	--	Protein tyrosine kinase
EVM0005899	174.24	25.56	2.77	5.00E-05	4.28E-04	--	--
EVM0004350	3905.39	571.31	2.77	5.00E-05	4.28E-04	Energy production and conversion	Aldo/keto reductase family
EVM0008192	298.23	43.62	2.77	5.00E-05	4.28E-04	Posttranslational modification, protein turnover, chaperones	ThiF family
EVM0005425	14.39	2.10	2.78	5.00E-05	4.28E-04	--	--
EVM0010863	82.42	12.01	2.78	5.00E-05	4.28E-04	General function prediction only	O-methyltransferase
EVM0012178	77.64	11.27	2.78	5.00E-05	4.28E-04	Defense mechanisms	NADH(P)-binding

EVM0006785	35.82	5.20	2.78	1.00E-04	7.96E-04	--	Frag1/DRAM/Sfk1 family
EVM0006962	180.75	26.20	2.79	5.00E-05	4.28E-04	Intracellular trafficking, secretion, and vesicular transport	ABC transporter
EVM0005231	124.54	18.01	2.79	5.00E-05	4.28E-04	Amino acid transport and metabolism	FAD dependent oxidoreductase
EVM0011129	153.21	22.14	2.79	5.00E-05	4.28E-04	--	--
EVM0008324	26.80	3.84	2.80	5.00E-05	4.28E-04	Secondary metabolites biosynthesis, transport and catabolism	Cytochrome P450
EVM0003709	37.73	5.39	2.81	5.00E-05	4.28E-04	Energy production and conversion	Galactose-1-phosphate uridyl transferase, N-terminal domain
EVM0005742	27.40	3.89	2.82	5.00E-05	4.28E-04	General function prediction only	short chain dehydrogenase
EVM0008462	7.07	1.00	2.82	5.00E-05	4.28E-04	--	NADH:flavin oxidoreductase / NADH oxidase family
EVM0011417	172.46	24.40	2.82	5.00E-05	4.28E-04	Energy production and conversion	Aldo/keto reductase family
EVM0004693	187.23	26.46	2.82	5.00E-05	4.28E-04	--	--
EVM0007867	198.98	28.09	2.82	5.00E-05	4.28E-04	--	--
EVM0011267	614.21	85.80	2.84	5.00E-05	4.28E-04	Energy production and conversion	Aldo/keto reductase family

EVM0004385	56.95	7.95	2.84	5.00E-05	4.28E-04	--	--
EVM0005375	26.27	3.67	2.84	5.00E-05	4.28E-04	--	--
EVM0007578	2.87	0.40	2.85	3.50E-04	2.36E-03	General function prediction only	GMC oxidoreductase
EVM0003237	209.49	29.09	2.85	5.00E-05	4.28E-04	Amino acid transport and metabolism	Glutamate/Leucine/Phenylalanine/Valine dehydrogenase
EVM0005998	91.63	12.63	2.86	5.00E-05	4.28E-04	Inorganic ion transport and metabolism	Adenylylsulphate kinase
EVM0001985	56.78	7.82	2.86	5.00E-05	4.28E-04	--	von Willebrand factor type A domain
EVM0011477	33.76	4.64	2.86	5.00E-05	4.28E-04	Posttranslational modification, protein turnover, chaperones	Glutathione S-transferase, N-terminal domain
EVM0000351	1206.68	165.48	2.87	5.00E-05	4.28E-04	--	Dyp-type peroxidase family
EVM0004300	125.57	17.17	2.87	5.00E-05	4.28E-04	Secondary metabolites biosynthesis, transport and catabolism	Cytochrome P450
EVM0011451	15.87	2.17	2.87	5.00E-05	4.28E-04	--	SH3-binding, glutamic acid-rich protein
EVM0006055	24.65	3.34	2.89	5.00E-05	4.28E-04	General function prediction only	short chain dehydrogenase
EVM0009468	1.81	0.24	2.89	5.00E-05	4.28E-04	RNA processing and modification	AAA domain

EVM0005679	70.13	9.42	2.90	5.00E-05	4.28E-04	--	--
EVM0010327	237.03	31.74	2.90	5.00E-05	4.28E-04	--	--
EVM0001730	750.97	99.93	2.91	5.00E-05	4.28E-04	Energy production and conversion	Malate synthase
EVM0009383	9.17	1.22	2.91	5.00E-05	4.28E-04	Amino acid transport and metabolism	Aminotransferase class IV
EVM0002645	150.45	19.76	2.93	5.00E-05	4.28E-04	Signal transduction mechanisms	Protein kinase domain
EVM0007893	51.12	6.69	2.93	5.00E-05	4.28E-04	--	Protein of unknown function (DUF3533)
EVM0007059	48.92	6.40	2.93	5.00E-05	4.28E-04	--	Domain of unknown function (DUF3415)
EVM0000662	70.34	9.13	2.95	5.00E-05	4.28E-04	--	--
EVM0002134	11.30	1.46	2.95	7.00E-04	4.23E-03	--	Caspase domain
EVM0009701	463.65	60.04	2.95	5.00E-05	4.28E-04	Posttranslational modification, protein turnover, chaperones	Glutathione peroxidase
EVM0006753	130.77	16.82	2.96	5.00E-05	4.28E-04	General function prediction only	Family of unknown function (DUF706)
EVM0006084	129.27	16.25	2.99	5.00E-05	4.28E-04	Secondary metabolites biosynthesis, transport and catabolism	Alcohol dehydrogenase GroES-like domain

EVM0006874	13.57	1.70	2.99	7.50E-04	4.49E-03	--	--
EVM0000446	208.02	26.06	3.00	5.00E-05	4.28E-04	Signal transduction mechanisms	Carboxylesterase family
EVM0003089	9.06	1.13	3.00	4.95E-03	2.13E-02	General function prediction only	Uncharacterised protein family (UPF0172)
EVM0004026	1301.47	162.68	3.00	5.00E-05	4.28E-04	Secondary metabolites biosynthesis, transport and catabolism	short chain dehydrogenase
EVM0007441	12.62	1.57	3.01	5.00E-05	4.28E-04	General function prediction only	Sugar (and other) transporter
EVM0003880	28.88	3.58	3.01	5.00E-05	4.28E-04	--	Formate/nitrite transporter
EVM0010708	133.79	16.50	3.02	5.00E-05	4.28E-04	--	Cerato-platanin
EVM0011713	17.74	2.17	3.03	5.00E-05	4.28E-04	Secondary metabolites biosynthesis, transport and catabolism	Cytochrome P450
EVM0008898	2222.79	270.45	3.04	5.00E-05	4.28E-04	--	--
EVM0010535	20.87	2.53	3.04	4.00E-04	2.65E-03	--	NmrA-like family
EVM0010323	297.16	36.00	3.05	5.00E-05	4.28E-04	Inorganic ion transport and metabolism	ATP-sulfurylase

EVM0000114	30.54	3.70	3.05	5.00E-05	4.28E-04	Posttranslational modification, protein turnover, chaperones	ATPase family associated with various cellular activities (AAA)
EVM0001558	589.99	70.38	3.07	5.00E-05	4.28E-04	--	--
EVM0006267	839.97	99.17	3.08	5.00E-05	4.28E-04	Energy production and conversion	Aldo/keto reductase family
EVM0004677	972.61	114.42	3.09	5.00E-05	4.28E-04	--	Serine carboxypeptidase S28
EVM0007084	173.02	20.30	3.09	5.00E-05	4.28E-04	--	--
EVM0011488	39.65	4.64	3.10	5.00E-05	4.28E-04	--	--
EVM0009846	37.11	4.33	3.10	5.00E-05	4.28E-04	General function prediction only	Sugar (and other) transporter
EVM0000802	260.31	30.23	3.11	5.00E-05	4.28E-04	--	--
EVM0008746	89.10	10.32	3.11	5.00E-05	4.28E-04	--	UvrD-like helicase C-terminal domain
EVM0009182	62.42	7.19	3.12	5.00E-05	4.28E-04	Function unknown	Major Facilitator Superfamily
EVM0011704	73.55	8.35	3.14	5.00E-05	4.28E-04	Inorganic ion transport and metabolism	ZIP Zinc transporter
EVM0008112	1059.00	120.13	3.14	5.00E-05	4.28E-04	Intracellular trafficking, secretion, and vesicular transport	ER lumen protein retaining receptor

EVM0001510	2069.18	234.08	3.14	5.00E-05	4.28E-04	Posttranslational modification, protein turnover, chaperones	Eukaryotic aspartyl protease
EVM0000833	177.97	19.89	3.16	5.00E-05	4.28E-04	Amino acid transport and metabolism	Isocitrate/isopropylmalate dehydrogenase
EVM0010518	3.18	0.35	3.17	6.40E-03	2.62E-02	--	--
EVM0007992	5.38	0.60	3.17	1.33E-02	4.70E-02	Carbohydrate transport and metabolism	Major intrinsic protein
EVM0006001	94.26	10.42	3.18	5.00E-05	4.28E-04	--	--
EVM0008735	870.16	95.73	3.18	5.00E-05	4.28E-04	--	NmrA-like family
EVM0004522	115.91	12.71	3.19	5.00E-05	4.28E-04	--	--
EVM0000923	78.37	8.59	3.19	5.00E-05	4.28E-04	Translation, ribosomal structure and biogenesis	Elongation factor Tu GTP binding domain
EVM0006386	244.37	26.75	3.19	5.00E-05	4.28E-04	--	--
EVM0012269	527.57	56.58	3.22	5.00E-05	4.28E-04	Cell wall/membrane/envelope biogenesis	SIS domain
EVM0002401	114.15	12.24	3.22	5.00E-05	4.28E-04	General function prediction only	O-methyltransferase
EVM0006758	12.78	1.37	3.22	7.75E-03	3.06E-02	--	--

EVM0008863	1412.91	149.72	3.24	5.00E-05	4.28E-04	--	Protein of unknown function (DUF3759)
EVM0009907	1.43	0.15	3.24	3.95E-03	1.77E-02	--	Aldo/keto reductase family
EVM0002747	1.61	0.17	3.24	5.50E-04	3.46E-03	RNA processing and modification	DEAD/DEAH box helicase
EVM0009621	23.87	2.51	3.25	5.00E-05	4.28E-04	--	--
EVM0000478	71.73	7.41	3.27	5.00E-05	4.28E-04	Signal transduction mechanisms	Protein kinase domain
EVM0004000	87.94	9.07	3.28	5.00E-05	4.28E-04	Carbohydrate transport and metabolism	Glucose-6-phosphate dehydrogenase, C-terminal domain
EVM0012087	291.82	30.09	3.28	5.00E-05	4.28E-04	General function prediction only	Animal haem peroxidase
EVM0003814	2.45	0.25	3.29	2.00E-04	1.46E-03	General function prediction only	GMC oxidoreductase
EVM0003996	242.12	24.81	3.29	5.00E-05	4.28E-04	--	--
EVM0006352	192.51	19.44	3.31	5.00E-05	4.28E-04	General function prediction only	short chain dehydrogenase
EVM0010745	14.85	1.49	3.32	5.00E-05	4.28E-04	--	--
EVM0001818	87.38	8.75	3.32	5.00E-05	4.28E-04	--	3'-5' exonuclease

EVM0007048	65.57	6.56	3.32	5.00E-05	4.28E-04	--	--
EVM0011328	41.48	4.14	3.32	5.00E-05	4.28E-04	RNA processing and modification	Ribonuclease III domain
EVM0005207	45.78	4.56	3.33	5.00E-05	4.28E-04	--	Ubiquitin family
EVM0002787	2575.10	253.63	3.34	5.00E-05	4.28E-04	Posttranslational modification, protein turnover, chaperones	Thioredoxin
EVM0008710	369.93	36.32	3.35	5.00E-05	4.28E-04	Posttranslational modification, protein turnover, chaperones	Subtilase family
EVM0005984	31.67	3.11	3.35	5.00E-05	4.28E-04	--	CVNH domain
EVM0009153	138.17	13.55	3.35	5.00E-05	4.28E-04	--	--
EVM0010847	649.73	63.53	3.35	5.00E-05	4.28E-04	--	Leucine Rich repeats (2 copies)
EVM0012135	42.60	4.15	3.36	5.00E-05	4.28E-04	--	--
EVM0004211	279.23	27.18	3.36	5.00E-05	4.28E-04	Replication, recombination and repair	ATPase family associated with various cellular activities (AAA)
EVM0004670	82.48	8.00	3.37	5.00E-05	4.28E-04	General function prediction only	Major Facilitator Superfamily
EVM0011124	164.93	15.93	3.37	5.00E-05	4.28E-04	--	Dioxygenase

EVM0007164	595.57	56.91	3.39	5.00E-05	4.28E-04	--	Dienelactone hydrolase family
EVM0002720	70.31	6.64	3.40	5.00E-05	4.28E-04	--	Serine carboxypeptidase S28
EVM0008923	2.37	0.22	3.43	3.25E-03	1.51E-02	--	--
EVM0009217	79.93	7.41	3.43	5.00E-05	4.28E-04	Secondary metabolites biosynthesis, transport and catabolism	Cytochrome P450
EVM0008962	324.22	29.98	3.43	5.00E-05	4.28E-04	General function prediction only	MOSC domain
EVM0001489	592.86	54.70	3.44	5.00E-05	4.28E-04	General function prediction only	Enoyl-(Acyl carrier protein) reductase
EVM0000637	159.66	14.72	3.44	5.00E-05	4.28E-04	--	Cytochrome P450
EVM0006816	774.23	70.01	3.47	5.00E-05	4.28E-04	--	--
EVM0004480	46.13	4.17	3.47	5.00E-05	4.28E-04	--	Glycosyl hydrolases family 15
EVM0011006	171.49	15.43	3.47	5.00E-05	4.28E-04	--	Protein of unknown function (DUF1524)
EVM0010117	72.60	6.47	3.49	5.00E-05	4.28E-04	--	--
EVM0010473	225.11	19.98	3.49	5.00E-05	4.28E-04	Secondary metabolites biosynthesis, transport and catabolism	Multicopper oxidase

EVM0005722	1056.14	91.94	3.52	5.00E-05	4.28E-04	General function prediction only	O-methyltransferase
EVM0001888	16.48	1.42	3.53	5.00E-05	4.28E-04	--	--
EVM0002639	9.80	0.83	3.55	2.90E-03	1.38E-02	--	Velvet factor
EVM0000934	10.34	0.88	3.56	5.00E-05	4.28E-04	--	Alpha/beta hydrolase family
EVM0002000	177.29	14.98	3.56	5.00E-05	4.28E-04	Posttranslational modification, protein turnover, chaperones	Prolyl oligopeptidase, N-terminal beta-propeller domain
EVM0001306	2.12	0.18	3.58	2.90E-03	1.38E-02	Lipid transport and metabolism	C2 domain
EVM0006726	90.72	7.46	3.60	5.00E-05	4.28E-04	Posttranslational modification, protein turnover, chaperones	ATPase family associated with various cellular activities (AAA)
EVM0002253	9.44	0.76	3.64	5.00E-05	4.28E-04	--	--
EVM0003142	3.58	0.28	3.67	1.30E-03	7.10E-03	Carbohydrate transport and metabolism	Major Facilitator Superfamily
EVM0002760	178.07	13.98	3.67	5.00E-05	4.28E-04	--	--
EVM0010044	62.48	4.90	3.67	5.00E-05	4.28E-04	--	--
EVM0000566	80.06	6.12	3.71	5.00E-05	4.28E-04	--	Cytochrome P450

EVM0010121	83.49	6.31	3.72	5.00E-05	4.28E-04	--	--
EVM0007799	7.54	0.57	3.73	5.00E-05	4.28E-04	--	Glycosyl hydrolases family 18
EVM0008197	143.10	10.63	3.75	5.00E-05	4.28E-04	Amino acid transport and metabolism	Carbon-nitrogen hydrolase
EVM0002710	219.48	16.10	3.77	5.00E-05	4.28E-04	--	Aldo/keto reductase family
EVM0011914	19.87	1.42	3.80	1.50E-04	1.14E-03	--	Cerato-platanin
EVM0010876	59.00	4.20	3.81	5.00E-05	4.28E-04	--	Fungal Zn(2)-Cys(6) binuclear cluster domain
EVM0008614	57.31	4.04	3.83	5.00E-05	4.28E-04	--	--
EVM0000680	114.15	7.99	3.84	5.00E-05	4.28E-04	Secondary metabolites biosynthesis, transport and catabolism	Cytochrome P450
EVM0009154	182.54	12.76	3.84	5.00E-05	4.28E-04	--	--
EVM0002822	190.24	13.16	3.85	5.00E-05	4.28E-04	--	Caspase domain
EVM0005498	381.86	26.37	3.86	5.00E-05	4.28E-04	--	--
EVM0000510	171.85	11.80	3.86	5.00E-05	4.28E-04	--	--

EVM0008821	44.97	3.05	3.88	5.00E-05	4.28E-04	--	Glycosyl hydrolases family 25
EVM0008117	181.98	12.00	3.92	5.00E-05	4.28E-04	--	--
EVM0008284	46.22	3.02	3.93	5.00E-05	4.28E-04	Amino acid transport and metabolism	Arginase family
EVM0008750	118.92	7.76	3.94	5.00E-05	4.28E-04	General function prediction only	Major Facilitator Superfamily
EVM0012233	79.77	5.11	3.97	5.00E-05	4.28E-04	--	--
EVM0009138	66.41	4.25	3.97	5.00E-05	4.28E-04	--	--
EVM0003104	443.70	28.10	3.98	5.00E-05	4.28E-04	General function prediction only	Major Facilitator Superfamily
EVM0012248	15.02	0.93	4.01	5.00E-05	4.28E-04	--	Protein of unknown function (DUF1399)
EVM0005724	417.58	25.34	4.04	5.00E-05	4.28E-04	--	Protein of unknown function (Ytp1)
EVM0001981	378.57	22.43	4.08	5.00E-05	4.28E-04	--	HD domain
EVM0002854	137.65	8.14	4.08	5.00E-05	4.28E-04	Secondary metabolites biosynthesis, transport and catabolism	Multicopper oxidase
EVM0009418	2053.38	120.46	4.09	5.00E-05	4.28E-04	--	--

EVM0008010	24.07	1.41	4.09	5.00E-05	4.28E-04	--	--
EVM0007102	572.04	32.96	4.12	5.00E-05	4.28E-04	--	--
EVM0003903	75.22	4.32	4.12	5.00E-05	4.28E-04	--	--
EVM0009696	63.62	3.64	4.13	5.00E-05	4.28E-04	Amino acid transport and metabolism	Aminotransferase class-V
EVM0004904	86.10	4.84	4.15	5.00E-05	4.28E-04	--	FAD binding domain
EVM0008387	394.95	22.13	4.16	5.00E-05	4.28E-04	--	--
EVM0002281	218.98	12.27	4.16	5.00E-05	4.28E-04	--	--
EVM0004458	89.15	4.94	4.17	5.00E-05	4.28E-04	--	--
EVM0011894	1198.07	65.91	4.18	5.00E-05	4.28E-04	Secondary metabolites biosynthesis, transport and catabolism	Cytochrome P450
EVM0011907	188.82	10.31	4.19	5.00E-05	4.28E-04	Coenzyme transport and metabolism	Polyprenyl synthetase
EVM0006935	56.65	3.08	4.20	5.00E-05	4.28E-04	--	UbiA prenyltransferase family
EVM0007914	24.61	1.33	4.21	5.00E-05	4.28E-04	--	--

EVM0003022	4138.79	216.17	4.26	5.00E-05	4.28E-04	Amino acid transport and metabolism	FAD dependent oxidoreductase
EVM0007317	11.61	0.60	4.26	5.00E-05	4.28E-04	--	Protein of unknown function (DUF1399)
EVM0010171	201.24	10.28	4.29	5.00E-05	4.28E-04	Energy production and conversion	Aldo/keto reductase family
EVM0004633	48.08	2.42	4.31	1.65E-03	8.65E-03	--	--
EVM0007038	9.24	0.45	4.35	6.50E-04	3.98E-03	--	--
EVM0009981	11.99	0.59	4.35	5.00E-05	4.28E-04	--	--
EVM0011428	100.27	4.75	4.40	5.00E-05	4.28E-04	Inorganic ion transport and metabolism	Sulfate transporter family
EVM0010332	44.42	2.09	4.41	5.00E-05	4.28E-04	--	--
EVM0005069	23.11	1.08	4.42	5.00E-05	4.28E-04	Energy production and conversion	FAD binding domain
EVM0012182	52.35	2.43	4.43	5.00E-05	4.28E-04	--	Glutathione S-transferase, N-terminal domain
EVM0006219	197.00	9.00	4.45	5.00E-05	4.28E-04	--	Serine carboxypeptidase
EVM0004142	1201.01	54.82	4.45	5.00E-05	4.28E-04	Intracellular trafficking, secretion, and vesicular transport	Vacuolar sorting protein 39 domain 1

EVM0006589	3.77	0.17	4.46	5.75E-03	2.41E-02	Carbohydrate transport and metabolism	Transaldolase
EVM0007634	191.31	8.67	4.46	5.00E-05	4.28E-04	--	--
EVM0003422	753.46	33.74	4.48	5.00E-05	4.28E-04	General function prediction only	Zinc-binding dehydrogenase
EVM0009739	636.83	27.95	4.51	5.00E-05	4.28E-04	Energy production and conversion	Aldo/keto reductase family
EVM0008958	109.72	4.48	4.61	5.00E-05	4.28E-04	--	--
EVM0002125	126.72	4.99	4.67	5.00E-05	4.28E-04	General function prediction only	Major Facilitator Superfamily
EVM0007544	25.66	1.00	4.68	5.00E-05	4.28E-04	Posttranslational modification, protein turnover, chaperones	Eukaryotic aspartyl protease
EVM0002085	822.30	30.87	4.74	5.00E-05	4.28E-04	--	Major Facilitator Superfamily
EVM0003690	58.60	2.03	4.85	5.00E-05	4.28E-04	--	Caspase domain
EVM0006864	967.54	32.73	4.89	5.00E-05	4.28E-04	--	Glyoxal oxidase N-terminus
EVM0009934	425.94	13.64	4.96	5.00E-05	4.28E-04	--	Uncharacterized conserved protein (DUF2183)
EVM0009784	110.14	3.49	4.98	5.00E-05	4.28E-04	Energy production and conversion	D-isomer specific 2-hydroxyacid dehydrogenase, NAD binding domain

EVM0002904	466.31	13.74	5.09	5.00E-05	4.28E-04	--	--
EVM0000127	1938.82	56.25	5.11	5.00E-05	4.28E-04	--	--
EVM0003743	30.49	0.88	5.12	5.00E-05	4.28E-04	Carbohydrate transport and metabolism	Major Facilitator Superfamily
EVM0001170	13.43	0.38	5.14	2.65E-03	1.28E-02	Inorganic ion transport and metabolism	ChaC-like protein
EVM0003059	335.60	9.02	5.22	5.00E-05	4.28E-04	Carbohydrate transport and metabolism	Glucosamine-6-phosphate isomerases/6-phosphogluconolactonase
EVM0007243	89.66	2.02	5.47	5.00E-05	4.28E-04	Secondary metabolites biosynthesis, transport and catabolism	Pyridine nucleotide-disulphide oxidoreductase
EVM0005595	36.50	0.79	5.53	5.00E-05	4.28E-04	--	Serine carboxypeptidase
EVM0000291	819.94	16.96	5.60	5.00E-05	4.28E-04	General function prediction only	GMC oxidoreductase
EVM0003051	504.27	9.48	5.73	5.00E-05	4.28E-04	--	--
EVM0000195	403.22	7.38	5.77	5.00E-05	4.28E-04	--	Serine carboxypeptidase S28
EVM0004715	138.65	2.47	5.81	5.00E-05	4.28E-04	--	--
EVM0005846	477.23	8.48	5.81	5.00E-05	4.28E-04	--	--

EVM0011539	51.89	0.90	5.85	5.00E-05	4.28E-04	General function prediction only	Uncharacterized alpha/beta hydrolase domain (DUF2235)
EVM0010247	467.59	7.52	5.96	5.00E-05	4.28E-04	Secondary metabolites biosynthesis, transport and catabolism	Cytochrome P450
EVM0001865	191.28	2.96	6.01	5.00E-05	4.28E-04	--	Pregnancy-associated plasma protein-A
EVM0008864	539.20	7.85	6.10	5.00E-05	4.28E-04	Energy production and conversion	Acetyl-CoA hydrolase/transferase N-terminal domain
EVM0001256	165.46	2.17	6.25	5.00E-05	4.28E-04	--	LVIVD repeat
EVM0006129	135.47	1.63	6.38	1.26E-02	4.49E-02	Posttranslational modification, protein turnover, chaperones	Subtilase family
EVM0001579	1118.44	11.76	6.57	5.00E-05	4.28E-04	General function prediction only	Family of unknown function (DUF706)
EVM0007573	4893.13	49.42	6.63	5.00E-05	4.28E-04	--	Fungal hydrophobin
EVM0008755	1761.64	17.35	6.67	5.00E-05	4.28E-04	--	Cupin domain
EVM0000645	2914.00	26.24	6.79	5.00E-05	4.28E-04	--	Hemopexin
EVM0010621	264.23	2.18	6.92	5.00E-05	4.28E-04	--	Deuterolysin metalloprotease (M35) family

EVM0011356	126.07	0.89	7.14	5.00E-05	4.28E-04	--	Glyoxal oxidase N-terminus
EVM0007266	2994.17	19.52	7.26	5.00E-05	4.28E-04	--	--
EVM0002793	4387.83	22.24	7.62	5.00E-05	4.28E-04	--	Cerato-platanin

Data Set S6 FPKM values for common DEGs of mushroom *P. eryngii* after *E. beijingensis* infection

gene ID	R1	R2	R3	Z1	Z2	Z3
EVM0006637	7.81	10.70	11.63	18.76	22.49	16.74
EVM0001573	1.30	1.66	1.69	4.00	5.95	3.71
EVM0005185	75.62	136.66	78.03	436.79	437.06	311.32
EVM0001452	5.34	6.49	3.30	21.38	35.11	26.78
EVM0008871	70.49	56.73	64.60	291.35	246.60	256.09
EVM0008898	1826.72	1620.69	2222.79	325.60	255.57	270.45
EVM0006594	269.00	421.38	689.09	118.87	115.37	245.31
EVM0010252	34.88	43.86	40.89	139.03	134.34	157.55
EVM0011675	37.64	31.01	26.10	9.43	10.86	8.51
EVM0002266	221.11	300.51	177.80	990.92	872.36	930.24
EVM0003976	145.42	195.19	127.76	43.18	55.88	42.32
EVM0009720	9.13	8.79	10.12	40.88	38.33	48.42
EVM0006068	2.89	2.92	5.92	10.66	17.08	12.16
EVM0001750	10.95	15.06	29.30	3.65	3.66	4.95
EVM0000131	2.44	5.59	3.95	10.51	11.76	11.08

EVM0007670	30.91	22.98	19.18	247.19	153.28	106.90
EVM0008628	32.71	20.34	17.86	9.17	9.18	7.42
EVM0008224	180.18	126.92	138.01	433.73	476.01	501.58
EVM0006099	83.14	130.16	92.45	241.37	365.45	395.05
EVM0000147	1.41	3.35	2.92	6.75	11.64	13.65
EVM0000467	2.76	2.39	4.37	19.06	14.03	18.75
EVM0009709	4.74	8.36	32.49	56.94	83.39	117.72
EVM0009146	0.39	1.57	0.76	3.17	4.62	4.78
EVM0012262	13.02	10.72	9.14	29.18	25.85	30.04
EVM0011230	8.09	12.94	17.92	74.23	98.61	67.68
EVM0008633	28.94	34.63	43.13	88.35	82.00	87.74
EVM0005216	46.67	44.31	46.06	108.37	110.45	152.40
EVM0006468	15.66	16.56	13.60	37.70	36.95	49.85
EVM0006335	118.87	68.22	116.74	245.18	372.66	422.08
EVM0006895	2.87	2.96	2.86	7.03	6.71	8.34
EVM0005135	8.93	11.96	13.43	20.49	27.52	30.55
EVM0009047	17.25	17.78	19.41	36.39	42.69	43.44
EVM0004085	201.70	274.18	1424.44	545.36	814.21	278.23
EVM0006976	19.35	13.80	13.03	41.25	40.89	44.26
EVM0010633	1350.09	739.01	518.20	3170.56	3281.28	5169.30
EVM0004688	26.10	23.99	18.59	121.36	93.72	75.99
EVM0011391	22.54	30.67	33.33	64.51	81.24	73.74
EVM0003817	5.13	5.59	4.77	20.70	20.50	18.81
EVM0003678	2.80	4.71	5.28	9.07	10.64	12.82
EVM0004031	11.09	10.64	9.34	158.24	124.33	141.70
EVM0007517	98.50	55.71	15.36	392.66	294.22	224.72

EVM0004818	11.25	13.48	10.11	41.23	44.44	107.07
EVM0007572	2.86	3.94	3.48	14.88	18.95	16.41
EVM0001336	53.25	37.80	64.58	25.30	17.30	26.20
EVM0008791	14.59	7.34	6.50	38.42	34.32	29.07
EVM0003855	4.12	4.75	3.50	13.64	13.38	18.23
EVM0006461	7.64	6.93	6.25	16.65	16.14	18.29
EVM0006795	1494.96	860.74	461.49	3582.30	2314.38	1683.99
EVM0010882	265.72	231.63	160.85	184.44	229.77	274.60
EVM0000252	18.24	16.06	15.85	36.90	32.99	42.69
EVM0011482	17.82	22.08	32.31	81.69	79.69	96.74
EVM0010721	5.31	6.47	7.09	1.81	1.87	1.96
EVM0001778	124.96	135.45	110.15	362.45	413.61	383.48
EVM0011691	9.28	12.75	12.58	26.36	28.52	48.57
EVM0002995	8.80	9.37	8.67	42.90	40.07	30.74
EVM0010015	0.34	0.66	0.37	1.46	1.98	3.03
EVM0009958	13.74	13.22	11.49	28.17	28.26	30.55
EVM0005225	16.22	19.80	39.79	25.31	14.44	17.46
EVM0006316	5.93	5.94	7.60	16.19	15.11	17.11
EVM0009341	9.53	10.40	10.72	28.18	24.81	30.32
EVM0008305	10.53	9.82	14.15	32.76	28.69	38.34
EVM0006301	28.63	31.86	123.07	74.11	97.07	40.33
EVM0008173	1.14	1.71	1.48	3.25	3.44	6.30
EVM0011757	4.27	3.67	1.95	9.82	10.83	8.88
EVM0000347	285.86	249.76	292.07	97.62	113.76	69.67
EVM0008335	59.07	49.06	36.68	20.26	21.15	17.14
EVM0011462	34.17	33.58	34.50	10.00	12.20	9.99

EVM0001350	357.09	145.73	376.65	1686.44	1864.80	1027.81
EVM0002963	54.85	100.39	72.68	21.60	31.23	31.53
EVM0003818	15.18	15.05	14.42	37.33	40.20	34.63
EVM0004778	31.63	62.74	27.45	194.28	276.37	352.76
EVM0002814	132.04	131.54	94.54	289.05	305.86	252.69
EVM0008835	35.82	55.75	32.71	88.70	149.26	136.13
EVM0001119	40.49	43.58	40.52	20.03	19.90	19.39
EVM0001810	290.27	190.23	283.77	124.20	64.65	104.23
EVM0000800	428.60	309.62	162.68	906.54	760.97	367.38
EVM0008064	17.02	12.24	10.34	35.15	31.56	33.26
EVM0004677	131.80	170.33	972.61	38.71	58.57	114.42
EVM0003103	59.11	58.14	52.01	297.01	208.62	237.94
EVM0011882	66.19	43.81	28.65	21.58	16.90	139.63
EVM0007905	18.04	19.62	13.87	39.74	44.43	55.09
EVM0001437	19.76	18.61	21.06	45.14	41.96	43.52
EVM0009691	36.99	31.92	22.42	76.22	83.08	75.39
EVM0005968	6.06	5.69	2.09	28.61	23.71	18.53
EVM0004038	1552.61	1024.34	1161.22	4225.45	2468.78	3149.55
EVM0012015	15.29	17.15	20.09	40.16	42.76	54.81
EVM0011621	51.54	60.74	49.66	138.09	129.47	108.33
EVM0009995	5.73	4.67	6.51	26.87	25.30	26.74
EVM0006946	18.89	10.95	9.90	67.22	40.71	30.38
EVM0009527	4.57	6.09	5.81	26.90	22.17	24.15
EVM0002148	67.67	32.23	18.94	851.70	669.23	844.41
EVM0012244	0.58	1.18	0.73	4.57	4.69	4.64
EVM0002993	7.18	10.03	13.20	17.86	20.82	27.54

EVM0008404	13.39	15.89	11.35	44.93	38.43	39.03
EVM0012102	17.50	18.46	18.41	40.60	41.74	44.99
EVM0010344	1807.48	1055.50	1120.67	3862.04	3949.47	5556.24
EVM0005285	1421.95	1034.32	755.82	6412.12	4536.14	5491.75
EVM0007162	15.05	14.24	18.75	64.07	51.71	46.92
EVM0010470	198.15	134.22	144.34	62.94	51.70	62.71
EVM0002623	12.13	10.92	8.06	25.21	23.11	26.15
EVM0000794	677.62	547.80	532.17	1891.66	1675.77	1984.75
EVM0005353	1.95	2.27	1.36	17.43	15.84	9.61
EVM0004549	312.60	324.29	201.58	1507.81	1646.46	1386.51
EVM0007837	2567.46	1876.37	1756.10	5847.03	3928.94	5291.10
EVM0007035	54.56	68.03	44.51	24.77	22.81	15.12
EVM0007826	24.23	23.42	19.64	63.15	64.42	70.79
EVM0010778	10.98	8.19	7.32	3.50	2.62	3.65
EVM0004358	181.56	154.92	195.63	604.80	554.44	658.94
EVM0004582	1.95	2.35	1.40	5.63	7.33	8.42
EVM0009399	19.92	19.78	28.13	74.45	60.91	64.87
EVM0006268	78.69	77.30	101.75	29.59	30.87	29.19
EVM0001552	13.45	13.94	12.27	55.19	55.87	41.72
EVM0007566	1914.58	1114.51	404.06	5997.38	4249.04	4360.24
EVM0006788	199.54	204.59	217.88	72.05	70.28	59.89
EVM0007065	120.18	74.32	45.00	304.16	195.25	124.25
EVM0008632	3.35	6.89	5.35	24.92	16.97	37.76
EVM0003548	9.49	8.36	6.31	33.24	31.64	28.99
EVM0004042	56.20	63.23	54.48	133.72	213.16	193.43
EVM0007796	29.71	54.09	23.12	65.68	181.52	116.93

EVM0011042	159.57	149.02	107.56	487.46	391.77	321.64
EVM0003373	3.94	2.09	0.93	9.27	7.70	13.38
EVM0008586	12.89	12.87	8.70	77.50	74.08	68.23
EVM0006314	2.66	4.06	1.77	7.89	9.95	11.01
EVM0003089	18.34	11.28	9.06	2.50	3.23	1.13
EVM0006561	6.30	7.62	4.00	24.23	21.48	17.61
EVM0011516	8.26	11.72	21.39	44.14	39.93	47.70
EVM0008302	76.36	33.75	49.03	163.78	84.87	103.98
EVM0002141	2.78	5.35	43.68	11.49	14.73	21.10
EVM0010493	7.85	10.22	26.31	26.03	24.50	58.24
EVM0011165	39.96	29.59	21.32	119.75	96.16	87.15
EVM0012070	1404.56	1068.93	1163.47	4015.74	2610.87	2743.83
EVM0006749	24.96	19.04	14.17	65.23	88.66	95.01
EVM0005012	2.15	4.61	3.68	13.66	10.52	18.32
EVM0009023	11.18	9.60	10.77	42.43	36.89	35.70
EVM0011687	37.08	46.34	33.85	10.72	9.58	8.79
EVM0000240	12.62	10.82	10.98	37.72	31.75	33.76
EVM0003528	111.38	80.61	21.72	480.04	456.82	301.01
EVM0002926	233.05	93.62	34.17	1117.65	956.06	725.08
EVM0001935	22.42	17.61	16.53	45.90	46.24	52.97
EVM0000282	155.66	157.30	213.49	1258.86	1159.63	1807.70
EVM0008890	11.81	17.09	18.59	5.85	8.53	6.96
EVM0008259	791.35	1280.14	843.42	251.28	541.81	294.90
EVM0011107	137.61	80.25	91.06	355.39	266.21	416.56
EVM0011808	7.72	8.34	7.83	5.45	4.25	6.78
EVM0006645	485.89	372.93	360.08	82.89	58.00	74.72

EVM0006896	412.41	214.29	66.84	1361.32	721.71	430.23
EVM0001466	13.98	14.05	13.74	4.67	5.73	4.38
EVM0002736	103.75	88.78	87.69	34.02	31.00	31.56
EVM0007949	12.09	14.57	10.48	56.70	44.95	53.00
EVM0005054	24.15	18.28	9.91	104.28	81.05	50.06
EVM0006132	24.32	57.10	85.94	174.52	185.61	229.13
EVM0011587	121.39	159.68	73.31	336.73	530.01	746.54
EVM0009204	10.20	10.50	11.51	31.67	30.76	34.94
EVM0006257	162.43	158.73	192.83	451.02	355.70	494.59
EVM0003296	214.70	142.35	183.26	580.61	427.41	397.72
EVM0008877	12.60	17.03	34.99	64.05	68.85	75.27
EVM0008982	1.33	3.01	1.22	6.25	10.57	17.36
EVM0007328	1928.08	1376.96	1407.82	4802.84	3142.71	4431.20
EVM0005703	18.56	21.51	24.18	54.75	59.51	52.82
EVM0000406	3.96	4.56	3.54	15.66	14.95	19.43
EVM0001342	29.29	31.02	26.47	153.60	144.47	110.10
EVM0010231	58.52	76.04	92.02	21.28	30.59	23.00
EVM0005865	439.25	229.42	356.73	2171.68	3627.32	4175.63
EVM0009699	51.54	59.57	59.94	308.49	257.35	227.55
EVM0003543	68.41	45.44	58.24	570.23	365.30	203.55
EVM0006648	3.63	4.23	10.86	11.48	16.81	3.84
EVM0009974	84.98	82.68	106.91	22.85	32.89	43.81
EVM0008622	99.12	75.43	60.22	256.65	223.50	278.68
EVM0000189	4.32	6.27	4.27	14.29	14.90	12.57
EVM0006060	5.47	5.94	4.96	12.67	15.14	19.72
EVM0005078	1.53	1.14	2.05	7.42	10.08	8.10

EVM0009103	7.65	5.56	3.60	21.83	30.53	17.60
EVM0001672	13.37	17.06	22.45	5.35	5.30	5.74
EVM0012202	1206.18	1409.84	2781.27	388.20	485.96	922.93
EVM0005323	3.08	5.98	6.86	18.96	15.72	23.13
EVM0007046	5.36	6.70	4.31	11.19	13.80	15.36
EVM0003008	72.60	55.18	23.24	208.02	142.78	237.88
EVM0009044	73.68	79.78	102.53	33.29	28.18	24.39
EVM0010702	11.31	11.60	12.09	43.06	35.03	50.43
EVM0003335	29.73	36.17	29.19	81.43	80.20	73.73
EVM0005614	92.97	93.07	105.86	27.28	27.97	27.76
EVM0012150	31.60	60.01	29.86	86.14	120.20	75.35
EVM0009153	56.94	26.15	138.17	3.85	5.75	13.55
EVM0009807	2341.01	1276.43	1330.92	7739.50	7974.27	11531.60
EVM0001405	143.68	143.94	169.09	57.08	56.30	62.70
EVM0005286	403.44	425.68	406.86	182.66	184.96	173.56
EVM0000264	0.97	1.10	0.51	2.37	5.88	1.37
EVM0001127	34.01	30.20	44.96	130.81	135.73	126.62
EVM0012049	3.07	2.62	3.32	39.20	39.34	59.99
EVM0010419	3.65	4.29	2.25	15.01	17.40	14.74
EVM0004577	27.55	24.45	24.64	78.20	52.61	52.64
EVM0011914	35.25	105.82	19.87	4.96	16.29	1.42
EVM0004173	11.83	22.04	66.71	52.31	57.02	140.40
EVM0008242	1.04	1.48	0.81	6.50	6.41	7.17
EVM0008112	670.65	718.63	1059.00	90.26	81.43	120.13
EVM0005227	61.43	55.84	39.94	137.05	158.73	135.16
EVM0000938	23.74	21.00	18.63	48.64	43.30	47.64

EVM0008739	374.83	362.39	394.01	120.64	131.42	101.13
EVM0010136	49.72	97.45	131.96	21.94	27.89	49.87
EVM0005980	46.60	41.85	5.06	19.03	20.92	30.28
EVM0000637	15.80	86.49	159.66	31.63	21.67	14.72
EVM0005665	79.76	98.29	131.64	83.52	65.06	35.23
EVM0007579	196.92	204.50	214.68	63.66	76.15	72.77
EVM0002694	4.83	4.22	3.97	13.70	12.82	13.92
EVM0008891	8.58	7.44	7.95	69.58	49.34	81.18
EVM0009404	14.69	12.49	15.36	66.19	69.28	78.43
EVM0007930	11.07	11.43	8.61	26.99	30.89	28.34
EVM0007361	17.76	17.90	19.72	57.99	49.81	62.71
EVM0008511	31.79	36.18	24.25	129.79	110.86	107.30
EVM0008438	16.67	19.82	19.61	38.81	39.78	49.36
EVM0007642	279.57	294.85	1836.29	12.38	19.17	286.69
EVM0009852	9.77	10.78	8.91	65.38	63.41	34.59
EVM0000435	51.56	45.83	26.09	139.96	158.90	230.91
EVM0000880	16.93	20.12	11.15	64.72	44.27	121.55
EVM0003129	3.16	2.64	2.58	9.28	10.21	8.56
EVM0003828	8.48	9.87	14.93	49.52	53.54	57.41
EVM0010247	33.91	268.99	467.59	14.40	17.39	7.52
EVM0007358	9.21	13.04	8.84	39.84	32.96	28.05
EVM0009599	5.50	5.52	3.50	1.16	1.27	1.18
EVM0006493	123.41	122.28	100.54	258.75	264.14	220.63
EVM0009981	3.71	3.40	11.99	0.49	0.81	0.59
EVM0010786	16.62	12.59	22.46	131.82	126.97	110.84
EVM0008465	29.96	29.83	46.77	327.87	304.46	319.96

EVM0007366	42.46	59.74	43.49	18.53	28.14	14.38
EVM0001135	109.20	101.93	87.88	77.23	80.76	55.88
EVM0008702	188.20	157.43	191.94	560.05	455.02	479.50
EVM0010921	10.80	12.13	11.95	38.09	50.96	50.39
EVM0006622	4.48	6.66	6.54	57.86	49.23	35.26
EVM0010530	40.07	57.40	95.47	230.12	212.30	204.71
EVM0004866	303.52	158.25	125.43	910.53	768.83	793.16
EVM0010583	443.78	329.63	268.20	902.05	973.19	1196.77
EVM0012239	2.11	1.76	0.99	7.20	5.43	5.37
EVM0007261	283.14	230.85	165.82	1209.34	1057.83	1267.30
EVM0008552	6.58	7.70	5.58	13.96	15.63	13.72
EVM0009840	14.42	17.24	11.08	36.54	38.79	39.39
EVM0007160	25.23	20.70	16.38	20.45	17.47	17.79
EVM0006868	746.83	824.81	762.13	290.39	295.30	188.25
EVM0004503	3.20	2.96	4.28	7.20	6.94	8.99
EVM0003318	29.54	27.53	39.89	173.34	159.23	202.36
EVM0005109	3.14	4.25	2.85	11.52	11.83	8.53
EVM0001419	184.77	216.66	179.59	478.79	575.26	505.13
EVM0002561	5.01	3.46	3.91	19.15	17.79	19.47
EVM0002222	8.09	8.41	9.31	4.00	3.16	3.44
EVM0010440	49.84	55.33	40.98	256.28	267.07	343.16
EVM0000390	10.65	8.67	4.02	31.92	27.02	29.31
EVM0008088	4.16	5.56	7.71	16.37	14.42	29.64
EVM0009031	4.48	3.81	4.37	24.64	20.03	33.21
EVM0001566	2.68	2.46	2.00	9.77	11.30	7.70
EVM0000151	253.39	186.42	301.75	865.86	1015.80	648.44

EVM0000318	5.06	3.51	3.10	0.63	0.64	0.62
EVM0009757	152.11	77.40	125.51	512.66	360.03	392.95
EVM0005744	12.23	9.94	8.96	2.07	2.37	3.90
EVM0005079	66.24	59.35	33.60	187.21	214.60	246.26
EVM0011840	15.75	17.63	17.37	38.66	41.60	34.77
EVM0007883	184.74	170.76	261.91	382.04	417.69	605.79
EVM0009544	81.36	58.48	87.78	33.60	25.57	13.78
EVM0006136	157.27	96.87	84.59	512.60	417.01	410.10
EVM0011806	977.12	609.02	647.26	1963.32	1380.14	1538.05
EVM0007712	48.98	37.77	28.42	9.10	9.99	8.63
EVM0008406	13.77	26.64	47.92	88.54	79.82	199.36
EVM0002114	223.28	154.53	182.00	80.09	67.49	65.92
EVM0008562	182.17	142.27	205.37	586.17	865.07	869.38
EVM0001738	1379.82	348.42	145.06	4240.67	1928.15	2200.62
EVM0010867	1471.16	928.29	382.18	3313.53	2378.57	1948.93
EVM0005272	811.62	408.78	404.12	1751.54	1587.89	2535.69
EVM0007568	178.71	127.56	104.78	526.23	303.20	381.71
EVM0002786	1913.05	1247.12	1521.28	4304.47	4081.63	3153.75
EVM0005279	429.38	619.87	307.74	1672.61	2080.40	1820.55
EVM0002125	8.30	20.91	126.72	2.55	3.05	4.99
EVM0010318	5.40	3.61	4.94	14.91	15.87	13.46
EVM0002270	2.33	2.93	4.45	26.09	11.86	46.89
EVM0005755	76.65	56.14	121.21	32.65	21.40	21.02
EVM0006805	100.27	133.31	107.23	412.25	294.00	374.56
EVM0010856	46.63	41.65	28.50	17.30	15.35	12.93
EVM0003787	76.12	67.47	57.92	157.01	150.51	164.19

EVM0006402	133.80	164.77	24.68	432.17	428.53	454.50
EVM0012211	28.44	24.74	19.84	65.38	57.71	77.25
EVM0008337	15.72	25.78	72.85	106.59	56.76	27.80
EVM0007661	8.33	8.98	13.62	43.70	43.54	34.23
EVM0000209	7.71	8.50	7.53	24.46	23.39	22.63
EVM0007331	2.74	3.46	4.19	11.03	11.04	12.89
EVM0011153	189.73	189.03	135.74	383.39	441.33	570.89
EVM0008761	13.32	21.69	13.60	47.49	45.37	59.88
EVM0011971	10.24	8.85	9.04	33.93	30.81	23.05
EVM0003936	43.31	77.86	66.45	150.05	161.09	172.18
EVM0001178	20.65	21.49	27.67	67.30	78.95	81.84
EVM0007506	269.85	189.76	224.15	624.50	522.92	458.19
EVM0003029	23.91	31.14	30.01	172.29	156.58	106.91
EVM0011790	49.97	26.16	35.04	10.85	7.20	10.70
EVM0008010	4.63	4.56	24.07	0.71	0.28	1.41
EVM0010621	41.11	29.42	264.23	1.54	1.85	2.18
EVM0007602	11.40	10.24	5.84	30.24	23.67	18.86
EVM0006689	17.21	14.87	12.72	81.39	72.27	66.24
EVM0011658	7.13	8.49	5.66	1.60	1.80	2.57
EVM0006981	20.16	15.64	11.02	144.03	122.70	147.01
EVM0004635	65.35	75.47	101.07	550.54	464.42	382.58
EVM0008537	8018.90	9774.36	2311.19	20193.20	30297.40	14034.10
EVM0008710	157.51	275.29	369.93	5.11	7.98	36.32
EVM0001877	6.85	4.63	4.08	2.81	1.82	1.58
EVM0008011	47.05	65.93	979.97	98.22	170.62	193.57
EVM0006845	17.01	15.45	10.33	40.29	33.19	32.38

EVM0000593	97.57	100.34	135.73	341.37	261.18	362.89
EVM0002338	10.46	13.22	10.13	49.96	40.84	44.68
EVM0001647	62.00	48.79	35.86	191.21	145.80	189.11
EVM0003051	99.99	150.00	504.27	39.84	49.02	9.48
EVM0008519	150.41	124.61	122.32	416.66	347.64	444.91
EVM0005014	1.97	4.72	8.10	14.41	17.51	24.13
EVM0007872	34.34	31.65	63.49	106.21	98.52	231.38
EVM0006494	13.62	15.86	15.29	57.63	44.29	45.11
EVM0011068	30.01	37.34	76.82	111.26	291.05	359.63
EVM0007942	81.46	113.13	399.70	186.36	50.15	71.75
EVM0009908	413.33	352.00	220.37	1253.82	1255.26	960.96
EVM0010148	191.45	173.26	177.16	643.25	398.39	420.48
EVM0004379	196.64	228.26	235.10	591.88	483.53	595.80
EVM0004319	36.64	32.83	26.02	74.19	79.47	116.50
EVM0003574	48.42	36.69	38.63	103.96	101.76	121.03
EVM0011239	91.93	62.13	74.61	257.86	317.03	309.36
EVM0004716	31.56	31.48	37.97	26.40	24.36	25.03
EVM0001259	15.49	16.51	12.62	14.72	15.40	9.60
EVM0006727	42.96	43.81	32.36	17.73	15.40	15.37
EVM0001189	44.76	42.53	47.14	101.31	98.39	108.13
EVM0002682	38.96	47.46	65.55	373.60	389.54	435.15
EVM0003224	65.35	61.67	67.85	151.56	178.72	209.58
EVM0000666	103.37	55.68	56.27	247.26	174.32	148.08
EVM0007445	2.78	3.29	2.65	9.26	9.80	23.57
EVM0001888	8.68	15.78	16.48	3.60	4.04	1.42
EVM0011852	32.55	45.43	46.96	197.48	163.64	155.72

EVM0005497	51.81	60.73	53.46	226.99	235.18	274.54
EVM0006340	78.81	52.61	56.11	19.07	18.99	23.83
EVM0001591	1.97	1.17	1.57	8.24	5.80	6.29
EVM0007305	349.97	247.84	332.47	808.63	740.78	821.83
EVM0000195	12.77	46.72	403.22	4.71	7.06	7.38
EVM0004054	399.42	437.54	2923.38	1389.50	965.46	554.24
EVM0001964	8.12	7.02	4.59	38.57	46.30	29.32
EVM0001335	9.27	13.23	10.05	25.29	27.96	28.83
EVM0002673	29.18	20.51	4.93	120.27	57.42	315.29
EVM0007146	878.23	732.45	1280.47	5607.20	4368.04	4054.33
EVM0005500	16.81	11.98	13.73	34.99	38.59	42.56
EVM0004965	3.40	2.58	2.64	18.85	18.91	22.29
EVM0007573	5359.43	3633.65	4893.13	1206.28	847.04	49.42
EVM0002948	39.63	33.72	30.61	115.44	90.82	118.09
EVM0004840	490.75	299.94	276.31	197.64	134.47	111.05
EVM0002297	215.72	237.19	271.37	1306.69	834.10	564.48
EVM0010605	962.90	754.98	717.39	2217.45	1525.74	1870.07
EVM0011758	11.07	10.85	15.85	12.07	15.52	13.93
EVM0011344	9.73	9.71	9.92	35.54	29.66	34.83
EVM0005557	73.60	49.62	46.12	20.38	21.20	18.52
EVM0009851	11.76	14.31	12.02	35.66	36.24	36.93
EVM0000907	1.40	2.79	1.60	6.04	6.73	5.17
EVM0006820	17.51	19.02	40.31	112.24	99.54	193.40
EVM0006726	302.99	204.54	219.90	140.31	130.51	156.35
EVM0002996	26.17	22.09	17.46	61.25	57.76	62.69
EVM0006700	186.14	162.97	195.99	763.41	638.54	526.28

EVM0011432	7.75	9.06	7.16	52.00	49.27	43.40
EVM0005691	0.55	0.43	0.56	1.94	1.60	3.42
EVM0004710	164.60	186.95	129.07	370.26	391.17	506.83
EVM0006122	32.58	39.58	26.98	10.04	9.33	8.83
EVM0000598	87.02	66.97	64.58	40.01	32.93	25.72
EVM0007667	162.90	147.00	273.96	67.05	73.16	93.46
EVM0001421	43.49	51.01	50.04	106.35	161.48	193.70
EVM0002115	30.58	16.32	25.02	3.36	5.15	6.37
EVM0007827	19.19	96.01	423.46	241.11	238.04	192.64
EVM0005915	24.78	11.51	12.02	64.70	29.42	100.28
EVM0008192	156.48	232.76	298.23	76.59	101.56	43.62
EVM0002257	28.03	30.87	26.14	58.63	67.12	152.50
EVM0002150	131.46	170.20	312.70	745.19	654.75	639.48
EVM0008163	4.77	4.18	1.87	12.71	12.15	12.12
EVM0011446	2630.22	1632.45	1369.67	7170.90	4570.86	6690.14
EVM0005976	75.67	107.64	183.50	328.96	373.40	452.68
EVM0004575	99.93	139.05	187.92	42.19	49.21	64.19
EVM0011121	3.69	14.51	145.48	21.23	33.34	56.01
EVM0005881	3.51	4.08	2.01	18.31	15.48	19.68
EVM0010521	22.11	41.02	159.20	566.26	126.46	356.79
EVM0000366	1.37	3.30	5.23	5.43	7.14	12.37
EVM0005236	393.07	407.49	327.58	994.81	994.97	1092.18
EVM0003322	5.49	9.08	5.71	20.02	26.32	21.72
EVM0008058	6.15	11.63	9.76	35.18	42.04	40.64
EVM0005796	1156.13	1012.35	1292.18	5651.83	3312.97	4545.87
EVM0003903	18.22	39.03	75.22	7.84	7.38	4.32

EVM0010158	41.47	53.50	53.16	104.62	157.86	227.91
EVM0001192	69.88	105.27	64.77	323.20	359.10	336.77
EVM0005366	22.53	24.96	16.34	47.83	54.67	41.49
EVM0001632	27.70	36.49	37.05	127.49	97.53	111.93
EVM0000483	176.73	165.09	103.27	412.13	353.73	307.65
EVM0007507	17.68	13.98	11.64	110.38	112.47	101.72
EVM0007196	310.01	234.14	182.61	845.81	783.54	823.45
EVM0008237	17.56	19.35	24.44	74.37	67.33	69.26
EVM0003453	156.96	113.79	69.03	519.42	383.15	332.90
EVM0007650	57.53	120.79	114.01	369.31	310.96	311.92
EVM0000381	13.58	15.88	11.44	44.33	42.04	32.95
EVM0010983	5.20	5.99	2.80	14.17	12.64	12.29
EVM0001816	11.08	10.76	8.27	32.78	36.39	29.99
EVM0009109	42.66	53.80	30.09	165.97	114.39	104.04
EVM0009206	16.19	26.38	42.48	44.90	76.45	190.71
EVM0001355	262.49	215.77	149.31	861.95	638.75	872.19
EVM0011261	25.40	21.47	21.91	62.85	55.30	49.26
EVM0000423	141.39	152.25	89.05	464.75	425.44	491.73
EVM0003241	18.07	30.80	37.16	162.41	171.23	90.35
EVM0005583	13.13	16.59	16.56	60.65	47.99	56.97
EVM0010981	1041.63	1381.82	290.73	2689.99	3628.46	1516.73
EVM0011499	10.11	13.28	8.32	24.96	33.64	19.14
EVM0005431	7.25	8.59	7.22	18.99	21.08	25.93
EVM0011804	4.66	4.39	5.19	21.10	21.51	19.64
EVM0000819	3.52	3.21	2.35	19.76	20.74	15.96
EVM0002657	350.18	408.67	362.68	956.58	957.32	1066.26

EVM0010558	19.86	18.17	13.17	59.63	54.39	35.02
EVM0011517	17.34	15.58	20.19	65.39	54.91	48.48
EVM0010061	78.77	48.69	55.99	230.53	166.45	144.62
EVM0004775	20.46	19.25	21.60	98.91	75.95	44.25
EVM0011006	4.98	18.23	171.49	1.50	2.40	15.43
EVM0007042	96.16	54.21	38.39	2483.20	1542.16	1037.88
EVM0012010	48.69	20.73	22.49	110.02	94.44	218.63
EVM0005171	16.83	14.80	14.11	39.07	32.94	28.60
EVM0009051	454.36	296.43	283.50	913.61	981.43	1325.40
EVM0000184	110.04	152.63	59.07	12.76	14.70	10.70
EVM0001814	5017.20	5417.96	3962.69	40681.30	30257.30	31870.50
EVM0007446	1162.29	1034.64	1647.58	466.01	393.16	371.57
EVM0009999	3.31	8.29	3.21	38.62	23.05	33.16
EVM0009955	11.05	11.02	10.02	31.05	32.01	21.91
EVM0000005	33.92	34.87	18.74	73.56	74.87	52.99
EVM0004341	81.75	99.35	65.39	497.62	438.56	380.43
EVM0006717	16.65	12.48	19.89	39.01	52.79	51.66
EVM0006542	48.12	52.74	2175.70	288.67	251.58	466.37
EVM0010012	6.44	11.95	11.00	25.48	24.89	42.92
EVM0000596	6.54	6.95	3.89	14.83	15.62	14.13
EVM0003992	15.22	14.16	17.66	44.33	44.92	53.99
EVM0002923	5995.82	4250.88	2245.12	20036.90	12227.00	12605.60
EVM0005245	26.13	24.45	22.88	63.68	64.79	47.20
EVM0008872	1.78	2.84	1.63	4.78	6.82	7.39
EVM0002076	325.41	184.01	300.25	839.77	823.43	1075.35
EVM0008722	195.10	340.50	155.69	92.39	62.89	61.14

EVM0008782	25.90	21.60	25.09	13.10	10.78	11.03
EVM0001331	27.81	36.49	48.73	8.17	11.22	11.13
EVM0000062	20.49	20.77	12.99	99.66	113.10	160.27
EVM0002999	48.51	53.51	53.99	22.10	23.77	18.24
EVM0004233	26.26	14.27	16.74	356.42	375.05	447.19
EVM0008504	39.76	30.44	58.39	100.53	90.04	212.76
EVM0005367	1.19	1.61	0.91	8.82	6.05	6.84
EVM0005110	30.58	30.25	29.61	136.78	145.03	123.58
EVM0004346	263.09	363.40	314.22	2506.01	2552.93	4460.27
EVM0010336	59.89	37.24	35.49	147.96	131.06	180.14
EVM0006282	1.37	2.76	2.91	8.53	12.24	10.63
EVM0002542	145.03	183.23	138.25	403.02	448.04	453.78
EVM0009586	48.27	49.10	39.13	111.26	98.82	161.55
EVM0007746	19.35	28.10	28.42	9.62	9.75	7.67
EVM0008497	577.43	701.39	460.74	1463.92	1462.32	1821.91
EVM0011608	1106.38	874.64	717.36	3057.75	2092.67	2478.86
EVM0008830	36.80	59.83	36.90	795.37	434.89	170.55
EVM0003437	16.24	22.07	25.10	7.70	8.98	8.74
EVM0005372	11.46	22.57	21.08	4.32	4.92	3.86
EVM0001076	8.68	6.27	4.96	23.56	30.99	23.45
EVM0007524	8.54	7.41	5.23	22.04	15.64	13.09
EVM0001246	10.24	8.49	11.08	37.89	55.45	101.35
EVM0002358	18.38	11.05	13.68	63.88	47.36	71.67
EVM0002081	2.60	2.03	1.38	15.03	18.86	17.51
EVM0004028	92.88	62.79	64.29	185.86	205.30	225.73
EVM0004661	6.04	5.54	5.29	13.84	13.84	15.12

EVM0010446	15.39	18.81	36.52	113.29	101.30	138.75
EVM0011910	45.62	57.76	60.21	19.83	28.09	20.81
EVM0011085	39.91	32.62	23.83	96.40	82.46	98.97
EVM0003560	18.66	19.19	13.70	77.76	73.30	72.15
EVM0008363	46.52	54.23	43.74	94.51	113.56	127.52
EVM0011097	378.34	303.47	183.48	855.96	956.27	981.53
EVM0011343	60.87	28.02	19.88	150.59	111.97	328.37
EVM0003743	7.10	6.24	30.49	1.10	2.40	0.88
EVM0006251	4.83	3.49	5.01	43.80	41.06	56.48
EVM0007684	199.30	137.09	183.12	36.61	33.25	36.89
EVM0008651	112.98	86.60	93.46	232.33	224.47	270.68
EVM0001681	258.91	268.76	199.69	863.46	719.84	965.94
EVM0009732	14.88	18.80	10.68	42.98	60.80	35.72
EVM0005521	102.86	98.68	142.17	42.74	36.68	59.70
EVM0001809	129.36	98.29	81.04	313.62	278.85	261.96
EVM0000289	31.95	27.95	22.98	67.13	58.88	51.27
EVM0007958	1.73	2.34	2.44	6.09	8.05	21.40
EVM0000204	803.35	890.32	629.60	2257.79	2303.93	2450.84
EVM0002245	29.50	24.28	30.43	83.48	64.74	73.87
EVM0009138	2.81	8.14	66.41	15.19	2.78	4.25
EVM0002154	0.68	1.66	0.84	2.08	4.29	3.47
EVM0005312	0.97	1.12	1.72	3.34	2.31	3.82
EVM0009845	12.24	13.77	12.95	53.49	40.20	40.87
EVM0008746	49.40	59.21	89.10	12.72	12.92	10.32
EVM0004868	20.24	18.65	13.62	46.64	38.84	39.98
EVM0011907	18.74	51.08	188.82	8.44	16.32	10.31

EVM0002548	15.32	13.79	18.97	53.40	54.15	53.22
EVM0008198	40.47	39.94	38.19	117.60	118.44	110.46
EVM0007266	1958.60	1631.18	2994.17	24.78	39.90	19.52
EVM0010589	69.26	85.97	174.65	799.29	462.72	445.54
EVM0004931	73.24	69.14	67.18	36.10	33.47	31.56
EVM0009748	22.57	20.75	19.92	59.69	48.64	46.49
EVM0009512	25.84	32.12	21.62	54.50	76.60	72.49
EVM0006624	19.39	21.18	22.97	52.21	48.23	51.79
EVM0003558	38.60	31.97	65.17	125.61	93.52	144.81
EVM0009298	65.31	38.01	56.13	16.29	14.33	13.66
EVM0007629	63.87	60.68	63.26	153.46	170.63	159.57
EVM0004228	10.15	10.08	12.13	48.12	46.11	62.28
EVM0002723	6.08	11.98	42.95	21.03	53.00	96.28
EVM0009591	10.09	9.10	6.85	41.55	47.61	44.76
EVM0001737	64.51	97.00	43.05	15.72	20.42	9.19
EVM0004887	2.66	3.64	7.47	1.18	1.77	1.66
EVM0005861	157.11	76.15	9.29	450.22	358.24	197.13
EVM0009798	42.06	47.15	18.72	197.31	176.51	156.91
EVM0008227	41.23	34.98	34.62	12.26	11.47	13.08
EVM0000478	45.23	56.73	71.73	10.54	11.75	7.41
EVM0011786	13.44	10.99	22.04	85.69	81.86	83.21
EVM0000557	23.01	12.73	20.43	53.55	52.98	67.81
EVM0007751	100.21	78.58	116.36	27.07	30.98	31.79
EVM0011961	104.37	145.44	114.49	348.96	315.05	315.80
EVM0002743	16.42	12.34	9.03	46.85	48.46	37.61
EVM0007323	699.26	429.64	388.35	2059.06	1877.24	3065.78

EVM0011919	29.00	27.01	27.40	64.03	54.58	70.06
EVM0009169	97.58	589.43	1408.42	44.41	115.82	586.51
EVM0005073	11.11	10.32	11.11	29.62	32.96	40.97
EVM0001520	2.87	4.32	2.40	11.11	15.87	33.19
EVM0002302	18.03	22.38	21.43	65.06	66.30	60.80
EVM0002044	53.36	94.18	40.93	149.08	225.88	103.39
EVM0011366	12.21	14.56	14.13	26.99	30.73	30.62
EVM0004320	141.78	149.65	137.75	62.00	59.82	63.67
EVM0001489	52.74	73.96	592.86	26.35	28.26	54.70
EVM0006748	105.07	96.81	78.41	44.52	41.16	37.40
EVM0000363	12.35	9.96	10.99	49.78	40.60	47.64
EVM0001661	5.76	6.71	6.38	64.32	65.06	55.31
EVM0008966	24.44	22.04	23.39	102.00	194.61	151.06
EVM0006677	24.68	23.91	12.11	51.24	51.92	29.64
EVM0004297	32.24	21.23	24.02	137.10	128.12	104.81
EVM0006292	2.18	5.14	2.78	10.24	14.73	10.69
EVM0007852	779.47	799.16	384.44	4462.47	3705.76	2074.10
EVM0006225	138.68	85.30	57.88	31.39	27.53	26.43
EVM0002910	10.36	16.37	11.60	76.95	73.90	58.76
EVM0001584	35.33	39.66	34.37	86.44	89.56	132.95
EVM0007418	54.66	43.76	44.52	147.90	104.37	125.75
EVM0000933	5.16	3.88	5.96	0.63	1.01	1.13
EVM0008748	5.95	14.50	48.27	24.02	29.11	17.70
EVM0008356	13.38	12.11	9.76	55.97	48.78	42.01
EVM0002787	119.17	1001.29	2575.10	275.13	323.55	253.63
EVM0007225	72.33	74.26	121.76	258.85	570.70	592.66

EVM0006187	0.74	1.96	3.53	9.76	10.02	8.37
EVM0003134	58.00	60.76	32.98	16.17	8.07	10.41
EVM0009858	33.46	33.92	27.87	79.52	80.81	90.71
EVM0006629	43.15	44.42	45.38	12.20	15.84	17.78
EVM0001122	37.62	27.42	25.05	160.49	139.35	172.64
EVM0008889	0.91	0.86	1.23	1.71	1.62	3.02
EVM0008931	53.49	52.62	39.74	23.27	21.83	16.88
EVM0005506	4.46	6.00	4.39	11.58	13.32	21.88
EVM0009846	20.45	22.15	37.11	9.94	5.72	4.33
EVM0005619	16.97	9.47	10.10	43.57	35.23	39.74
EVM0008951	27.26	29.06	24.36	81.36	67.44	82.19
EVM0008230	15.30	17.37	24.18	66.75	58.98	74.24
EVM0006543	1.87	3.63	2.71	8.60	8.27	19.06
EVM0005265	32.85	47.44	110.83	260.64	230.58	281.64
EVM0003316	490.45	552.91	471.55	1118.51	1114.40	1089.32
EVM0006288	22.63	22.48	19.08	51.99	46.42	38.56
EVM0005679	23.45	18.98	70.13	10.16	8.17	9.42
EVM0000628	22.06	17.00	34.15	94.65	88.10	112.13
EVM0005515	2.40	9.55	17.78	6.77	3.54	3.90
EVM0003839	801.48	516.29	537.50	1889.09	1370.46	1654.23
EVM0008650	53.76	73.28	15.28	281.20	206.52	75.60
EVM0001562	143.45	135.12	139.15	38.42	60.05	55.68
EVM0002944	23.42	23.88	21.25	9.39	11.24	9.90
EVM0008343	11.12	6.92	7.63	34.89	31.11	31.31
EVM0007514	33.18	137.65	18.82	82.54	429.80	210.86
EVM0011240	257.40	225.96	299.49	111.20	88.62	109.41

EVM0000114	5.33	9.83	30.54	38.01	4.70	3.70
EVM0003721	254.23	166.50	84.33	596.65	461.01	438.78
EVM0000905	32.00	27.14	23.39	88.32	92.62	93.07
EVM0005845	45.97	32.84	29.53	16.02	14.43	10.30
EVM0005402	1.59	2.39	2.38	6.87	7.16	8.72
EVM0002732	51.09	68.12	128.68	19.86	12.32	20.56
EVM0007764	23.91	19.32	19.91	4.11	4.41	3.66
EVM0004958	8.98	9.86	11.61	37.18	54.76	106.06
EVM0008721	1.57	1.68	2.30	9.93	6.36	4.94
EVM0002952	34.73	29.64	27.88	15.18	14.68	13.22
EVM0006917	1.96	4.18	1.68	16.28	17.73	12.58
EVM0005087	137.39	91.68	91.19	280.83	232.42	311.07
EVM0007132	0.86	0.60	0.99	2.66	3.26	4.05
EVM0005203	45.67	38.75	36.14	15.76	17.21	17.59
EVM0012190	3.47	3.58	6.88	0.82	1.08	2.51
EVM0011123	62.48	59.11	49.31	25.21	21.40	19.16
EVM0011785	4.26	5.92	4.52	8.87	13.65	14.45
EVM0009049	16.16	11.25	7.76	3.00	1.37	2.37
EVM0006660	601.05	559.24	711.10	197.09	211.01	220.73
EVM0003583	661.53	822.18	56.60	213.60	289.96	1326.27
EVM0009651	3.21	4.96	4.91	12.01	11.60	12.97
EVM0006126	24.83	28.63	21.17	58.23	72.95	97.29
EVM0012187	8.45	10.51	10.73	22.91	22.08	28.82
EVM0003929	11.97	17.66	13.23	3.34	6.90	5.11
EVM0006117	11.59	14.98	9.64	40.49	32.42	25.75
EVM0010645	9.49	9.87	8.74	36.14	28.49	29.48

EVM0005139	323.22	160.92	37.04	1364.38	877.27	591.64
EVM0011013	14.04	11.45	6.39	67.94	64.79	55.62
EVM0012127	23.24	18.30	19.67	47.84	41.10	45.57
EVM0008720	251.03	239.89	208.73	99.23	104.78	71.42
EVM0000512	6.92	9.72	9.27	3.34	3.52	3.66
EVM0007593	128.29	108.89	110.22	277.65	246.16	237.91
EVM0009027	41.30	34.24	71.92	90.32	153.59	189.06
EVM0005145	30.58	31.58	38.58	4.98	12.04	13.03
EVM0008755	63.02	71.14	1761.64	8.99	9.38	17.35
EVM0010669	117.55	96.29	90.91	359.05	243.59	202.04
EVM0000211	29.99	31.24	24.18	64.00	62.49	65.38
EVM0003326	29.04	34.24	32.29	134.59	100.26	87.47
EVM0007277	769.92	590.66	708.74	1683.76	1235.39	1596.78
EVM0012095	7.11	7.23	7.67	22.21	18.64	18.66
EVM0011387	65.19	77.10	110.47	28.38	26.65	18.67
EVM0002121	25.61	22.65	19.84	8.95	7.49	5.97
EVM0010870	17.58	14.22	18.91	119.36	87.23	90.66
EVM0005036	19.90	18.21	14.34	91.96	89.13	101.37
EVM0003306	30.60	37.08	48.58	185.23	191.95	169.53
EVM0002558	11.74	11.05	11.62	35.51	27.09	24.16
EVM0002898	18.52	13.73	14.77	68.49	65.76	84.06
EVM0003416	22.42	25.30	22.70	79.59	51.63	54.03
EVM0010403	6.85	4.09	7.81	29.37	17.08	22.98
EVM0000419	56.33	47.31	44.43	183.64	163.41	175.18
EVM0008035	27.70	24.86	18.88	11.75	11.08	8.75
EVM0007187	2.41	5.62	3.62	15.76	25.43	26.03

EVM0002760	6.22	39.53	178.07	73.45	15.99	13.98
EVM0003745	4.61	5.02	6.42	12.31	13.20	14.65
EVM0004134	52.49	51.54	46.71	167.16	146.79	174.72
EVM0003609	366.16	221.00	122.45	966.62	771.77	841.86
EVM0004301	13.59	22.59	27.42	40.53	52.40	80.38
EVM0004405	1.28	1.91	1.28	4.38	4.66	7.75
EVM0006195	17.40	13.98	13.10	97.00	82.97	94.70
EVM0005253	643.40	425.02	213.46	2114.47	1332.13	1036.20
EVM0005127	77.37	87.46	51.92	748.41	713.49	657.87
EVM0002551	20.53	24.13	15.15	56.42	52.33	53.00
EVM0002953	14.48	38.71	180.59	3.32	9.67	70.64
EVM0001104	4.48	6.02	4.48	139.31	90.23	41.99
EVM0001256	98.71	107.28	165.46	5.30	3.62	2.17
EVM0008758	1.28	1.13	2.54	0.21	0.21	0.71
EVM0011475	5.23	6.49	5.42	20.53	23.79	21.76
EVM0010141	72.42	48.19	40.37	209.87	178.63	226.56
EVM0003167	14.40	13.76	37.21	181.82	215.43	516.16
EVM0006881	12.47	16.18	14.67	64.73	79.95	79.43
EVM0007451	105.78	84.78	78.06	37.32	34.11	32.40
EVM0008059	238.02	33.09	66.52	79.59	113.28	105.69
EVM0002805	149.26	128.92	117.02	300.83	295.79	398.44
EVM0005613	14.14	18.39	19.51	50.67	53.56	76.71
EVM0010422	5.58	4.63	4.02	42.54	42.69	45.67
EVM0010862	95.69	90.28	89.85	231.41	211.19	302.39
EVM0006570	792.75	718.07	502.56	3557.49	2755.72	2569.12
EVM0001473	24.18	25.08	20.28	59.25	54.27	58.50

EVM0005197	9.63	35.23	14.21	19.52	82.94	28.70
EVM0010575	22.57	20.51	14.56	53.30	44.72	48.88
EVM0007616	18.76	24.00	23.95	65.25	61.74	65.73
EVM0004461	16.00	14.63	32.27	40.31	77.34	82.20
EVM0008894	19.08	28.54	34.83	113.02	57.75	77.90
EVM0002854	44.72	96.47	137.65	93.53	27.12	8.14
EVM0009876	9.36	8.31	11.28	53.35	54.78	52.85
EVM0004513	60.65	76.28	82.31	204.10	186.79	230.98
EVM0010109	8.69	7.58	7.76	18.14	20.68	18.17
EVM0006915	19.97	28.66	21.29	115.05	105.38	74.44
EVM0008461	9.67	10.87	12.87	46.45	36.33	47.64
EVM0006626	51.89	45.70	65.78	257.00	312.50	205.11
EVM0005846	6.02	9.38	477.23	1.61	2.40	8.48
EVM0007696	7.81	8.52	7.57	33.26	17.12	23.32
EVM0012149	59.55	61.11	94.14	28.84	25.29	30.02
EVM0009698	1577.37	1174.02	1049.89	4373.42	2720.55	3909.93
EVM0006874	30.80	40.47	13.57	2.54	3.64	1.70
EVM0011734	5.99	6.18	2.25	14.73	20.81	7.59
EVM0010265	33.25	43.39	35.36	113.06	108.98	80.93
EVM0008481	11.72	10.28	7.22	28.45	29.48	38.97
EVM0001981	5.86	72.41	378.57	26.02	10.40	22.43
EVM0004142	1008.37	565.17	1201.01	56.52	57.81	54.82
EVM0001277	4.40	4.91	5.83	22.39	25.03	21.51
EVM0012233	1.25	12.95	79.77	3.21	3.31	5.11
EVM0000932	98.37	62.30	56.84	271.11	216.15	252.19
EVM0008213	1093.56	778.98	798.91	2286.68	2190.78	2780.10

EVM0002372	6.14	8.81	12.43	24.88	27.99	50.32
EVM0011950	601.16	577.86	418.92	2042.67	2141.59	1347.21
EVM0001725	24.27	16.94	9.80	49.91	35.37	32.87
EVM0008881	231.32	197.99	317.37	65.30	60.98	79.47
EVM0006028	235.04	88.99	26.35	592.51	581.35	235.43
EVM0004082	2.07	2.45	3.29	0.58	1.01	0.58
EVM0004026	32.57	921.67	1301.47	73.39	92.37	162.68
EVM0005121	147.61	166.37	190.59	469.78	418.83	448.80
EVM0010213	7709.96	1148.45	681.01	2576.26	2672.67	3955.13
EVM0000202	148.92	149.45	271.29	47.18	36.66	42.39
EVM0004221	7.22	6.81	1.70	44.37	40.02	207.12
EVM0012007	20.42	17.93	21.95	56.96	61.23	72.15
EVM0008313	21.42	24.55	27.63	10.18	9.35	13.63
EVM0001525	32.01	33.65	27.70	100.94	69.49	67.45
EVM0003267	9620.57	7123.21	1260.86	46550.10	36948.10	34941.10
EVM0004698	81.53	65.13	60.10	230.51	256.36	326.74
EVM0007377	10.04	11.94	9.47	26.93	27.15	26.23
EVM0011226	140.90	121.74	128.12	298.96	318.86	339.63
EVM0011879	38.22	18.02	42.12	85.35	107.65	185.59
EVM0010715	73.74	53.79	38.90	164.65	145.51	155.98
EVM0011088	21.26	14.00	20.87	85.72	55.00	98.79
EVM0002681	11.19	12.43	10.79	33.61	28.42	35.29
EVM0005169	1355.12	1103.72	4332.32	206.52	164.25	674.83
EVM0000883	4.16	3.80	3.86	14.72	11.15	10.79
EVM0002674	9.74	8.85	4.93	1.52	2.34	1.53
EVM0002912	170.90	156.77	152.57	655.95	540.40	503.86

EVM0008150	5.20	41.66	80.30	31.31	16.20	15.36
EVM0008211	14.80	17.24	13.55	36.44	35.84	28.25
EVM0008292	16.07	16.60	12.39	43.43	37.24	37.02
EVM0003945	216.14	205.26	180.22	101.73	85.30	76.53
EVM0004809	43.74	40.01	39.76	127.43	118.60	137.53
EVM0000393	940.82	461.63	281.92	3545.94	2377.79	1992.78
EVM0001579	37.82	175.78	1118.44	10.27	33.77	11.76
EVM0001519	7.75	8.33	8.66	29.64	39.11	40.24
EVM0005377	4.34	6.19	7.43	18.99	16.93	15.72
EVM0009623	7.20	26.53	30.69	310.96	208.31	548.84
EVM0002451	2.49	2.84	2.95	20.67	20.22	25.34
EVM0006992	9.57	12.70	22.34	43.56	46.74	56.72
EVM0005649	178.28	153.19	123.43	479.93	407.26	430.64
EVM0009546	73.84	97.32	270.71	41.52	42.58	58.81
EVM0002754	7.81	10.25	17.95	64.06	69.76	71.17
EVM0002660	9.50	8.84	7.30	48.78	33.00	36.65
EVM0010935	21.44	16.37	16.67	76.02	72.16	106.17
EVM0003967	141.19	119.12	102.09	337.15	286.42	268.77
EVM0005254	38.61	26.67	36.33	209.01	144.42	238.27
EVM0003951	36.10	36.31	58.59	119.44	124.85	148.43
EVM0009347	15.04	16.22	15.58	71.76	52.85	38.60
EVM0003465	15.24	23.72	23.61	49.19	51.36	58.39
EVM0010235	2.28	3.00	1.38	4.86	6.12	4.71
EVM0002281	68.12	47.04	218.98	14.92	7.08	12.27
EVM0004156	0.66	0.96	0.74	2.67	2.60	2.20
EVM0006723	155.42	135.41	29.57	794.72	704.37	1458.58

EVM0005429	9.52	10.17	15.80	41.78	36.83	39.97
EVM0001247	90.27	34.90	25.31	183.43	117.26	93.81
EVM0002391	399.28	207.59	123.60	2143.23	1159.11	1386.81
EVM0000869	161.53	219.77	474.64	545.40	768.15	1314.67
EVM0009500	100.93	121.52	155.93	48.23	51.03	54.85
EVM0005306	44.42	52.40	41.70	117.29	114.02	121.68
EVM0002711	20.51	17.89	15.39	85.56	93.88	103.19
EVM0006159	40.98	47.41	136.24	102.02	136.47	60.24
EVM0006020	33.57	40.69	62.17	103.92	144.13	280.92
EVM0006479	40.01	34.65	19.38	7.56	9.23	7.04
EVM0000333	20.83	24.34	29.16	8.72	11.84	6.81
EVM0006914	2.53	5.67	5.04	8.65	11.63	14.55
EVM0004495	299.00	260.54	251.97	674.21	555.58	534.01
EVM0009944	7.36	8.88	6.66	22.75	23.15	19.45
EVM0007596	20.18	21.58	20.25	59.46	51.00	54.73
EVM0010264	22.62	19.41	12.22	191.62	215.88	226.65
EVM0005335	7.90	17.23	11.32	26.98	36.23	32.83
EVM0002127	55.73	38.35	36.60	115.98	103.06	121.66
EVM0010113	33.98	29.57	61.21	104.28	139.57	201.03
EVM0008730	8.47	16.51	17.63	36.22	52.29	96.77
EVM0001016	3.40	5.47	5.53	13.39	18.51	22.66
EVM0004238	22.13	23.97	23.90	80.54	73.47	60.16
EVM0009090	19.21	25.19	14.37	118.55	266.06	149.64
EVM0007527	271.98	227.68	220.25	99.60	100.76	104.46
EVM0007254	8026.47	6323.28	7240.86	36270.50	24518.90	31828.00
EVM0000530	33.76	32.20	27.77	11.51	14.44	9.76

EVM0000784	9.86	9.47	7.75	23.58	27.84	20.82
EVM0008917	21.73	19.92	21.10	55.42	48.96	72.47
EVM0006789	107.49	83.87	131.04	44.24	35.06	47.82
EVM0002798	5.25	3.75	3.13	23.12	17.23	15.83
EVM0011948	45.34	47.96	54.37	17.28	21.16	27.06
EVM0003022	7.83	448.11	4138.79	2.09	7.75	216.17
EVM0002867	30.59	34.56	34.40	124.43	117.39	87.22
EVM0002582	321.38	320.26	326.95	135.71	120.20	94.50
EVM0011435	56.24	55.96	70.56	14.19	14.72	10.88
EVM0008447	47.15	49.59	51.60	182.04	136.13	122.92
EVM0003122	41.25	43.47	53.18	14.40	16.92	13.46
EVM0005126	2.58	3.47	4.20	5.93	7.86	9.52
EVM0004911	537.24	415.95	288.88	1110.72	1480.83	1632.91
EVM0002782	27.83	25.36	34.88	12.54	9.81	13.02
EVM0009733	8.69	8.04	12.02	26.23	34.16	63.75
EVM0006949	41.28	68.14	69.91	222.96	212.58	277.54
EVM0007199	136.75	107.14	167.20	592.09	404.82	379.02
EVM0003386	4.47	12.67	5.95	12.11	26.78	12.04
EVM0011601	146.90	125.16	76.61	401.96	310.07	225.49
EVM0008827	3.30	3.91	6.39	0.96	0.95	1.59
EVM0006578	103.07	98.13	93.35	263.57	233.00	272.30
EVM0011492	260.14	336.75	1057.93	639.74	808.34	516.65
EVM0005758	66.90	58.97	38.11	152.55	176.41	241.58
EVM0000108	44.65	60.50	53.59	22.23	27.61	22.39
EVM0008637	27.01	31.67	74.73	26.63	20.78	19.82
EVM0002747	1.62	1.73	1.61	0.25	0.38	0.17

EVM0009214	135.72	152.31	176.79	481.27	512.95	393.79
EVM0007388	15.18	14.33	14.44	58.82	51.11	62.90
EVM0010500	36.53	36.63	30.57	87.67	96.06	80.45
EVM0008978	23.38	19.12	13.31	5.54	5.81	4.73
EVM0011270	10.79	9.23	6.95	34.83	27.27	39.84
EVM0010842	132.66	133.19	145.53	60.94	64.84	57.70
EVM0003732	21.94	39.73	43.85	51.82	47.85	31.36
EVM0008140	48.80	28.50	32.62	11.29	10.38	13.48
EVM0001827	2.49	3.26	3.41	11.15	11.16	11.29
EVM0004966	2.64	3.33	3.63	6.24	7.14	8.02

Data Set S7 Genes invovled in oxidoreductase activity and oxidation-reduction process

oxidoreductase activity	log2(fold change R1vsZ1)	log2(fold change R2vsZ2)	log2(fold change R2vsZ2)	R1 vs Z1 q value	R2 vs Z2 q value	R3 vs Z3 q value	annotation
EVM0000006	-0.88	-0.03	1.18	2.08E-02	9.61E-01	7.96E-04	Cytochrome P450
EVM0000040	1.16	0.61	0.15	7.96E-04	9.59E-02	7.48E-01	Flavin-binding monooxygenase-like
EVM0000093	-1.51	-0.45	-0.72	7.96E-04	3.22E-01	6.79E-02	HEAT repeats
EVM0000151	-1.77	-2.45	-1.10	4.28E-04	4.28E-04	1.01E-02	Cytochrome b5-like Heme/Steroid binding domain
EVM0000204	-1.49	-1.37	-1.96	2.36E-03	2.92E-03	4.28E-04	short chain dehydrogenase
EVM0000295	1.12	1.10	1.75	7.55E-03	5.26E-02	2.92E-03	Catalase
EVM0000375	-1.36	-0.59	-1.36	4.28E-04	1.46E-01	4.28E-04	Fatty acid hydroxylase superfamily
EVM0000503	-0.54	0.06	1.46	2.21E-01	9.21E-01	4.28E-04	lactate/malate dehydrogenase, NAD binding domain
EVM0000528	-1.04	-0.24	-0.31	3.10E-02	6.34E-01	5.51E-01	Acyl-CoA dehydrogenase, C-terminal domain
EVM0000538	-1.82	-0.75	-1.40	4.28E-04	9.63E-02	4.98E-03	Putative FMN-binding domain
EVM0000564	0.17	-0.71	-1.27	7.44E-01	5.81E-02	4.28E-04	Isocitrate/isopropylmalate dehydrogenase
EVM0000566	-0.24	0.58	3.71	6.58E-01	1.68E-01	4.28E-04	Cytochrome P450

EVM0000600	-1.01	-0.61	-1.42	4.23E-03	9.58E-02	4.28E-04	Nitronate monooxygenase
EVM0000621	0.03	0.09	1.01	9.52E-01	8.63E-01	8.44E-03	Oxidoreductase family, NAD-binding Rossmann fold
EVM0000637	-1.00	2.00	3.44	8.22E-03	4.28E-04	4.28E-04	Cytochrome P450
EVM0000680	0.61	1.42	3.84	1.78E-01	1.46E-03	4.28E-04	Cytochrome P450
EVM0000705	-0.94	-1.26	-1.78	1.30E-02	4.28E-04	4.28E-04	Iron/manganese superoxide dismutases, C-terminal domain
EVM0000711	1.10	0.91	0.91	1.14E-03	5.94E-03	6.64E-03	Cytochrome P450
EVM0000762	-1.81	-1.24	-0.64	4.28E-04	4.28E-04	1.28E-01	Protein of unknown function (DUF1295)
EVM0000773	-0.58	-1.30	-2.03	1.55E-01	7.96E-04	4.28E-04	NADH-ubiquinone oxidoreductase B18 subunit (NDUFB7)
EVM0000781	-0.51	0.10	2.18	2.22E-01	8.52E-01	4.28E-04	FAD dependent oxidoreductase
EVM0000804	-0.63	-0.11	-1.67	1.43E-01	8.33E-01	4.28E-04	Acyl-CoA dehydrogenase, C-terminal domain
EVM0000833	0.79	0.27	3.16	2.31E-02	5.36E-01	4.28E-04	Isocitrate/isopropylmalate dehydrogenase
EVM0000891	-0.43	0.56	1.21	3.12E-01	1.40E-01	4.28E-04	Pyridoxamine 5'-phosphate oxidase
EVM0000944	-0.04	-0.45	2.13	9.49E-01	2.57E-01	4.28E-04	Aldo/keto reductase family
EVM0000973	0.82	0.26	2.36	7.90E-02	6.46E-01	4.28E-04	FMN-dependent dehydrogenase
EVM0001011	-0.64	1.15	-0.43	6.63E-02	1.14E-03	3.35E-01	Cytochrome P450
EVM0001128	-0.14	1.53	0.07	8.27E-01	4.28E-04	9.11E-01	Cytochrome P450
EVM0001166	0.02	0.77	2.21	9.76E-01	3.76E-02	4.28E-04	Cytochrome P450
EVM0001244	-0.53	-0.54	-1.42	2.63E-01	2.30E-01	3.19E-03	Dyp-type peroxidase family
EVM0001285	0.27	-0.31	2.15	5.57E-01	4.79E-01	4.28E-04	Alternative oxidase
EVM0001322	0.80	0.95	1.20	2.22E-02	3.98E-03	2.36E-03	Cytochrome P450
EVM0001489	1.00	1.39	3.44	4.73E-03	4.28E-04	4.28E-04	Enoyl-(Acyl carrier protein) reductase
EVM0001579	1.88	2.38	6.57	4.28E-04	4.28E-04	4.28E-04	Family of unknown function (DUF706)
EVM0001740	-1.94	0.00	1.01	4.28E-04	9.95E-01	6.64E-03	2OG-Fe(II) oxygenase superfamily
EVM0001774	0.48	0.64	1.25	2.90E-01	1.25E-01	2.36E-03	Cytochrome P450

EVM0001778	-1.54	-1.61	-1.80	4.28E-04	4.28E-04	4.28E-04	Pyridoxamine 5'-phosphate oxidase
EVM0001859	-0.20	-0.37	1.01	6.56E-01	4.22E-01	1.68E-02	D-isomer specific 2-hydroxyacid dehydrogenase, NAD binding domain
EVM0001905	-0.92	0.53	2.57	5.70E-03	2.14E-01	4.28E-04	GMC oxidoreductase
EVM0001912	0.21	0.62	1.09	6.33E-01	9.07E-02	2.36E-03	EPSP synthase (3-phosphoshikimate 1-carboxyvinyltransferase)
EVM0002090	0.33	0.07	1.19	4.27E-01	8.87E-01	4.28E-04	Taurine catabolism dioxygenase TauD, TfdA family
EVM0002137	1.16	0.51	1.35	5.70E-03	2.15E-01	7.96E-04	D-arabinono-1,4-lactone oxidase
EVM0002177	-0.40	-0.16	-1.71	3.20E-01	7.32E-01	4.28E-04	short chain dehydrogenase
EVM0002201	-0.95	0.15	1.65	1.77E-03	7.48E-01	4.28E-04	Aldehyde dehydrogenase family
EVM0002262	-0.23	-1.06	-0.14	6.48E-01	1.87E-02	8.17E-01	Oxoglutarate and iron-dependent oxygenase degradation C-term
EVM0002281	2.19	2.73	4.16	4.28E-04	4.28E-04	4.28E-04	--
EVM0002287	2.11	-0.13	-1.54	1.51E-02	8.71E-01	9.73E-03	Cytochrome P450
EVM0002309	-0.28	-0.97	-1.20	4.96E-01	3.46E-03	4.28E-04	NAD-dependent glycerol-3-phosphate dehydrogenase C-terminus
EVM0002372	-2.02	-1.67	-2.02	4.28E-04	4.28E-04	4.28E-04	Cytochrome P450
EVM0002430	1.10	0.46	0.77	3.19E-03	2.63E-01	4.26E-02	Dehydrogenase E1 component
EVM0002432	-1.52	0.48	-0.66	6.64E-03	4.60E-01	2.80E-01	Cytochrome P450
EVM0002521	-0.95	-0.77	-1.17	1.32E-02	4.16E-02	7.96E-04	Acyl-CoA dehydrogenase, C-terminal domain
EVM0002562	-0.92	-0.33	2.38	6.17E-03	4.50E-01	4.28E-04	AhpC/TSA family
EVM0002722	-1.79	-0.66	-0.67	4.28E-04	1.06E-01	1.07E-01	Multicopper oxidase
EVM0002729	0.09	-0.38	-2.16	8.72E-01	3.78E-01	4.28E-04	Ergosterol biosynthesis ERG4/ERG24 family
EVM0002811	-0.49	-1.25	-2.09	3.89E-01	3.46E-03	4.28E-04	Thioredoxin
EVM0002854	-1.06	1.83	4.08	2.07E-03	4.28E-04	4.28E-04	Multicopper oxidase
EVM0002892	-0.93	-0.80	-1.03	1.16E-02	3.20E-02	7.10E-03	FAD binding domain

EVM0002918	0.47	1.06	1.57	2.72E-01	1.77E-03	4.28E-04	Cytochrome P450
EVM0003014	0.62	1.07	1.63	7.91E-02	1.01E-02	4.28E-04	Methylenetetrahydrofolate reductase
EVM0003022	1.91	5.85	4.26	4.28E-04	4.28E-04	4.28E-04	FAD dependent oxidoreductase
EVM0003034	0.38	0.90	1.32	3.13E-01	2.03E-02	2.07E-03	Formate--tetrahydrofolate ligase
EVM0003191	0.98	0.28	1.28	4.98E-03	5.14E-01	2.92E-03	Cytochrome P450
EVM0003237	0.12	1.47	2.85	8.12E-01	4.28E-04	4.28E-04	Glutamate/Leucine/Phenylalanine/Valine dehydrogenase
EVM0003243	0.77	0.89	2.38	2.70E-02	5.94E-03	4.28E-04	AMP-binding enzyme
EVM0003304	1.48	0.58	0.69	2.29E-02	3.28E-01	3.11E-01	Cytochrome P450
EVM0003346	-1.43	-0.11	-0.55	7.96E-04	8.26E-01	1.68E-01	Nitronate monooxygenase
EVM0003361	-2.68	-2.67	-0.84	4.28E-04	4.28E-04	1.81E-01	Lipoxygenase
EVM0003398	0.78	0.19	-1.54	1.00E+00	8.28E-01	4.07E-02	Cytochrome P450
EVM0003471	-0.03	1.43	0.79	9.68E-01	2.92E-03	1.08E-01	GMC oxidoreductase
EVM0003552	-0.52	-0.96	-1.14	1.49E-01	4.73E-03	1.14E-03	Enoyl-(Acyl carrier protein) reductase
EVM0003609	-1.40	-1.80	-2.78	4.28E-04	4.28E-04	4.28E-04	Cytochrome oxidase c subunit VIb
EVM0003803	-1.32	0.54	0.26	1.34E-02	2.70E-01	6.09E-01	NADH:flavin oxidoreductase / NADH oxidase family
EVM0003814	-0.88	5.08	3.29	1.01E-01	4.28E-04	1.46E-03	GMC oxidoreductase
EVM0003882	-1.41	-0.92	1.24	4.28E-04	5.94E-03	7.96E-04	Pyridine nucleotide-disulphide oxidoreductase
EVM0003917	0.90	0.15	1.73	1.62E-01	8.50E-01	2.36E-03	Aldehyde dehydrogenase family
EVM0003929	1.84	1.36	1.37	4.28E-04	1.14E-03	7.96E-04	Taurine catabolism dioxygenase TauD, TfdA family
EVM0003976	1.75	1.80	1.59	4.28E-04	4.28E-04	4.28E-04	Conserved region in glutamate synthase
EVM0004000	0.03	1.45	3.28	9.52E-01	4.28E-04	4.28E-04	Glucose-6-phosphate dehydrogenase, C-terminal domain
EVM0004021	0.27	0.60	1.97	5.19E-01	1.17E-01	4.28E-04	Cytochrome P450
EVM0004069	0.39	1.00	0.29	3.25E-01	3.46E-03	4.85E-01	FAD linked oxidases, C-terminal domain
EVM0004108	-1.17	-0.51	-0.77	6.41E-03	2.91E-01	7.94E-02	Cytochrome P450

EVM0004177	0.93	1.22	2.66	7.77E-03	3.98E-03	4.28E-04	Nitrite and sulphite reductase 4Fe-4S domain
EVM0004215	-0.59	-1.54	-1.65	1.02E-01	4.28E-04	4.28E-04	Cytochrome c oxidase subunit Va
EVM0004219	-0.34	2.03	2.28	3.89E-01	4.28E-04	4.28E-04	NADH:flavin oxidoreductase / NADH oxidase family
EVM0004285	0.01	1.57	1.39	9.87E-01	4.28E-04	4.28E-04	Multicopper oxidase
EVM0004300	0.10	3.62	2.87	9.15E-01	4.28E-04	4.28E-04	Cytochrome P450
EVM0004320	1.19	1.32	1.11	7.96E-04	4.28E-04	4.28E-04	Aldo/keto reductase family
EVM0004345	-1.42	-1.23	-0.54	4.28E-04	1.14E-03	1.42E-01	Taurine catabolism dioxygenase TauD, TfdA family
EVM0004424	0.25	0.38	1.41	5.55E-01	3.28E-01	4.28E-04	Histidinol dehydrogenase
EVM0004456	-1.27	-1.25	-1.71	6.50E-02	8.91E-02	2.94E-02	Alcohol dehydrogenase GroES-like domain
EVM0004461	-1.33	-2.40	-1.35	4.28E-04	4.28E-04	4.28E-04	Putative oxidoreductase C terminal
EVM0004488	-0.69	-0.67	-3.77	6.58E-02	8.95E-02	4.28E-04	Multicopper oxidase
EVM0004518	-1.04	-0.78	1.46	1.08E-02	6.28E-02	4.28E-04	Aldehyde dehydrogenase family
EVM0004655	0.25	0.81	1.77	6.09E-01	2.98E-02	4.28E-04	Methylenetetrahydrofolate reductase
EVM0004661	-1.20	-1.32	-1.51	2.07E-03	1.77E-03	4.28E-04	Cytochrome P450
EVM0004712	0.21	0.98	2.35	6.95E-01	1.06E-02	4.28E-04	short chain dehydrogenase
EVM0004732	0.72	-0.44	1.19	5.01E-02	2.49E-01	4.28E-04	Acyl-CoA dehydrogenase, C-terminal domain
EVM0004740	-0.43	0.10	1.57	3.08E-01	8.46E-01	4.28E-04	Iron-containing alcohol dehydrogenase
EVM0004894	-0.62	-0.18	1.22	9.74E-02	7.10E-01	4.28E-04	FAD binding domain
EVM0005148	0.05	0.05	1.41	9.19E-01	9.29E-01	4.28E-04	6-phosphogluconate dehydrogenase, C-terminal domain
EVM0005231	-0.41	1.33	2.79	3.80E-01	7.96E-04	4.28E-04	FAD dependent oxidoreductase
EVM0005275	-1.34	-0.96	-1.01	2.08E-02	6.50E-02	6.15E-02	FAD binding domain
EVM0005310	0.65	1.92	2.03	1.00E-01	4.28E-04	4.28E-04	Enoyl-(Acyl carrier protein) reductase
EVM0005316	0.78	0.76	1.93	3.30E-01	2.66E-01	3.58E-02	Dioxygenase
EVM0005338	-1.28	-0.34	0.12	4.28E-04	4.14E-01	8.27E-01	Zinc-binding dehydrogenase
EVM0005390	0.26	-0.36	-1.06	5.39E-01	3.65E-01	7.96E-04	short chain dehydrogenase
EVM0005468	-0.48	0.06	1.52	3.02E-01	9.16E-01	4.28E-04	Pyrroline-5-carboxylate reductase dimerisation

EVM0005495	-0.10	0.85	1.57	9.13E-01	1.54E-01	6.87E-03	Ketopantoate reductase PanE/ApbA C terminal
EVM0005518	-1.19	-0.46	-1.17	4.63E-02	4.33E-01	3.18E-02	non-haem dioxygenase in morphine synthesis N-terminal
EVM0005567	1.92	0.79	0.18	4.28E-04	4.42E-02	7.38E-01	Ribonucleotide reductase, small chain
EVM0005612	-0.11	0.38	1.11	8.30E-01	3.39E-01	7.96E-04	Aldehyde dehydrogenase family
EVM0005631	-0.93	-0.46	-1.77	1.44E-01	4.49E-01	1.08E-02	Domain of unknown function (DUF3415)
EVM0005691	-1.83	-1.89	-2.61	5.94E-03	1.47E-02	1.46E-03	Cytochrome P450
EVM0005762	0.01	-0.69	-1.40	9.82E-01	5.61E-02	4.28E-04	Thioredoxin
EVM0005768	0.41	-0.86	-1.19	3.93E-01	3.41E-02	2.07E-03	Peroxidase
EVM0005773	0.28	1.11	0.41	4.88E-01	4.28E-04	2.85E-01	Cytochrome P450
EVM0005861	-1.52	-2.23	-4.41	1.14E-03	4.28E-04	4.28E-04	Catalase
EVM0005891	-1.32	-1.88	-0.63	4.28E-04	4.28E-04	8.10E-02	Pyridine nucleotide-disulphide oxidoreductase
EVM0005925	-0.27	1.85	1.69	5.51E-01	4.28E-04	4.28E-04	Cytochrome P450
EVM0005970	-1.01	0.51	1.89	1.28E-02	2.10E-01	4.28E-04	3-hydroxyanthranilic acid dioxygenase
EVM0005975	0.00	0.46	1.63	9.94E-01	2.10E-01	4.28E-04	Flavin-binding monooxygenase-like
EVM0006055	-1.25	1.12	2.89	5.65E-02	3.04E-02	4.28E-04	short chain dehydrogenase
EVM0006084	-0.91	0.38	2.99	2.20E-02	3.64E-01	4.28E-04	Alcohol dehydrogenase GroES-like domain
EVM0006117	-1.80	-1.11	-1.42	4.28E-04	3.72E-03	4.28E-04	Aldo/keto reductase family
EVM0006352	-0.58	1.37	3.31	1.89E-01	4.28E-04	4.28E-04	short chain dehydrogenase
EVM0006432	-1.67	0.89	1.82	4.28E-04	3.95E-02	4.28E-04	Fatty acid desaturase
EVM0006458	-1.23	-0.45	-0.36	4.28E-04	2.42E-01	3.70E-01	Cytochrome P450
EVM0006467	-0.65	0.21	1.24	1.05E-01	6.65E-01	7.96E-04	Zinc-binding dehydrogenase
EVM0006502	-1.14	-1.14	-0.56	4.28E-04	7.96E-04	1.24E-01	Alcohol dehydrogenase GroES-like domain
EVM0006570	-2.17	-1.94	-2.35	4.28E-04	4.28E-04	4.28E-04	Pyridoxamine 5'-phosphate oxidase
EVM0006605	0.23	0.15	1.56	6.09E-01	7.68E-01	4.28E-04	Alcohol dehydrogenase GroES-like domain
EVM0006652	-1.14	0.56	1.34	1.14E-03	1.22E-01	7.96E-04	Cytochrome P450

EVM0006662	-1.63	-0.55	-0.28	4.28E-04	1.74E-01	5.18E-01	Hydroxymethylglutaryl-coenzyme A reductase
EVM0006670	-2.87	-0.07	-2.52	1.14E-01	9.55E-01	4.98E-02	Cytochrome P450
EVM0006718	-0.27	1.25	0.74	5.78E-01	4.23E-03	2.25E-01	FAD dependent oxidoreductase
EVM0006753	-1.68	-0.13	2.96	4.28E-04	8.34E-01	4.28E-04	Family of unknown function (DUF706)
EVM0006754	0.17	-1.59	-0.19	7.35E-01	4.28E-04	7.93E-01	Coproporphyrinogen III oxidase
EVM0006768	-0.49	-1.25	0.42	2.10E-01	4.28E-04	2.80E-01	Cytochrome P450
EVM0006770	-2.56	0.13	-0.29	4.28E-04	8.52E-01	5.71E-01	Domain of unknown function (DUF3415)
EVM0006858	-0.79	0.65	2.02	2.24E-02	8.43E-02	4.28E-04	Aldehyde dehydrogenase family
EVM0006901	0.94	-0.08	2.13	6.64E-03	8.77E-01	4.28E-04	Acetohydroxy acid isomeroreductase, catalytic domain
EVM0006992	-2.19	-1.88	-1.34	4.28E-04	4.28E-04	4.28E-04	Cytochrome P450
EVM0007015	1.66	0.94	1.36	4.28E-04	1.22E-02	4.28E-04	Acyl transferase domain
EVM0007048	-1.00	0.06	3.32	2.93E-02	9.25E-01	4.28E-04	--
EVM0007059	-0.92	-0.34	2.93	6.53E-02	5.05E-01	4.28E-04	Domain of unknown function (DUF3415)
EVM0007176	0.48	-0.48	-1.64	1.96E-01	2.15E-01	4.28E-04	D-isomer specific 2-hydroxyacid dehydrogenase, NAD binding domain
EVM0007202	-2.74	-0.13	-1.07	4.28E-04	8.48E-01	2.56E-02	non-haem dioxygenase in morphine synthesis N-terminal
EVM0007311	-0.90	0.57	1.66	1.30E-02	1.22E-01	4.28E-04	Cytochrome P450
EVM0007315	-0.07	0.06	1.25	8.86E-01	9.09E-01	4.28E-04	Homoserine dehydrogenase
EVM0007336	1.59	0.11	0.74	4.28E-04	8.19E-01	5.69E-02	AhpC/TSA family
EVM0007461	-2.31	-0.74	-0.70	4.28E-04	8.26E-02	1.11E-01	Aldo/keto reductase family
EVM0007494	-0.38	-0.60	-1.31	3.21E-01	9.65E-02	4.28E-04	Glutaredoxin
EVM0007521	-0.07	-0.03	-1.10	8.85E-01	9.56E-01	3.19E-03	Cytochrome P450
EVM0007795	-1.01	0.09	1.52	3.46E-03	8.62E-01	4.28E-04	Cytochrome P450
EVM0007958	-1.81	-1.78	-3.13	4.28E-04	4.28E-04	4.28E-04	Multicopper oxidase
EVM0008002	-3.30	-1.47	0.74	4.28E-04	4.28E-04	6.32E-02	Flavodoxin

EVM0008061	0.34	1.28	1.63	4.15E-01	4.28E-04	4.28E-04	GMC oxidoreductase
EVM0008150	-2.59	1.36	2.39	4.28E-04	4.23E-03	4.28E-04	Aldo/keto reductase family
EVM0008238	-0.36	0.53	2.66	4.41E-01	2.61E-01	4.28E-04	Cytochrome P450
EVM0008251	-0.11	0.29	-1.01	8.19E-01	4.89E-01	7.96E-04	Aldo/keto reductase family
EVM0008259	1.66	1.24	1.52	4.28E-04	4.23E-03	7.96E-04	Putative oxidoreductase C terminal
EVM0008262	-1.05	-0.76	0.69	1.77E-03	3.31E-02	5.32E-02	NAD binding domain of 6-phosphogluconate dehydrogenase
EVM0008303	-0.51	1.83	5.48	4.68E-01	1.96E-02	2.74E-01	short chain dehydrogenase
EVM0008324	-1.77	0.66	2.80	4.28E-04	1.85E-01	4.28E-04	Cytochrome P450
EVM0008383	-0.17	-0.81	-1.91	7.16E-01	2.37E-02	4.28E-04	NADH ubiquinone oxidoreductase subunit NDUFA12
EVM0008497	-1.34	-1.06	-1.98	5.22E-03	1.14E-02	4.28E-04	MaoC like domain
EVM0008503	-0.81	0.34	1.13	8.50E-02	4.96E-01	5.22E-03	short chain dehydrogenase
EVM0008522	1.56	0.34	0.56	4.28E-04	3.90E-01	1.28E-01	FAD dependent oxidoreductase
EVM0008582	-0.98	-0.03	1.96	3.64E-02	9.58E-01	4.28E-04	short chain dehydrogenase
EVM0008651	-1.04	-1.37	-1.53	1.77E-03	4.28E-04	4.28E-04	Copper/zinc superoxide dismutase (SODC)
EVM0008666	0.03	1.32	1.21	9.61E-01	4.28E-04	1.14E-03	Dioxygenase
EVM0008720	1.34	1.19	1.55	4.28E-04	1.14E-03	4.28E-04	Ribonucleotide reductase, barrel domain
EVM0008803	-1.45	-0.71	0.42	4.28E-04	3.89E-02	2.82E-01	FAD binding domain
EVM0008889	-1.80	-2.79	-1.90	8.22E-03	5.22E-03	2.36E-03	Cytochrome P450
EVM0008945	0.56	0.58	1.29	1.40E-01	1.20E-01	4.28E-04	Pyridine nucleotide-disulphide oxidoreductase
EVM0008970	-0.42	1.80	2.68	3.14E-01	4.28E-04	4.28E-04	Cytochrome P450
EVM0009164	-0.79	0.16	1.97	2.70E-01	8.31E-01	2.01E-02	Aldehyde dehydrogenase family
EVM0009294	-0.35	-0.51	-1.43	4.33E-01	2.17E-01	4.28E-04	Fatty acid hydroxylase superfamily
EVM0009319	0.41	1.03	1.70	5.98E-01	2.66E-01	4.28E-04	Cytochrome P450
EVM0009384	0.83	0.46	1.75	1.32E-02	2.10E-01	4.28E-04	Isocitrate/isopropylmalate dehydrogenase

EVM0009514	1.09	0.14	1.32	7.96E-04	7.66E-01	4.28E-04	Glyceraldehyde 3-phosphate dehydrogenase, C-terminal domain
EVM0009547	-0.80	-0.51	-2.15	4.10E-02	2.27E-01	4.28E-04	Cytochrome P450
EVM0009549	-0.48	0.28	1.09	2.45E-01	5.42E-01	1.77E-03	Zinc-binding dehydrogenase
EVM0009575	-0.23	-0.58	-1.05	5.88E-01	9.72E-02	1.14E-03	Acyl-CoA dehydrogenase, C-terminal domain
EVM0009632	0.01	1.40	1.33	9.86E-01	7.96E-04	4.28E-04	Copper amine oxidase, enzyme domain
EVM0009683	-0.16	0.09	2.06	7.55E-01	8.52E-01	4.28E-04	Cytochrome P450
EVM0009692	-1.44	-0.01	0.60	4.28E-04	9.84E-01	1.06E-01	Aldo/keto reductase family
EVM0009701	-0.97	0.83	2.95	1.32E-02	4.07E-02	4.28E-04	Glutathione peroxidase
EVM0009755	-0.60	-0.47	-1.67	2.39E-01	4.09E-01	4.28E-04	Cytochrome P450
EVM0009784	0.14	1.50	4.98	8.47E-01	3.46E-03	4.28E-04	D-isomer specific 2-hydroxyacid dehydrogenase, NAD binding domain
EVM0010016	0.09	-0.31	1.04	8.56E-01	4.62E-01	2.65E-03	Oxidoreductase family, NAD-binding Rossmann fold
EVM0010226	-1.37	-0.64	1.32	4.28E-04	1.19E-01	1.14E-03	Iron/manganese superoxide dismutases, C-terminal domain
EVM0010256	-2.93	-1.02	-0.82	4.28E-04	2.67E-02	9.00E-02	GMC oxidoreductase
EVM0010470	1.65	1.38	1.20	4.28E-04	4.28E-04	4.28E-04	Proteasome/cyclosome repeat
EVM0010473	-0.83	1.51	3.49	2.56E-02	4.28E-04	4.28E-04	Multicopper oxidase
EVM0010485	-0.64	-1.17	-2.45	6.50E-02	4.28E-04	4.28E-04	Cytochrome P450
EVM0010517	-1.23	-0.05	0.04	4.28E-04	9.30E-01	9.33E-01	SelR domain
EVM0010628	0.04	-0.04	2.33	9.55E-01	9.54E-01	4.28E-04	Taurine catabolism dioxygenase TauD, TfdA family
EVM0010699	-0.59	2.41	2.49	1.09E-01	1.20E-02	4.28E-04	Aldo/keto reductase family
EVM0010749	-1.23	0.12	1.44	1.40E-02	8.55E-01	2.92E-03	Cytochrome P450
EVM0010820	-0.54	-1.89	-2.58	1.47E-01	4.28E-04	4.28E-04	Cytochrome oxidase c subunit VIb
EVM0010845	-1.30	-0.28	-0.37	4.52E-02	6.89E-01	5.91E-01	Domain of unknown function (DUF3415)
EVM0010922	-0.72	-1.21	-1.57	7.54E-02	7.96E-04	4.28E-04	Nitronate monooxygenase

EVM0010946	-1.16	-0.18	-0.23	4.63E-02	7.93E-01	7.06E-01	Pyridine nucleotide-disulphide oxidoreductase
EVM0010988	-1.24	0.14	0.47	4.28E-04	7.86E-01	2.60E-01	Alcohol dehydrogenase GroES-like domain
EVM0011026	-0.65	-0.99	-1.16	5.60E-02	2.36E-03	4.28E-04	Semialdehyde dehydrogenase, dimerisation domain
EVM0011062	-1.15	-1.07	0.45	2.07E-03	1.46E-03	2.41E-01	lactate/malate dehydrogenase, NAD binding domain
EVM0011151	0.05	1.06	1.38	9.30E-01	3.98E-03	4.28E-04	Cytochrome P450
EVM0011181	-1.48	-0.99	-0.89	4.28E-04	9.08E-03	2.13E-02	GMC oxidoreductase
EVM0011216	0.51	0.16	1.51	1.64E-01	7.31E-01	4.28E-04	Oxidoreductase family, NAD-binding Rossmann fold
EVM0011238	1.18	0.67	-1.85	7.96E-04	5.63E-02	4.28E-04	Cytochrome P450
EVM0011287	-1.14	0.01	1.73	2.92E-03	9.84E-01	4.28E-04	Cytochrome P450
EVM0011343	-1.31	-2.00	-4.05	4.28E-04	4.28E-04	4.28E-04	Glutaredoxin
EVM0011359	-0.83	-1.30	-2.20	1.44E-02	4.28E-04	4.28E-04	ETC complex I subunit conserved region
EVM0011379	-1.82	-0.91	-0.40	4.28E-04	1.06E-02	3.21E-01	non-haem dioxygenase in morphine synthesis N-terminal
EVM0011422	0.46	-1.11	1.57	2.58E-01	2.36E-03	6.86E-02	Alcohol dehydrogenase GroES-like domain
EVM0011443	0.11	-0.16	1.20	8.15E-01	7.42E-01	4.28E-04	Saccharopine dehydrogenase
EVM0011505	-0.08	-1.19	-1.78	9.26E-01	6.70E-02	3.95E-02	Cytochrome P450
EVM0011552	-1.30	-0.94	-1.13	4.28E-04	7.55E-03	2.65E-03	Fatty acid desaturase
EVM0011559	-1.16	0.04	0.10	2.07E-03	9.35E-01	8.43E-01	FAD linked oxidases, C-terminal domain
EVM0011689	-1.13	-0.09	0.08	3.72E-03	8.78E-01	8.94E-01	non-haem dioxygenase in morphine synthesis N-terminal
EVM0011731	0.80	-0.66	-2.34	5.55E-02	9.27E-02	4.28E-04	Peroxidase, family 2
EVM0011732	-0.28	-0.52	2.54	6.07E-01	2.72E-01	4.28E-04	homogentisate 1,2-dioxygenase
EVM0011888	-1.33	-0.83	-1.45	4.01E-02	1.85E-01	2.82E-02	Peroxidase
EVM0011894	-0.08	3.47	4.18	8.80E-01	4.28E-04	4.28E-04	Cytochrome P450
EVM0011921	-0.93	-0.95	-1.21	1.04E-01	6.38E-02	8.00E-03	Aldehyde dehydrogenase family
EVM0011981	0.25	0.23	1.37	5.88E-01	6.37E-01	4.28E-04	Aldehyde dehydrogenase family

EVM0012038	-0.41	-0.62	-1.83	3.27E-01	9.94E-02	4.28E-04	3-beta hydroxysteroid dehydrogenase/isomerase family
EVM0012096	0.01	-0.05	2.21	9.93E-01	9.45E-01	4.28E-04	Glyceraldehyde 3-phosphate dehydrogenase, C-terminal domain
EVM0012116	-1.95	-1.32	0.87	4.28E-04	4.28E-04	1.28E-02	Redoxin
EVM0012120	-0.57	-0.84	-1.70	1.63E-01	2.27E-02	4.28E-04	3-hydroxyacyl-CoA dehydrogenase, NAD binding domain
EVM0012216	0.23	-0.75	1.18	5.91E-01	2.74E-02	4.28E-04	short chain dehydrogenase
oxidation-reduction process							
EVM0000868	1.24	0.37	1.36	4.28E-04	3.35E-01	4.28E-04	Aconitase family (aconitate hydratase)
EVM0000933	3.04	1.94	2.40	4.28E-04	7.96E-04	4.28E-04	Malic enzyme, NAD binding domain
EVM0001216	-0.72	0.33	1.26	5.41E-02	4.24E-01	4.28E-04	Citrate synthase
EVM0001679	0.62	0.38	1.21	1.40E-01	4.04E-01	2.92E-03	Alpha amylase, C-terminal all-beta domain
EVM0001730	0.05	0.07	2.91	9.33E-01	8.99E-01	4.28E-04	Malate synthase
EVM0001902	-1.09	-0.71	-0.57	1.14E-03	4.01E-02	1.12E-01	Lyase
EVM0004079	-0.95	0.53	1.01	9.02E-02	2.30E-01	1.30E-02	Phosphoadenosine phosphosulfate reductase family
EVM0004970	0.18	0.26	1.06	6.73E-01	5.26E-01	5.70E-03	Malic enzyme, NAD binding domain
EVM0005877	-0.77	-1.24	-1.14	2.80E-02	4.28E-04	7.96E-04	Ubiquinol-cytochrome C reductase complex 14kD subunit
EVM0006700	-2.04	-1.97	-1.43	4.28E-04	4.28E-04	1.14E-03	Aconitase family (aconitate hydratase)
EVM0007606	-0.59	-0.04	1.54	1.41E-01	9.49E-01	4.28E-04	Malic enzyme, NAD binding domain
EVM0011448	0.48	0.62	1.64	1.92E-01	7.82E-02	4.28E-04	glucanotransferase domain of human glycogen debranching enzyme
EVM0011502	0.78	0.72	1.13	3.29E-02	4.94E-02	1.14E-03	Citrate synthase
